# Niche breadth and biodiversity change derived from marine Amphipoda species off Iceland

**DOI:** 10.1002/ece3.8802

**Published:** 2022-04-06

**Authors:** Anne‐Nina Lörz, Jens Oldeland, Stefanie Kaiser

**Affiliations:** ^1^ Institute for Marine Ecosystems and Fisheries Science Center for Earth System Research and Sustainability (CEN) Universität Hamburg Hamburg Germany; ^2^ Eco‐Systems Hamburg Germany; ^3^ Department of Invertebrate Zoology and Hydrobiology Faculty of Biology and Environmental Protection University of Łódź Łódź Poland; ^4^ INES Integrated Environmental Solutions UG Wilhelmshaven Germany

**Keywords:** climate change, distributional shifts, Huisman‐Olff‐Fresco modelling, marine Amphipoda, Nordic seas, range–occupancy relationship, species level

## Abstract

Understanding the ecological requirements and thresholds of individual species is crucial to better predict potential outcomes of climate change on species distribution. In particular, species optima and lower and upper limits along resource gradients require attention. Based on Huisman‐Olff‐Fresco (HOF) models, we determined species‐specific responses along gradients of nine environmental parameters including depth in order to estimate niche attributes of 30 deep‐sea benthic amphipods occurring around Iceland. We, furthermore, examined the relationships between niche breadth, occupancy, and geographic range assuming that species with a wider niche are spatially more widely dispersed and vice versa. Overall, our results reveal that species react very differently to environmental gradients, which is independent of the family affiliation of the respective species. We could infer a strong relationship between occupancy and geographic range and also relate this to differences in niche breadth; that is specialist species with a narrow niche had a more limited distribution and may thus be more threatened by changing environmental conditions than generalist species, which are more widespread. Given the preponderance of rare species in the deep sea, this implies that many species could be at risk. However, this must be carefully weighed against geographical data gaps in this area, given that many deep‐sea areas are severely undersampled and the true distribution of most species is unknown. After all, our results underline that an accurate taxonomic classification is of crucial importance, without which ecological niche properties cannot be determined and which is hence fundamental for the assessment and understanding of changes in biodiversity in the face of increasing human perturbations.

## Graphical abstract

In this study, we used HOF models for the first time to calculate species response curves and corresponding niche attributes for marine amphipods. We could show responses were species‐specific and could not be transferred to other members of the same family.

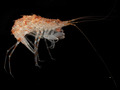

## 1 INTRODUCTION

### 1.1 Effects of climate change on marine environments

Due to increasing atmospheric carbon dioxide (CO_2_) partial pressure, it is forecast that global atmospheric temperatures will rise in the range of 2.0 and 4.5°C by 2100 (IPCC, 2007, 2021) and thus also in the sea. In addition, the absorption of CO_2_ by the ocean will lead to changes in ocean geochemistry, above all acidification and a decline in calcium carbonate saturation (Orr et al., 2005), with profound risks for the marine biota. The direct effects of increased CO_2_ emissions—ocean warming and acidification—cause a myriad of indirect effects, including freshening due to melting ice caps and shrinking sea ice at the poles and associated increased solar UV light penetration, greater stratification of the water column that also affects nutrient flux to the benthos, as well as oxygen depletion among many others (Doney et al., 2012; Hoegh‐Guldberg & Bruno, 2010). “It is virtually certain that the Arctic will continue to warm more than global surface temperature, with high confidence above two times the rate of global warming” (IPCC, 2021, p. 19), and all of this is likely to have significant impact on marine populations, species and communities (e.g., Ainsworth et al., 2020). However, still little is known about the complex effects of climate change on marine benthic ecosystems (Hoegh‐Guldberg & Bruno, 2010; Melo‐Merino et al., 2020; Pinsky et al., 2020; Poloczanska et al., 2016).

### 1.2 Effects of climate change on benthic species (response and range‐occupancy)

Possible species reactions to changing marine environmental conditions are shifts in geographic or bathymetric distributions, extinction, adaptation, or tolerance, as derived from past climatic events (e.g., Cordellier & Pfenninger, 2009; Dawson et al., 2011; Dynesius & Jansson, 2014). How individual species will react to environmental change depends on their intrinsic (physiological) and extrinsic (environmental) limitations (Walther et al., 2002). That is, abiotic variables set the upper and lower limits, in which a species can survive and reproduce (i.e., fundamental niche, Hutchinson, 1957), whereas dispersal ability may influence how rapidly it can respond to variation in climatic conditions (Barnes et al., 2009). Thus, marine taxa with limited dispersal abilities, such as those exhibiting a lecithotrophic or brooding reproductive mode, may be especially prone to environmental changes (Sewell & Hoffmann, 2011, but see Lucey et al., 2015). Furthermore, species with larger niche breadth are hypothesized to be able to occur in many different habitats and have a larger distribution (Brown, 1984). In contrast, species with a small geographical distribution would also have a narrow ecological niche. This hypothesis is called the *range–occupancy relationship* or Brown's hypothesis (Brown et al., 1996). For many benthic deep‐sea species, the actual geographical distribution is unknown, but Lörz, Kaiser, et al. (2021) studied the biogeography of the crustacean order of Amphipoda around Iceland for which many species with few observations exist.

### 1.3 Conditions at the Greenland‐Iceland‐Faroe Ridge

Located at the border between the northern North Atlantic and the Arctic seas, waters around Iceland are a key area for water mass exchange and deep‐water formation. As part of the Atlantic Meridional Overturning Circulation (AMOC), the region is also central for heat transfer and maintenance of regional climatic conditions for the neighboring nations (Hansen & Østerhus, 2000; Osterhus & Gammelsrod, 1999). It has been shown that water mass properties, notably temperature and salinity, and depth are primarily responsible for shaping the contemporary distribution of benthic species around Iceland (Brix et al., 2018; Brix & Svavarsson, 2010; Dauvin et al., 2012; Jöst et al., 2019; Lörz, Kaiser, et al., 2021; Weisshappel, 2000). Here, the presence of the Greenland‐Iceland‐Faroe (GIF) ridge, which stretches from Greenland via Iceland and the Faroe Islands to Scotland, represents a strong and mutual bathymetric barrier to water mass exchange and species distribution between the North Atlantic and the Nordic Seas. The predicted climate‐related changes in the physico‐chemical environment therefore have the potential to significantly influence species distributions, especially for those species with a narrow niche breadth, as the conditions in which they can thrive would be further constrained (e.g., Slatyer et al., 2013).

### 1.4 Amphipoda (Crustacea, Arthropoda)

Amphipoda are key players of marine benthic systems. They are widespread and common in Icelandic waters (Brix et al., 2018; Dauvin et al., 2012; Lörz, Kaiser, et al., 2021; Weisshappel & Svavarsson, 1998). Amphipoda differ widely in their functional traits regarding feeding types and mobility levels. Since all female amphipods brood their young in a ventral marsupium (brood pouch) until they are released as juveniles, it is believed that generally the dispersal ability of benthic Amphipoda is limited. Moreover, amphipods have been shown to exhibit, direct or indirect, responses to climate‐related effects, such as to acidification (Egilsdottir et al., 2009; Passarelli et al., 2017; Schram et al., 2016), warming (Mouritsen et al., 2005), salinity changes (Egilsdottir et al., 2009), or shifts in food availability (Havermans et al., 2019; Horton et al., 2020). However, to date, such studies have only used a limited set of variables (one or two), but knowingly the effects of climate change are complex and factors may be interrelated (e.g., Parmesan, 2006). In addition, there is only a very limited understanding of the ecological requirements of individual marine amphipods, which is crucial though for the identification of environmental variables that determine contemporary distributions and how these may change in the face of climate change scenarios for the region. In particular, niche breadth has been rarely quantified for Amphipoda and until today the range–occupancy hypothesis was tested only for a small subset of five Amphipoda (Gaston & Spicer, 2001). These authors were not sure whether Brown’s hypothesis (Brown et al., 1996) can be upheld at all and for Amphipoda in particular. Yet, no further attempts were made to test this hypothesis for marine crustaceans.

### 1.5 Study aims

**FIGURE 1:**
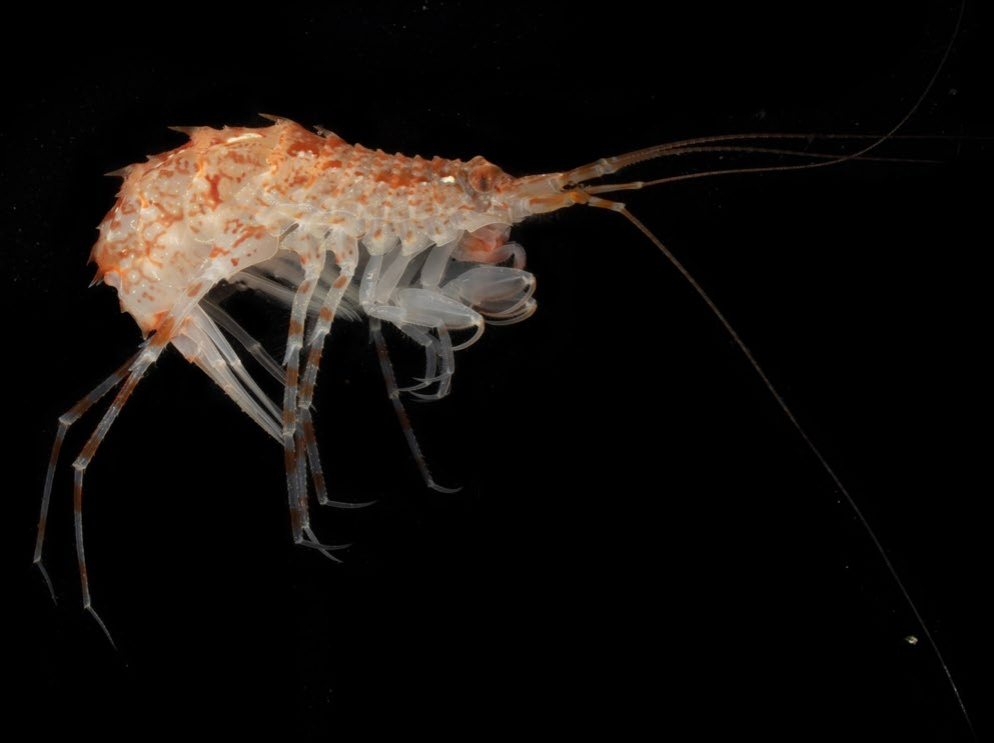
One of the 30 amphipod species from the North Atlantic investigated in detail: *Rhachotropis aculeata* Lepechin, 1780. Photographed by Karlotta Kürzel

In our study, we analyze the ecological niche breadth of Amphipoda across a set of eight major environmental gradients, including near bottom sea water temperature, pH, salinity and several proxies of food availability, and depth, some of which are key to shaping the distribution of amphipods (Davin et al., 2012; Lörz, Kaiser, et al., 2021; Weisshappel & Svavarsson, 1998) and are also expected to change due to regional warming (Astthorsson et al., 2007; IPCC, 2021; Sweetman et al., 2017). Therefore, we modelled species response curves (SRC) based on Huisman et al. (1993, also termed HOF) hierarchical regression approach to quantify the ecological niche of selected Icelandic amphipod species. HOF models explicitly calculate basic niche parameters (optima, lower + upper limits) for a species. Although commonly used in plant‐ and paleoecology, this approach has been scarcely applied in zoo‐ecology (Michaelis & Diekmann, 2017), and to our knowledge never in a marine context. In addition, we examined range–occupation relationships, and whether this is related to the range of environmental conditions in which a species occurs. Specifically, we sought to investigate the following questions:

How do individual species (e.g., Figure 1) respond to major marine environmental gradients and in relation to their niche attributes?
Do species responses correlate with their family assignment in Amphipoda?
Is the size of a species’ geographic range governed by its niche breadth (cf. Brown et al., 1996)?

## 2 MATERIALS AND METHODS

### 2.1 Species and environmental data

In a previous study (Lörz, Kaiser, et al., 2021), the biogeography of deep‐sea amphipod communities was analyzed for the area around Iceland and a large dataset (Lörz, Brix, et al., 2021) was compiled from recent and historical expeditions sampling benthic invertebrates. The dataset consists of 355 amphipod species (equaling about 5% of all marine amphipod species known worldwide) with 71,108 individuals from 532 localities. For most species, only a small number of localities (<20) were available, insufficient to study niche characters. We have filtered a set of suitable species by the number of localities (>30) which resulted in a subset of 30 species from nine families (Table 1). For these species, we added additional observations from the GBIF database (www.gbif.org) in order to improve the coverage of the environmental gradients. As the abundance data of the GBIF data were not provided, we transformed all abundance information to presence‐absence data.

**TABLE 1:** List of analyzed species and their number of occurrences in Lörz, Kaiser, et al. (2021), extracted from GBIF and in terms of analyzed hexagonal cells

Nr.	Family	Species	Author	Lörz, Kaiser, et al. (2021)	GBIF	Occ_hex
1	Amphilochidae	*Amphilochus anoculus*	Tandberg & Vader, 2018	44	19	63
2		*Amphilochus hamatus*	(Stephensen, 1925)	31	25	50
3		*Amphilochus manudens*	Spence Bate, 1862	106	2485	2544
4		*Amphilochus tenuimanus*	Boeck, 1871	50	336	384
5		*Gitanopsis bispinosa*	(Boeck, 1871)	53	408	455
6	Calliopiidae	*Cleippides quadricuspis*	Heller, 1875	48	204	248
7		*Halirages fulvocinctus*	(M. Sars, 1858)	81	467	486
8		*Haliragoides inermis*	(G.O. Sars, 1883)	42	431	463
9		*Laothoes meinerti*	Boeck, 1871	35	140	174
10	Caprellidae	*Aeginella spinosa*	Boeck, 1861	83	86	162
11		*Caprella ciliata*	G.O. Sars, 1883	31	129	156
12		*Caprella microtuberculata*	G. O. Sars, 1879	28	26	48
13	Cressidae	*Cressa carinata*	Stephensen, 1931	41	10	51
14		*Cressina monocuspis*	Stephensen, 1931	37	4	41
15	Eusiridae	*Eusirus holmii*	Hansen, 1887	39	58	110
16		*Rhachotropis aculeata*	(Lepechin, 1780)	61	1553	1512
17		*Rhachotropis inflata*	(G.O. Sars, 1883)	52	258	269
18	Liljeborgiidae	*Liljeborgia fissicornis*	(Sars, 1858)	26	1551	1577
19	Oedicerotidae	*Arrhis phyllonyx*	(Sars, 1858)	44	1748	1674
20		*Monoculodes packardi*	Boeck, 1871	26	903	826
21	Phoxocephalidae	*Harpinia crenulata*	(Boeck, 1871)	72	6028	5867
22		*Harpinia mucronata*	G. O. Sars, 1879	32	2611	2632
23		*Harpinia propinqua*	Sars, 1891	108	2078	2099
24		*Leptophoxus falcatus*	(G.O. Sars, 1883)	29	1512	1535
25		*Paraphoxus oculatus*	(G. O. Sars, 1879)	43	2350	2380
26	Stegocephalidae	*Andaniella pectinata*	G.O. Sars, 1883	47	61	98
27		*Andaniexis lupus*	Berge & Vader, 1997	54	33	86
28		*Phippsia roemeri*	Schellenberg, 1925	30	84	114
29		*Stegocephaloides auratus*	(G.O. Sars, 1883)	44	15	59
30		*Stegocephalus inflatus*	Krøyer, 1842	56	1749	1696

The same study (Lörz, Kaiser, et al., 2021) also compiled information on environmental variables; however, these were sampled for large‐scale hexagons which were not suitable for our purpose. We thus used the original raster datasets which were downloaded from the Bio‐Oracle 2.1 database (Assis et al., 2018) using the R‐package *sdmpredictors* (Bosch, 2020). We gathered a dataset for eight major environmental parameters from which we expect to act as resource gradients for the selected Amphipoda (Table 2). The environmental parameters were extracted at the sampling localities of the occurrence data and two datasets were prepared for the analysis; a presence–absence matrix (PAM) and a matrix containing the environmental information for each locality.

**TABLE 2:** List of marine environmental parameters used as resource gradients

Acronym	Parameter	Units	Source
depth	Bathymetry	meters	GEBCO URL: http://gebco.net Bathymetry URL: http://www.emodnet‐bathymetry.eu/
pH	pH	unitless	Word Ocean Database URL: https://www.ncei.noaa.gov/
dFe	Dissolved iron concentration	µmol/m^2^	Global Ocean Biogeochemistry NON ASSIMILATIVE Hindcast (PISCES) URL: http://marine.copernicus.eu/
dNO_3_	Dissolved Nitrate concentration	µmol/m^2^	Global Ocean Biogeochemistry NON ASSIMILATIVE Hindcast (PISCES) URL: http://marine.copernicus.eu/
dO_2_	Dissolved oxygen concentration	µmol/m^2^	Global Ocean Biogeochemistry NON ASSIMILATIVE Hindcast (PISCES) URL: http://marine.copernicus.eu/
tmean	Mean sea water temperature	°C	Global Ocean Physics Reanalysis ECMWF ORAP5.0 (1979–2013) URL: http://marine.copernicus.eu/
phyto	Carbon phytoplankton biomass	µmol/m^2^	Global Ocean Biogeochemistry NON ASSIMILATIVE Hindcast (PISCES) URL: http://marine.copernicus.eu/
salinity	Sea water salinity	PSS	Global Ocean Physics Reanalysis ECMWF ORAP5.0 (1979–2013) URL: http://marine.copernicus.eu/
velo	Current velocity	m/s	Global Ocean Physics Reanalysis ECMWF ORAP5.0 (1979–2013) URL: http://marine.copernicus.eu/

Note

Environmental parameters initially extracted from the BIO‐ORACLE 2.0 database (Assis et al., 2018). All parameters are long‐term maxima at minimum depth, except pH and bathymetry.

### 2.2 HOF models

Species response curves for the 30 Amphipoda species were modelled using the hierarchical logistic regression approach suggested by Huisman et al. (1993). It was long assumed that these exist in the form of linear or unimodal distributions (Jongman et al., 1995; Whittaker, 1967). This concept has been extended recently to also allow skewed and bimodal forms (Austin, 1987; Jansen & Oksanen, 2013). In contrast to some of the previous methods for SRCs, the HOF modelling approach (Huisman et al., 1993) combines five hierarchical logistic regression models with increasing degrees of complexity, which can be fitted to the various possibilities of SRCs. We used the R‐package eHOF (Jansen & Oksanen, 2013) to fit five different model types, that is, (I) no‐response, (II) linear, (III) sigmoidal, (IV) unimodal, and (V) skewed unimodal. We did not use the bimodal models proposed by Jansen and Oksanen (2013) as they seemed to create unrealistic results caused by outlying observations along the gradients. Best models were selected using the lowest AIC after bootstrapping the models 200 times. We then extracted the model type (i.e. shape), optima, and central lower and upper border for each species‐parameter combination. Although it was suggested to also use the outer border for describing a species’ niche (Heegaard, 2002) we only focus on the central border as these are more reliable and less influenced by single observations.

### 2.3 Range–occupancy analysis

For each species, we calculated the occupied area in square kilometers using a convex hull sketching the outer limits of the known occurrences. We assume that species also occur within the area covered by the convex hull and the given environmental conditions. We excluded all terrestrial areas such as continents and islands. For calculating the area per species, we transformed the coordinates from geographic coordinates to a northern hemispheric projected coordinate system (EPSG: 6931, Brodzik et al., 2012). Range–occupancy relationships were then analyzed at two different levels. First, we analyzed the occupancy—area relationship with species as observations. A linear regression model was fitted on the log10–log10 scale. Second, we analyzed actual range–occupancy analysis by relating the amplitude or niche breadth, that is, the difference between the higher and lower central boundaries identified by the HOF modelling. Relationships were modelled with robust regression models (rlm, Venables & Ripley, 2002) as these are less affected by outlying observations.

## 3 RESULTS

### 3.1 HOF models

**FIGURE 2:**
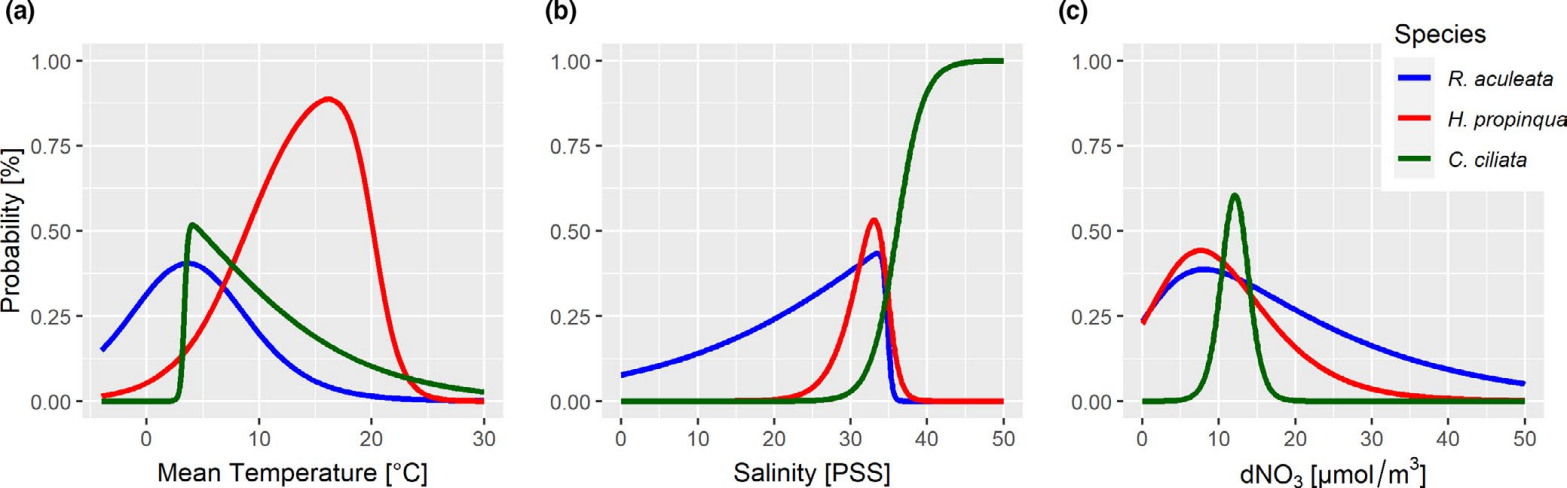
Species Response Curves of three selected species from three families: *Rhachotropis aculeata* (Eusiridae), *Harpinia propinqua* (Phoxocephalidae), and *Caprella ciliata* (Caprellidae) shown for three different resource gradients

HOF models are shown for three selected species that had contrasting distribution patterns in our data set (Lörz, Brix, et al., 2021) as well as different family assignments and lifestyles: *Rhachotropis aculeata* (Eusiridae) (Figure 1), *Harpinia propinqua* (Phoxocephalidae) and *Caprella ciliata* (Caprellidae) (Figure 2). Optima and niche breadth based on nine environmental gradients for all other species are summarized in Appendix S1. All figures (*n* = 360) representing species response curves for each species and each environmental gradient are included in Appendix S2.

*Rhachotropis aculeata* and *Harpinia propinqua* showed different mean temperature optima with a similar amplitude (width of the curve), both species are classic “unimodal” (Figure 2a). The high temperature values for *H*. *propinqua* are based on few records of this species from observations in the Caribbean. *Caprella ciliata* had a clear threshold pattern at 3°C, below which this species did not occur. The species response curves for salinity (Figure 2b) revealed a strong contrast between *R*. *aculeata* and *C*. *ciliata*. 35 PSS is the maximal value for *Rhachotropis aculeata* and by contrast the minimum value for *C*. *ciliata*. *H*. *propinqua*, as most species studied, had a narrow tolerance for salinity. All species showed an optimum at low to medium nitrate levels (Figure 2c). *C*. *ciliata* is restricted to a range between 8 and 18 g/ml.

Nitrate, phosphate, iron, and carbon phytoplankton biomass can be used as proxies for nutrient supply that is available for the amphipod species. Overall, *H*. *propinqua* and *R*. *aculeata* showed similar responses, whereas *C*. *ciliata* had a much narrower amplitude for all four parameters.

**FIGURE 3:**
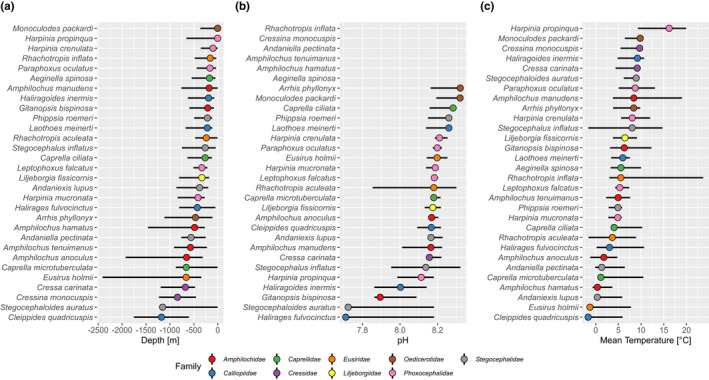
Niche breadth (lines) and optima (points) for 30 Amphipod species across the gradients of (a) depth, (b) pH, (c) temperature. Colors correspond to the nine different families

The niche breadth and optima for 30 Amphipod species across all environmental parameters was investigated. The gradients of depth‐, pH, and temperature are illustrated in Figure 3a–c. The pattern of the amphipod species is different for the tested nine parameters. Slight similarities in niche breadth is observed for dNO_3_, biomass, and dFe. Also, the nine amphipod families did not show a coherent pattern in any of the environmental parameters investigated. The niche breadth and optima for all amphipods for the parameters nitrate, phosphate, iron, oxygen, and carbon phytoplankton biomass are shown in the Appendix S1.

The shallowest depth distribution is shown by species of the families Oedicerotidae, Phoxoceohalidae, and Eusiridae, especially the Eusiridae also show very deep depth gradients. Species of Calliopiidae and Stegocephalidae occur in waters with relatively low pH (7.7. pH), but members of the same family also occur in 8.2 pH. The temperature gradient for warmest waters is shown, as expected, for similar species that occur in shallow depth, *Harpinia propinqua* and *Rhachotropis inflata*. Only a few species of amphipods were sampled in water temperatures below zero degree Celsius.

### 3.2 Range–occupancy analysis

**FIGURE 4:**
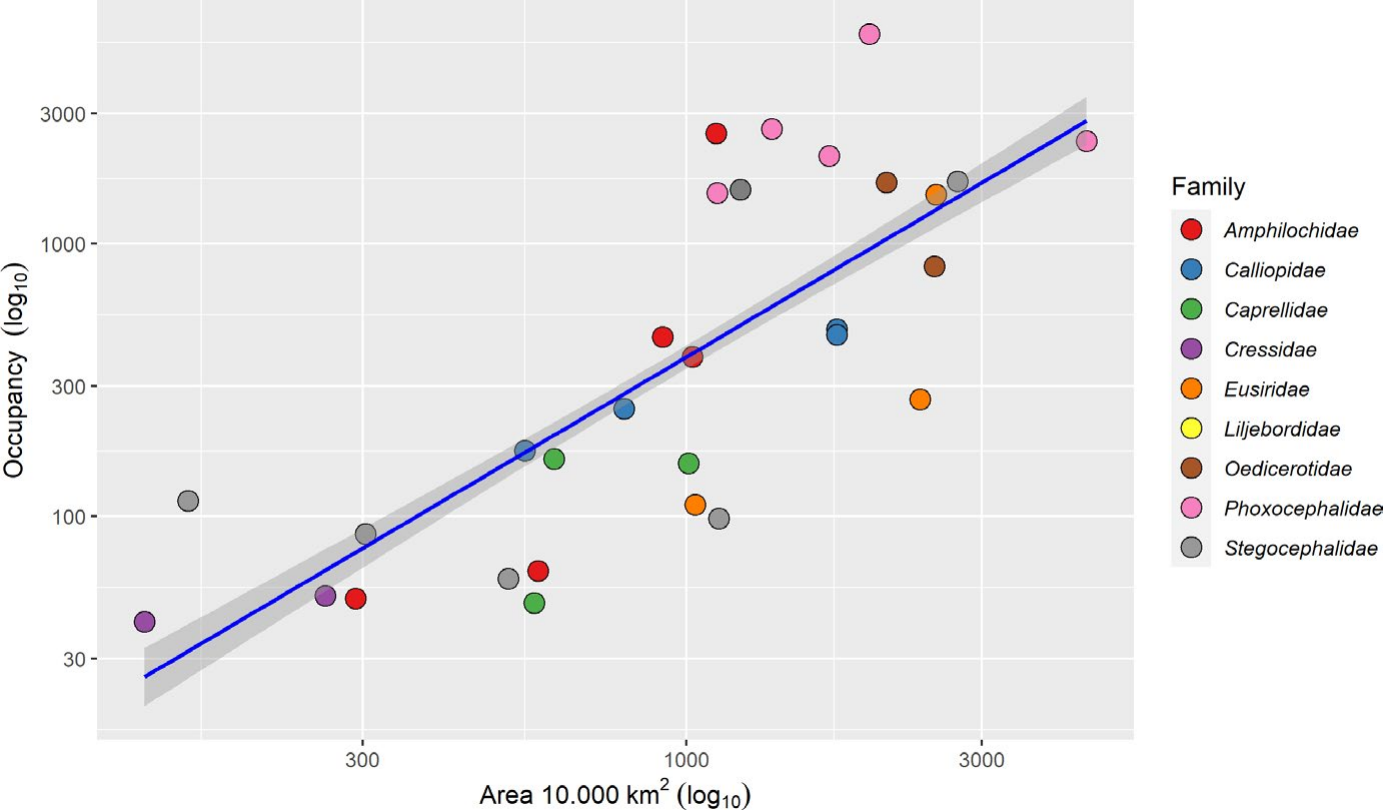
Occupancy–area relationship for Amphipod species (*n* = 30). Both axes are on the log10 scale, colors indicate family status. Area values were divided by 10,000 to better display *x*‐axis labels. A linear model was fitted (*R*² 0.95) to measure the relationship

We found a strong positive occupancy–area relationship for amphipod species (*n* = 30) using a linear regression model. The observed relationship (*R*²: 0.59) had a highly significant slope (*β* = 1.34, SE = 0.058), *t* = 22.883, *p* < .001) on the log‐log scale (Figure 4). Rare species (low occupancy) showed a low distribution while widespread species showed high occupancy values. Some families, such as Stegocephalidae and Amphilochidae, included species with low, medium and high distributions. Species with mainly low occupancy and area belonged to the Cressidae while species belonging to Phoxocephalidae and Oedicerotidae showed high area and occupancy values.

**FIGURE 5:**
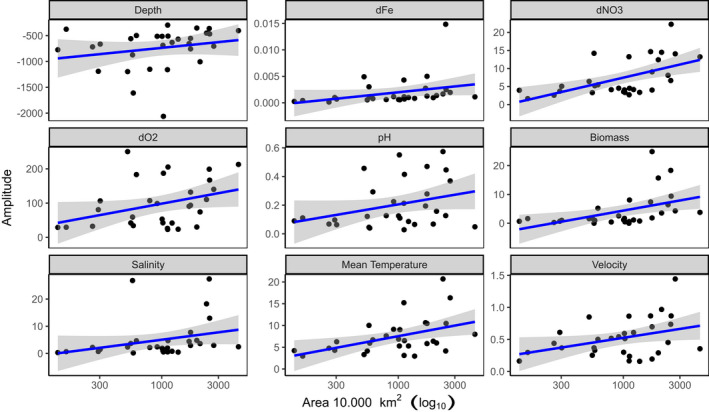
Niche breadth (Amplitude) range size relationships for 30 Amphipod species. Range size (area) was scaled to 10,000 km² to better display x‐axis labels. Fitted models are ordinary least squares (OLS) models

The Niche breadth (Amplitude) range size relationships for 30 Amphipod species is shown in Figure 5. Range size (area) was scaled to 10,000 km² to better display x‐axis labels. Fitted models are robust regression models. The corresponding statistics are summarized in Table 3. All models revealed positive slopes indicating that the general hypothesis of amplitude–range size relationship holds true across different parameters. From nine parameters, four show a significant relationship at the 5% level (nitrate, salinity, carbon phytoplankton biomass, and velocity) and further three at the 10% significance level. Only pH and depth were not significant probably due to large scatter. The nitrate‐model had the best fit (*R*²: 0.31) followed by phytobiomass and temperature.

**TABLE 3:** Linear Regression models of Amplitude–Area relationships. Grey marked models are significant at the 5% level

Parameter	*R*²	Sigma	*t*‐value	*p*‐value
nitrate	0.316	4.291	12.936	.001
phyto	0.218	5.334	7.819	.009
tmean	0.212	3.759	7.538	.010
velo	0.137	0.280	4.434	.044
oxy	0.125	64.675	4.005	.055
ph	0.101	0.160	3.158	.086
iron	0.100	0.003	3.114	.089
salinity	0.090	6.939	2.762	.108
depth	0.048	394.967	1.423	.243

## 4 DISCUSSION

We examined environmental thresholds for selected species of benthic amphipods distributed around Iceland using SRCs. Depending on the shape of the SRC to environmental gradient, species optima and the upper and lower bounds where species occur, that is, their niche breadth were assessed.

### 4.1 How do individual species respond to major marine environmental gradients?

Our first objective was to determine species‐specific responses along gradients of eight environmental parameters and depth in order to estimate niche attributes in amphipods. Most species responded to all parameters examined (Appendix S1), but the shape of the obtained SRCs differed (Figure 1). In our three example species, the differences in mean temperature and salinity were particularly pronounced, while responses to nitrate were not that different; here, all species showed unimodal curves with their optima at low to moderate NO_3_ values; however, in *C*. *ciliata*, the range in which the species can occur for this factor was much narrower than in the other two species.

Nitrate and, likewise, phosphate and iron, are limiting nutrients that control primary productivity in surface waters of the oceans (Arrigo, 2005; Ellwood et al., 2018). Thus, the fact that all of the species examined here have their optimum at low to moderate (<15 µMol/m^³^) nitrate concentrations indicates that they prefer regions that are less nutrient‐rich.

Climatic scenarios for the Arctic and sub‐Arctic regions contain forecasts for significant changes in productivity (e.g., Smith et al., 2000). For example, a strong stratification of the water column due to melting sea‐ice cover and freshwater input in addition to an increased coastal runoff of organic matter can favor enhanced phytoplankton spring blooms (Mann & Lazier, 1991). This in turn can cause a cascade of ecological effects on nutrient cycles that can reach from the surface through the water column to great ocean depths, with the potential to significantly change composition and diversity of the communities there (as shown for abyssal scavenging amphipods, Horton et al., 2020). As productivity changes, we thus expect alterations in the distribution of the amphipod fauna and the replacement with nutrient‐tolerant species.

Waters around Iceland are characterized by very different water masses, including strong temperature gradients across the GIF ridge and with increasing depth. The different environmental conditions are reflected in the composition of the biota, with some species being adapted to cold polar conditions (north of the GIF) and others thriving in warmer (North Atlantic) temperatures (e.g., Brix & Svavarsson, 2010; Lörz, Kaiser, et al., 2021; Schnurr et al., 2014; Weisshappel & Svavarsson, 1998). This contrasting pattern became also evident in the current study; temperature optima of *R*. *aculeata* and *C*. *ciliata* were markedly lower (at ~3°C) compared to *H*. *propinqua* (ca. 15°C). In *C*. *ciliata*, the temperature optimum coincides with a lower threshold below which this species cannot endure. *Caprella ciliata* shows a strong threshold at 3°C, implying its difficulties of coping with cooling waters. While no general cooling of North Atlantic is excepted, changes in the currents are forecasted (Loterhus et al., 2021; Puerta et al., 2020) which might bring cold currents to the current habitat of these caprellids. Predicted warming will be more problematic for cold‐adapted species, such as *R*. *aculeata* or *Amphilochus hamatus* (Figure 2c). However, temperature would have to rise by more than 5°C, which is toward the upper bound of what is predicted for the Arctic region by current climate models (Seneviratne et al., 2018). The distribution of *H*. *propinqua* via open access biogeographical data from OBIS and GBIF has been documented as far as the Caribbean. While large spatial distributions are confirmed for some amphipod species (e.g., *R*. *aculaeta*, or scavenging *Eurythenes gryllus* and *Paralicella tenuipes*: Havermans et al., 2019; Jażdżewska et al., 2021; Lörz et al., 2018), these would have to be evaluated more precisely for *H*. *propinqua* (and most other species in the data set), so that with more corresponding data temperature optima for this species could shift. To avoid rising temperatures, species could migrate poleward or into deeper waters, but this only applies to deep‐sea species that are already used to high hydrostatic pressures (Brown & Thatje, 2015; Lörz, Kaiser, et al., 2021); for more shallow‐water (<1000 m) species, such as *Andaniexis lupus*, *Harpinia mucronata*, or *Phippsia roemeri* in our study (Figure 3a), this may not be an option. In addition, a possible potential northward shift in the Arctic is limited by the expansion of the coastline (with the central part of the Arctic being a large deep‐sea basin). Therefore, warming water temperatures may pose a greater risk to these species (Figure 3c). On the other hand, regional warming together with increased maritime traffic due to opening seaways also offer “opportunities” for species invasions to the Arctic and sub‐Arctic (Beermann et al., 2020; Chan et al., 2019; Goldsmit et al., 2020; Węsławski et al., 2018). Thereby, the loss of endemic species due to changing conditions enables non‐native species to fill new emerging ecological niches (Goldsmit et al., 2020). Native species either cannot tolerate new environmental conditions and therefore have to move to other areas, or they lose their competitive advantage over non‐native species, which may even be equally or better adapted to the new conditions and replace native species (Byers, 2002).

The clearest responses were observed for salinity in our analysis (Figure 2b), which is in a way not surprising, since amphipod species knowingly are strongly influenced by water masses with specific salinity regimes (Lörz, Kaiser, et al., 2021; Weisshappel, 2000). *R*. *aculeata* (Figure 1) showed the greatest tolerance to different salt contents, although this species cannot tolerate PSS > 35. In contrast, *C*. *ciliata* occurs only in waters above PSS 35 and therefore needs a significantly higher salinity than *R*. *aculeata*. *H*. *propinqua* has a very narrow salinity range with an optimum at medium PSS levels (approx. 32.5). This narrow niche for salinity can also be seen in most other amphipod species in our study (Figure 4). A decrease in salinity, for example, as a result of glacial melt due to warming and the subsequent discharge of freshwater, can therefore lead to serious consequences for several species, especially in coastal regions of the Arctic and sub‐Arctic (e.g., Węsławski et al., 2011). For the species we have examined, this would apply above all to *C*. *ciliata*, a species that can be found off the coast of Greenland, but basically all species in our study, except for *R*. *aculeata*, *Halirages fulvocinctus*, and *Monoculodes packardi* (Appendix S1), would not be able to endure low saline waters. This strong influence of salinity on amphipod performance is also illustrated by the results of Egilsdottir et al. (2009) who found out that lowering the salt content, more than lowering pH, has a negative impact on embryonic development in a (temperate) intertidal amphipod species. Similarly, Brown et al. (2020) found lower salinity to reduce energy budgets (and thus in the longer term growth and reproduction) in an Arctic amphipod species.

There are several studies that investigated effects of decreasing pH on marine amphipods (Benítez et al., 2016; Brown et al., 2020; Egilsdottir et al., 2009; Goulding et al., 2017; Hauton et al., 2009; Passarelli et al., 2017; Schram et al., 2016), but some species appear to be more resilient than others (Passarelli et al., 2017). Crustaceans may not be as much affected by ocean acidification as mollusks or echinoderms, because their exoskeletal CaCO_3_ is mostly in the more stable form of calcite rather than the more soluble aragonite form (Whiteley, 2011 and citations therein). Yet, direct effects of ocean acidification on amphipods have also been demonstrated, for example, by impairing metabolic processes and thus fitness of species (Borges et al., 2018; Hauton et al., 2009). In addition, indirect effects include, for example, habitat changes where species depend on calcifying organisms to cling on (such as caprellids; Lim & Harley, 2018). In Icelandic surface waters, a decrease in pH from 8.13 to 8.08 was observed between 1985 and 2008 (Olafsson et al., 2009). For some shallow water species such as *Caprella ciliata*, *Monoculodes packardi* or *Phippsia roemeri*, this already represents a critical threshold (Figure 3b). Although the process is slower in deep waters, acidification is also observed in the North Atlantic deep‐sea. The deep convection activity in the North Atlantic Subpolar Gyre injects surface waters loaded with anthropogenic CO_2_ into lower layers, causing the remarkable acidification rate observed in the Iceland Basin (−0.0016 ± 0.0002 per year) (Olafsson et al., 2009; Vázquez‐Rodríguez et al., 2012). Overall, however, sensitivities to ocean acidification appear to differ between species and are due to differences in lifestyle and ability to adapt to environmental change (Lucey et al., 2015; Whiteley, 2011).

### 4.2 Do species responses correlate with their family assignment in Amphipoda?

In our study, the three selected species, *H*. *propinqua* (Phoxocephalidae), *C*. *ciliata* (Caprellidae), and *R*. *aculeata* (Eusiridae) showed different responses to environmental gradients and depth (Figure 2). However, these could not be directly transferred to other members of the respective family, that is, not all species within the same family showed the same pattern (Figure 3). It should be noted here that some families in our data set were only represented by 1–2 species (Table 1). Other families, such as the Amphilochidae or Phoxocephalidae, were better represented, so species within these families would be more likely to reflect the range of responses in those families.

Within amphipods, certain families can be assigned to certain functional traits, with regard to their mobility and feeding behavior. For example, phoxocephalids are fossorial and burrow within soft sediments (De Broyer et al., 2003). They are generally considered as predators (Guerra‐García et al., 2014; Oliver et al., 1982; Oliver & Slattery, 1985). Caprellid species are often epibionts, associated with other organisms such as algae, hydrozoans, bryozoans (Caine, 1989; Smith & Hirano, 1995), or even commensals of some marine invertebrates including echinoderms (Guerra‐García, 2001; Guerra‐García et al., 2008) and decapods (Martin & Pettit, 1998). Eusirids are abundant members of the deep‐sea fauna off Iceland (Weisshappel, 2000), known to be hyperbenthic predators with good swimming capabilities (Bousfield & Hendrycks, 1995). However, for many deep‐sea Amphipoda species the life trait is unknown. Furthermore, feeding behavior can differ within a family; for example, Amphilochidae contain both carnivorous and omnivorous species (Guerra‐García et al., 2014). Yet even if the family lifestyle is known, great differences in species responses within the families became obvious, such as such as the between the three *Harpinia* species (*H*. *crenulata*, *H*. *mucronata* and *H*. *propinqua*) showing quite different temperature optima at ca. 8, 5, and 16°C (Figure 3c). Similarly, species of Eusiridae (*E*. *holmii*, *R*. *aculeata* and *R*. *inflata*) displayed very different response to different parameters, such as temperature, salinity, and depth (Figure 3a–c). Furthermore, eusirid species can show a wide tolerance along one environmental gradient whilst being very specific to another. *Eusirus holmii*, for example, shows a very narrow temperature distribution—it is only collected in waters colder than 1°C—but has a wide depth range, 400 to 1600 m. *R*. *aculeata* is the opposite: it has a wide temperature tolerance (−1°C to +6°C), and a relative narrow depth distribution, 100–600 m. Since all eusirids are a hyperbenthic group of animals, we would have assumed that due to their strong swimming ability, they disperse more easily (see also Weisshappel, 2000), resulting in wider occurrence of species. However, different Eusiridae species show different biogeographic patterns, which likely also relates to their species‐specific environmental preferences (see also Lester et al., 2007, and discussions therein).

### 4.3 Is the size of a species’ geographic range governed by its niche breadth?

Brown's hypothesis (Brown et al., 1996) predicts that species that are able to inhabit or use a variety of environmental resources (i.e., have a wider niche breadth) are more widespread. In addition, there is evidence that generalist species have greater evolutionary success, as measured by species longevity (Kammer et al., 1997). Conversely, specialized species that have a narrow niche and ultimately a small predicted range could be significantly more sensitive to changing environmental conditions (Slatyer et al., 2013). With this in mind, we assessed range–occupancy relationships of amphipods and related them to their environmental requirements.

We found a strong relationship between geographic range size and occupancy in that species being found at fewer locations had a more restricted spatial spread and could be thus classified as rare (McClain, 2021), compared to “abundant” species containing numerous records in our data set (Figure 4). In addition, widespread species could tolerate a wider variation for certain parameters, notably nitrate, phytobiomass, mean temperature, and velocity (Table 3, Figure 5). By contrast, species with more limited ranges were more specialized (= narrower niche). There were marked differences between the families in that Cressidae, Stegocephalidae, and Caprellidae generally have fewer biogeographic records coupled with a more limited spatial distribution, while Phoxocephallidae and Oedicerotidae include species with wider ranges and higher frequencies (Figure 4). The relationship between geographic range size and niche breadth has been previously assessed in estuarine and fully marine amphipods; Gaston and Spicer (2001) investigated five species of *Gammarus* and found little evidence in support of Brown's hypothesis. Yet, when considering only fully marine species, a correlation between abundance, geographic spread and niche breadth could be established. However, this relationship does not seem to be unequivocal, as some studies are unable to derive any correlation (Gaston & Spicer, 2001) or provide mixed results (Gregory & Gaston, 2000).

Local rarity is not necessarily linked to small geographic ranges, but could also be a sampling artifact (McClain, 2021). This especially holds true for the deep sea, where many regions are hugely undersampled. On the other hand, there are also a number of species, for which we doubt their extraordinarily widespread distribution, including *Andaniella pectinate* and *Paraphoxus oculatus*. This could be due to incorrect taxonomic identification. In particular, individuals with conspicuous features such as spines (e.g. *Eusirus holmii*) may be erroneously assigned to the same morphospecies, while less obvious features are overlooked. In addition, there are few standard works on amphipod taxonomy, and these include monographs by G.O. Sars (1891) based on North Atlantic species. It can be assumed that this work was used to identify species around the world ultimately resulting in the incorrect species assignment. However, as already mentioned for *Rhachotropis aculeata* and others, some widespread occurrences have been confirmed by molecular means, so that it cannot be excluded *per se*, but has to be tested individually.

Nevertheless, rarity is a common feature also in better‐known regions and the form of rarity, with species having low frequencies and being limited in their distribution appears to be most widely occurring trait (McClain, 2021). By contrast, generalist species using a wide range of resources and habitats are relatively rare, which might also be linked to the energetic cost performing this life style (Gaston & Spicer, 2001). For our study, this leads to some ramifications, in that we selected 30 amphipods that had sufficient data points, while the majority of the species retrieved from the data set by Lörz, Kaiser, et al. (2021) did not. If a relationship between range/occupation and niche width exists, as our analysis suggests, it would mean that the remaining ~300 species that we did not study would have a limited geographic range and thus narrow niche breadth. With changing environmental conditions as forecasted for the area (Seneviratne et al., 2018), these species would have to shift their ranges or might go extinct.

## 5 CONCLUSIONS

In this study, we used HOF models for the first time to calculate species response curves and corresponding niche attributes for marine amphipods. We showed responses to be species specific, which could not be transferred to other members of the same family. In addition, a relationship between niche breadths, occupancy, and geographic range could be confirmed suggesting that widespread species are able to tolerate a wider environmental spectrum as opposed to those with a more limited distribution. From this, in turn, it can be deduced that (A) because most deep‐sea species appear to be rare, one might assume that many also have a narrow niche and are therefore at risk, and (B) community or family‐level assessments are insufficient, but climate‐change effects must be addressed on the species level. Most deep‐sea species are undescribed and their geographical distribution and thus also ecological requirements are unknown. This is where taxonomy comes in as the fundamental science for understanding and assessment of biodiversity Our results confirm that precise taxonomic information is necessary in order to record the distribution of species and their changes, on which all ecological model analyses are then based. Models are a first approximation of the niche but more data from more locations are needed to better predict species’ niches. Considering threats to biodiversity, new knowledge of existing species and discovery of undescribed species are urgently thus required.

## CONFLICT OF INTEREST

None declared.

## AUTHOR CONTRIBUTIONS

**Anne‐Nina Lörz:** Conceptualization (equal); Funding acquisition (lead); Project administration (lead); Writing – original draft (equal). **Jens Oldeland:** Data curation (equal); Formal analysis (equal); Methodology (equal); Visualization (equal). **Stefanie Kaiser:** Conceptualization (equal); Methodology (equal); Visualization (equal); Writing – original draft (equal).

## Supporting information

Appendix S1

Appendix S2

## Data Availability

Raw data are available as open access at the Center for Sustainable Research Data Management at the University of Hamburg: https://doi.org/10.25592/uhhfdm.10118
