# Supplementary material for: Niche breadth and biodiversity change derived from marine Amphipoda species off Iceland
Source: Ecol Evol. 2022 Apr 6;12(4):e8802. doi: 10.1002/ece3.8802 (PMC8986549; doi:10.1002/ece3.8802)
Supplement: Supplementary file 1 — Appendix S1 [file ECE3-12-e8802-s002.docx]

| Appendix 1: Optima and niche breadth (from \| to) for the 30 Amphipoda species resulting from the species response curve analysis across the nine environmental gradients. Empty cells identify no optima (Model-Type 1). | | | | | | | | | | |
| --- | --- | --- | --- | --- | --- | --- | --- | --- | --- | --- |
| **Family** | **Species** | **Depth** | **Biomass** | **Iron** | **Nitrate** | **Velocity** | **pH** | **Salinity** | **Oxygen** | **Tmean** |
| Caprellidae | *Aeginella spinosa* | -172.68 | 0.73 | 0.84 | 12.07 | 0.31 | 176.12 | 0.7 | 5.54 |  |
|  |  |  | (-545.76 \| -48.29) | (0.25 \| 5.45) | (0.44 \| 1.23) | (8.37 \| 13.85) | (0.18 \| 0.68) | (172.42 \| 355.75) | (0.42 \| 0.83) | (3.28 \| 9.99) |
| Amphilochidae | *Amphilochus anoculus* | -651.73 | 0.02 | 0.62 | 14.57 | 0.01 | 313.98 | 0.89 | 1.78 | 8.17 |
|  |  | 34.87 | (-1924.74 \| -314.13) | (0.02 \| 1) | (0.62 \| 1.75) | (10.98 \| 16.19) | (0.01 \| 0.34) | (283.88 \| 318.17) | (0.7 \| 1.08) | (-1.19 \| 4.76) |
| Amphilochidae | *Amphilochus hamatus* | -484.17 | 0.02 |  | 13.64 |  |  | 0.89 | 0.37 |  |
|  |  |  | (-1459.63 \| -269.28) | (0.02 \| 0.69) |  | (11.79 \| 15.49) |  |  | (0.76 \| 1.03) | (-0.66 \| 3.64) |
| Amphilochidae | *Amphilochus manudens* | -180.96 | 16.59 | 1.01 | 0.03 | 0.15 | 279.58 | 0.5 | 8.39 | 8.16 |
|  |  | 34.09 | (-760.39 \| 398.47) | (16.59 \| 16.59) | (0.85 \| 5.15) | (0.03 \| 13.28) | (0.07 \| 0.93) | (266.46 \| 292.71) | (0.01 \| 0.83) | (3.78 \| 19.01) |
| Amphilochidae | *Amphilochus tenuimanus* | -569.92 | 0.5 | 0.76 | 13.28 | 0.14 | 289.99 | 0.82 | 4.94 |  |
|  |  | 34.98 | (-913.5 \| -226.35) | (0.12 \| 1.82) | (0.65 \| 1.27) | (10.44 \| 14.51) | (0.09 \| 0.68) | (266.27 \| 308.71) | (0.54 \| 0.94) | (2.29 \| 7.59) |
| Stegocephalidae | *Andaniella pectinata* | -558.79 | 0.02 | 1 | 12.61 | 0.16 |  | 0.7 | 1.33 |  |
|  |  | 35.35 | (-762.52 \| -253.37) | (0.02 \| 8.08) | (0.79 \| 1.51) | (9.5 \| 14.01) | (0.04 \| 0.27) |  | (0.44 \| 0.97) | (-0.13 \| 6.4) |
| Stegocephalidae | *Andaniexis lupus* | -379.16 | 0.9 | 0.85 | 11.71 | 0.39 | 296.72 | 0.18 | 0.34 | 8.16 |
|  |  |  | (-863.2 \| -199.58) | (0.23 \| 1.23) | (0.75 \| 1.45) | (9.17 \| 14.24) | (0.05 \| 0.42) | (264.43 \| 370.91) | (0.18 \| 0.99) | (-0.42 \| 5.84) |
| Oedicerotidae | *Arrhis phyllonyx* | -465.45 | 0.99 | 1.25 | 11.76 | 0.05 | 277.13 | 0.63 | 8.37 | 8.32 |
|  |  | 35.37 | (-1116.02 \| -110.48) | (0.24 \| 3.8) | (0.81 \| 2.18) | (0.31 \| 14.79) | (0.03 \| 0.99) | (247.62 \| 321.91) | (0.03 \| 0.97) | (3.82 \| 9.81) |
| Caprellidae | *Caprella ciliata* | -264.09 | 0.79 | 0.84 | 12.07 | 0.29 | 311.21 | 0.69 | 4.05 | 8.28 |
|  |  | 33.88 | (-634.58 \| -121.45) | (0.37 \| 1.54) | (0.53 \| 1.14) | (10.3 \| 13.85) | (0.14 \| 0.44) | (265.53 \| 318.75) | (0.52 \| 0.83) | (3.36 \| 10.24) |
| Caprellidae | *Caprella microtuberculata* | -658.64 | 10.78 | 0.99 | 0.26 | 0.71 | 276.06 | 0.08 | 1.12 | 8.18 |
|  |  |  | (-877.26 \| -5) | (10.78 \| 10.78) | (0.74 \| 3.76) | (0.26 \| 14.46) | (0.34 \| 0.71) | (260.22 \| 319.59) | (0.08 \| 0.88) | (0.47 \| 10.49) |
| Calliopiidae | *Cleippides quadricuspis* | -1,177.56 | 0.02 | 0.62 | 14.82 | 0.05 | 173.03 | 1.08 | -1.67 | 8.17 |
|  |  | 35.24 | (-1753.07 \| -602.06) | (0.02 \| 0.44) | (0.62 \| 1.23) | (11.9 \| 16.03) | (0.04 \| 0.55) | (161.71 \| 268.86) | (0.73 \| 1.73) | (-1.67 \| 5.93) |
| Cressidae | *Cressa carinata* | -677.33 | 0.15 | 0.78 | 14.28 | 0.36 | 229.17 | 0.88 | 9.15 | 8.16 |
|  |  |  | (-1192.06 \| -475.11) | (0.06 \| 0.32) | (0.71 \| 0.85) | (12.99 \| 15.57) | (0.14 \| 0.58) | (223.87 \| 256.24) | (0.8 \| 0.97) | (4.37 \| 9.15) |
| Cressidae | *Cressina monocuspis* | -841.52 | 0.21 | 0.73 | 14.59 | 0.16 | 237.21 | 0.85 | 9.69 |  |
|  |  | 35.08 | (-1228.28 \| -454.76) | (0.04 \| 0.71) | (0.6 \| 0.85) | (12.61 \| 16.57) | (0.07 \| 0.24) | (233.1 \| 262.16) | (0.74 \| 0.96) | (5.47 \| 9.69) |
| Eusiridae | *Eusirus holmii* | -659.28 | 0.02 | 0.82 | 14.13 |  | 134.55 | 0.91 | -1.27 | 8.2 |
|  |  | 34.87 | (-2406.97 \| -344.41) | (0.02 \| 0.5) | (0.64 \| 1.21) | (11.92 \| 15.26) |  | (134.55 \| 321.79) | (0.71 \| 1.34) | (-1.27 \| 7.78) |
| Amphilochidae | *Gitanopsis bispinosa* | -209.14 | 0.69 | 1.67 | 13.63 | 0.21 | 172.22 | 0.67 | 6.3 | 7.89 |
|  |  | 32.73 | (-593.36 \| -80.69) | (0.2 \| 2.74) | (0.82 \| 1.99) | (10.21 \| 14.73) | (0.1 \| 0.64) | (158.77 \| 257.61) | (0.36 \| 0.98) | (3.17 \| 12.28) |
| Calliopiidae | *Halirages fulvocinctus* | -427.58 |  | 0.51 | 12.19 | 0.11 | 185.73 | 0.83 | 3.03 | 7.71 |
|  |  | 33.72 | (-805.67 \| -49.49) |  | (0.51 \| 5.51) | (6.88 \| 15.95) | (0.03 \| 0.73) | (161.52 \| 293.59) | (0.27 \| 1.21) | (0.19 \| 10.65) |
| Calliopiidae | *Haliragoides inermis* | -191.87 | 0.66 | 1.64 | 13.94 | 0.15 | 173.16 | 1.02 | 9.2 | 8 |
|  |  | 33.04 | (-625.31 \| -72.66) | (0.11 \| 1.95) | (1.01 \| 2.28) | (10.9 \| 14.92) | (0.08 \| 0.27) | (157.66 \| 251.41) | (0.67 \| 1.11) | (4.81 \| 10.66) |
| Phoxocephalidae | *Harpinia crenulata* | -99.22 | 0.74 | 1.74 | 9.62 | 0.2 | 274.84 | 0.64 | 8.04 | 8.21 |
|  |  | 36.76 | (-354.49 \| -2) | (0.46 \| 16.16) | (1.24 \| 2.21) | (0 \| 12.41) | (0.08 \| 0.37) | (257.78 \| 288.03) | (0 \| 0.83) | (5.6 \| 12) |
| Phoxocephalidae | *Harpinia mucronata* | -412.60 | 0.48 | 1.5 | 10.92 | 0.11 | 285.86 | 0.74 | 4.87 | 8.19 |
|  |  | 35.35 | (-839.27 \| -271.31) | (0.21 \| 0.97) | (1.08 \| 1.92) | (9.55 \| 12.92) | (0.07 \| 0.23) | (277.87 \| 301.66) | (0.62 \| 0.86) | (2.77 \| 5.73) |
| Phoxocephalidae | *Harpinia propinqua* | -2.00 | 3.23 | 1.07 | 7.62 | 0.21 | 199.64 | 0.29 | 16.19 | 8.11 |
|  |  | 33.05 | (-656.05 \| -2) | (1.12 \| 8.51) | (0.71 \| 3.46) | (1 \| 15.66) | (0.07 \| 0.93) | (162.59 \| 253.31) | (0.13 \| 0.67) | (9.3 \| 19.95) |
| Calliopiidae | *Laothoes meinerti* | -218.95 | 0.56 | 0.52 | 12.31 | 0.33 | 287.61 | 0.7 | 5.96 | 8.26 |
|  |  |  | (-671.81 \| -113.06) | (0.21 \| 1.89) | (0.52 \| 1.06) | (10.48 \| 13.78) | (0.18 \| 0.43) | (266.48 \| 308.73) | (0.56 \| 0.85) | (3.39 \| 7.53) |
| Phoxocephalidae | *Leptophoxus falcatus* | -330.99 | 0.6 | 1.38 | 10.59 | 0.11 | 284.51 | 0.65 | 5.3 | 8.18 |
|  |  | 35.22 | (-511.67 \| -215.44) | (0.35 \| 0.93) | (1.03 \| 2.16) | (9.45 \| 12.15) | (0.06 \| 0.23) | (269.74 \| 292.92) | (0.49 \| 0.79) | (4.28 \| 7.4) |
| Liljeborgiidae | *Liljeborgia fissicornis* | -331.63 | 0.66 | 1.19 | 11.2 | 0.1 | 285.15 | 0.67 | 6.44 | 8.17 |
|  |  | 35.30 | (-810.33 \| -178.86) | (0.27 \| 1.4) | (0.87 \| 1.86) | (9.13 \| 13.28) | (0.05 \| 0.66) | (256.71 \| 298.57) | (0.47 \| 0.85) | (3.79 \| 9.09) |
| Oedicerotidae | *Monoculodes packardi* | -2.00 | 0.72 | 1.46 | 0.16 | 0.18 | 196.29 | 0.01 | 9.8 | 8.32 |
|  |  | 32.95 | (-363.51 \| -2) | (0.39 \| 18.71) | (1.14 \| 15.98) | (0.16 \| 6.81) | (0.12 \| 0.86) | (153.99 \| 353.1) | (0.01 \| 0.45) | (6.41 \| 10.56) |
| Phoxocephalidae | *Paraphoxus oculatus* | -162.05 | 1.14 | 1.24 | 7.84 | 0.18 | 270.48 | 0 | 8.71 | 8.2 |
|  |  | 36.88 | (-438.36 \| -35.23) | (0.45 \| 4.21) | (0.84 \| 1.96) | (3.16 \| 16.37) | (0.09 \| 0.44) | (93.42 \| 306.36) | (0 \| 1.12) | (5.07 \| 13.05) |
| Stegocephalidae | *Phippsia roemeri* | -215.92 | 0.63 | 0.67 | 12.03 | 0.31 | 284.82 | 0.66 | 4.9 | 8.26 |
|  |  | 34.09 | (-491.44 \| -116.15) | (0.26 \| 1.86) | (0.59 \| 1.01) | (11.42 \| 13.06) | (0.16 \| 0.45) | (275.85 \| 305.38) | (0.63 \| 0.77) | (2.84 \| 5.81) |
| Eusiridae | *Rhachotropis aculeata* | -238.75 | 1.17 | 1.21 | 8.07 | 0.14 | 316.85 | 0.04 | 3.65 | 8.18 |
|  |  | 33.49 | (-471.98 \| -5.53) | (0.18 \| 9.57) | (0.69 \| 3.18) | (0.63 \| 22.88) | (0.06 \| 0.93) | (233.32 \| 400.39) | (0.04 \| 2.07) | (-1.58 \| 8.89) |
| Eusiridae | *Rhachotropis inflata* | -157.40 | 1.23 | 0.55 | 11.48 | 0.46 | 204.86 | 0.49 | 5.52 |  |
|  |  |  | (-481.03 \| -30.79) | (0.47 \| 6.89) | (0.55 \| 2.23) | (6.91 \| 15.01) | (0.23 \| 0.68) | (183.2 \| 293.75) | (0.25 \| 0.74) | (3 \| 23.7) |
| Stegocephalidae | *Stegocephaloides auratus* | -1,153.51 | 1.09 |  | 11.95 |  | 33.65 | 2.75 | 8.92 | 7.72 |
|  |  | 33.13 | (-1199.41 \| -3) | (0.29 \| 1.88) |  | (8.74 \| 15.15) |  | (33.65 \| 284.05) | (1.31 \| 2.75) | (6.18 \| 9.49) |
| Stegocephalidae | *Stegocephalus inflatus* | -262.62 | 0.79 | 1.33 | 7.36 | 0.06 | 340.72 | 0.59 | 8.03 | 8.14 |
|  |  | 33.61 | (-748.31 \| -43.67) | (0.12 \| 4.41) | (0.79 \| 2.74) | (4.31 \| 18.37) | (0.03 \| 1.47) | (229.04 \| 369.33) | (0.17 \| 1.38) | (-1.64 \| 14.72) |
