# Supplementary material for: Niche breadth and biodiversity change derived from marine Amphipoda species off Iceland
Source: Ecol Evol. 2022 Apr 6;12(4):e8802. doi: 10.1002/ece3.8802 (PMC8986549; doi:10.1002/ece3.8802)
Supplement: Supplementary file 2 — Appendix S2 [file ECE3-12-e8802-s001.pdf]

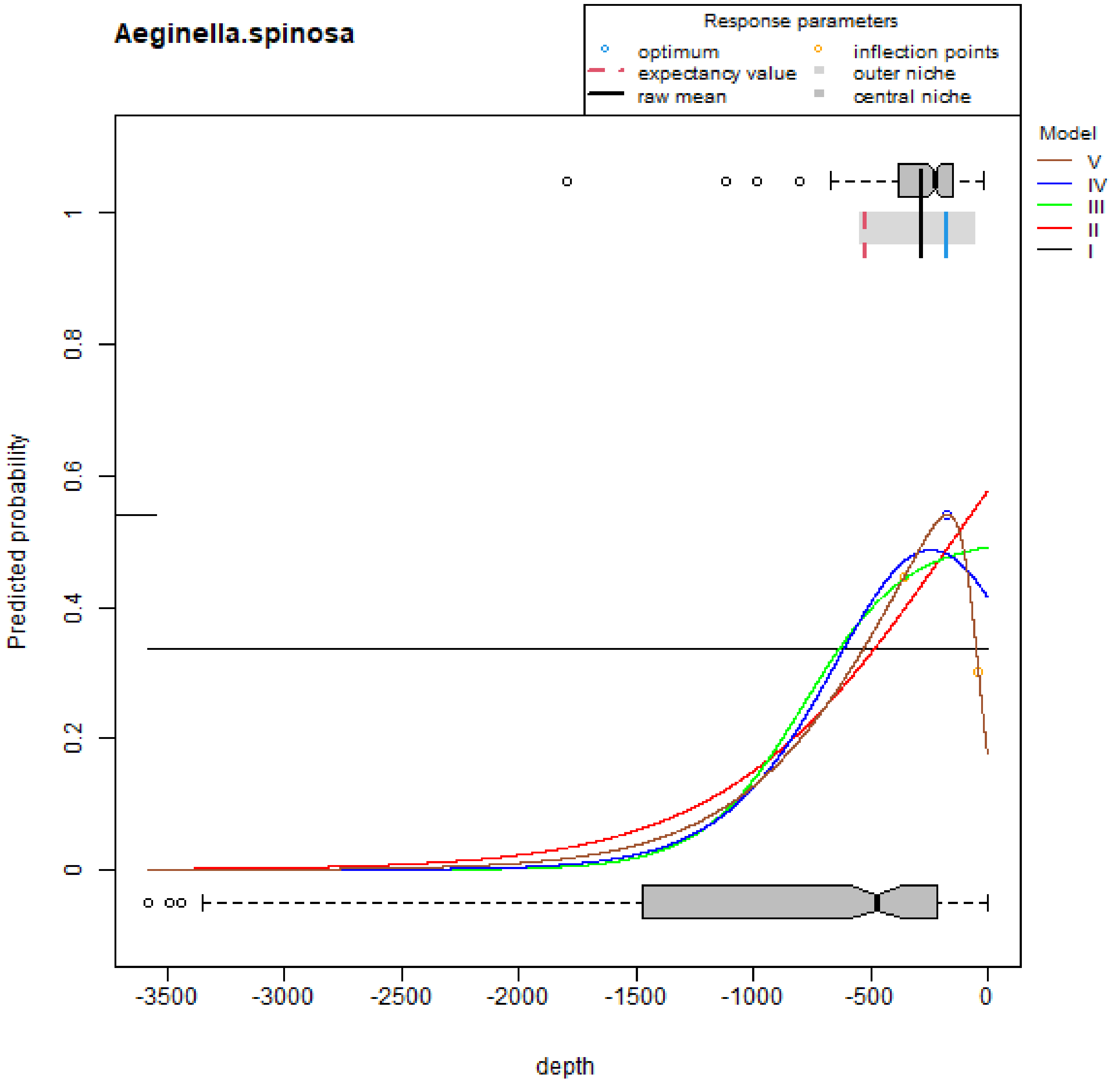

Aeginella.spinosa
Response parameters
optimum
expectancy value
raw mean
inflection points
outer niche
central niche
Model
V
IV
III
II
I
Predicted probability
0
0.2
0.4
0.6
0.8
1
-3500
-3000
-2500
-2000
-1500
-1000
-500
0
depth

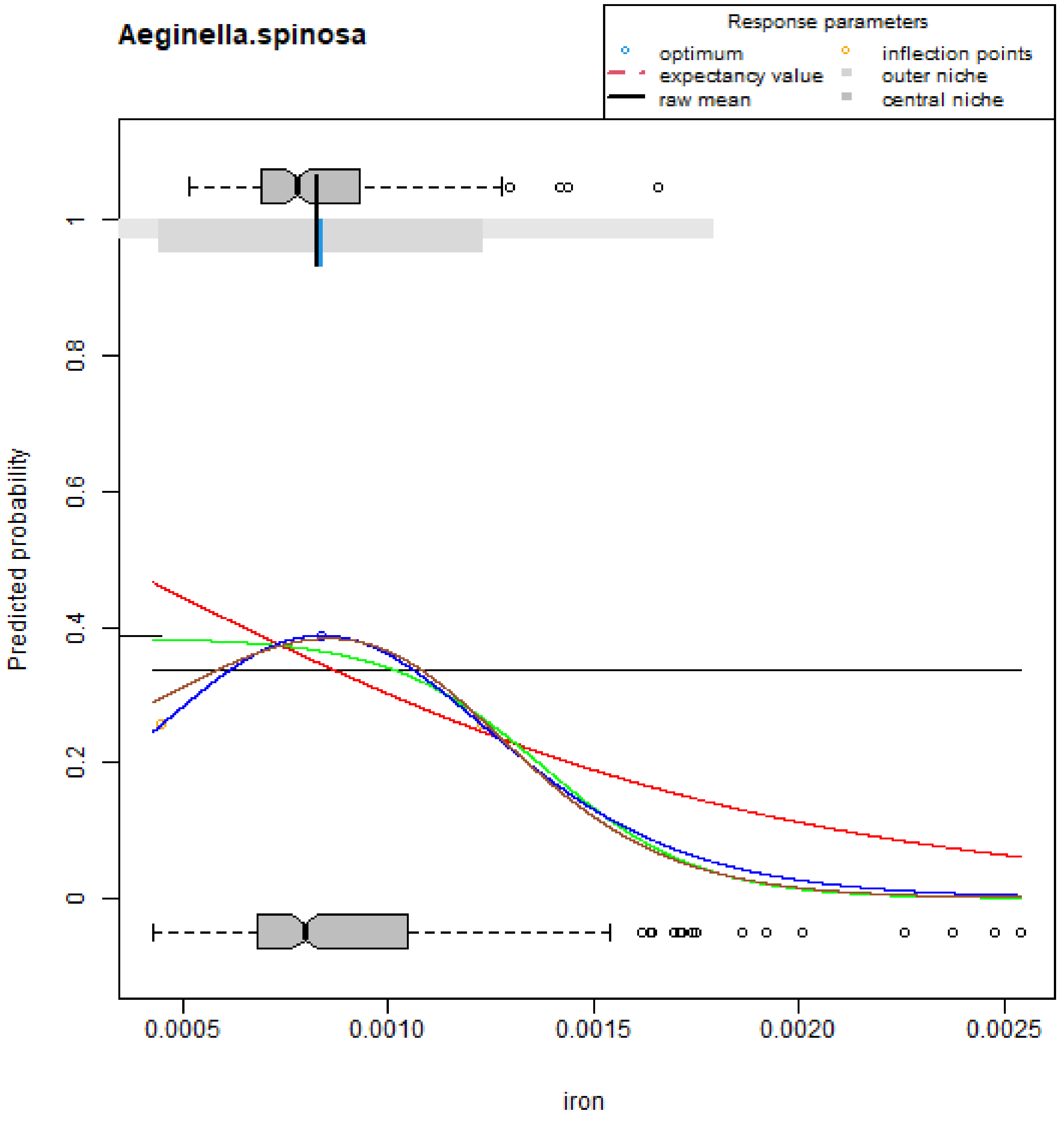
Aeginella.spinosa
Response parameters
optimum
expectancy value
raw mean
inflection points
outer niche
central niche
Predicted probability
0
0.2
0.4
0.6
0.8
1
0.0005
0.0010
0.0015
0.0020
0.0025
iron

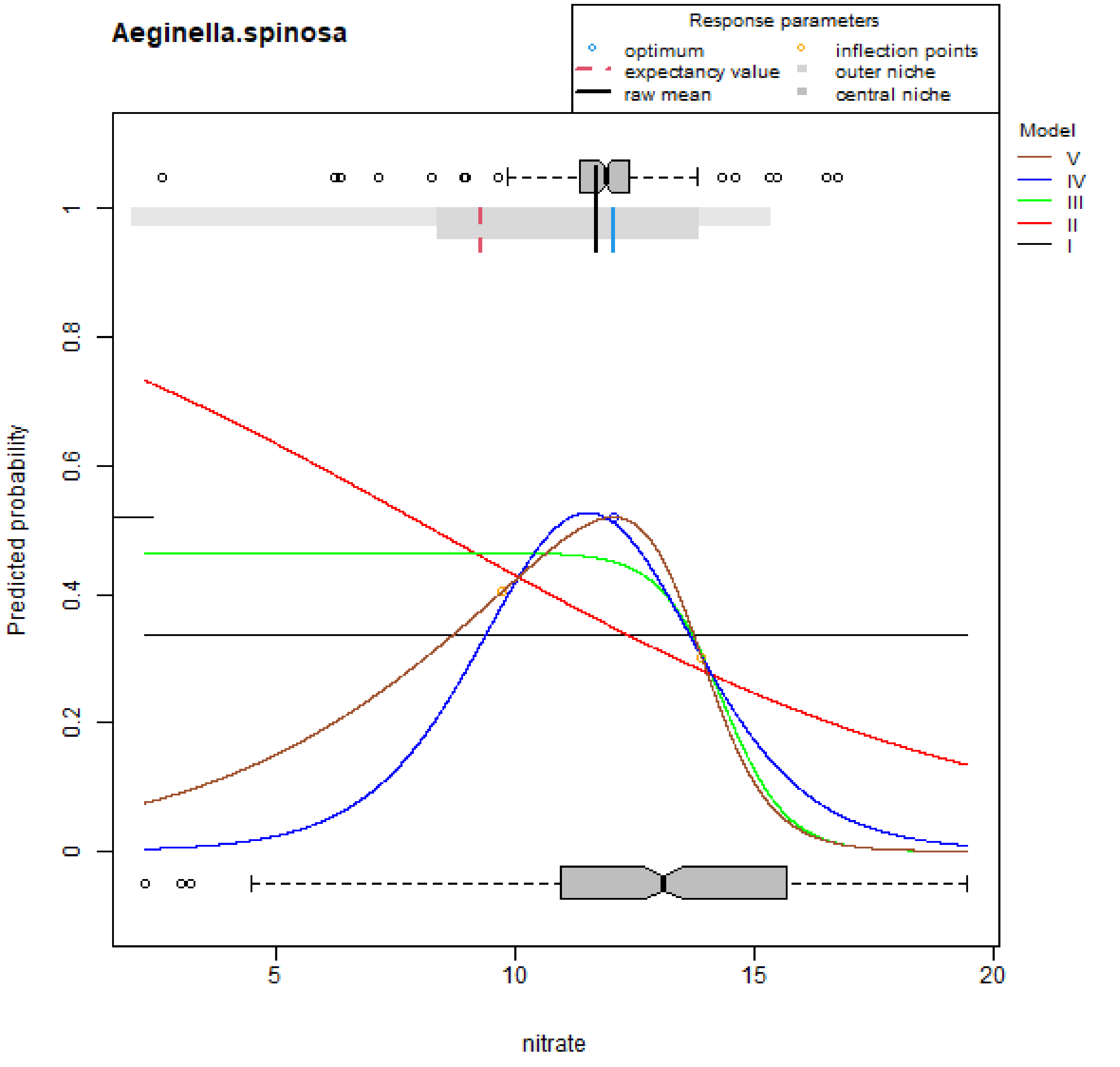

Aeginella.spinosa
Response parameters
optimum
expectancy value
raw mean
inflection points
outer niche
central niche
Model
V
IV
III
II
I
Predicted probability
0
0.2
0.4
0.6
0.8
1
5
10
15
20
nitrate

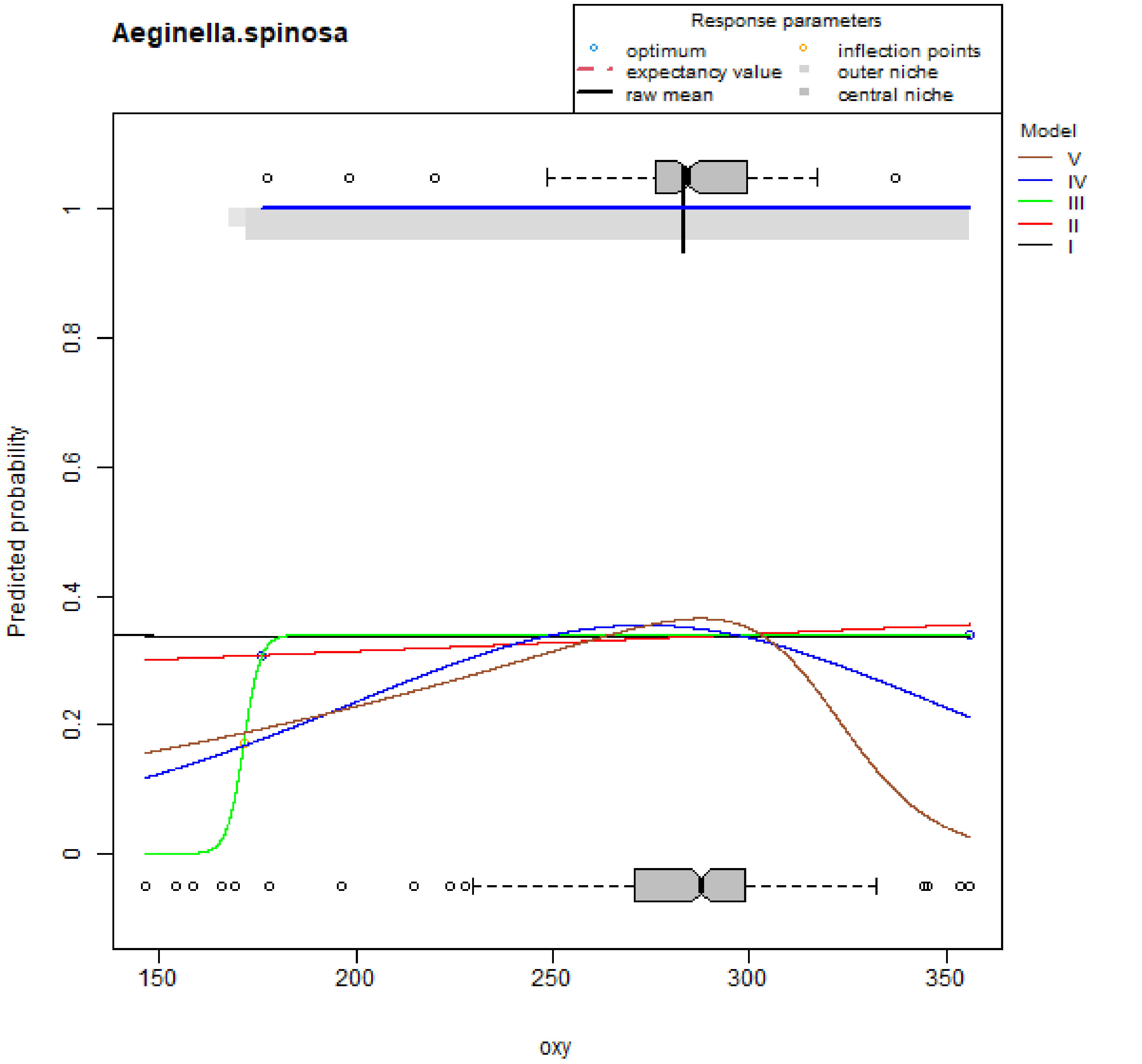

Aeginella.spinosa
Response parameters
optimum
expectancy value
raw mean
inflection points
outer niche
central niche
Model
V
IV
III
II
I
Predicted probability
0
0.2
0.4
0.6
0.8
1
150
200
250
300
350
oxy

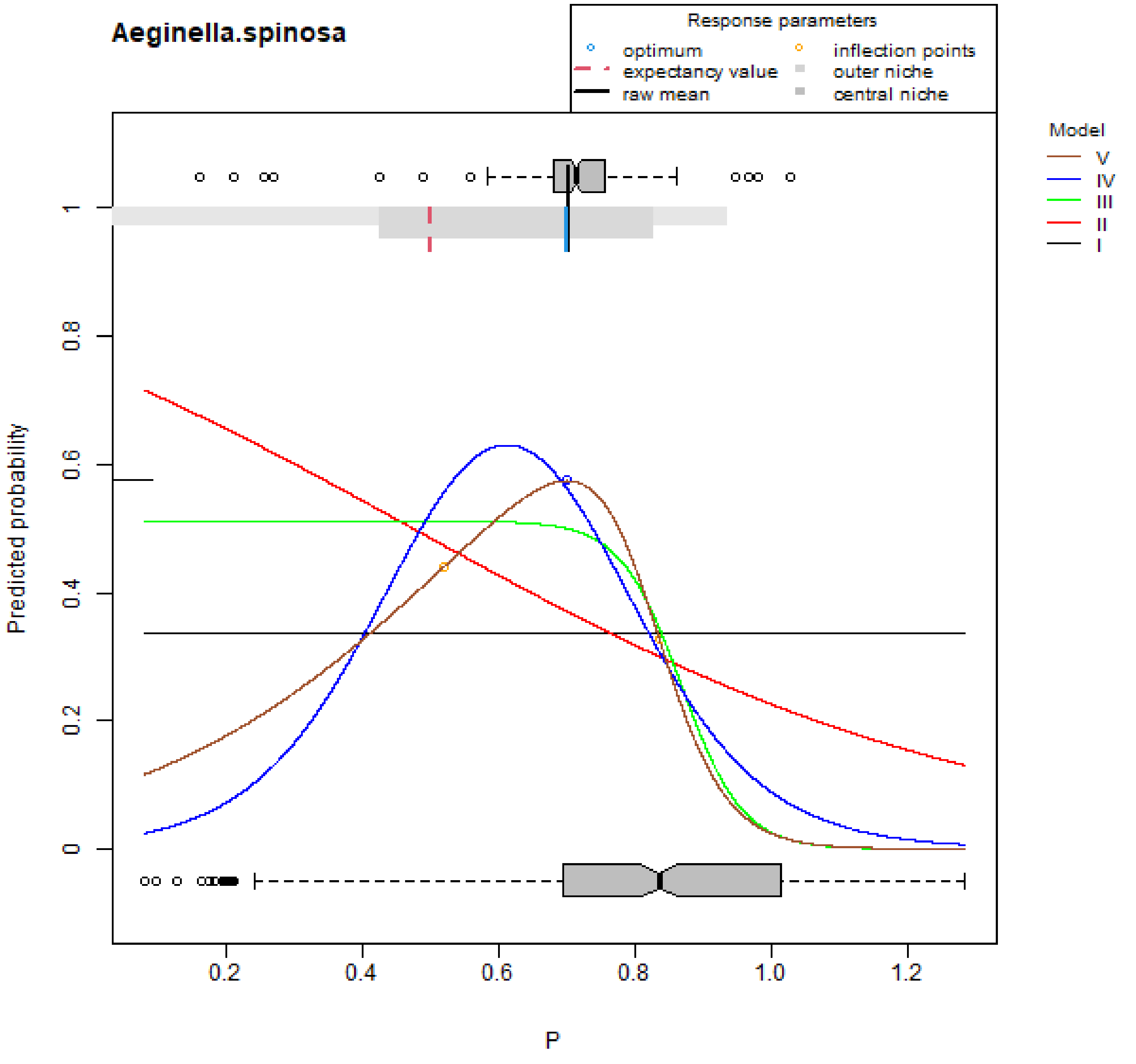

Aeginella.spinosa
Response parameters
optimum
expectancy value
raw mean
inflection points
outer niche
central niche
Model
V
IV
III
II
I
Predicted probability
0
0.2
0.4
0.6
0.8
1
0.2
0.4
0.6
0.8
1.0
1.2
P

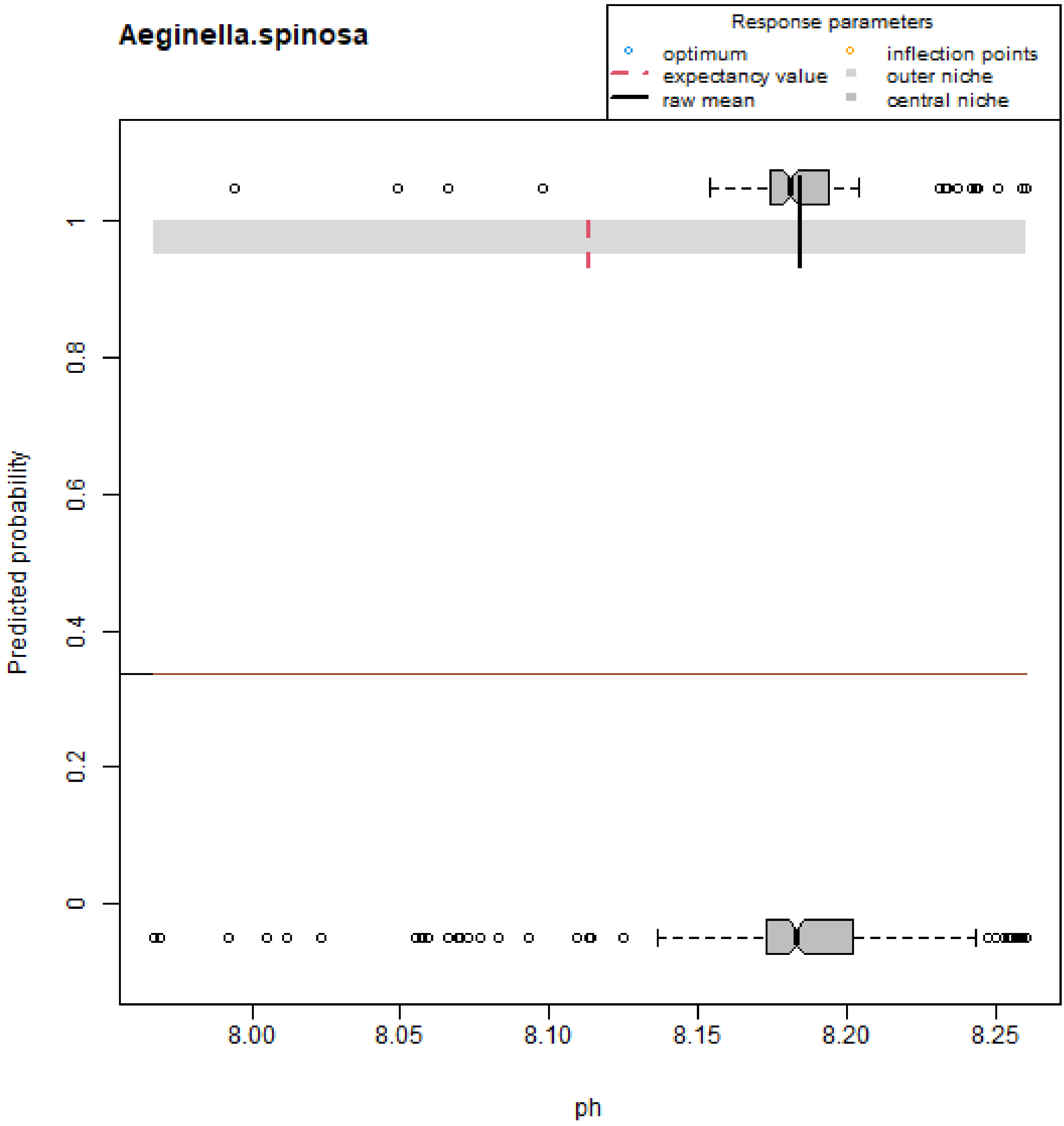

Aeginella.spinosa
Response parameters
optimum
expectancy value
raw mean
inflection points
outer niche
central niche
Predicted probability
0
0.2
0.4
0.6
0.8
1
8.00
8.05
8.10
8.15
8.20
8.25
ph

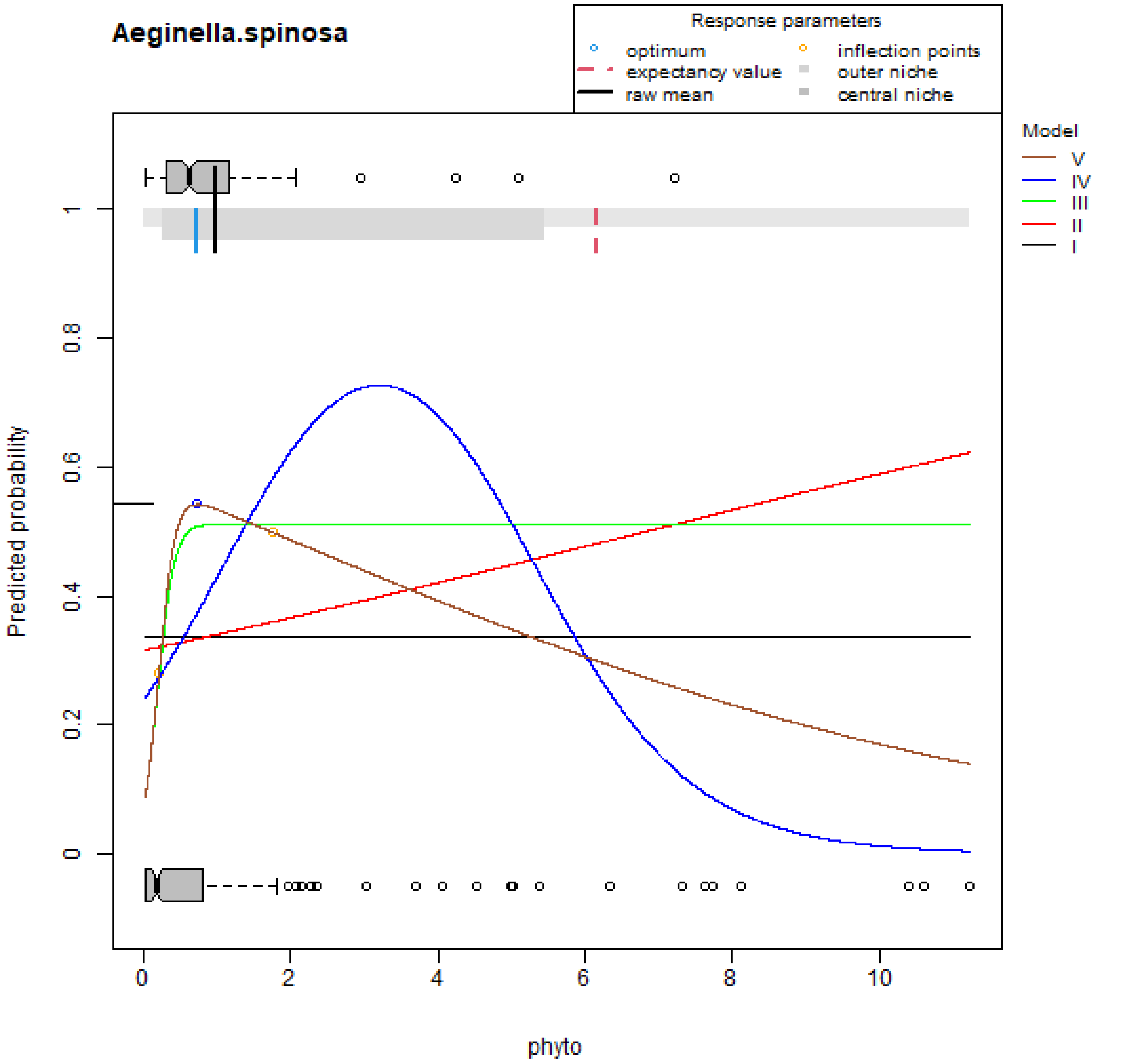
Aeginella.spinosa
Response parameters
optimum
expectancy value
raw mean
inflection points
outer niche
central niche
Model
V
IV
III
II
I
Predicted probability
0
0.2
0.4
0.6
0.8
1
0
2
4
6
8
10
phyto

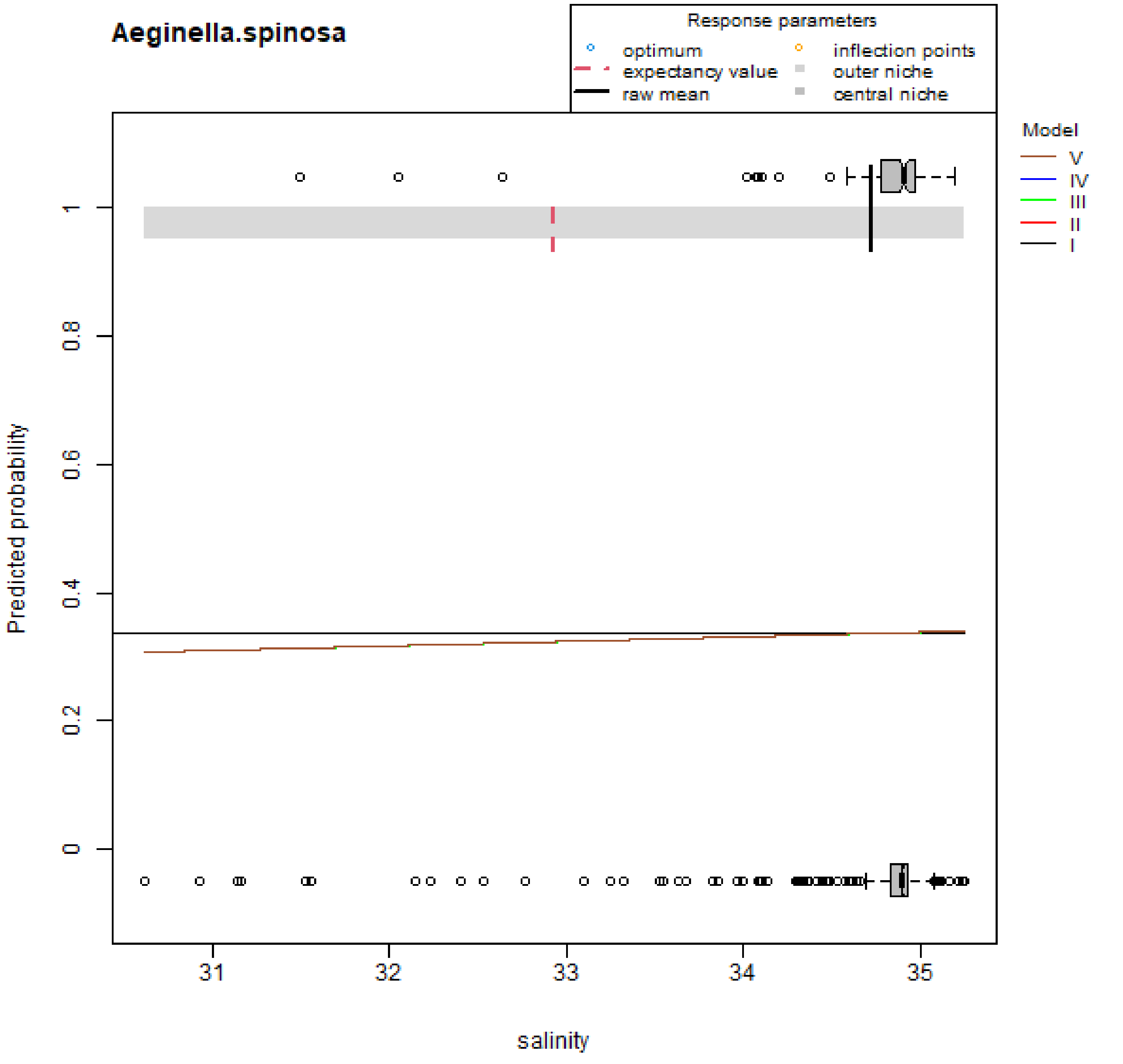

Aeginella.spinosa
Response parameters
optimum
expectancy value
raw mean
inflection points
outer niche
central niche
Model
V
IV
III
II
I
Predicted probability
0
0.2
0.4
0.6
0.8
1
31
32
33
34
35
salinity

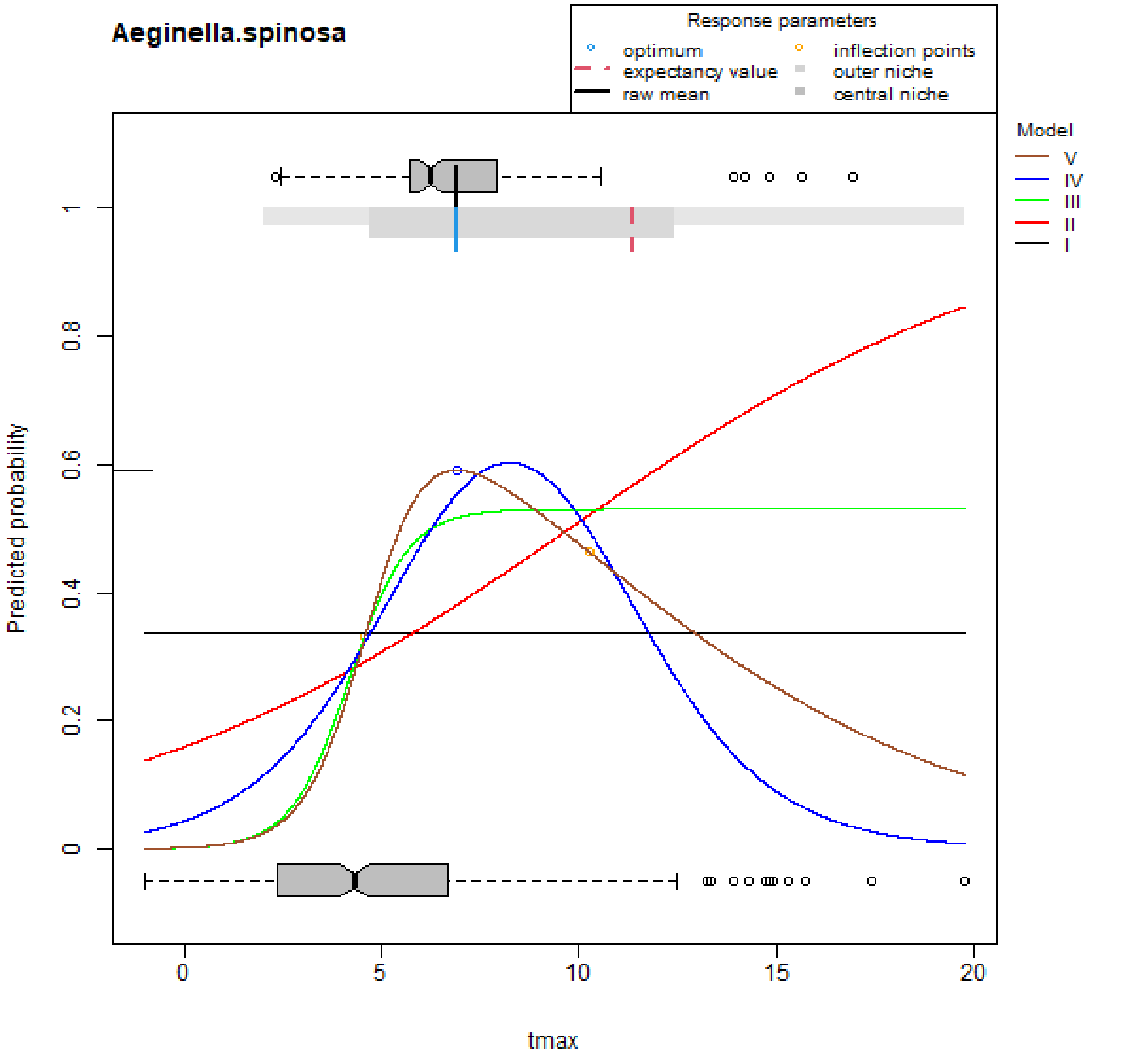

Aeginella.spinosa
Response parameters
optimum
expectancy value
raw mean
inflection points
outer niche
central niche
Model
V
IV
III
II
I
Predicted probability
0
0.2
0.4
0.6
0.8
1
0
5
10
15
20
tmax

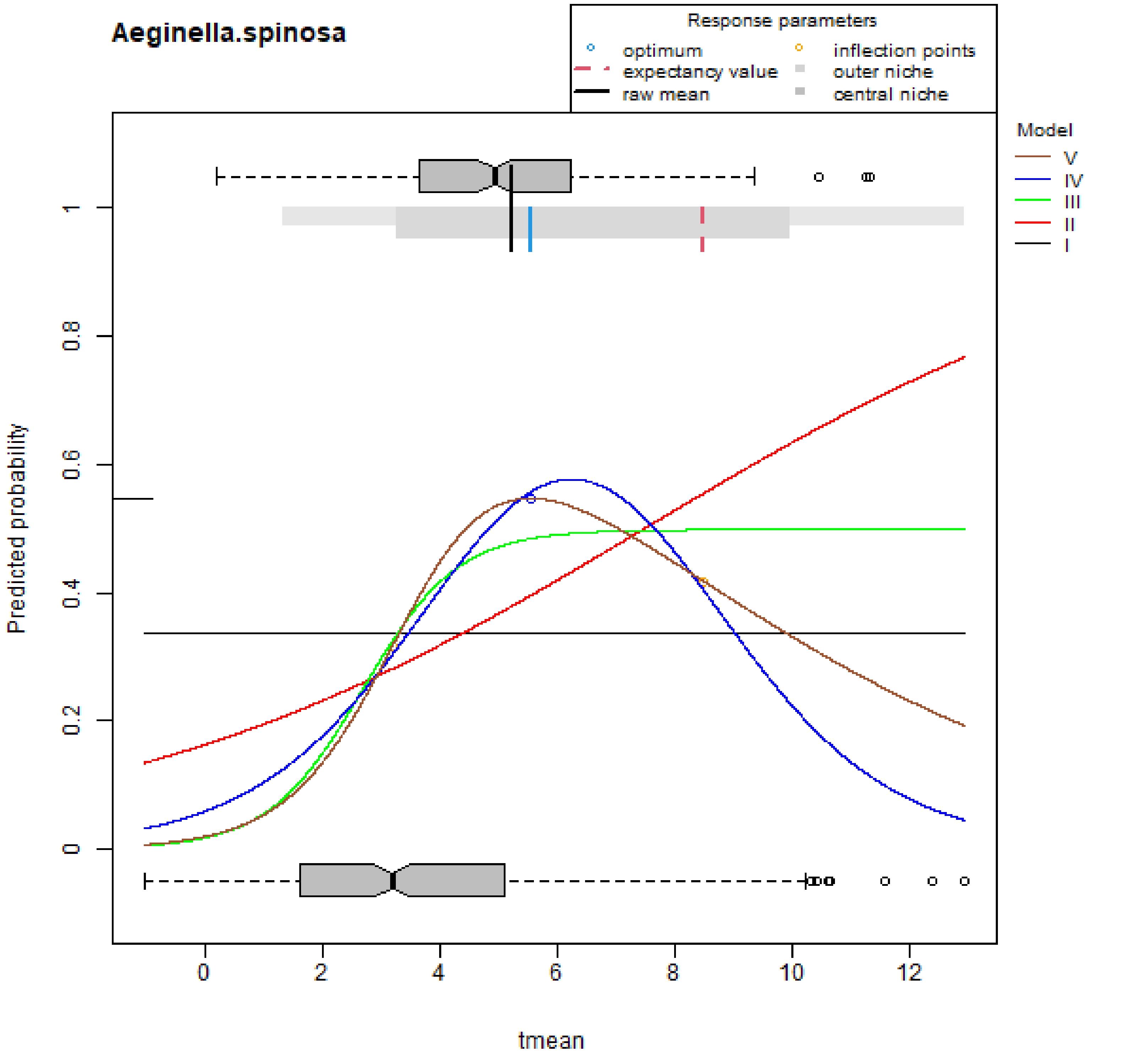
Aeginella.spinosa
Response parameters
optimum
expectancy value
raw mean
inflection points
outer niche
central niche
Model
V
IV
III
II
I
Predicted probability
0
0.2
0.4
0.6
0.8
1
0
2
4
6
8
10
12
tmean

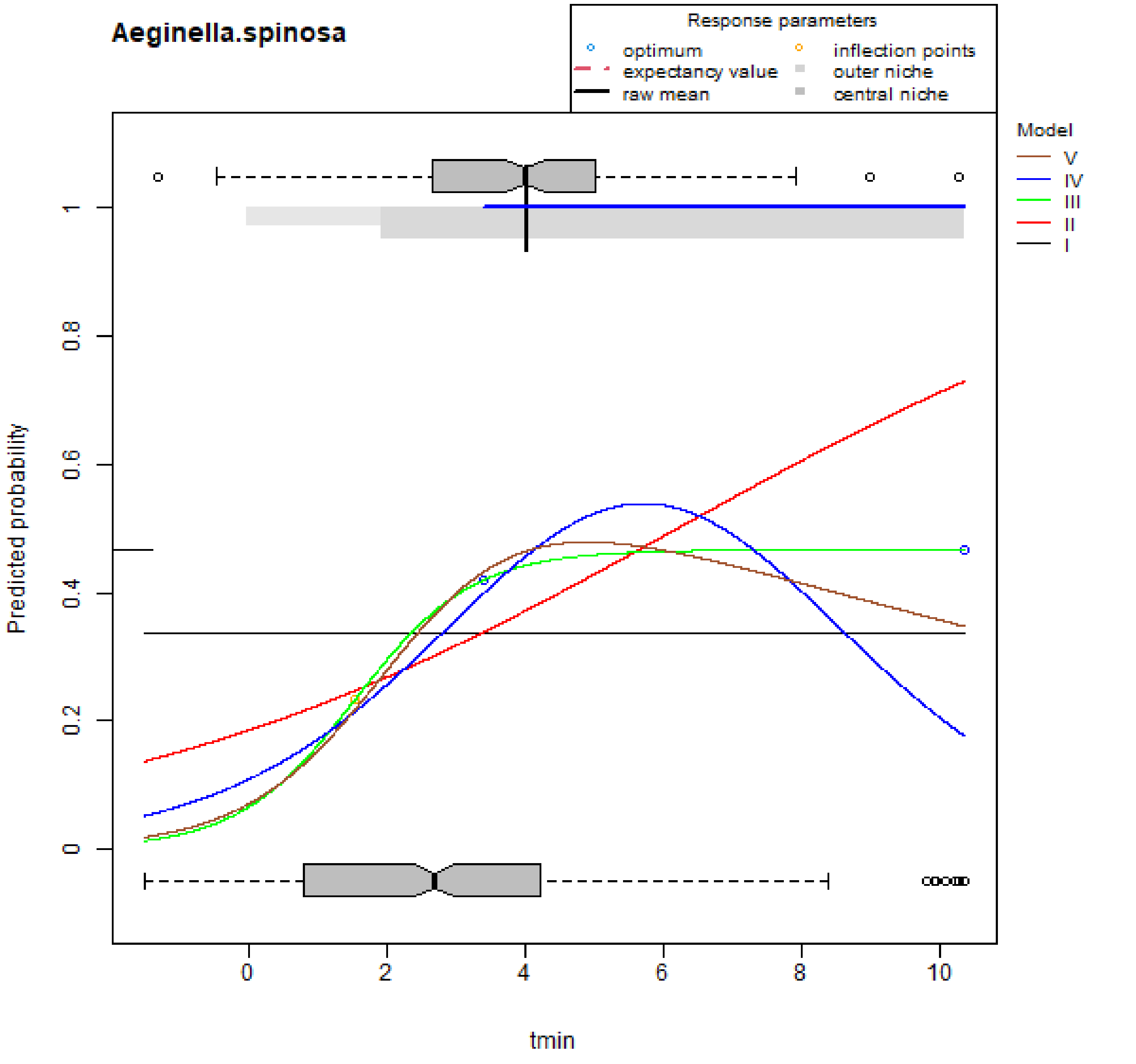

Aeginella.spinosa
Response parameters
optimum
expectancy value
raw mean
inflection points
outer niche
central niche
Model
V
IV
III
II
I
Predicted probability
0
0.2
0.4
0.6
0.8
1
0
2
4
6
8
10
tmin

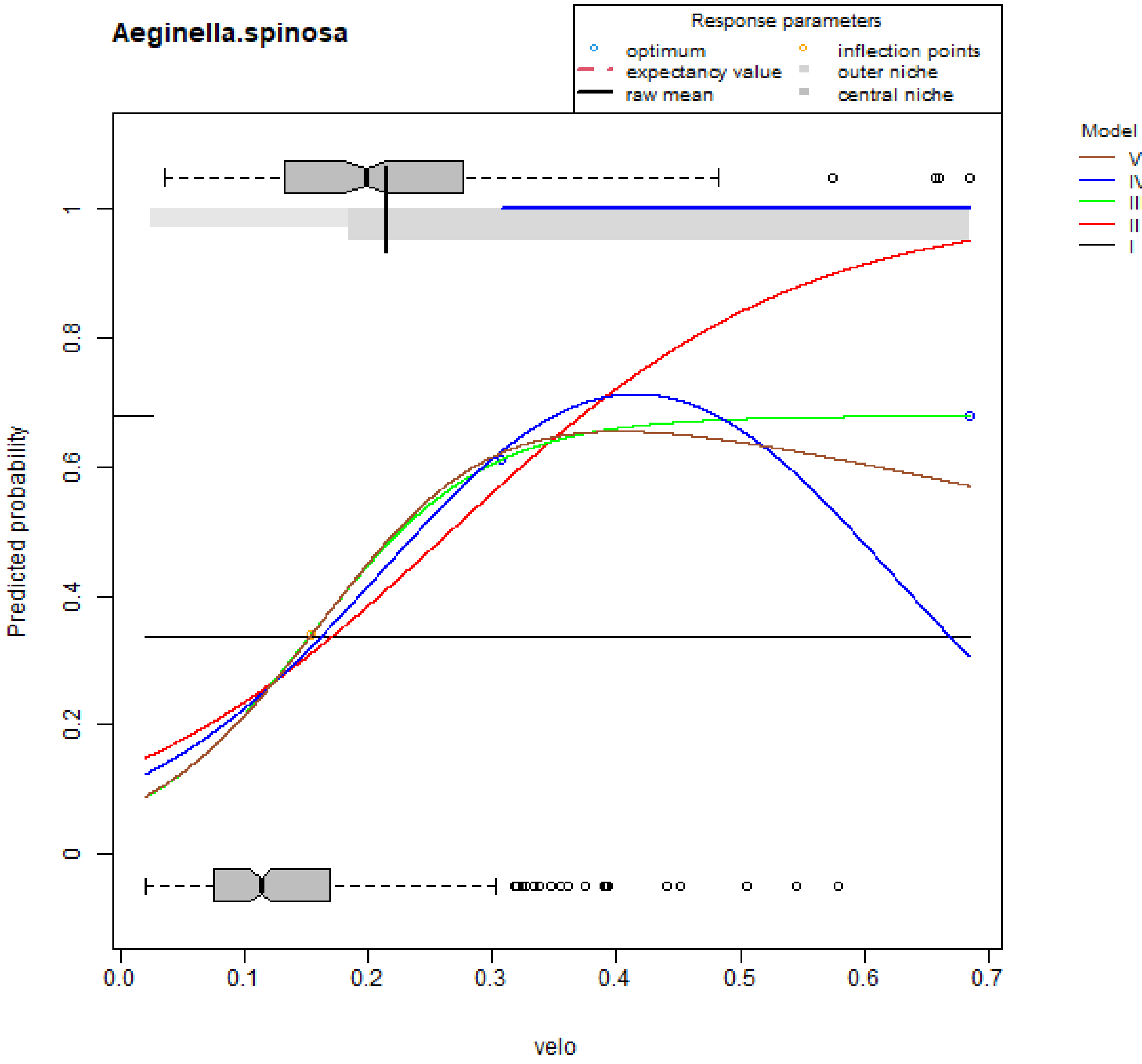

Aeginella.spinosa
Response parameters
optimum
expectancy value
raw mean
inflection points
outer niche
central niche
Model
Predicted probability
0
0.2
0.4
0.6
0.8
1
0.0
0.1
0.2
0.3
0.4
0.5
0.6
0.7
velo

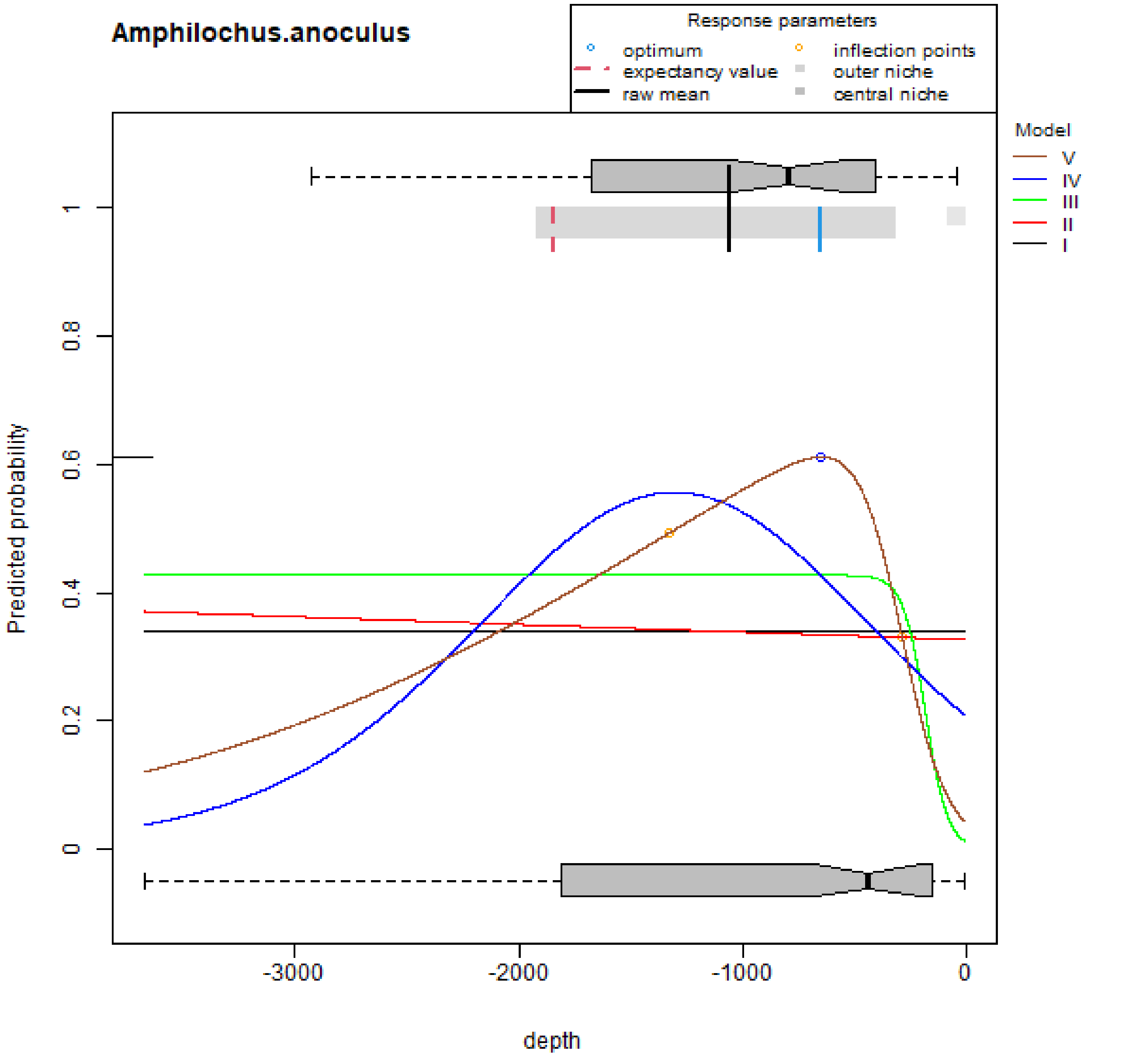

Amphilochus.anoculus
Response parameters
optimum
expectancy value
raw mean
inflection points
outer niche
central niche
Model
V
IV
III
II
I
Predicted probability
0
0.2
0.4
0.6
0.8
1
-3000
-2000
-1000
0
depth

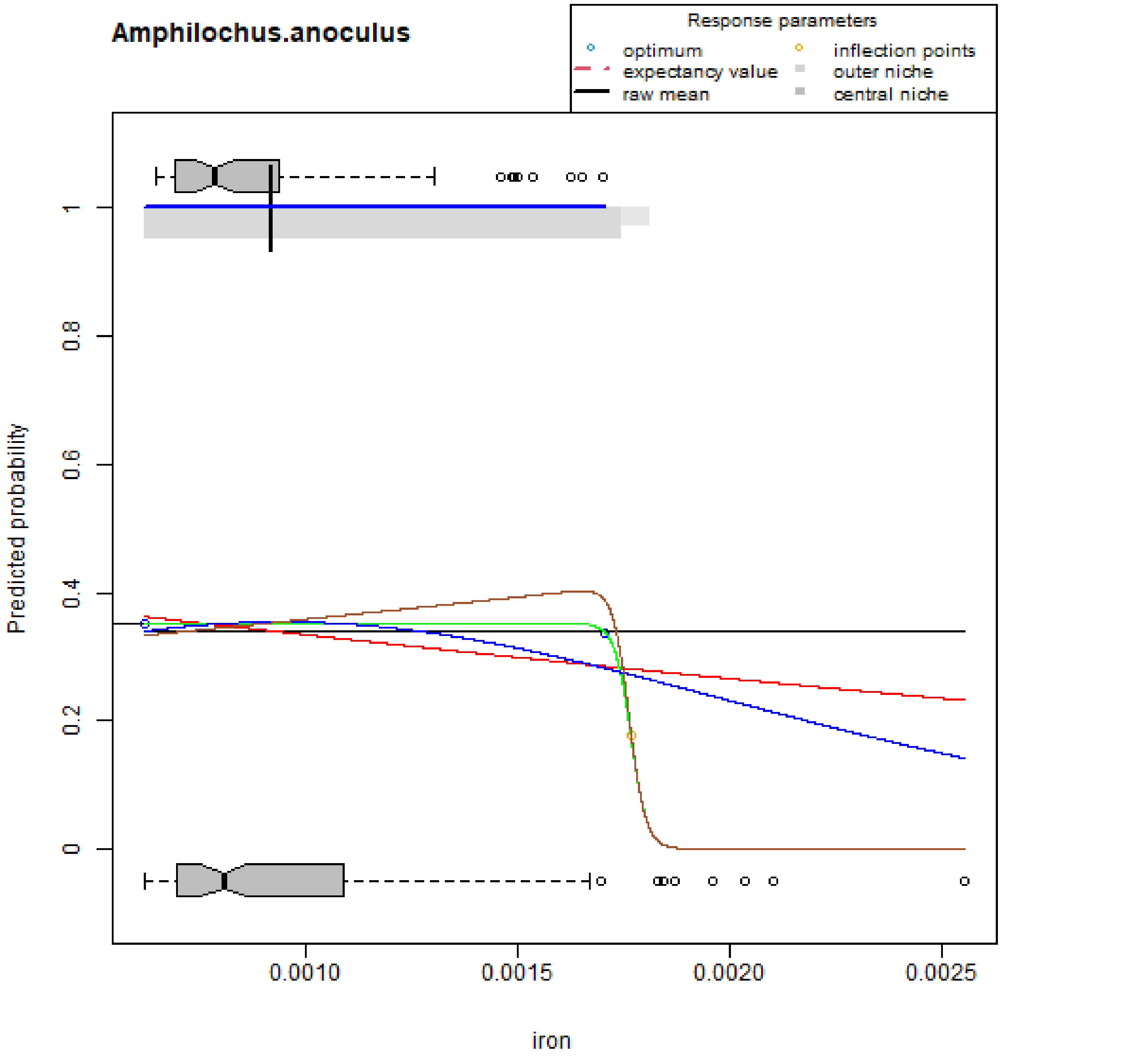

Amphilochus.anoculus
Response parameters
optimum
expectancy value
raw mean
inflection points
outer niche
central niche
Predicted probability
0
0.2
0.4
0.6
0.8
1
0.0010
0.0015
0.0020
0.0025
iron

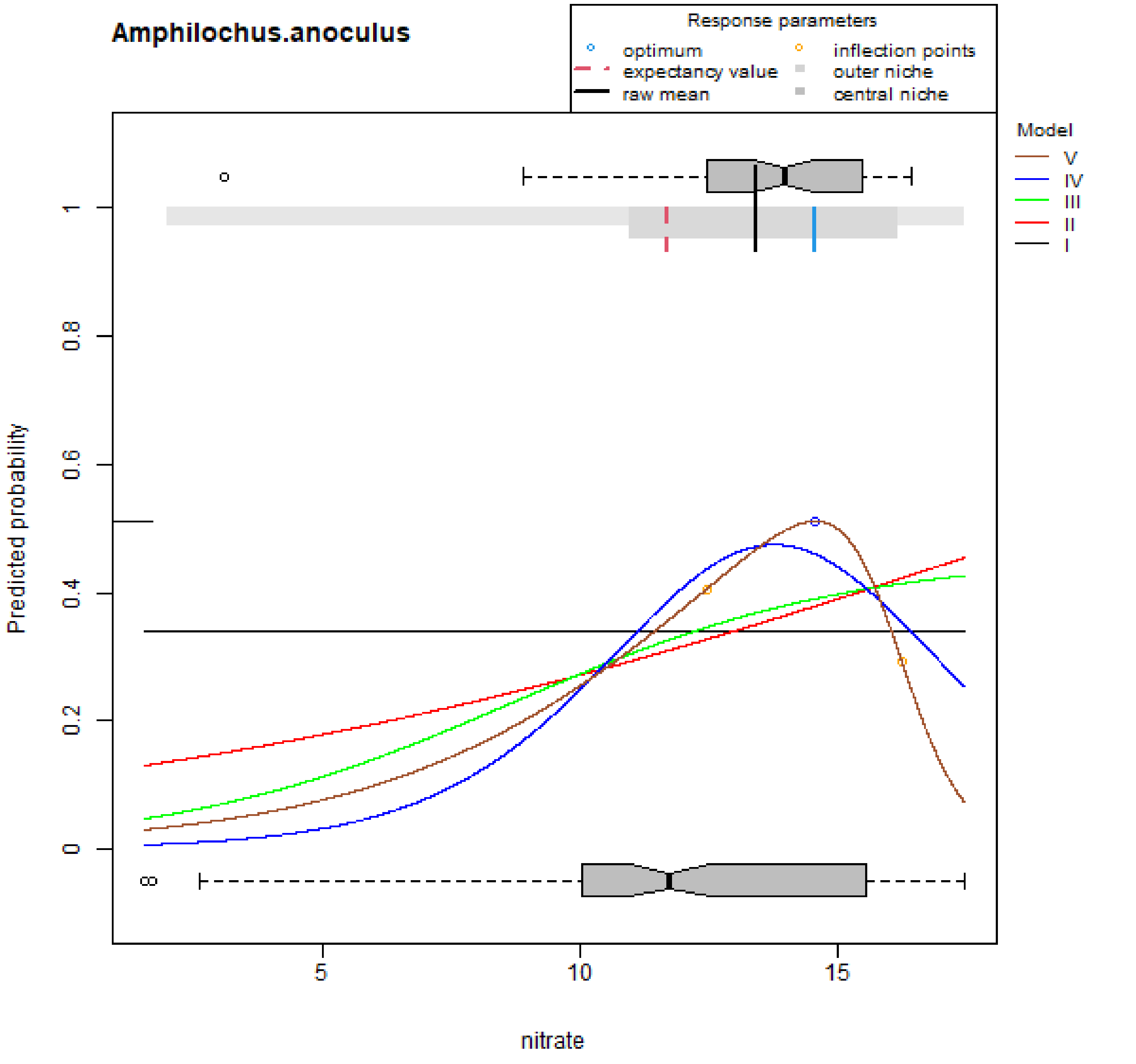

Amphilochus.anoculus
Response parameters
optimum
expectancy value
raw mean
inflection points
outer niche
central niche
Model
V
IV
III
II
I
Predicted probability
0
0.2
0.4
0.6
0.8
1
5
10
15
nitrate

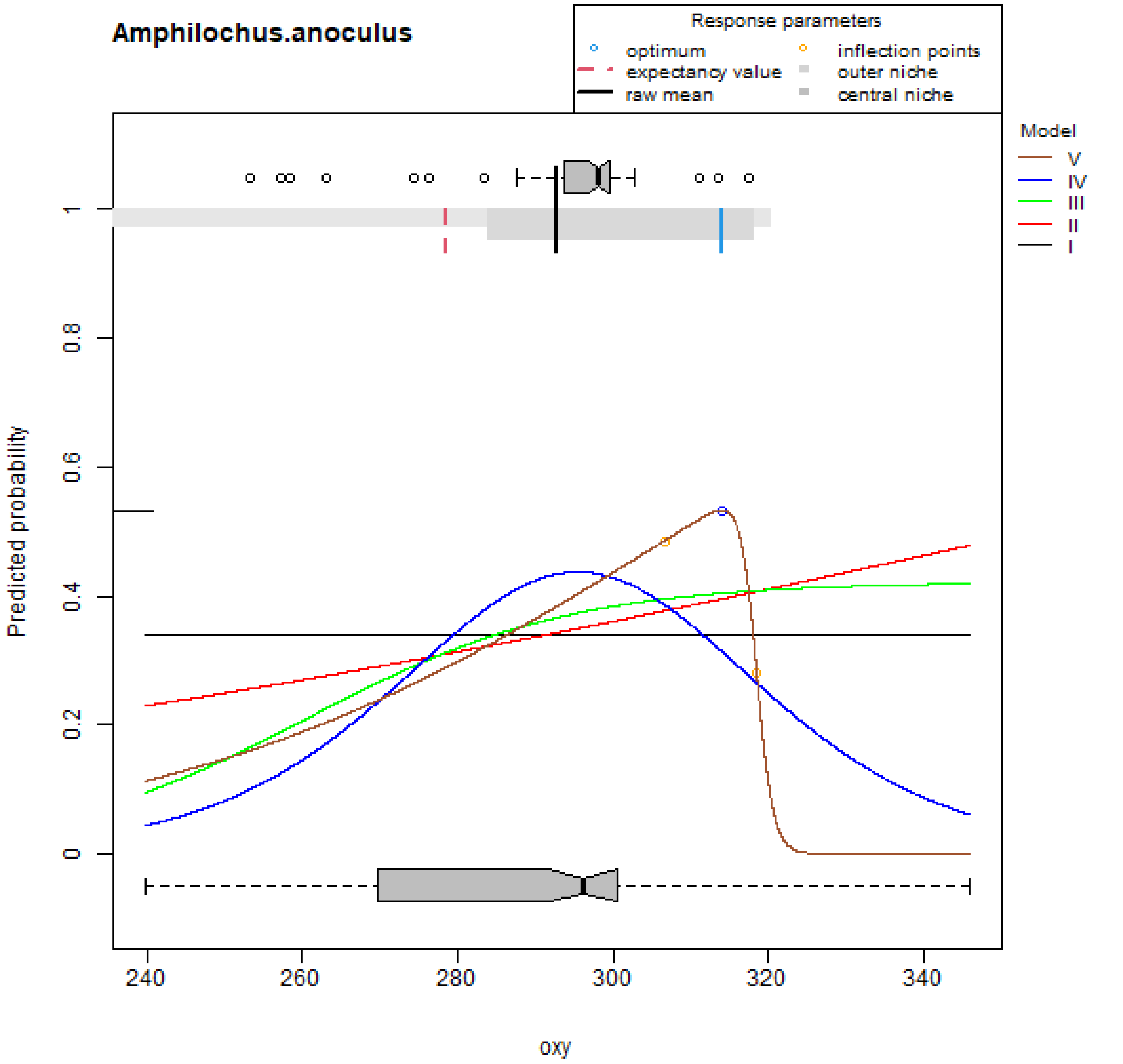

Amphilochus.anoculus
Response parameters
optimum
expectancy value
raw mean
inflection points
outer niche
central niche
Model
V
IV
III
II
I
Predicted probability
0
0.2
0.4
0.6
0.8
1
240
260
280
300
320
340
oxy

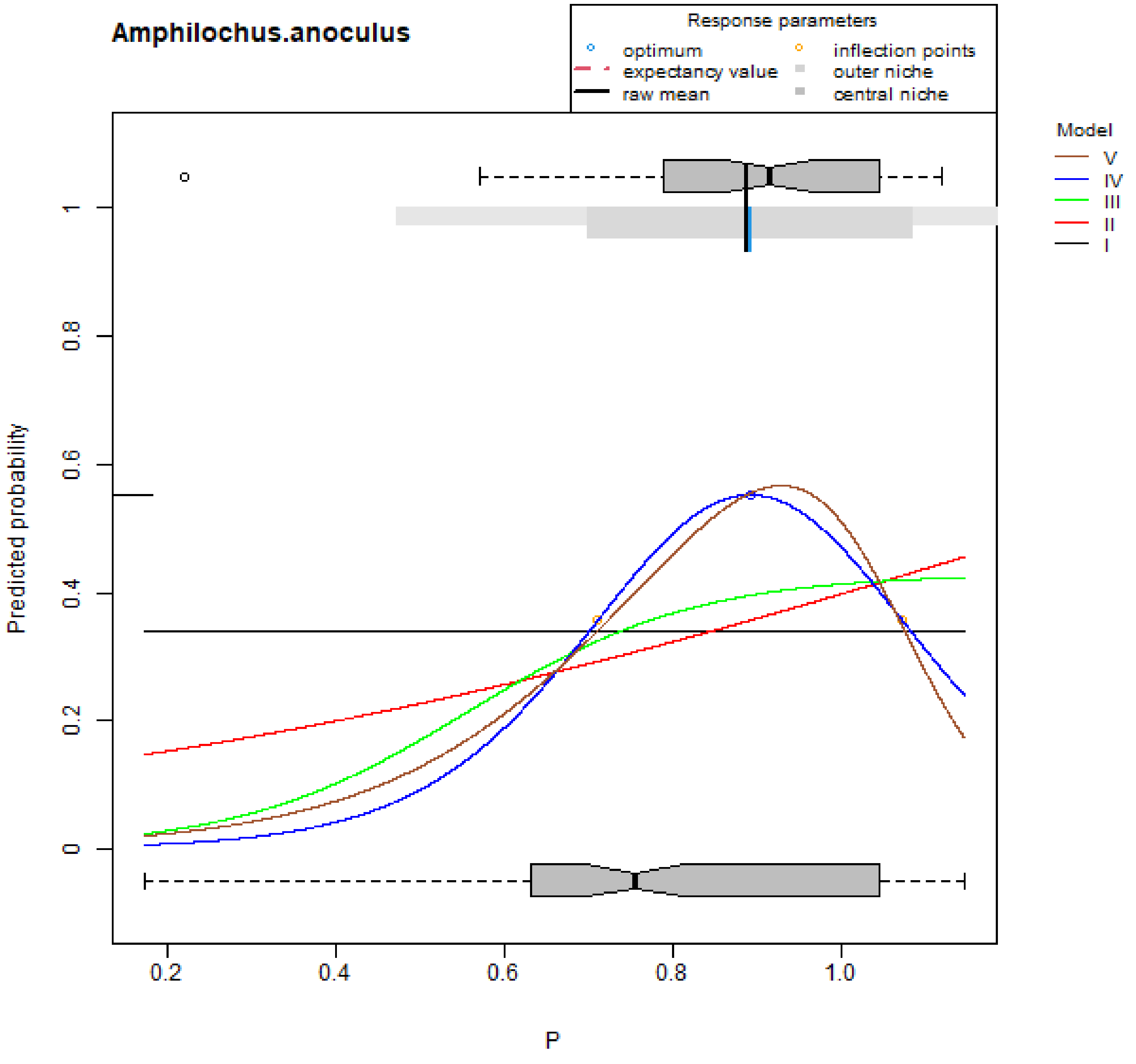

Amphilochus.anoculus
Response parameters
optimum
expectancy value
raw mean
inflection points
outer niche
central niche
Model
V
IV
III
II
I
Predicted probability
0
0.2
0.4
0.6
0.8
1
0.2
0.4
0.6
0.8
1.0
P

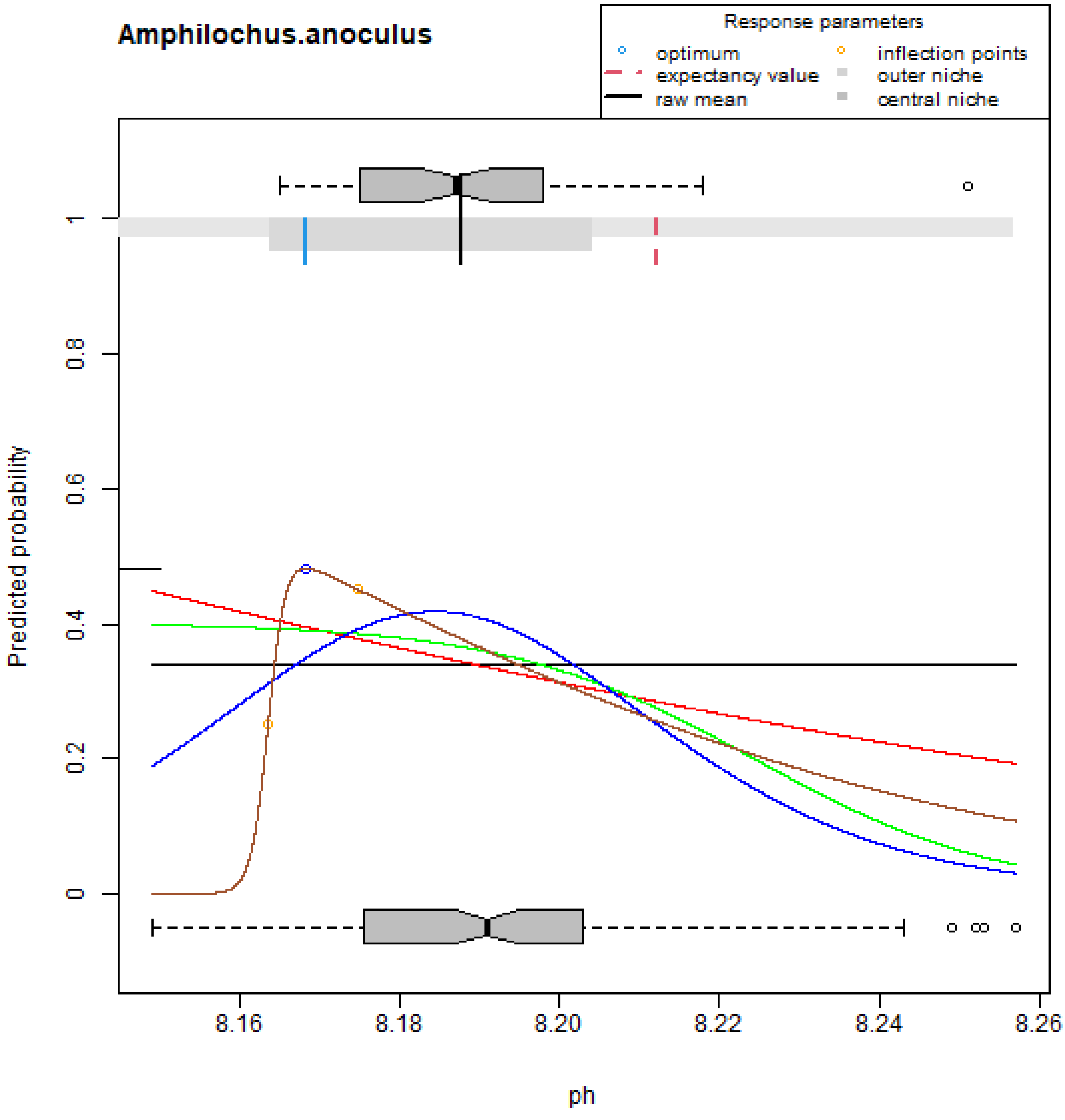
Amphilochus.anoculus
Response parameters
optimum
expectancy value
raw mean
inflection points
outer niche
central niche
Predicted probability
0
0.2
0.4
0.6
0.8
1
8.16
8.18
8.20
8.22
8.24
8.26
ph

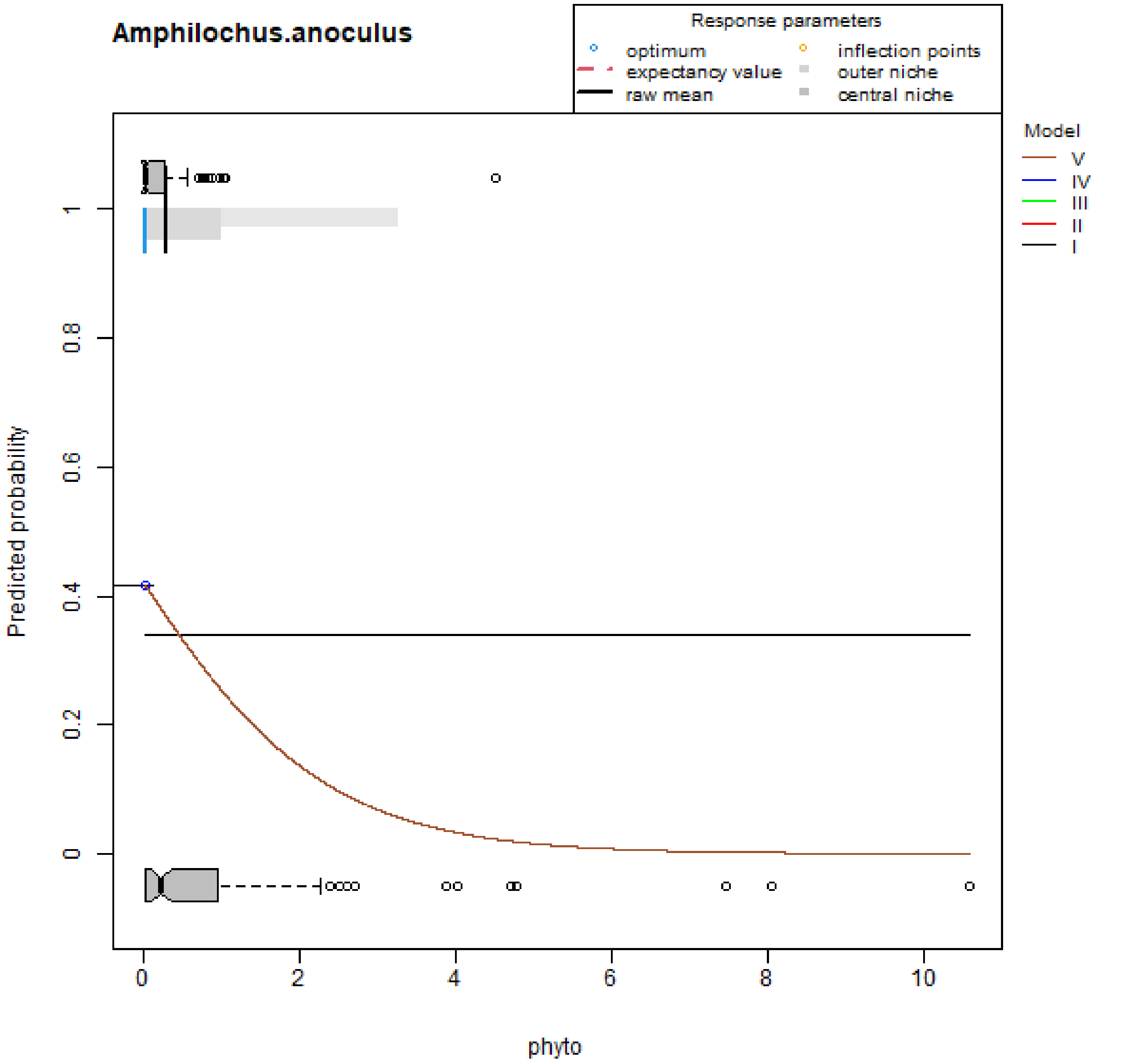

Amphilochus.anoculus
Response parameters
optimum
expectancy value
raw mean
inflection points
outer niche
central niche
Model
V
IV
III
II
I
Predicted probability
0
0.2
0.4
0.6
0.8
1
0
2
4
6
8
10
phyto

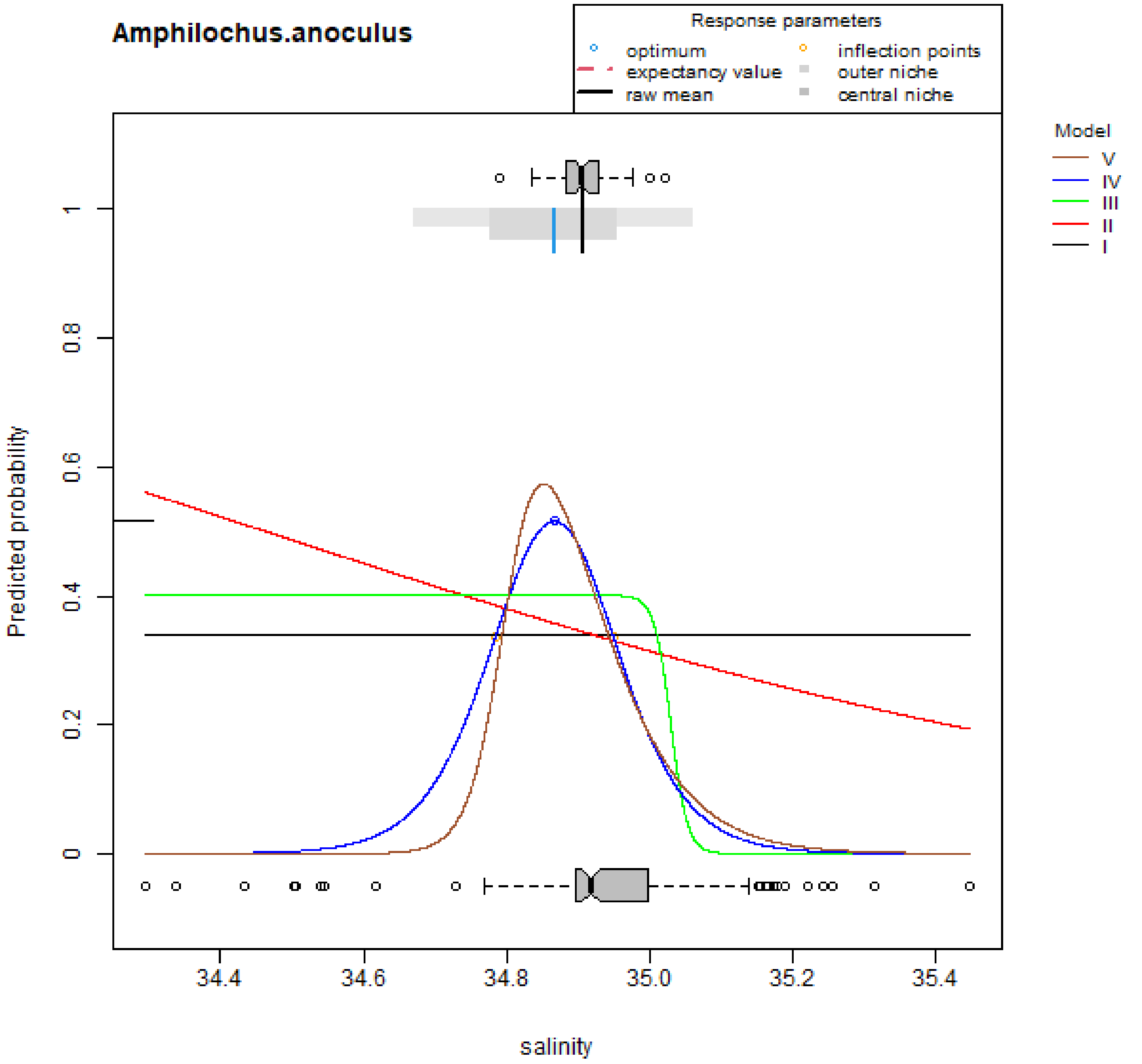

Amphilochus.anoculus
Response parameters
optimum
expectancy value
raw mean
inflection points
outer niche
central niche
Model
V
IV
III
II
I
Predicted probability
0
0.2
0.4
0.6
0.8
1
34.4
34.6
34.8
35.0
35.2
35.4
salinity

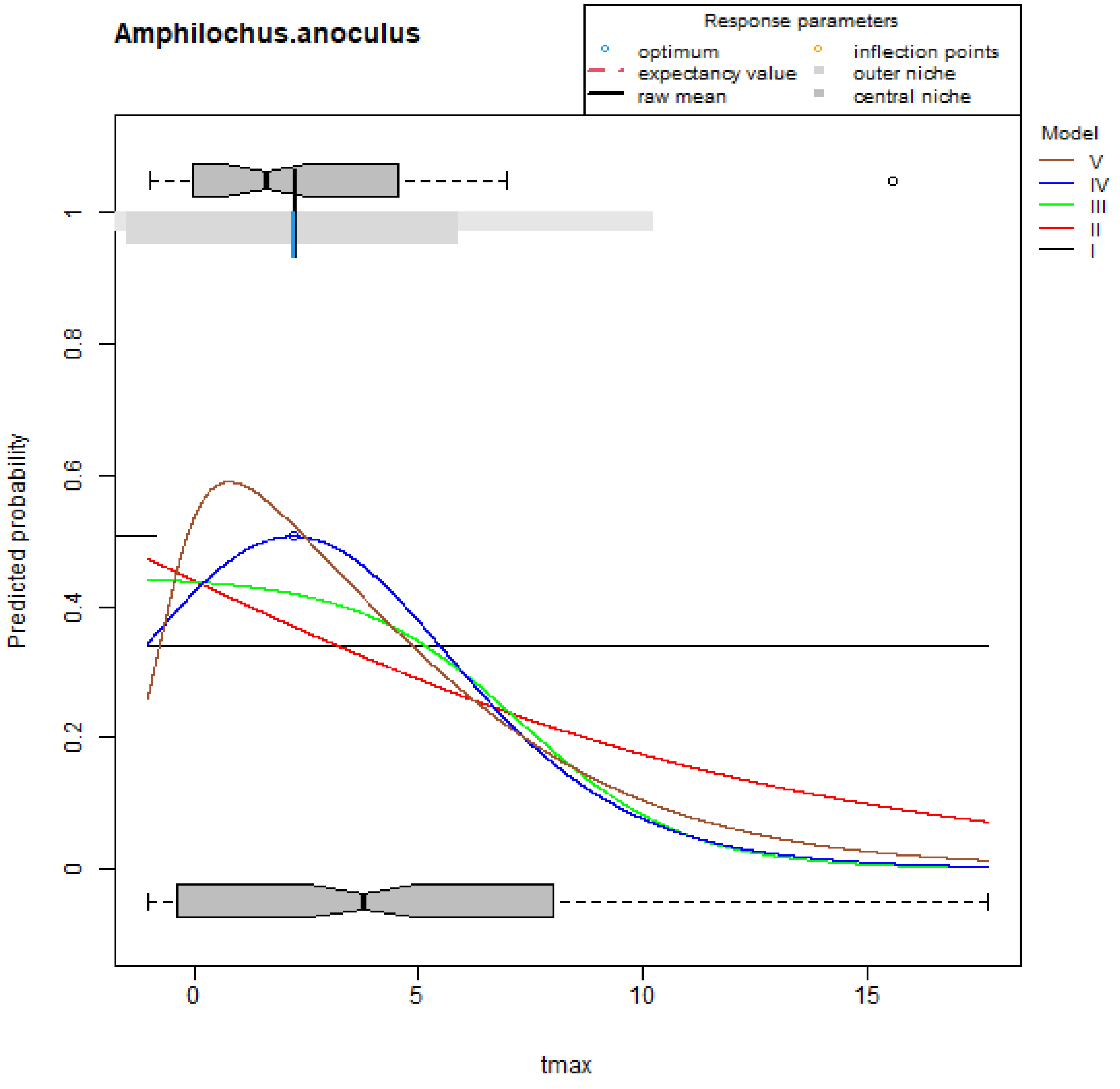

Amphilochus.anoculus
Response parameters
optimum
expectancy value
raw mean
inflection points
outer niche
central niche
Model
V
IV
III
II
I
Predicted probability
0
0.2
0.4
0.6
0.8
1
0
5
10
15
tmax

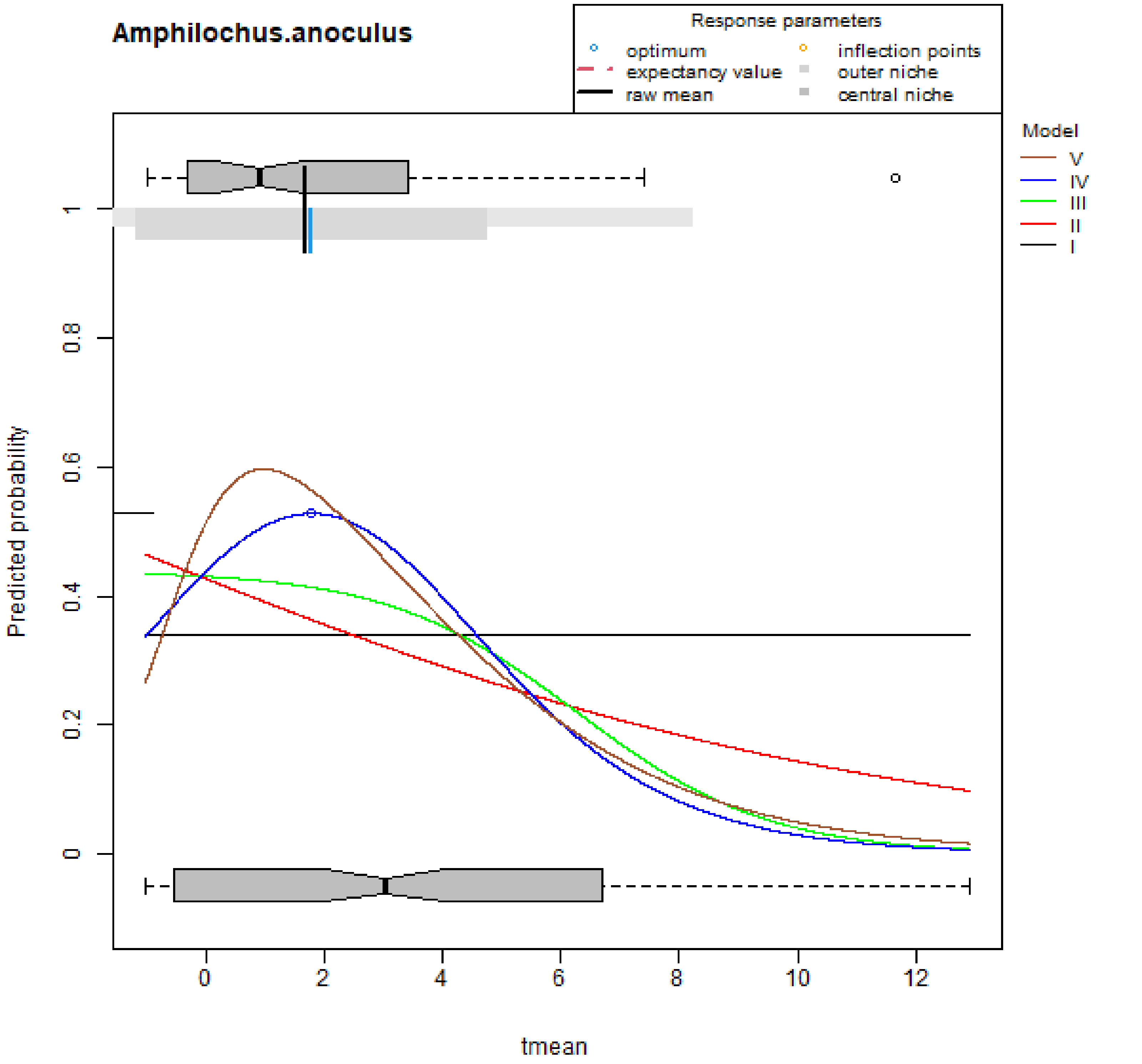

Amphilochus.anoculus
Response parameters
optimum
expectancy value
raw mean
inflection points
outer niche
central niche
Model
V
IV
III
II
I
Predicted probability
0
0.2
0.4
0.6
0.8
1
0
2
4
6
8
10
12
tmean

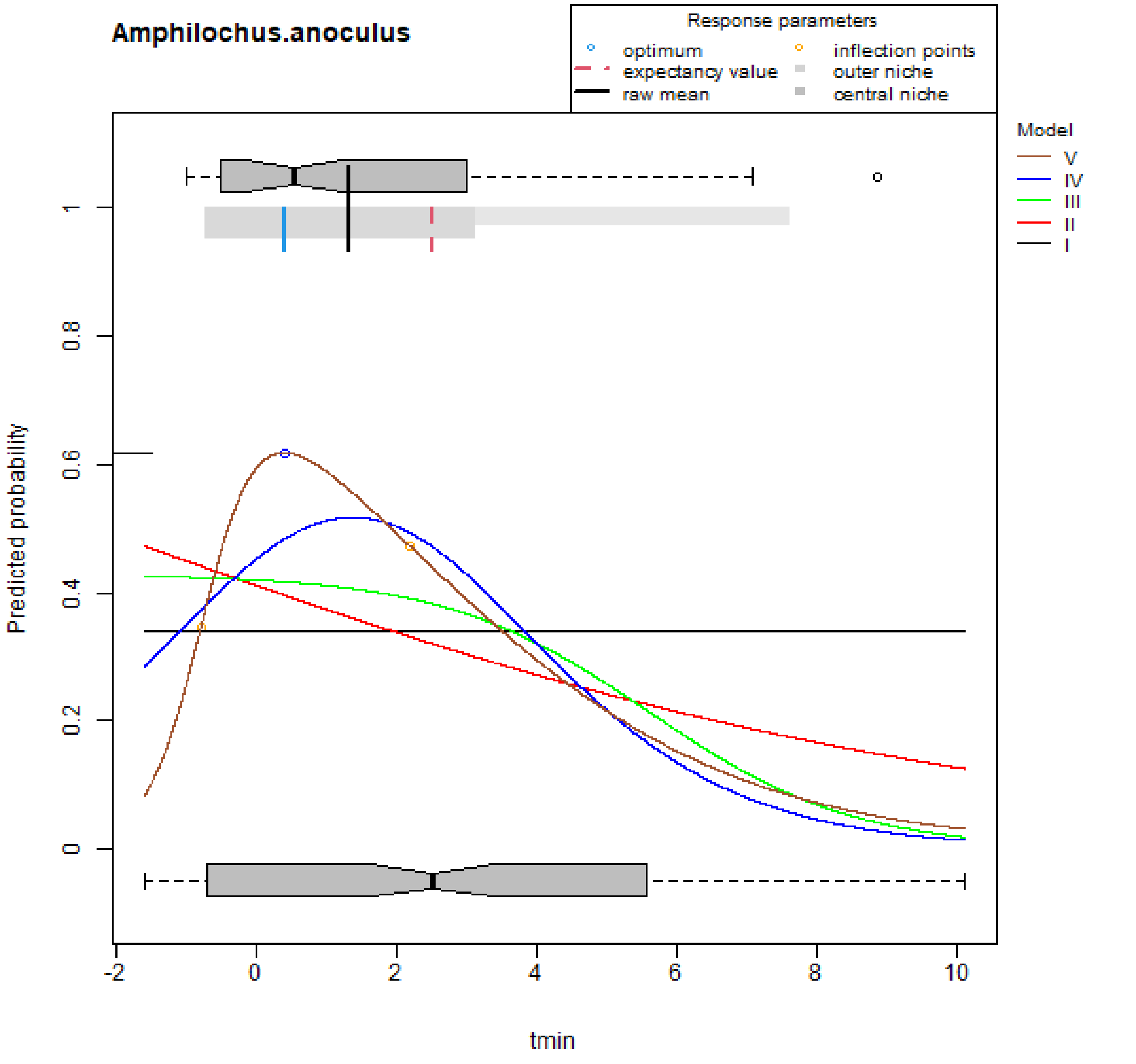

Amphilochus.anoculus
Response parameters
optimum
expectancy value
raw mean
inflection points
outer niche
central niche
Model
V
IV
III
II
I
Predicted probability
0
0.2
0.4
0.6
0.8
1
-2
0
2
4
6
8
10
tmin

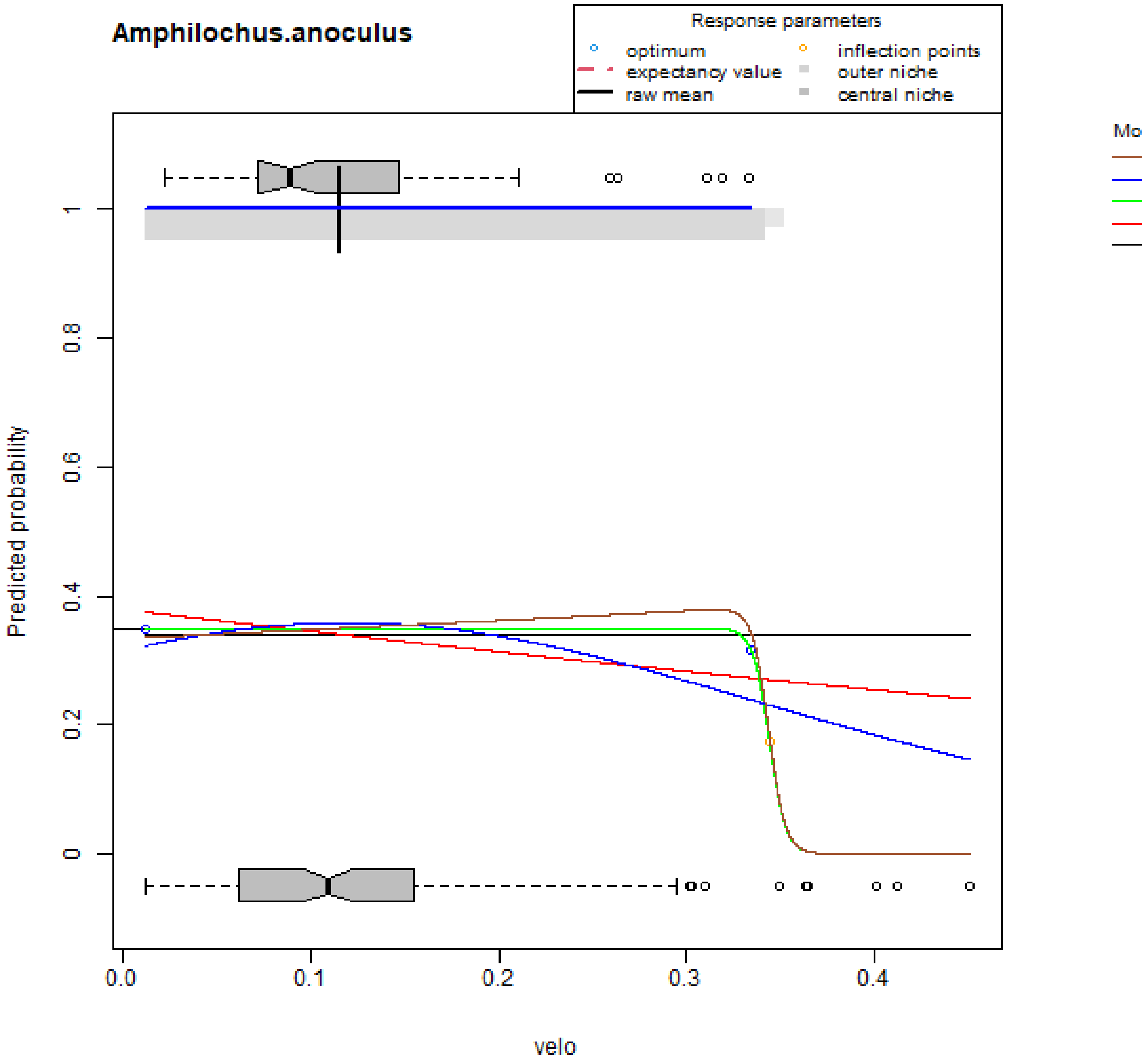

Amphilochus.anoculus
Response parameters
optimum
expectancy value
raw mean
inflection points
outer niche
central niche
Mo
Predicted probability
0
0.2
0.4
0.6
0.8
1
0.0
0.1
0.2
0.3
0.4
velo

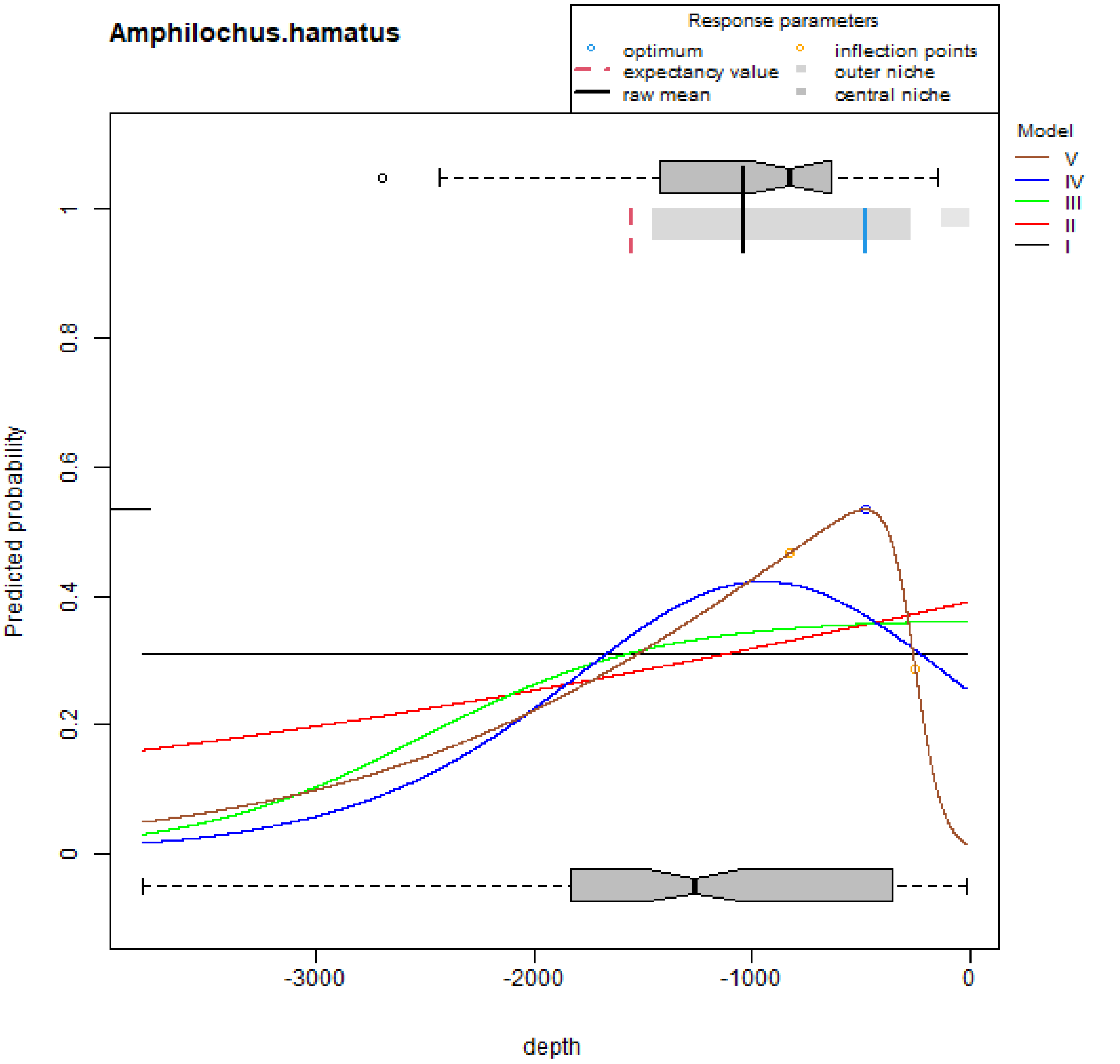

Amphilochus.hamatus
Response parameters
optimum
expectancy value
raw mean
inflection points
outer niche
central niche
Model
V
IV
III
II
I
Predicted probability
0
0.2
0.4
0.6
0.8
1
-3000
-2000
-1000
0
depth

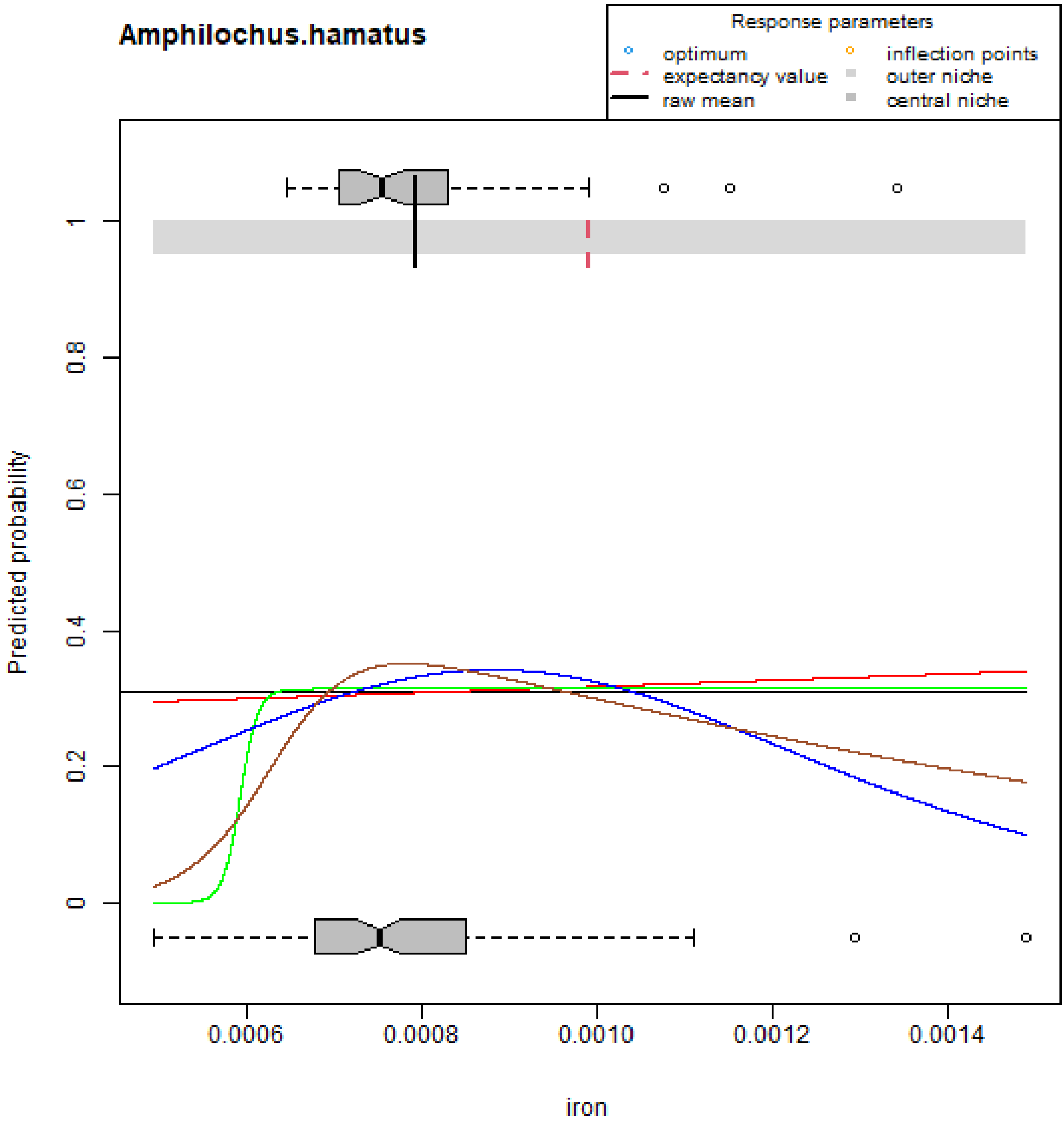

Amphilochus.hamatus
Response parameters
optimum
expectancy value
raw mean
inflection points
outer niche
central niche
Predicted probability
0
0.2
0.4
0.6
0.8
1
0.0006
0.0008
0.0010
0.0012
0.0014
iron

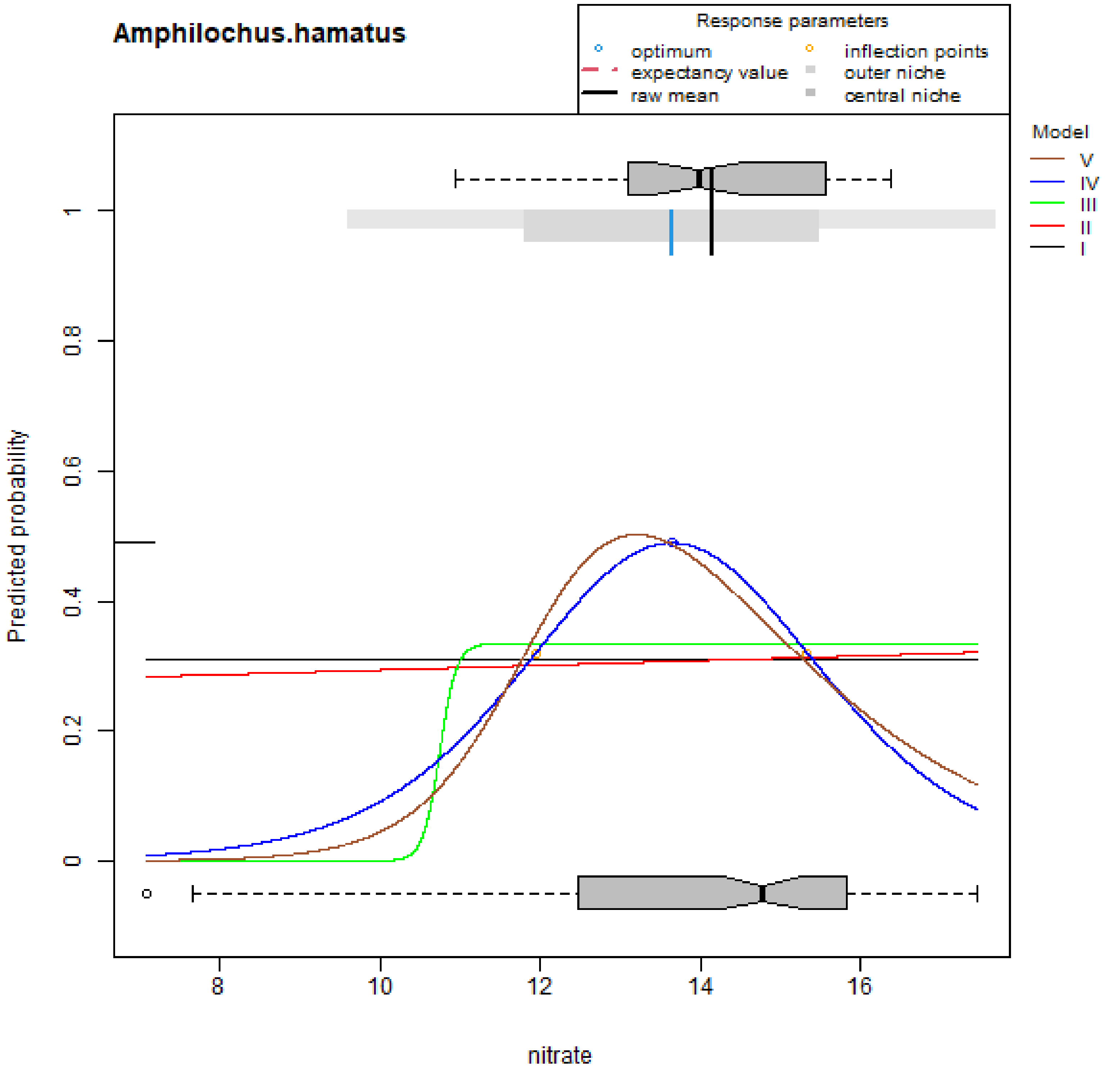
Amphilochus.hamatus
Response parameters
optimum
inflection points
expectancy value
outer niche
raw mean
central niche
Model
V
IV
III
II
I
Predicted probability
0
0.2
0.4
0.6
0.8
1
8
10
12
14
16
nitrate

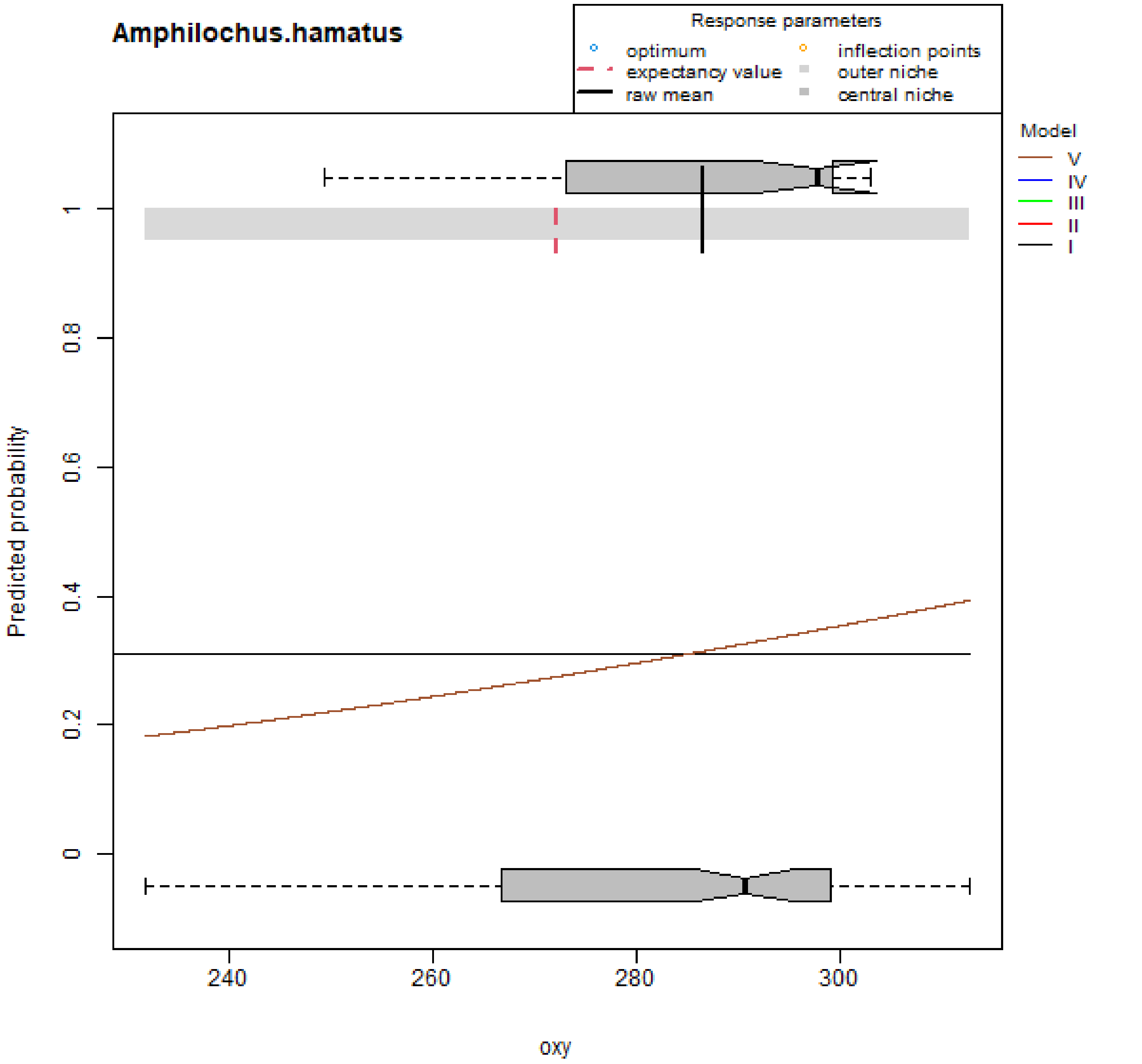

Amphilochus.hamatus
Response parameters
optimum
expectancy value
raw mean
inflection points
outer niche
central niche
Model
V
IV
III
II
I
Predicted probability
0
0.2
0.4
0.6
0.8
1
240
260
280
300
oxy

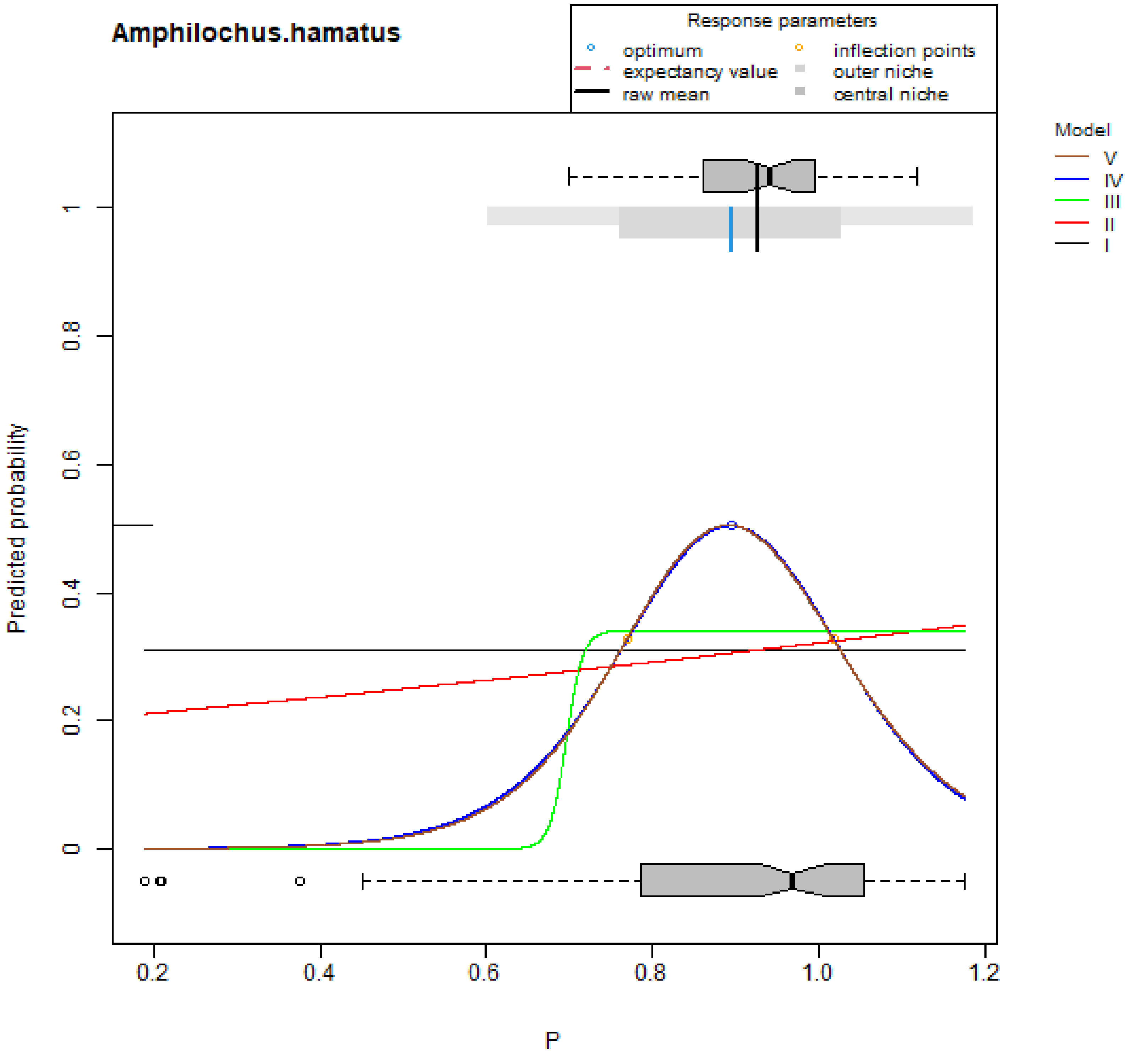

Amphilochus.hamatus
Response parameters
optimum
expectancy value
raw mean
inflection points
outer niche
central niche
Model
V
IV
III
II
I
Predicted probability
0
0.2
0.4
0.6
0.8
1
0.2
0.4
0.6
0.8
1.0
1.2
P

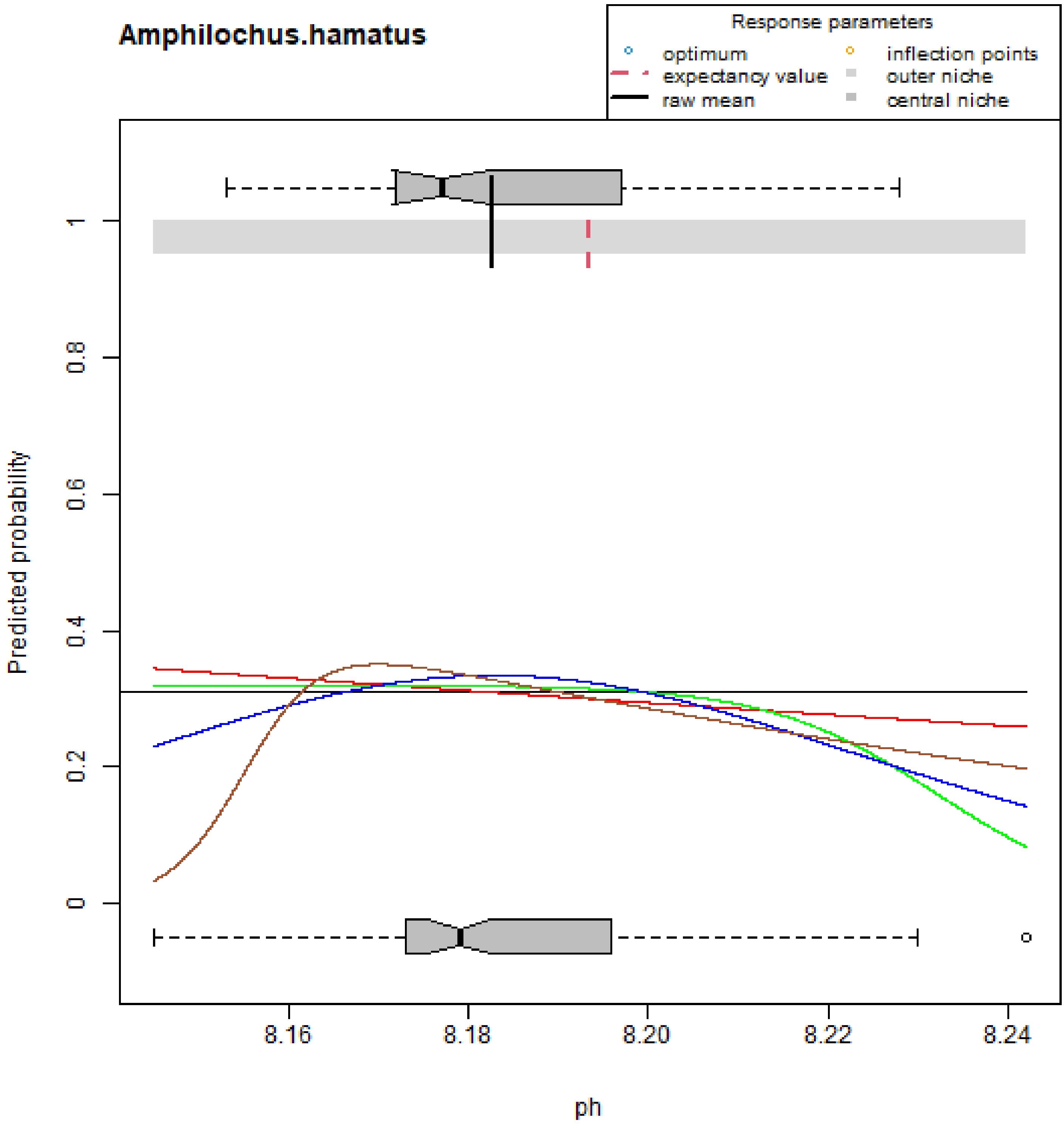
Amphilochus.hamatus
Response parameters
optimum
expectancy value
raw mean
inflection points
outer niche
central niche
Predicted probability
0
0.2
0.4
0.6
0.8
1
8.16
8.18
8.20
8.22
8.24
ph

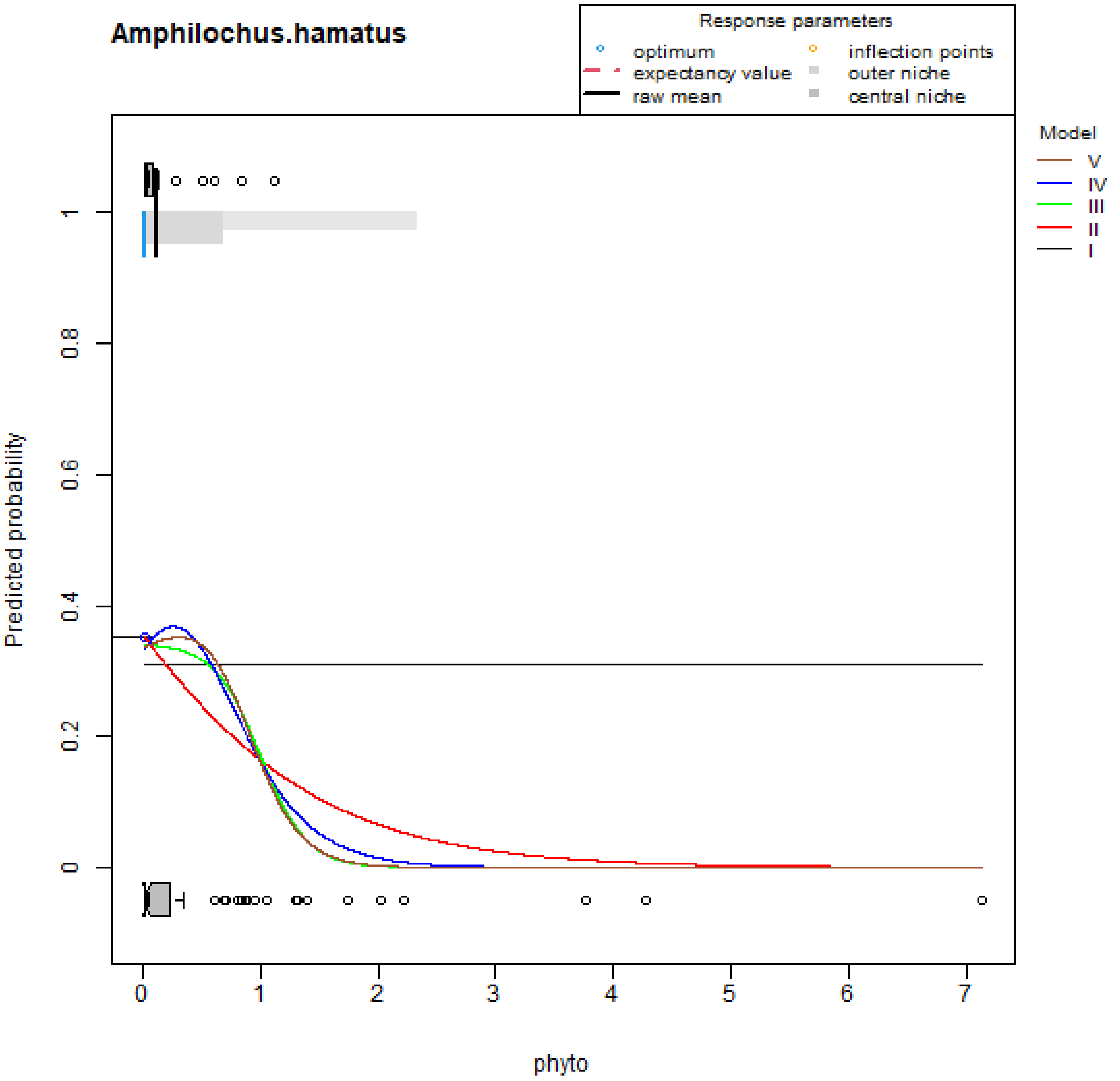
Amphilochus.hamatus
Response parameters
optimum
expectancy value
raw mean
inflection points
outer niche
central niche
Model
V
IV
III
II
I
Predicted probability
0
0.2
0.4
0.6
0.8
1
0
1
2
3
4
5
6
7
phyto

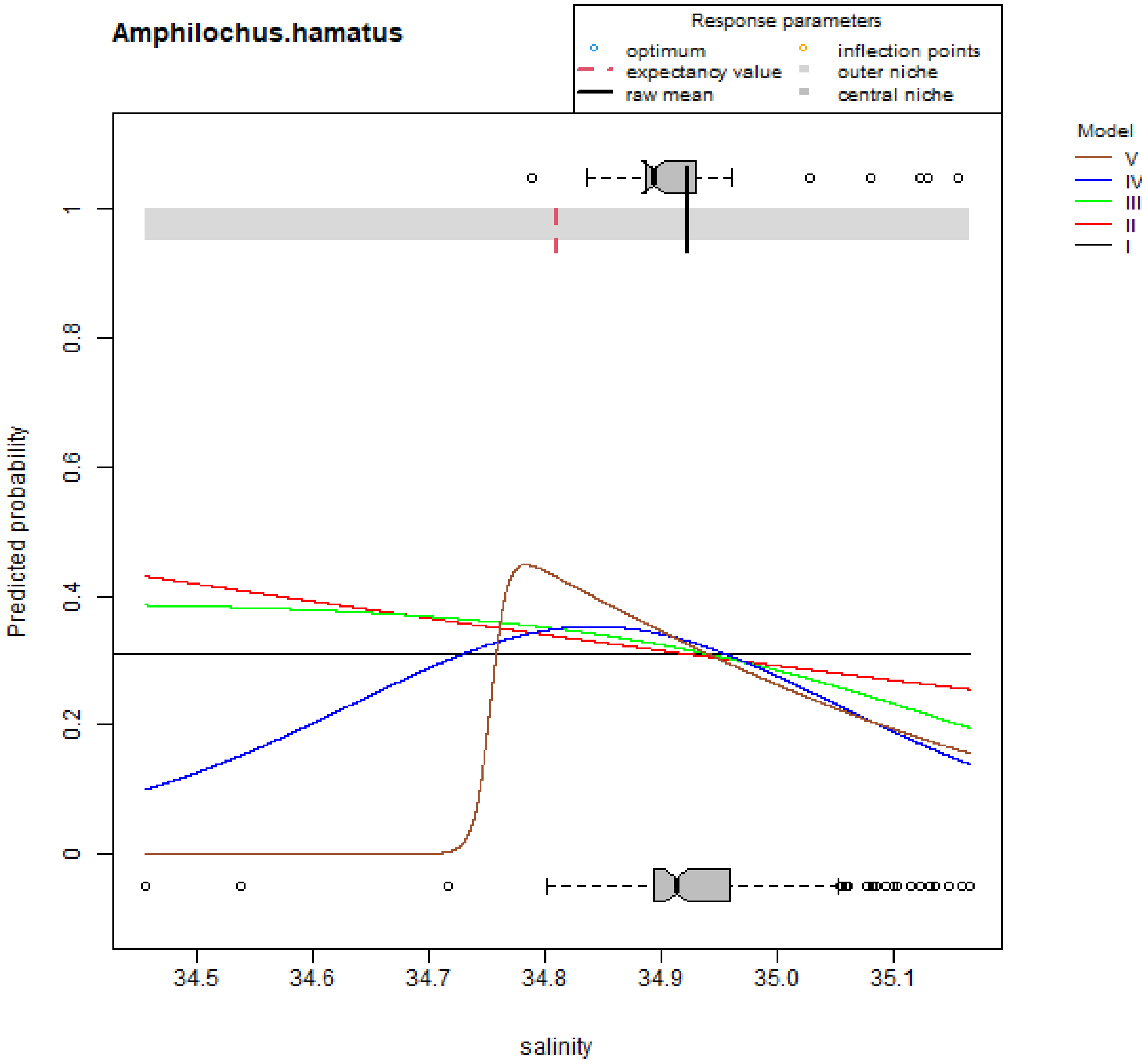
Amphilochus.hamatus
Response parameters
optimum
inflection points
expectancy value
outer niche
raw mean
central niche
Model
V
IV
III
II
I
Predicted probability
0
0.2
0.4
0.6
0.8
1
34.5
34.6
34.7
34.8
34.9
35.0
35.1
salinity

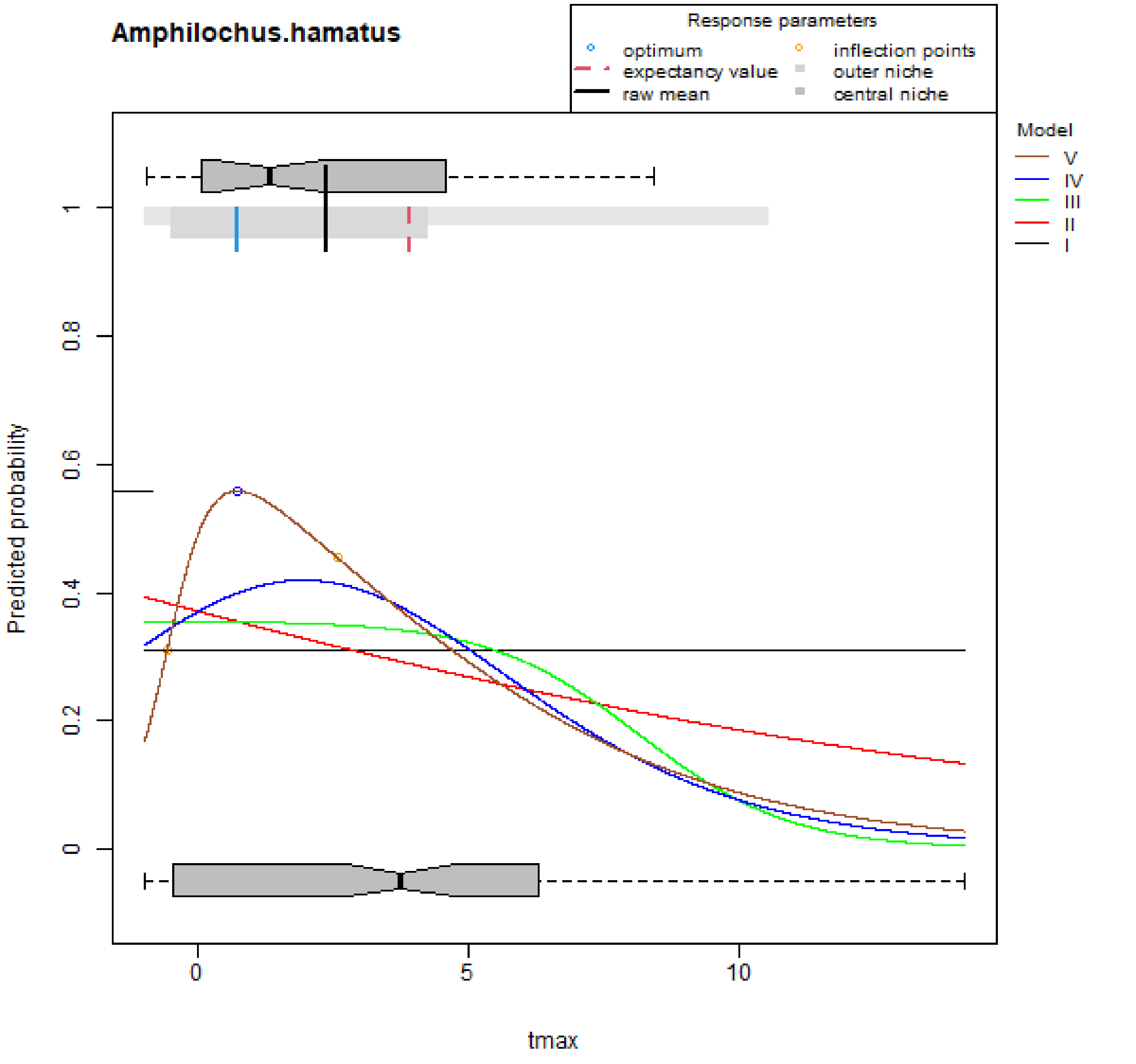
Amphilochus.hamatus
Response parameters
optimum
expectancy value
raw mean
inflection points
outer niche
central niche
Model
V
IV
III
II
I
Predicted probability
0
0.2
0.4
0.6
0.8
1
0
5
10
tmax

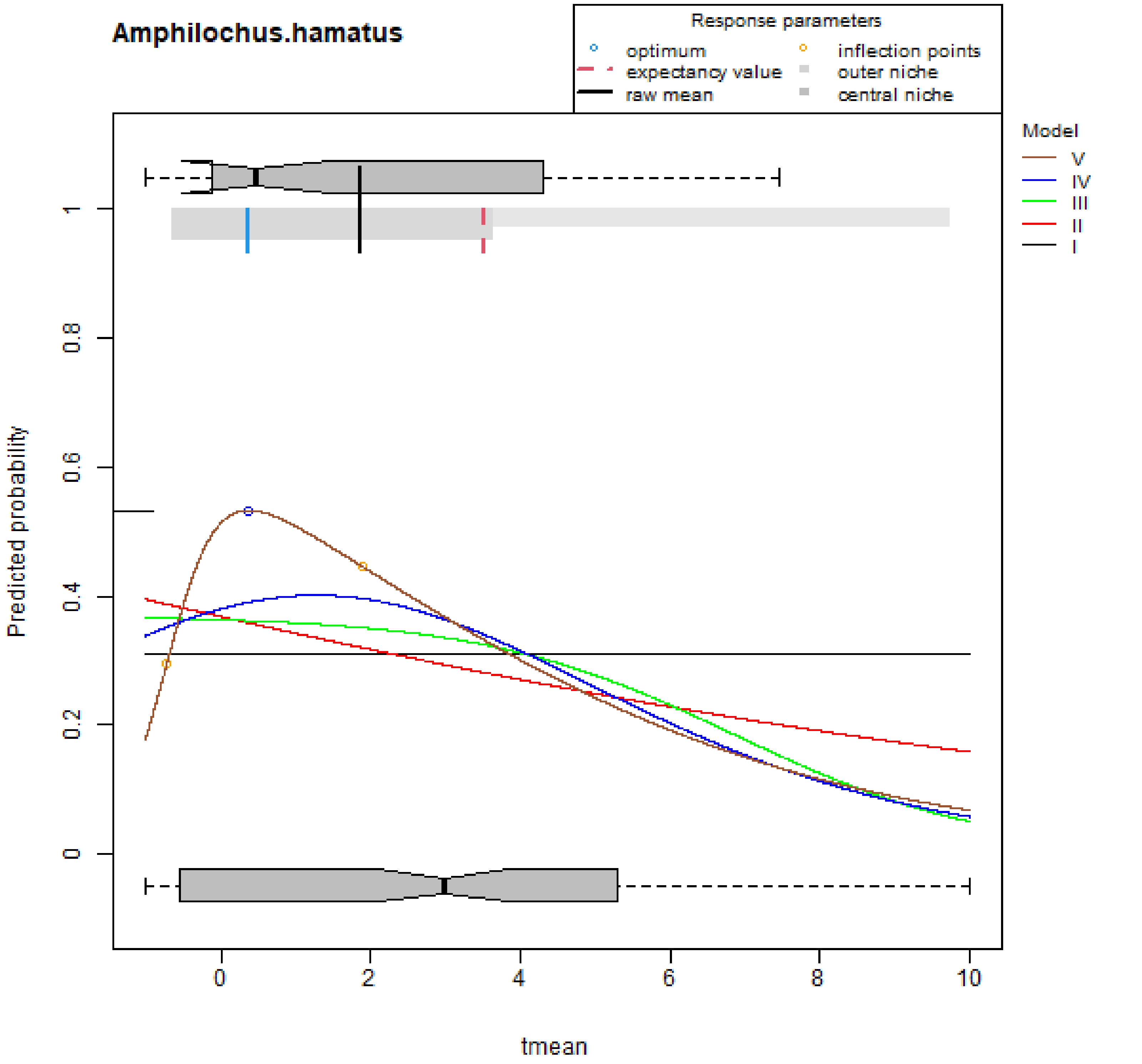

Amphilochus.hamatus
Response parameters
optimum
expectancy value
raw mean
inflection points
outer niche
central niche
Model
V
IV
III
II
I
Predicted probability
0
0.2
0.4
0.6
0.8
1
0
2
4
6
8
10
tmean

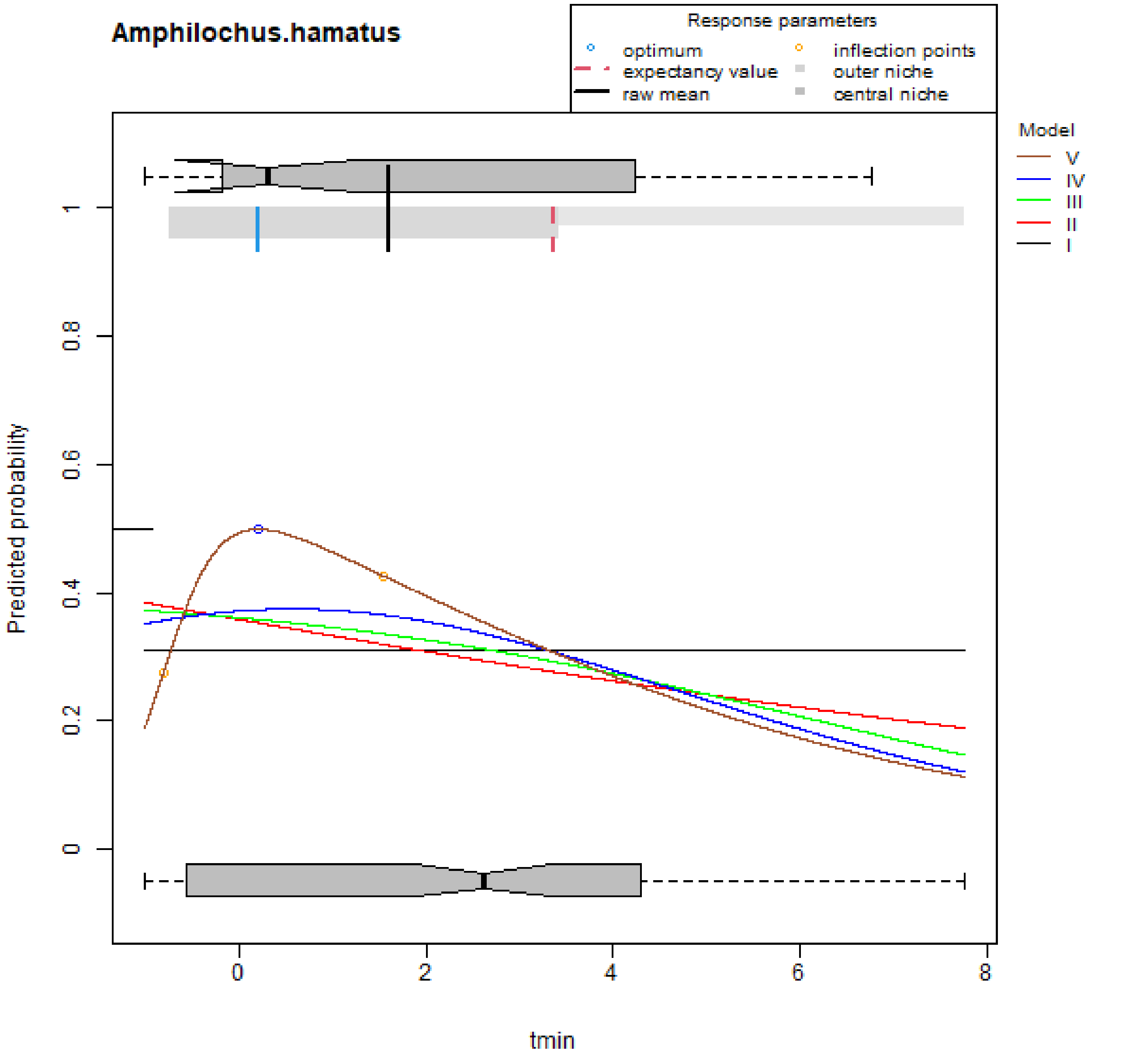
Amphilochus.hamatus
Response parameters
optimum
expectancy value
raw mean
inflection points
outer niche
central niche
Model
V
IV
III
II
I
Predicted probability
0
0.2
0.4
0.6
0.8
1
0
2
4
6
8
tmin

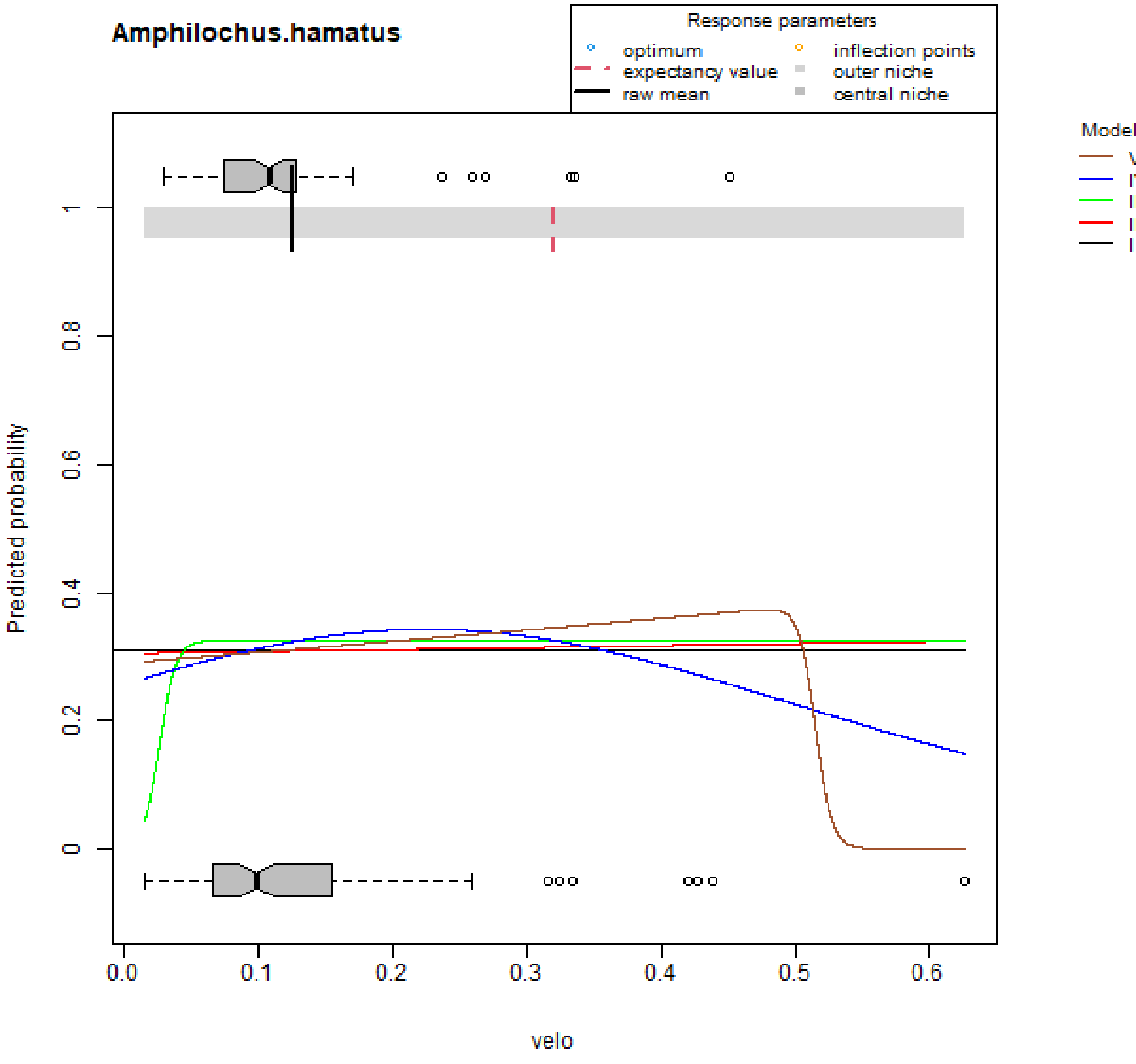
Amphilochus.hamatus
Response parameters
optimum
expectancy value
raw mean
inflection points
outer niche
central niche
Model
Predicted probability
0
0.2
0.4
0.6
0.8
1
0.0
0.1
0.2
0.3
0.4
0.5
0.6
velo

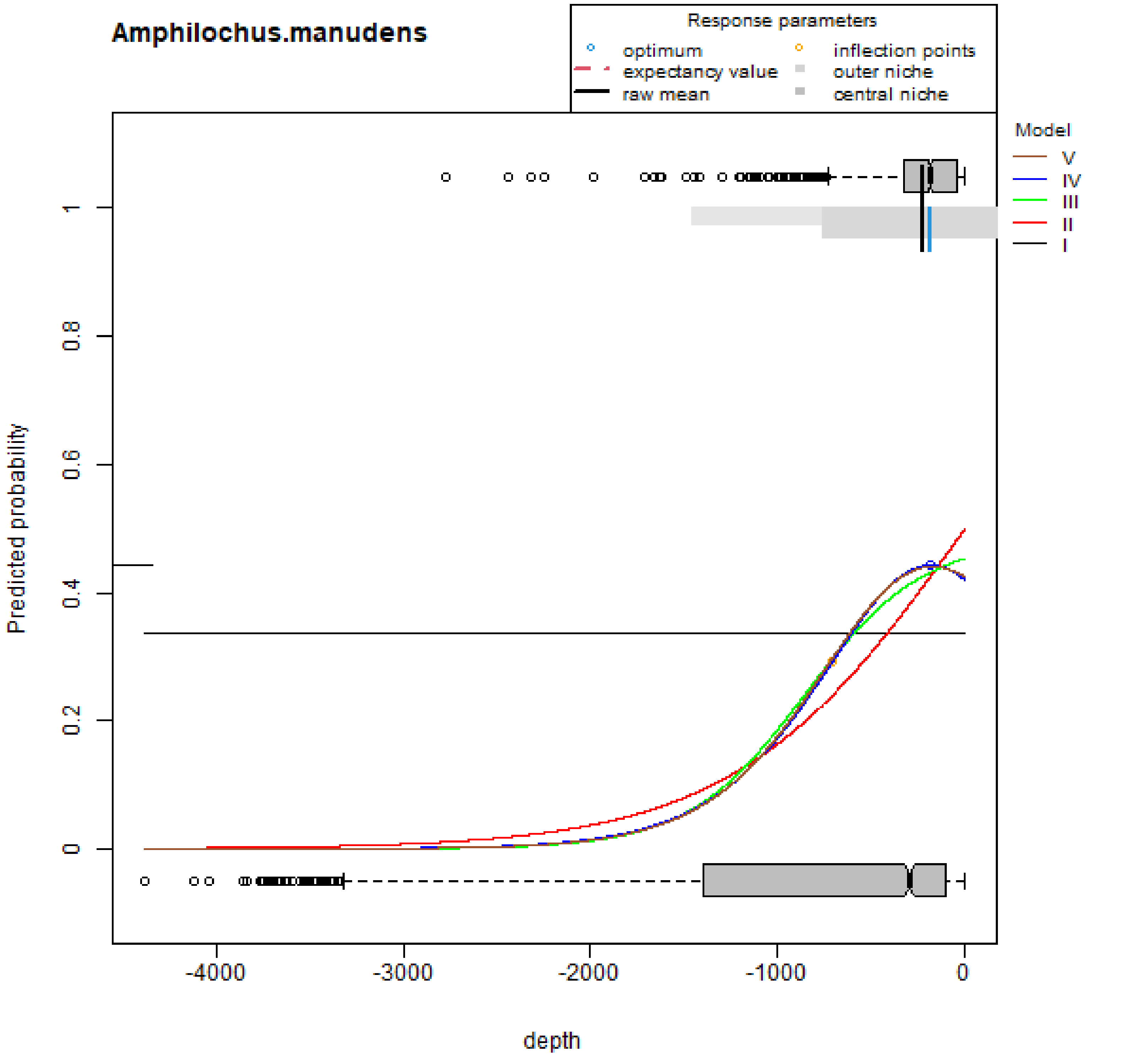

Amphilochus.manudens
Response parameters
optimum
expectancy value
raw mean
inflection points
outer niche
central niche
Model
V
IV
III
II
I
Predicted probability
0
0.2
0.4
0.6
0.8
1
-4000
-3000
-2000
-1000
0
depth

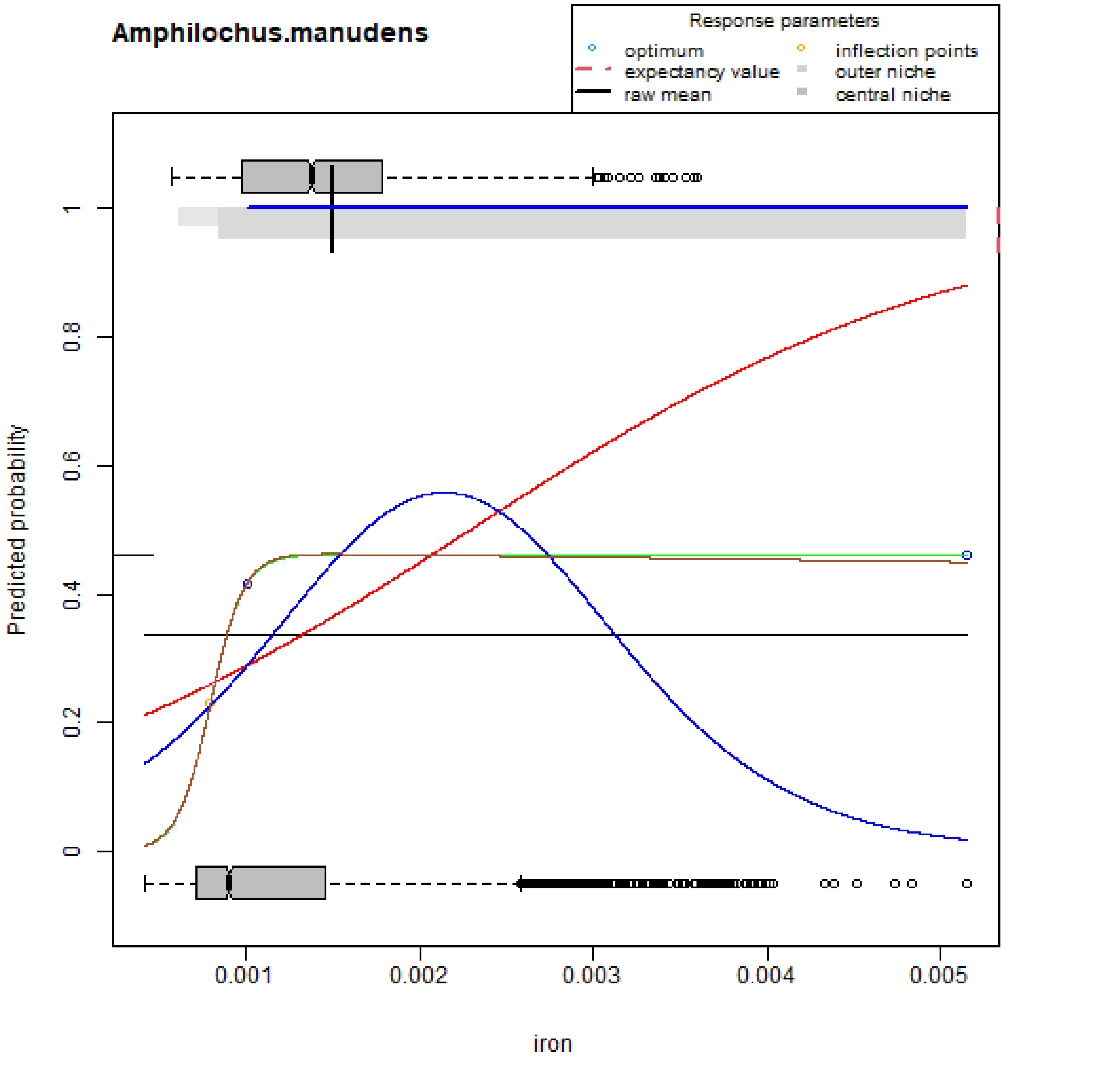

Amphilochus.manudens
Response parameters
optimum
inflection points
expectancy value
outer niche
raw mean
central niche
Predicted probability
0
0.2
0.4
0.6
0.8
1
0.001
0.002
0.003
0.004
0.005
iron

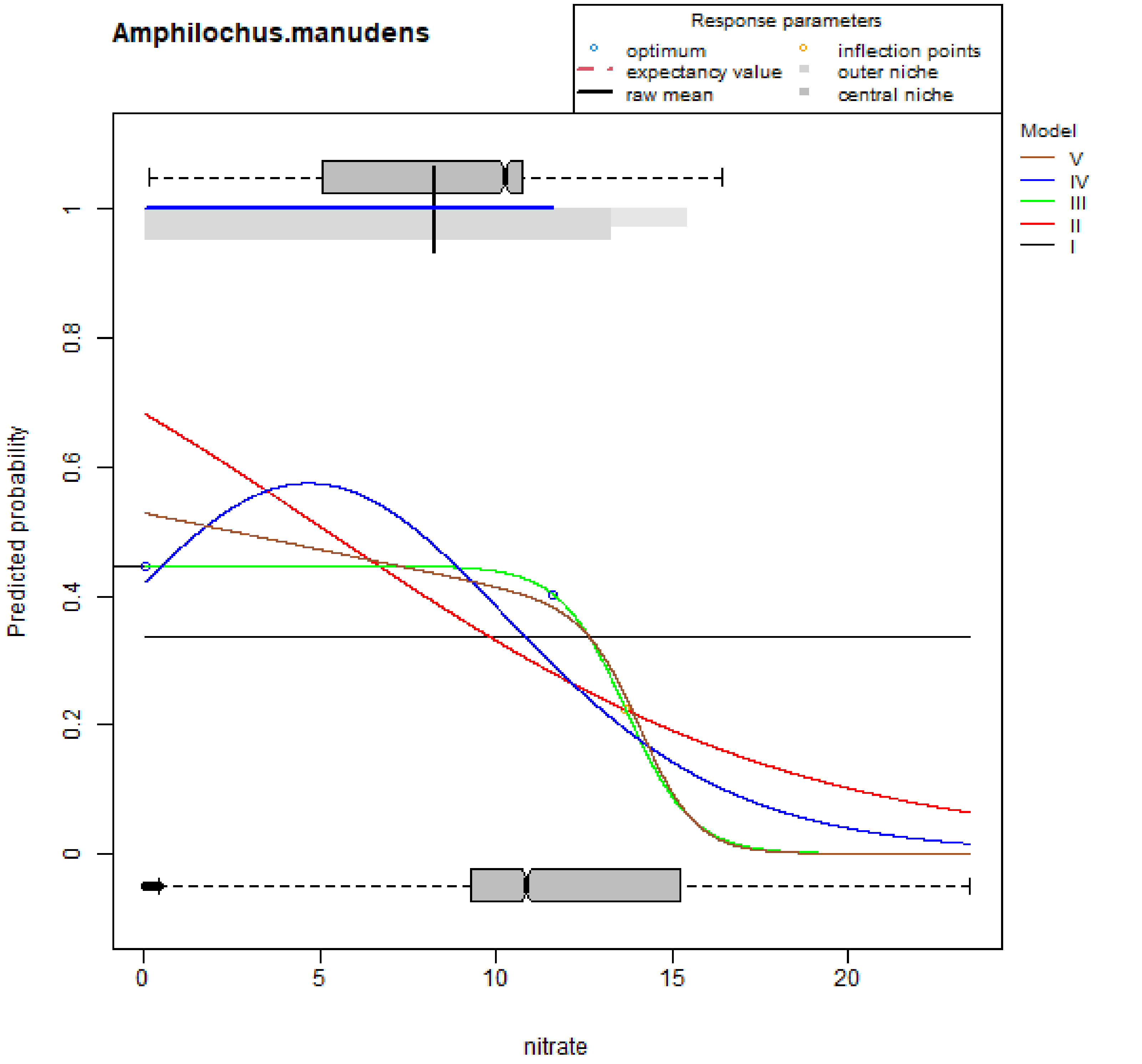

Amphilochus.manudens
Response parameters
optimum
expectancy value
raw mean
inflection points
outer niche
central niche
Model
V
IV
III
II
I
Predicted probability
0
0.2
0.4
0.6
0.8
1
0
5
10
15
20
nitrate

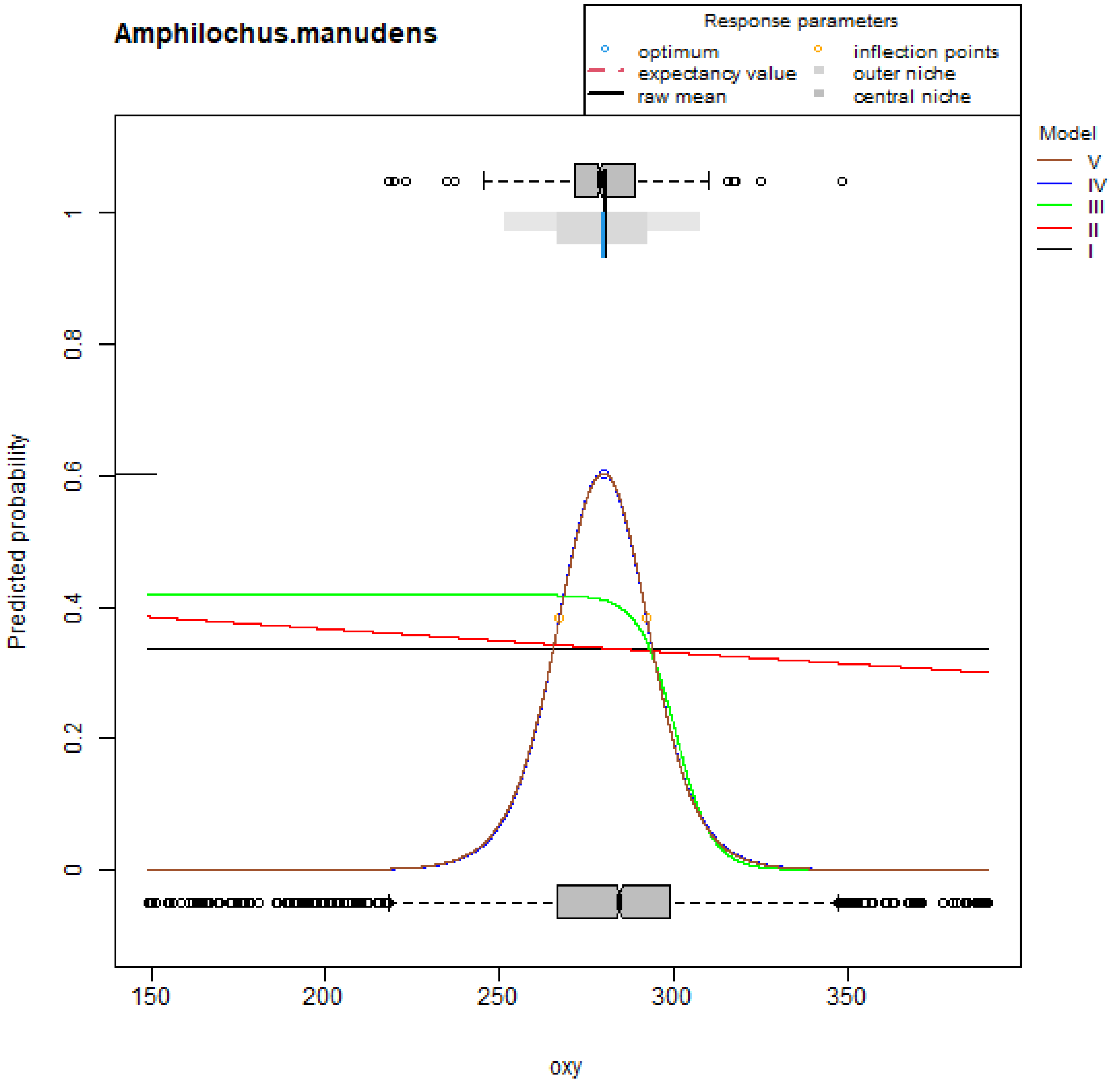

Amphilochus.manudens
Response parameters
optimum
expectancy value
raw mean
inflection points
outer niche
central niche
Model
V
IV
III
II
I
Predicted probability
0
0.2
0.4
0.6
0.8
1
150
200
250
300
350
oxy

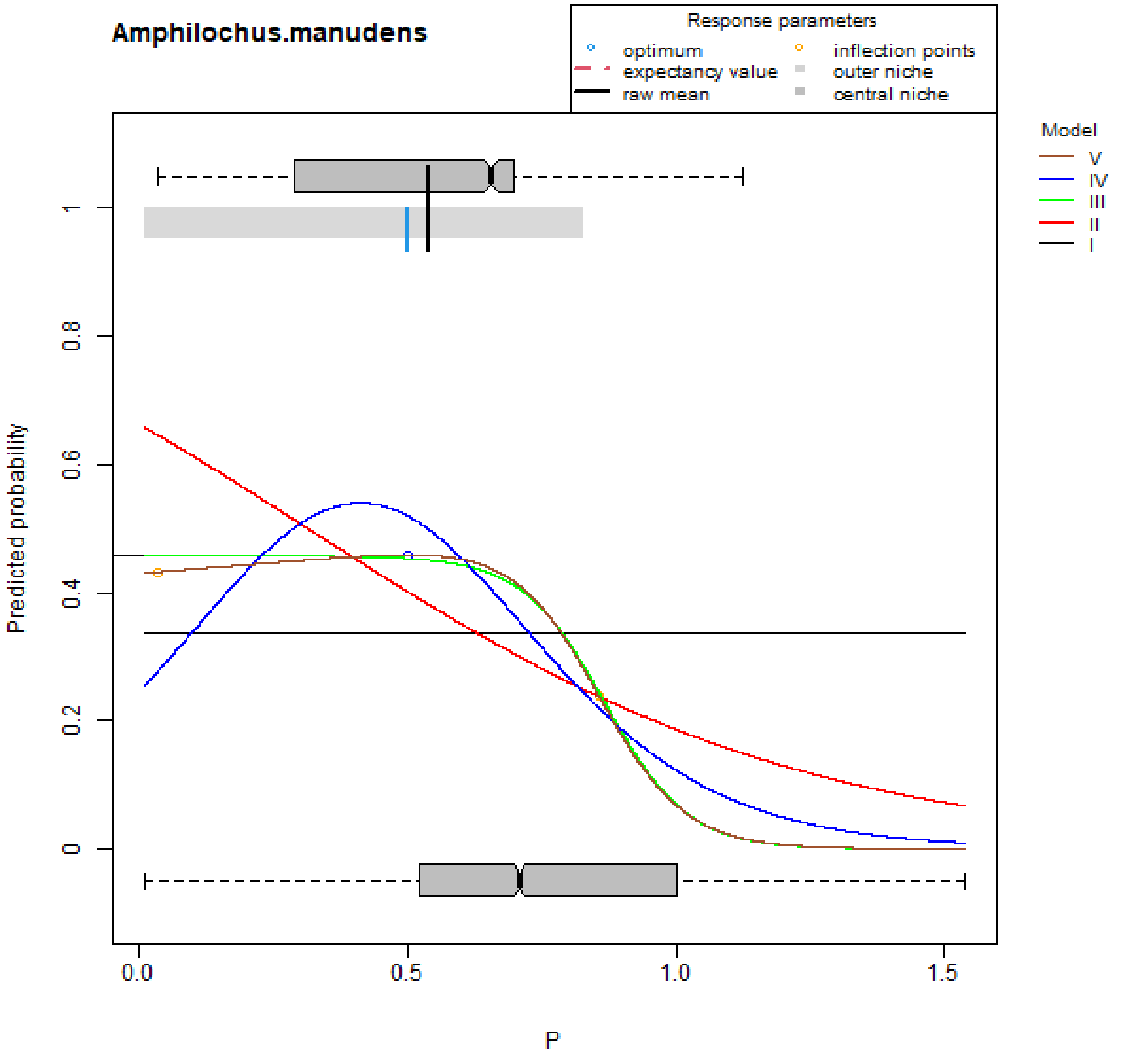

Amphilochus.manudens
Response parameters
optimum
inflection points
expectancy value
outer niche
raw mean
central niche
Model
V
IV
III
II
I
Predicted probability
0
0.2
0.4
0.6
0.8
1
0.0
0.5
1.0
1.5
P

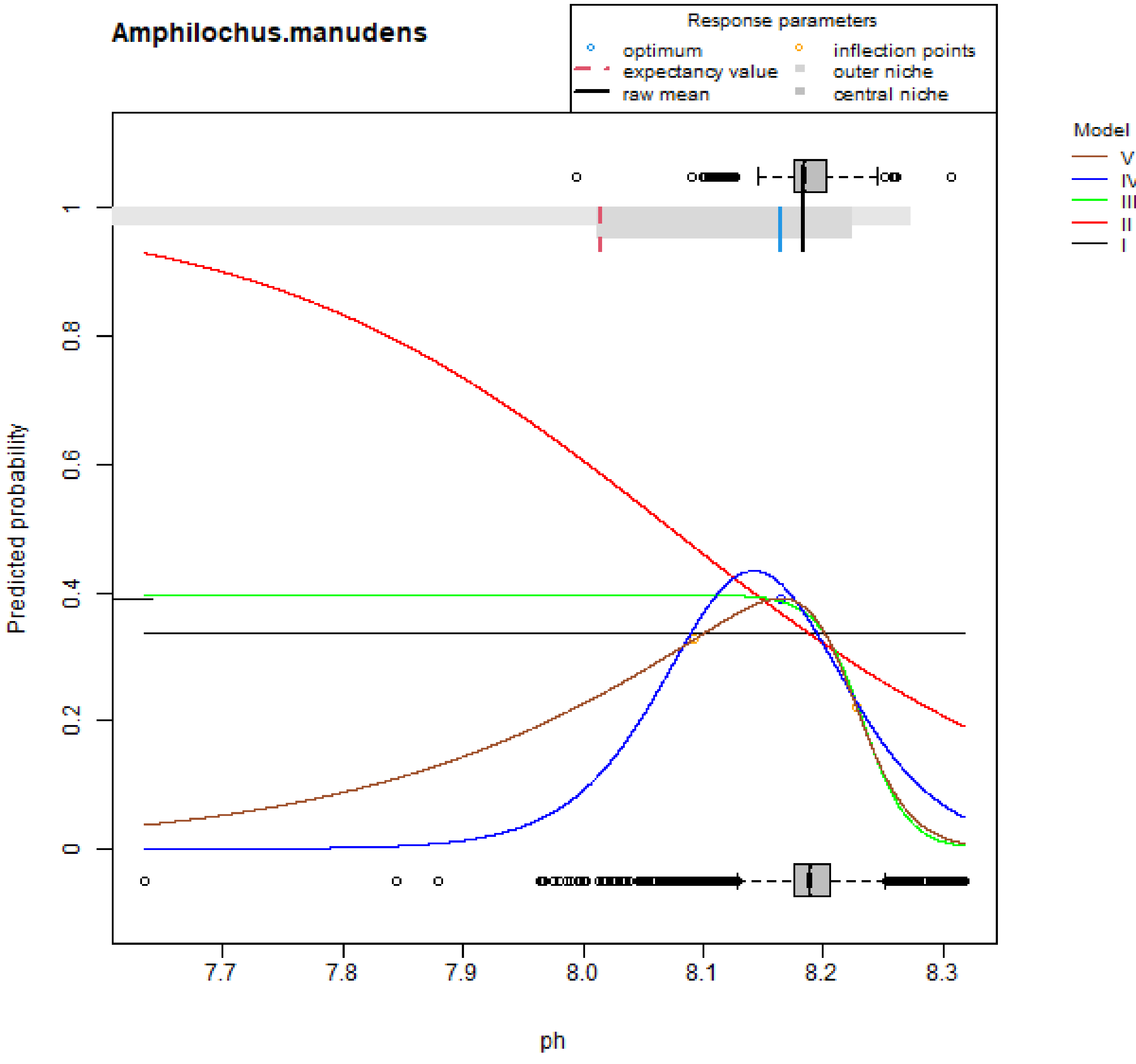

Amphilochus.manudens
Response parameters
optimum
expectancy value
raw mean
inflection points
outer niche
central niche
Model
Predicted probability
0
0.2
0.4
0.6
0.8
1
7.7
7.8
7.9
8.0
8.1
8.2
8.3
ph

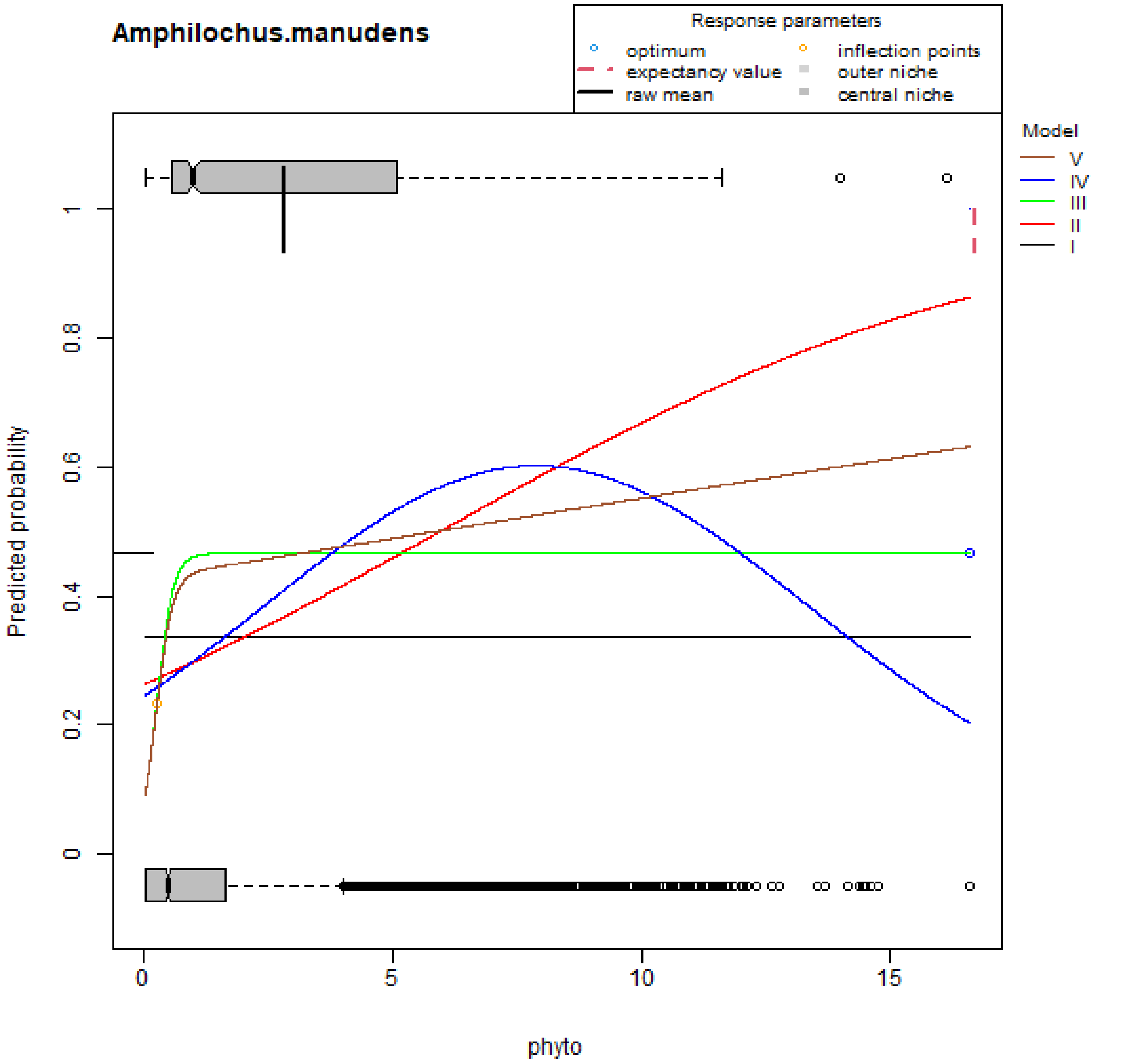

Amphilochus.manudens
Response parameters
optimum
expectancy value
raw mean
inflection points
outer niche
central niche
Model
V
IV
III
II
I
Predicted probability
0
0.2
0.4
0.6
0.8
1
0
5
10
15
phyto

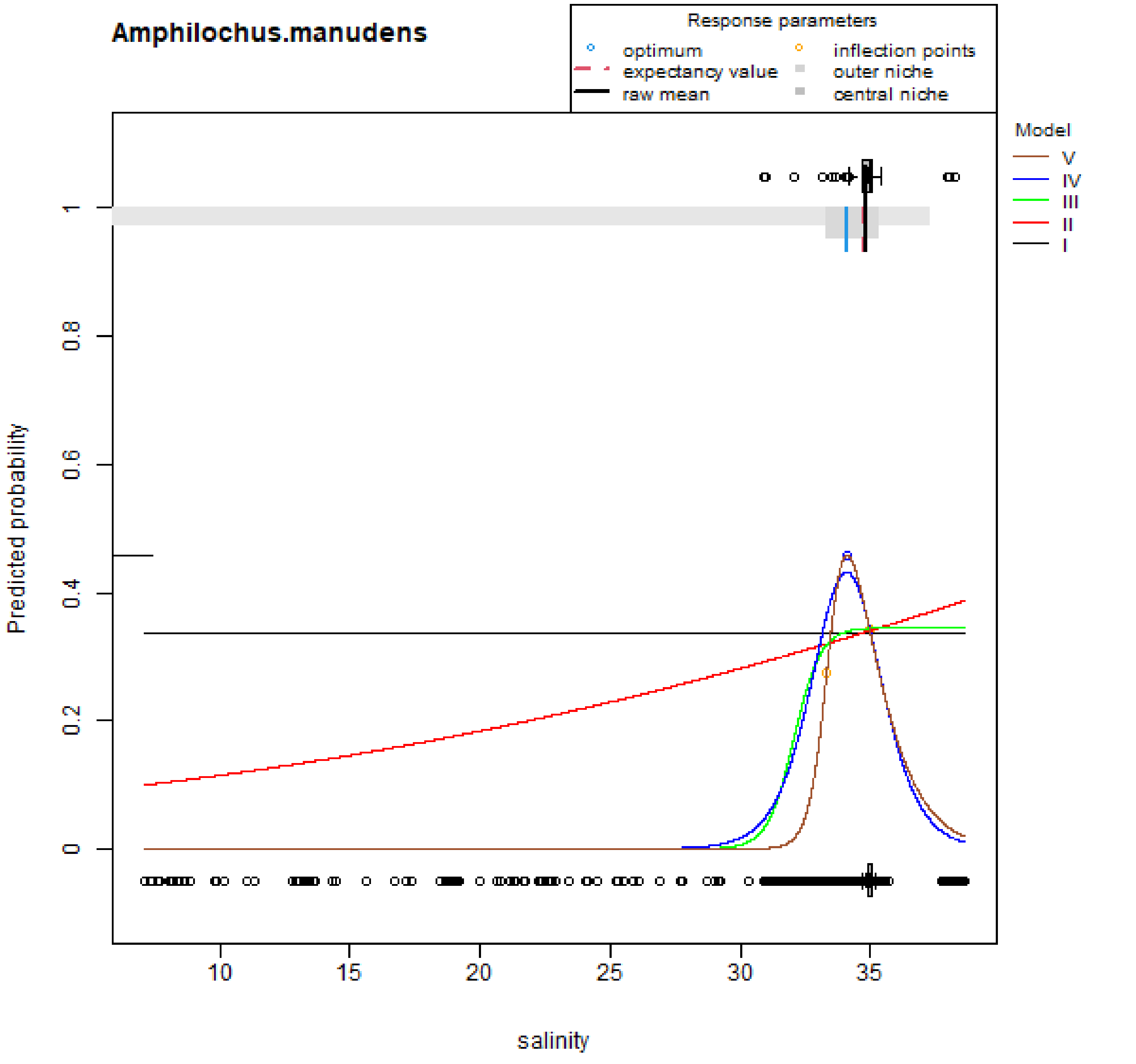
Amphilochus.manudens
Response parameters
optimum
expectancy value
raw mean
inflection points
outer niche
central niche
Model
V
IV
III
II
I
Predicted probability
0
0.2
0.4
0.6
0.8
1
10
15
20
25
30
35
salinity

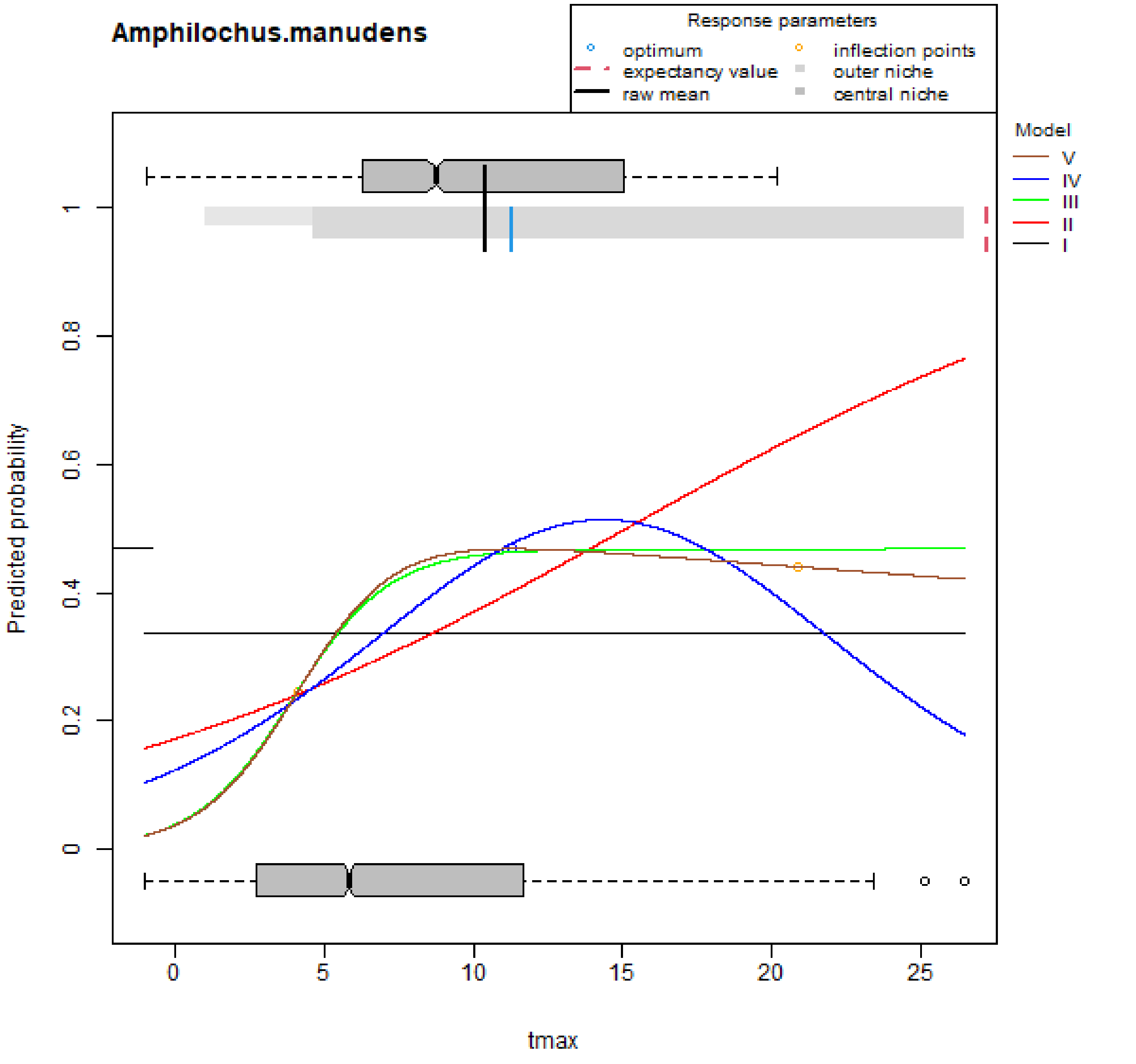

Amphilochus.manudens
Response parameters
optimum
expectancy value
raw mean
inflection points
outer niche
central niche
Model
V
IV
III
II
I
Predicted probability
0
0.2
0.4
0.6
0.8
1
0
5
10
15
20
25
tmax

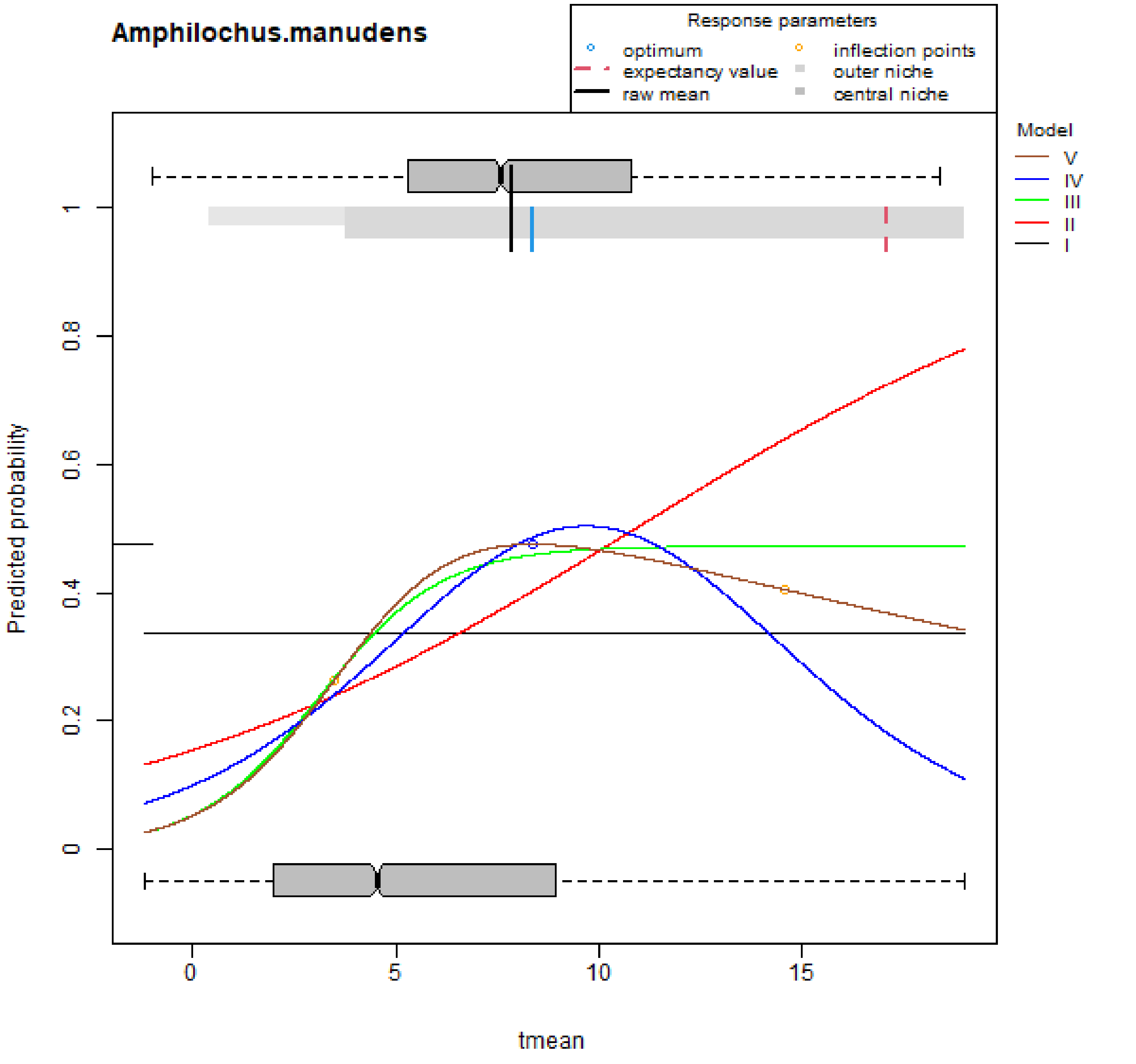

Amphilochus.manudens
Response parameters
optimum
expectancy value
raw mean
inflection points
outer niche
central niche
Model
V
IV
III
II
I
Predicted probability
0
0.2
0.4
0.6
0.8
1
0
5
10
15
tmean

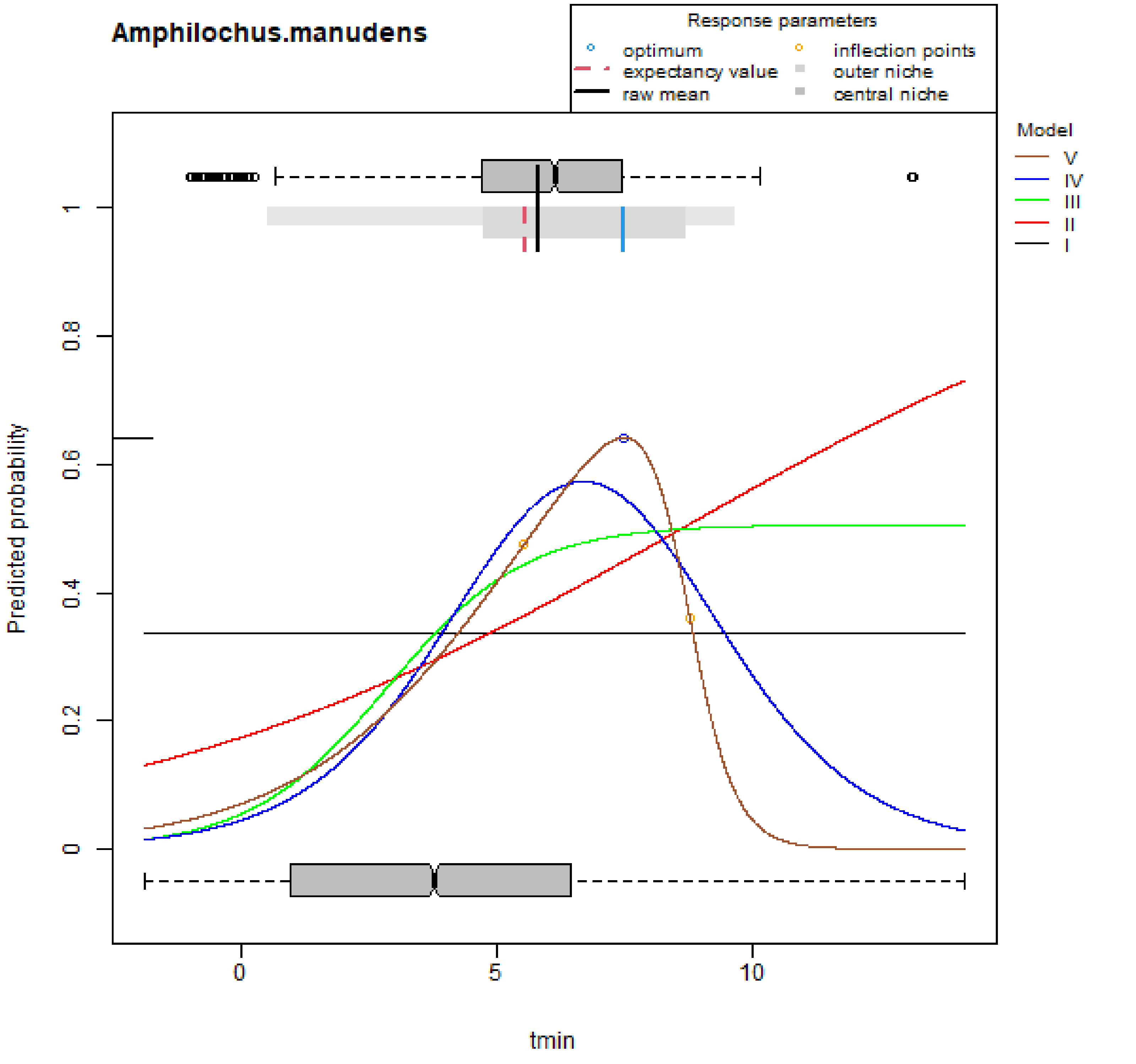

Amphilochus.manudens
Response parameters
optimum
expectancy value
raw mean
inflection points
outer niche
central niche
Model
V
IV
III
II
I
Predicted probability
0
0.2
0.4
0.6
0.8
1
0
5
10
tmin

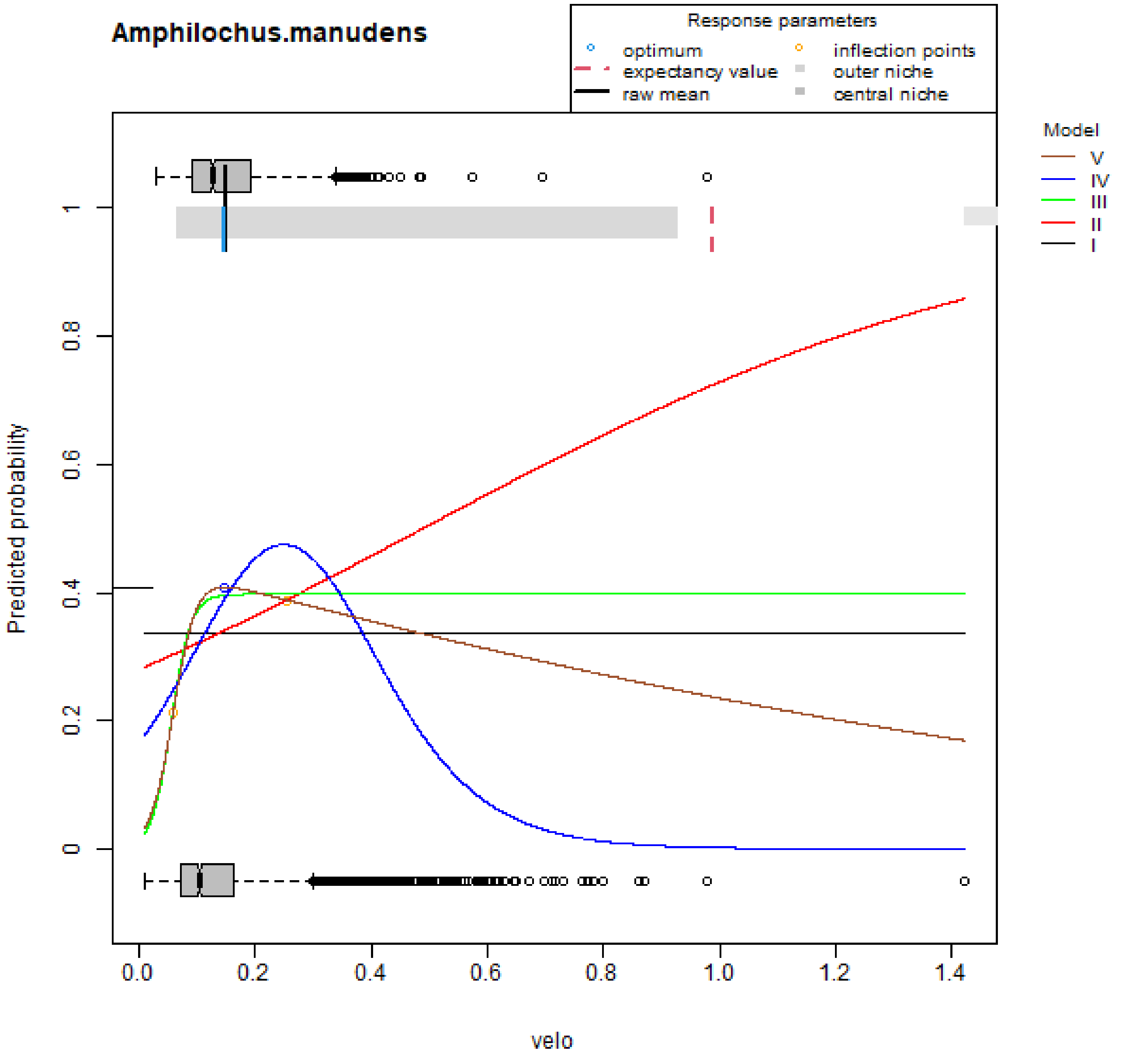
Amphilochus.manudens
Response parameters
optimum
expectancy value
raw mean
inflection points
outer niche
central niche
Model
V
IV
III
II
I
Predicted probability
0
0.2
0.4
0.6
0.8
1
0.0
0.2
0.4
0.6
0.8
1.0
1.2
1.4
velo

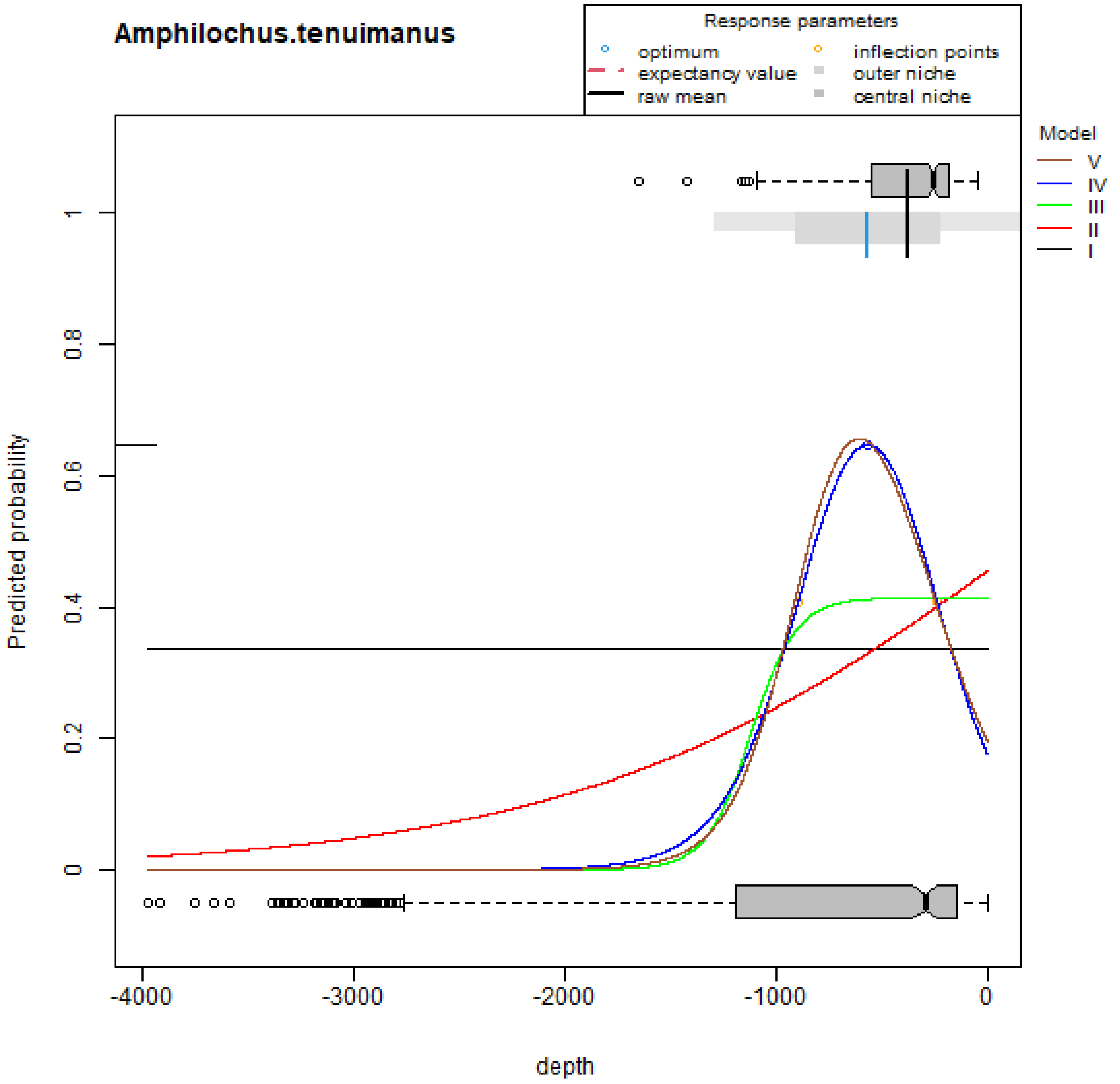

Amphilochus.tenuimanus
Response parameters
optimum
expectancy value
raw mean
inflection points
outer niche
central niche
Model
V
IV
III
II
I
Predicted probability
0
0.2
0.4
0.6
0.8
1
-4000
-3000
-2000
-1000
0
depth

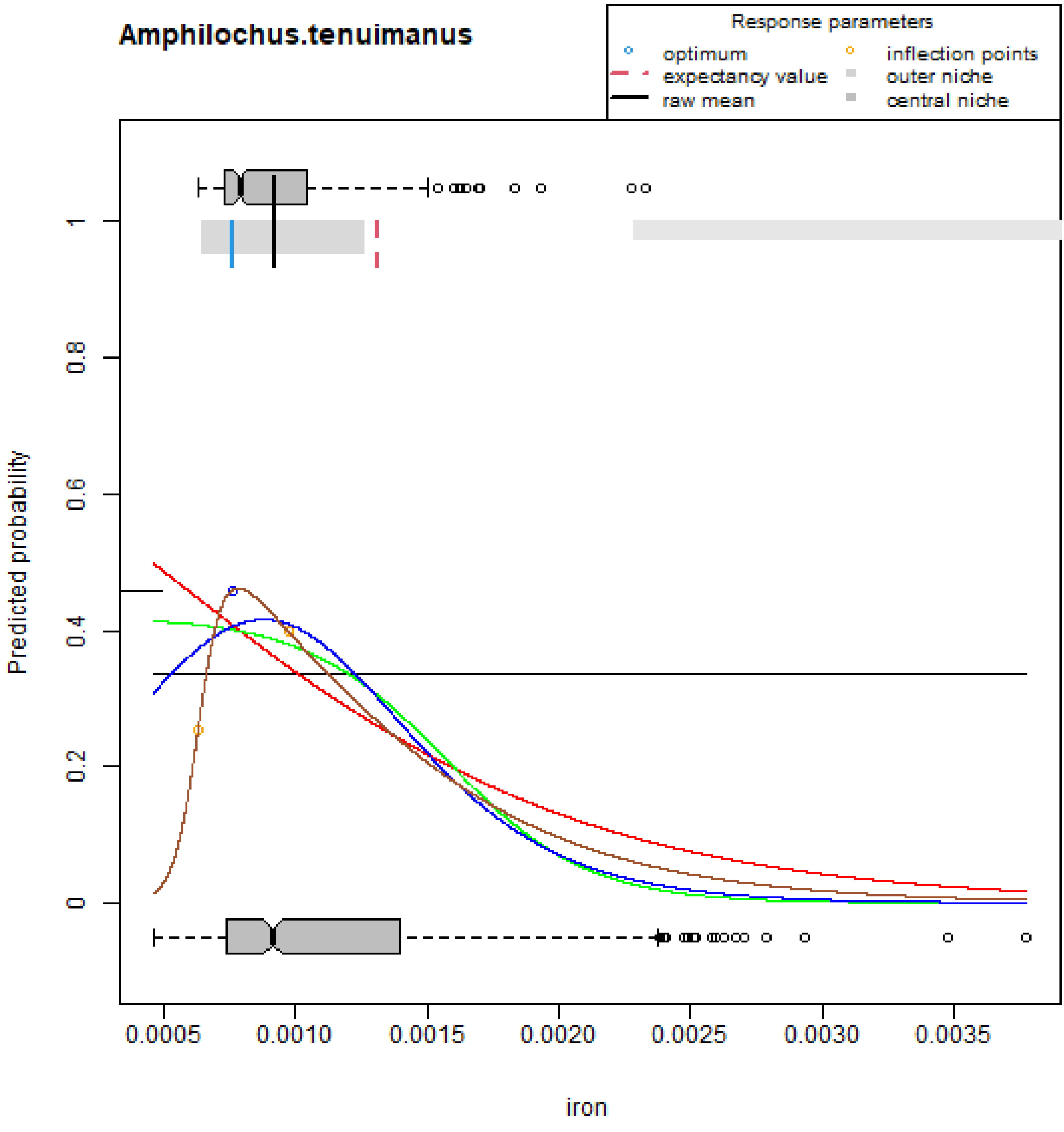

Amphilochus.tenuimanus
Response parameters
optimum
expectancy value
raw mean
inflection points
outer niche
central niche
Predicted probability
0
0.2
0.4
0.6
0.8
1
0.0005
0.0010
0.0015
0.0020
0.0025
0.0030
0.0035
iron

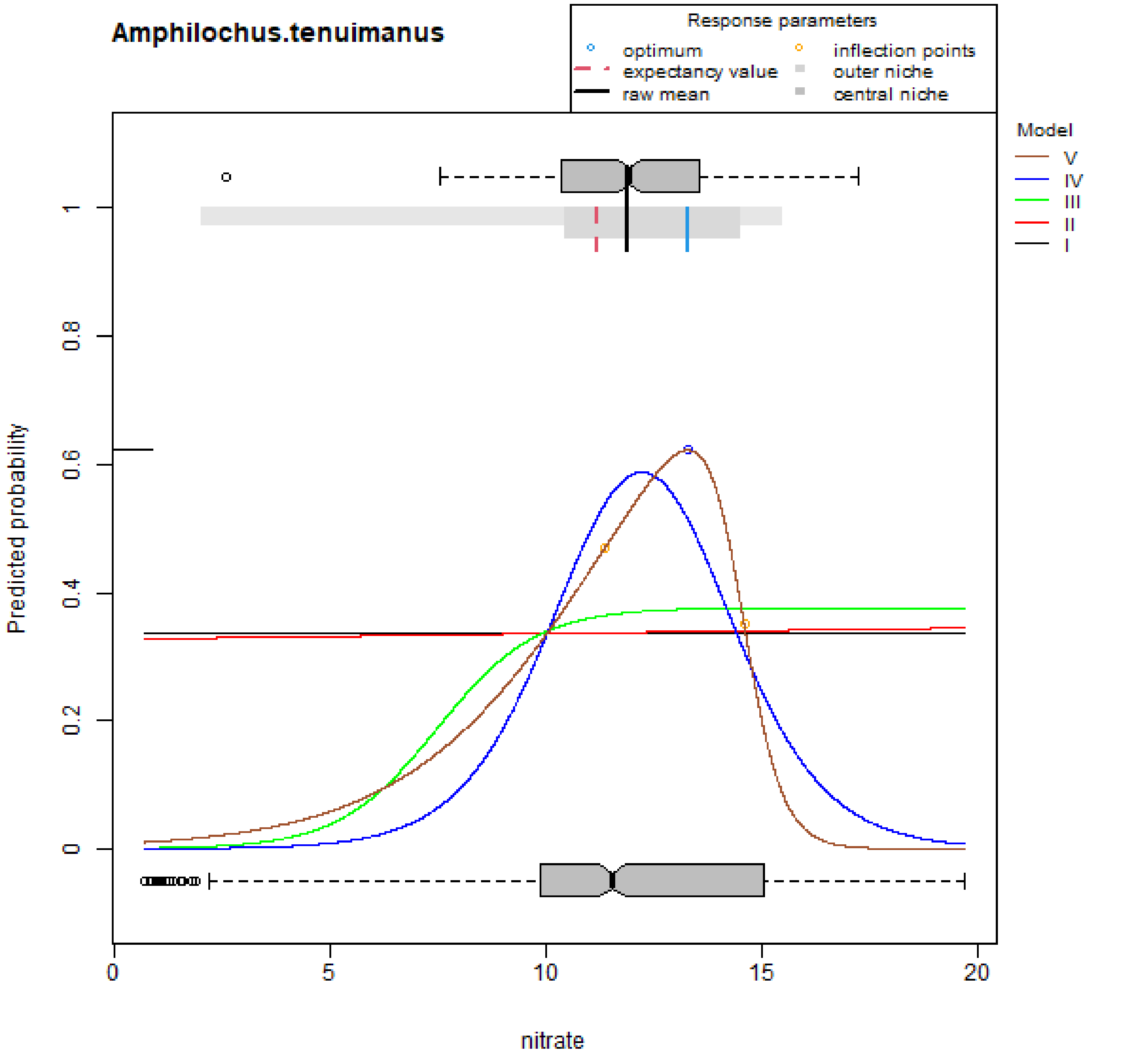
Amphilochus.tenuimanus
Response parameters
optimum
expectancy value
raw mean
inflection points
outer niche
central niche
Model
V
IV
III
II
I
Predicted probability
0
0.2
0.4
0.6
0.8
1
nitrate
0
5
10
15
20

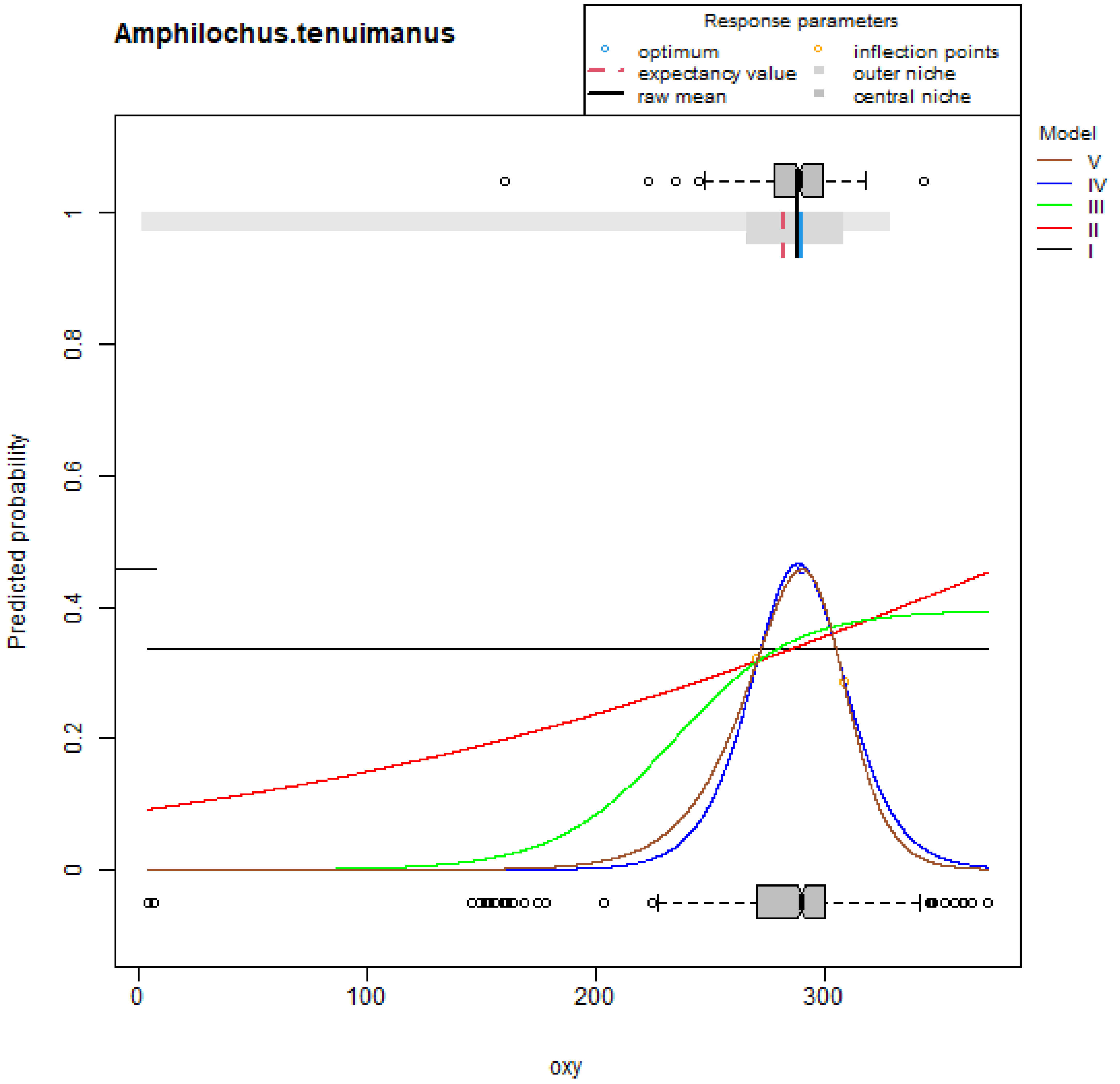
Amphilochus.tenuimanus
Response parameters
optimum
expectancy value
raw mean
inflection points
outer niche
central niche
Model
V
IV
III
II
I
Predicted probability
0
0.2
0.4
0.6
0.8
1
0
100
200
300
oxy

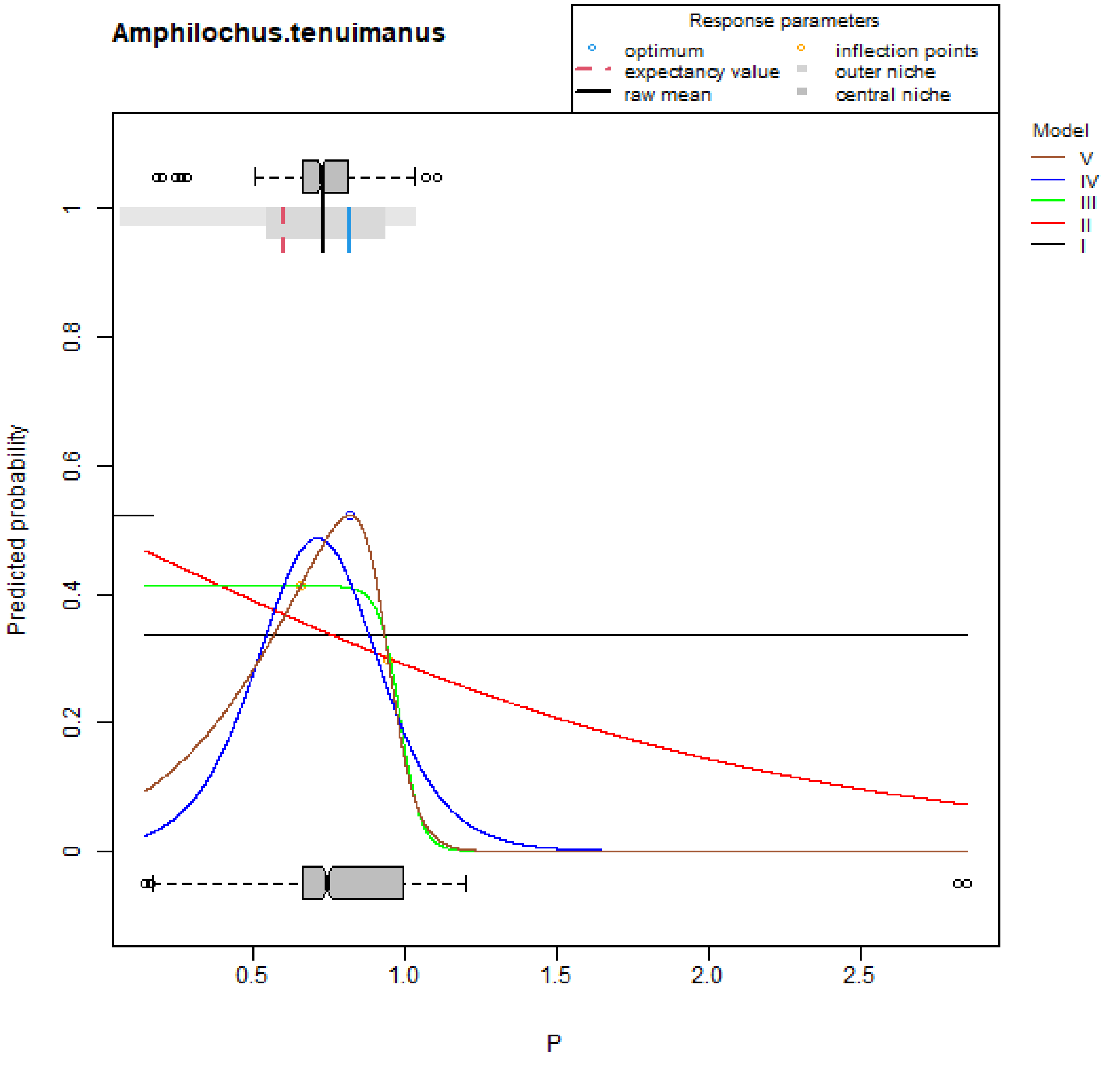
Amphilochus.tenuimanus
Response parameters
optimum
expectancy value
raw mean
inflection points
outer niche
central niche
Model
V
IV
III
II
I
Predicted probability
0
0.2
0.4
0.6
0.8
1
0.5
1.0
1.5
2.0
2.5
P

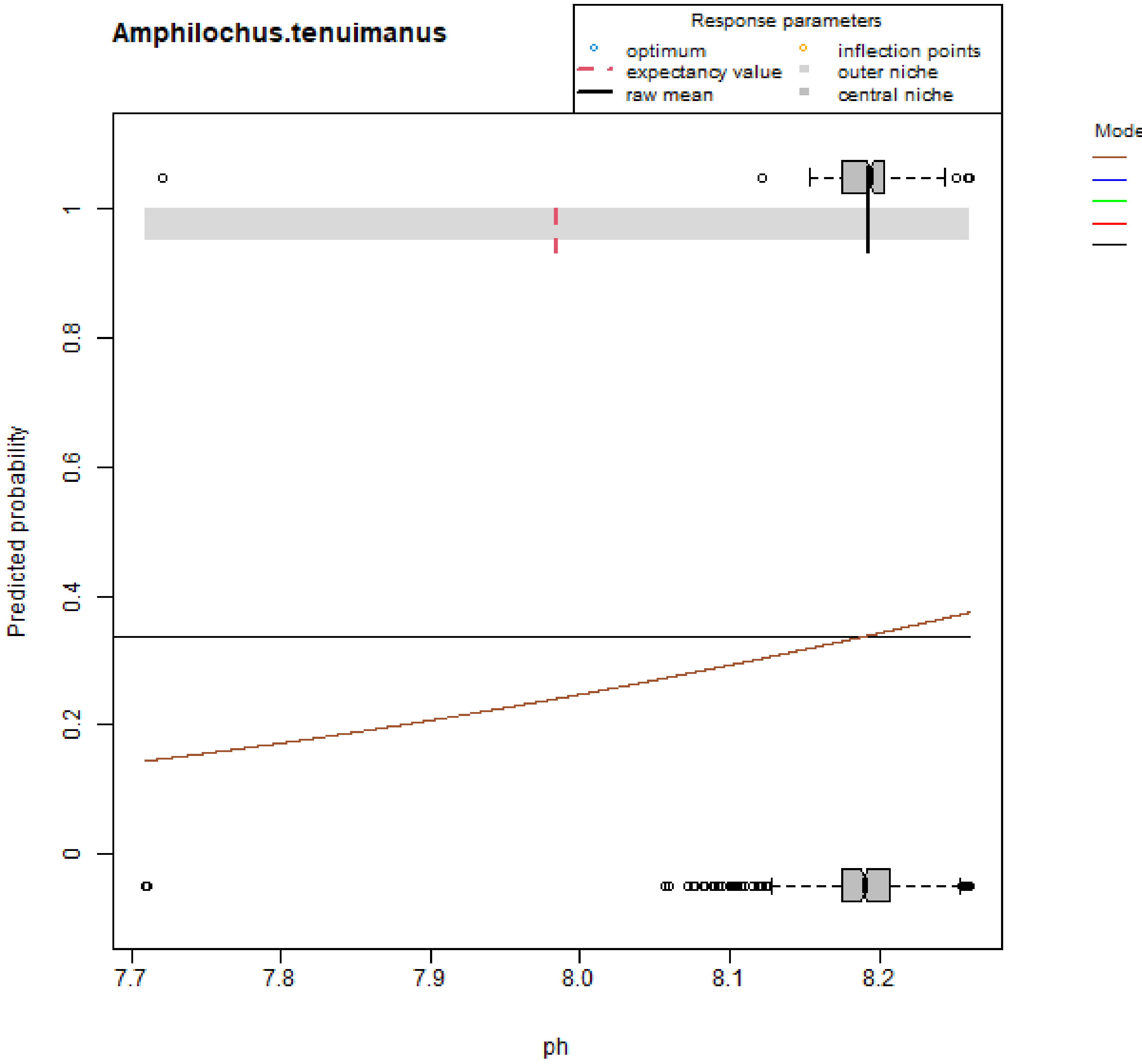
Amphilochus.tenuimanus
Response parameters
optimum
expectancy value
raw mean
inflection points
outer niche
central niche
Predicted probability
0
0.2
0.4
0.6
0.8
1
7.7
7.8
7.9
8.0
8.1
8.2
ph

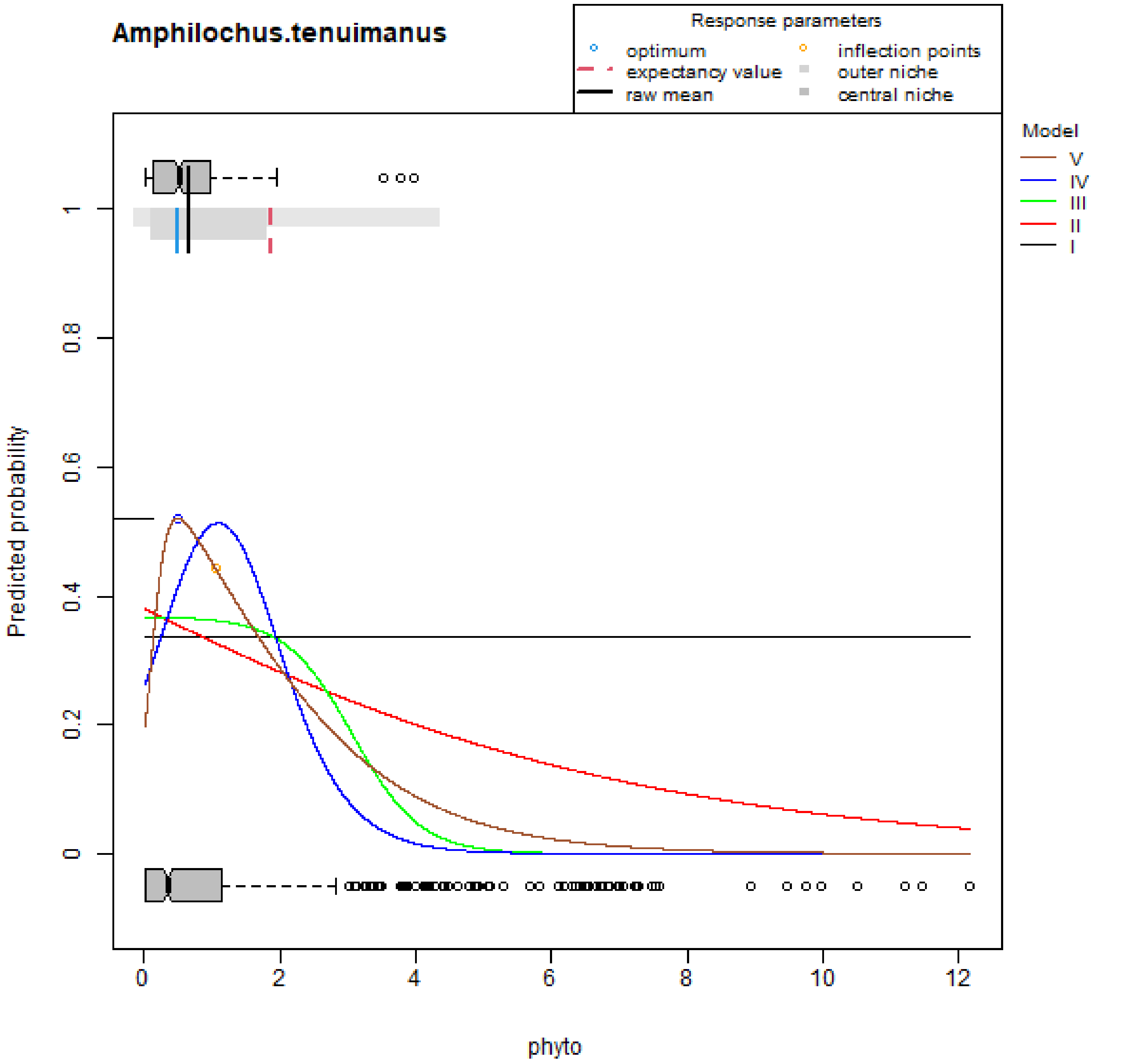
Amphilochus.tenuimanus
Response parameters
optimum
expectancy value
raw mean
inflection points
outer niche
central niche
Model
V
IV
III
II
I
Predicted probability
0
0.2
0.4
0.6
0.8
1
0
2
4
6
8
10
12
phyto

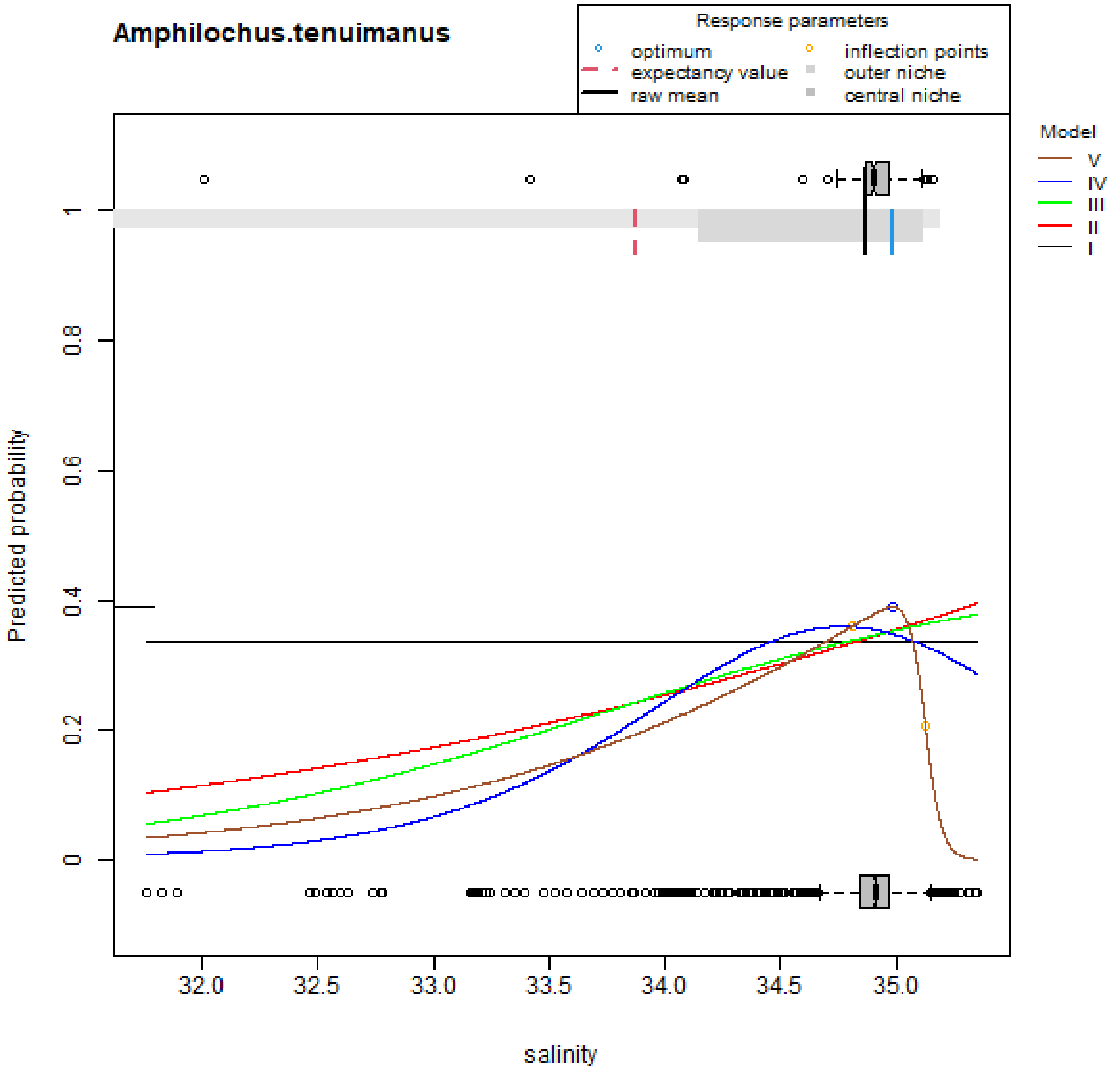

Amphilochus.tenuimanus
Response parameters
optimum
expectancy value
raw mean
inflection points
outer niche
central niche
Model
V
IV
III
II
I
Predicted probability
0
0.2
0.4
0.6
0.8
1
32.0
32.5
33.0
33.5
34.0
34.5
35.0
salinity

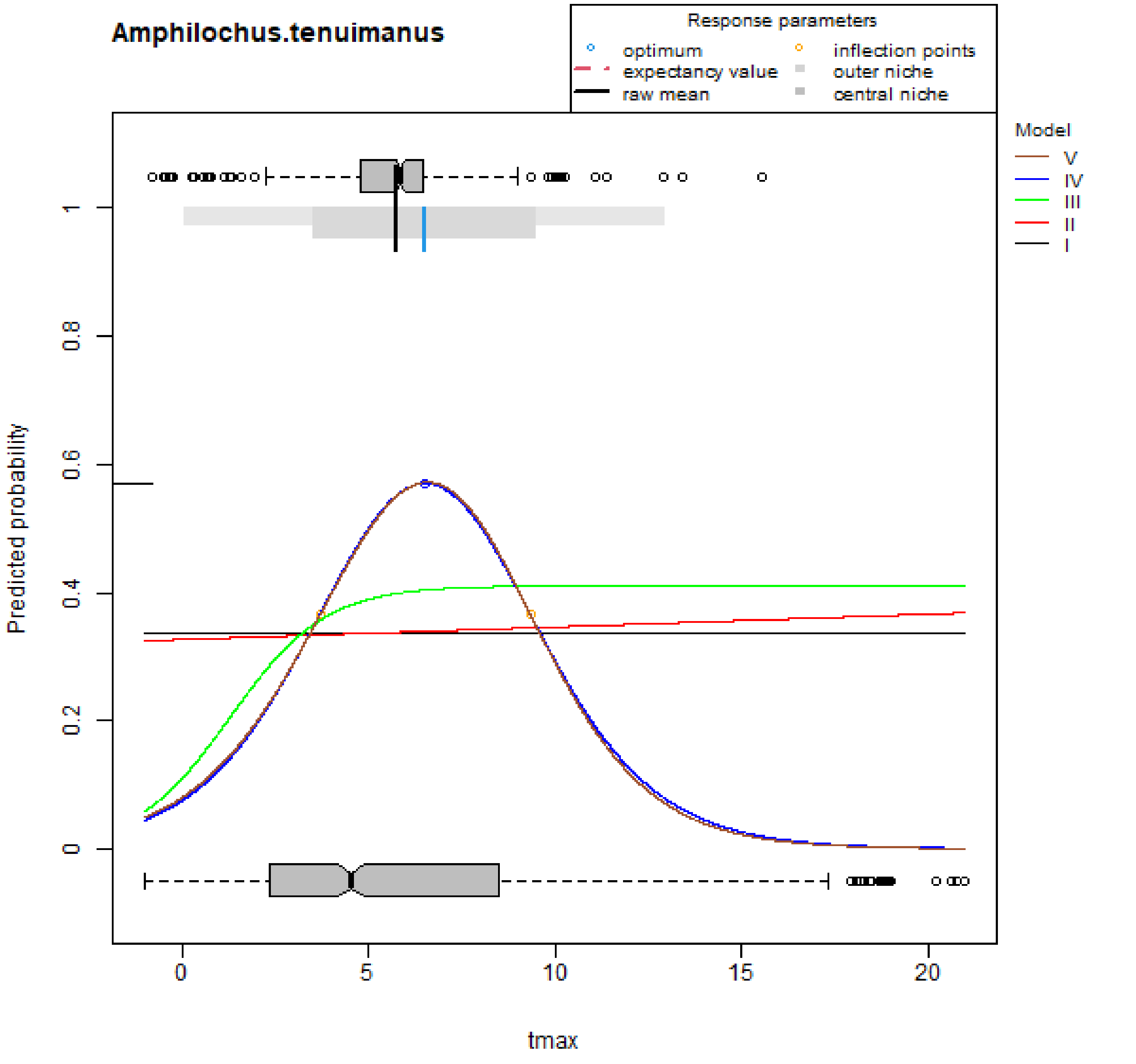

Amphilochus.tenuimanus
Response parameters
optimum
expectancy value
raw mean
inflection points
outer niche
central niche
Model
V
IV
III
II
I
Predicted probability
0
0.2
0.4
0.6
0.8
1
0
5
10
15
20
tmax

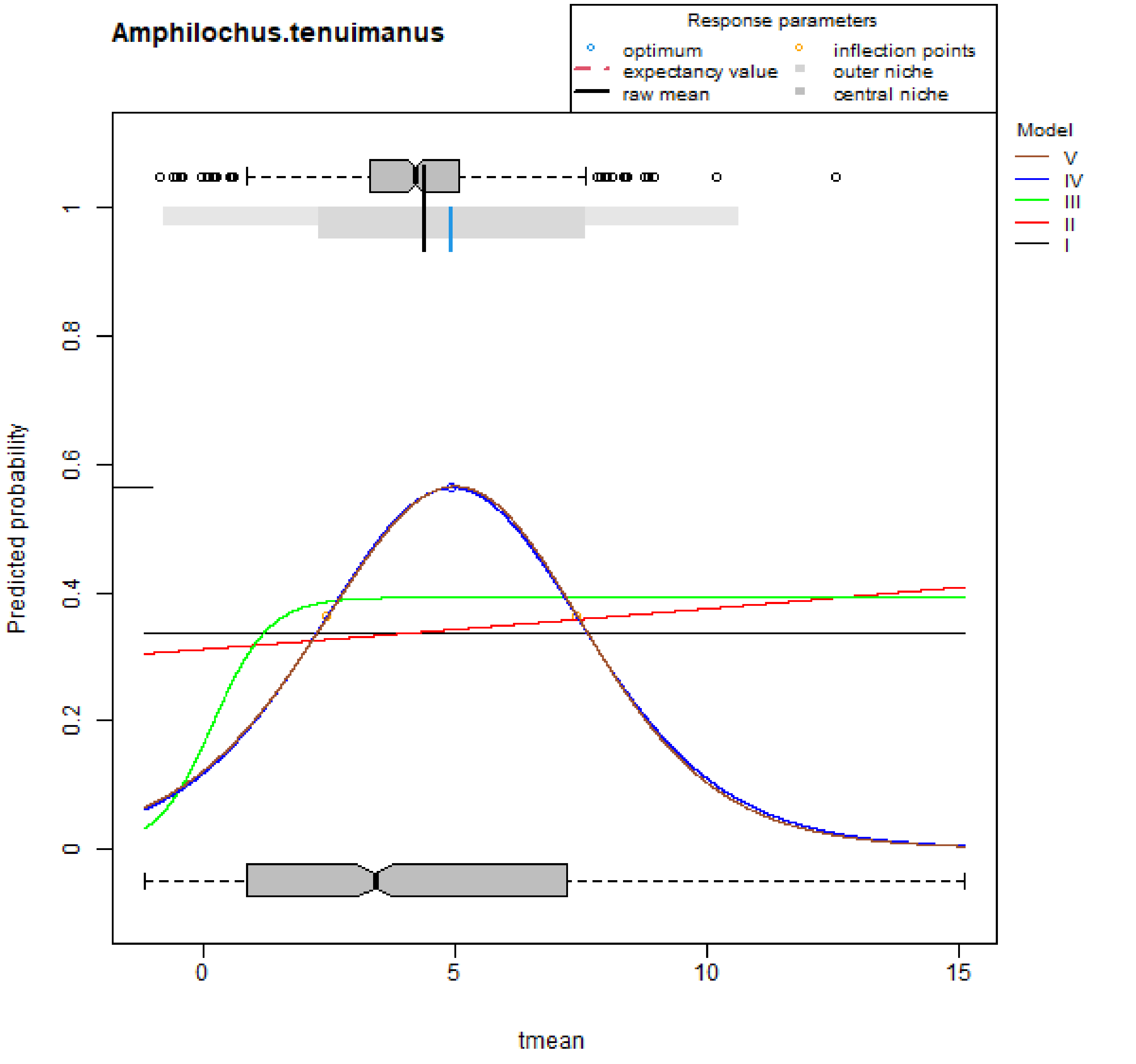

Amphilochus.tenuimanus
Response parameters
optimum
expectancy value
raw mean
inflection points
outer niche
central niche
Model
V
IV
III
II
I
Predicted probability
0
0.2
0.4
0.6
0.8
1
0
5
10
15
tmean

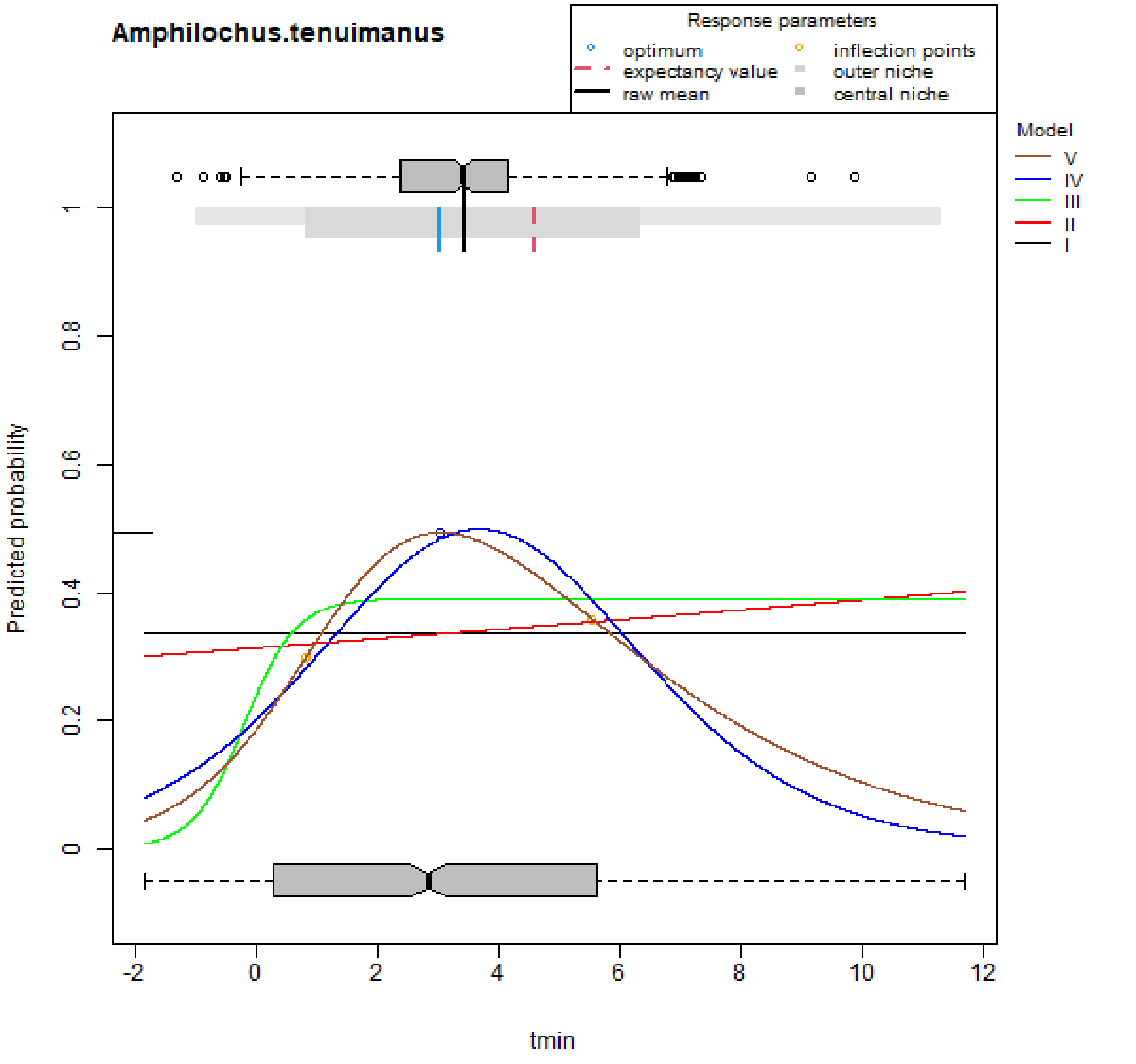

Amphilochus.tenuimanus
Response parameters
optimum
expectancy value
raw mean
inflection points
outer niche
central niche
Model
V
IV
III
II
I
Predicted probability
0
0.2
0.4
0.6
0.8
1
-2
0
2
4
6
8
10
12
tmin

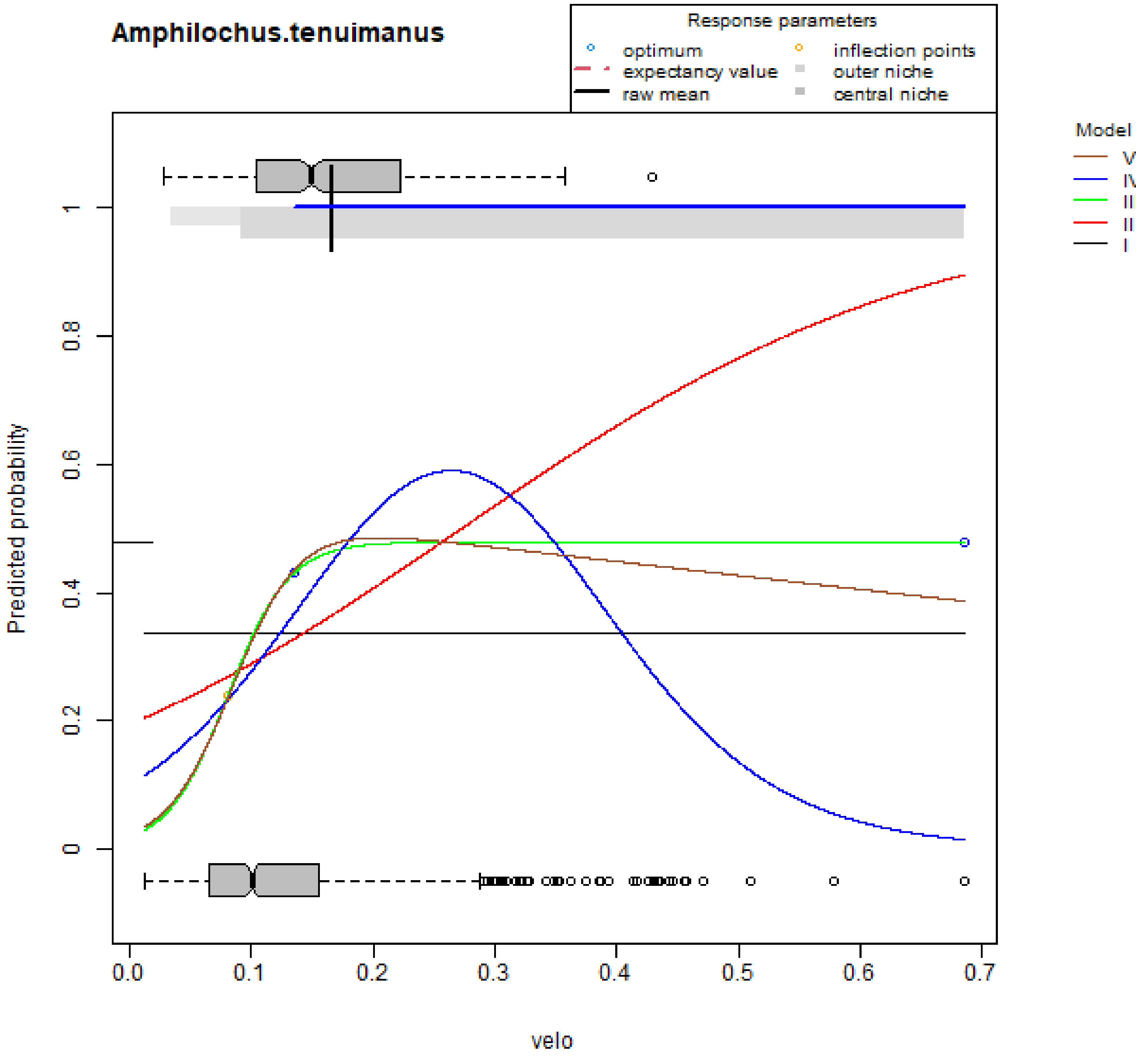
Amphilochus.tenuimanus
Response parameters
optimum
expectancy value
raw mean
inflection points
outer niche
central niche
Model
Predicted probability
0
0.2
0.4
0.6
0.8
1
0.0
0.1
0.2
0.3
0.4
0.5
0.6
0.7
velo

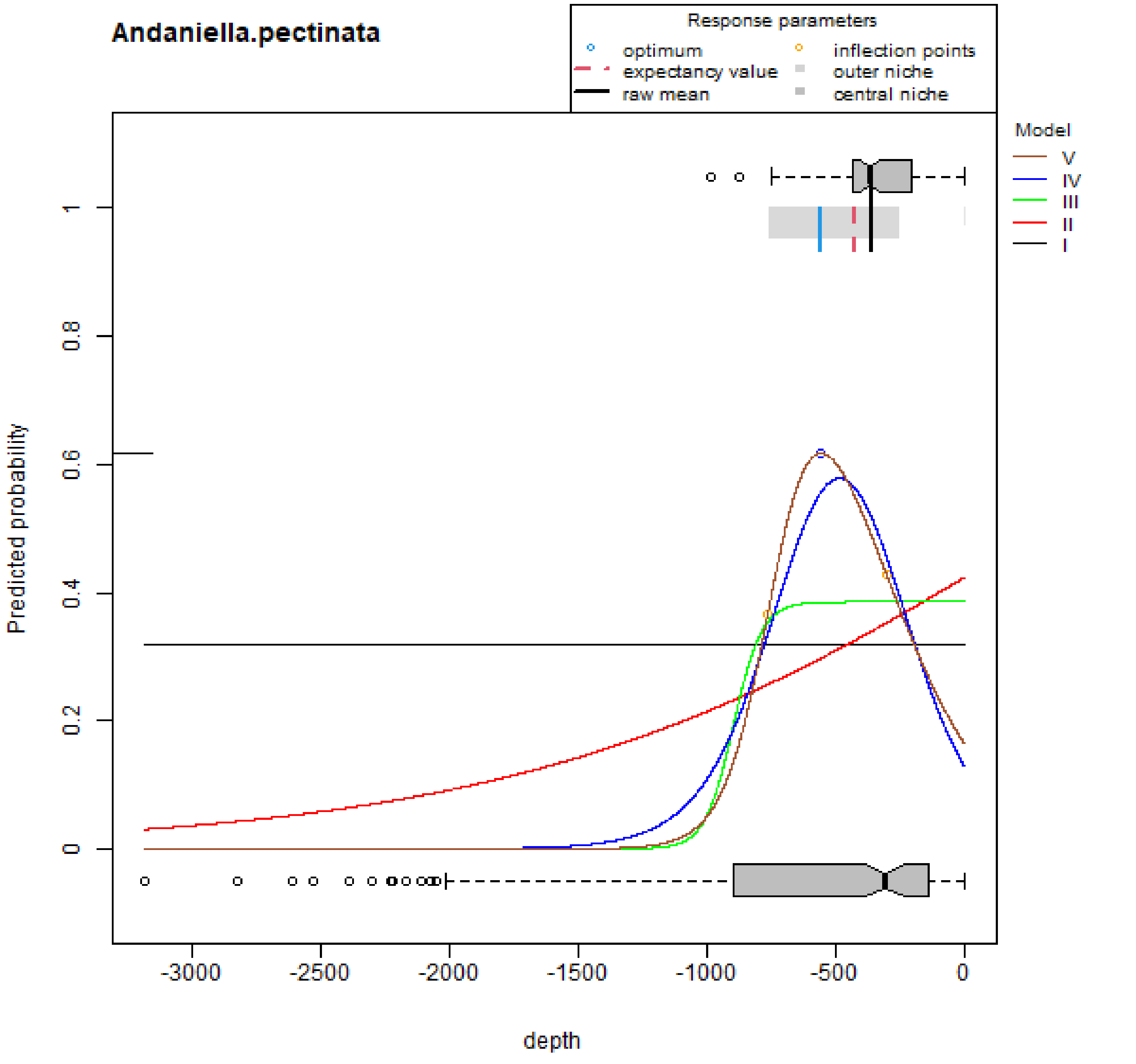

Andaniella.pectinata
Response parameters
optimum
expectancy value
raw mean
inflection points
outer niche
central niche
Model
V
IV
III
II
I
Predicted probability
0
0.2
0.4
0.6
0.8
1
-3000
-2500
-2000
-1500
-1000
-500
0
depth

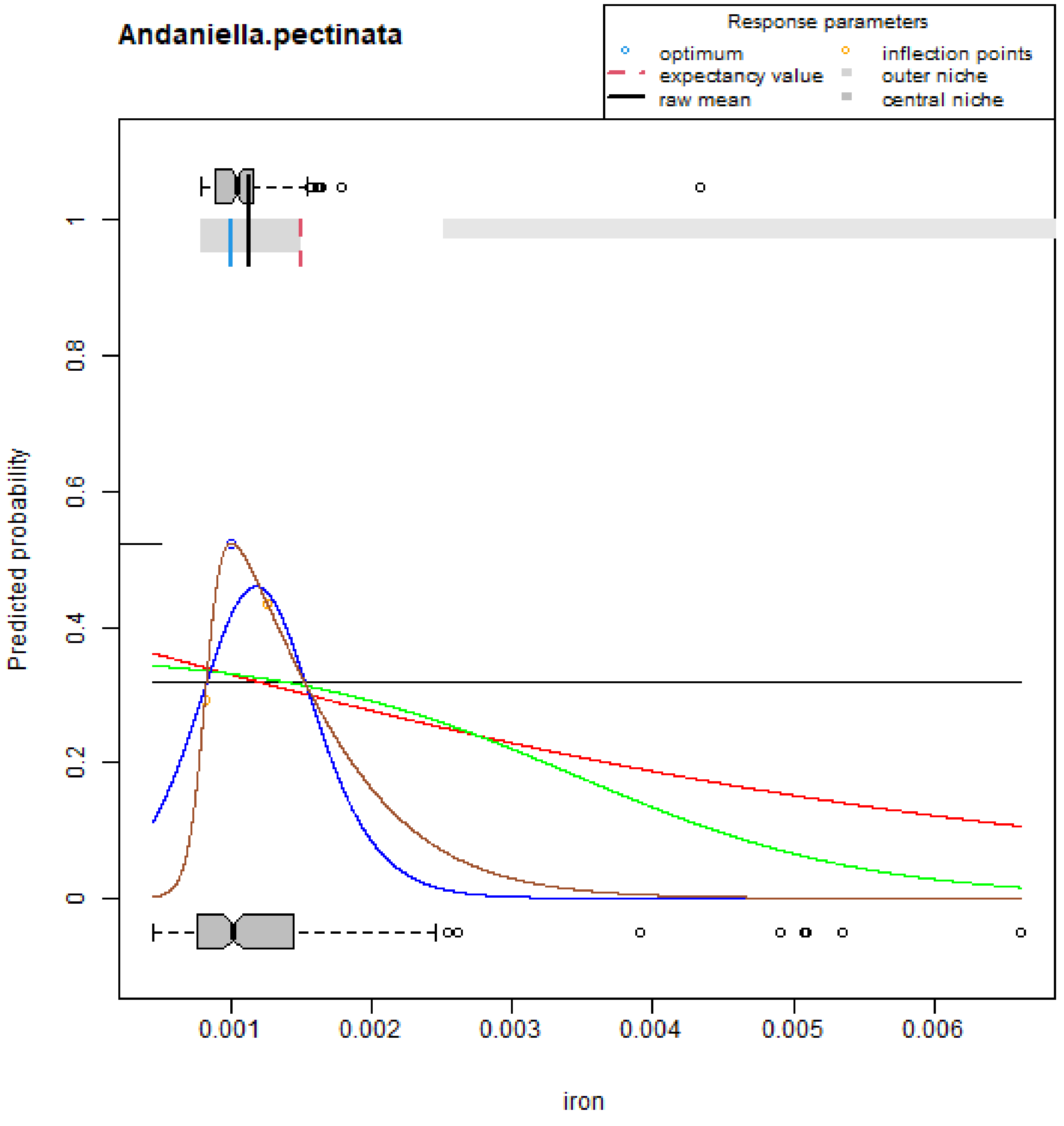

Andaniella.pectinata
Response parameters
optimum
expectancy value
raw mean
inflection points
outer niche
central niche
Predicted probability
0
0.2
0.4
0.6
0.8
1
0.001
0.002
0.003
0.004
0.005
0.006
iron

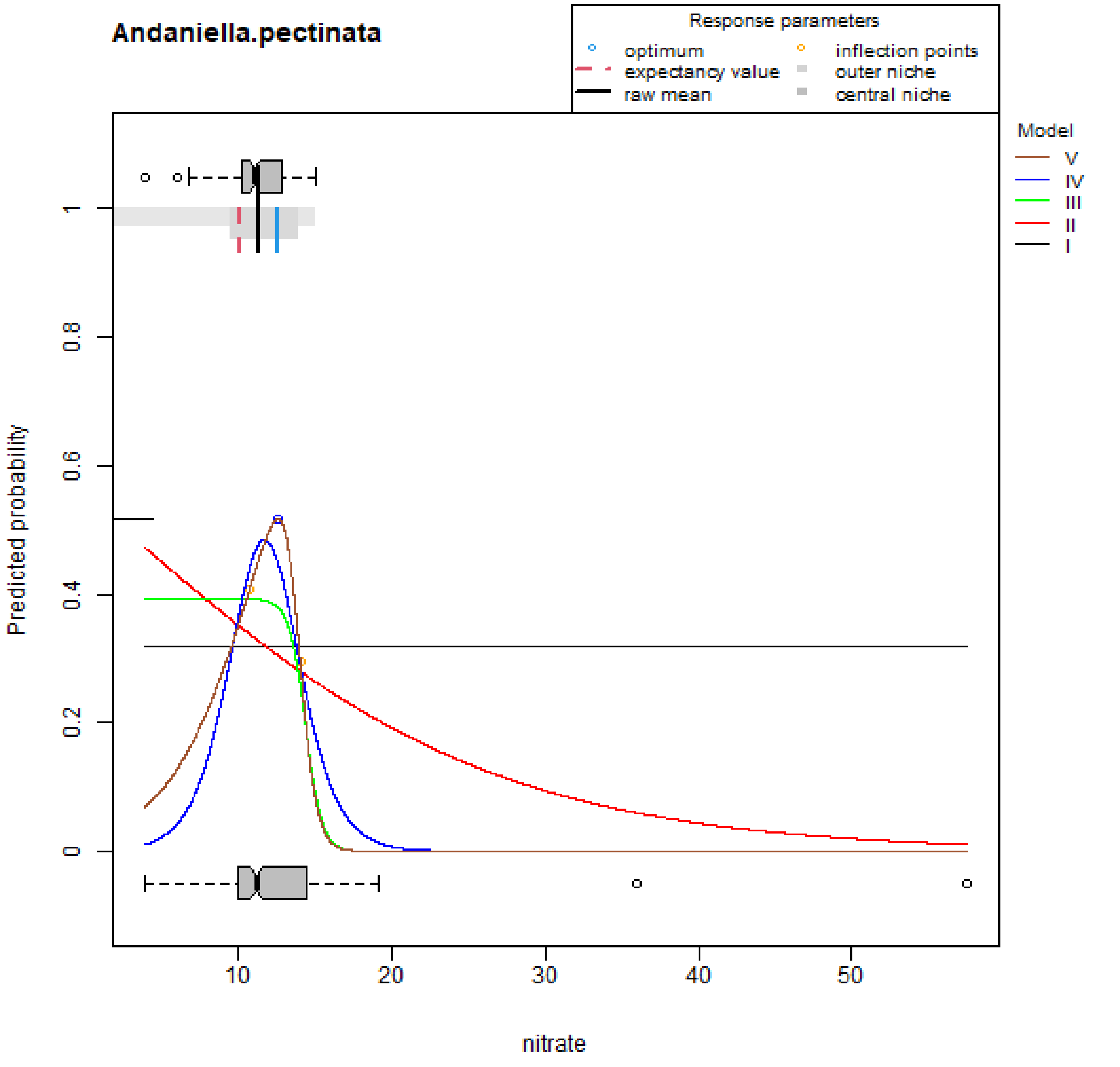
Andaniella.pectinata
Response parameters
optimum
expectancy value
raw mean
inflection points
outer niche
central niche
Model
V
IV
III
II
I
Predicted probability
0
0.2
0.4
0.6
0.8
1
10
20
30
40
50
nitrate

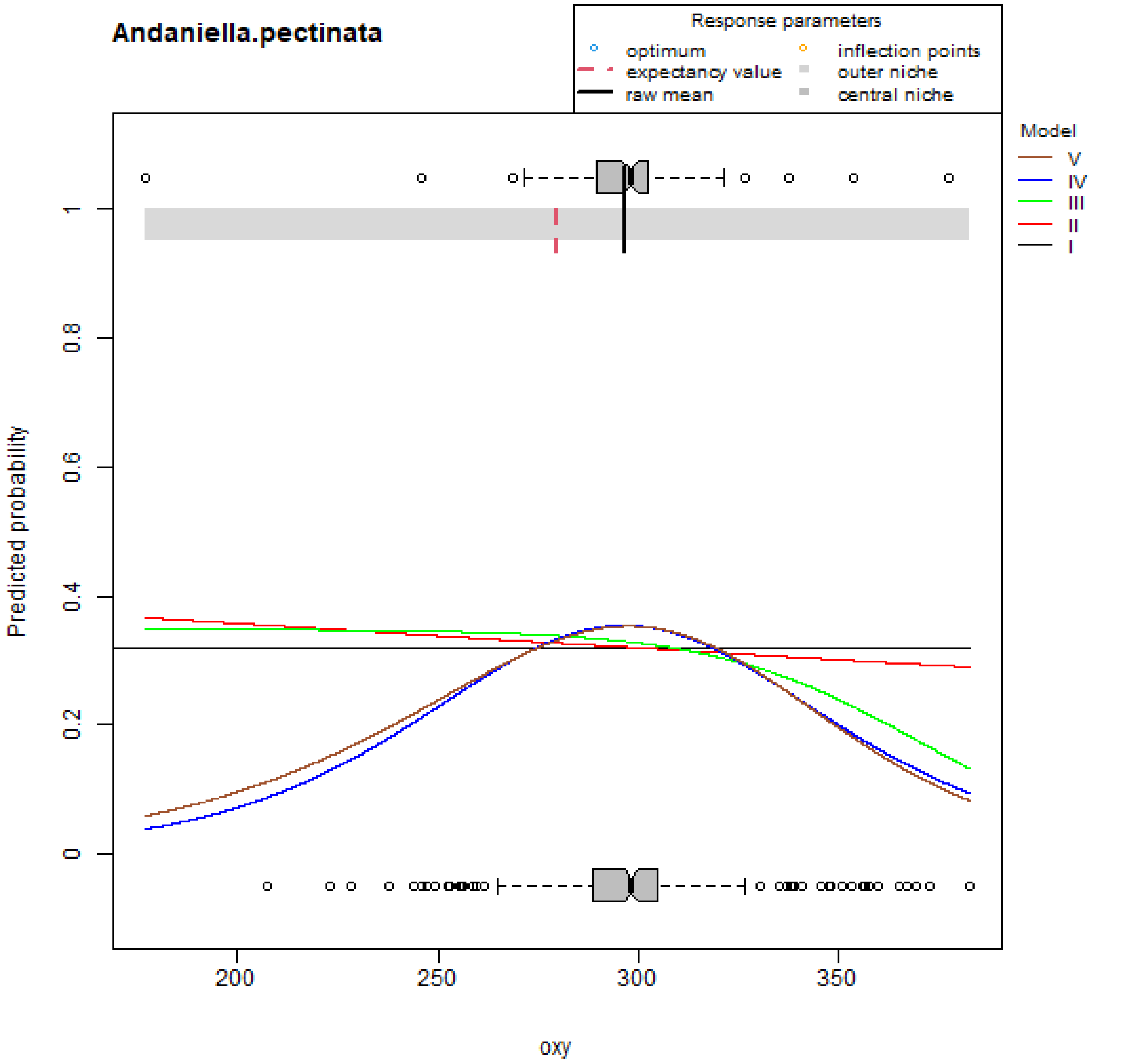
Andaniella.pectinata
Response parameters
optimum
expectancy value
raw mean
inflection points
outer niche
central niche
Model
V
IV
III
II
I
Predicted probability
0
0.2
0.4
0.6
0.8
1
200
250
300
350
oxy

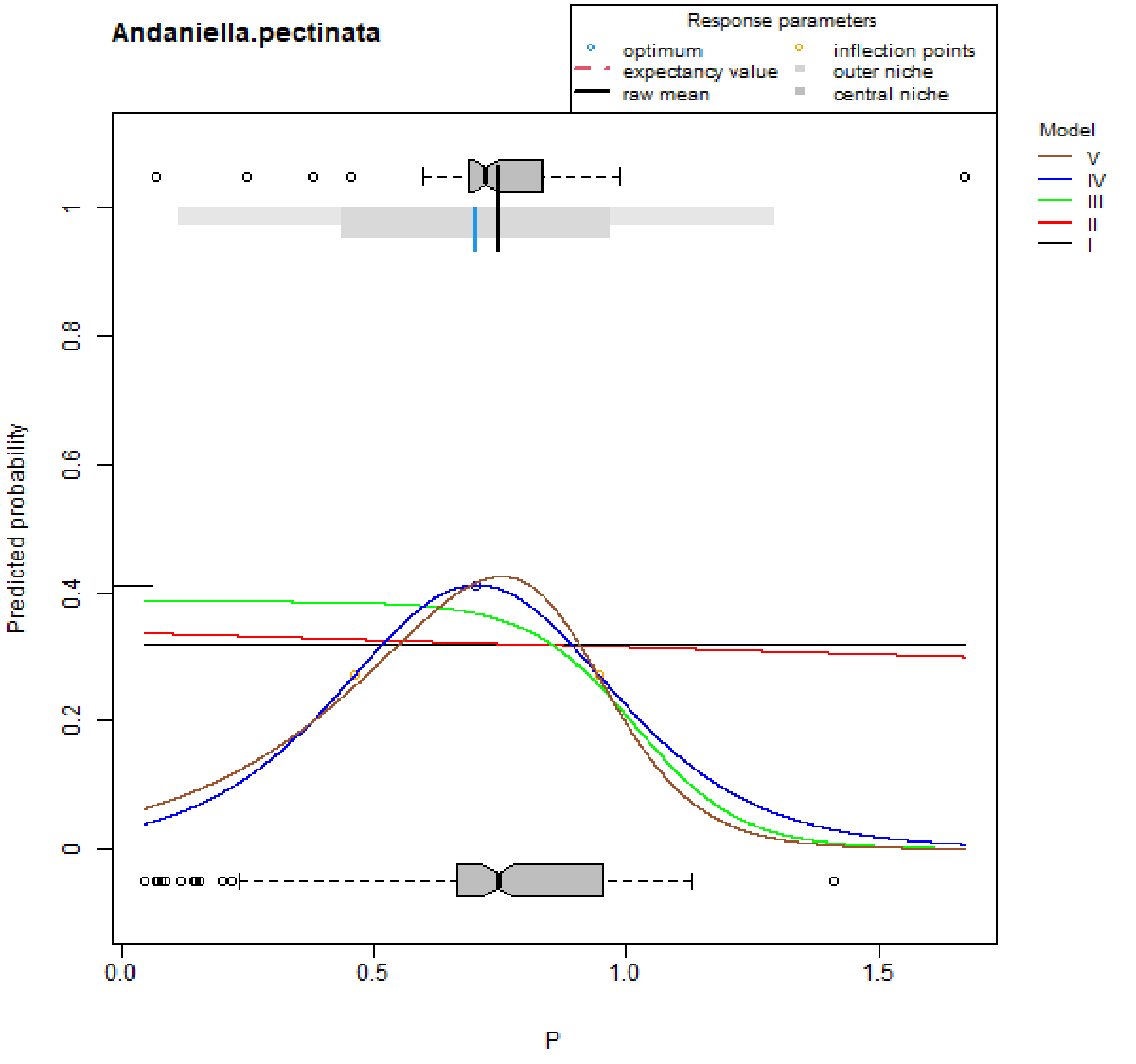

Andaniella.pectinata
Response parameters
optimum
expectancy value
raw mean
inflection points
outer niche
central niche
Model
V
IV
III
II
I
Predicted probability
0
0.2
0.4
0.6
0.8
1
0.0
0.5
1.0
1.5
P

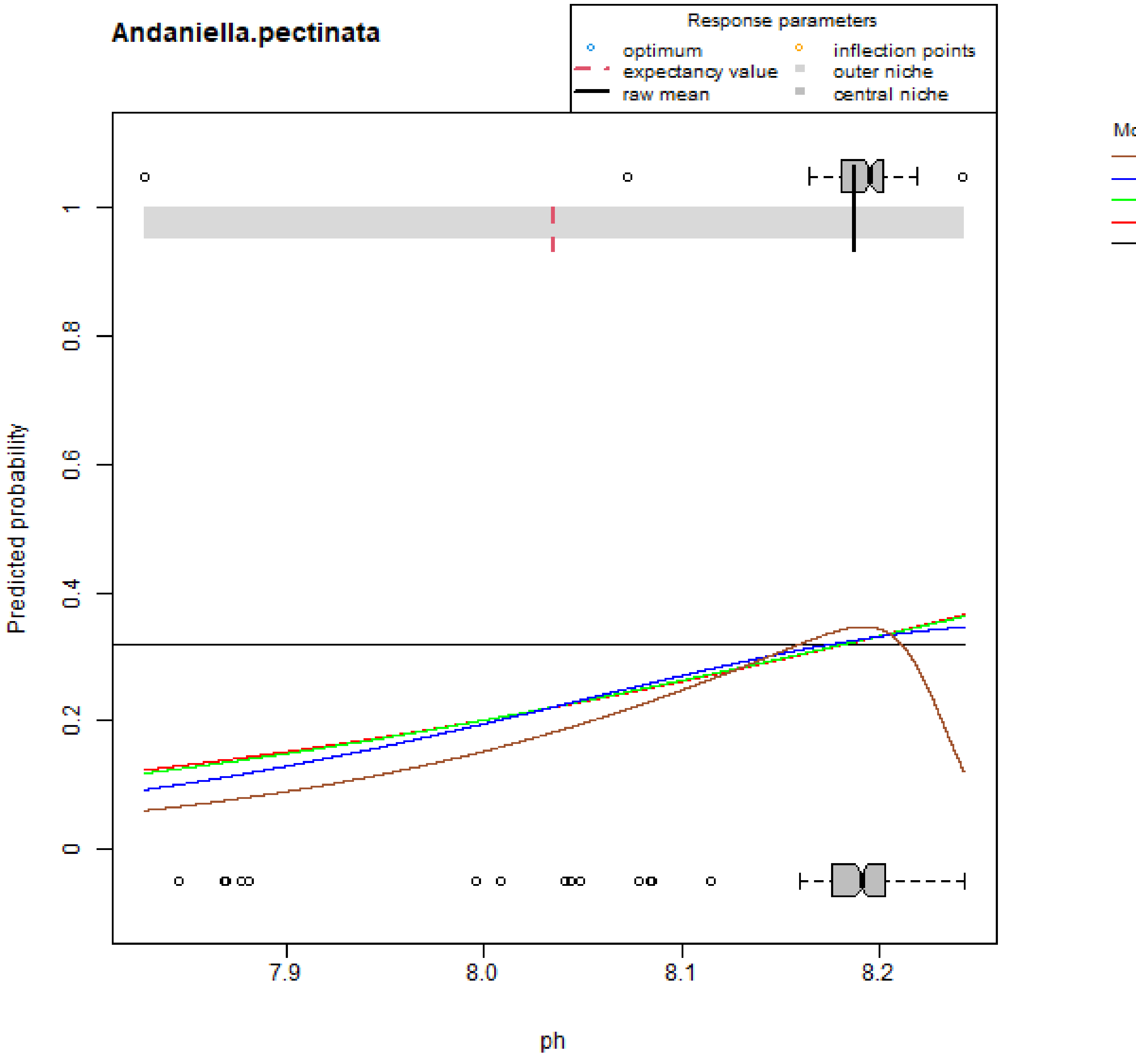

Andaniella.pectinata
Response parameters
optimum
expectancy value
raw mean
inflection points
outer niche
central niche
Predicted probability
0
0.2
0.4
0.6
0.8
1
7.9
8.0
8.1
8.2
ph

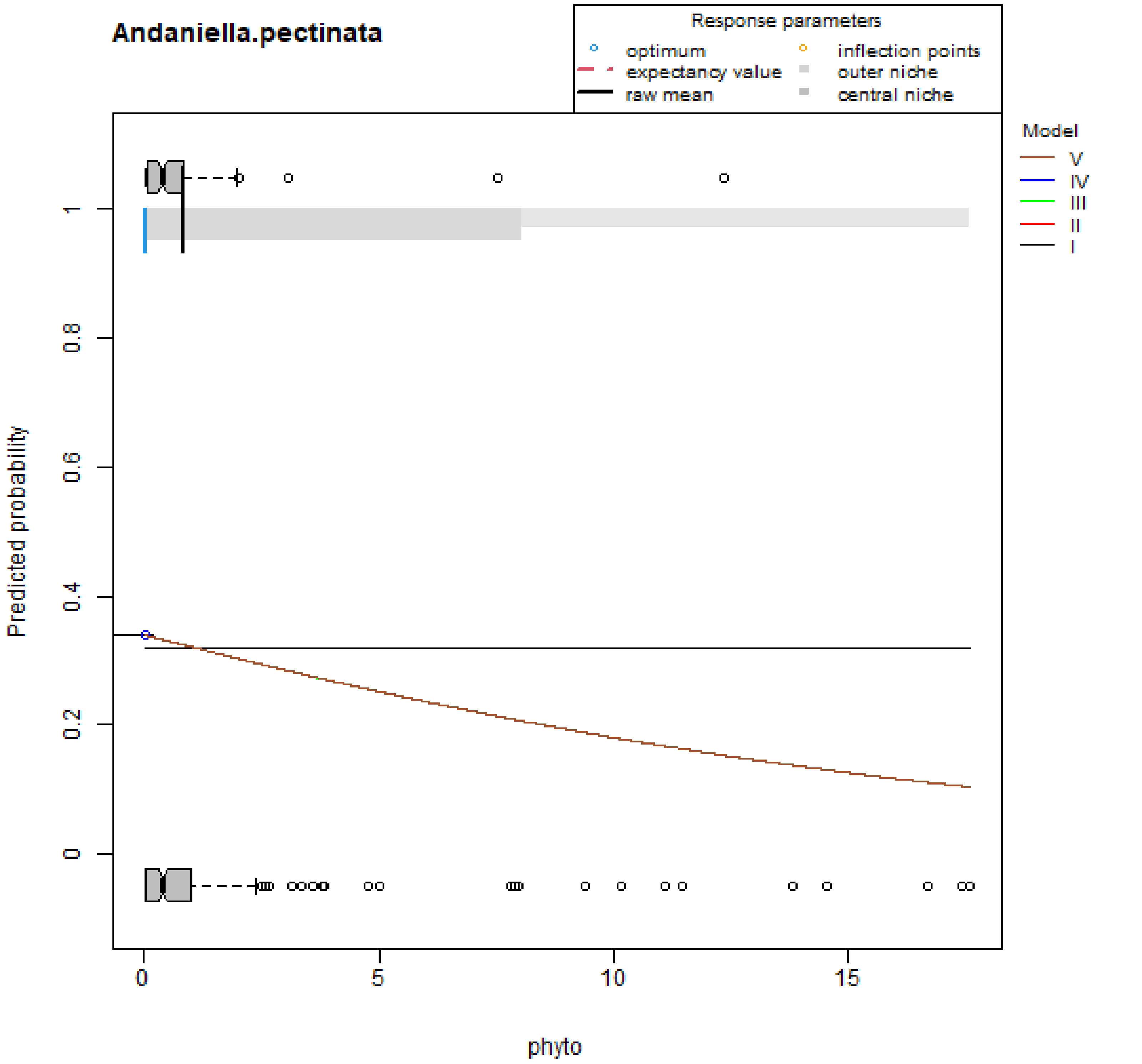

Andaniella.pectinata
Response parameters
optimum
expectancy value
raw mean
inflection points
outer niche
central niche
Model
V
IV
III
II
I
Predicted probability
0
0.2
0.4
0.6
0.8
1
0
5
10
15
phyto

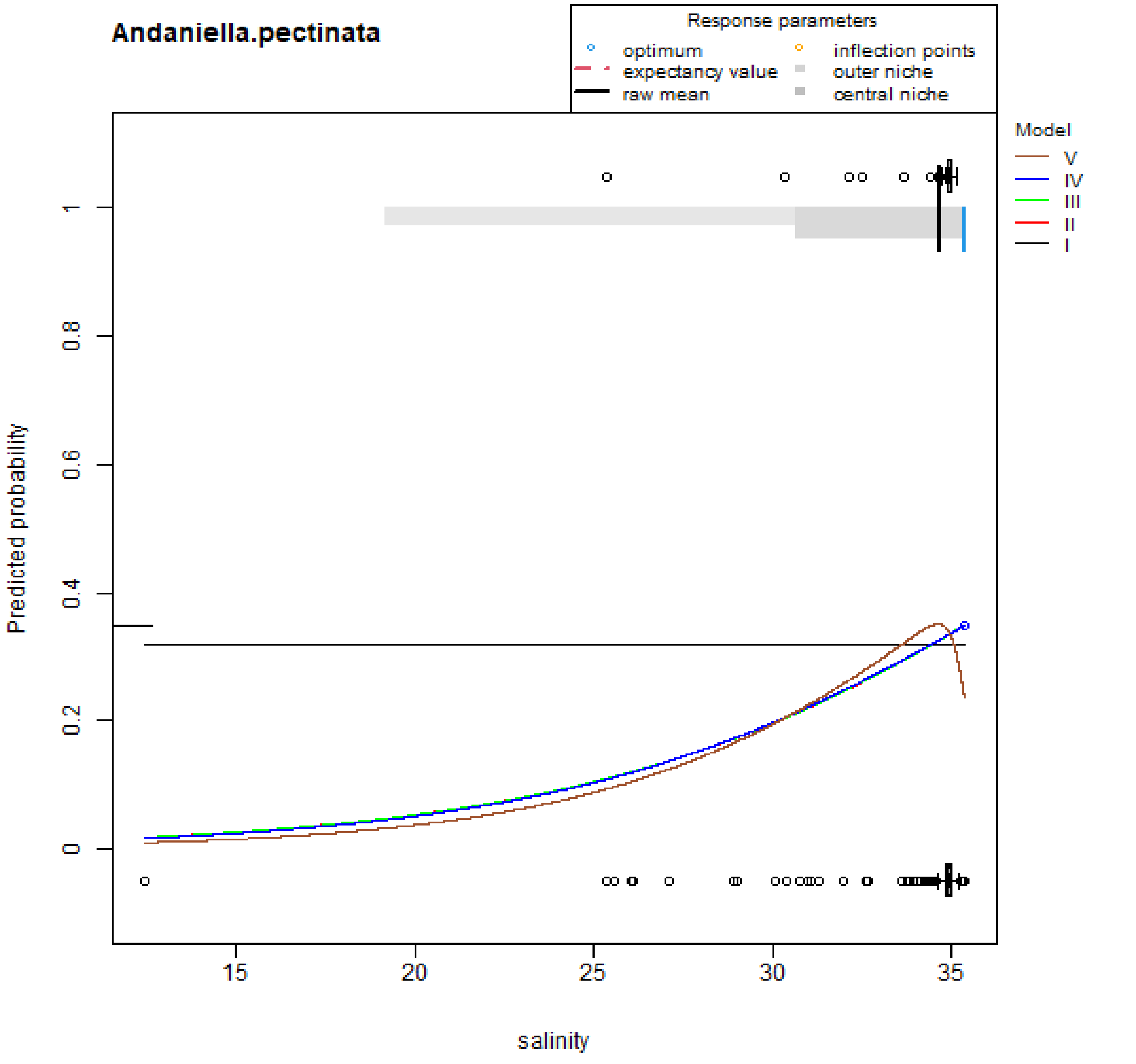

Andaniella.pectinata
Response parameters
optimum
expectancy value
raw mean
inflection points
outer niche
central niche
Model
V
IV
III
II
I
Predicted probability
0
0.2
0.4
0.6
0.8
1
15
20
25
30
35
salinity

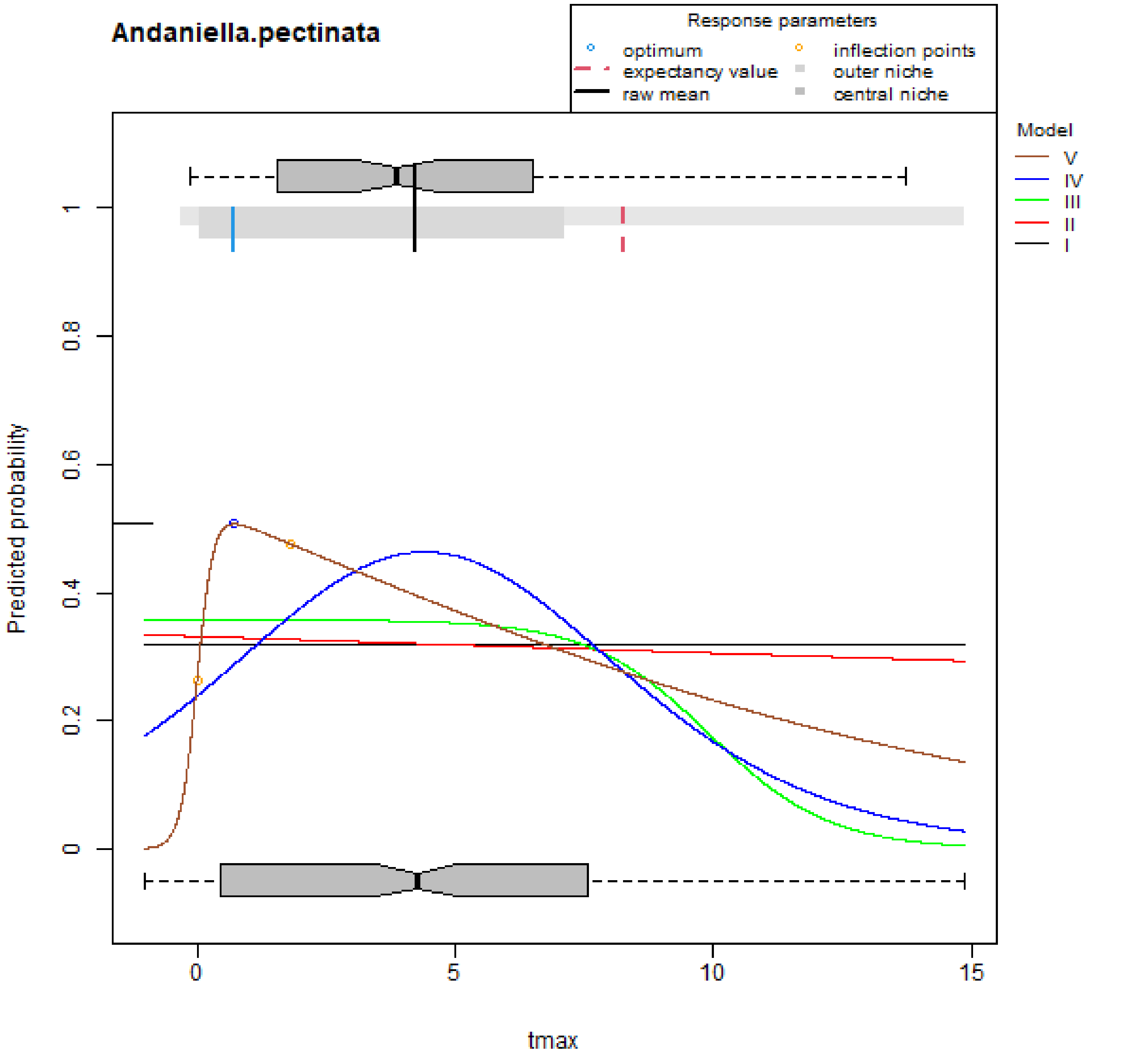
Andaniella.pectinata
Response parameters
optimum
expectancy value
raw mean
inflection points
outer niche
central niche
Model
V
IV
III
II
I
Predicted probability
0
0.2
0.4
0.6
0.8
1
0
5
10
15
tmax

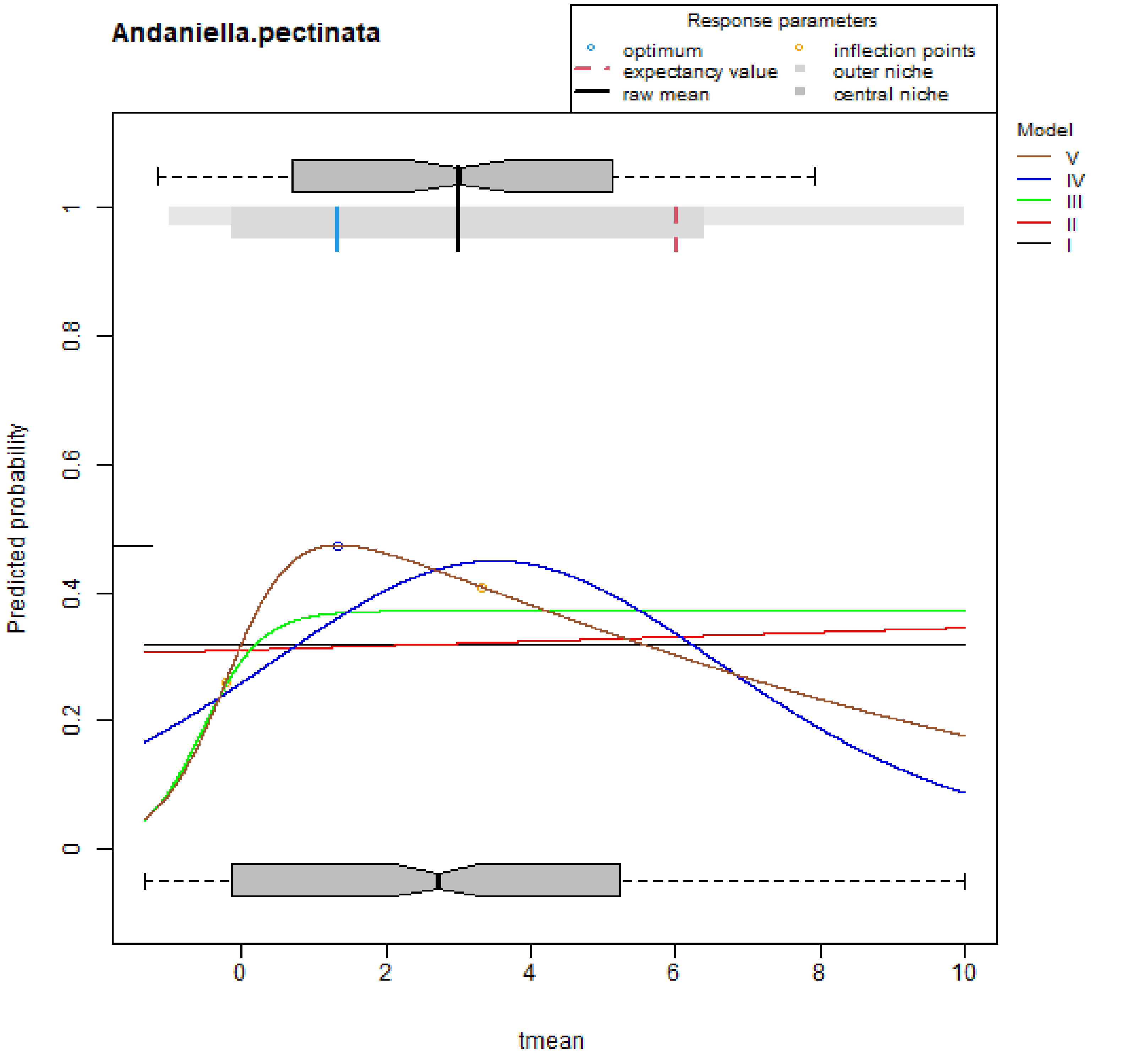
Andaniella.pectinata
Response parameters
optimum
expectancy value
raw mean
inflection points
outer niche
central niche
Model
V
IV
III
II
I
Predicted probability
0
0.2
0.4
0.6
0.8
1
0
2
4
6
8
10
tmean

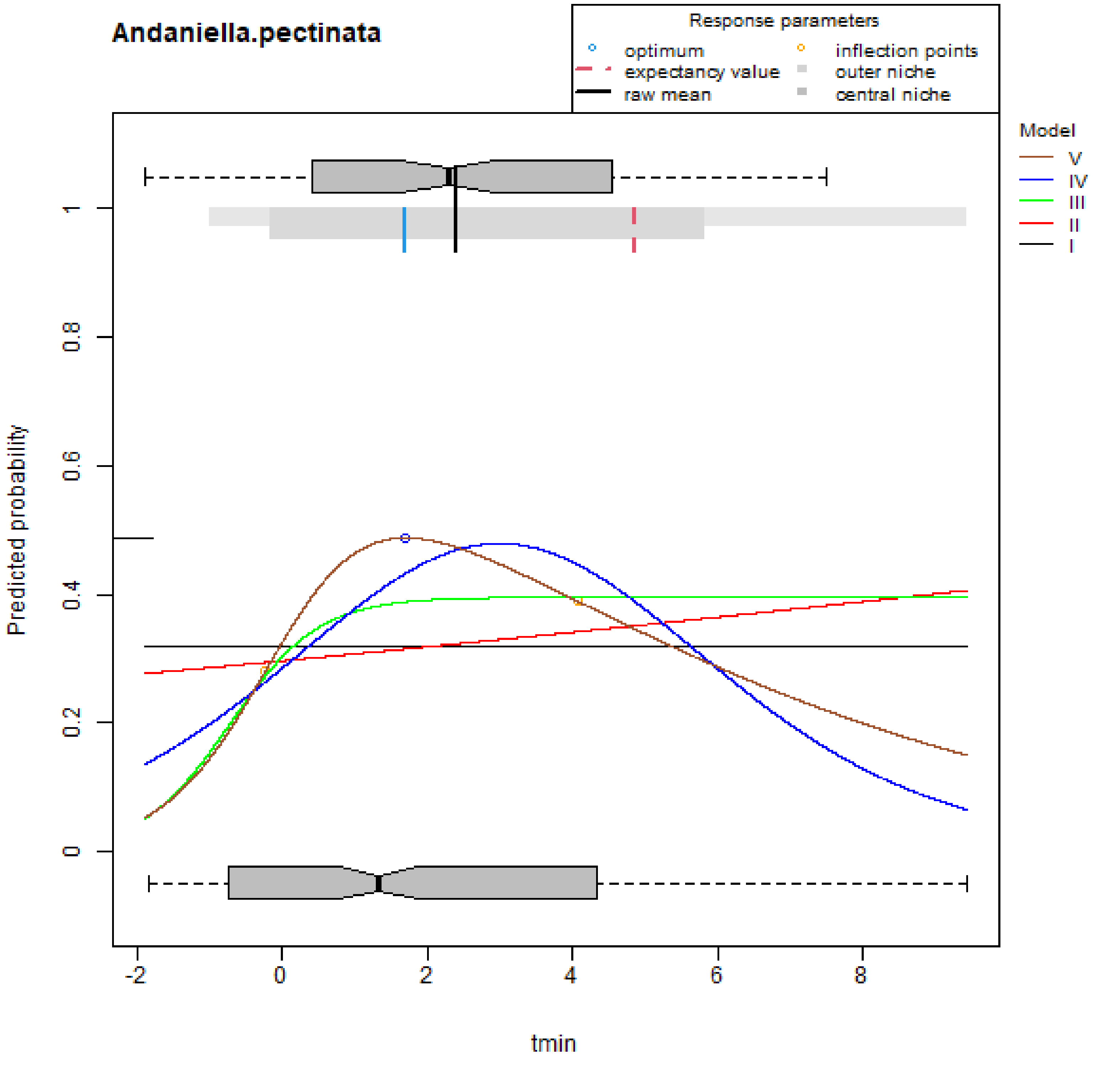

Andaniella.pectinata
Response parameters
optimum
expectancy value
raw mean
inflection points
outer niche
central niche
Model
V
IV
III
II
I
Predicted probability
0
0.2
0.4
0.6
0.8
1
-2
0
2
4
6
8
tmin

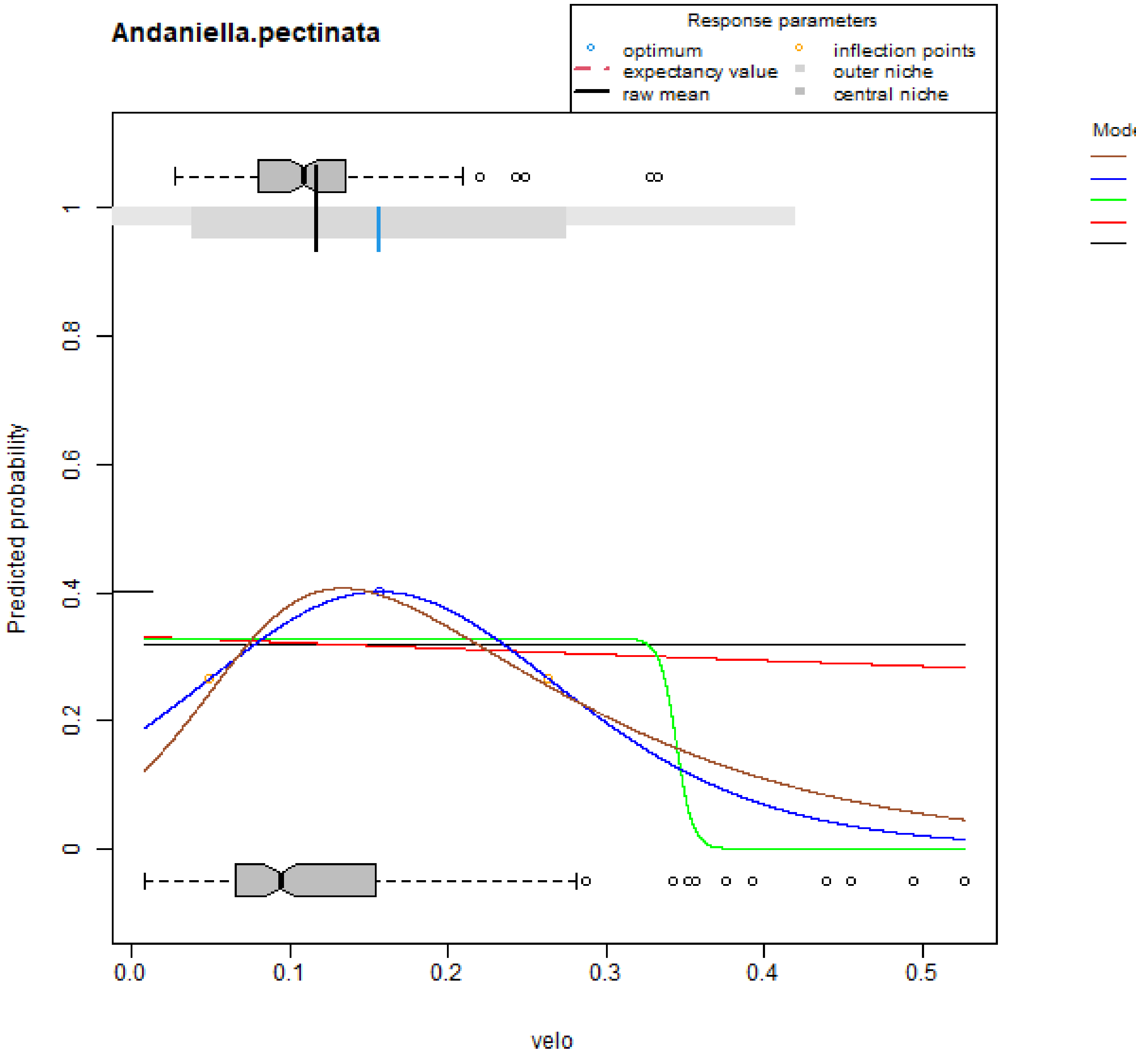
Andaniella.pectinata
Response parameters
optimum
expectancy value
raw mean
inflection points
outer niche
central niche
Mode
Predicted probability
0
0.2
0.4
0.6
0.8
1
0.0
0.1
0.2
0.3
0.4
0.5
velo

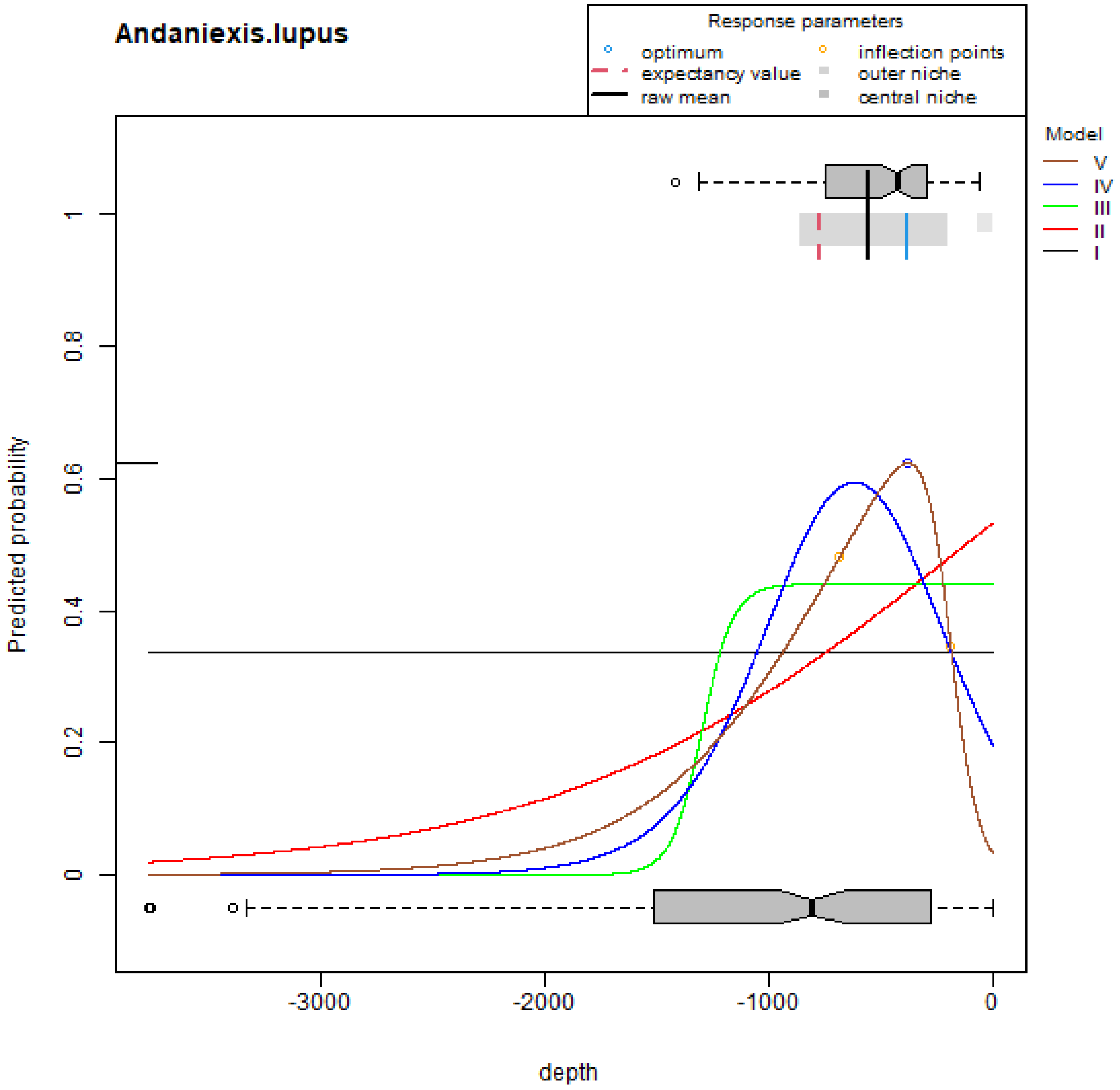
Andaniexis.lupus
Response parameters
optimum
expectancy value
raw mean
inflection points
outer niche
central niche
Model
V
IV
III
II
I
Predicted probability
0
0.2
0.4
0.6
0.8
1
-3000
-2000
-1000
0
depth

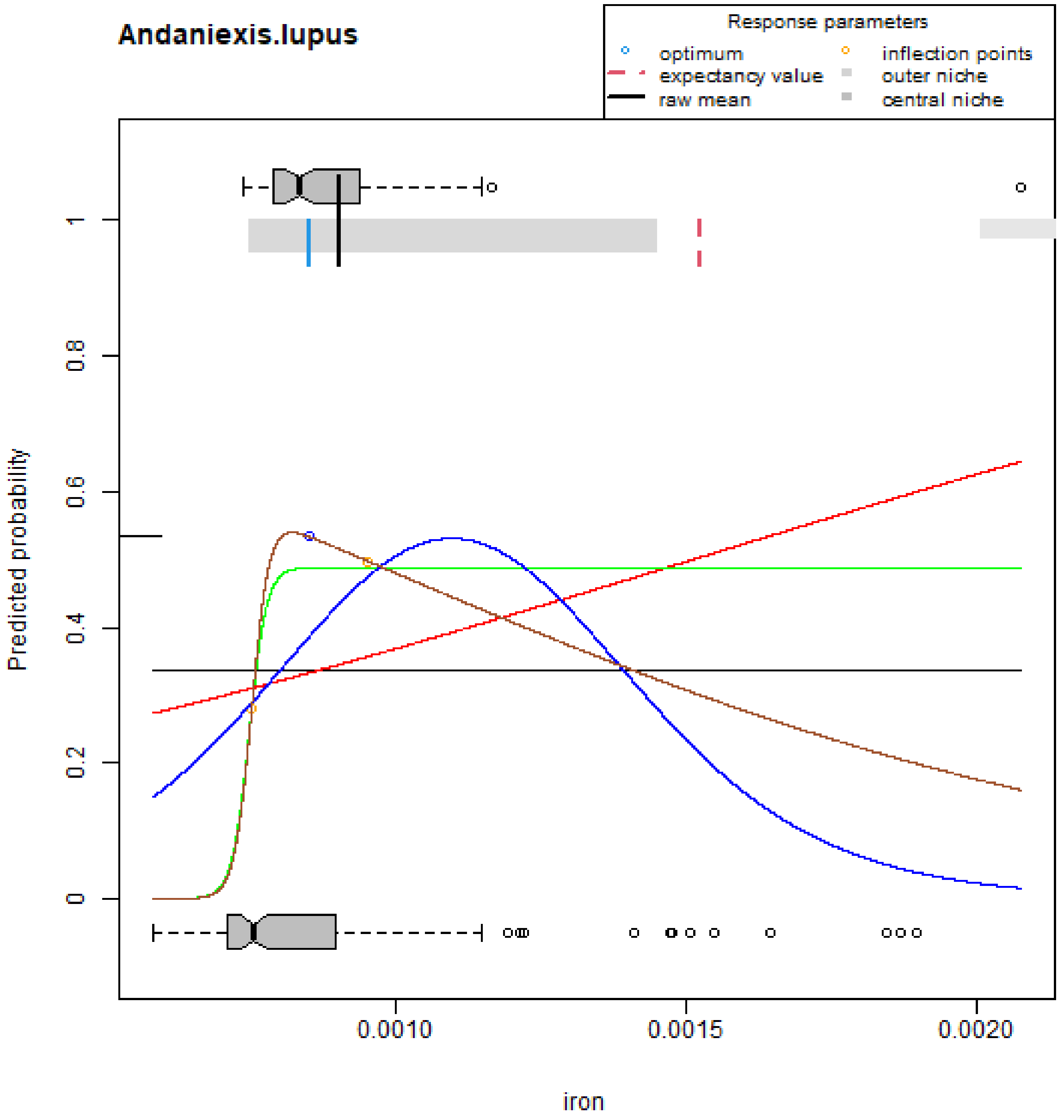

Andaniexis.lupus
Response parameters
optimum
expectancy value
raw mean
inflection points
outer niche
central niche
Predicted probability
0
0.2
0.4
0.6
0.8
1
0.0010
0.0015
0.0020
iron

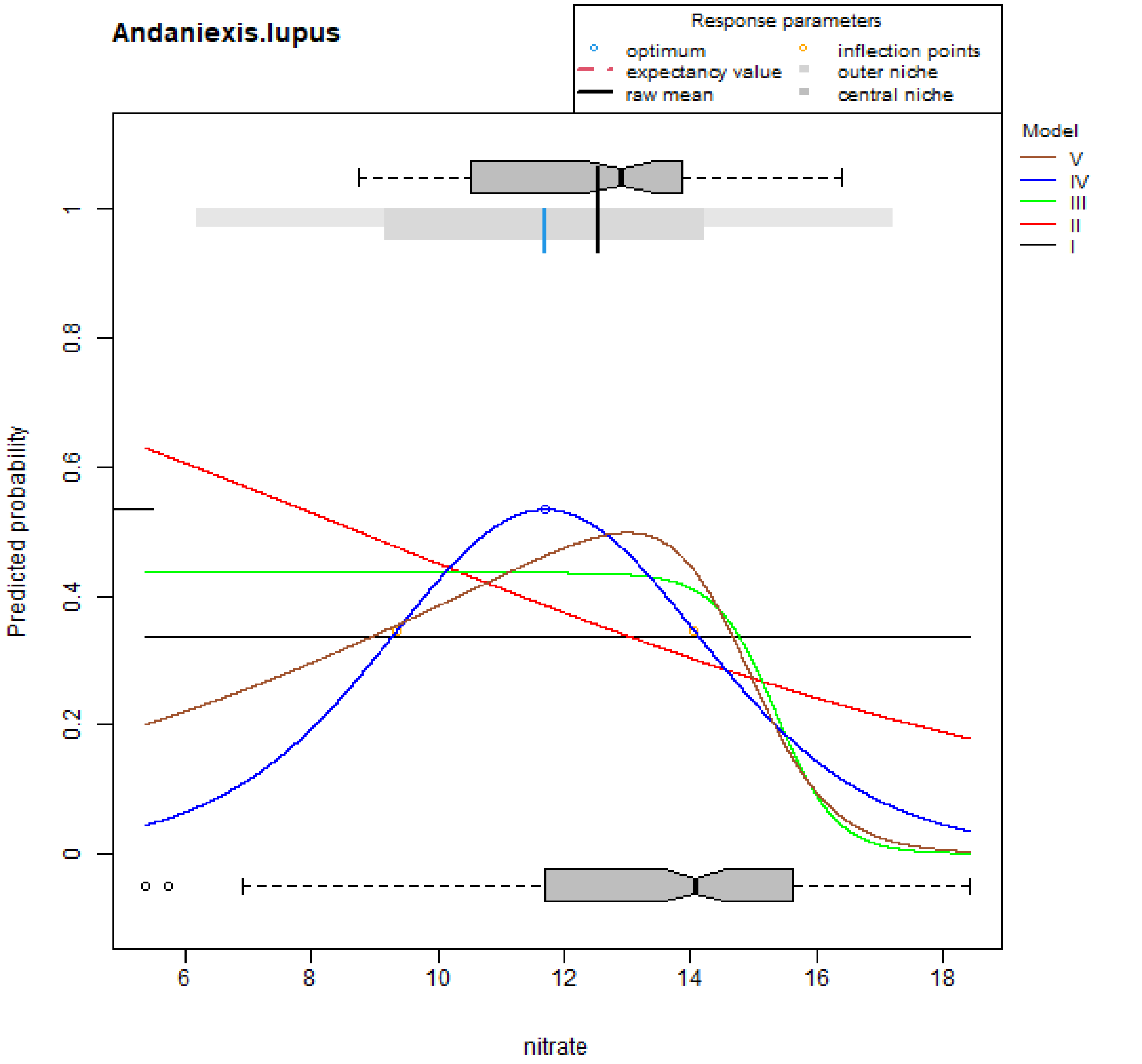
Andaniexis.lupus
Response parameters
optimum
expectancy value
raw mean
inflection points
outer niche
central niche
Model
V
IV
III
II
I
Predicted probability
0
0.2
0.4
0.6
0.8
1
6
8
10
12
14
16
18
nitrate

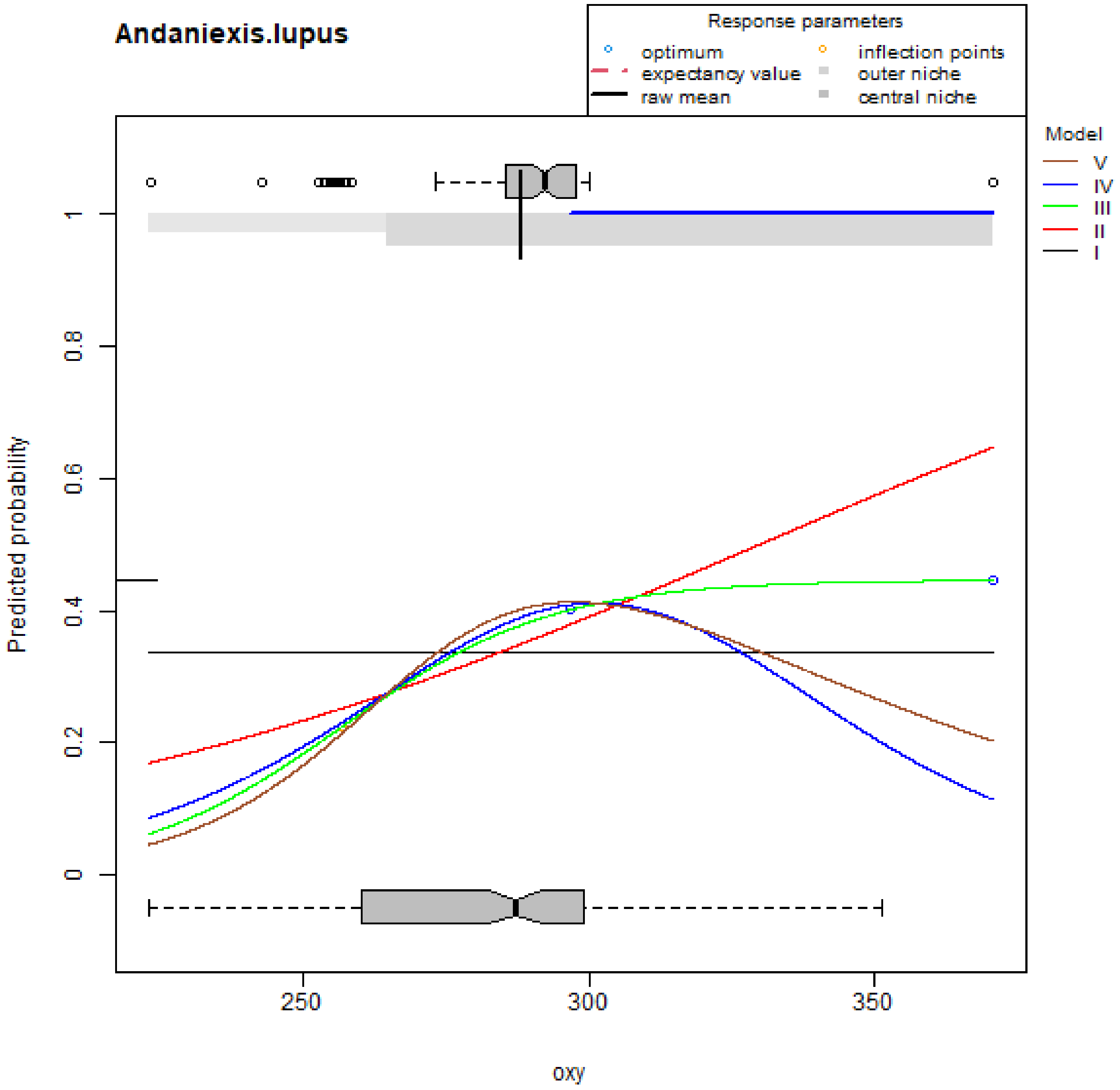

Andaniexis.lupus
Response parameters
optimum
expectancy value
raw mean
inflection points
outer niche
central niche
Model
V
IV
III
II
I
Predicted probability
0
0.2
0.4
0.6
0.8
1
250
300
350
oxy

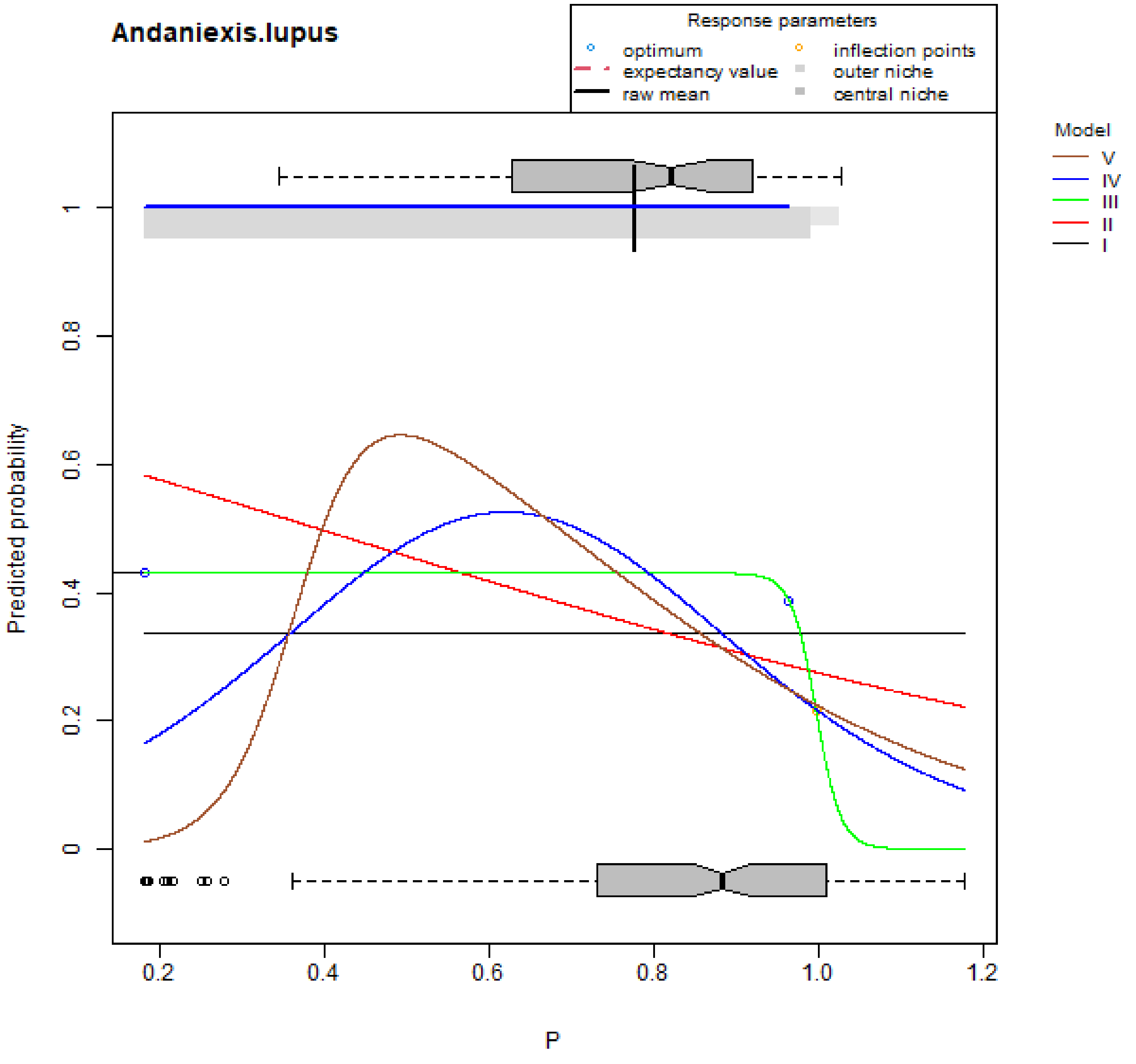

Andaniexis.lupus
Response parameters
optimum
expectancy value
raw mean
inflection points
outer niche
central niche
Model
V
IV
III
II
I
Predicted probability
0
0.2
0.4
0.6
0.8
1
0.2
0.4
0.6
0.8
1.0
1.2
P

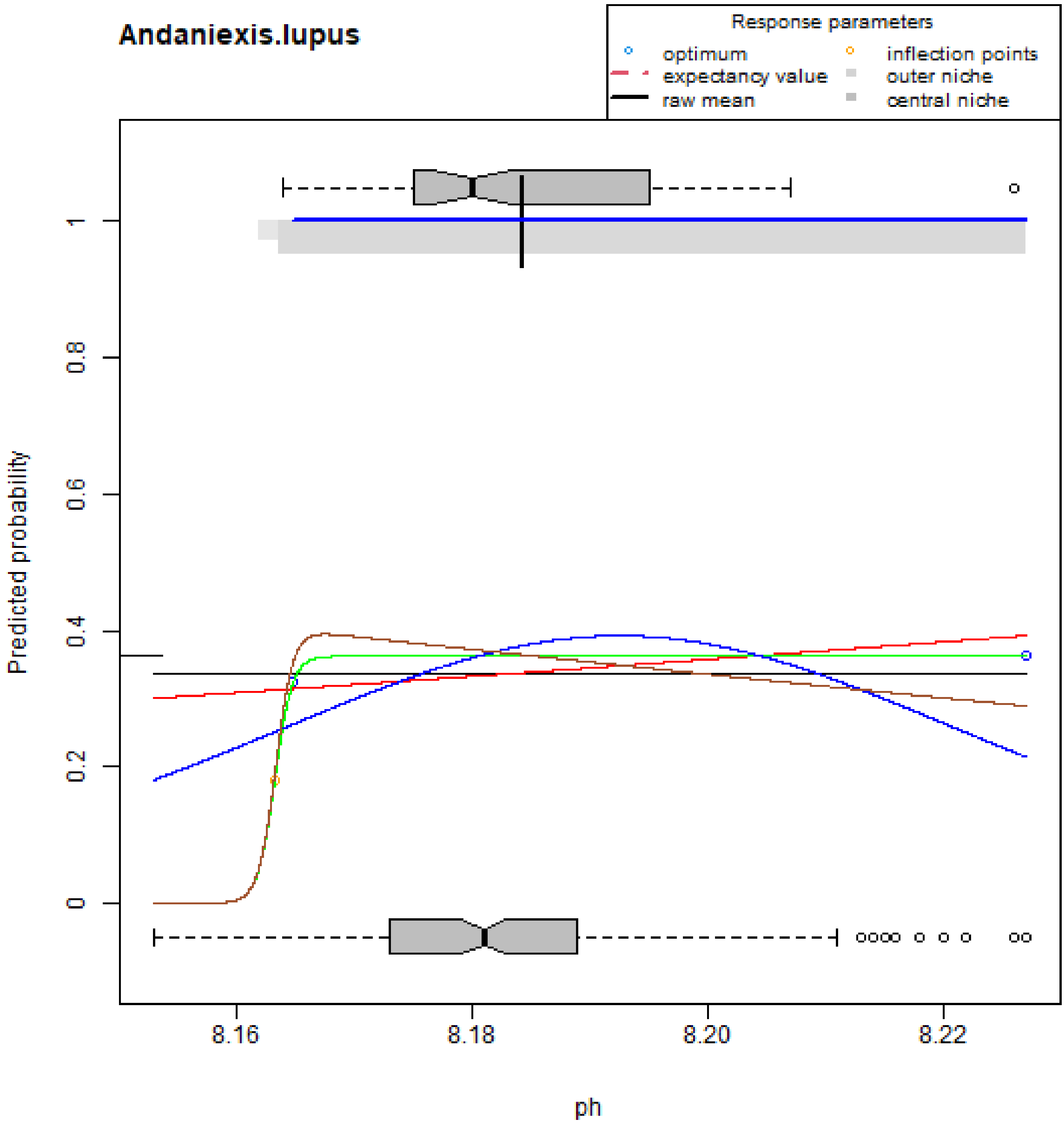

Andaniexis.lupus
Response parameters
optimum
expectancy value
raw mean
inflection points
outer niche
central niche
Predicted probability
0
0.2
0.4
0.6
0.8
1
8.16
8.18
8.20
8.22
ph

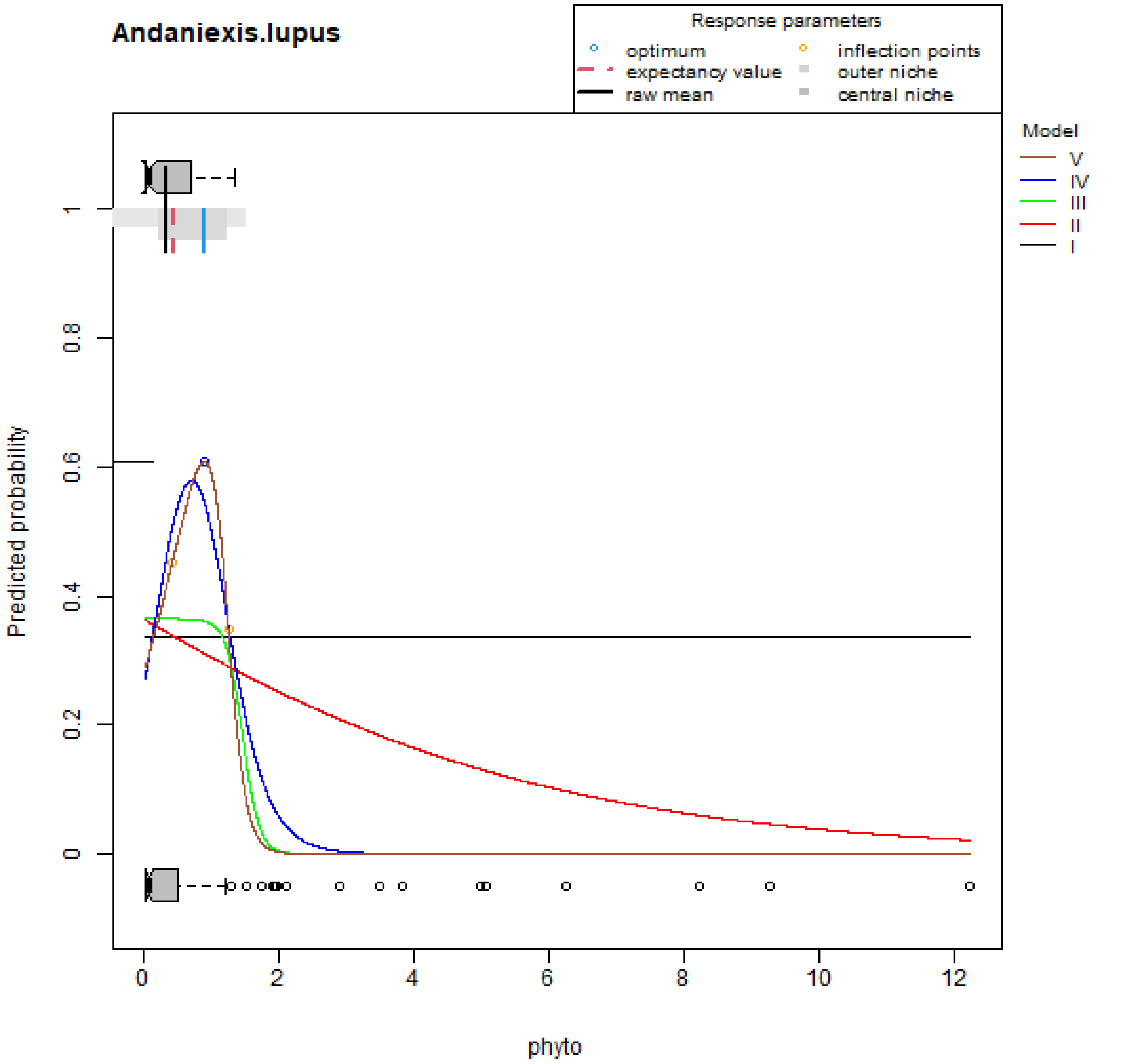
Andaniexis.lupus
Response parameters
optimum
expectancy value
raw mean
inflection points
outer niche
central niche
Model
V
IV
III
II
I
Predicted probability
0
0.2
0.4
0.6
0.8
1
0
2
4
6
8
10
12
phyto

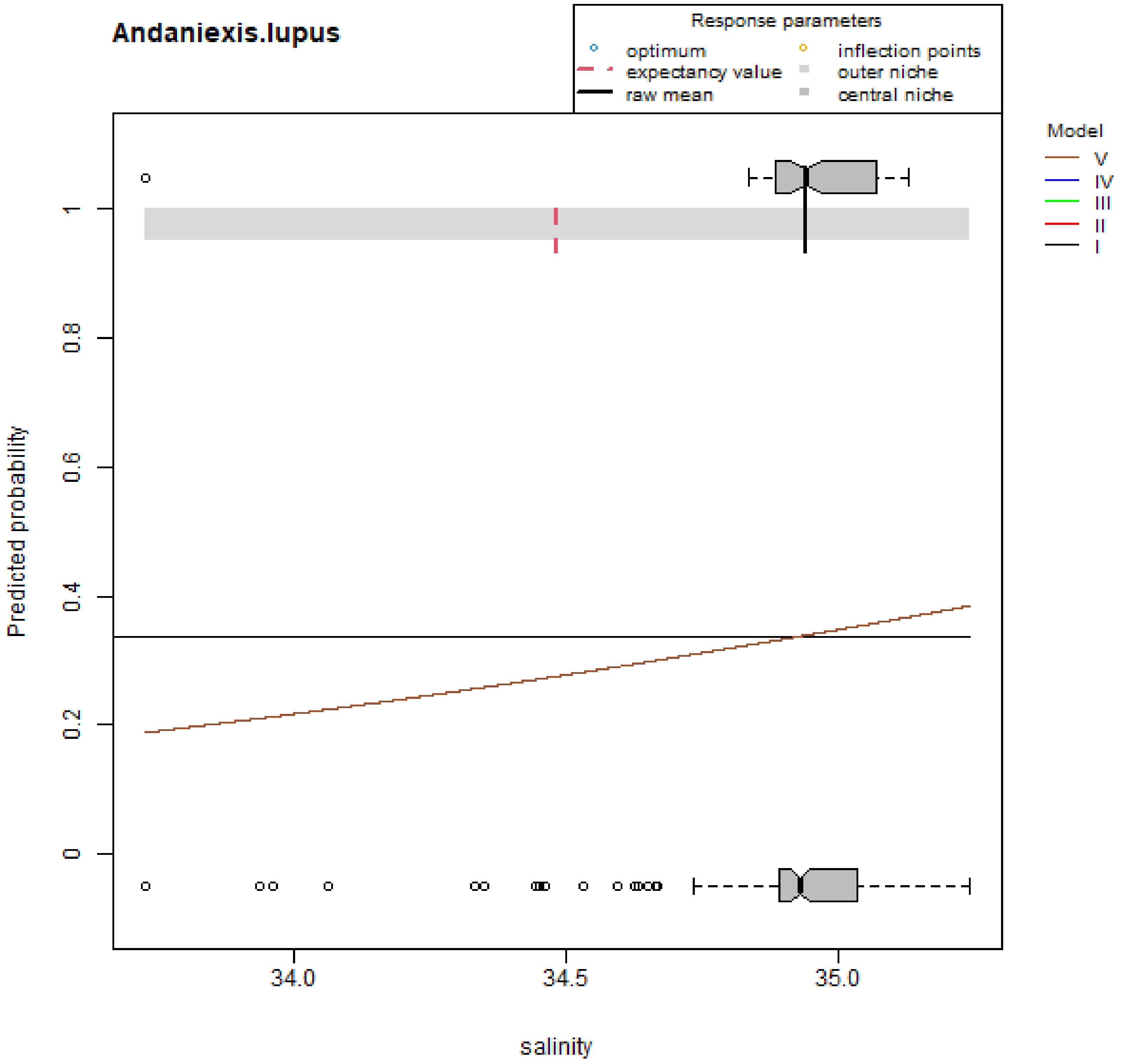
Andaniexis.lupus
Response parameters
optimum
inflection points
expectancy value
outer niche
raw mean
central niche
Model
V
IV
III
II
I
Predicted probability
0
0.2
0.4
0.6
0.8
1
34.0
34.5
35.0
salinity

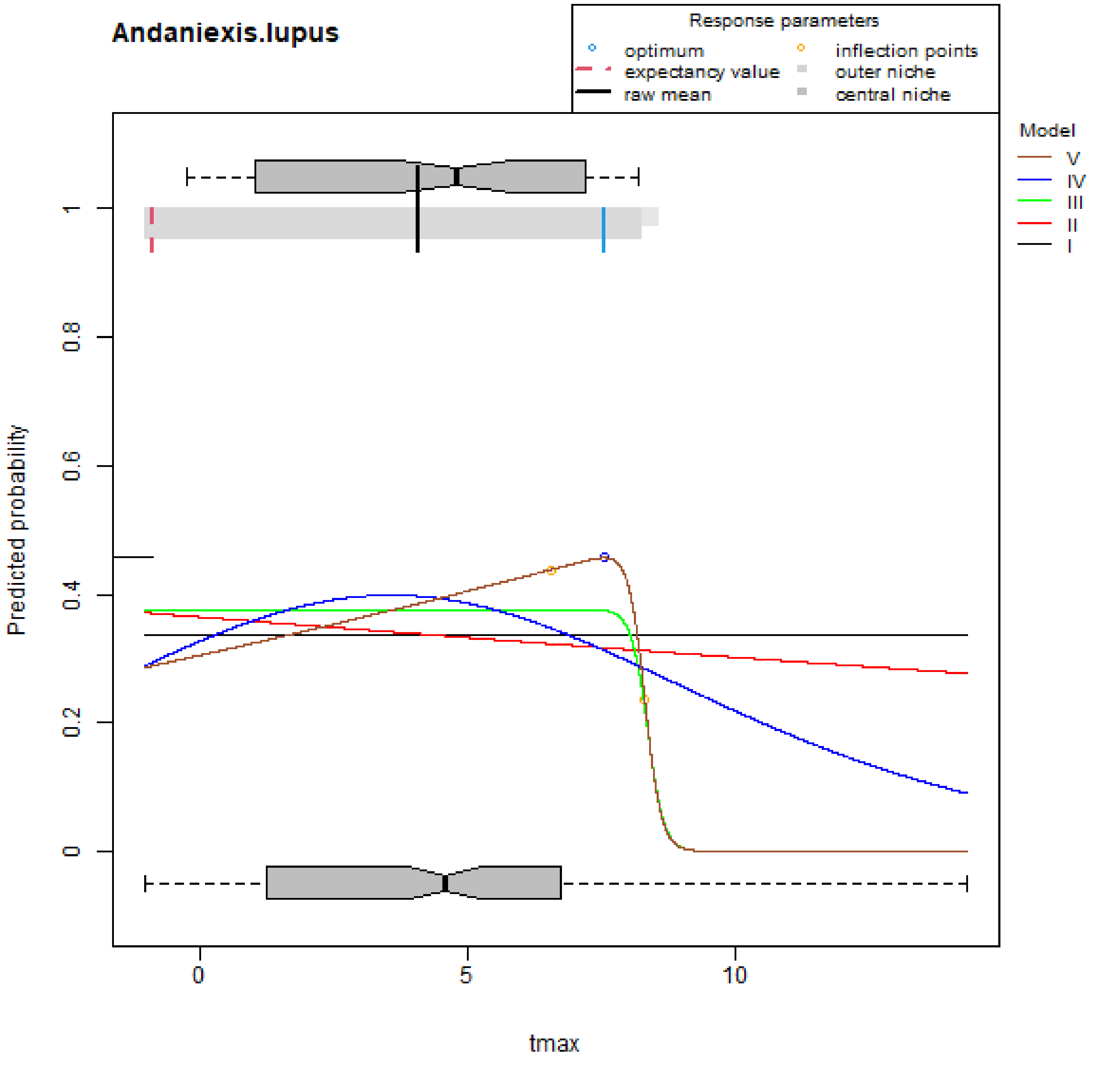

Andaniexis.lupus
Response parameters
optimum
expectancy value
raw mean
inflection points
outer niche
central niche
Model
V
IV
III
II
I
Predicted probability
0
0.2
0.4
0.6
0.8
1
0
5
10
tmax

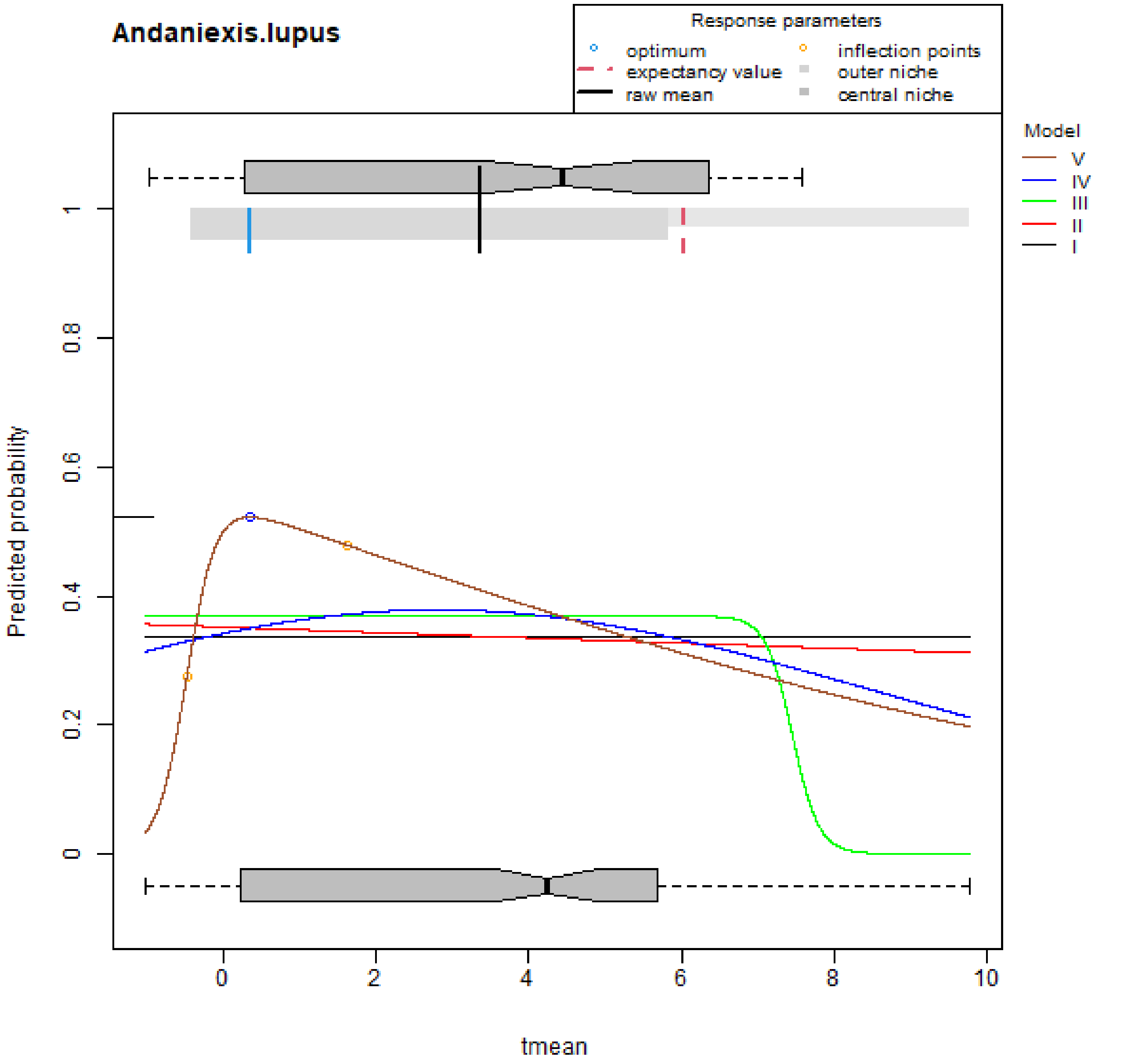

Andaniexis.lupus
Response parameters
optimum
expectancy value
raw mean
inflection points
outer niche
central niche
Model
V
IV
III
II
I
Predicted probability
0
0.2
0.4
0.6
0.8
1
0
2
4
6
8
10
tmean

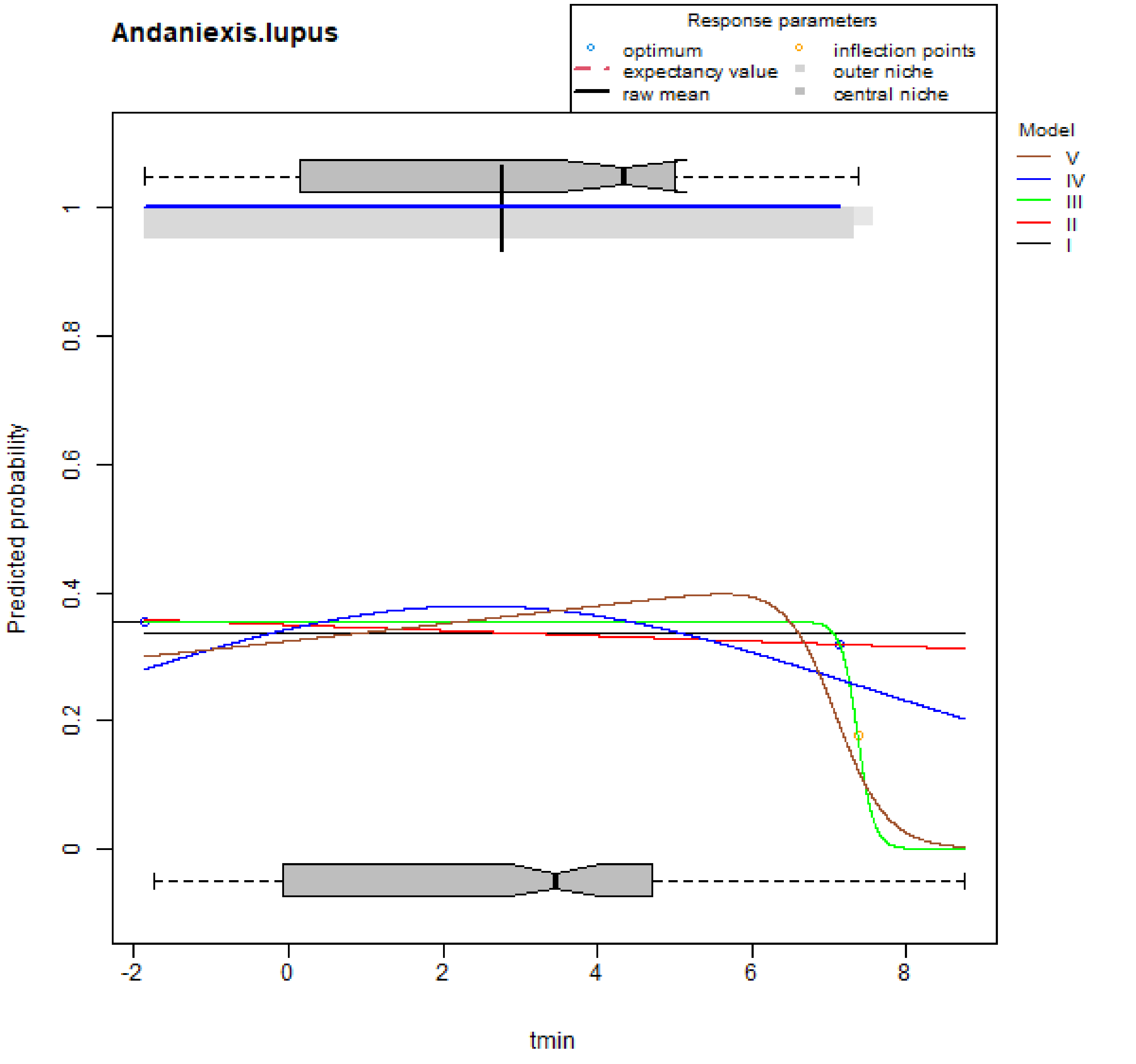
Andaniexis.lupus
Response parameters
optimum
inflection points
expectancy value
outer niche
raw mean
central niche
Model
V
IV
III
II
I
Predicted probability
0
0.2
0.4
0.6
0.8
1
-2
0
2
4
6
8
tmin

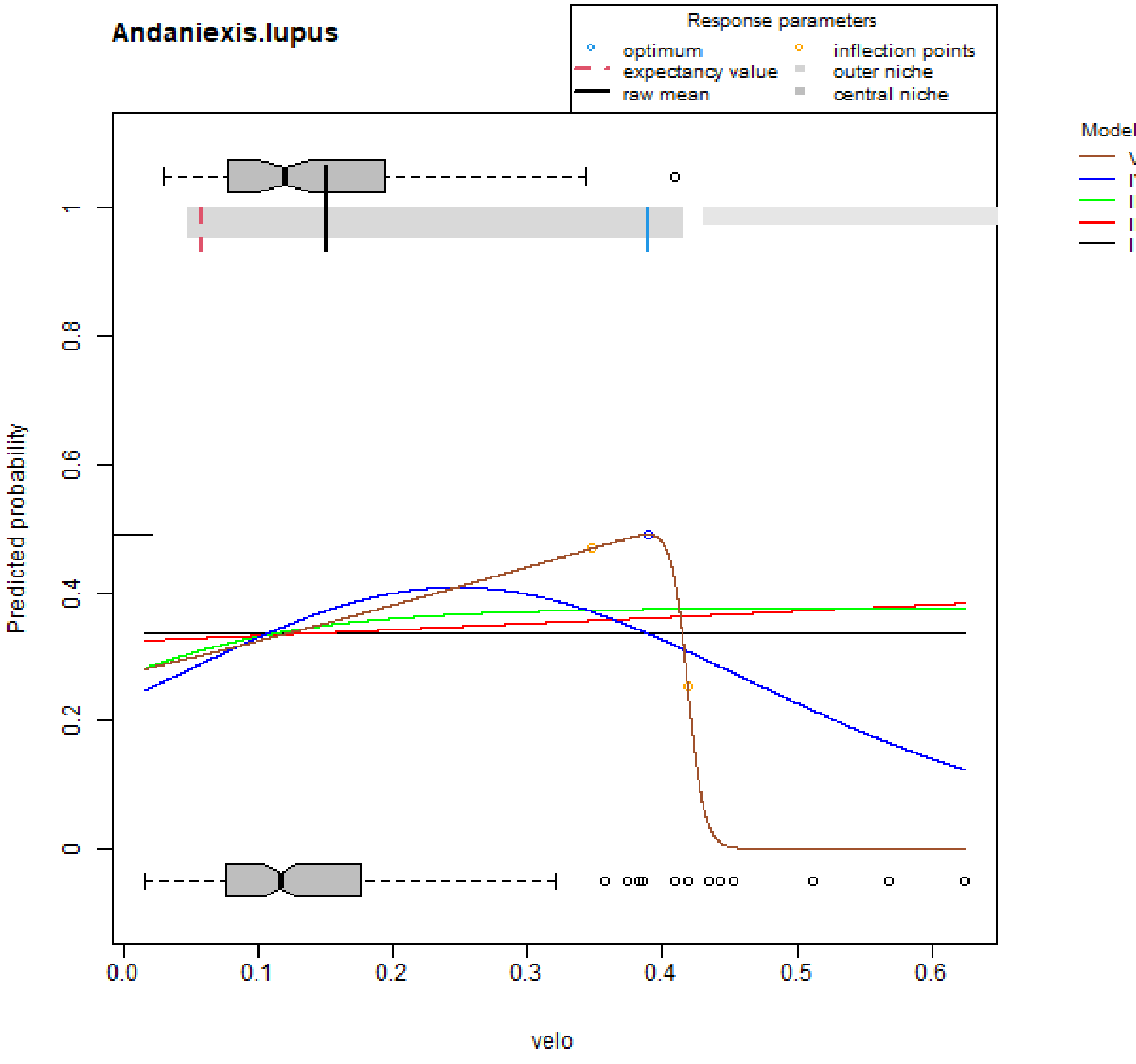
Andaniexis.lupus
Response parameters
optimum
expectancy value
raw mean
inflection points
outer niche
central niche
Model
Predicted probability
0
0.2
0.4
0.6
0.8
1
0.0
0.1
0.2
0.3
0.4
0.5
0.6
velo

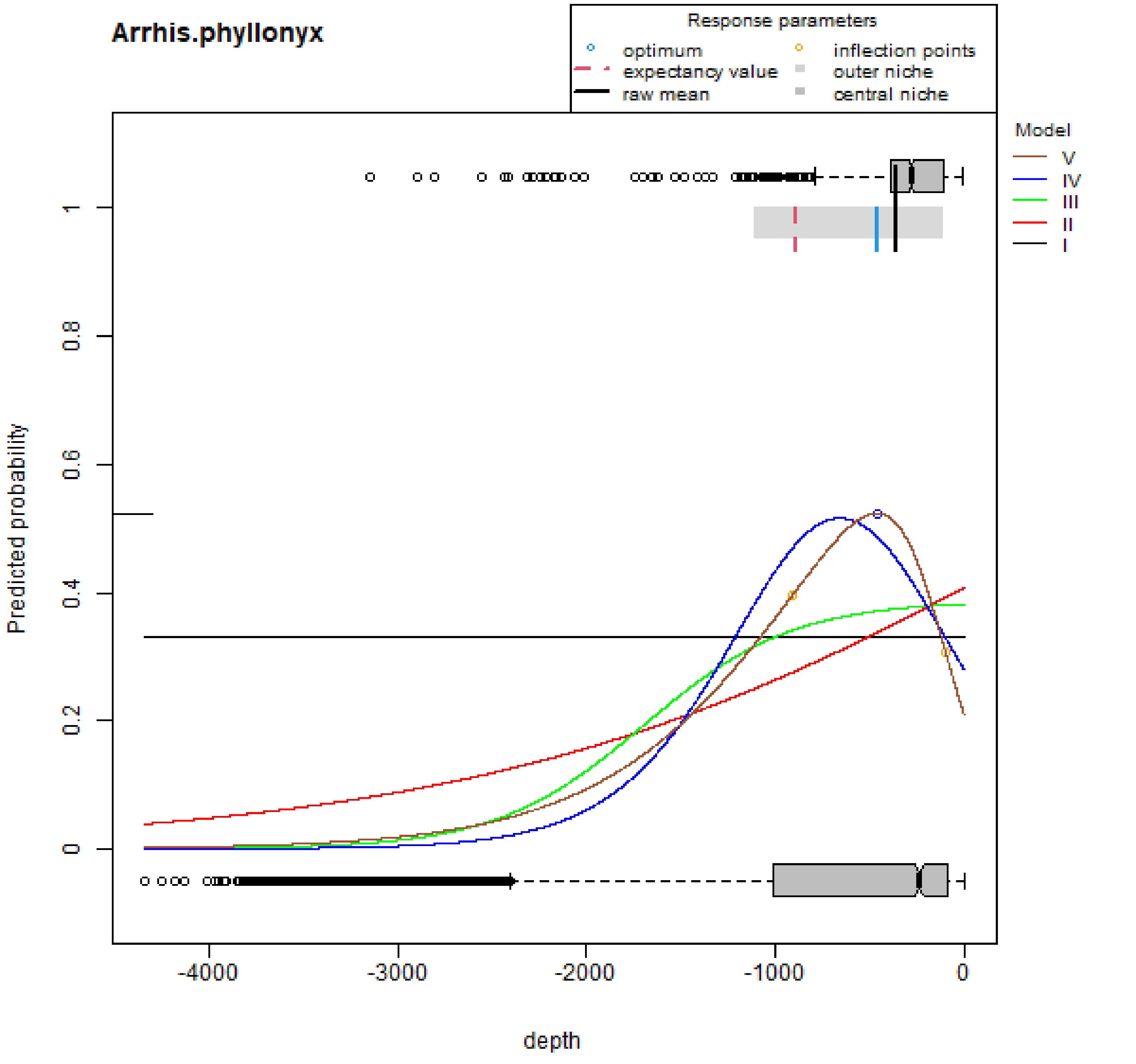
Arrhis.phyllonyx
Response parameters
optimum
expectancy value
raw mean
inflection points
outer niche
central niche
Model
V
IV
III
II
I
Predicted probability
0
0.2
0.4
0.6
0.8
1
-4000
-3000
-2000
-1000
0
depth

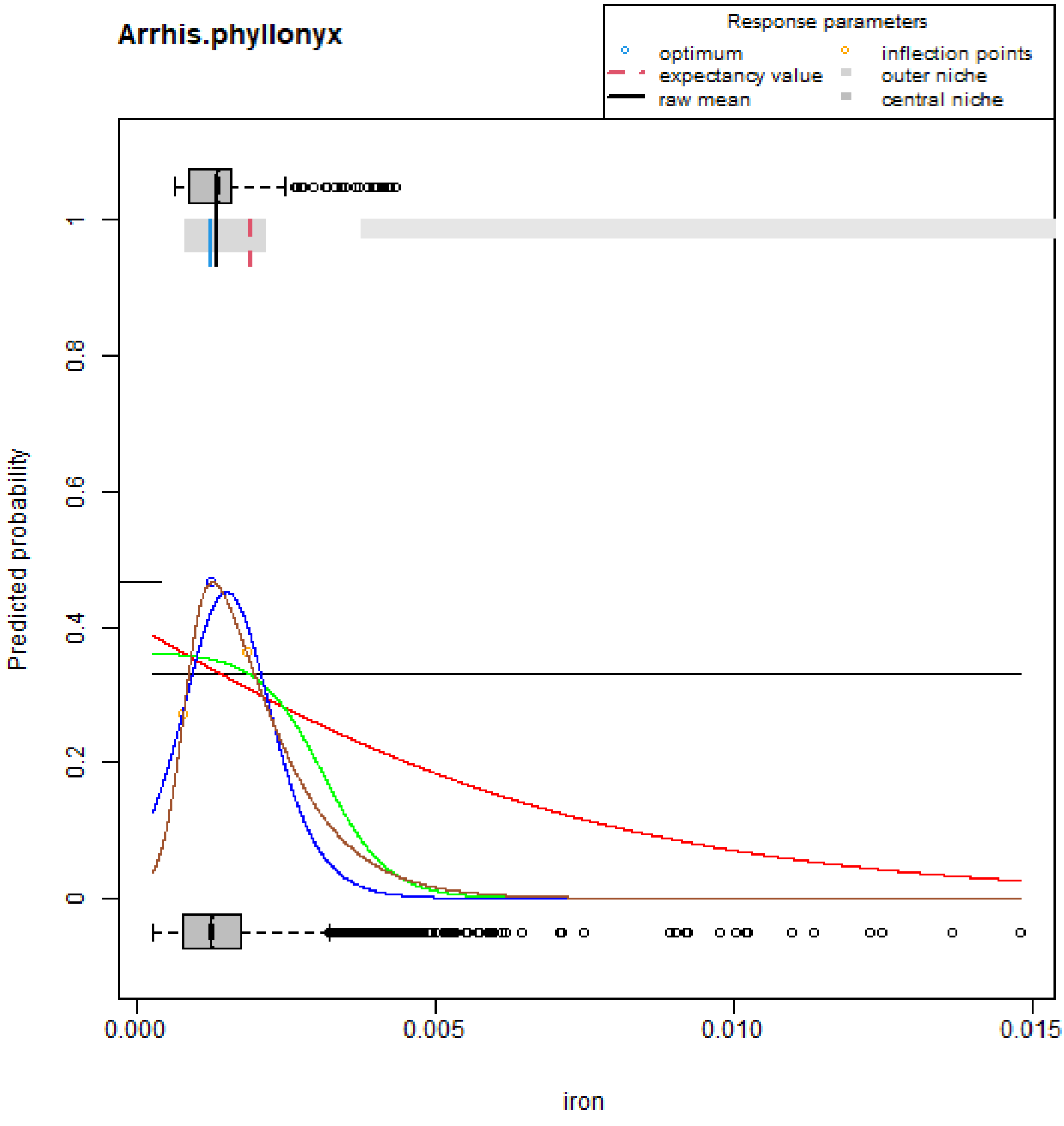

Arrhis.phyllonyx
Response parameters
optimum
expectancy value
raw mean
inflection points
outer niche
central niche
Predicted probability
0
0.2
0.4
0.6
0.8
1
0.000
0.005
0.010
0.015
iron

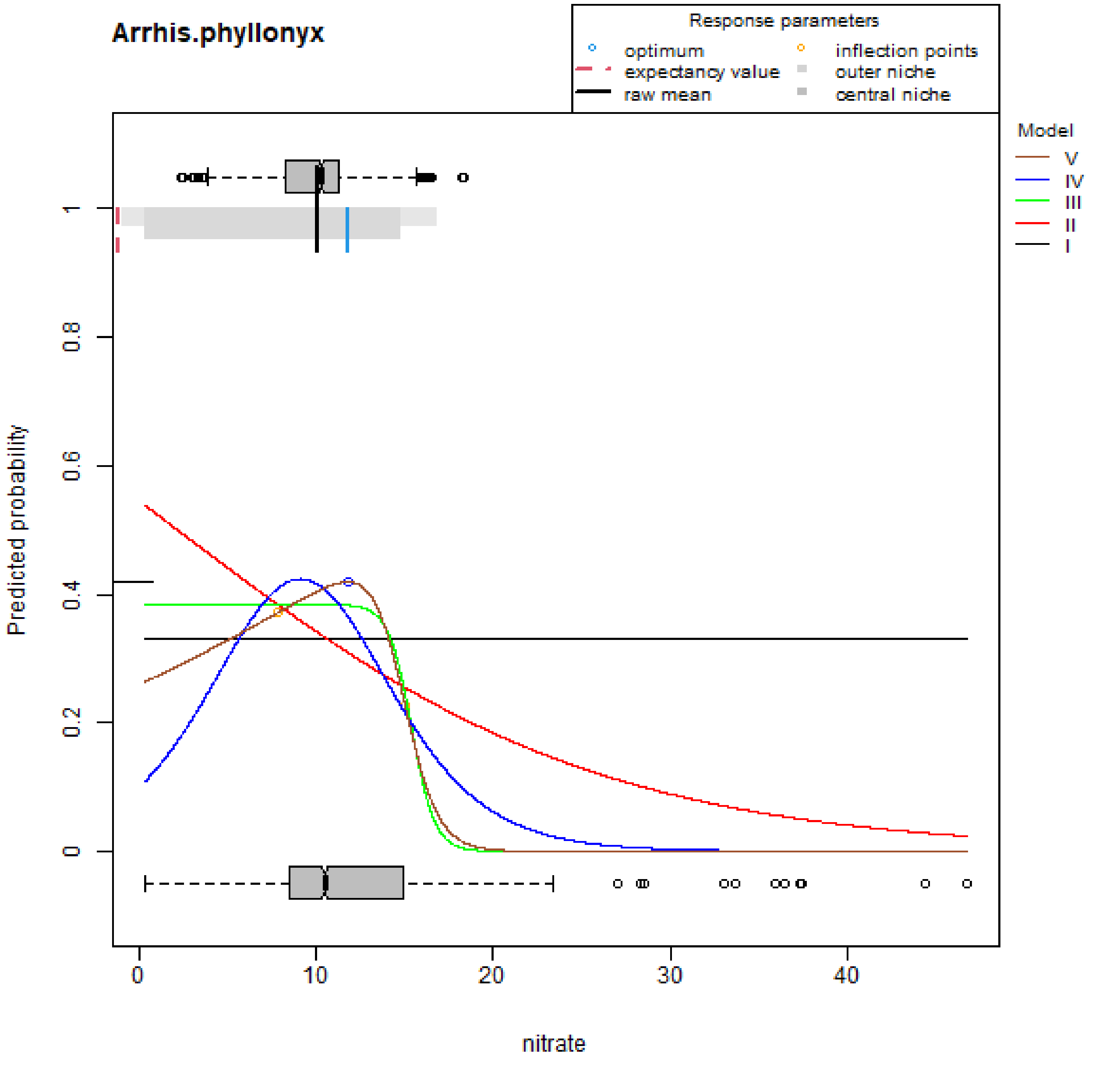

Arrhis.phyllonyx
Response parameters
optimum
expectancy value
raw mean
inflection points
outer niche
central niche
Model
V
IV
III
II
I
Predicted probability
0
0.2
0.4
0.6
0.8
1
0
10
20
30
40
nitrate

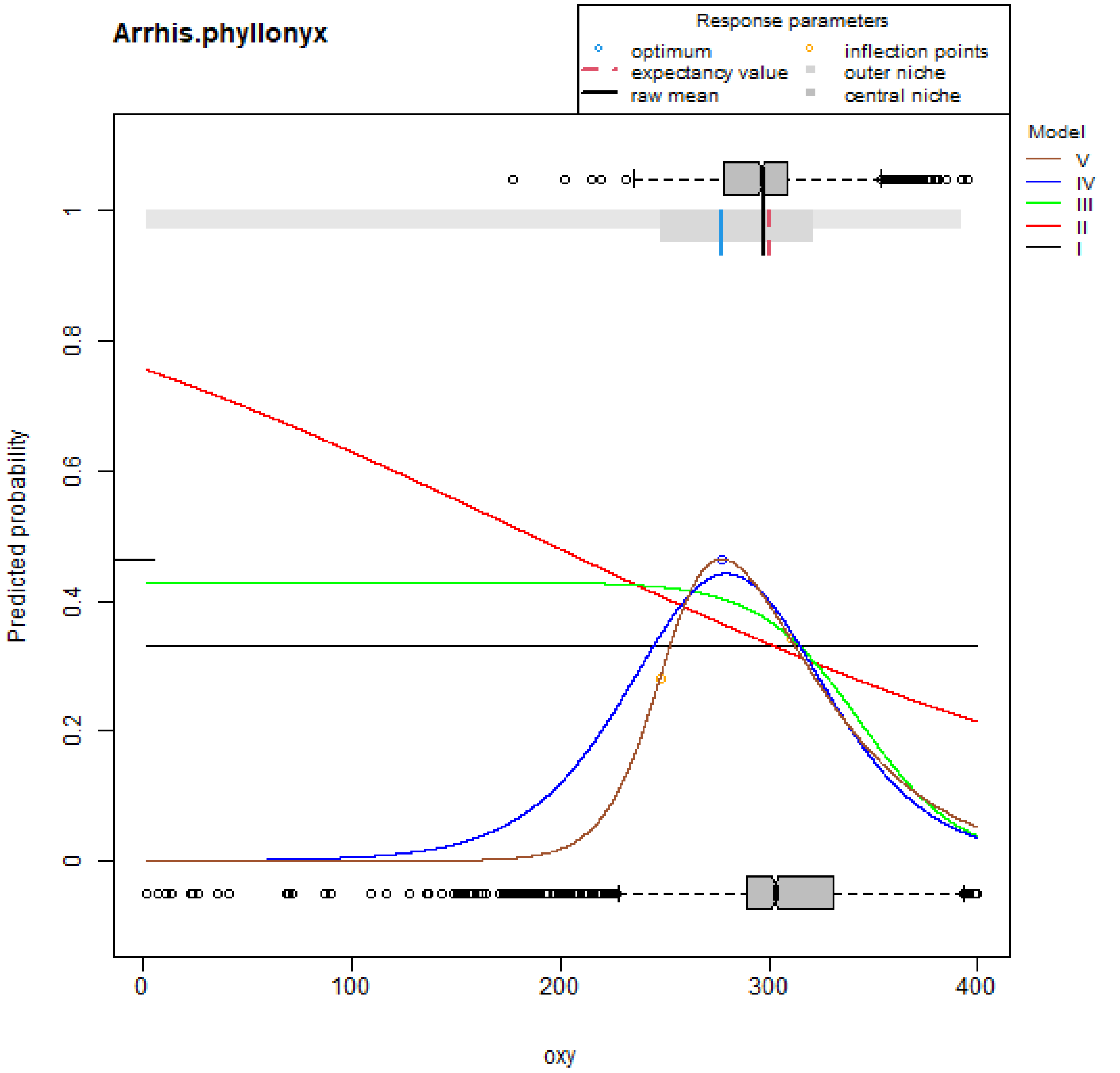
Arrhis.phyllonyx
Response parameters
optimum
expectancy value
raw mean
inflection points
outer niche
central niche
Model
V
IV
III
II
I
Predicted probability
0
0.2
0.4
0.6
0.8
1
0
100
200
300
400
oxy

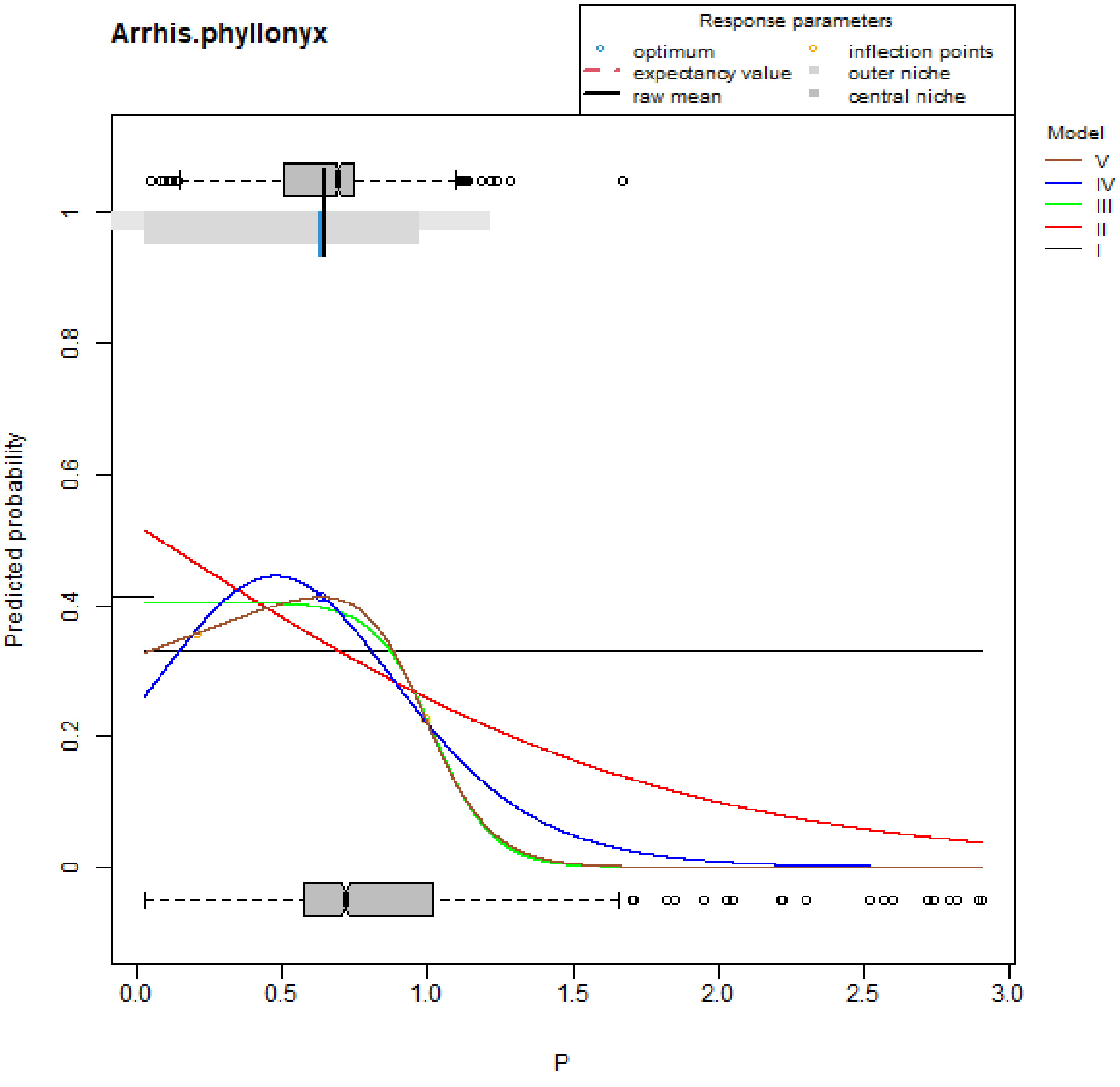

Arrhis.phyllonyx
Response parameters
optimum
expectancy value
raw mean
inflection points
outer niche
central niche
Model
V
IV
III
II
I
Predicted probability
0
0.2
0.4
0.6
0.8
1
0.0
0.5
1.0
1.5
2.0
2.5
3.0
P

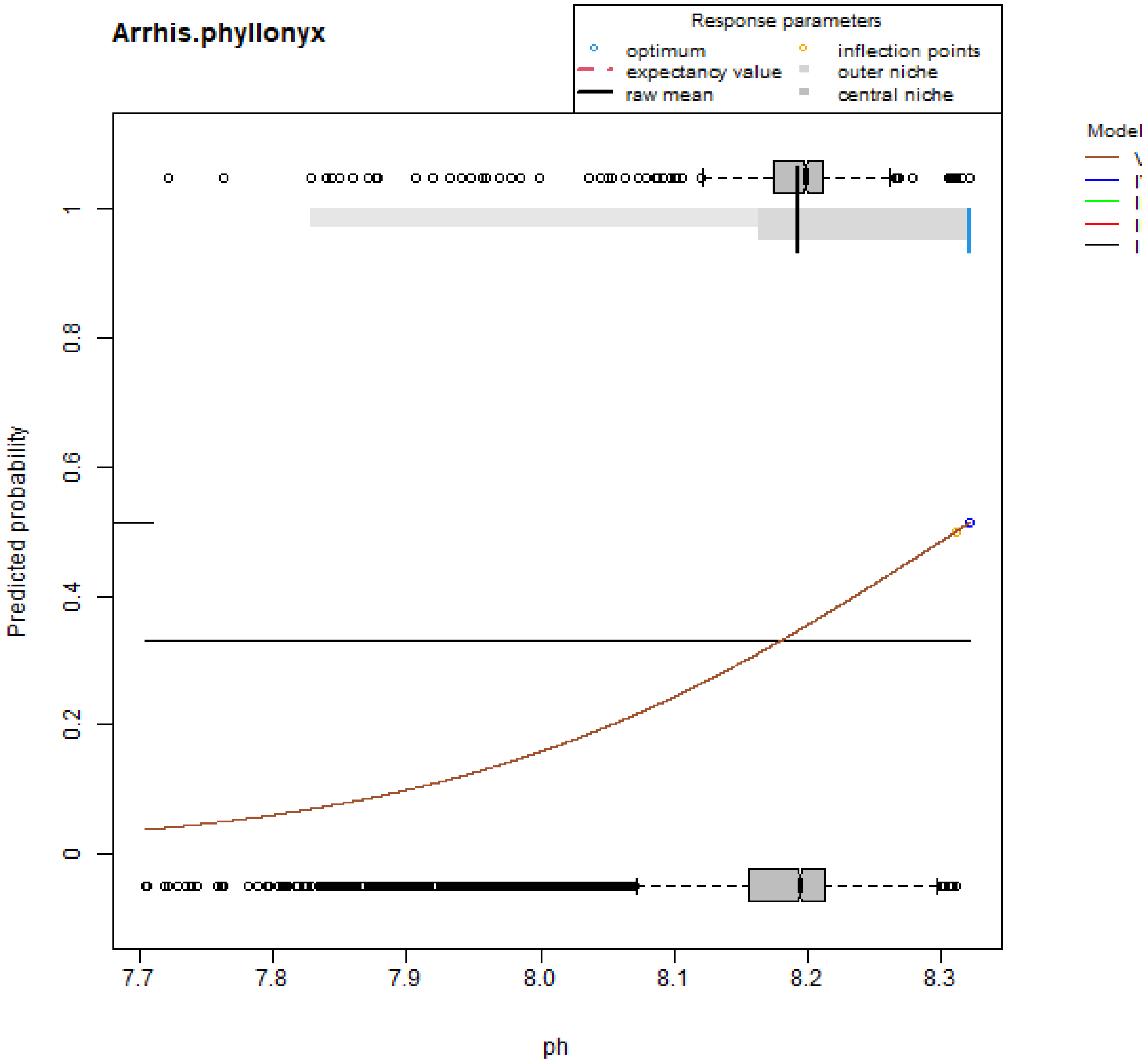

Arrhis.phyllonyx
Response parameters
optimum
expectancy value
raw mean
inflection points
outer niche
central niche
Model
Predicted probability
0
0.2
0.4
0.6
0.8
1
7.7
7.8
7.9
8.0
8.1
8.2
8.3
ph

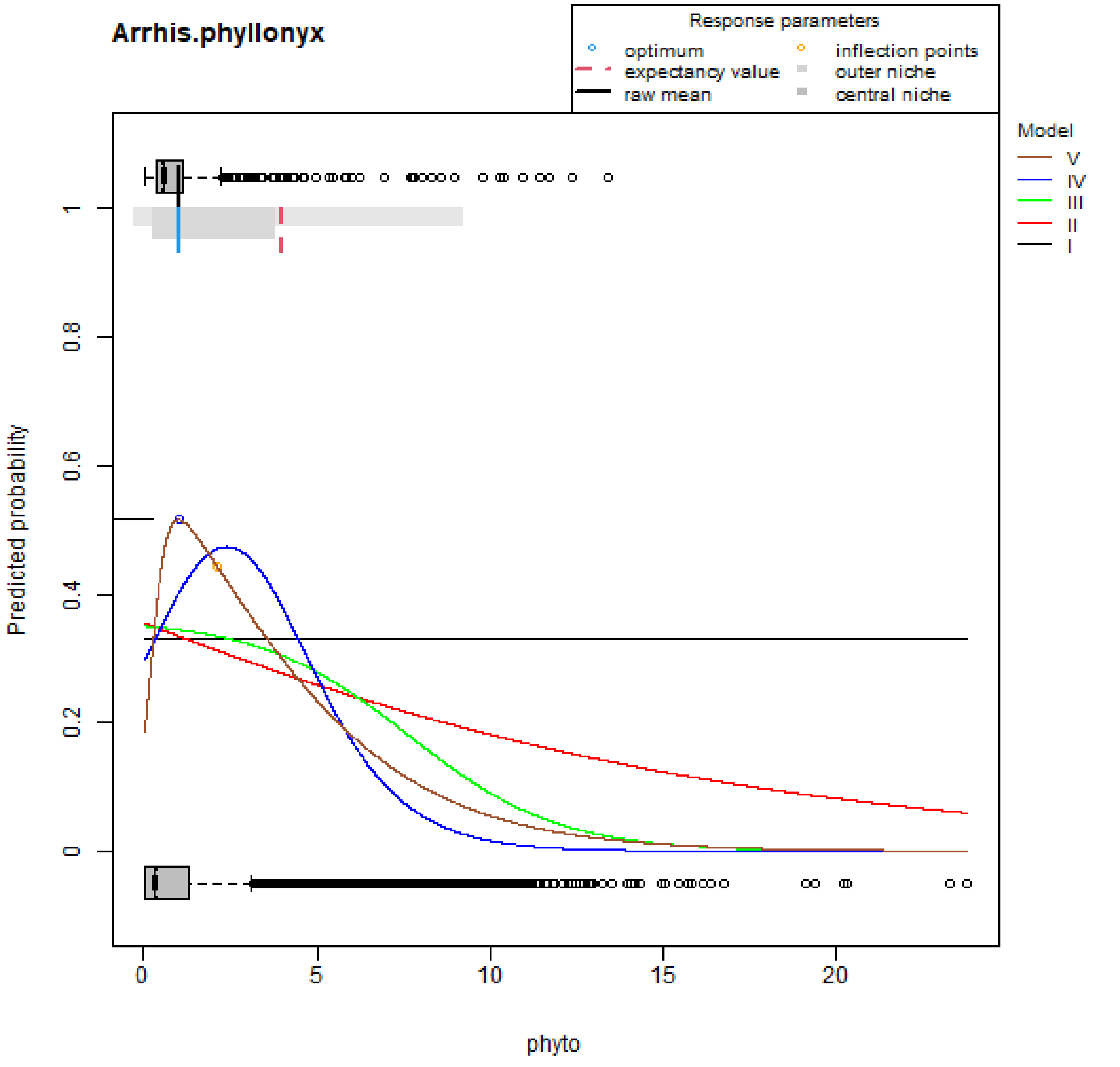

Arrhis.phyllonyx
Response parameters
optimum
expectancy value
raw mean
inflection points
outer niche
central niche
Model
V
IV
III
II
I
Predicted probability
0
0.2
0.4
0.6
0.8
1
0
5
10
15
20
phyto

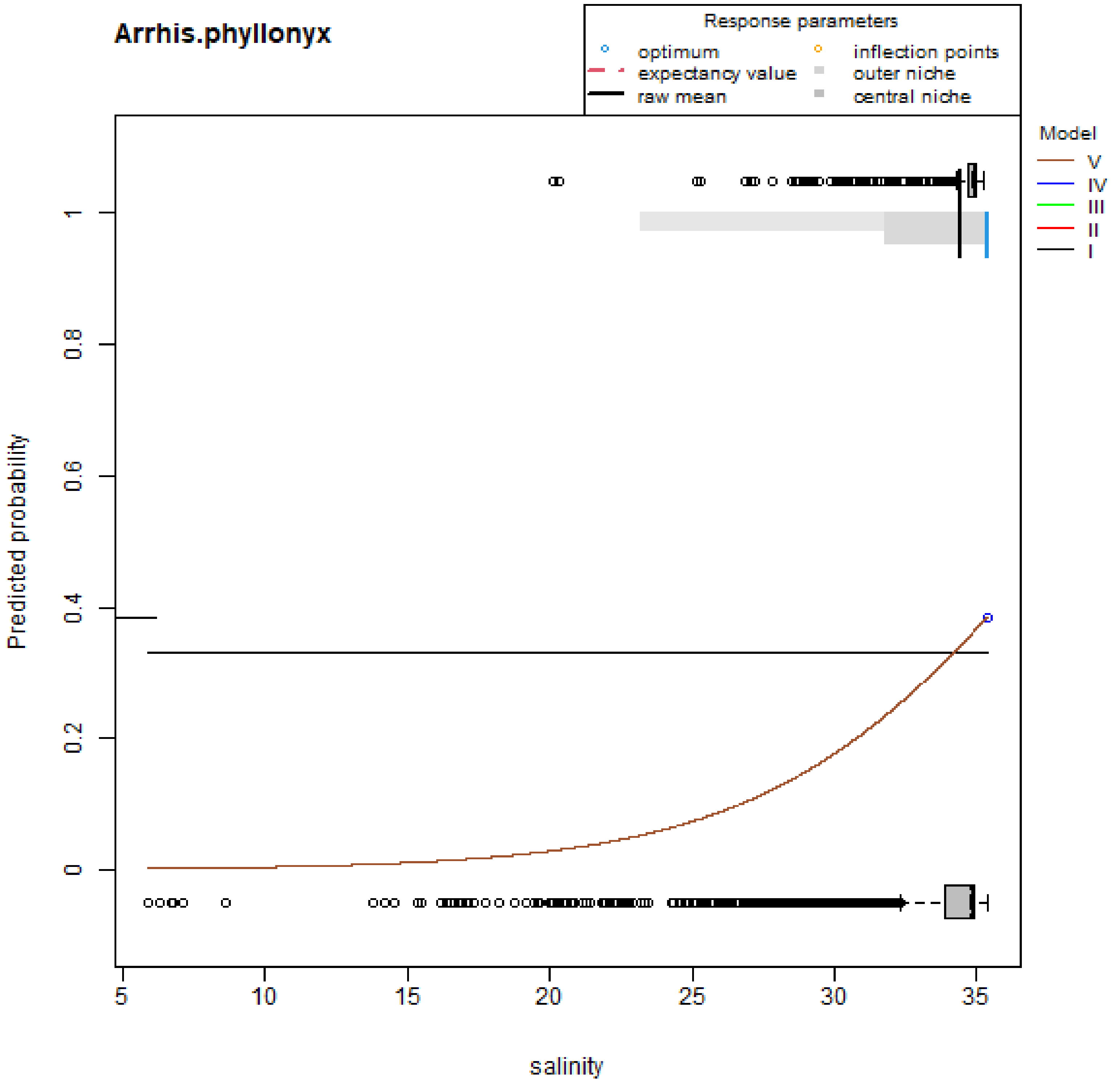

Arrhis.phyllonyx
Response parameters
optimum
expectancy value
raw mean
inflection points
outer niche
central niche
Model
V
IV
III
II
I
Predicted probability
0
0.2
0.4
0.6
0.8
1
5
10
15
20
25
30
35
salinity

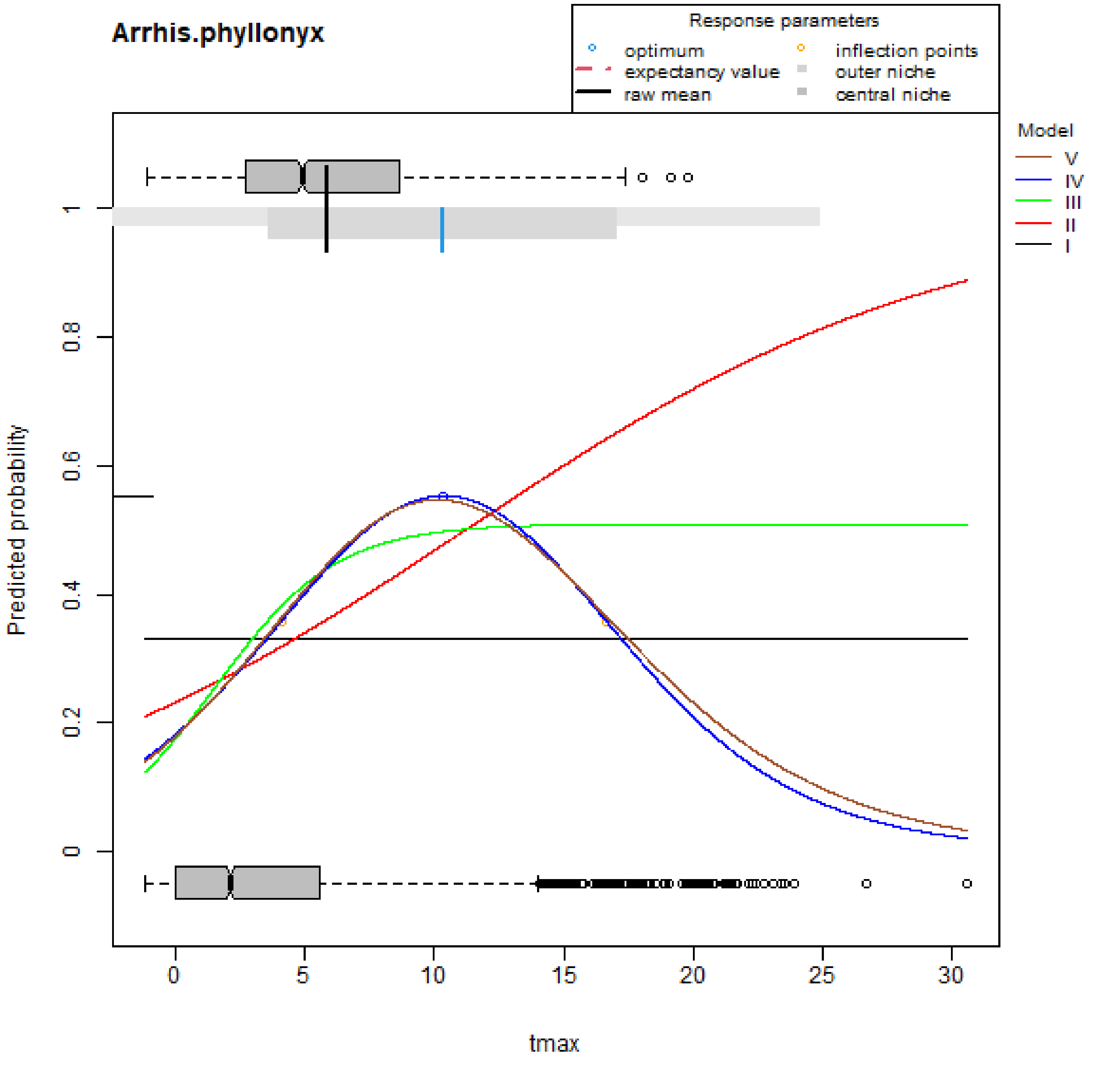

Arrhis.phyllonyx
Response parameters
optimum
expectancy value
raw mean
inflection points
outer niche
central niche
Model
V
IV
III
II
I
Predicted probability
0
0.2
0.4
0.6
0.8
1
0
5
10
15
20
25
30
tmax

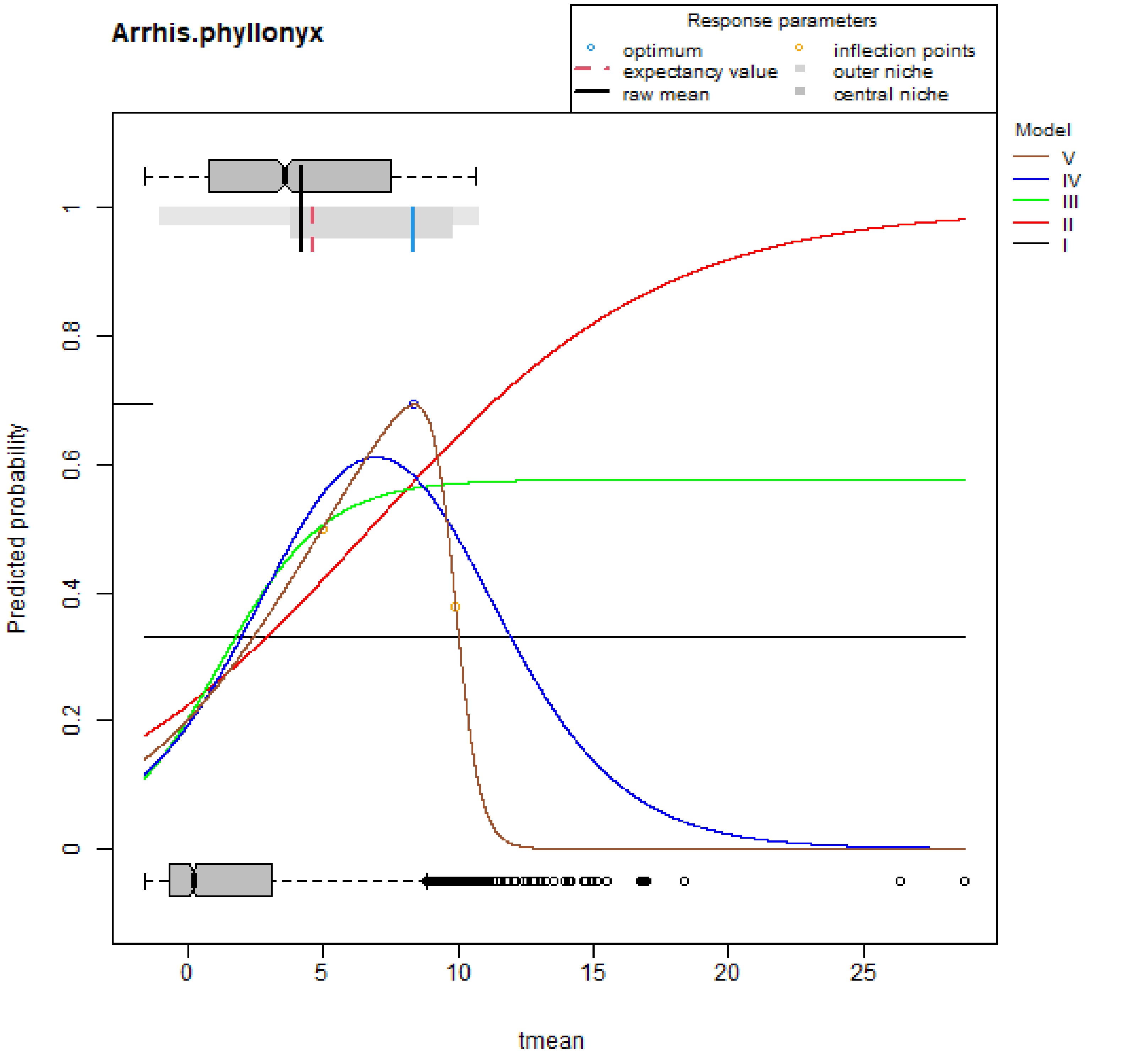

Arrhis.phyllonyx
Response parameters
optimum
expectancy value
raw mean
inflection points
outer niche
central niche
Model
V
IV
III
II
I
Predicted probability
0
0.2
0.4
0.6
0.8
1
0
5
10
15
20
25
tmean

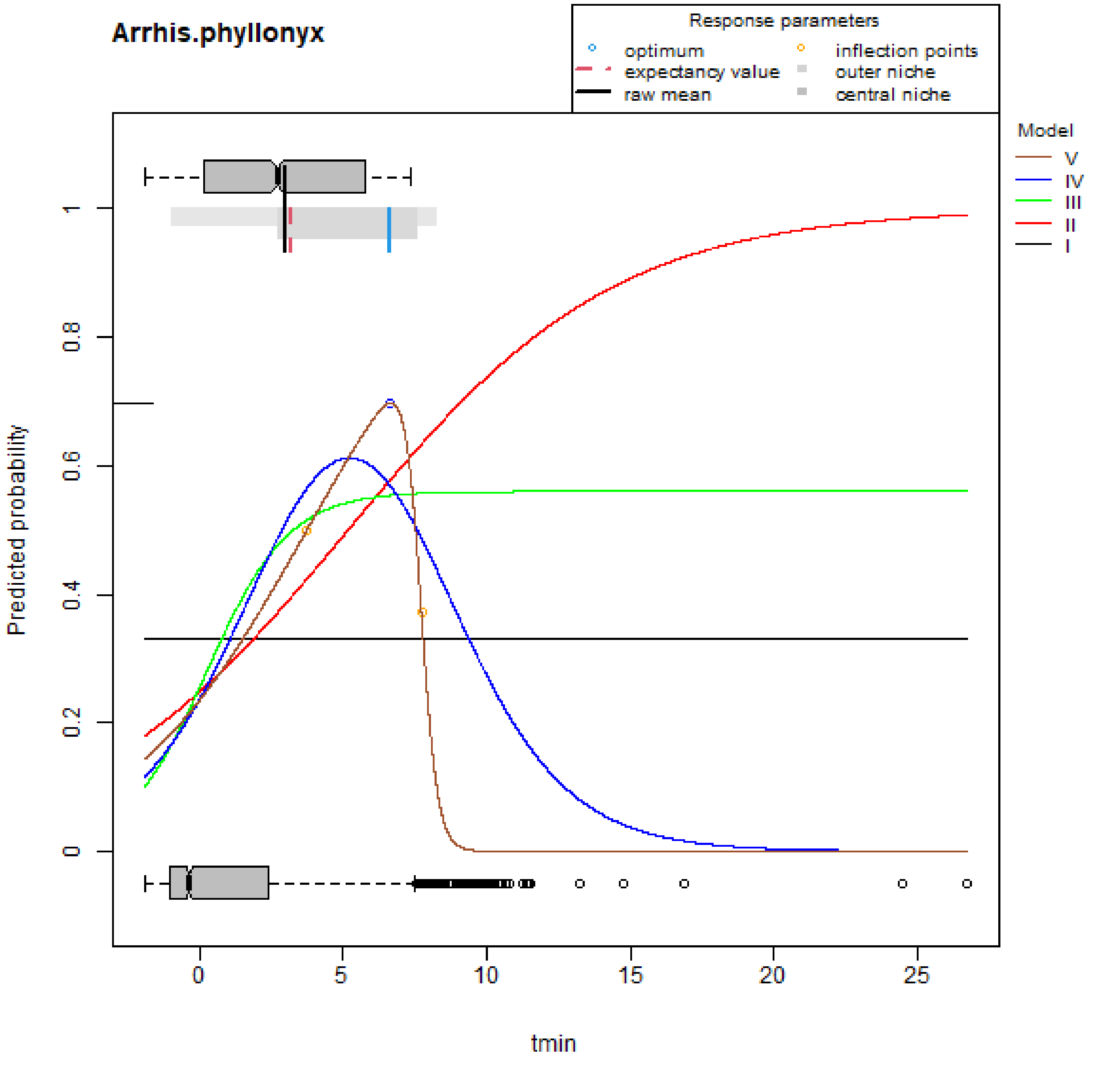

Arrhis.phyllonyx
Response parameters
optimum
expectancy value
raw mean
inflection points
outer niche
central niche
Model
V
IV
III
II
I
Predicted probability
0
0.2
0.4
0.6
0.8
1
0
5
10
15
20
25
tmin

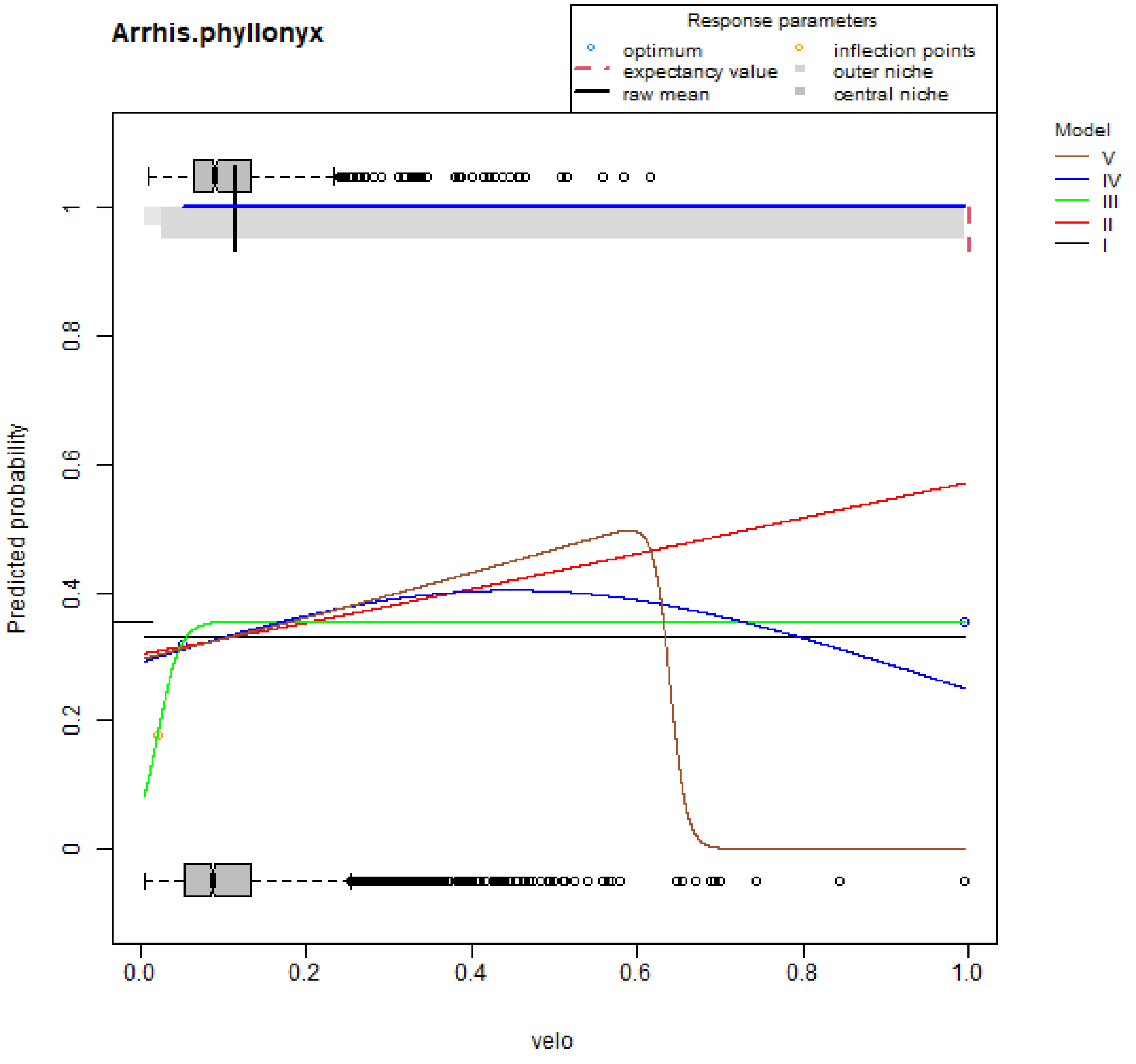

Arrhis.phyllonyx
Response parameters
optimum
expectancy value
raw mean
inflection points
outer niche
central niche
Model
V
IV
III
II
I
Predicted probability
0
0.2
0.4
0.6
0.8
1
0.0
0.2
0.4
0.6
0.8
1.0
velo

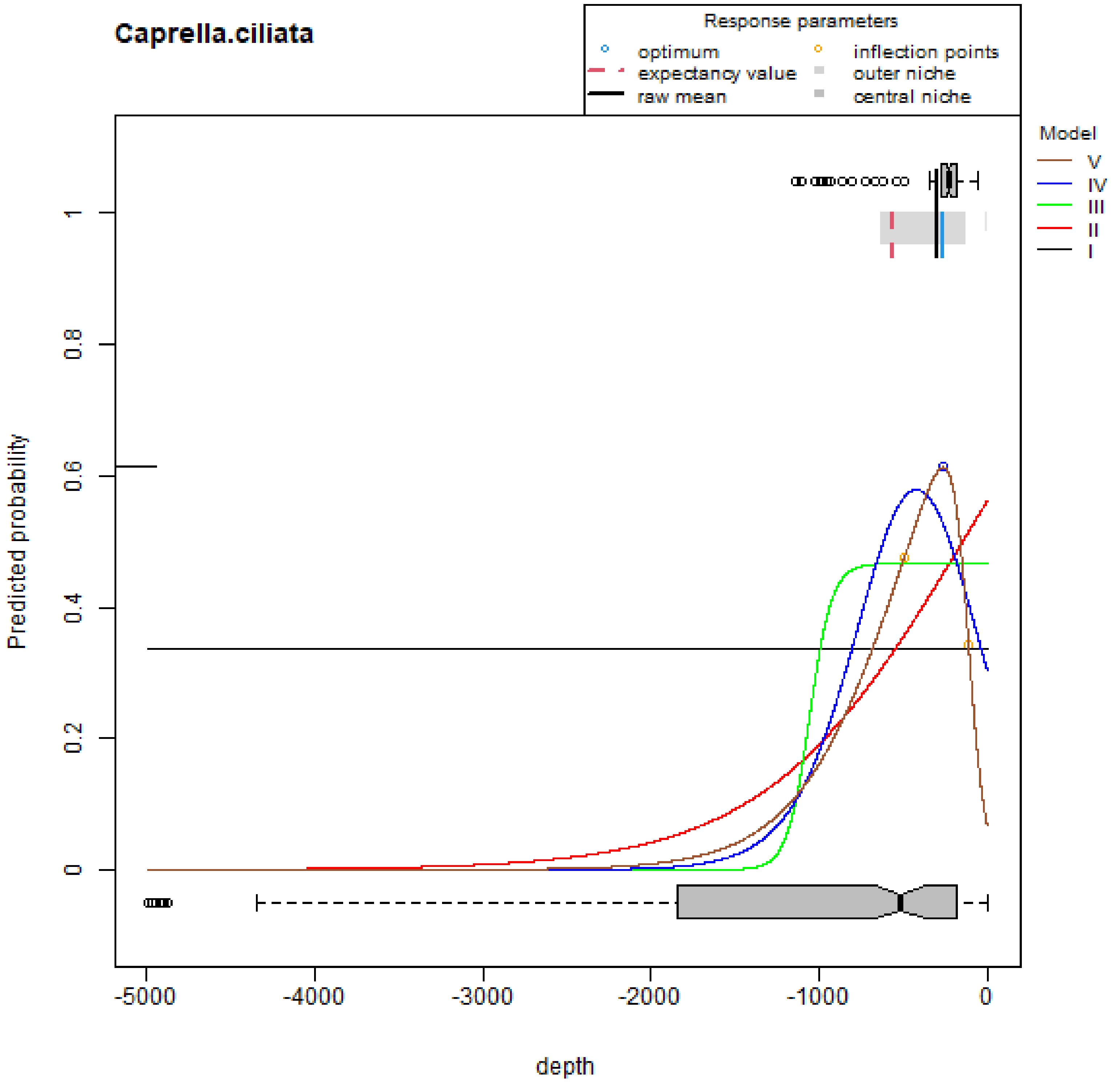

Caprella.ciliata
Response parameters
optimum
expectancy value
raw mean
inflection points
outer niche
central niche
Model
V
IV
III
II
I
Predicted probability
0
0.2
0.4
0.6
0.8
1
-5000
-4000
-3000
-2000
-1000
0
depth

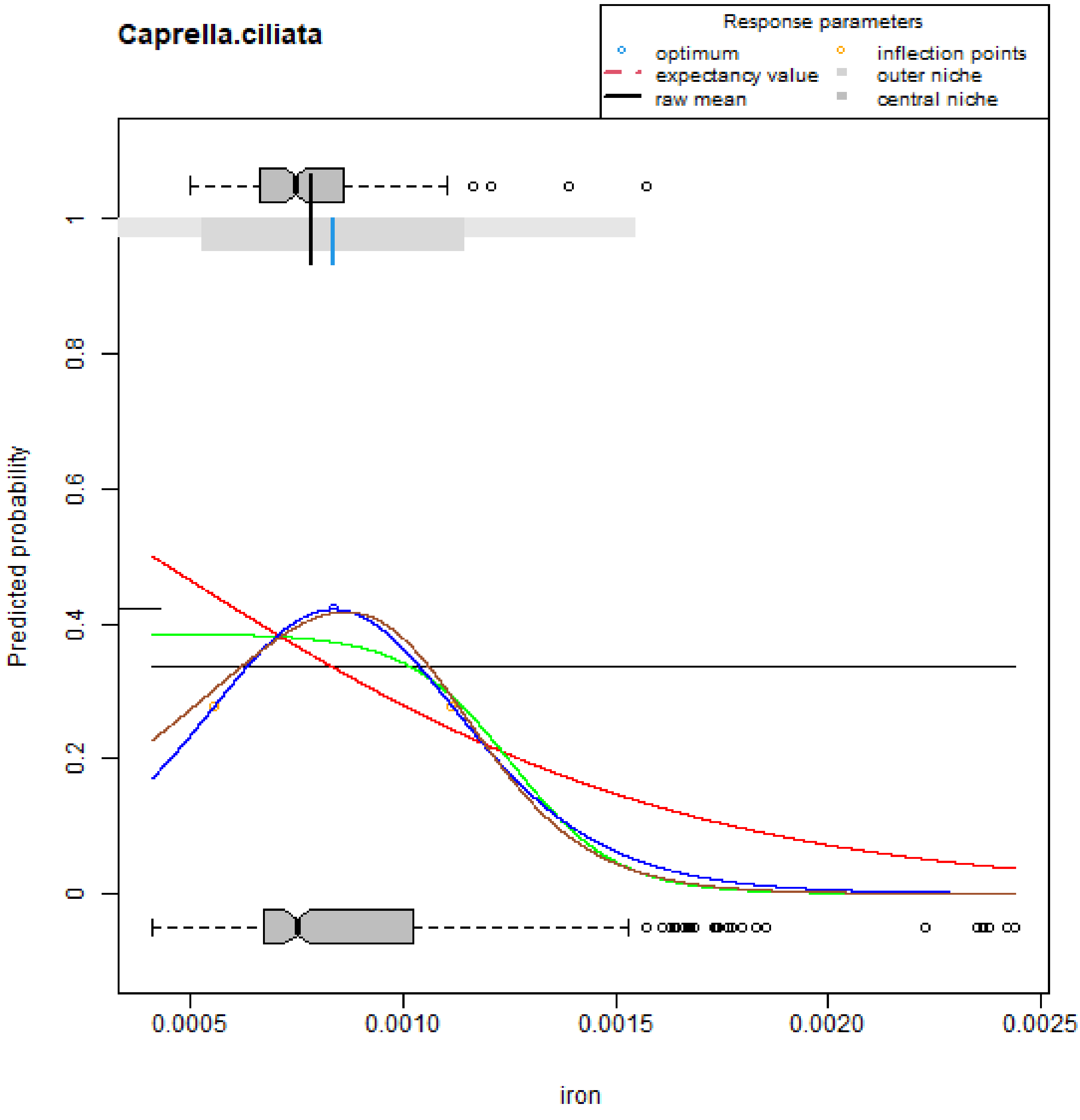

Caprella.ciliata
Response parameters
optimum
expectancy value
raw mean
inflection points
outer niche
central niche
Predicted probability
0
0.2
0.4
0.6
0.8
1
0.0005
0.0010
0.0015
0.0020
0.0025
iron

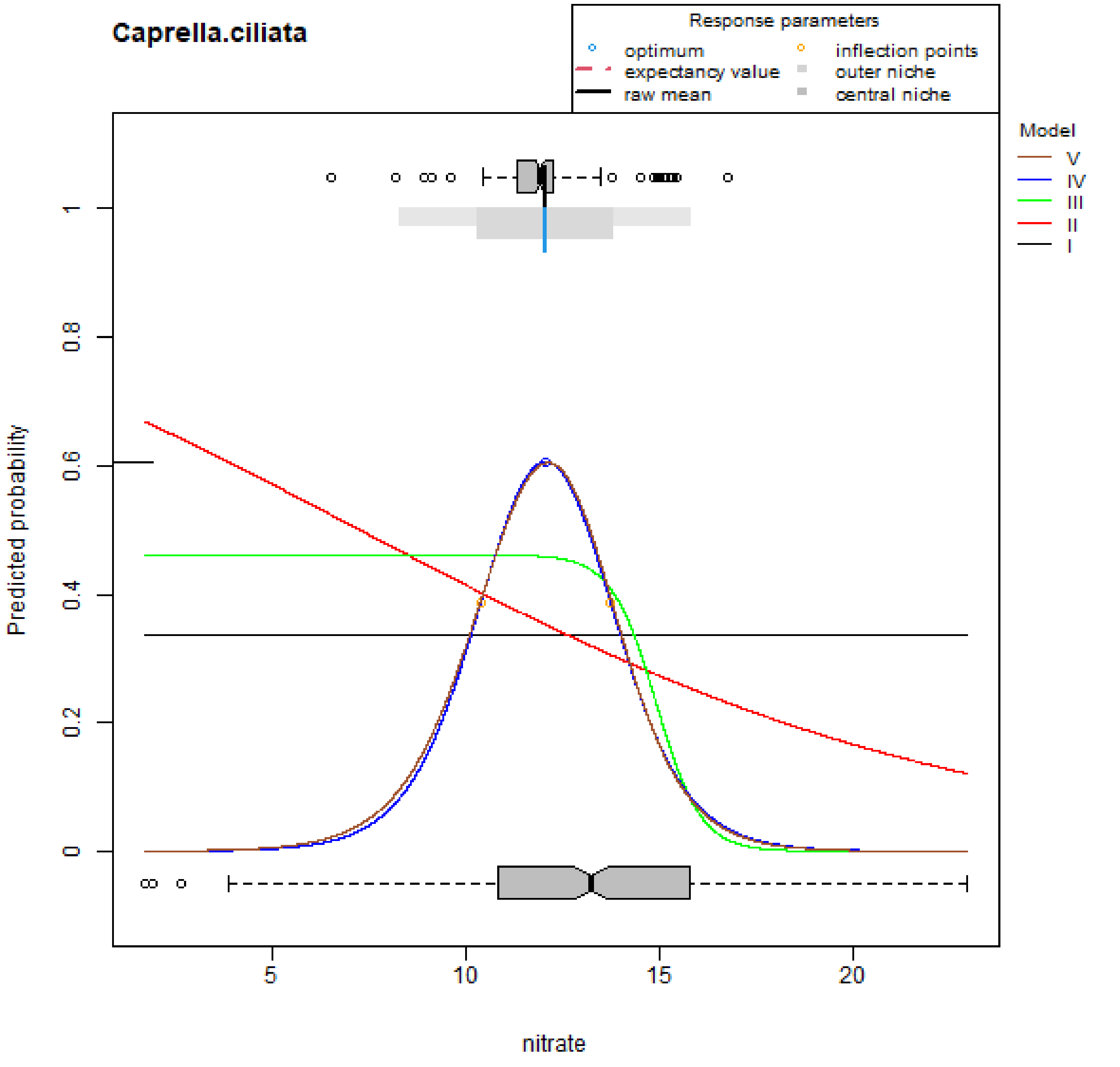

Caprella.ciliata
Response parameters
optimum
expectancy value
raw mean
inflection points
outer niche
central niche
Model
V
IV
III
II
I
Predicted probability
0
0.2
0.4
0.6
0.8
1
5
10
15
20
nitrate

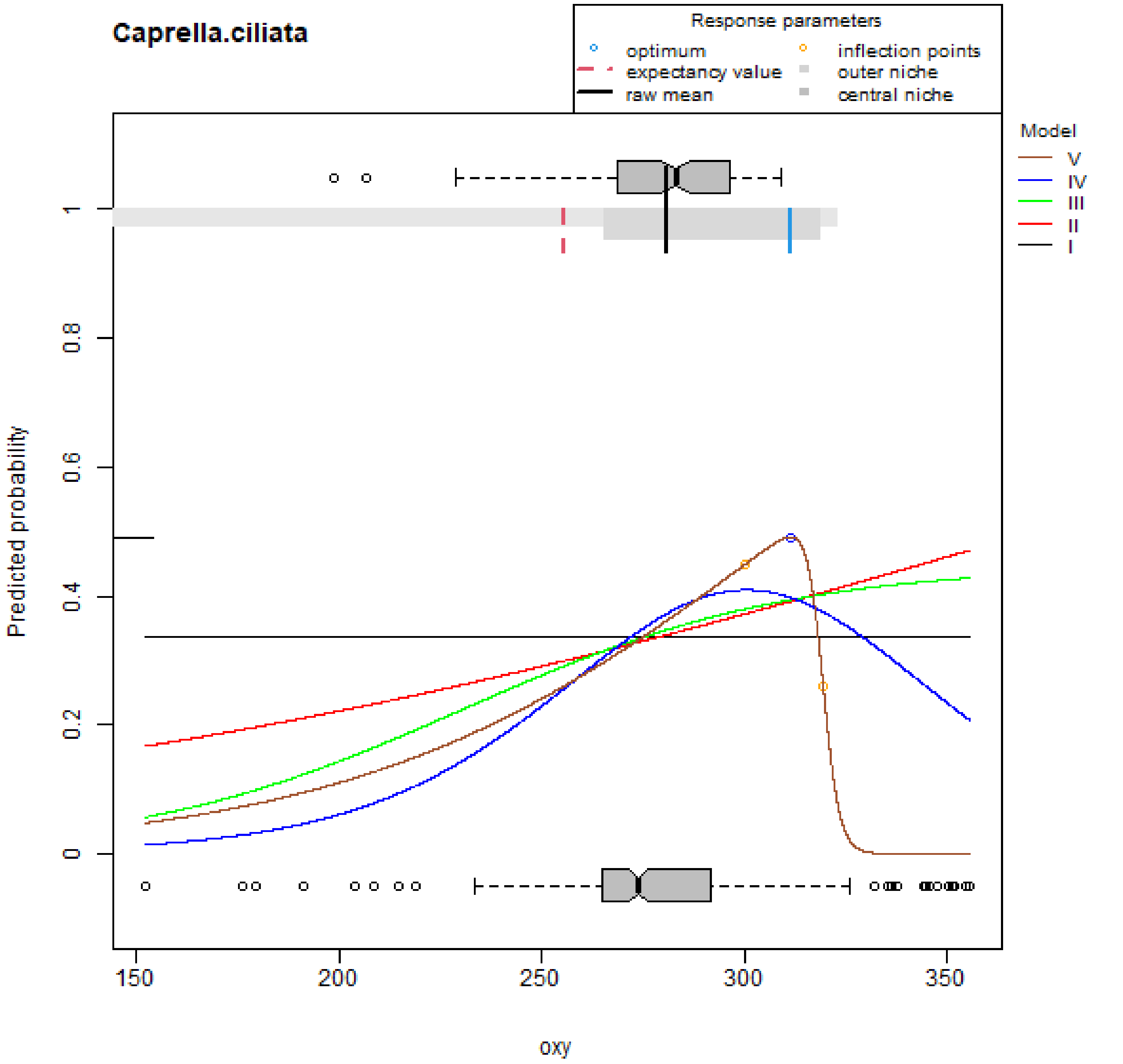
Caprella.ciliata
Response parameters
optimum
expectancy value
raw mean
inflection points
outer niche
central niche
Model
V
IV
III
II
I
Predicted probability
0
0.2
0.4
0.6
0.8
1
150
200
250
300
350
oxy

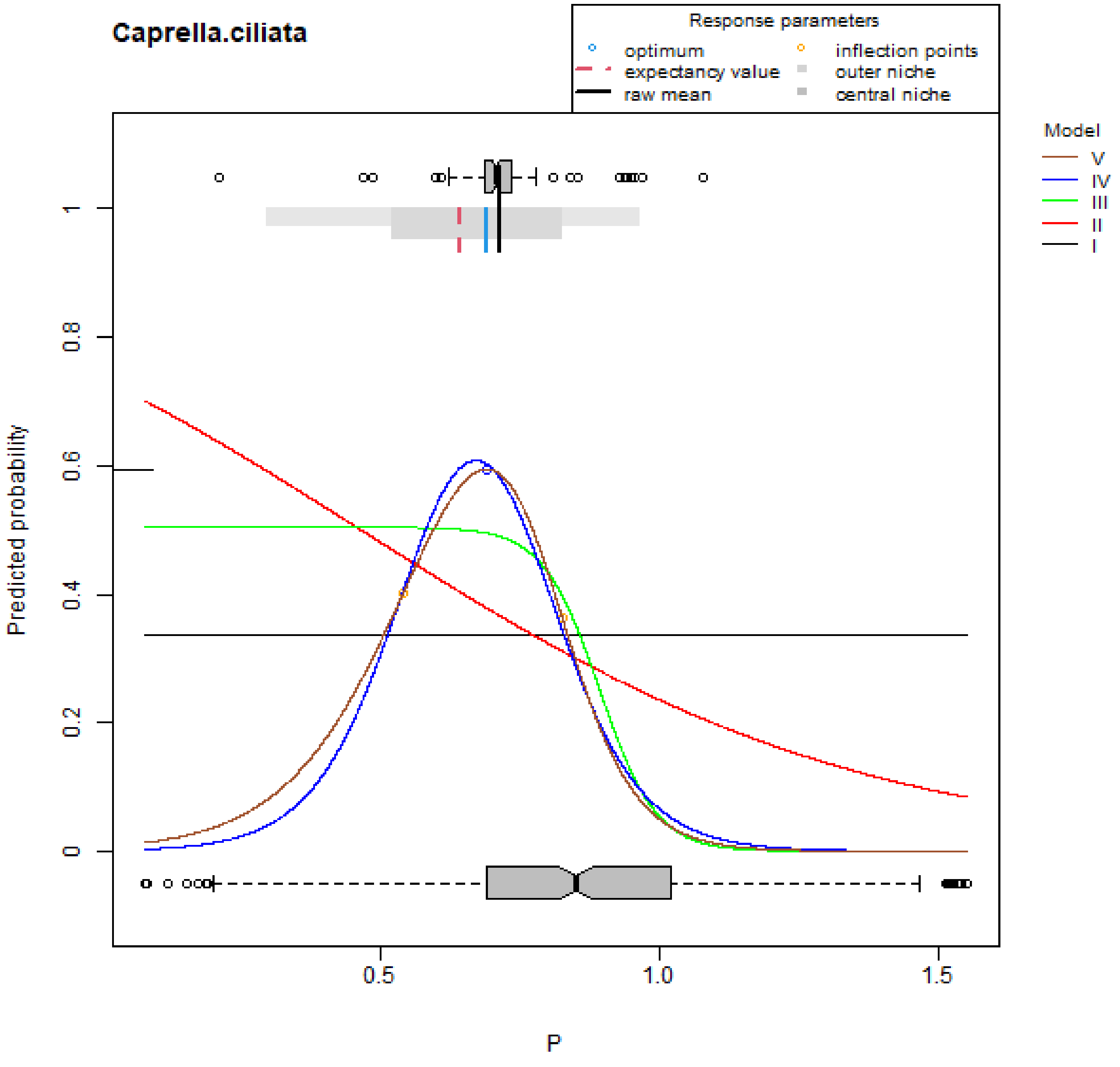

Caprella.ciliata
Response parameters
optimum
expectancy value
raw mean
inflection points
outer niche
central niche
Model
V
IV
III
II
I
Predicted probability
0
0.2
0.4
0.6
0.8
1
0.5
1.0
1.5
P

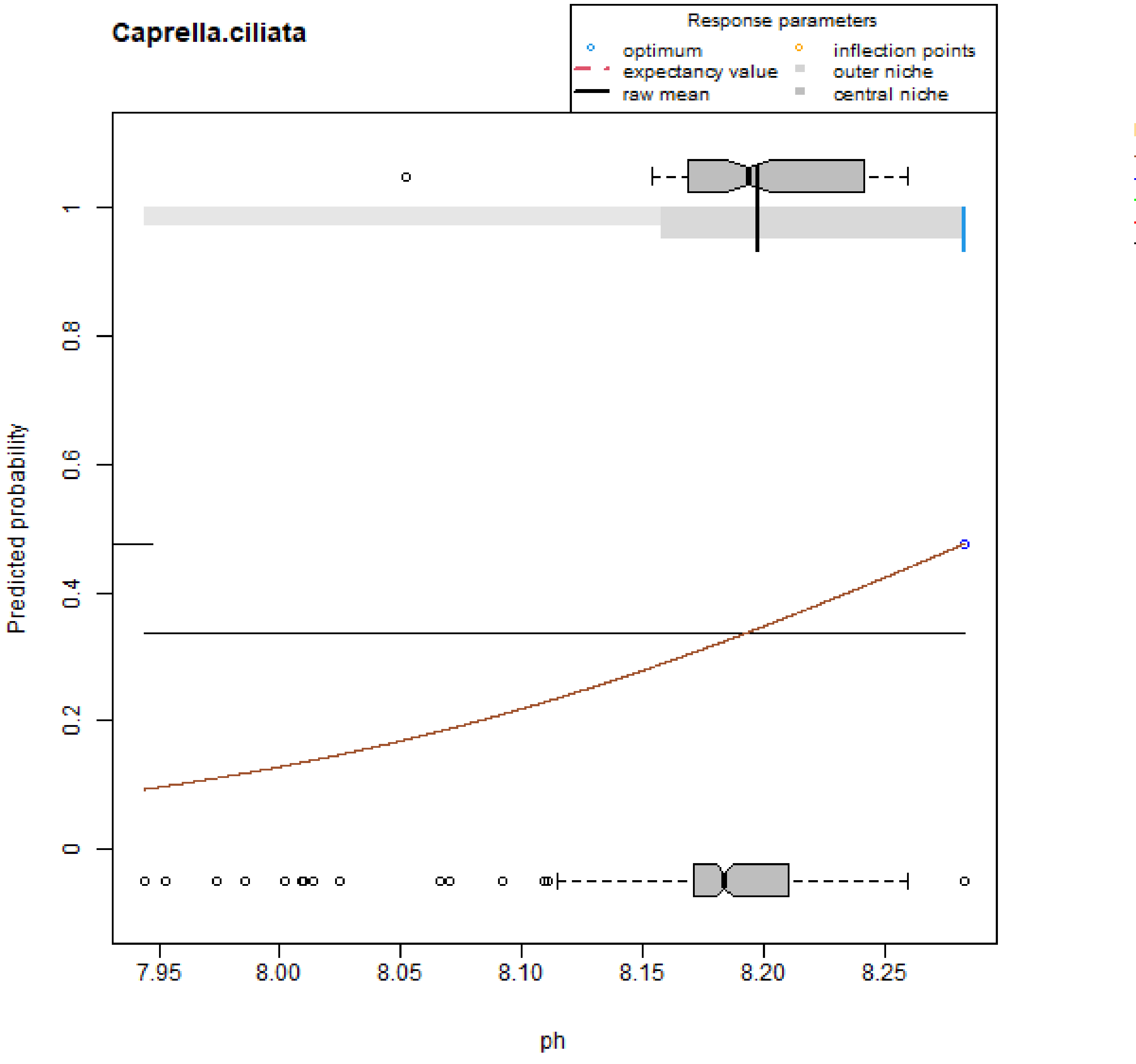

Caprella.ciliata
Response parameters
optimum
expectancy value
raw mean
inflection points
outer niche
central niche
Predicted probability
0
0.2
0.4
0.6
0.8
1
7.95
8.00
8.05
8.10
8.15
8.20
8.25
ph

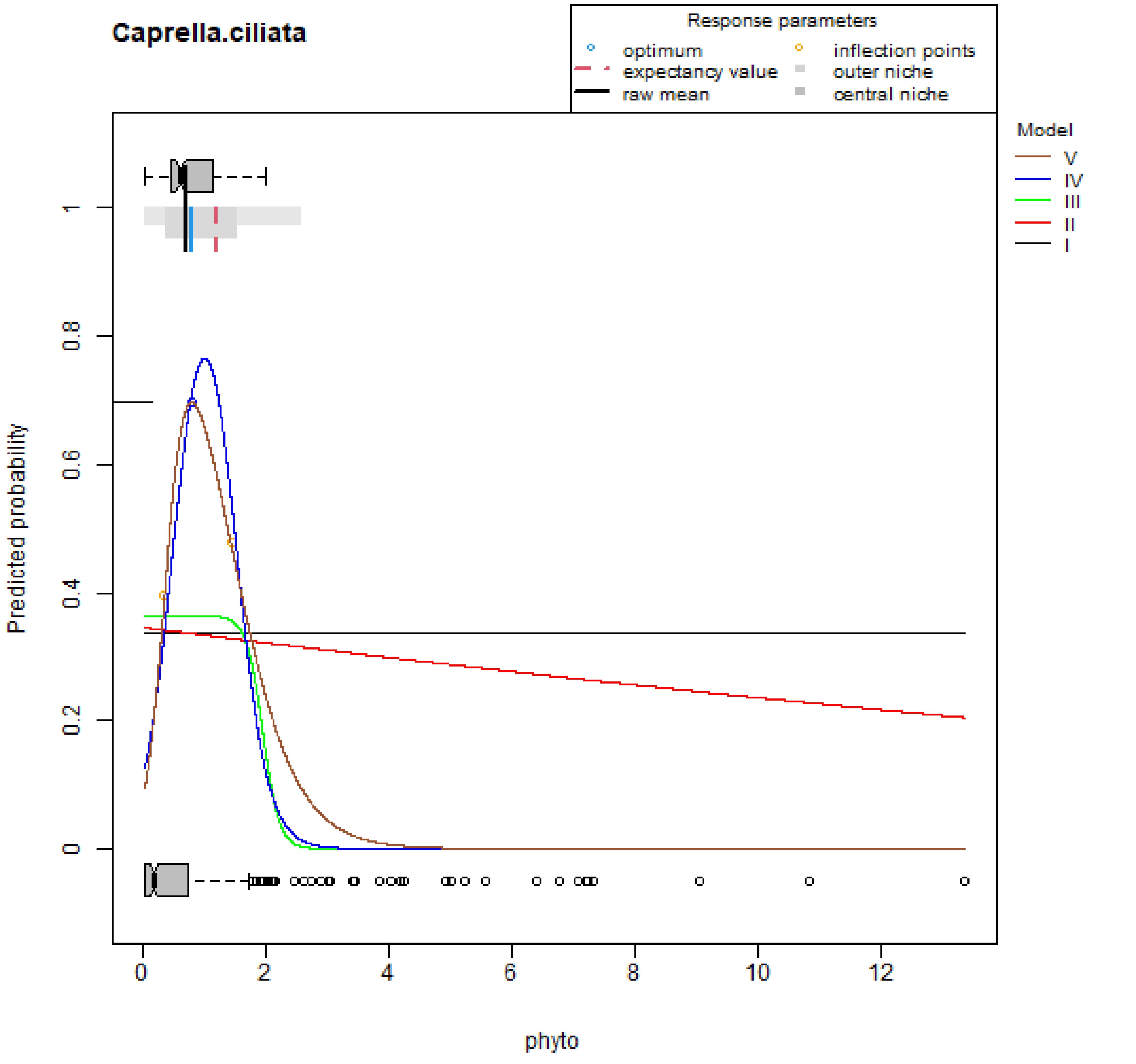

Caprella.ciliata
Response parameters
optimum
expectancy value
raw mean
inflection points
outer niche
central niche
Model
V
IV
III
II
I
Predicted probability
0
0.2
0.4
0.6
0.8
1
0
2
4
6
8
10
12
phyto

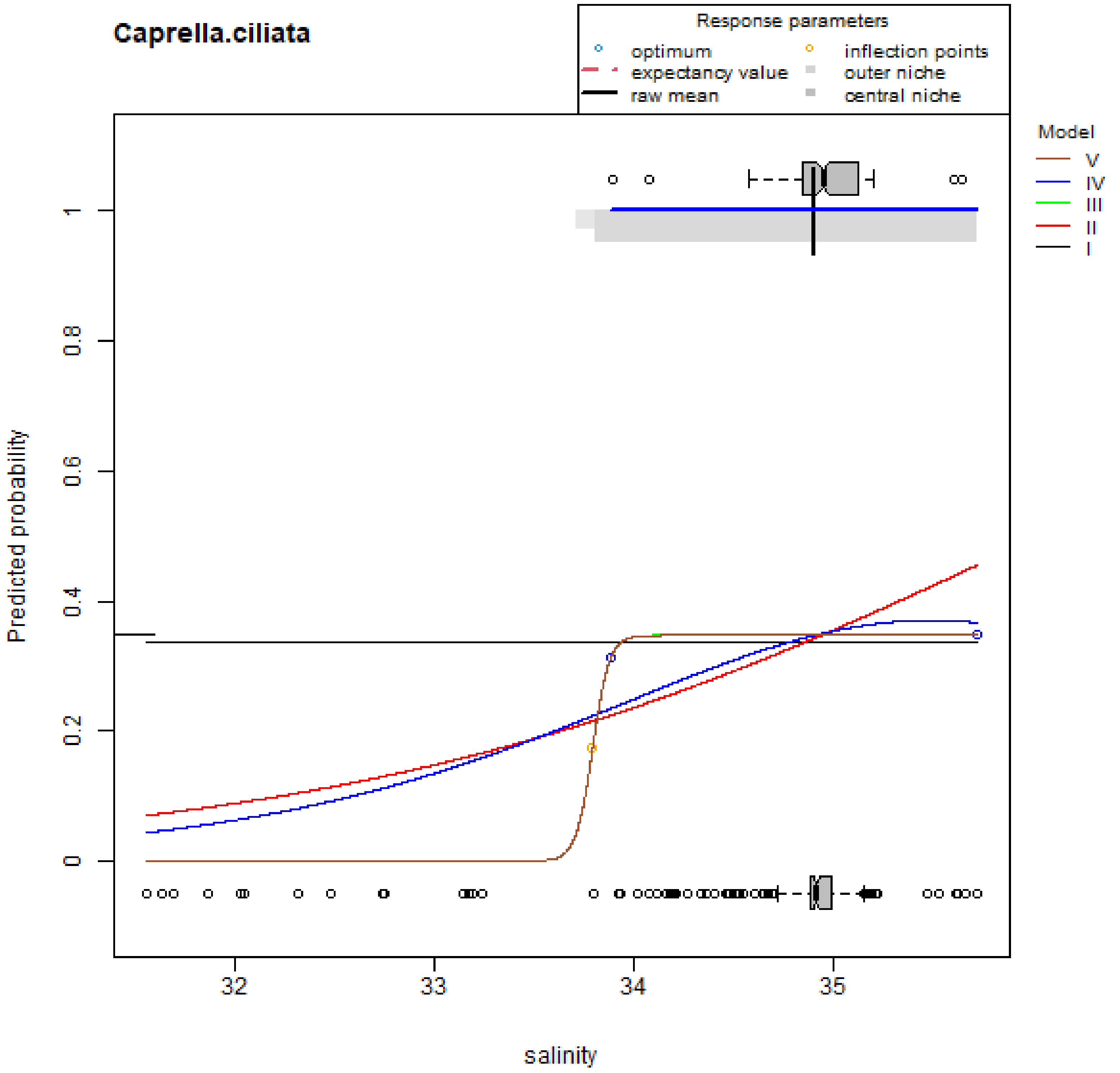

Caprella.ciliata
Response parameters
optimum
expectancy value
raw mean
inflection points
outer niche
central niche
Model
V
IV
III
II
I
Predicted probability
0
0.2
0.4
0.6
0.8
1
32
33
34
35
salinity

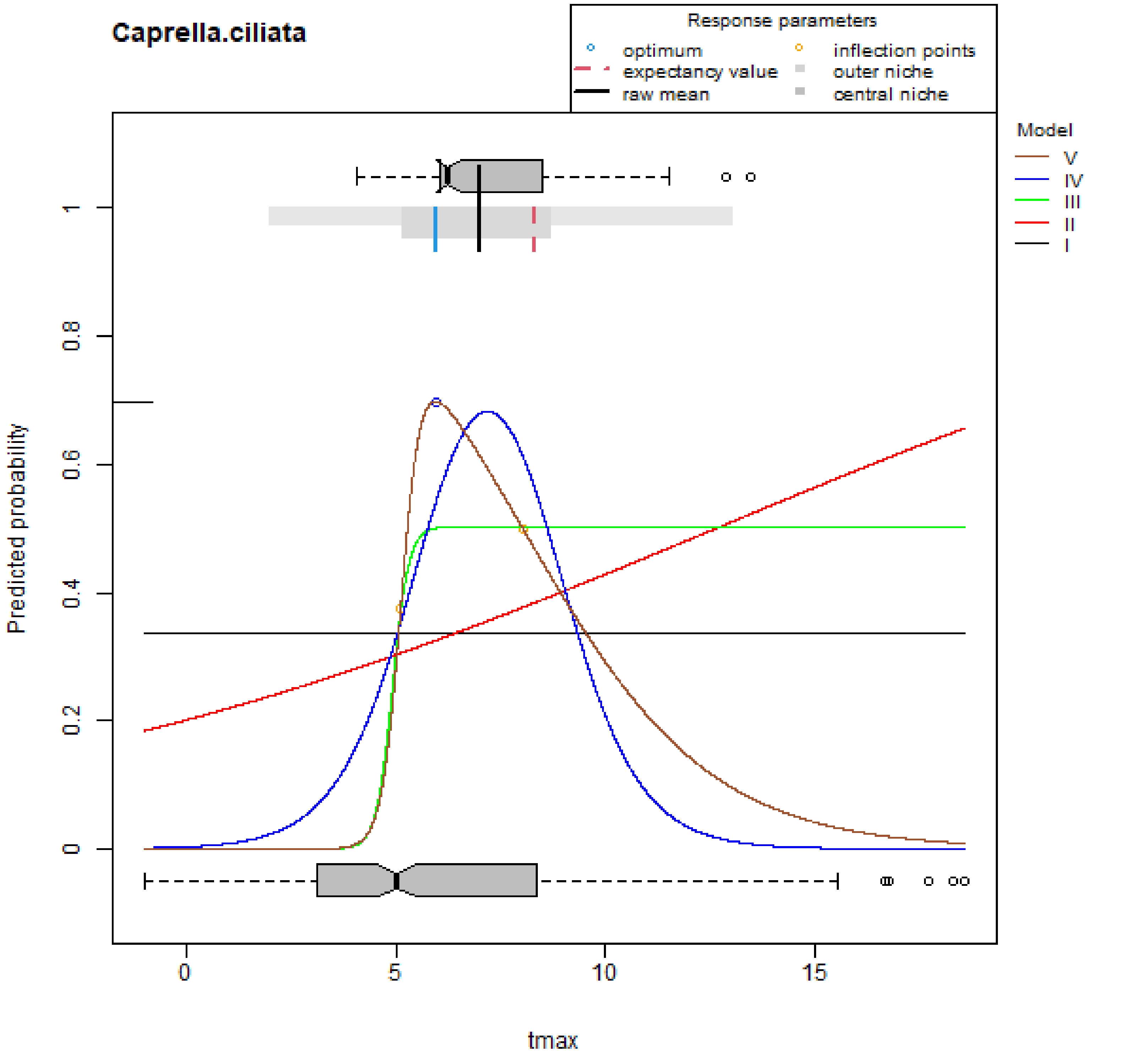
Caprella.ciliata
Response parameters
optimum
expectancy value
raw mean
inflection points
outer niche
central niche
Model
V
IV
III
II
I
Predicted probability
0
0.2
0.4
0.6
0.8
1
0
5
10
15
tmax

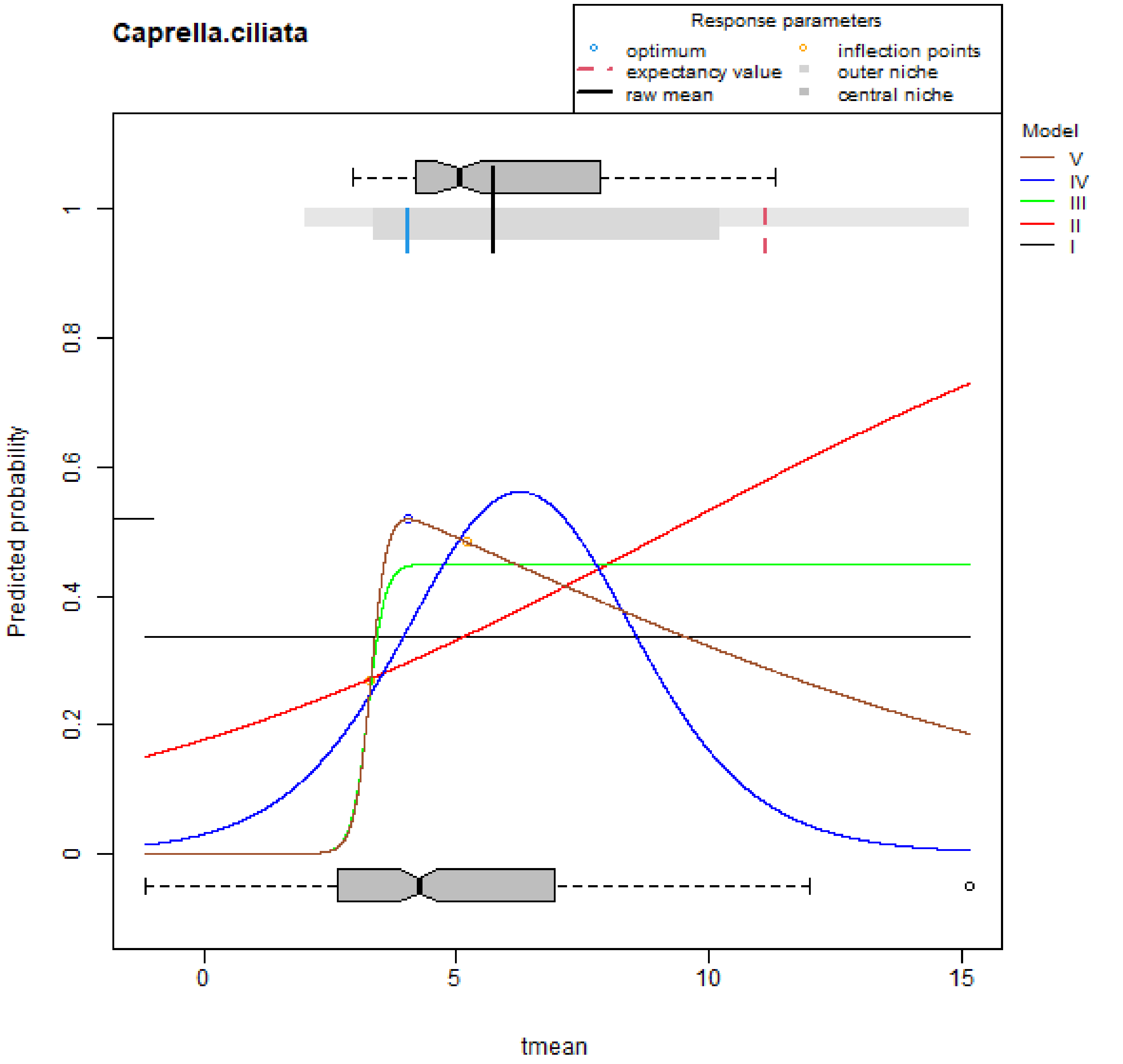
Caprella.ciliata
Response parameters
optimum
expectancy value
raw mean
inflection points
outer niche
central niche
Model
V
IV
III
II
I
Predicted probability
0
0.2
0.4
0.6
0.8
1
0
5
10
15
tmean

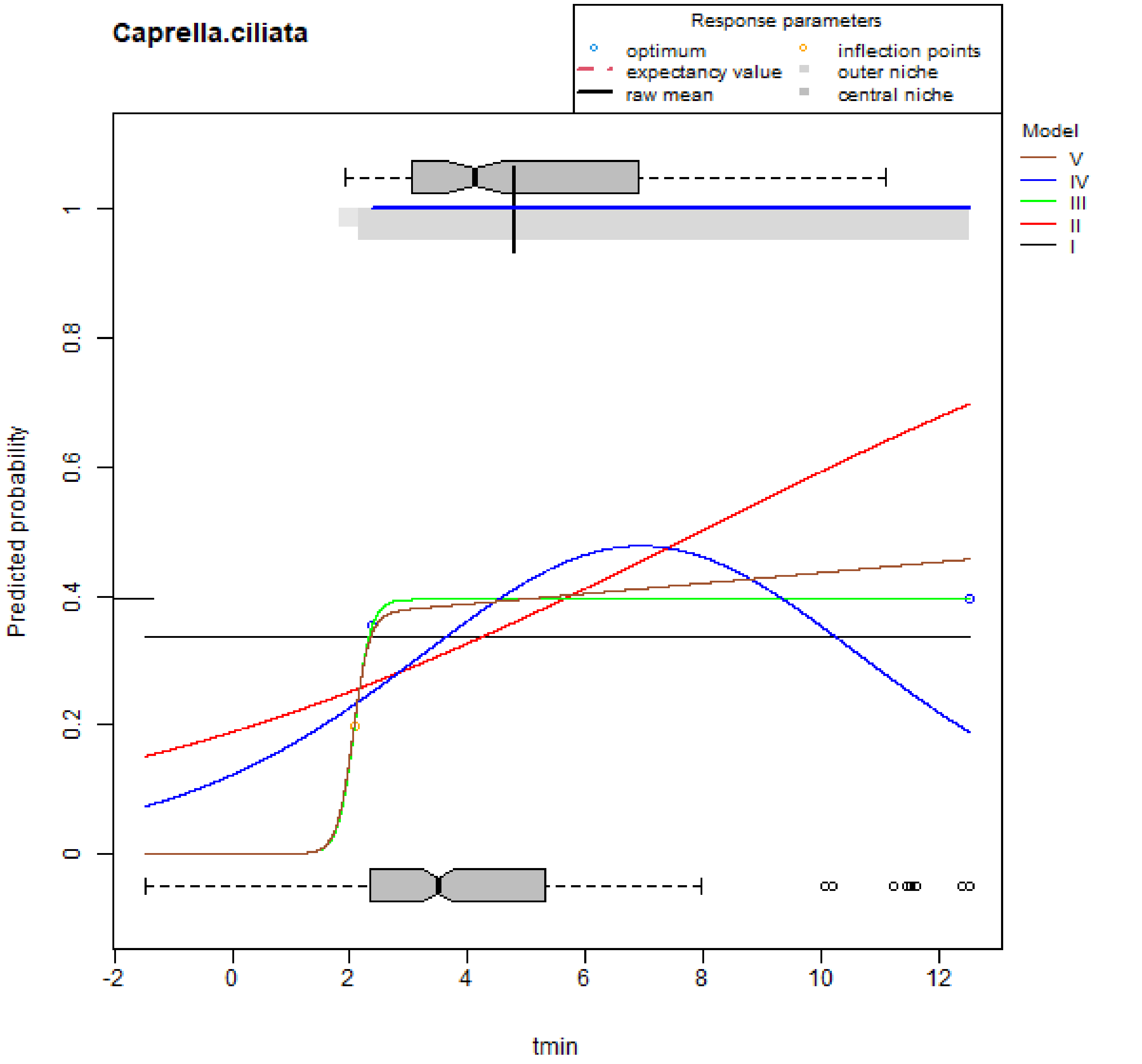
Caprella.ciliata
Response parameters
optimum
expectancy value
raw mean
inflection points
outer niche
central niche
Model
V
IV
III
II
I
Predicted probability
0
0.2
0.4
0.6
0.8
1
-2
0
2
4
6
8
10
12
tmin

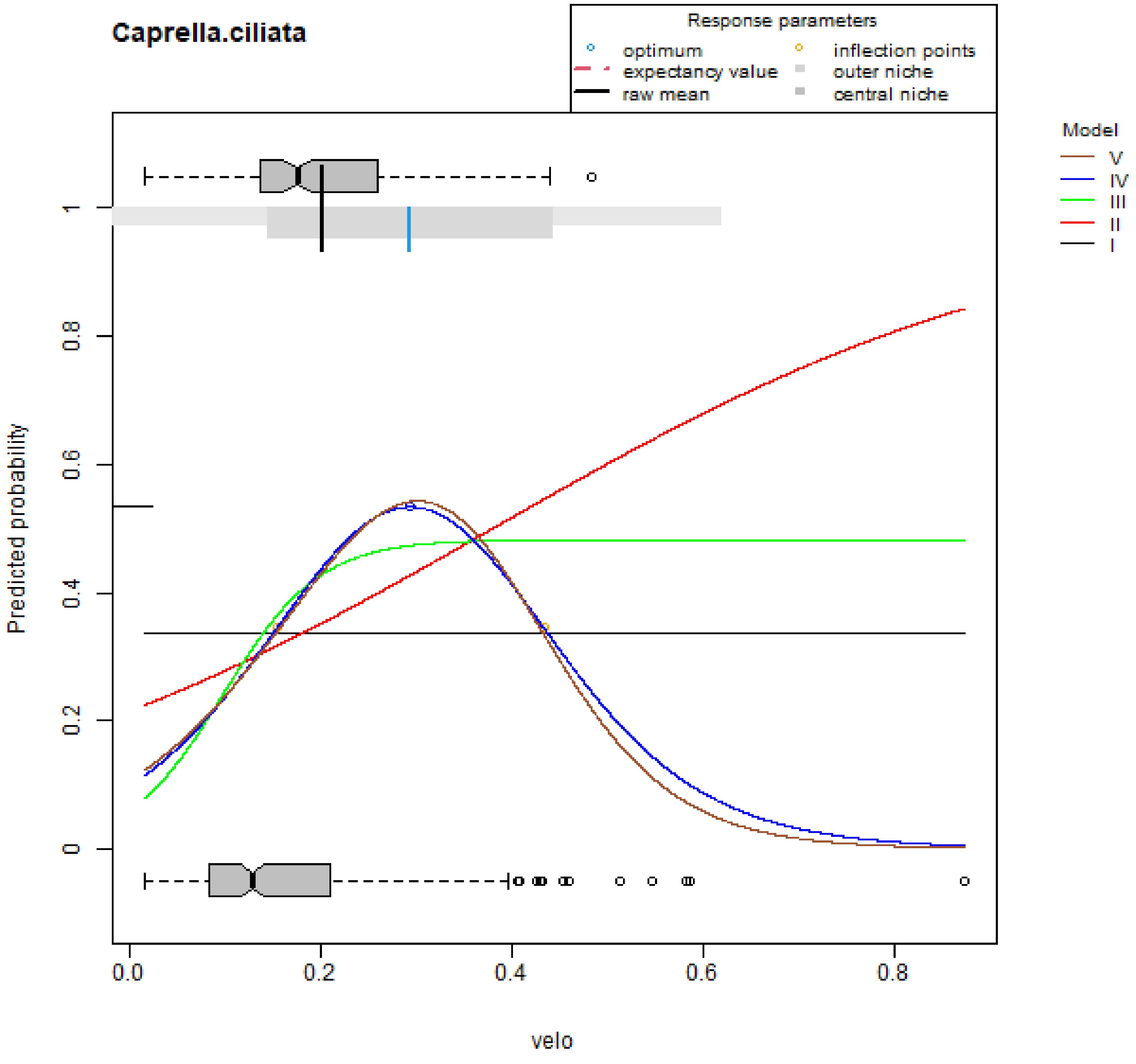
Caprella.ciliata
Response parameters
optimum
expectancy value
raw mean
inflection points
outer niche
central niche
Model
V
IV
III
II
I
Predicted probability
0
0.2
0.4
0.6
0.8
1
0.0
0.2
0.4
0.6
0.8
velo

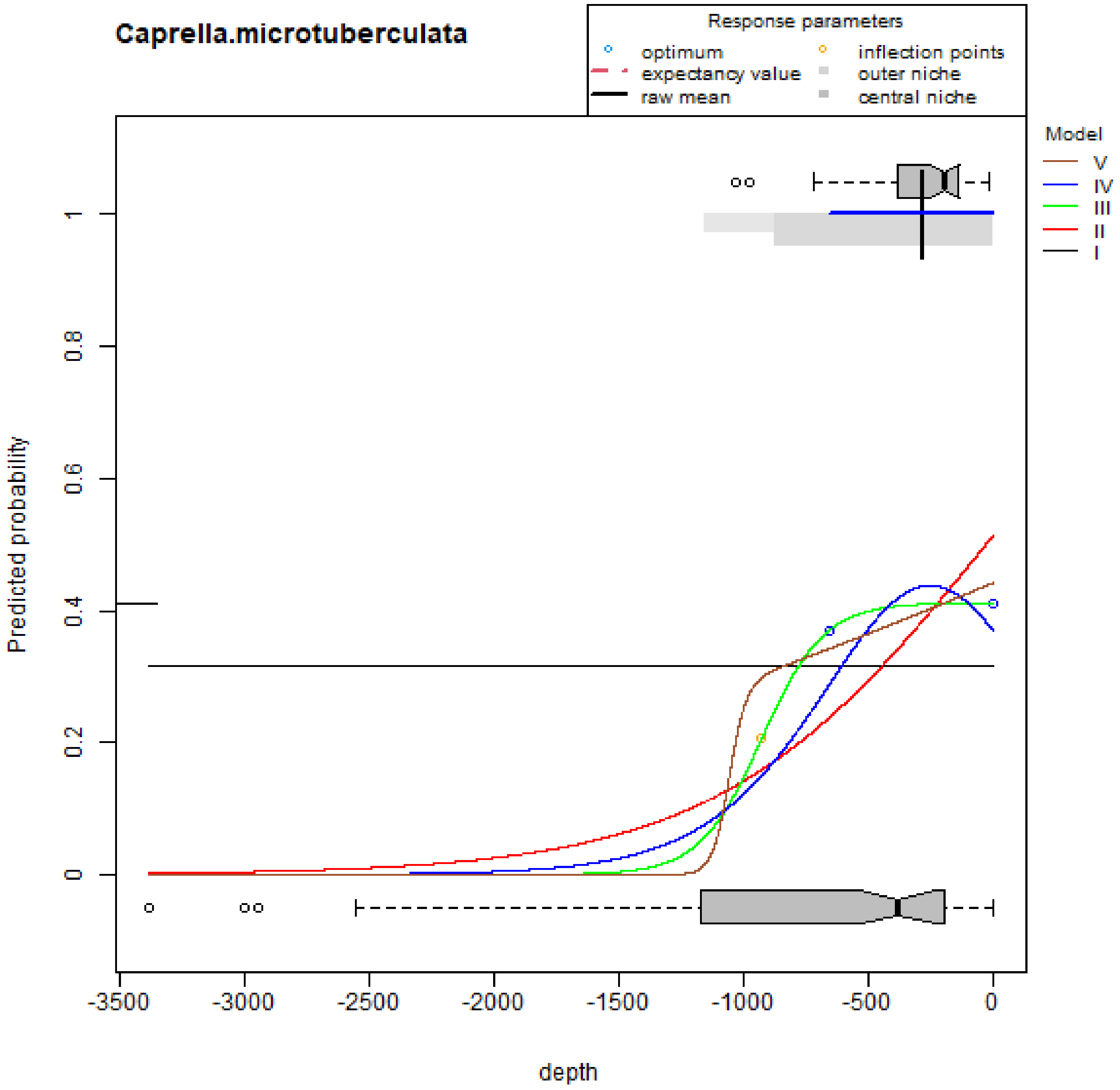

Caprella.microtuberculata
Response parameters
optimum
expectancy value
raw mean
inflection points
outer niche
central niche
Model
V
IV
III
II
I
Predicted probability
0
0.2
0.4
0.6
0.8
1
-3500
-3000
-2500
-2000
-1500
-1000
-500
0
depth

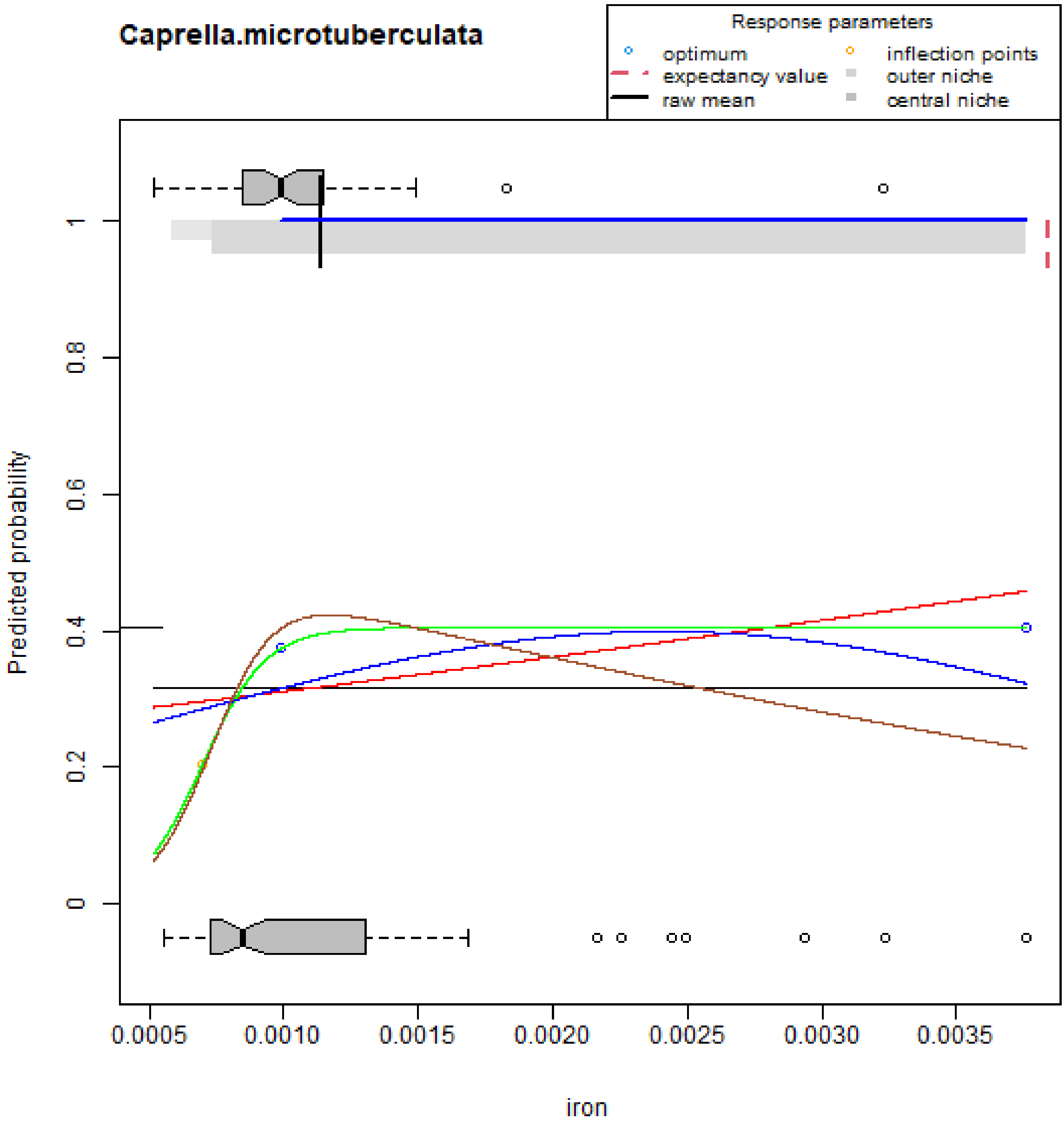

Caprella.microtuberculata
Response parameters
optimum
expectancy value
raw mean
inflection points
outer niche
central niche
Predicted probability
0
0.2
0.4
0.6
0.8
1
0.0005
0.0010
0.0015
0.0020
0.0025
0.0030
0.0035
iron

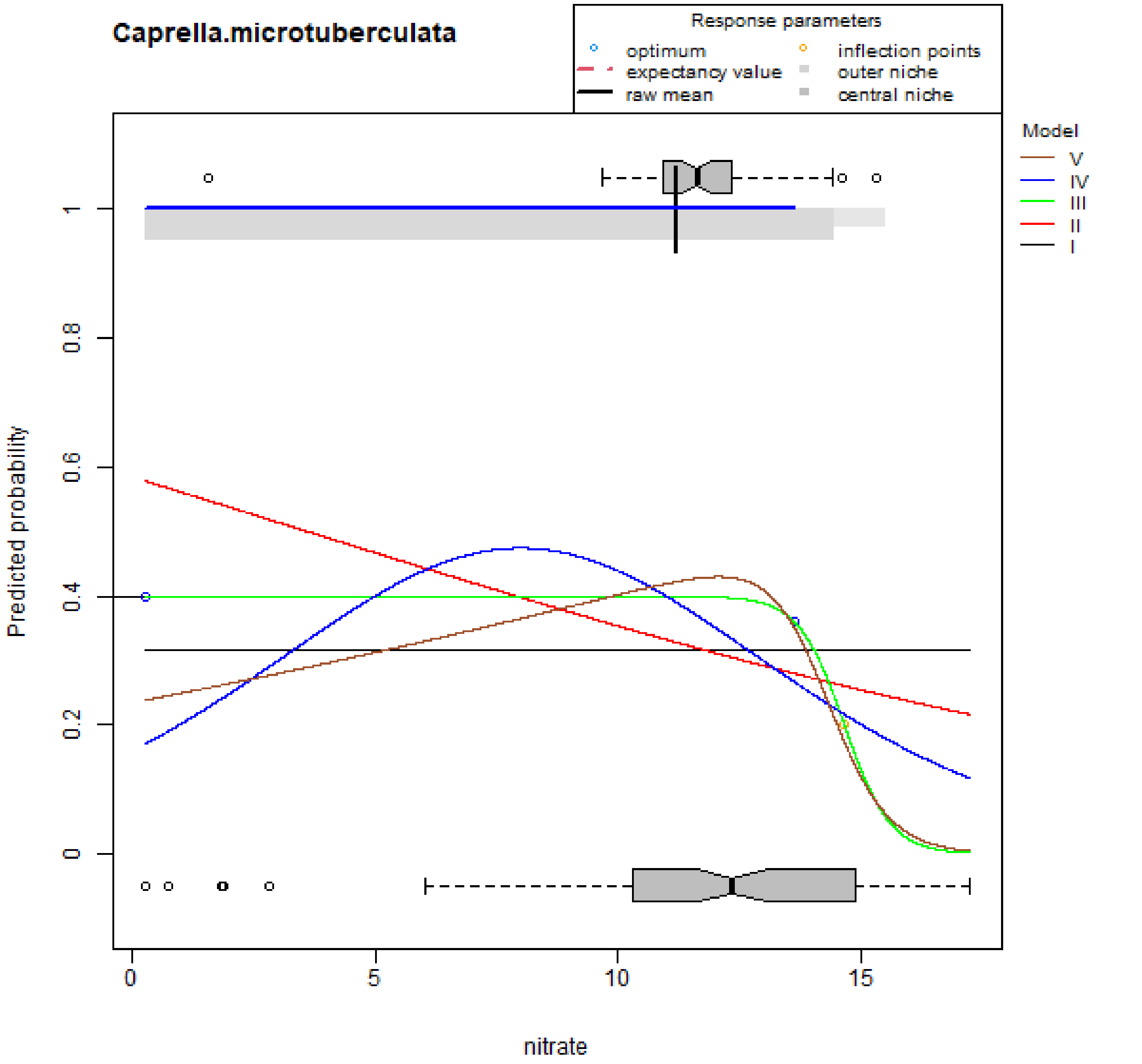

Caprella.microtuberculata
Response parameters
optimum
expectancy value
raw mean
inflection points
outer niche
central niche
Model
V
IV
III
II
I
Predicted probability
0
0.2
0.4
0.6
0.8
1
0
5
10
15
nitrate

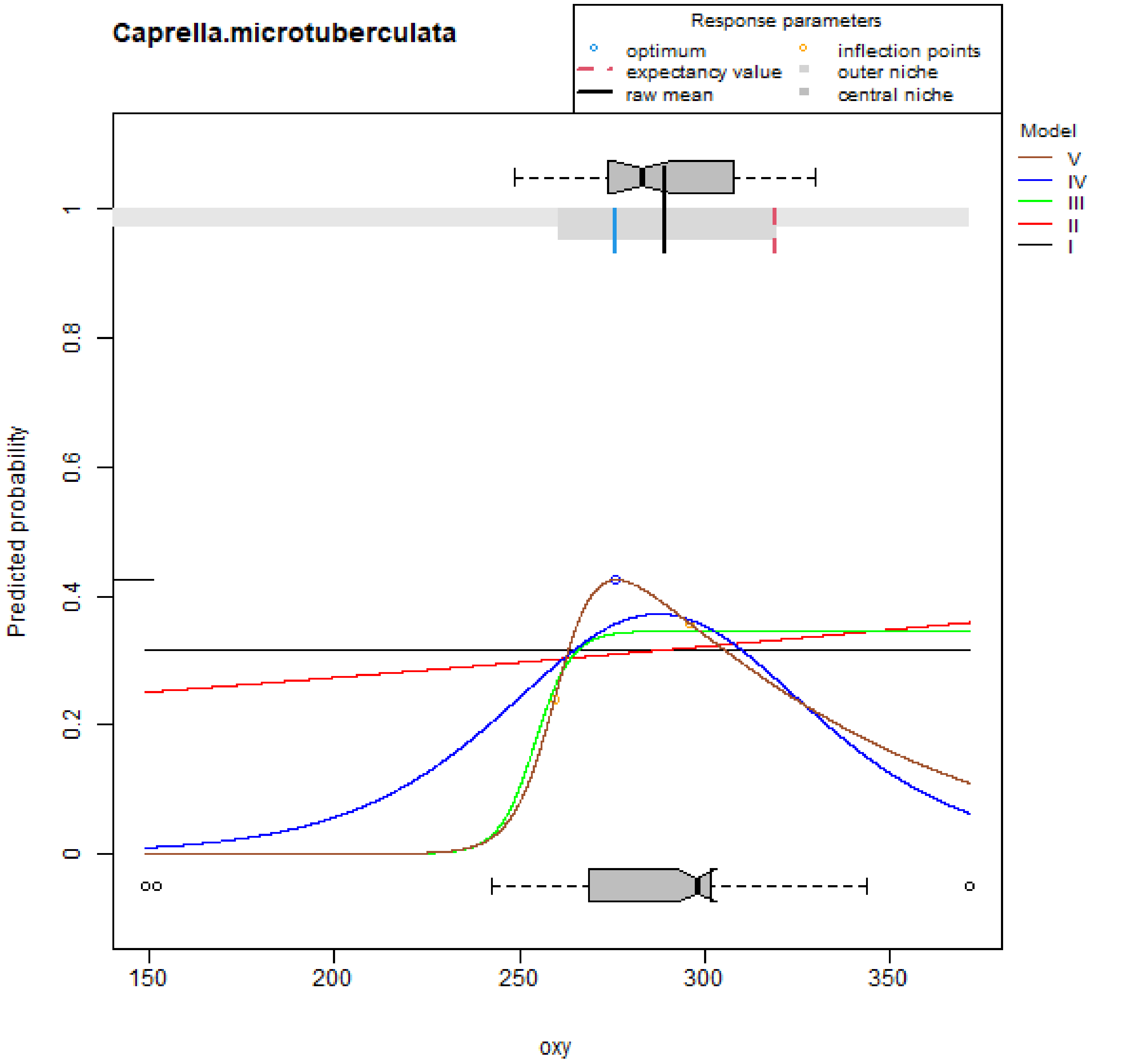
Caprella.microtuberculata
Response parameters
optimum
expectancy value
raw mean
inflection points
outer niche
central niche
Model
V
IV
III
II
I
Predicted probability
0
0.2
0.4
0.6
0.8
1
150
200
250
300
350
oxy

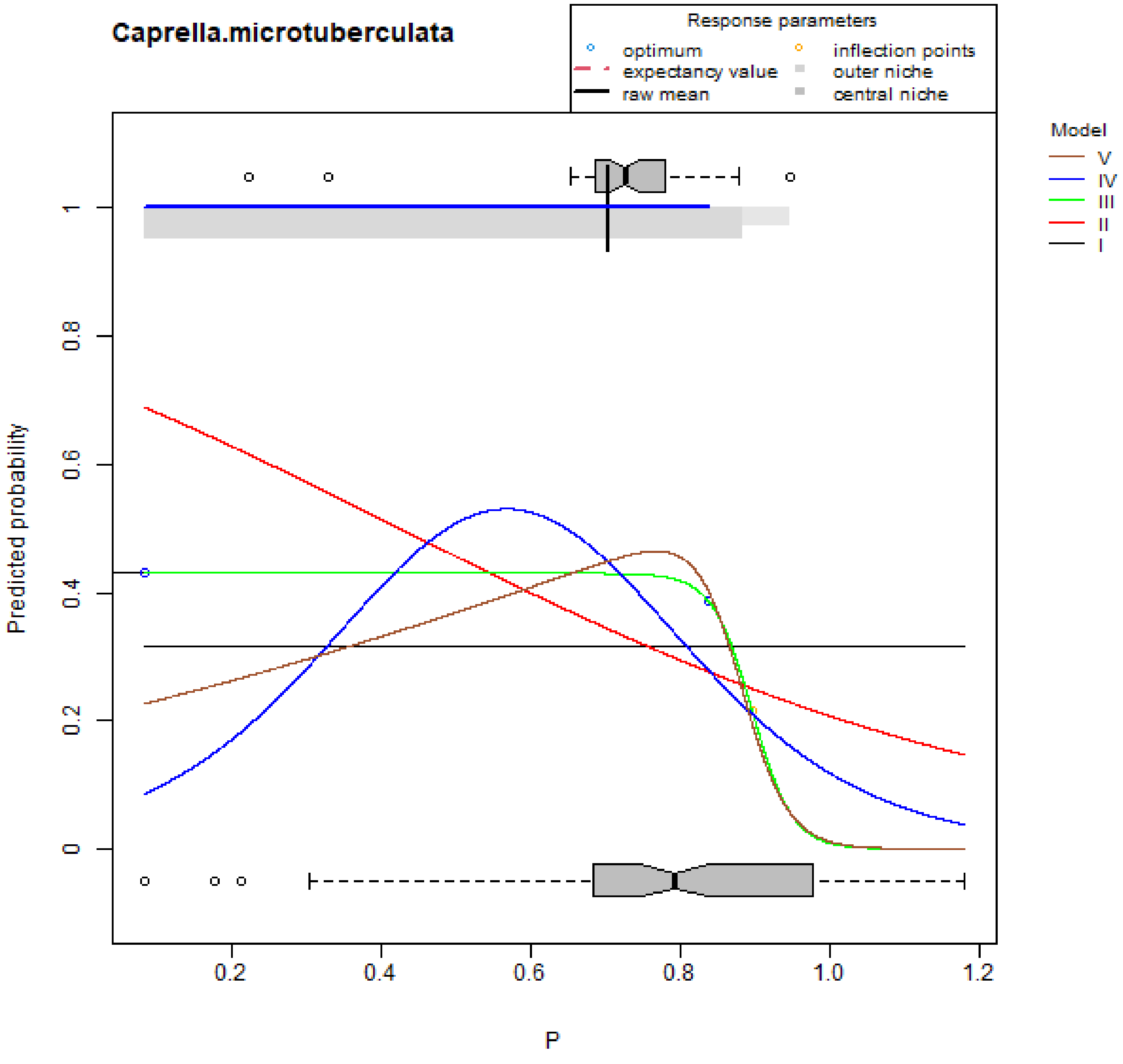

Caprella.microtuberculata
Response parameters
optimum
expectancy value
raw mean
inflection points
outer niche
central niche
Model
V
IV
III
II
I
Predicted probability
0
0.2
0.4
0.6
0.8
1
0.2
0.4
0.6
0.8
1.0
1.2
P

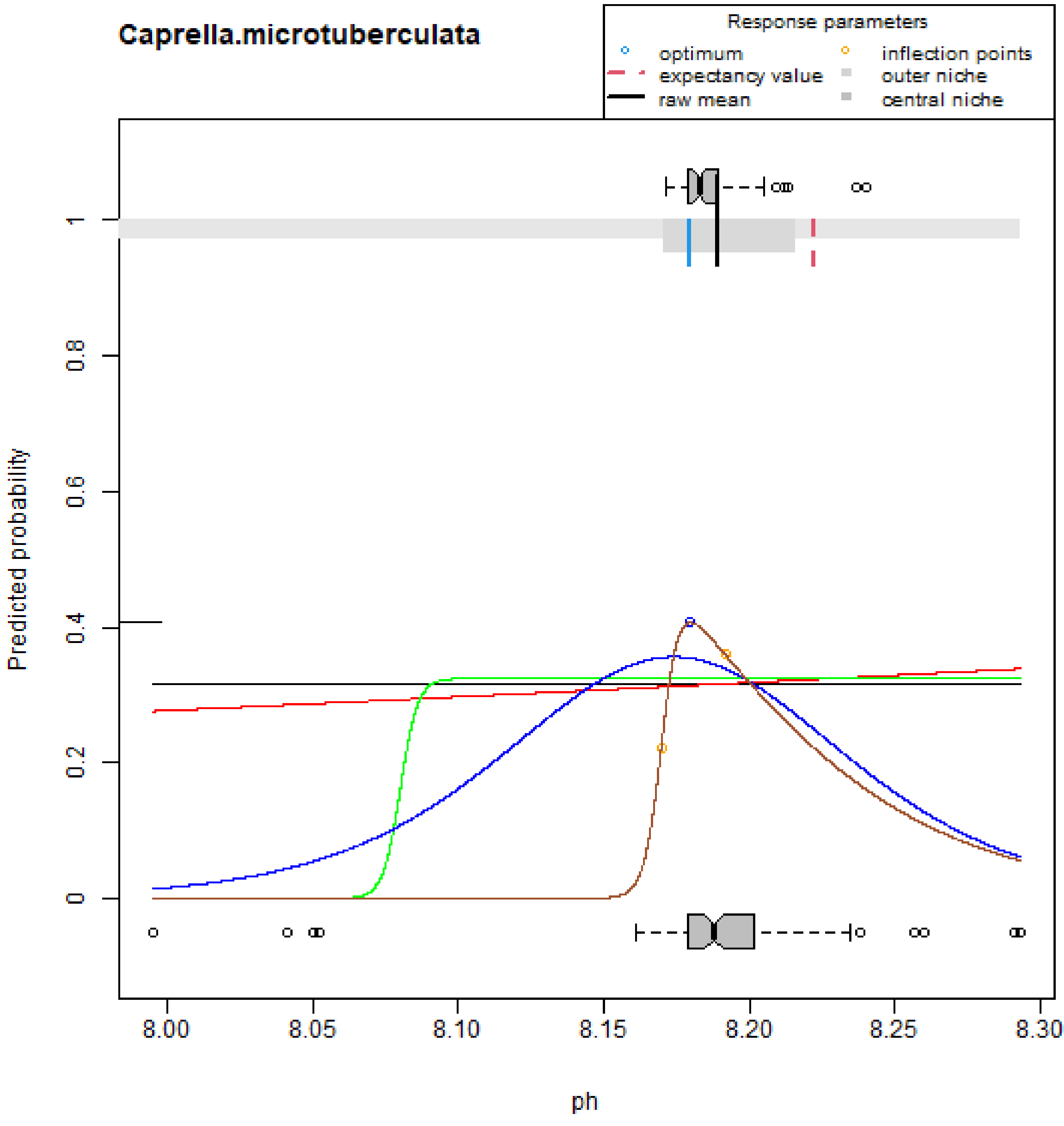

Caprella.microtuberculata
Response parameters
optimum
expectancy value
raw mean
inflection points
outer niche
central niche
Predicted probability
0
0.2
0.4
0.6
0.8
1
8.00
8.05
8.10
8.15
8.20
8.25
8.30
ph

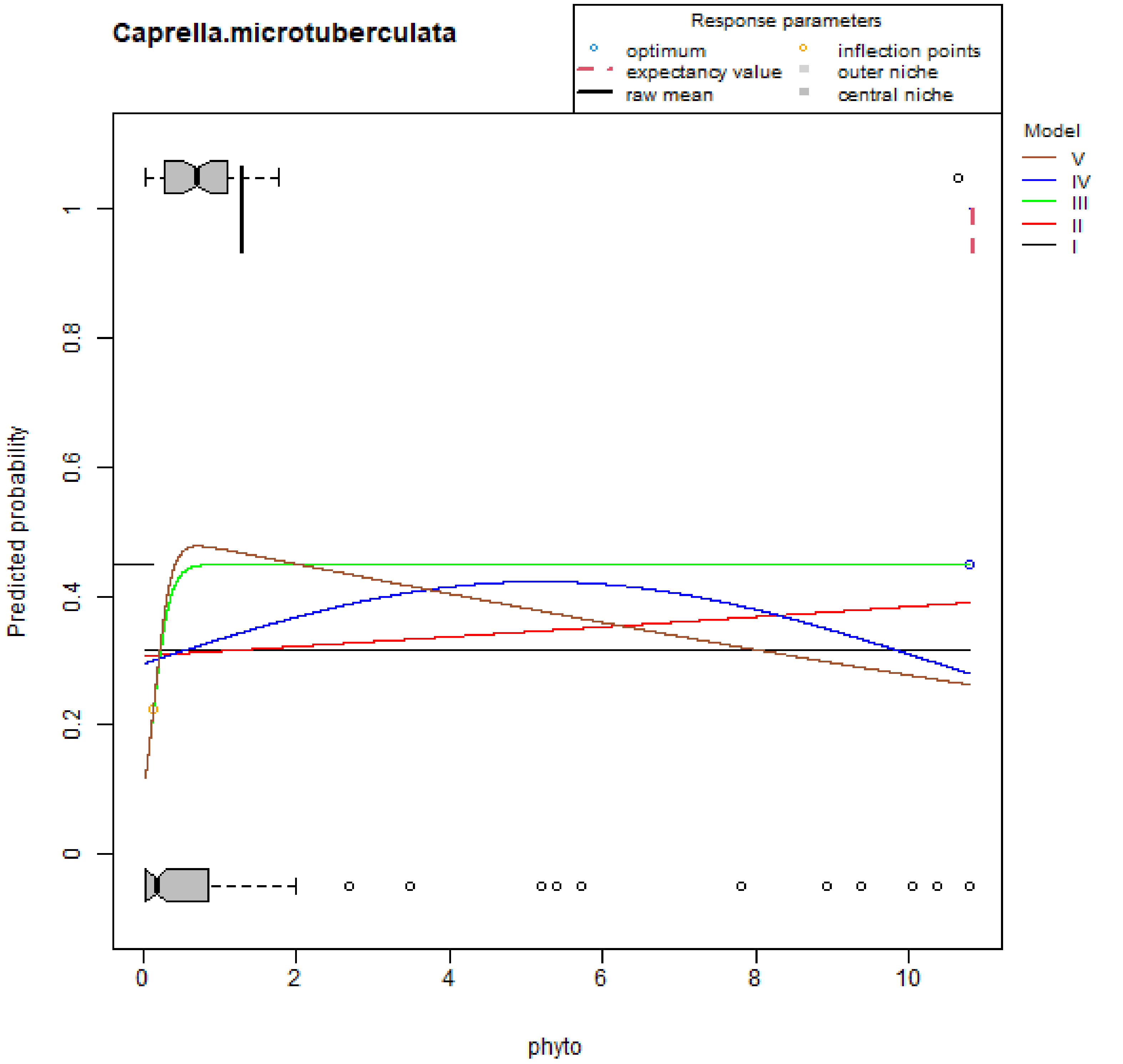
Caprella.microtuberculata
Response parameters
optimum
expectancy value
raw mean
inflection points
outer niche
central niche
Model
V
IV
III
II
I
Predicted probability
0
0.2
0.4
0.6
0.8
1
0
2
4
6
8
10
phyto

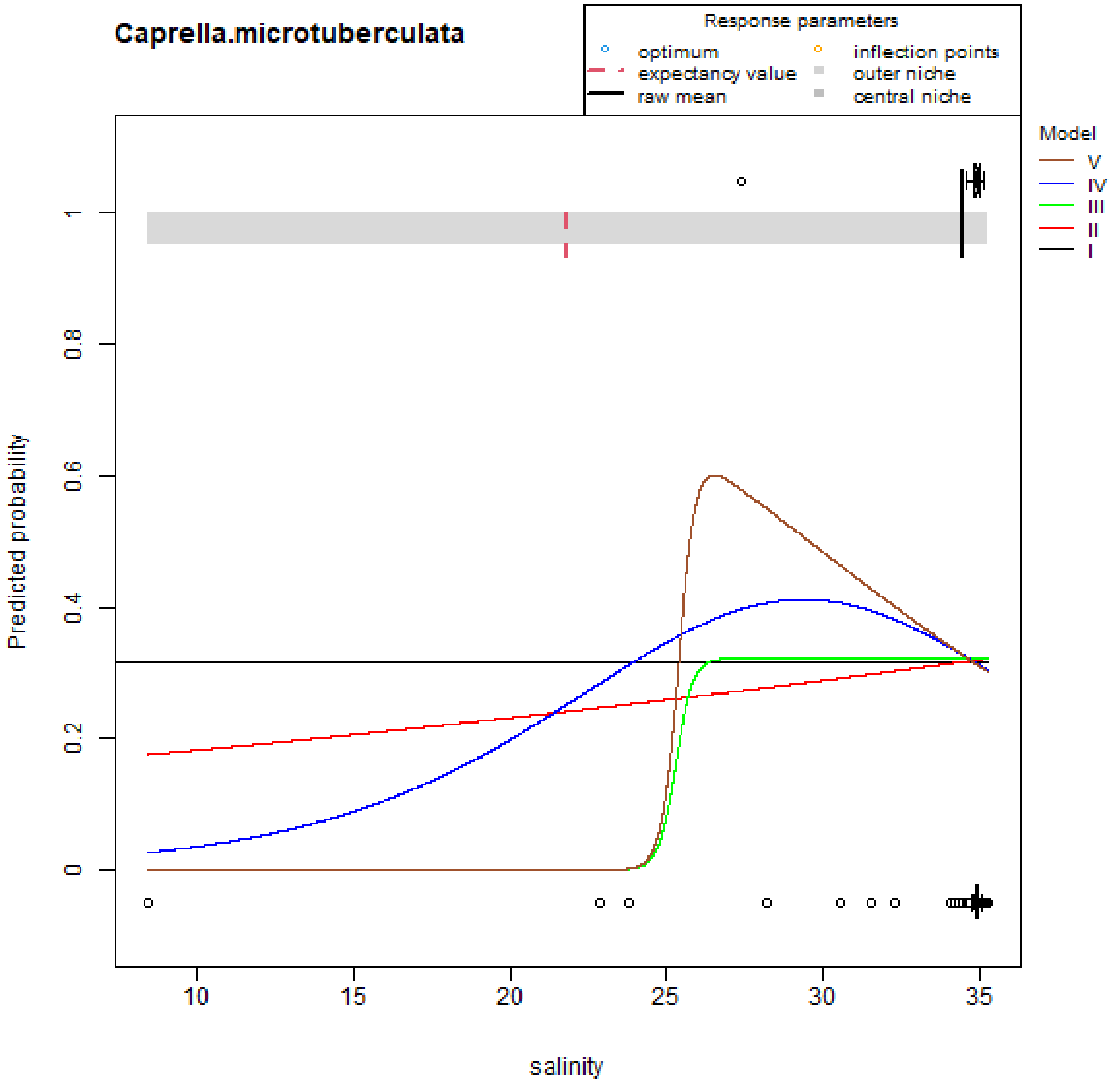

Caprella.microtuberculata
Response parameters
optimum
inflection points
expectancy value
outer niche
raw mean
central niche
Model
V
IV
III
II
I
Predicted probability
0
0.2
0.4
0.6
0.8
1
10
15
20
25
30
35
salinity

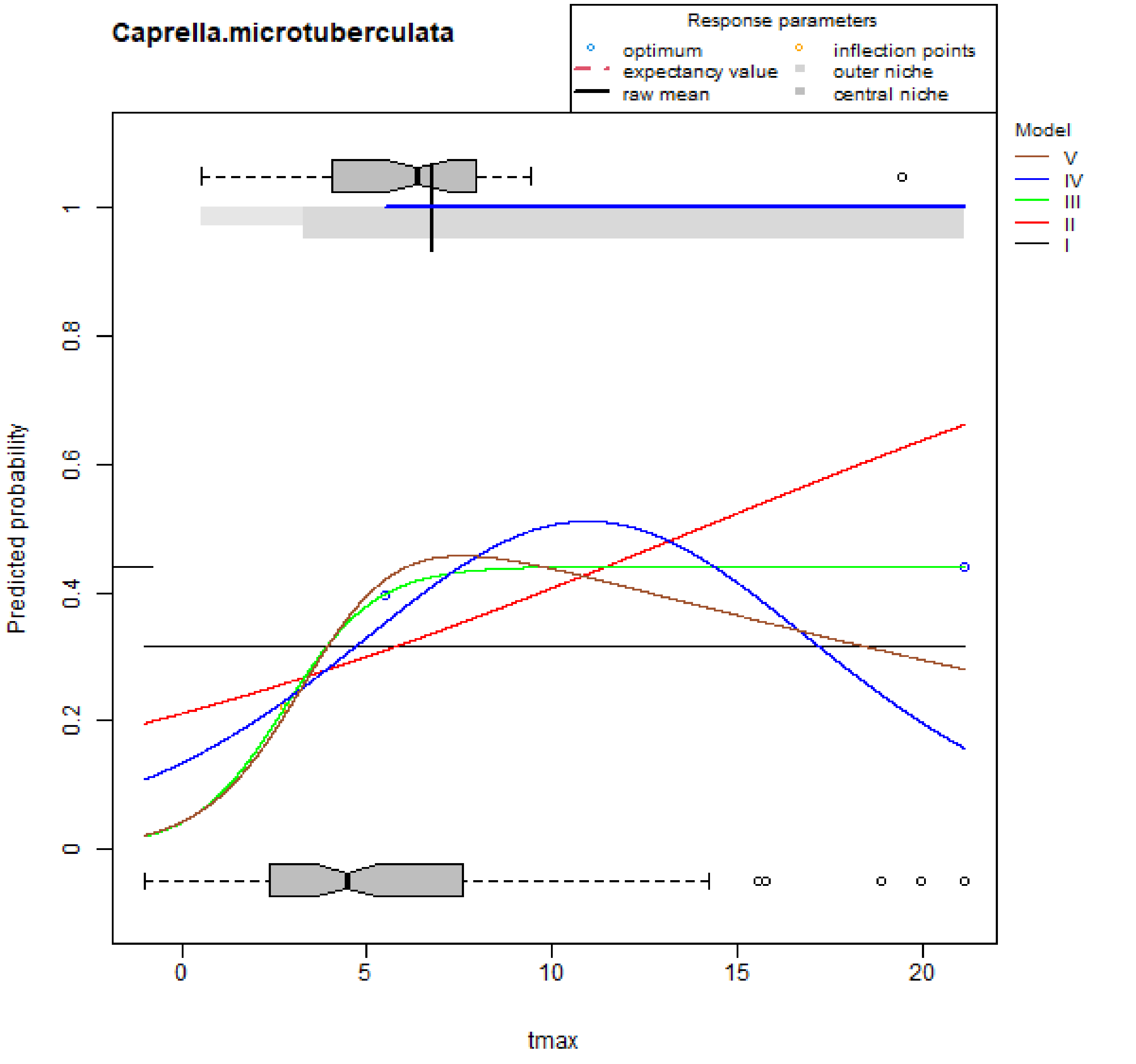

Caprella.microtuberculata
Response parameters
optimum
inflection points
expectancy value
outer niche
raw mean
central niche
Model
V
IV
III
II
I
Predicted probability
0
0.2
0.4
0.6
0.8
1
0
5
10
15
20
tmax

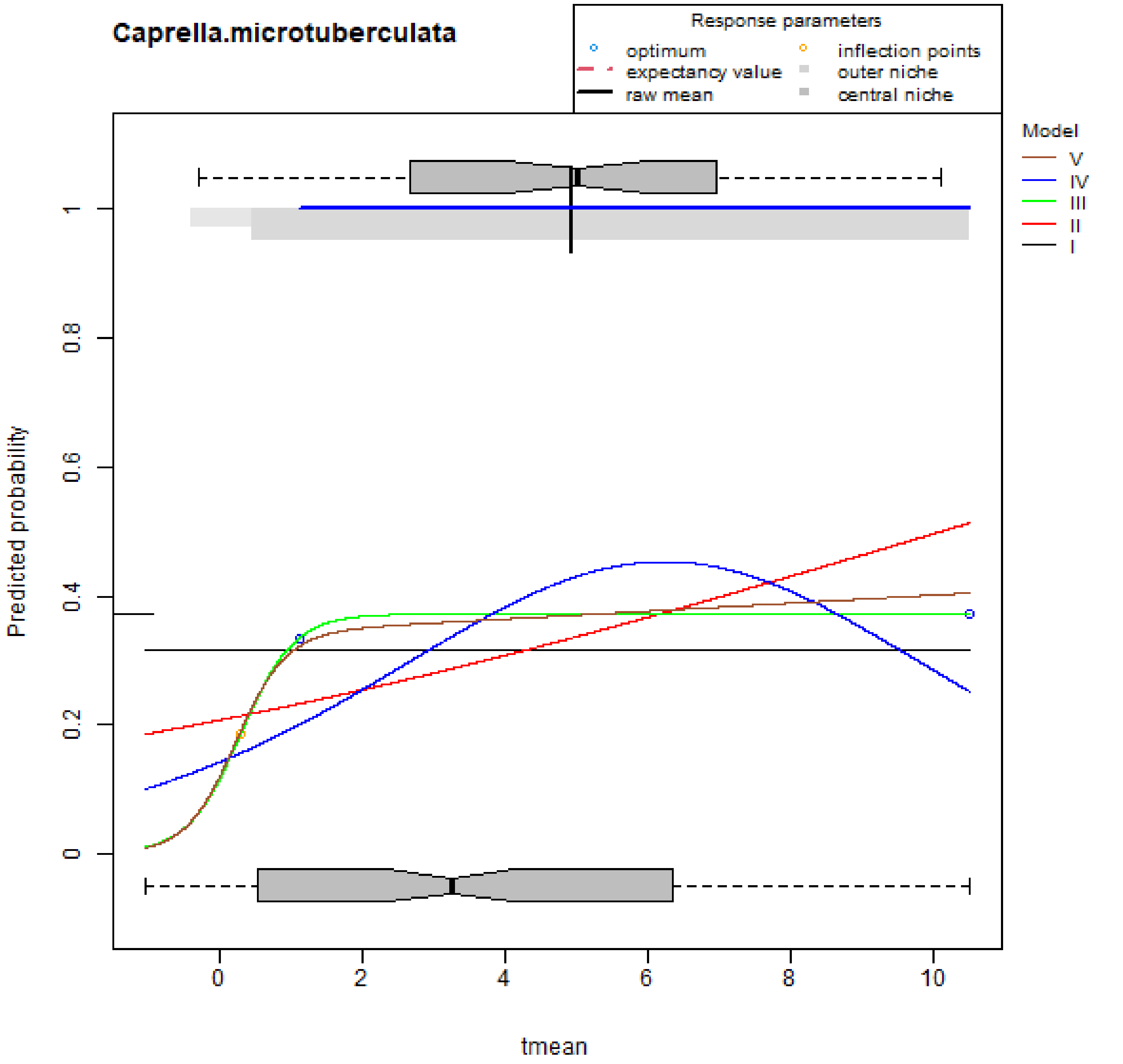

Caprella.microtuberculata
Response parameters
optimum
expectancy value
raw mean
inflection points
outer niche
central niche
Model
V
IV
III
II
I
1
0.8
0.6
0.4
0.2
0
Predicted probability
0
2
4
6
8
10
tmean

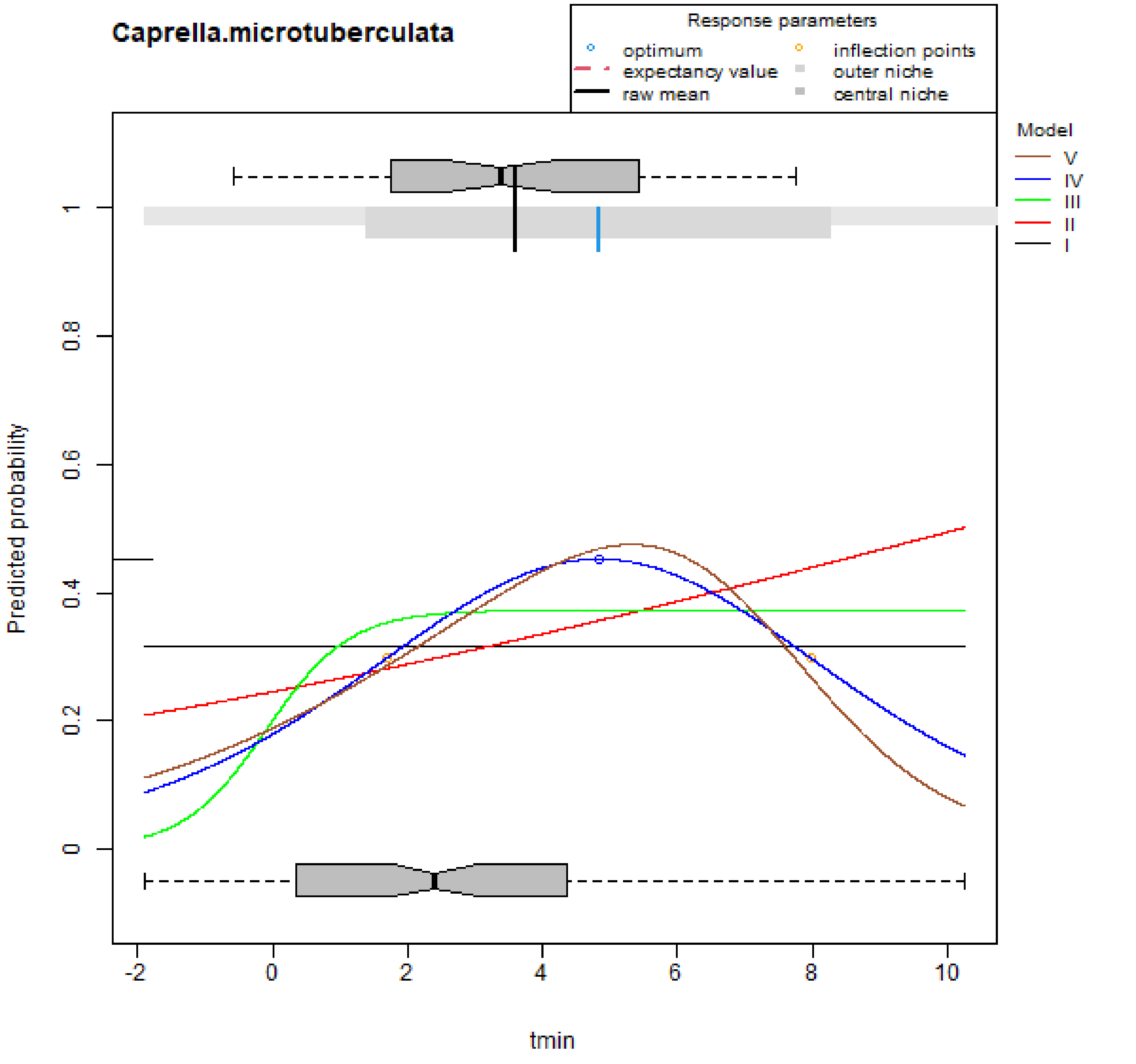

Caprella.microtuberculata
Response parameters
optimum
expectancy value
raw mean
inflection points
outer niche
central niche
Model
V
IV
III
II
I
Predicted probability
0
0.2
0.4
0.6
0.8
1
-2
0
2
4
6
8
10
tmin

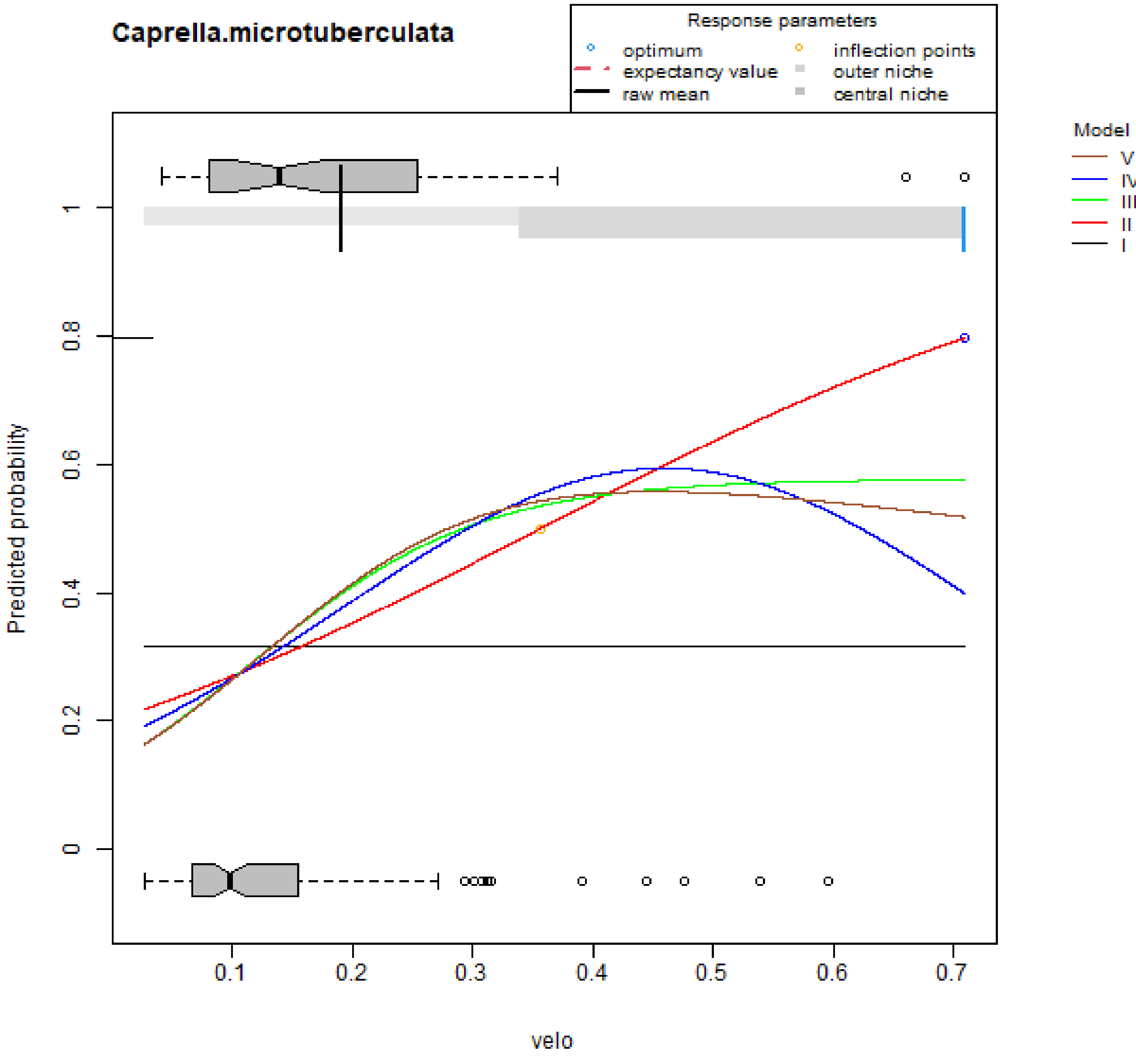

Caprella.microtuberculata
Response parameters
optimum
expectancy value
raw mean
inflection points
outer niche
central niche
Model
V
IV
III
II
I
Predicted probability
0
0.2
0.4
0.6
0.8
1
0.1
0.2
0.3
0.4
0.5
0.6
0.7
velo

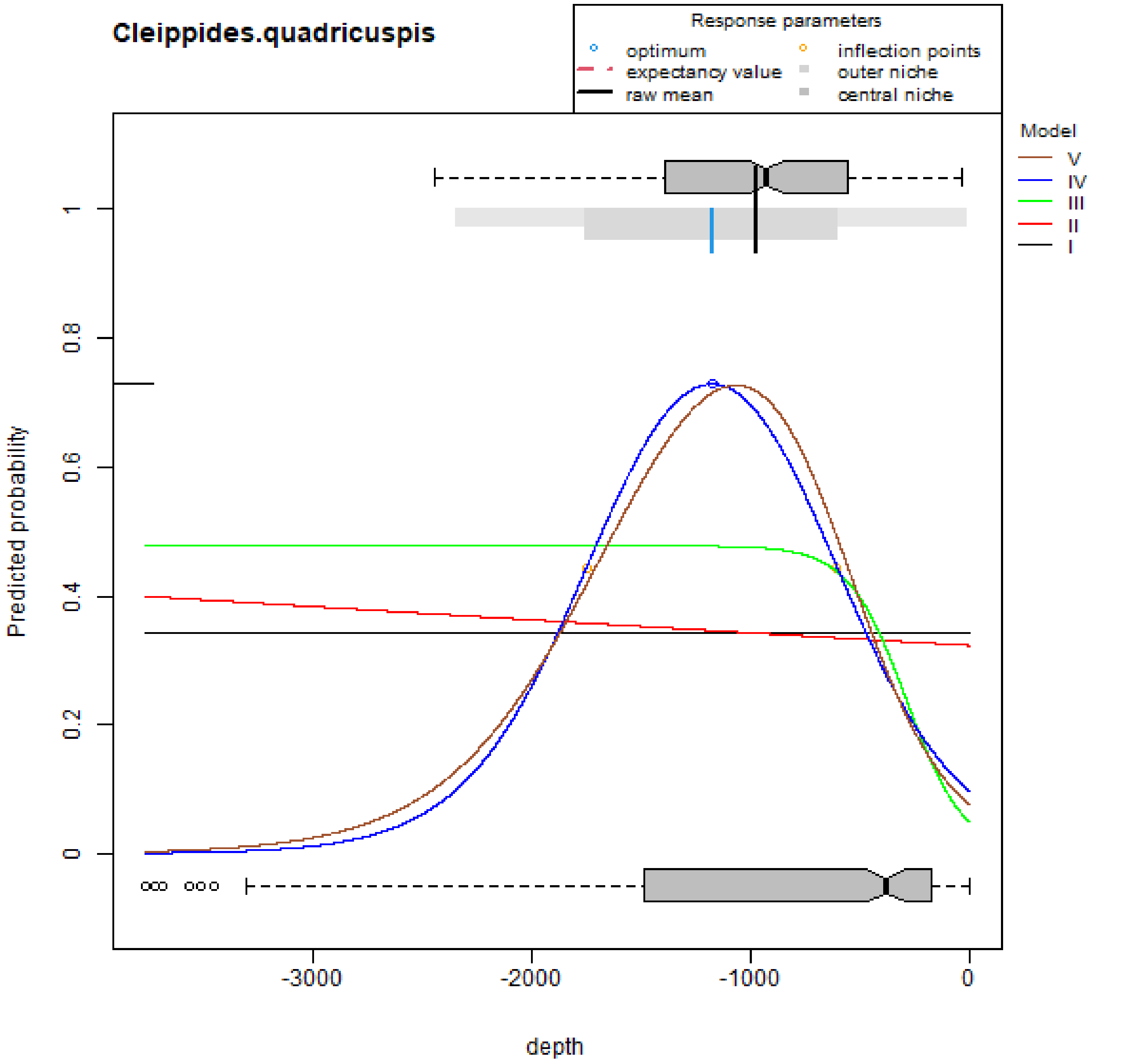

Cleippides.quadricuspis
Response parameters
optimum
expectancy value
raw mean
inflection points
outer niche
central niche
Model
V
IV
III
II
I
Predicted probability
0
0.2
0.4
0.6
0.8
1
-3000
-2000
-1000
0
depth

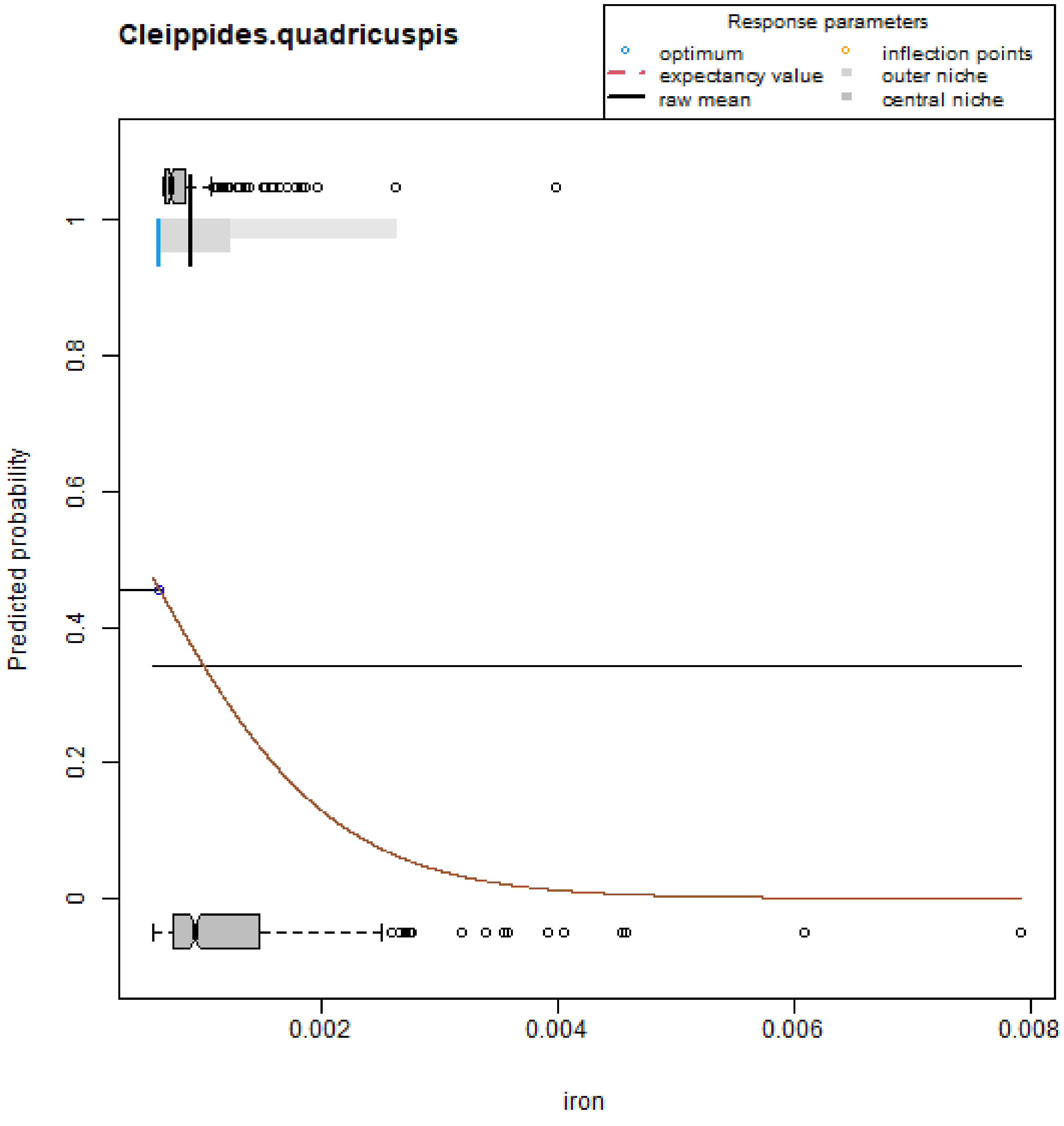

Cleippides.quadricuspis
Response parameters
optimum
expectancy value
raw mean
inflection points
outer niche
central niche
Predicted probability
0
0.2
0.4
0.6
0.8
1
0.002
0.004
0.006
0.008
iron

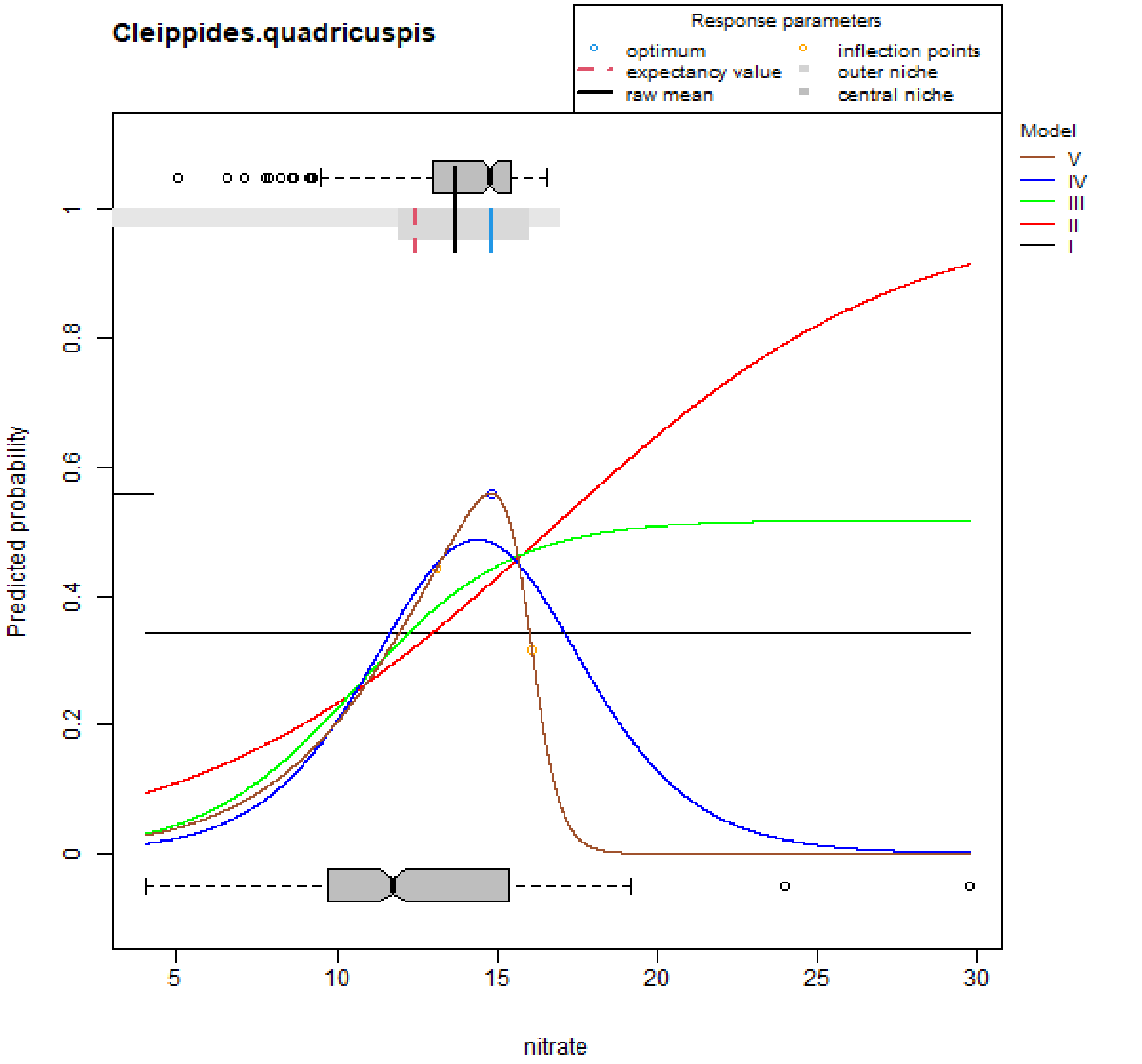

Cleippides.quadricuspis
Response parameters
optimum
expectancy value
raw mean
inflection points
outer niche
central niche
Model
V
IV
III
II
I
Predicted probability
0
0.2
0.4
0.6
0.8
1
5
10
15
20
25
30
nitrate

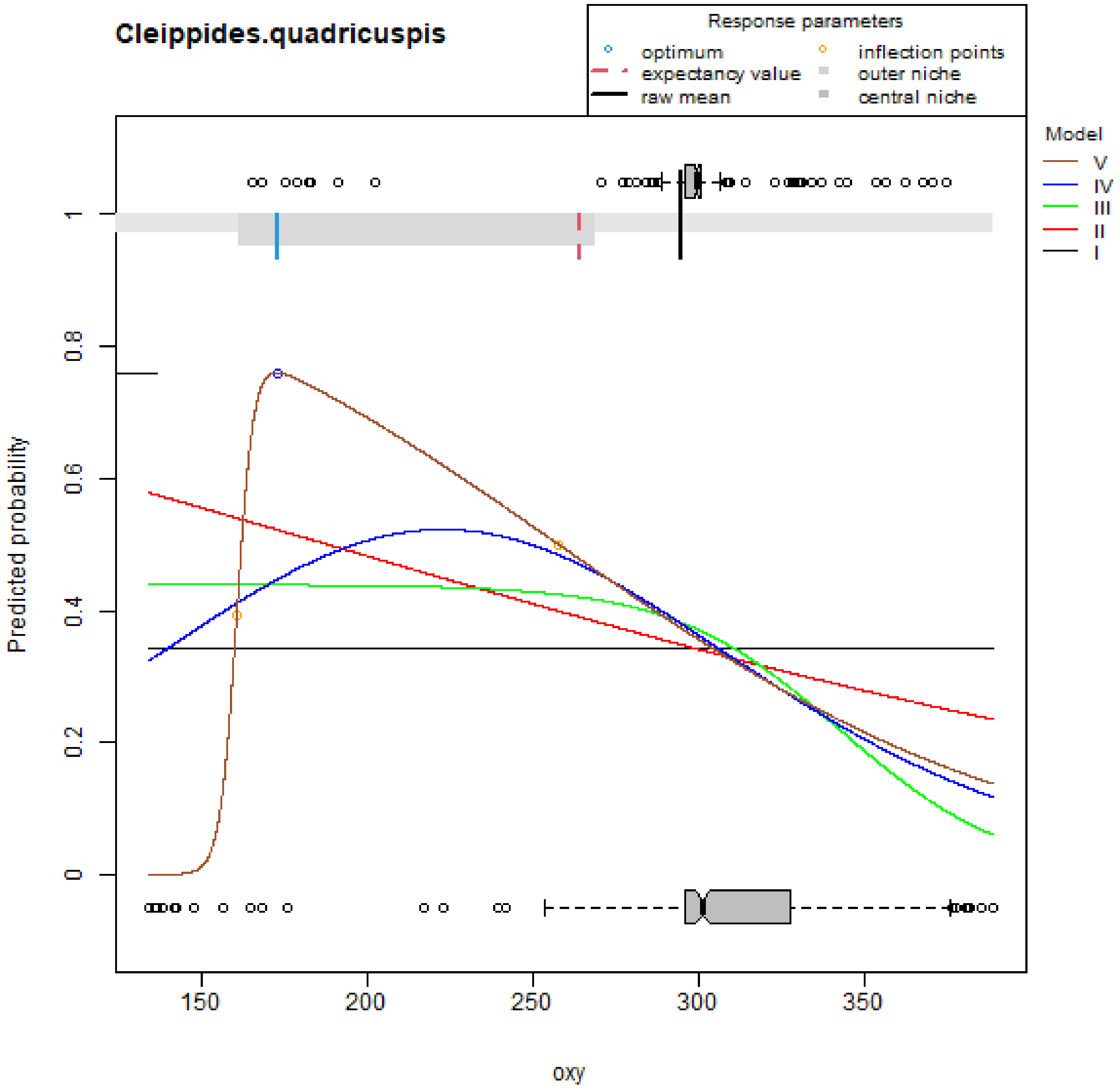
Cleippides.quadricuspis
Response parameters
optimum
expectancy value
raw mean
inflection points
outer niche
central niche
Model
V
IV
III
II
I
Predicted probability
0
0.2
0.4
0.6
0.8
1
150
200
250
300
350
oxy

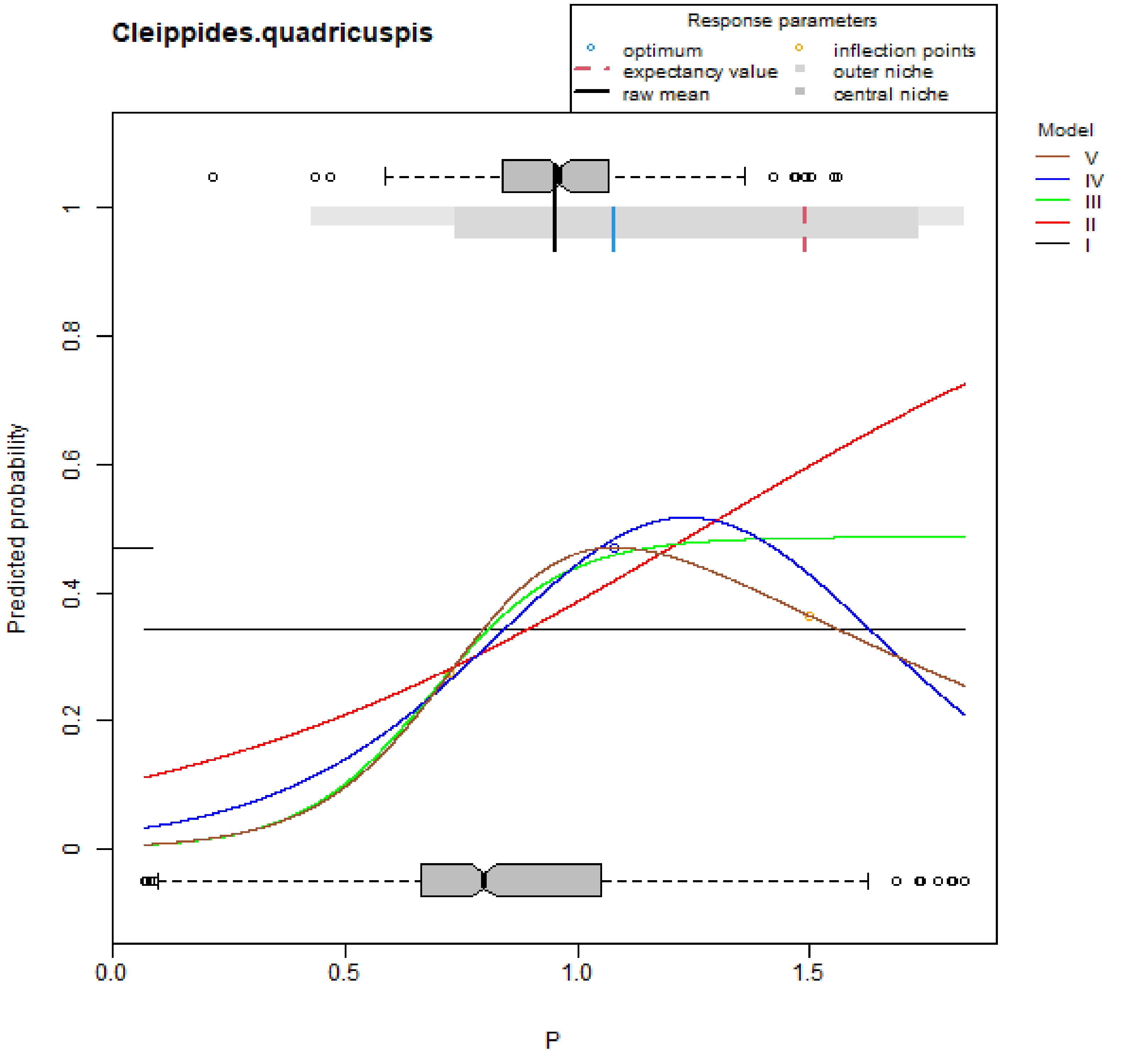

Cleippides.quadricuspis
Response parameters
optimum
expectancy value
raw mean
inflection points
outer niche
central niche
Model
V
IV
III
II
I
Predicted probability
0
0.2
0.4
0.6
0.8
1
0.0
0.5
1.0
1.5
P

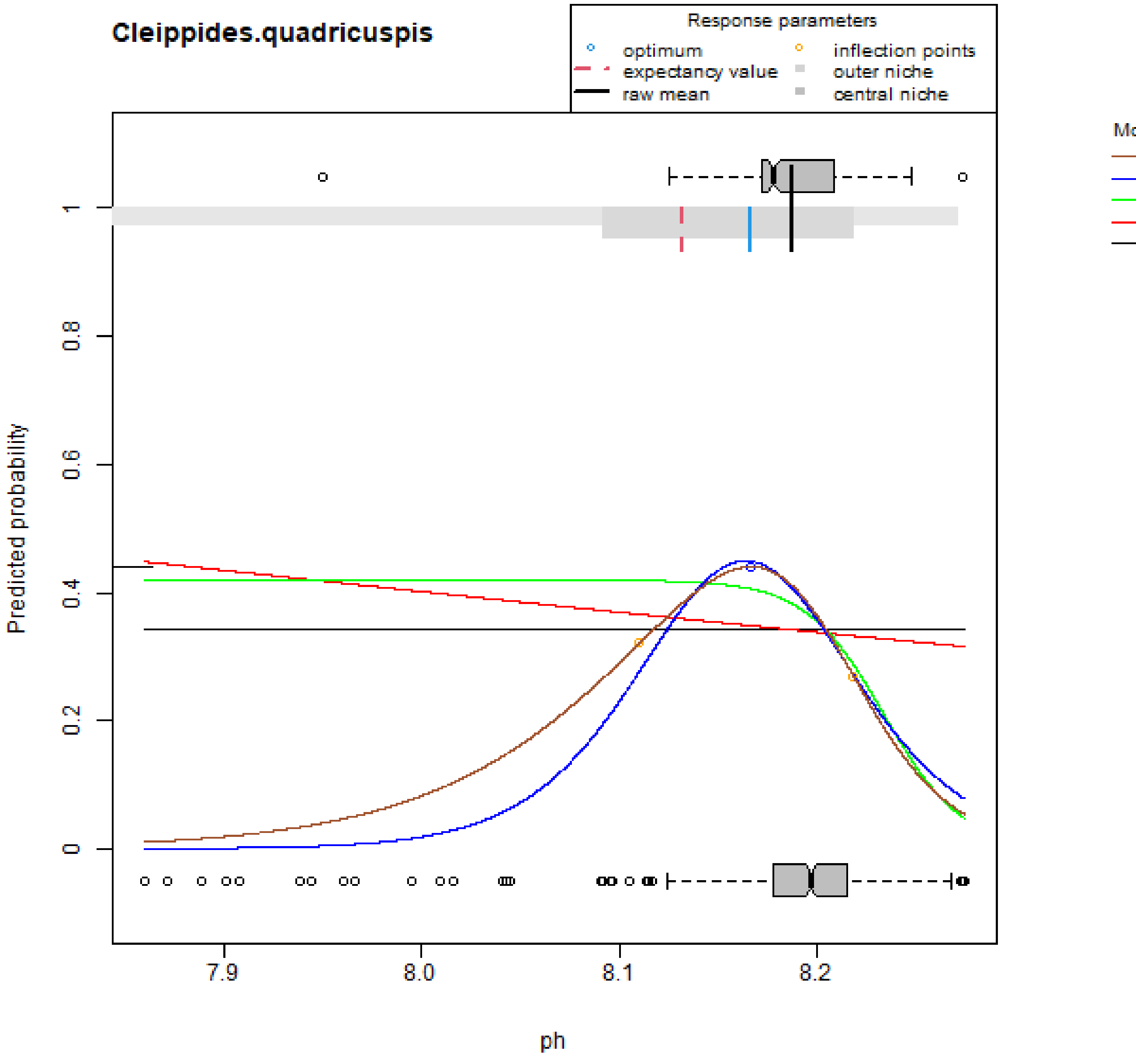

Cleippides.quadricuspis
Response parameters
optimum
expectancy value
raw mean
inflection points
outer niche
central niche
Mo
Predicted probability
0
0.2
0.4
0.6
0.8
1
7.9
8.0
8.1
8.2
ph

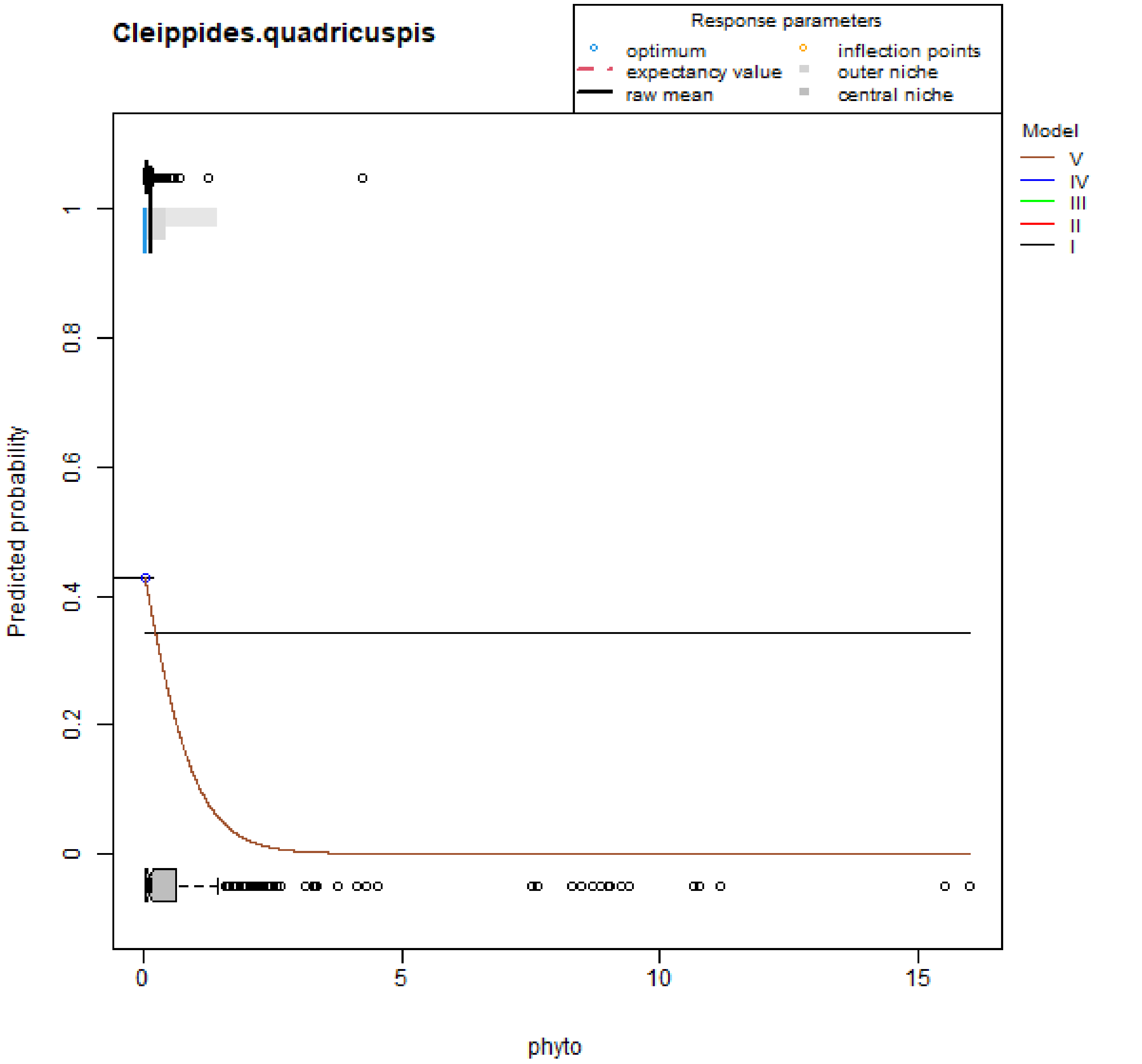

Cleippides.quadricuspis
Response parameters
optimum
expectancy value
raw mean
inflection points
outer niche
central niche
Model
V
IV
III
II
I
Predicted probability
0
0.2
0.4
0.6
0.8
1
0
5
10
15
phyto

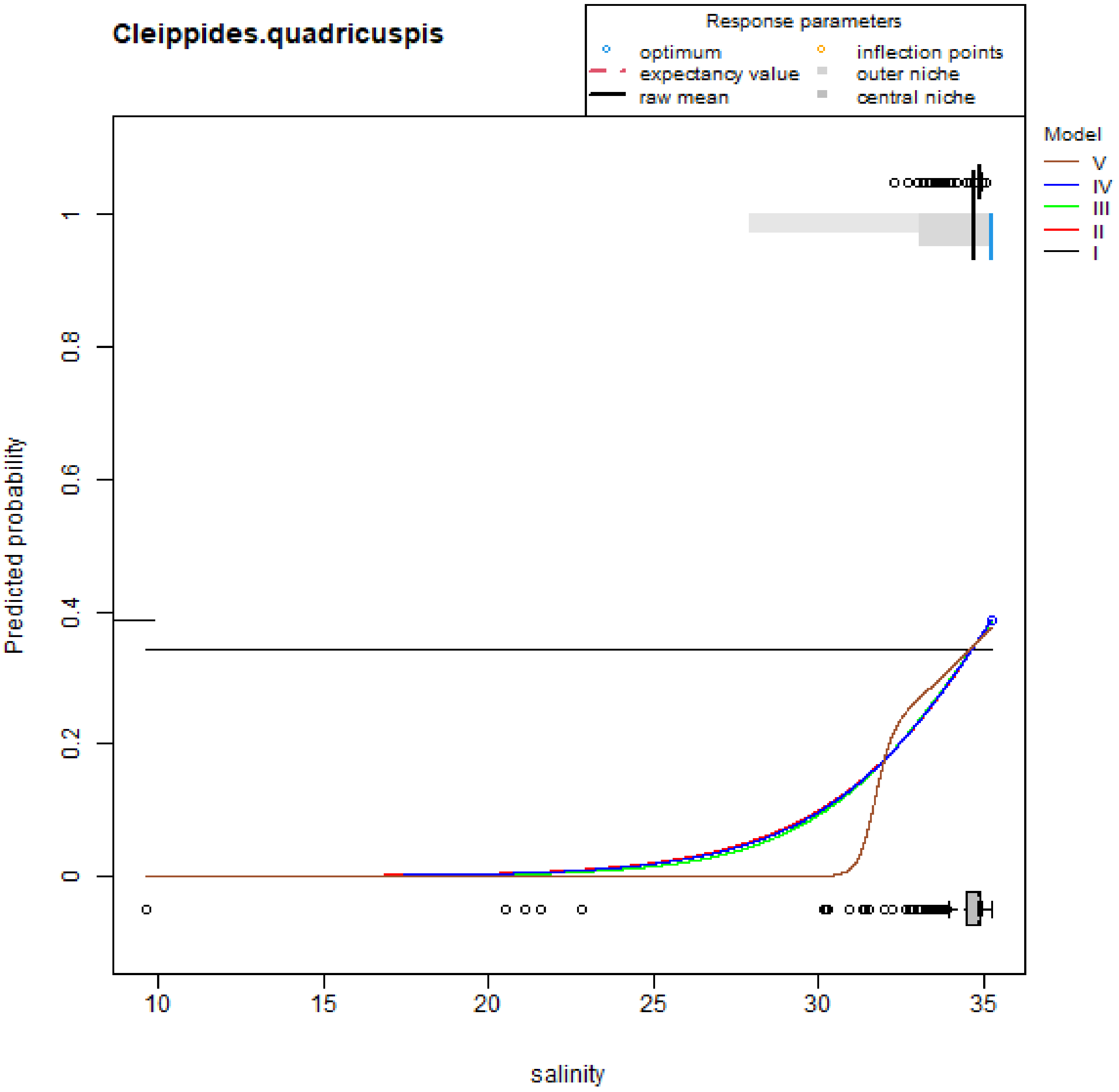
Cleippides.quadricuspis
Response parameters
optimum
expectancy value
raw mean
inflection points
outer niche
central niche
Model
V
IV
III
II
I
Predicted probability
0
0.2
0.4
0.6
0.8
1
10
15
20
25
30
35
salinity

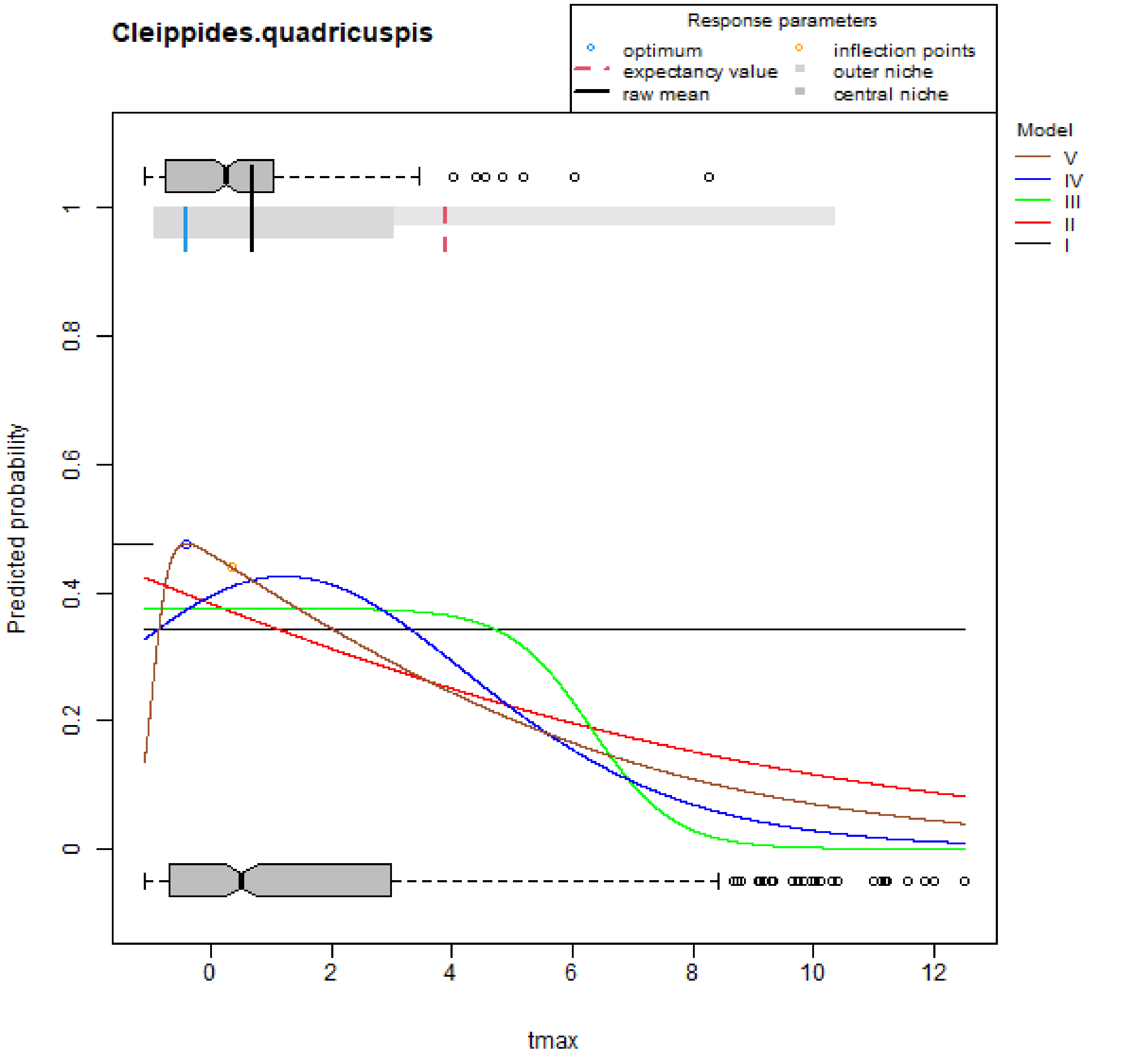

Cleippides.quadricuspis
Response parameters
optimum
expectancy value
raw mean
inflection points
outer niche
central niche
Model
V
IV
III
II
I
Predicted probability
0
0.2
0.4
0.6
0.8
1
0
2
4
6
8
10
12
tmax

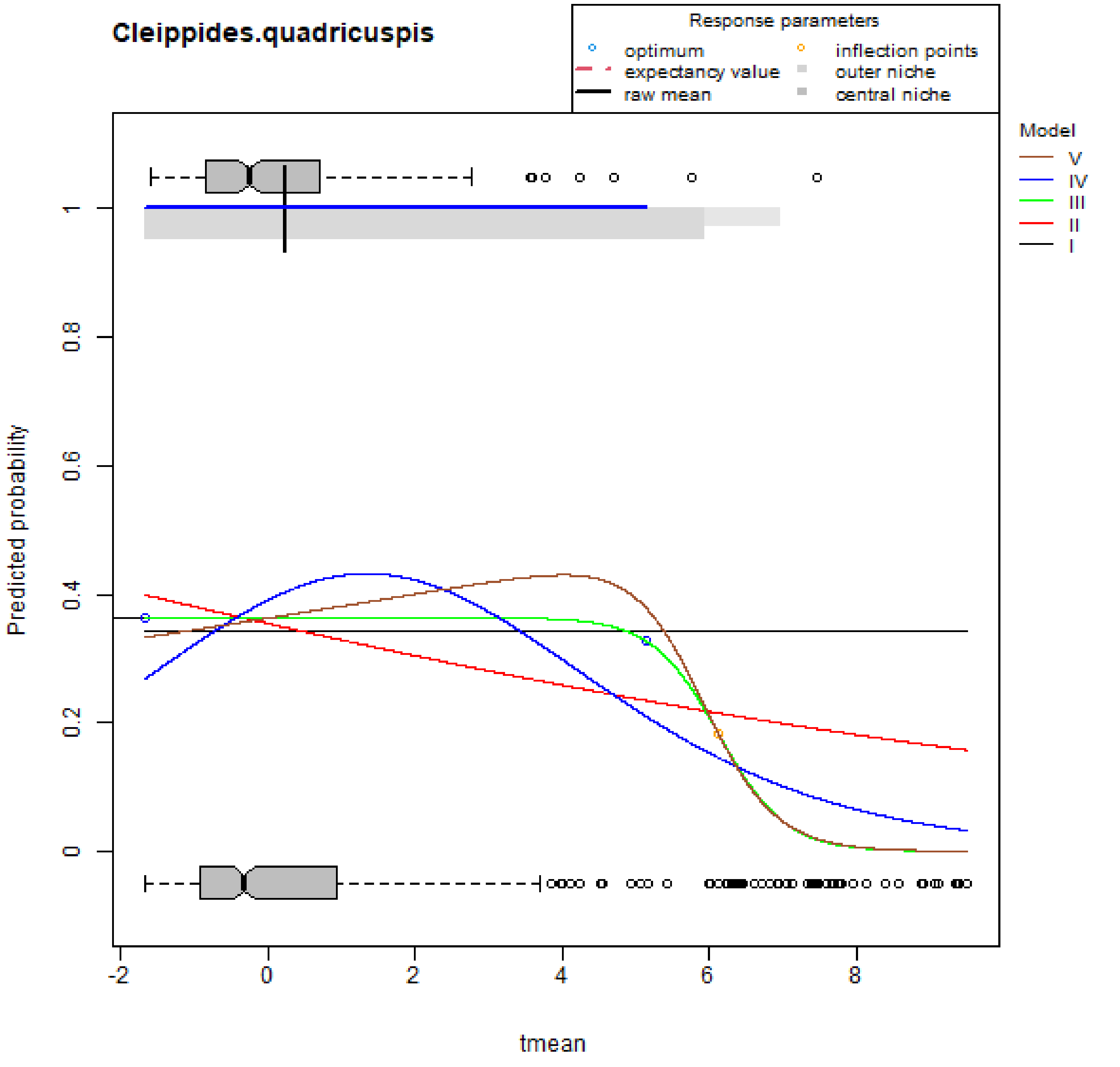
Cleippides.quadricuspis
Response parameters
optimum
expectancy value
raw mean
inflection points
outer niche
central niche
Model
V
IV
III
II
I
Predicted probability
0
0.2
0.4
0.6
0.8
1
-2
0
2
4
6
8
tmean

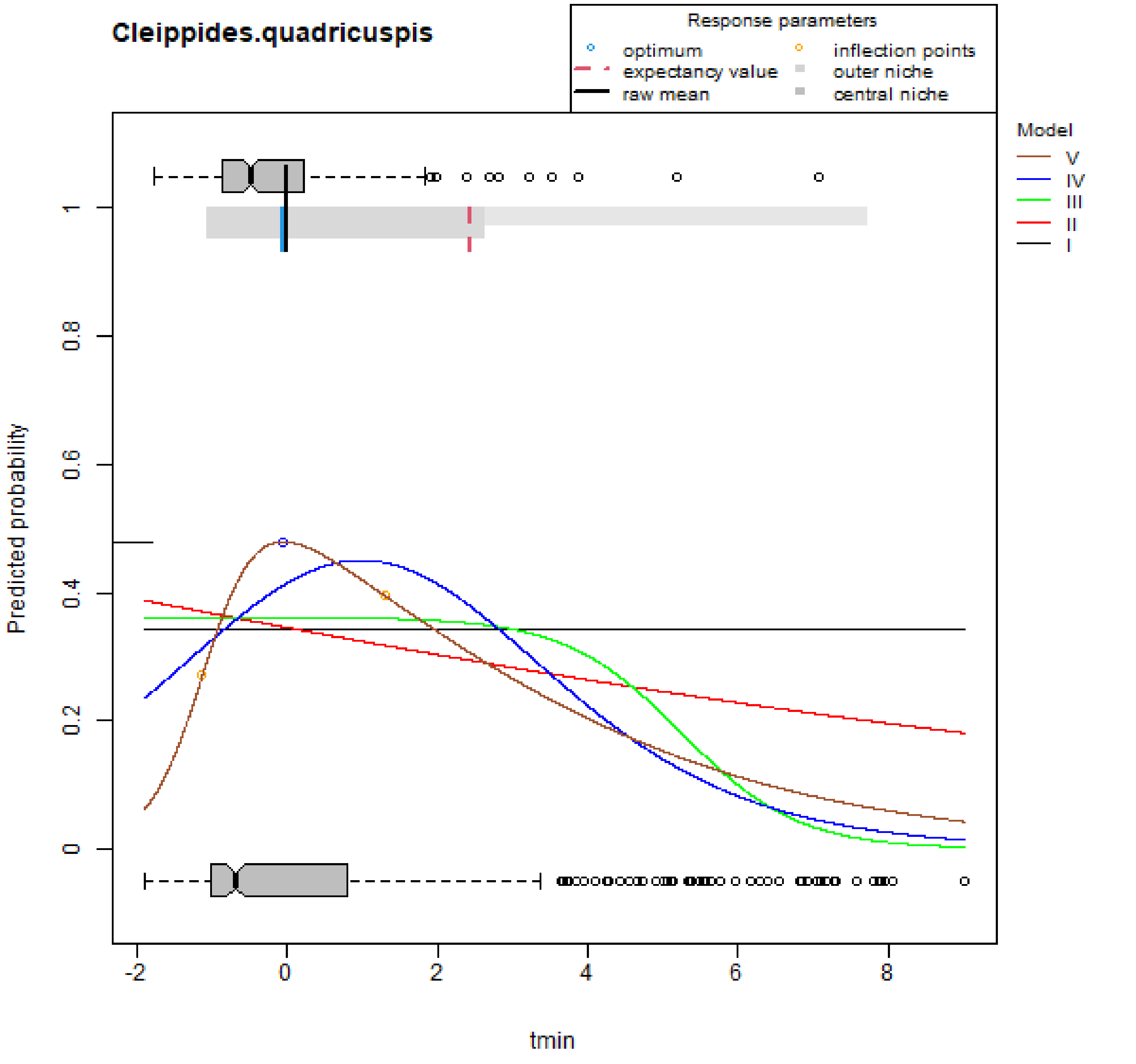

Cleippides.quadricuspis
Response parameters
optimum
expectancy value
raw mean
inflection points
outer niche
central niche
Model
V
IV
III
II
I
Predicted probability
0
0.2
0.4
0.6
0.8
1
-2
0
2
4
6
8
tmin

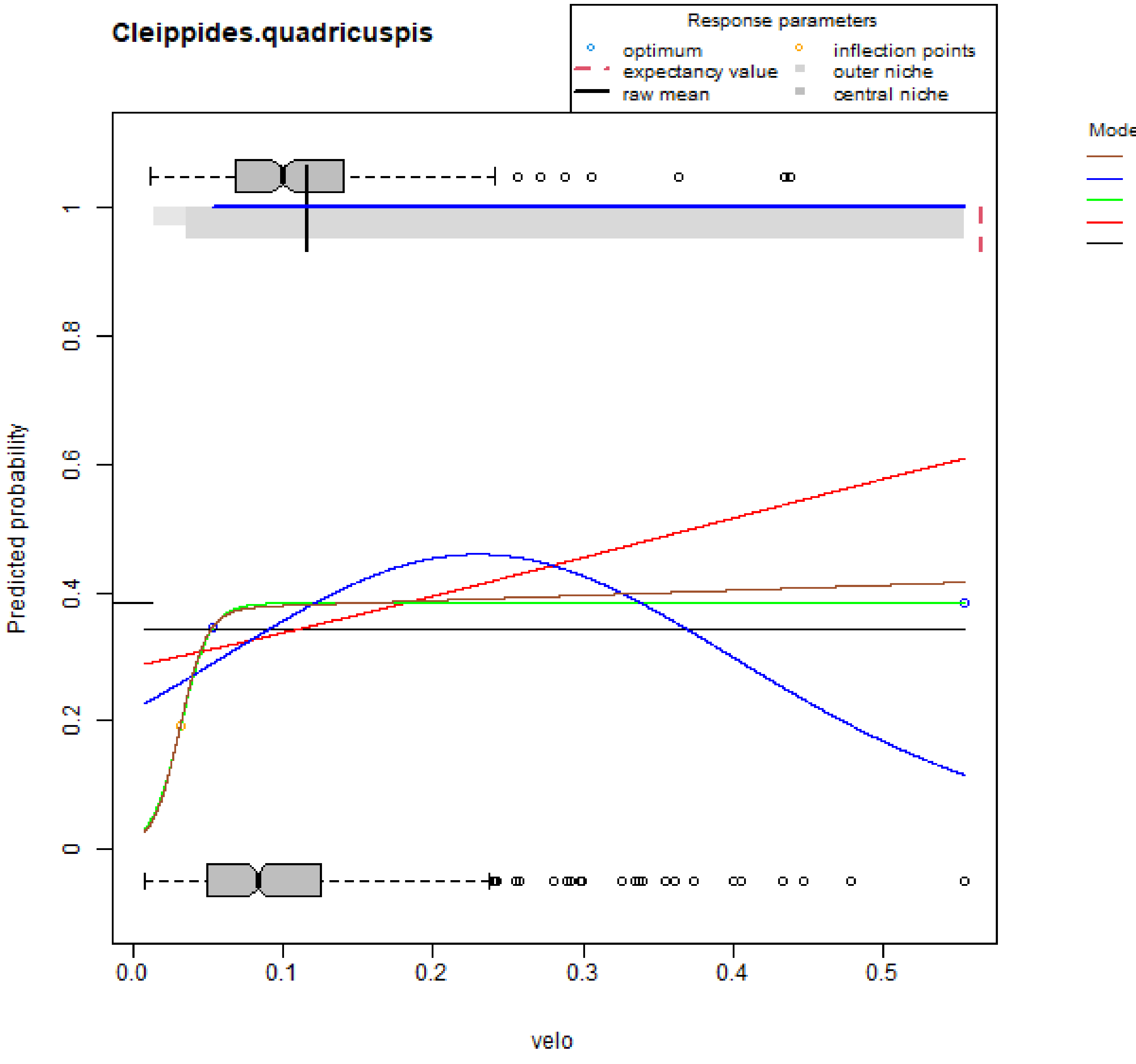

Cleippides.quadricuspis
Response parameters
optimum
inflection points
expectancy value
outer niche
raw mean
central niche
Mode
Predicted probability
0
0.2
0.4
0.6
0.8
1
0.0
0.1
0.2
0.3
0.4
0.5
velo

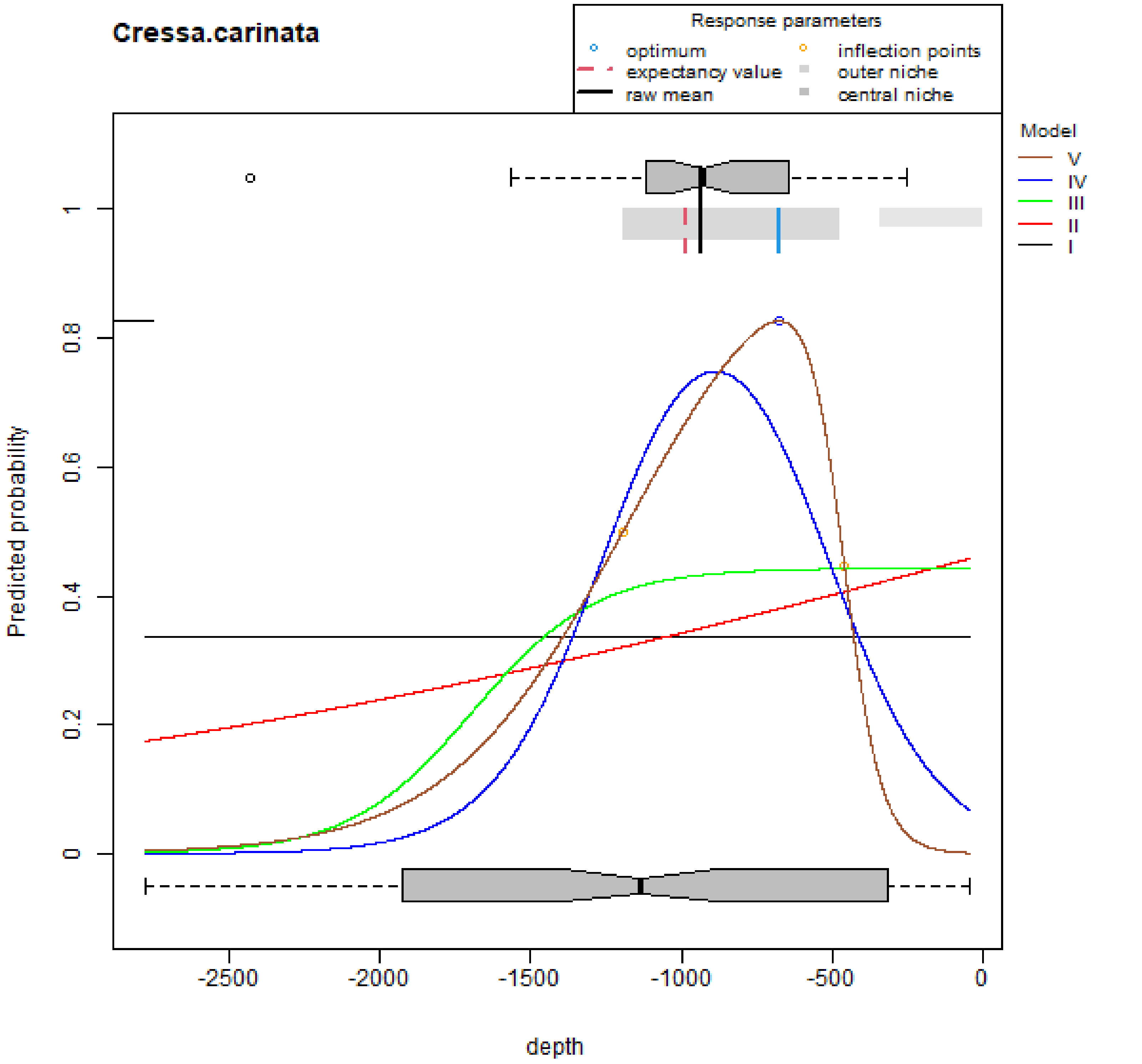

Cressa.carinata
Response parameters
optimum
inflection points
expectancy value
outer niche
raw mean
central niche
Model
V
IV
III
II
I
Predicted probability
0
0.2
0.4
0.6
0.8
1
-2500
-2000
-1500
-1000
-500
0
depth

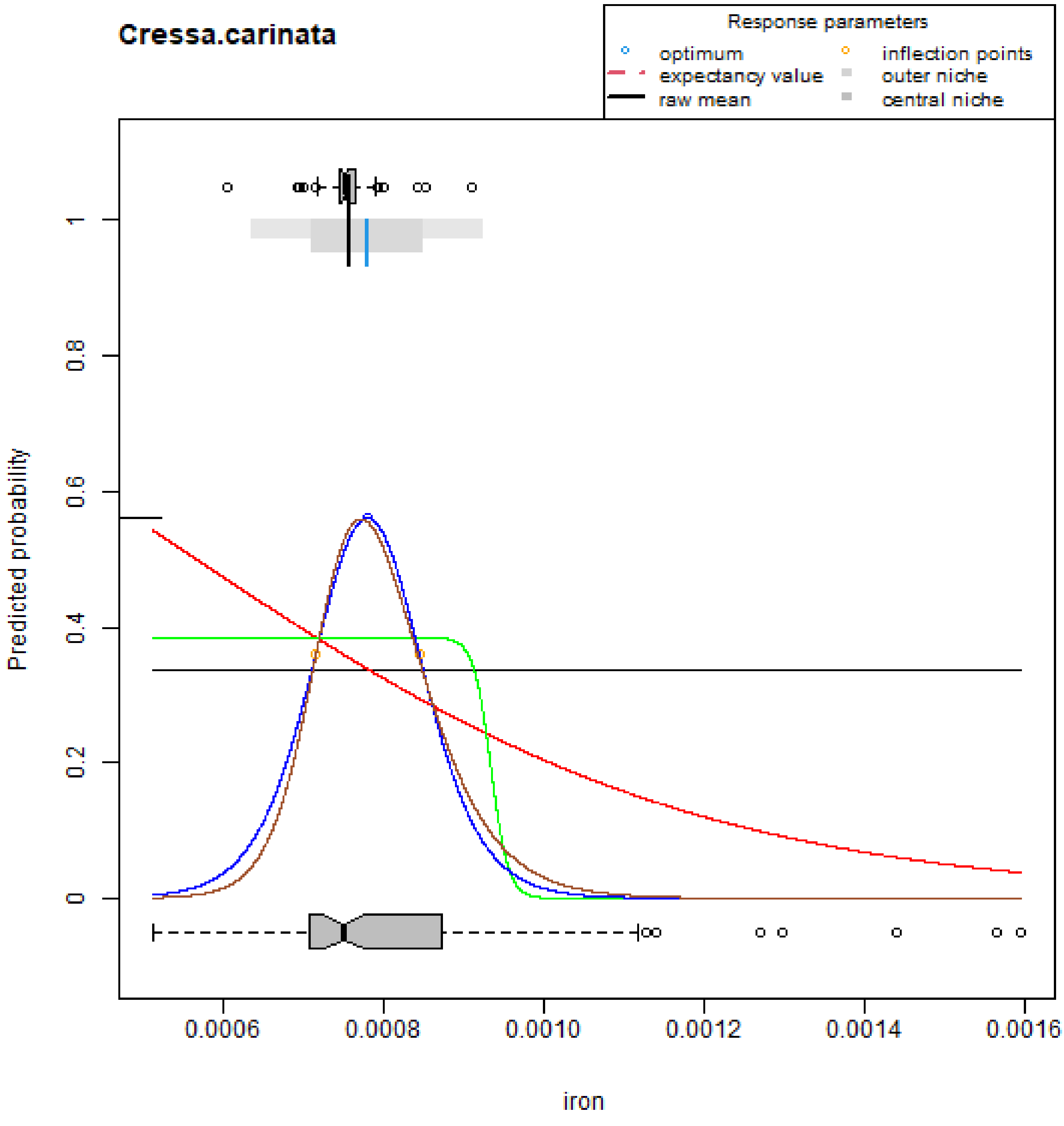

Cressa.carinata
Response parameters
optimum
expectancy value
raw mean
inflection points
outer niche
central niche
Predicted probability
0
0.2
0.4
0.6
0.8
1
0.0006
0.0008
0.0010
0.0012
0.0014
0.0016
iron

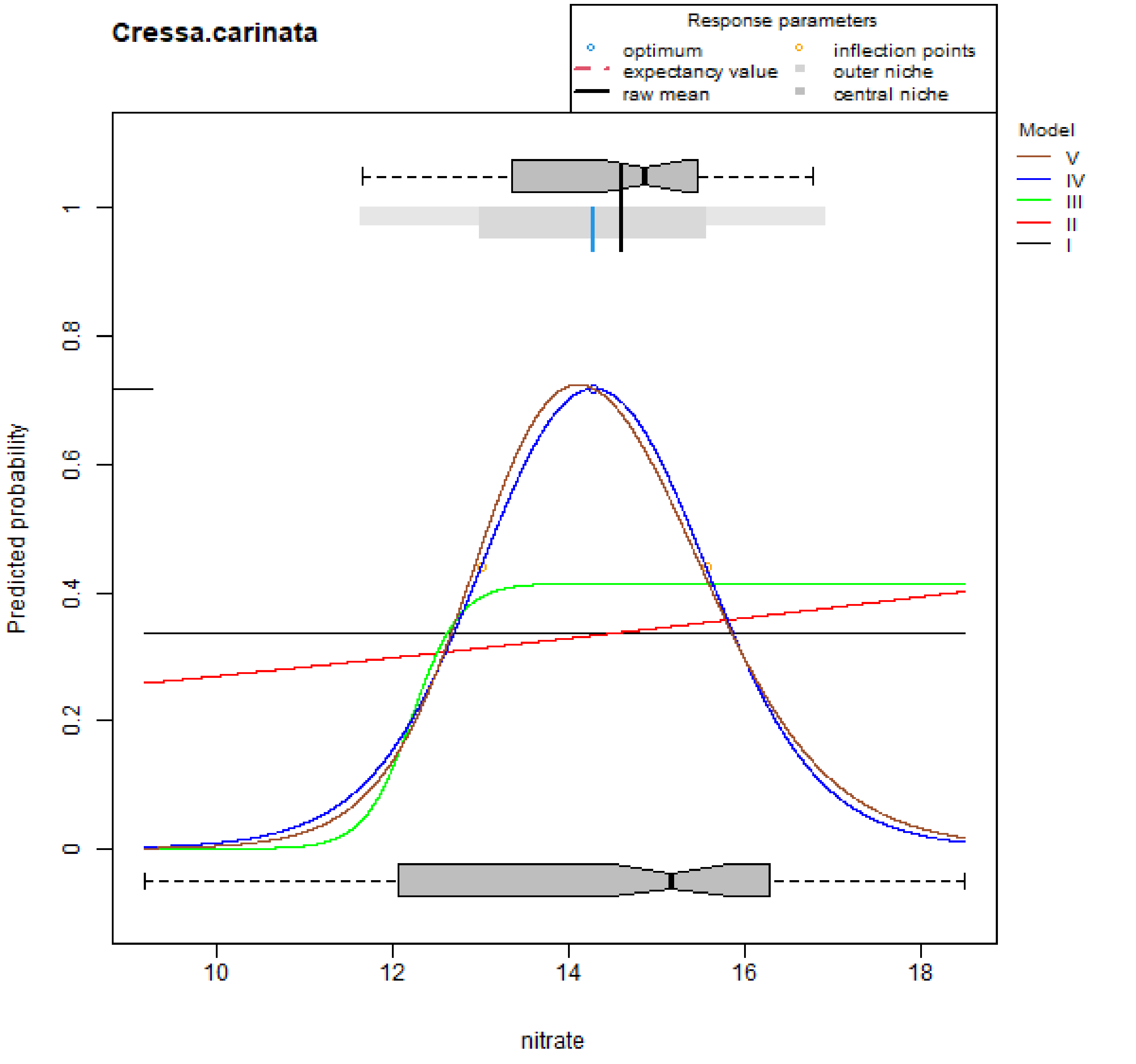

Cressa.carinata
Response parameters
optimum
expectancy value
raw mean
inflection points
outer niche
central niche
Model
V
IV
III
II
I
Predicted probability
0
0.2
0.4
0.6
0.8
1
10
12
14
16
18
nitrate

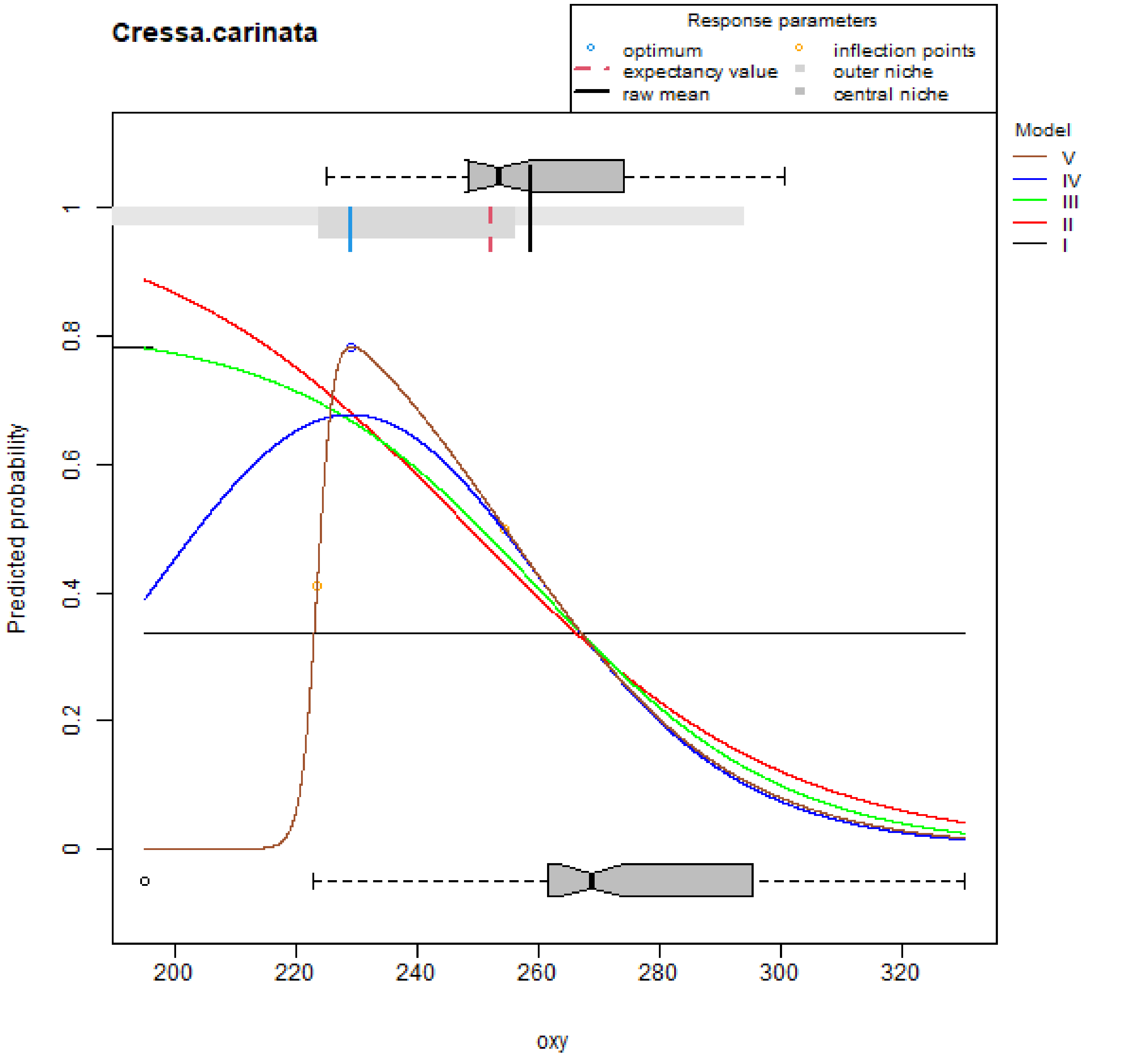
Cressa.carinata
Response parameters
optimum
expectancy value
raw mean
inflection points
outer niche
central niche
Model
V
IV
III
II
I
Predicted probability
0
0.2
0.4
0.6
0.8
1
200
220
240
260
280
300
320
oxy

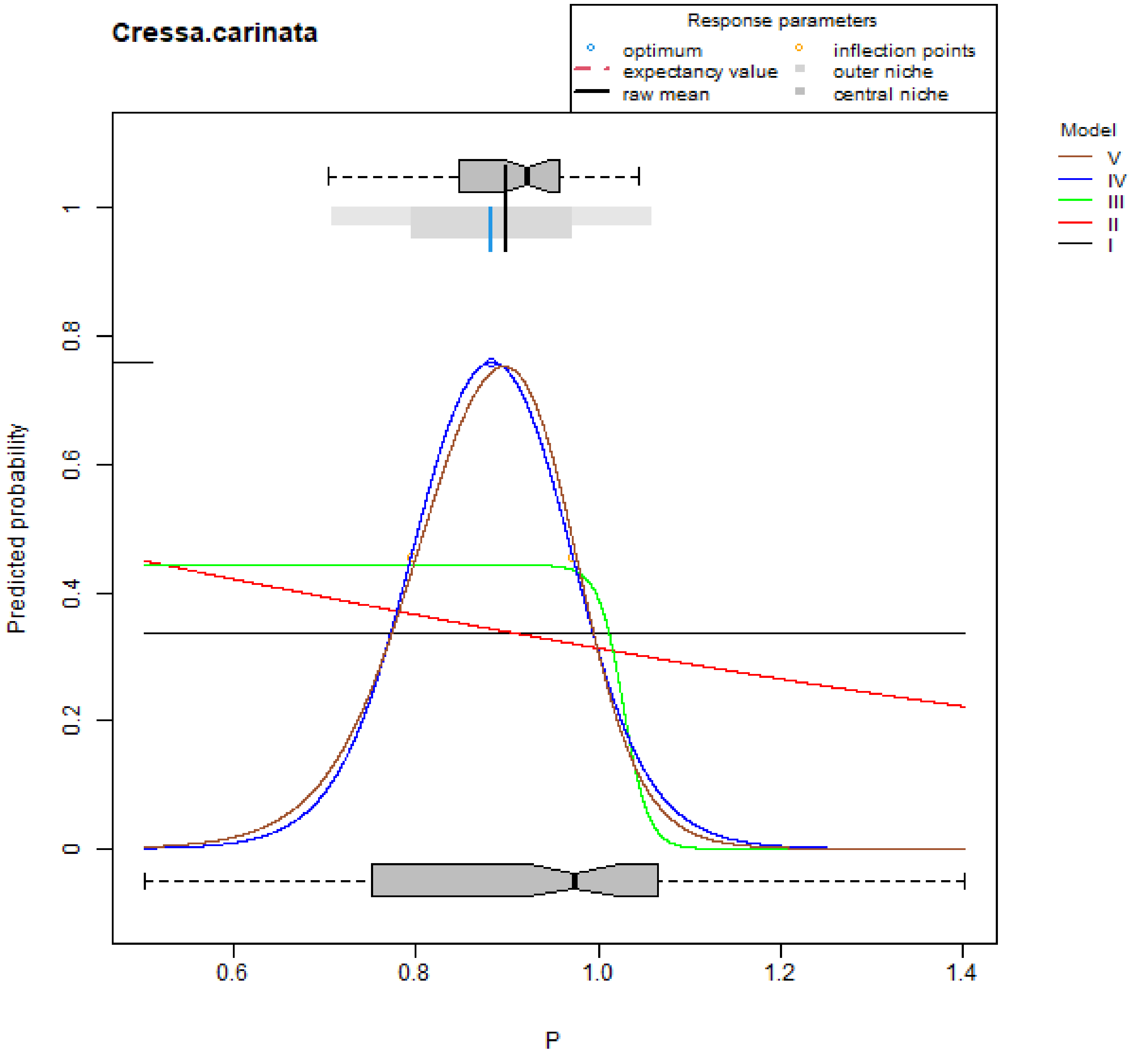
Cressa.carinata
Response parameters
optimum
expectancy value
raw mean
inflection points
outer niche
central niche
Model
V
IV
III
II
I
Predicted probability
0
0.2
0.4
0.6
0.8
1
0.6
0.8
1.0
1.2
1.4
P

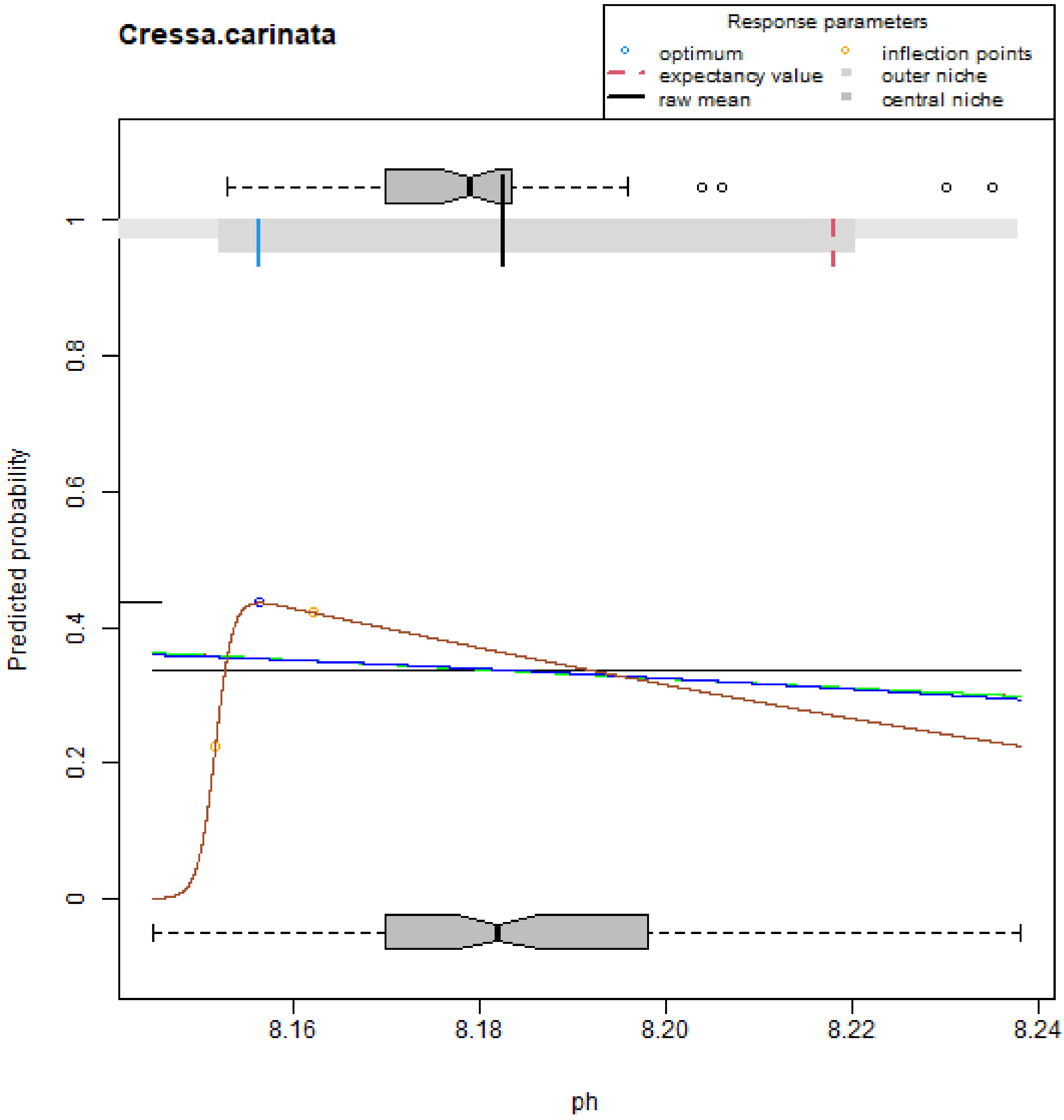

Cressa.carinata
Response parameters
optimum
expectancy value
raw mean
inflection points
outer niche
central niche
Predicted probability
0
0.2
0.4
0.6
0.8
1
8.16
8.18
8.20
8.22
8.24
ph

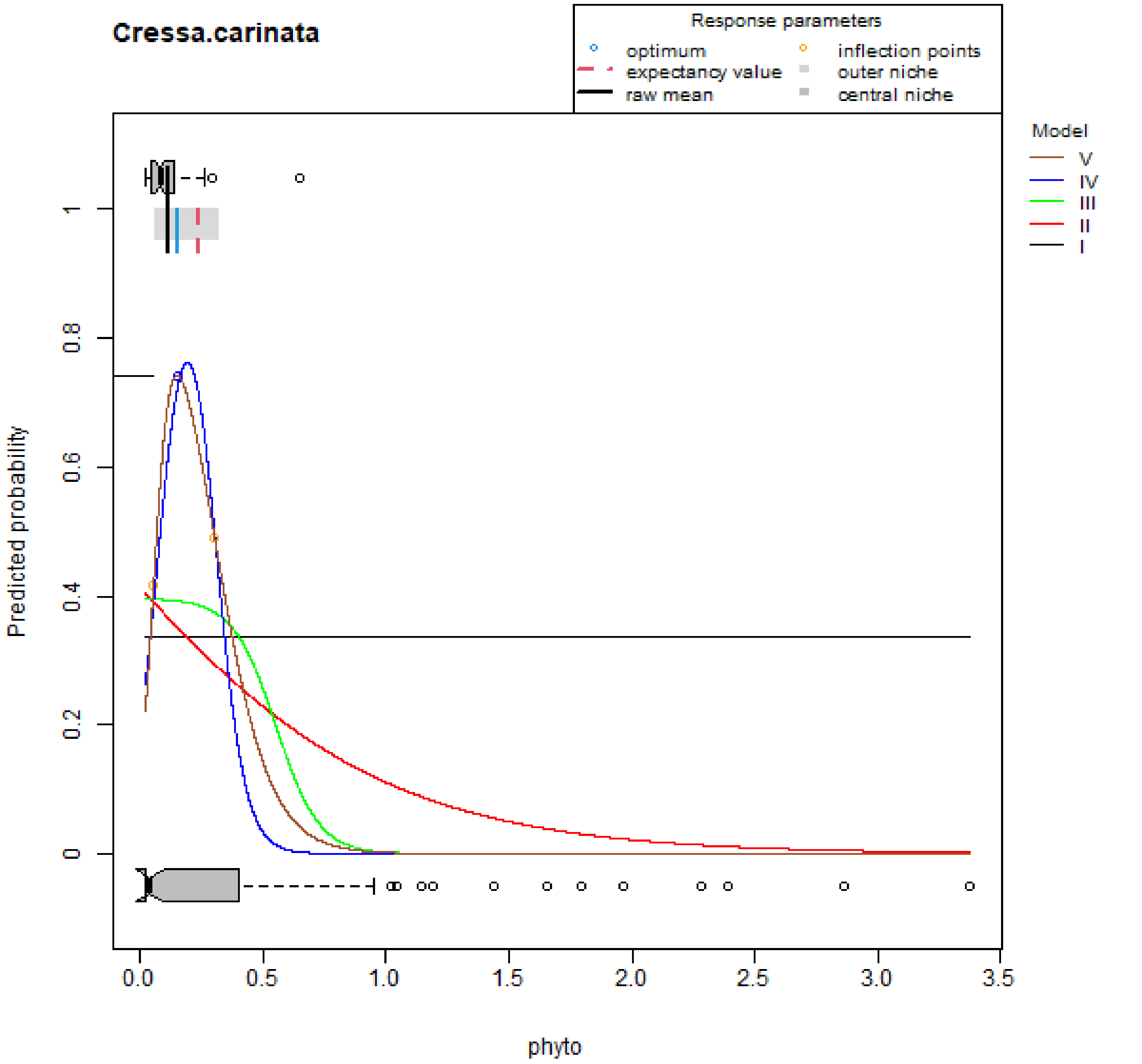
Cressa.carinata
Response parameters
optimum
expectancy value
raw mean
inflection points
outer niche
central niche
Model
V
IV
III
II
I
Predicted probability
0
0.2
0.4
0.6
0.8
1
0.0
0.5
1.0
1.5
2.0
2.5
3.0
3.5
phyto

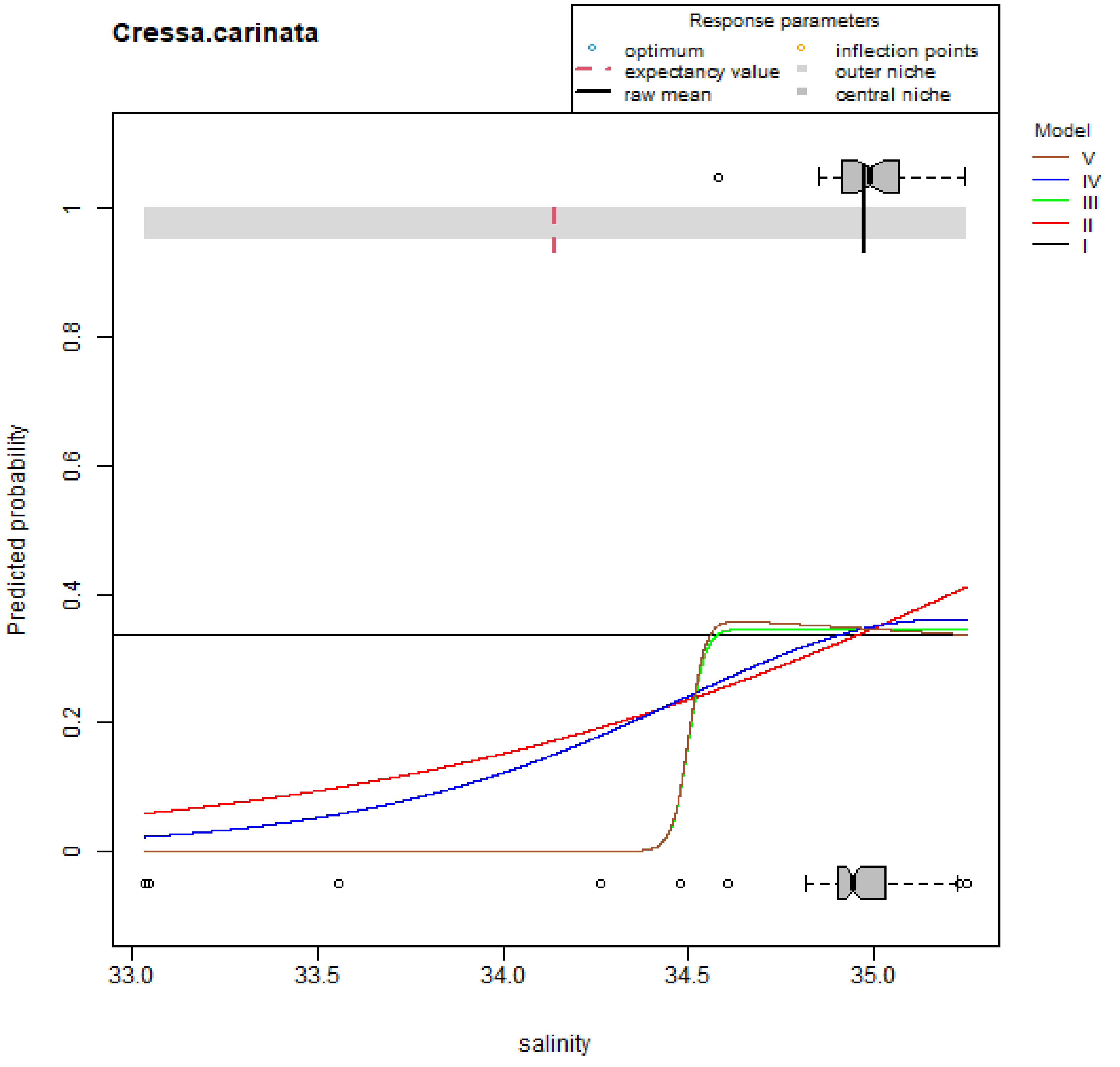

Cressa.carinata
Response parameters
optimum
expectancy value
raw mean
inflection points
outer niche
central niche
Model
V
IV
III
II
I
Predicted probability
0
0.2
0.4
0.6
0.8
1
33.0
33.5
34.0
34.5
35.0
salinity

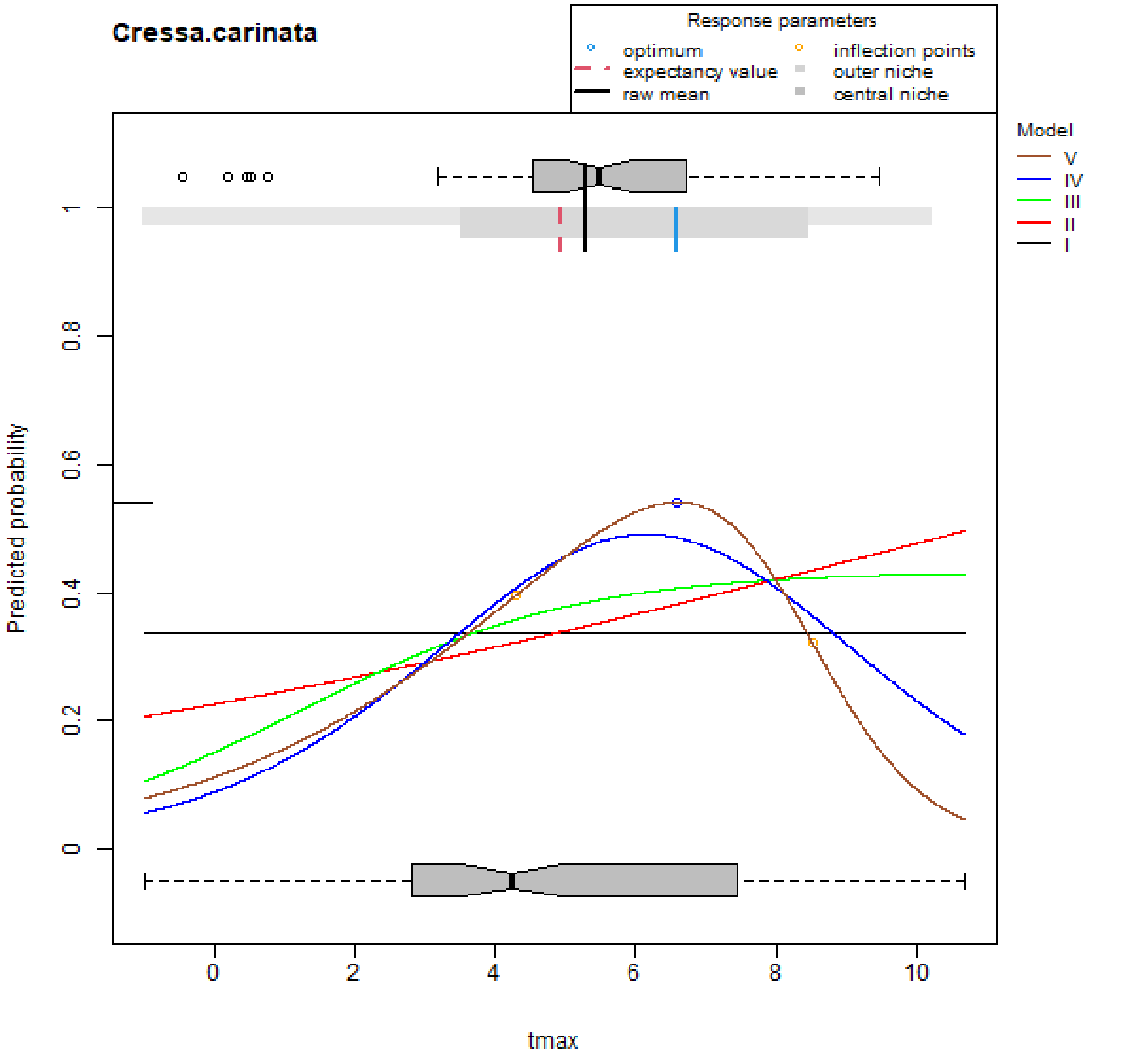

Cressa.carinata
Response parameters
optimum
expectancy value
raw mean
inflection points
outer niche
central niche
Model
V
IV
III
II
I
Predicted probability
0
0.2
0.4
0.6
0.8
1
0
2
4
6
8
10
tmax

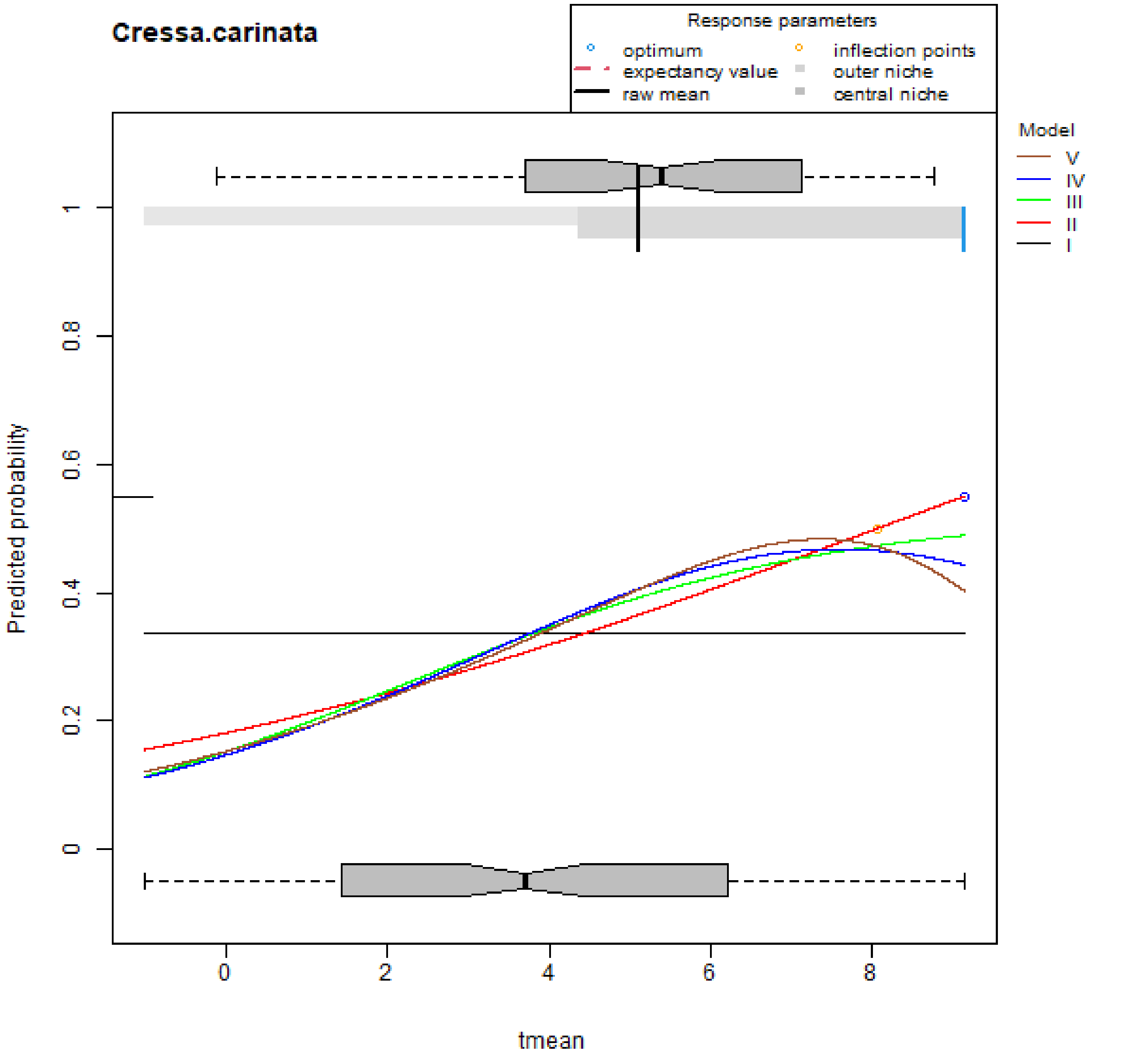

Cressa.carinata
Response parameters
optimum
expectancy value
raw mean
inflection points
outer niche
central niche
Model
V
IV
III
II
I
Predicted probability
0
0.2
0.4
0.6
0.8
1
0
2
4
6
8
tmean

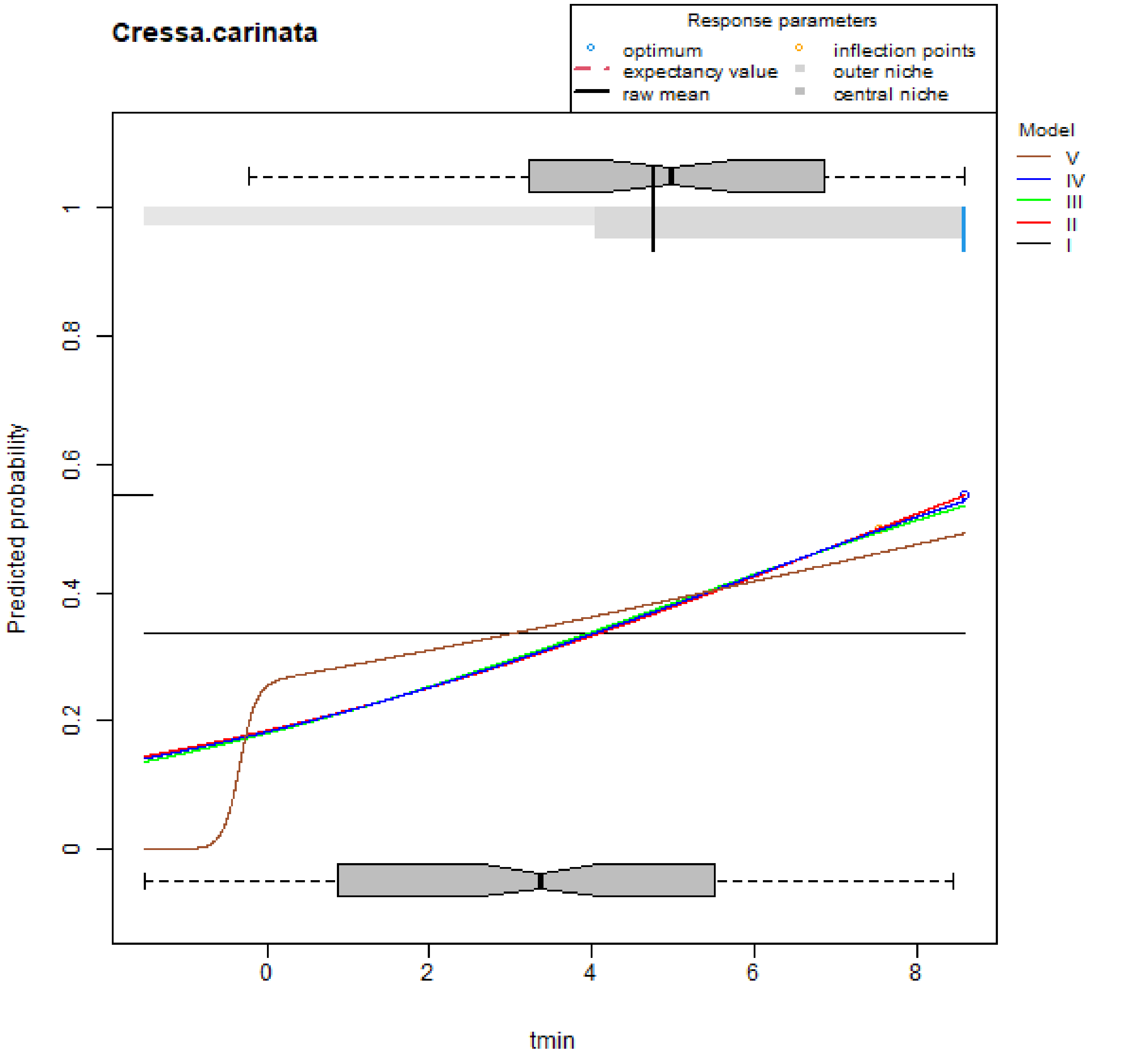

Cressa.carinata
Response parameters
optimum
expectancy value
raw mean
inflection points
outer niche
central niche
Model
V
IV
III
II
I
Predicted probability
0
0.2
0.4
0.6
0.8
1
0
2
4
6
8
tmin

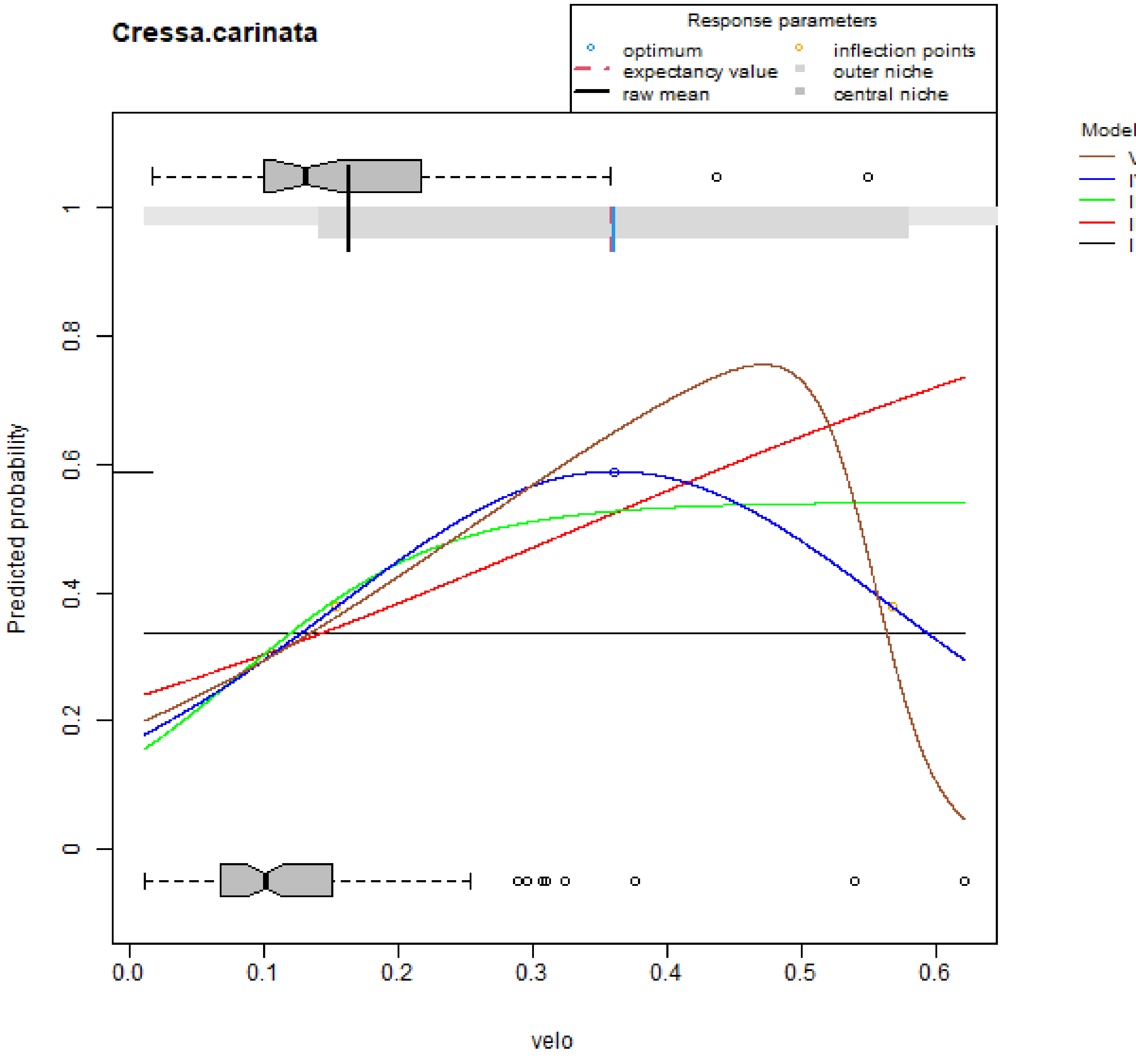

Cressa.carinata
Response parameters
optimum
expectancy value
raw mean
inflection points
outer niche
central niche
Model
Predicted probability
velo
0
0.2
0.4
0.6
0.8
1
0.0
0.1
0.2
0.3
0.4
0.5
0.6

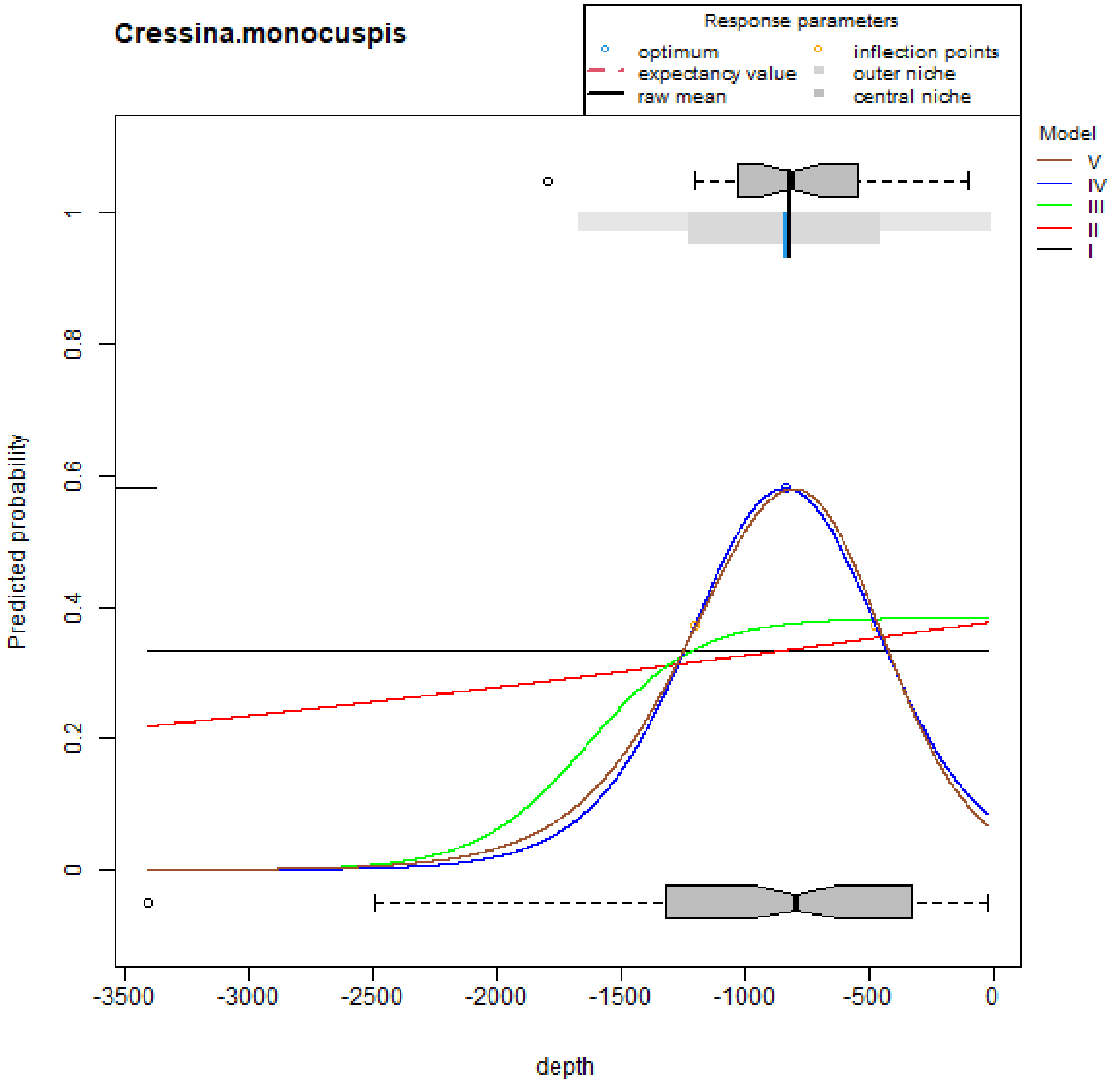

Cressina.monocuspis
Response parameters
optimum
expectancy value
raw mean
inflection points
outer niche
central niche
Model
V
IV
III
II
I
Predicted probability
0
0.2
0.4
0.6
0.8
1
-3500
-3000
-2500
-2000
-1500
-1000
-500
0
depth

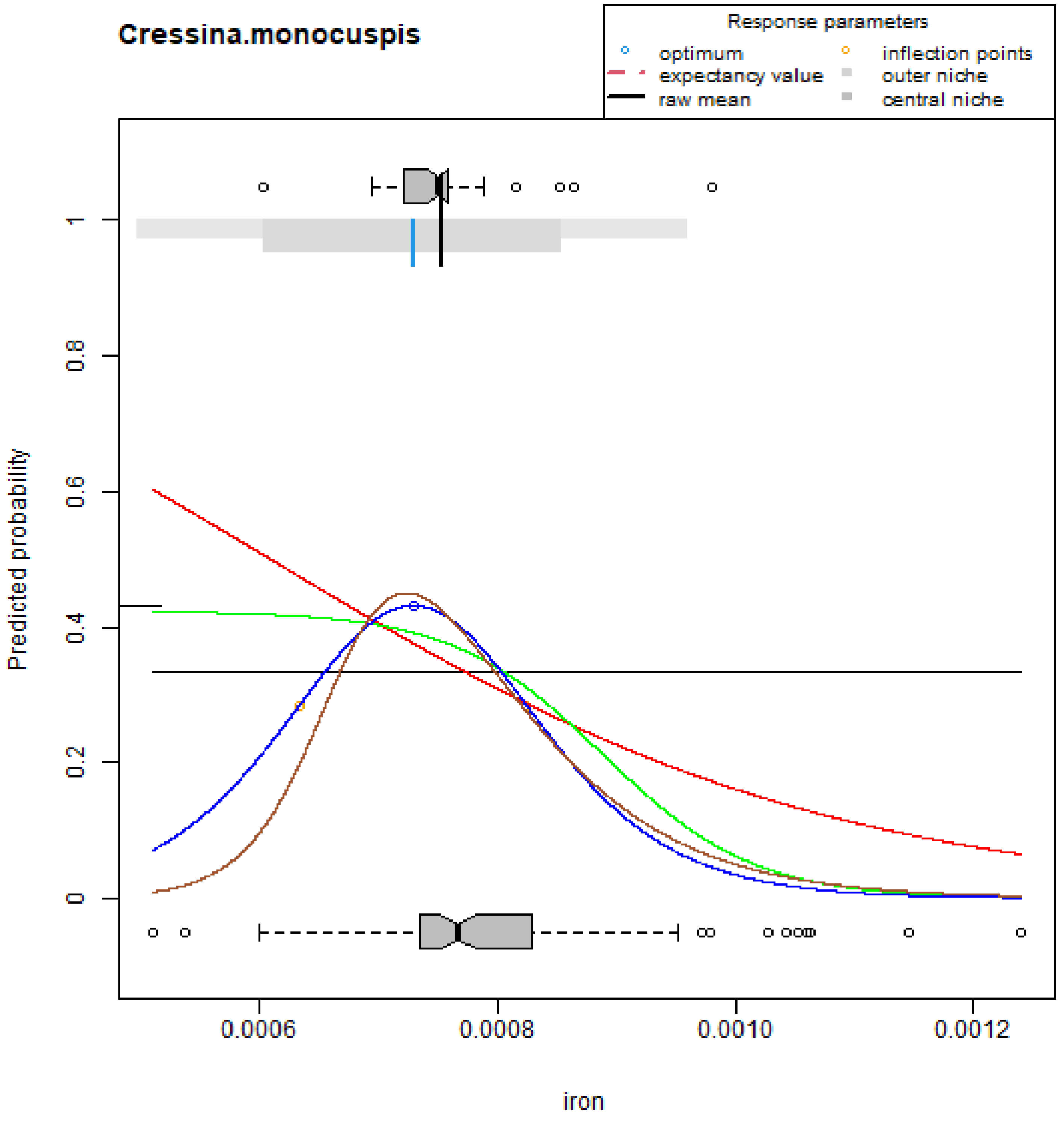

Cressina.monocuspis
Response parameters
optimum
expectancy value
raw mean
inflection points
outer niche
central niche
Predicted probability
0
0.2
0.4
0.6
0.8
1
0.0006
0.0008
0.0010
0.0012
iron

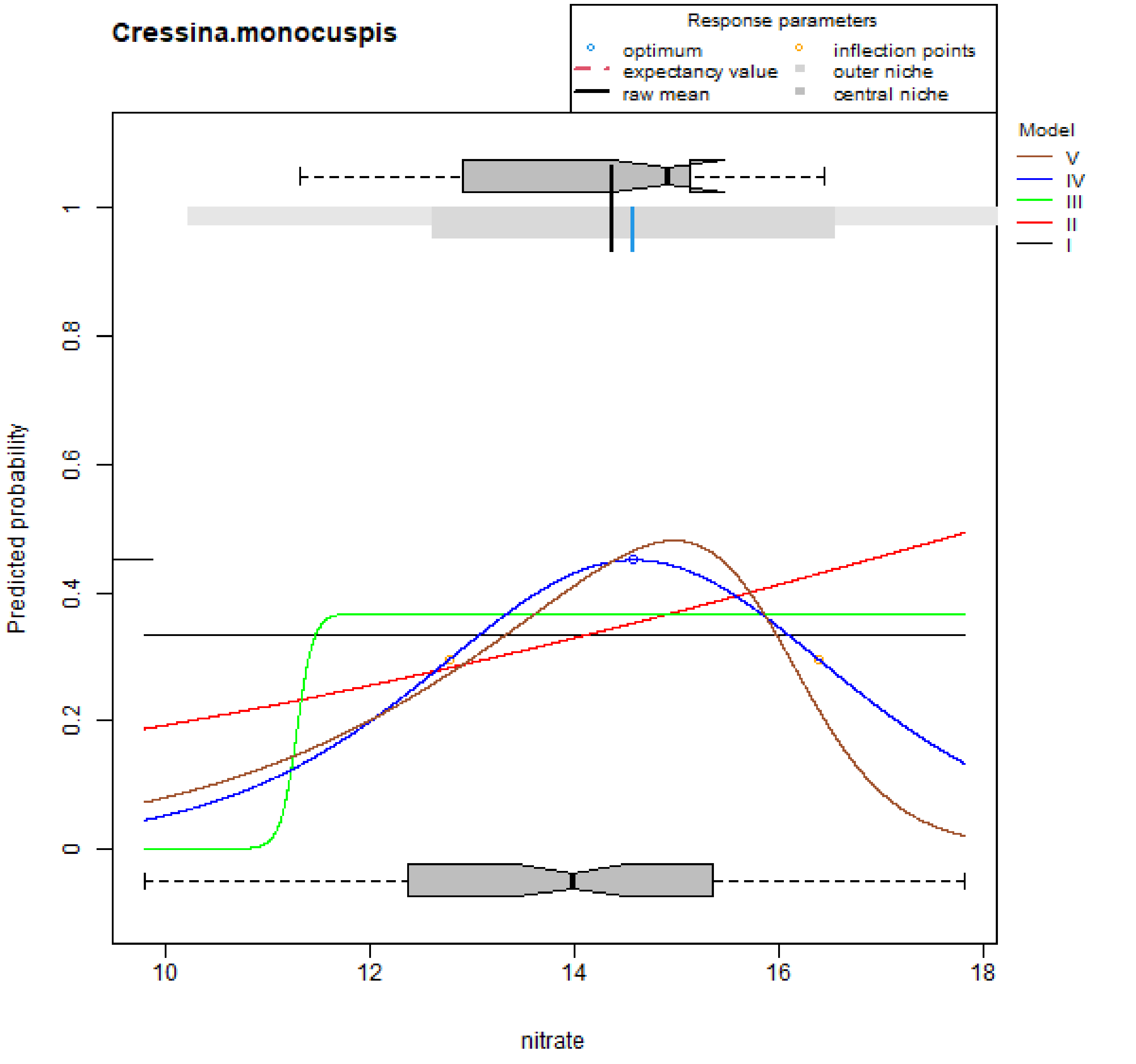
Cressina.monocuspis
Response parameters
optimum
expectancy value
raw mean
inflection points
outer niche
central niche
Model
V
IV
III
II
I
Predicted probability
0
0.2
0.4
0.6
0.8
1
10
12
14
16
18
nitrate

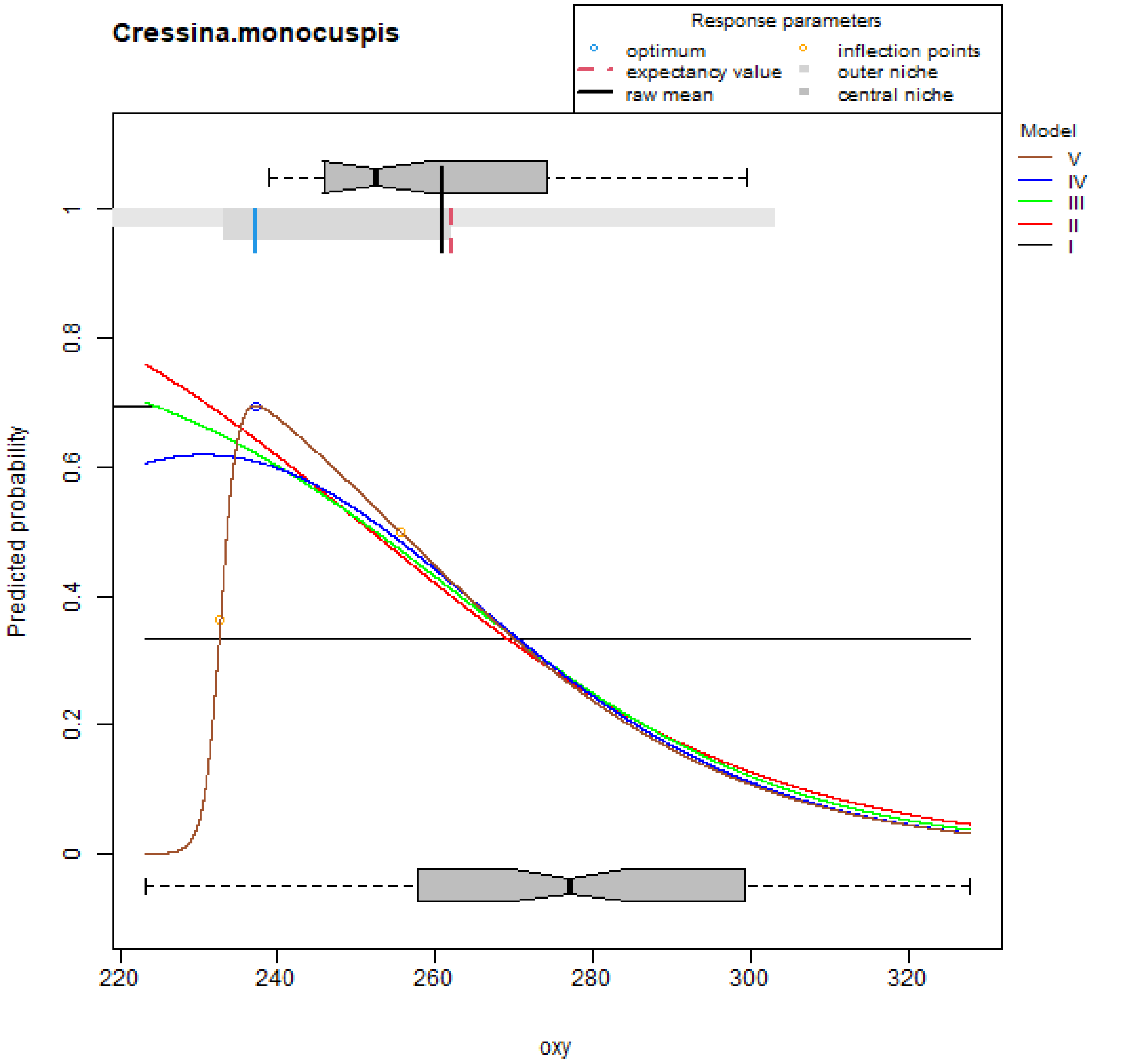

Cressina.monocuspis
Response parameters
optimum
expectancy value
raw mean
inflection points
outer niche
central niche
Model
V
IV
III
II
I
Predicted probability
0
0.2
0.4
0.6
0.8
1
220
240
260
280
300
320
oxy

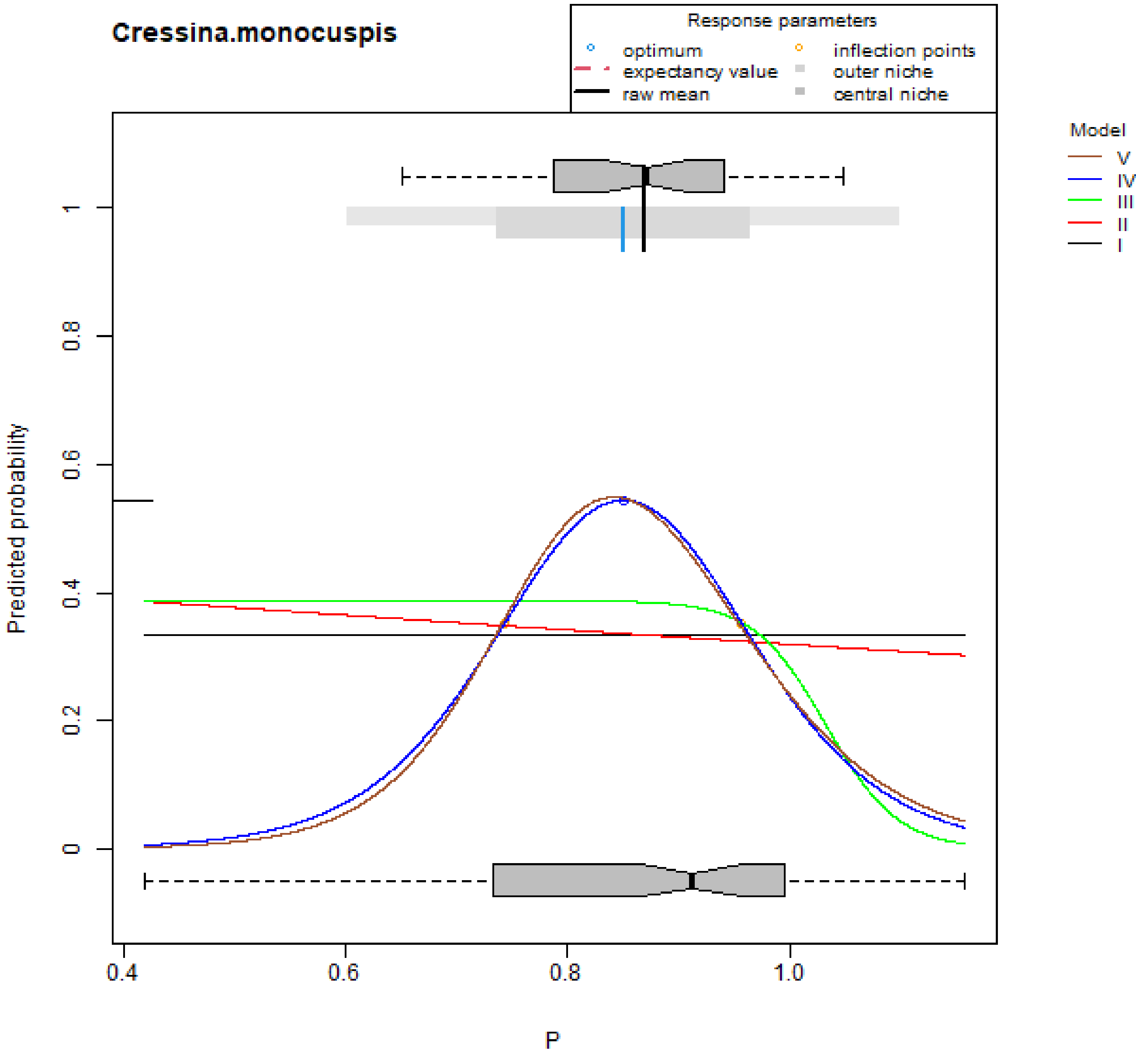
Cressina.monocuspis
Response parameters
optimum
expectancy value
raw mean
inflection points
outer niche
central niche
Model
V
IV
III
II
I
Predicted probability
0
0.2
0.4
0.6
0.8
1
0.4
0.6
0.8
1.0
P

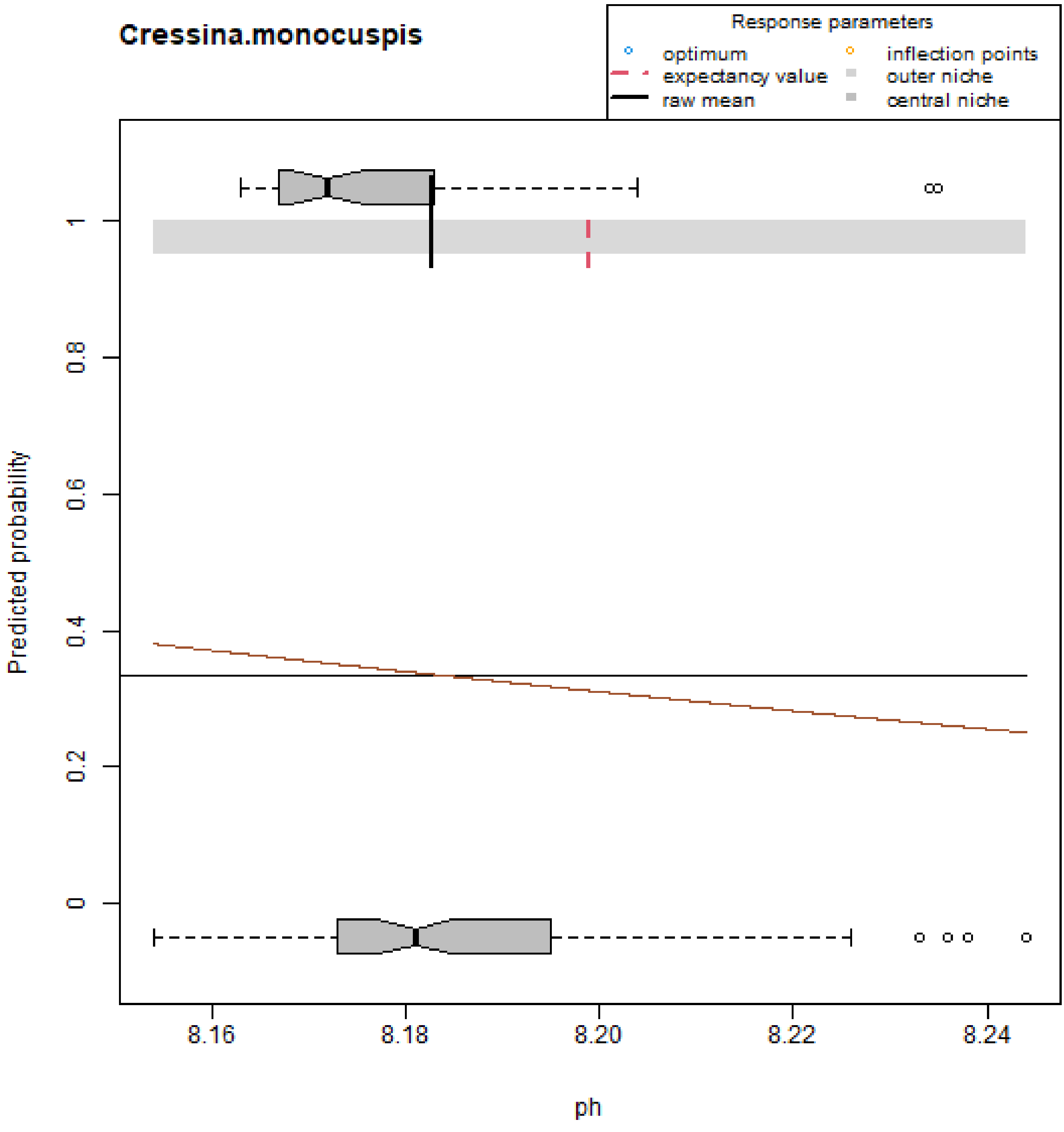

Cressina.monocuspis
Response parameters
optimum
expectancy value
raw mean
inflection points
outer niche
central niche
Predicted probability
0
0.2
0.4
0.6
0.8
1
8.16
8.18
8.20
8.22
8.24
ph

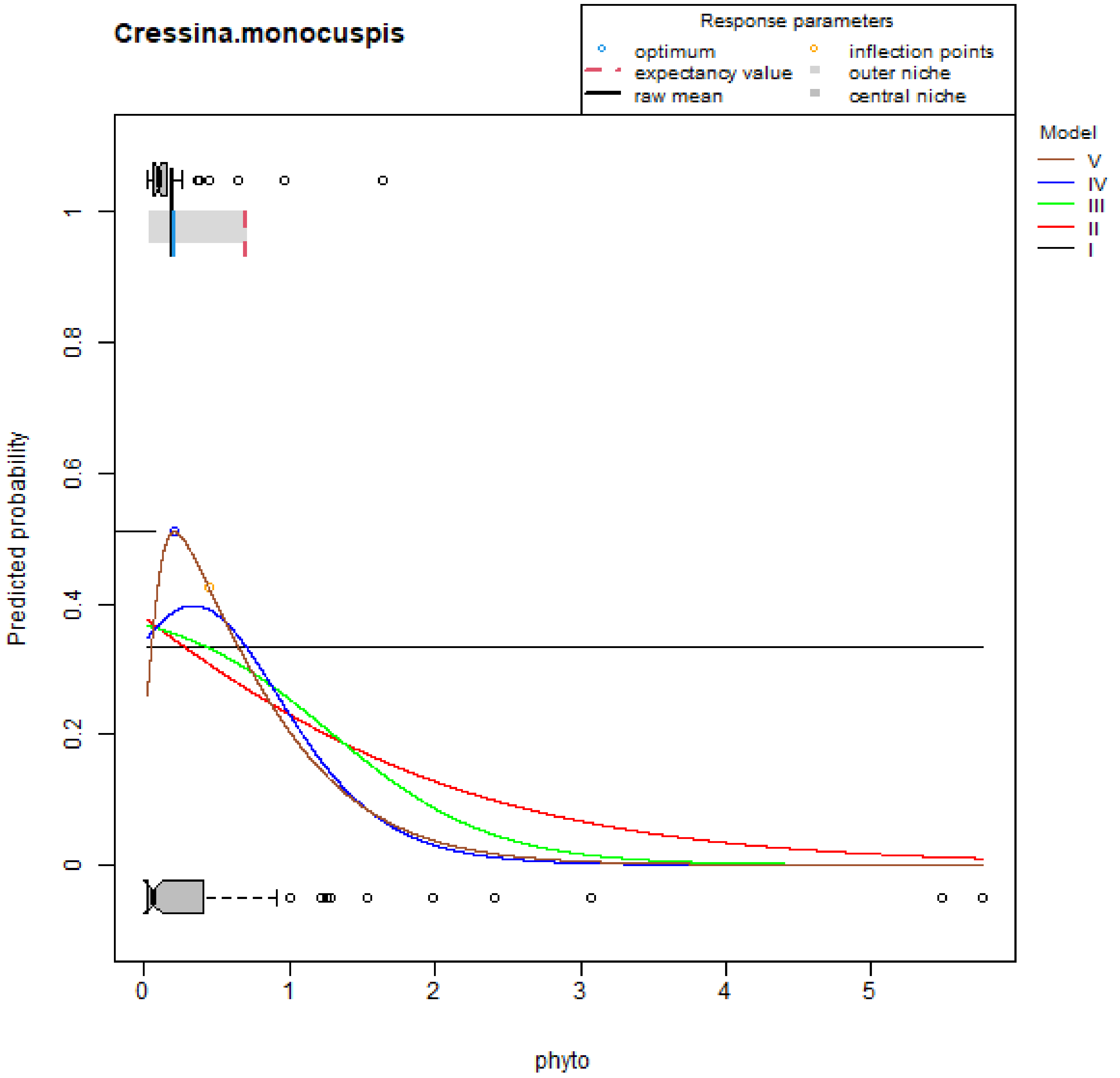

Cressina.monocuspis
Response parameters
optimum
expectancy value
raw mean
inflection points
outer niche
central niche
Model
V
IV
III
II
I
Predicted probability
0
0.2
0.4
0.6
0.8
1
0
1
2
3
4
5
phyto

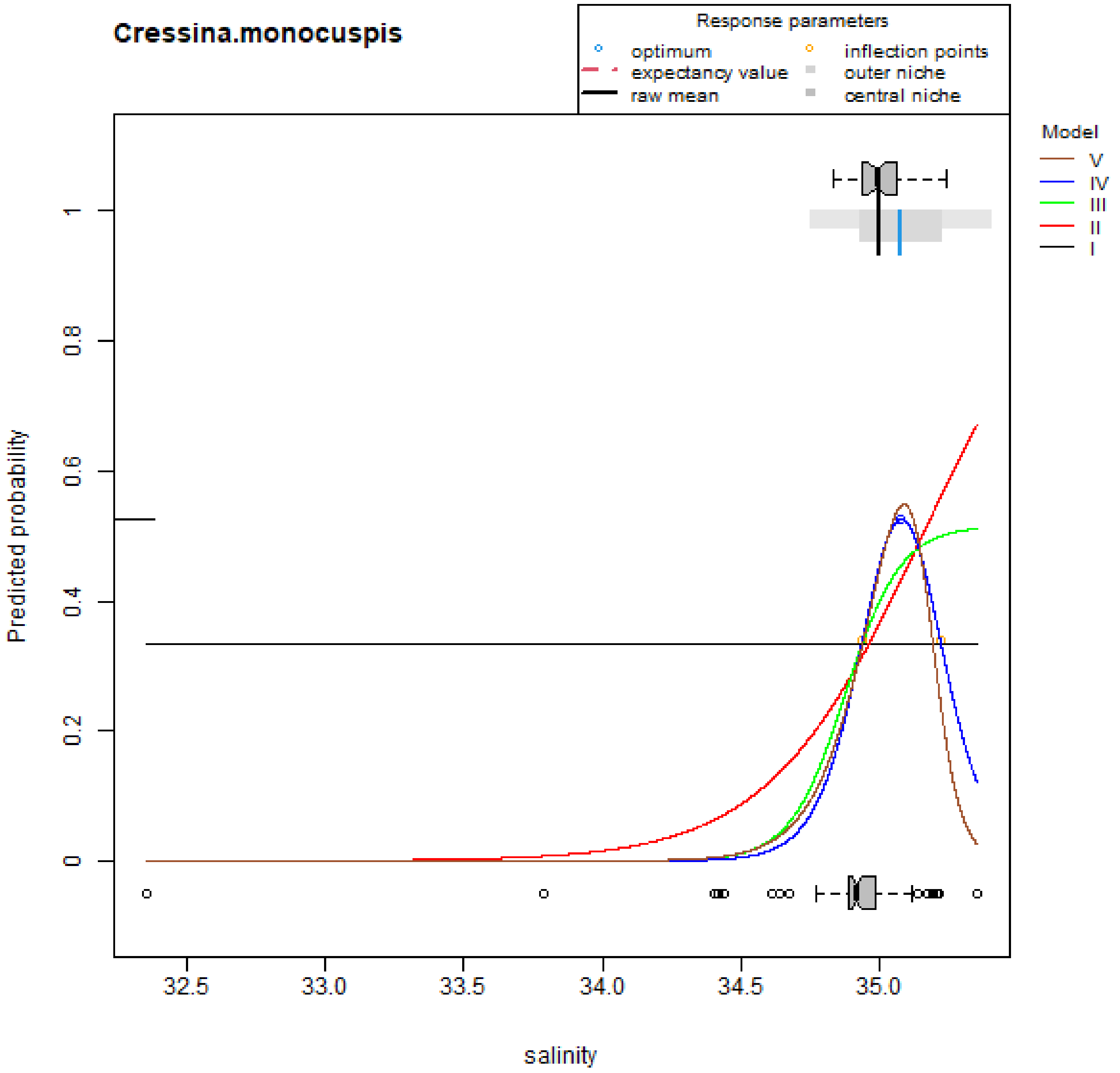

Cressina.monocuspis
Response parameters
optimum
expectancy value
raw mean
inflection points
outer niche
central niche
Model
V
IV
III
II
I
Predicted probability
0
0.2
0.4
0.6
0.8
1
32.5
33.0
33.5
34.0
34.5
35.0
salinity

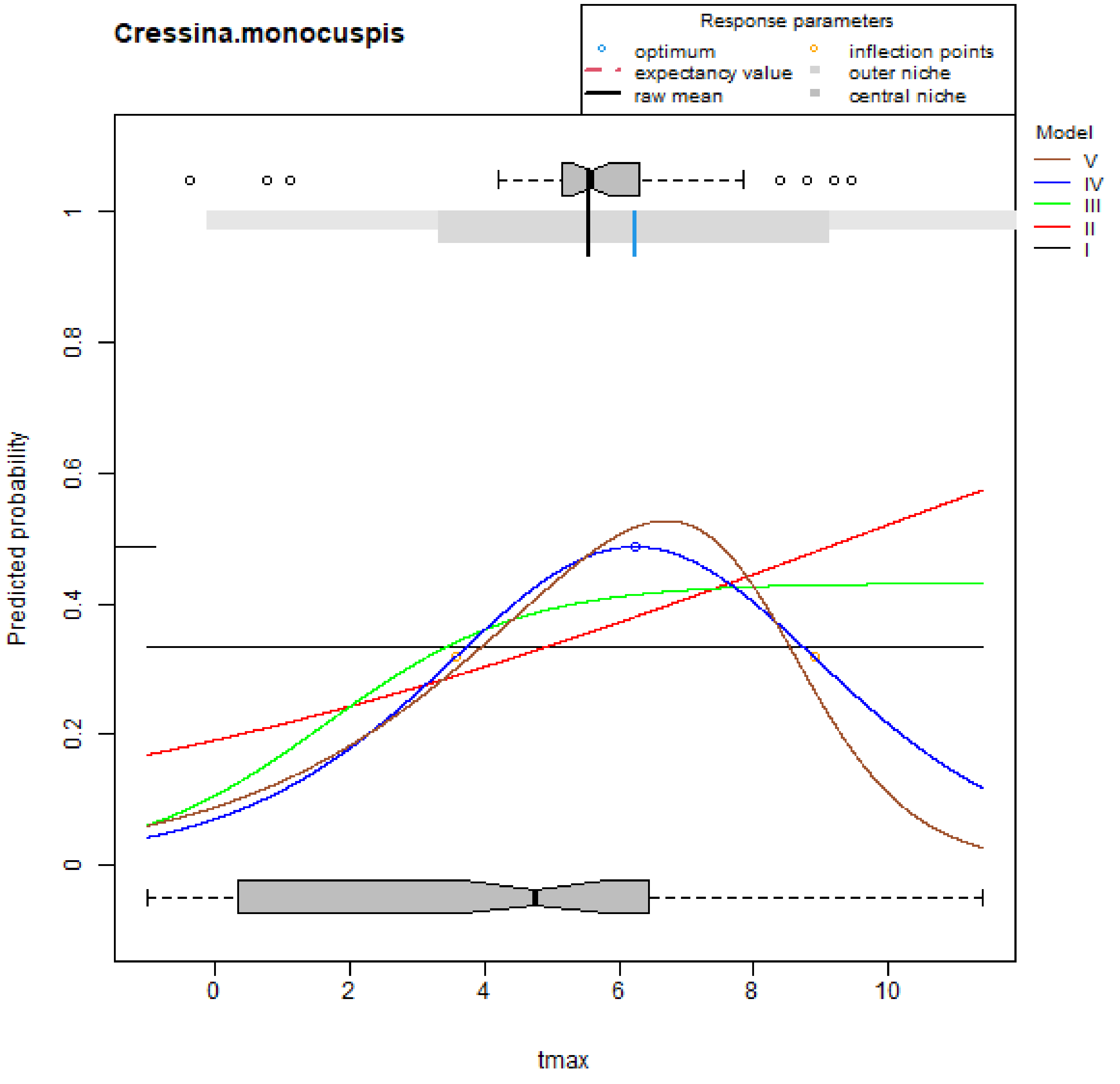
Cressina.monocuspis
Response parameters
optimum
expectancy value
raw mean
inflection points
outer niche
central niche
Model
V
IV
III
II
I
Predicted probability
0
0.2
0.4
0.6
0.8
1
0
2
4
6
8
10
tmax

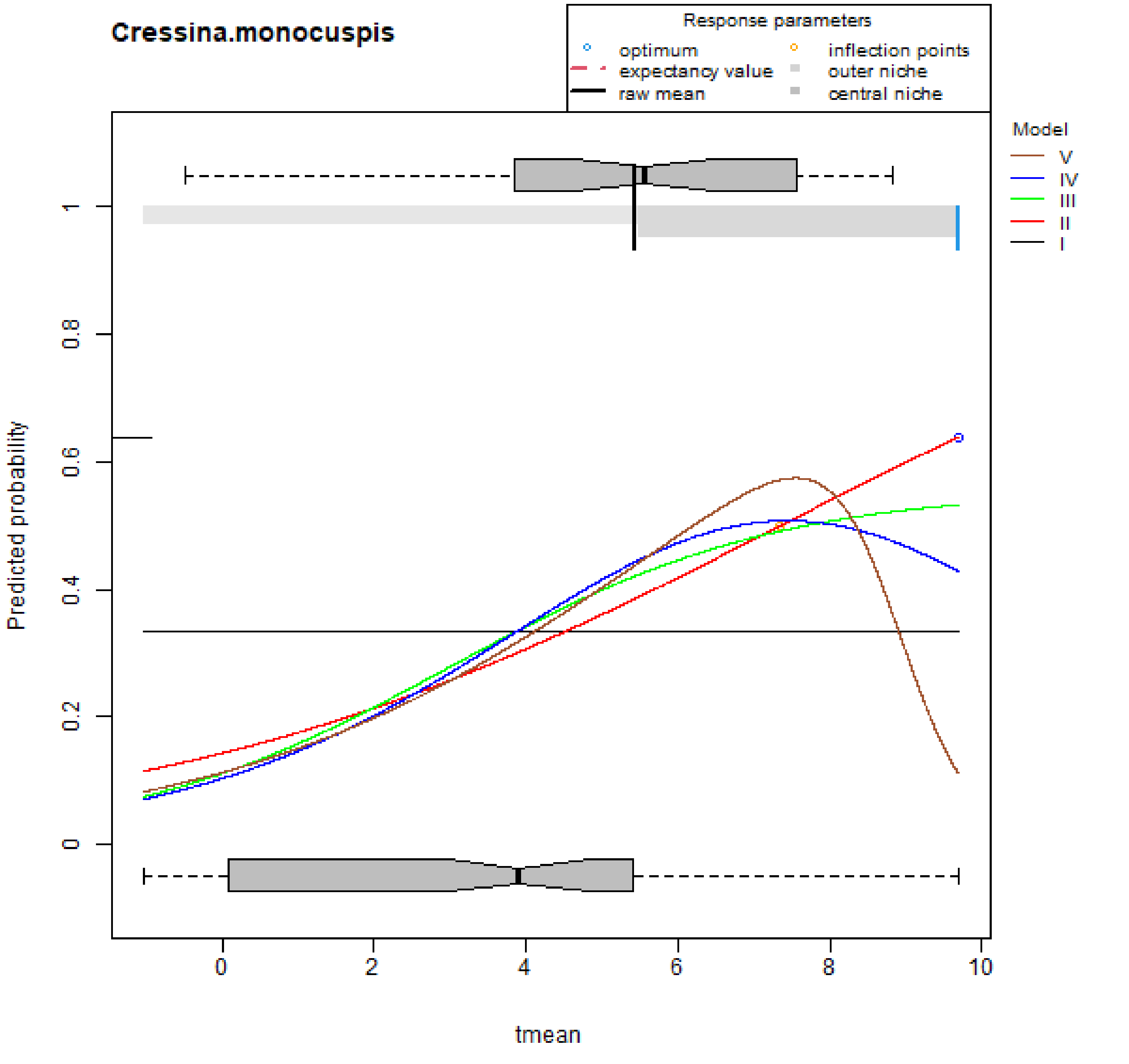

Cressina.monocuspis
Response parameters
optimum
expectancy value
raw mean
inflection points
outer niche
central niche
Model
V
IV
III
II
I
Predicted probability
0
0.2
0.4
0.6
0.8
1
0
2
4
6
8
10
tmean

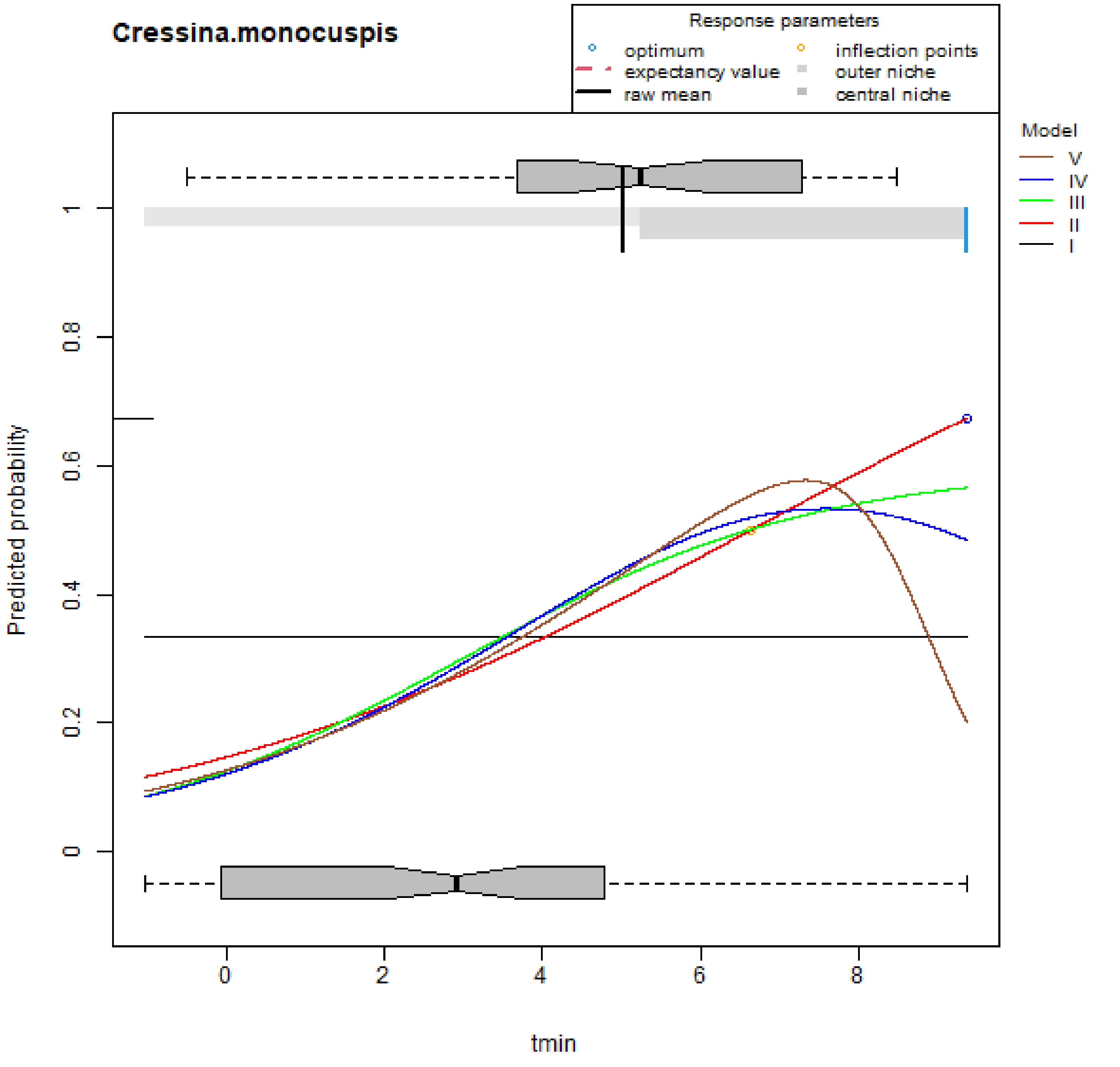

Cressina.monocuspis
Response parameters
optimum
expectancy value
raw mean
inflection points
outer niche
central niche
Model
V
IV
III
II
I
Predicted probability
0
0.2
0.4
0.6
0.8
1
0
2
4
6
8
tmin

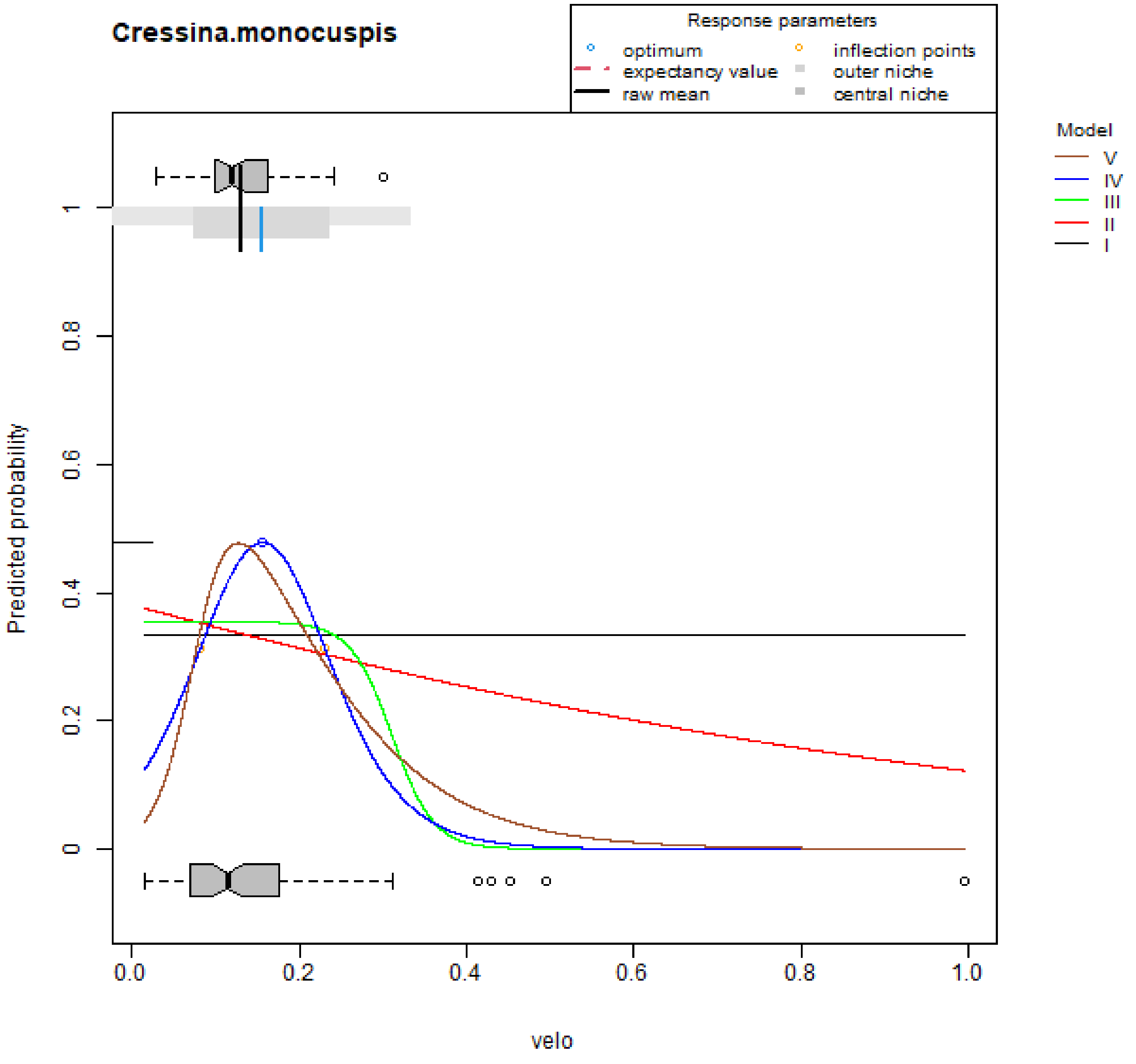

Cressina.monocuspis
Response parameters
optimum
inflection points
expectancy value
outer niche
raw mean
central niche
Model
V
IV
III
II
I
Predicted probability
0
0.2
0.4
0.6
0.8
1
0.0
0.2
0.4
0.6
0.8
1.0
velo

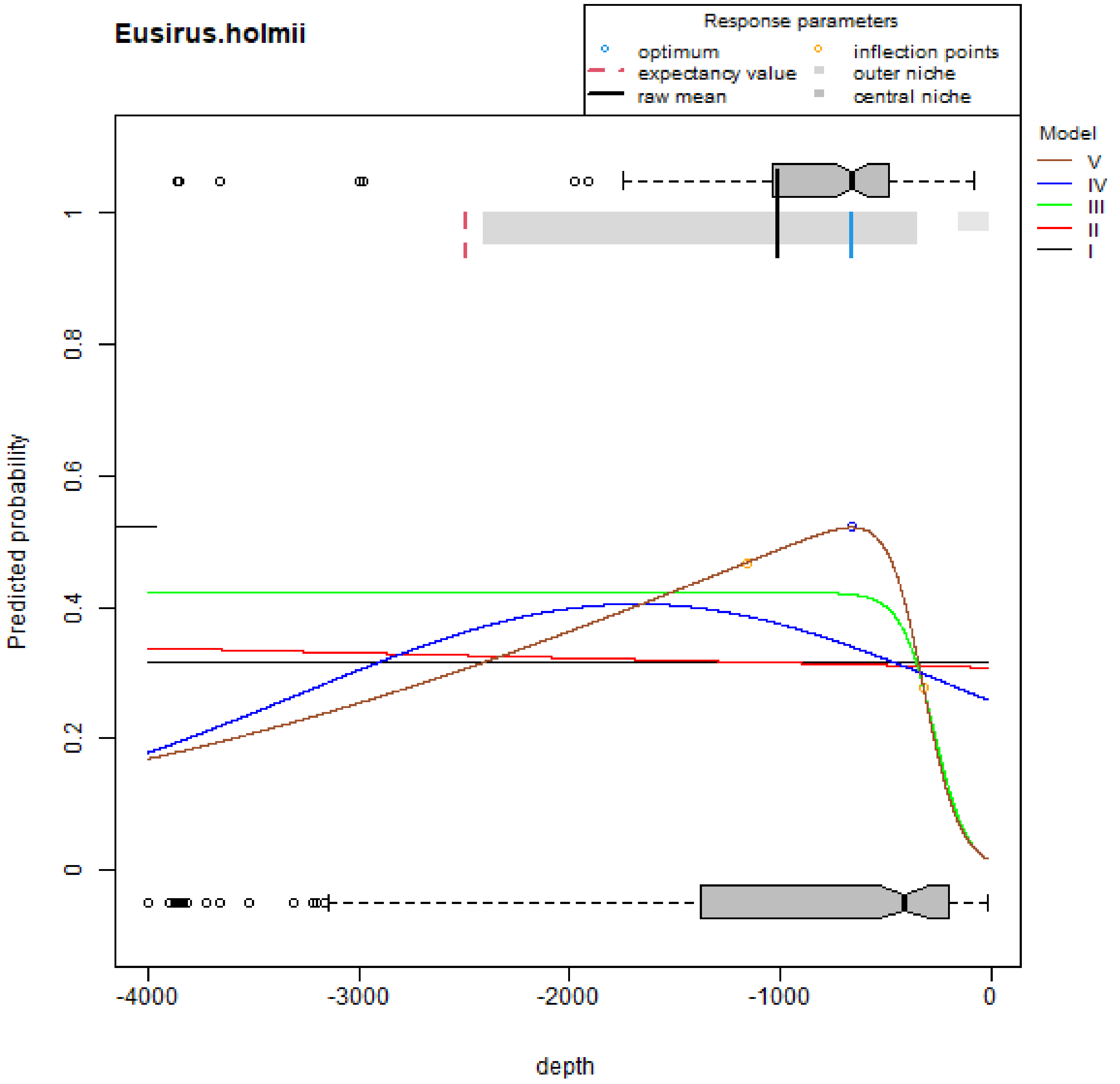
Eusirus.holmii
Response parameters
optimum
expectancy value
raw mean
inflection points
outer niche
central niche
Model
V
IV
III
II
I
Predicted probability
0
0.2
0.4
0.6
0.8
1
-4000
-3000
-2000
-1000
0
depth

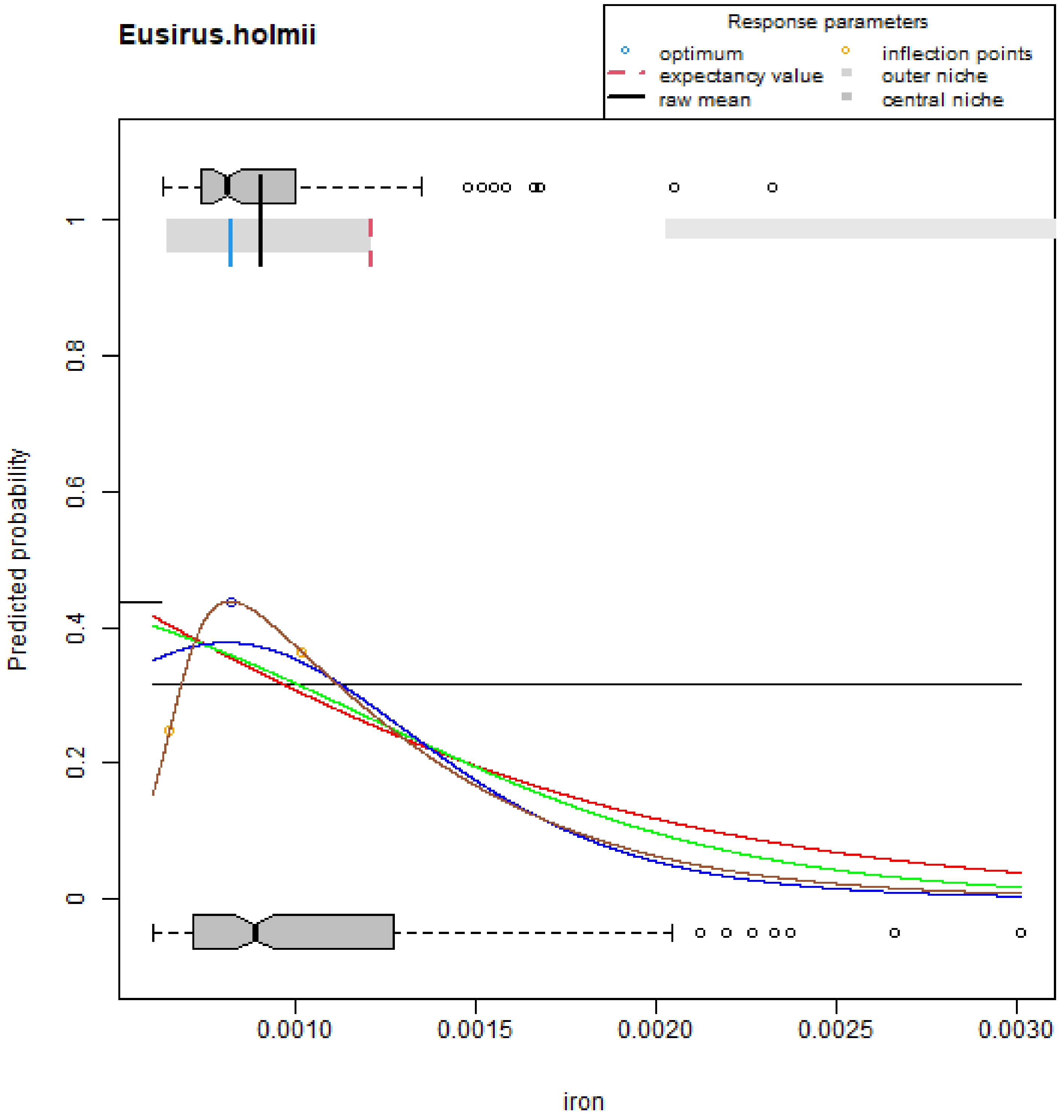

Eusirus.holmii
Response parameters
optimum
expectancy value
raw mean
inflection points
outer niche
central niche
Predicted probability
0
0.2
0.4
0.6
0.8
1
0.0010
0.0015
0.0020
0.0025
0.0030
iron

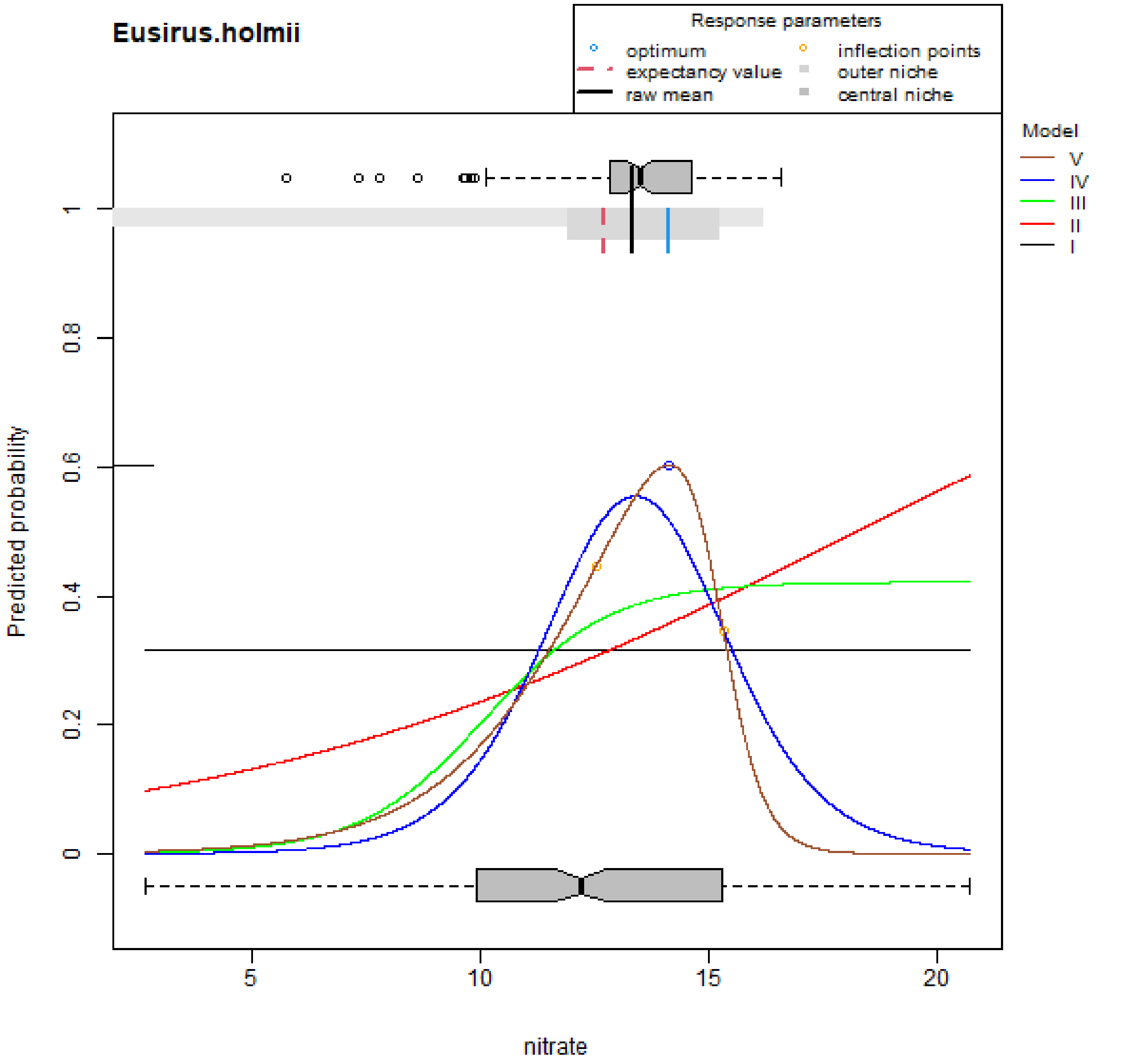

Eusirus.holmii
Response parameters
optimum
expectancy value
raw mean
inflection points
outer niche
central niche
Model
V
IV
III
II
I
Predicted probability
0
0.2
0.4
0.6
0.8
1
5
10
15
20
nitrate

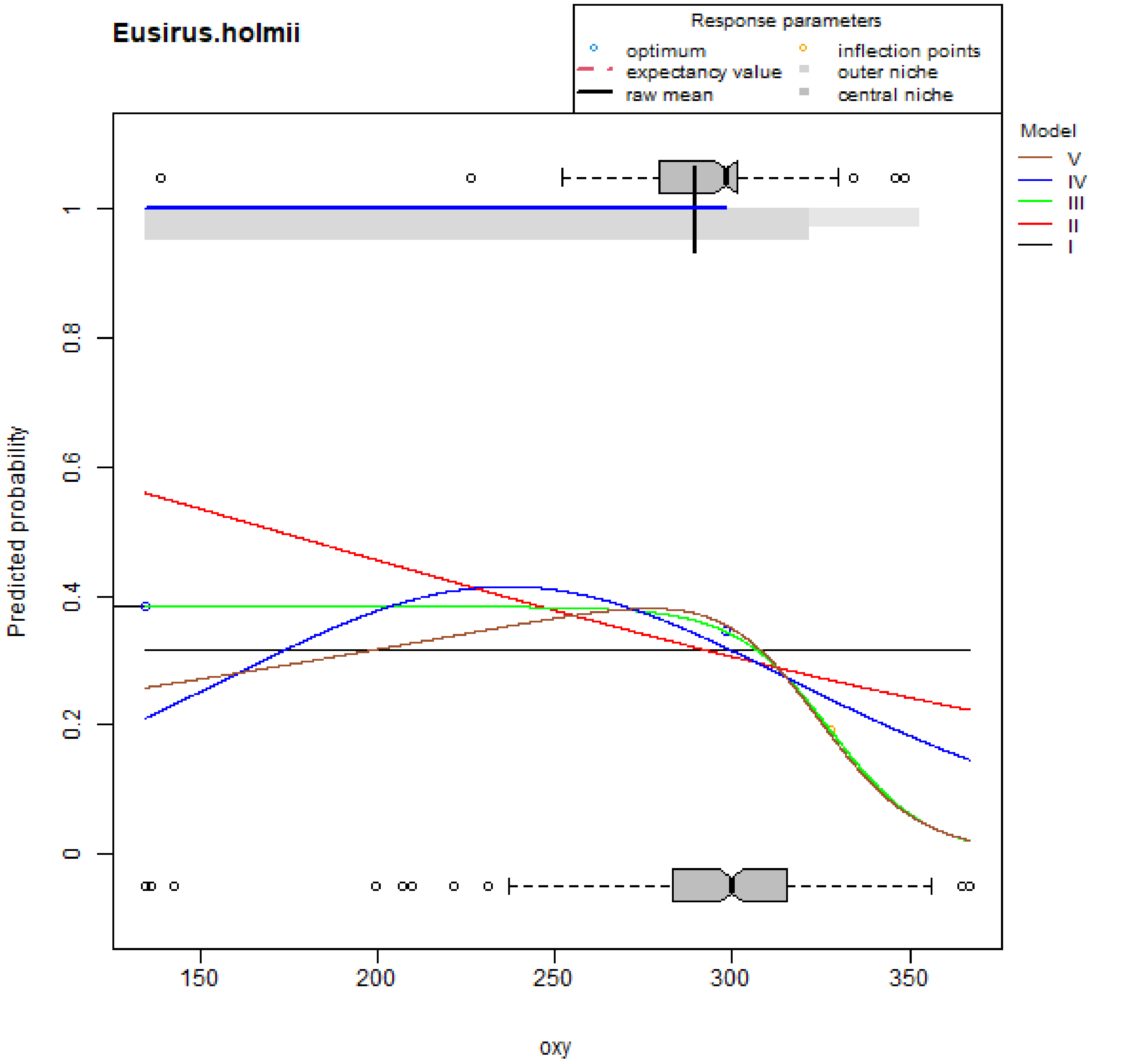
Eusirus.holmii
Response parameters
optimum
expectancy value
raw mean
inflection points
outer niche
central niche
Model
V
IV
III
II
I
Predicted probability
0
0.2
0.4
0.6
0.8
1
150
200
250
300
350
oxy

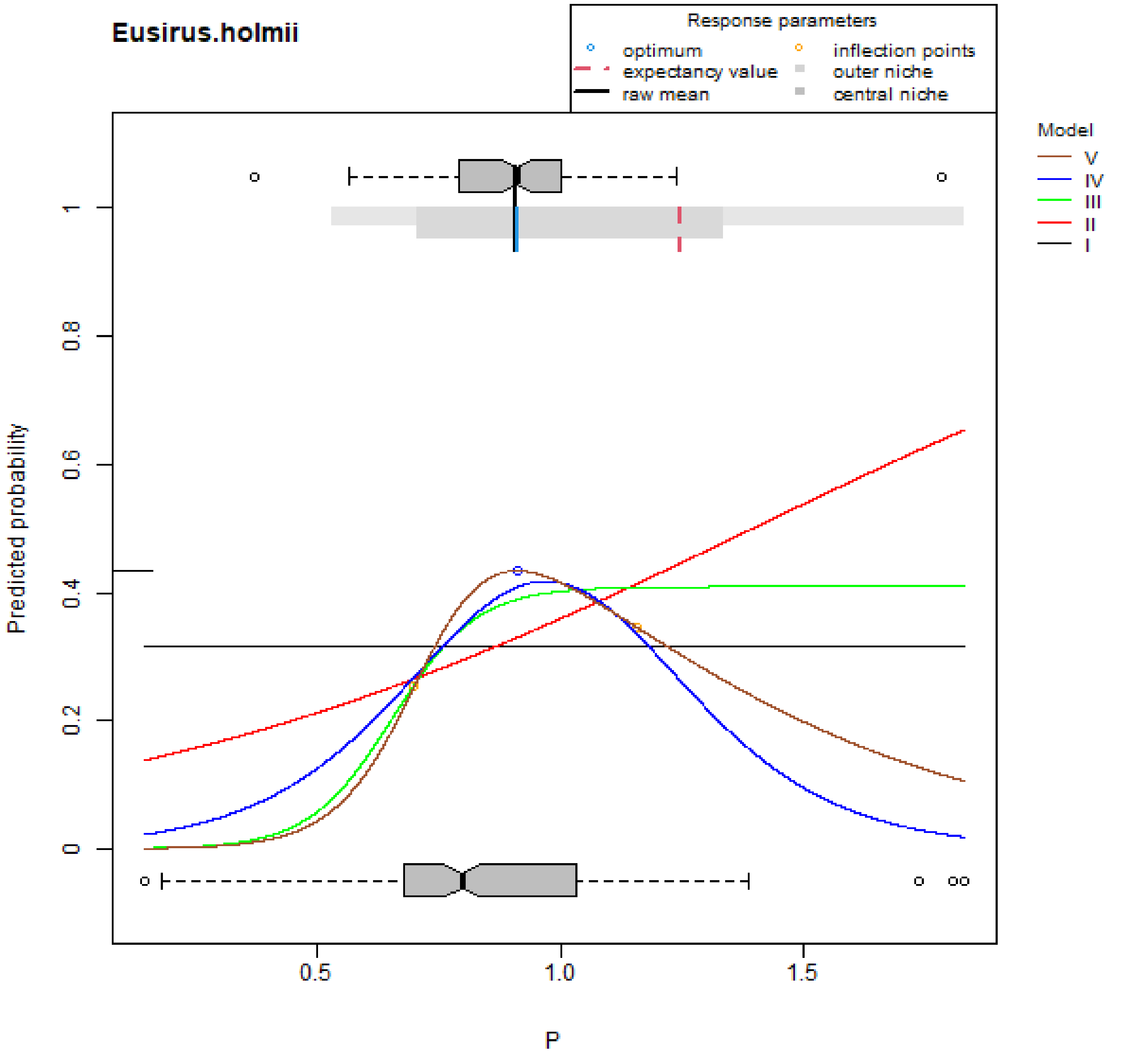

Eusirus.holmii
Response parameters
optimum
expectancy value
raw mean
inflection points
outer niche
central niche
Model
V
IV
III
II
I
Predicted probability
0
0.2
0.4
0.6
0.8
1
0.5
1.0
1.5
P

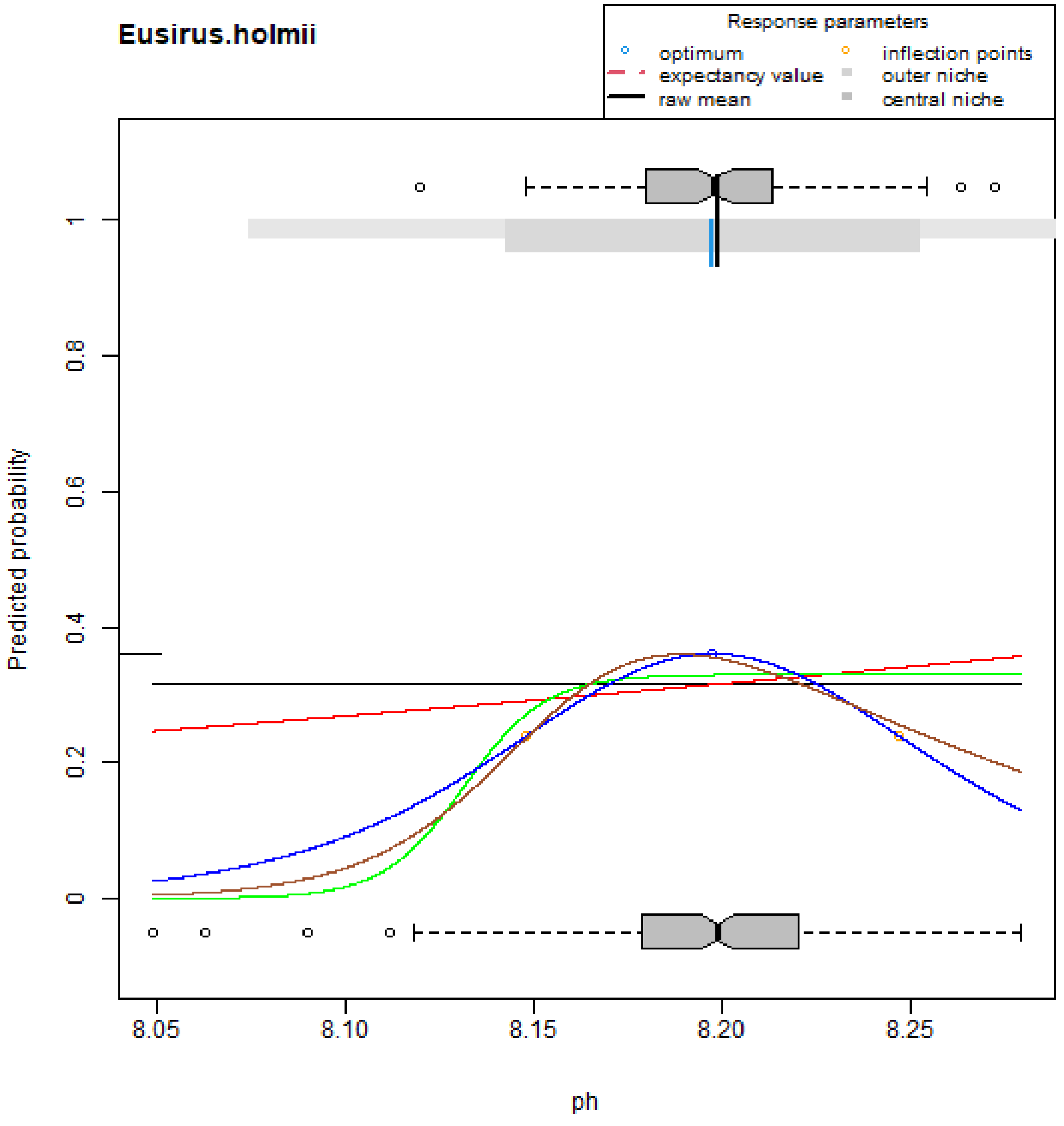
Eusirus.holmii
Response parameters
optimum
expectancy value
raw mean
inflection points
outer niche
central niche
Predicted probability
0
0.2
0.4
0.6
0.8
1
8.05
8.10
8.15
8.20
8.25
ph

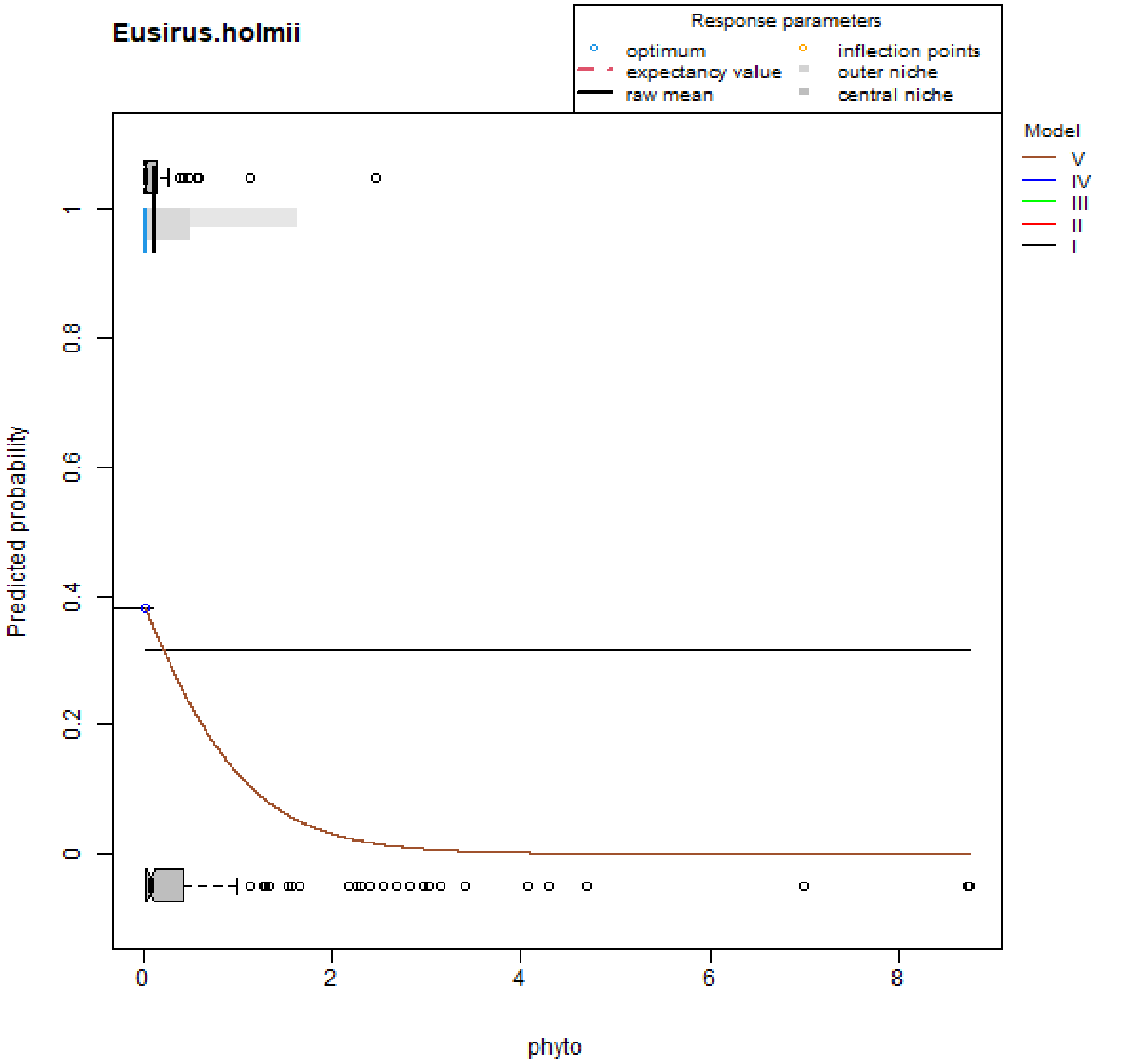
Eusirus.holmii
Response parameters
optimum
expectancy value
raw mean
inflection points
outer niche
central niche
Model
V
IV
III
II
I
Predicted probability
0
0.2
0.4
0.6
0.8
1
0
2
4
6
8
phyto

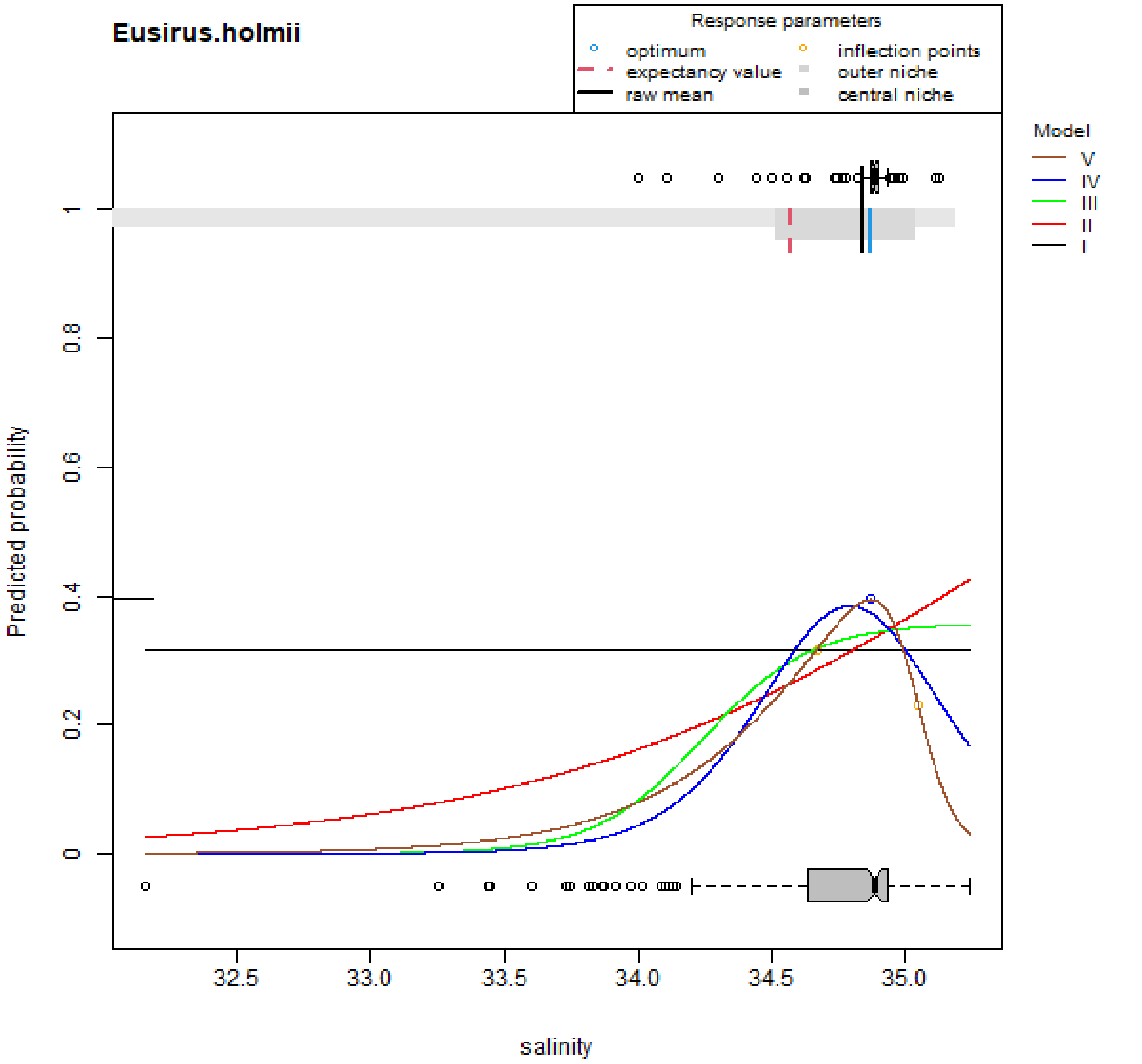

Eusirus.holmii
Response parameters
optimum
expectancy value
raw mean
inflection points
outer niche
central niche
Model
V
IV
III
II
I
Predicted probability
0
0.2
0.4
0.6
0.8
1
32.5
33.0
33.5
34.0
34.5
35.0
salinity

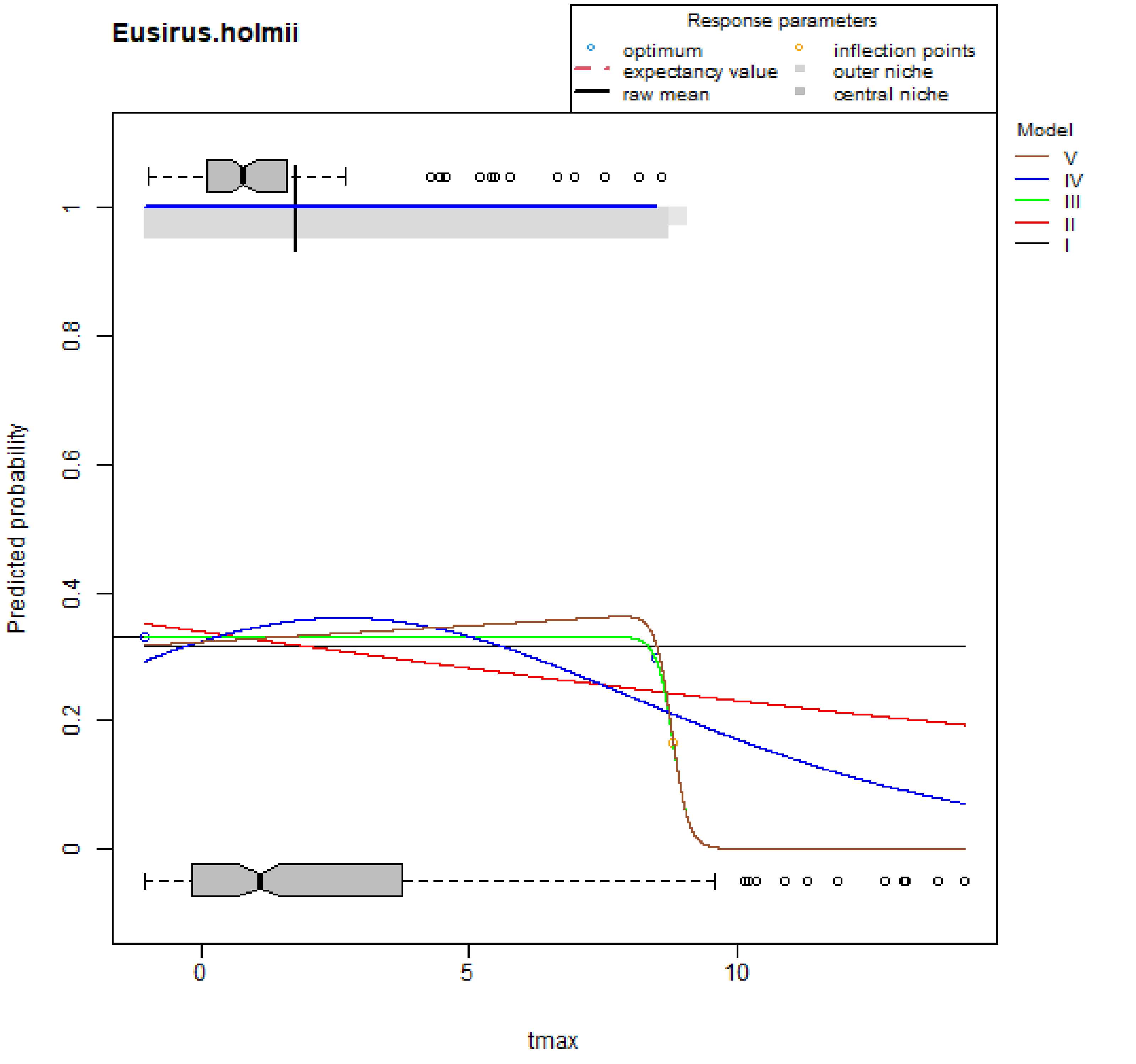

Eusirus.holmii
Response parameters
optimum
expectancy value
raw mean
inflection points
outer niche
central niche
Model
V
IV
III
II
I
Predicted probability
0
0.2
0.4
0.6
0.8
1
0
5
10
tmax

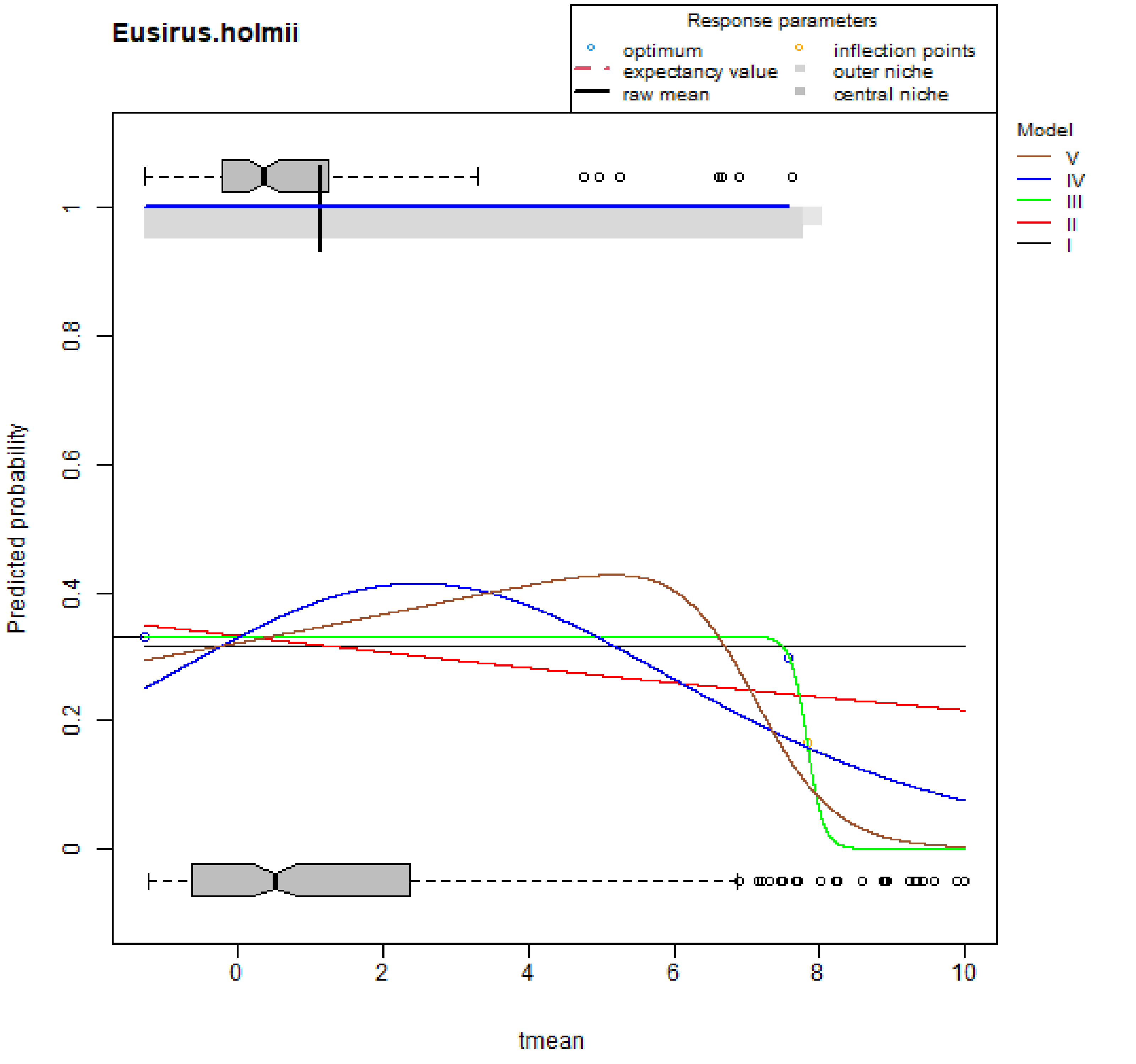
Eusirus.holmii
Response parameters
optimum
expectancy value
raw mean
inflection points
outer niche
central niche
Model
V
IV
III
II
I
Predicted probability
0
0.2
0.4
0.6
0.8
1
0
2
4
6
8
10
tmean

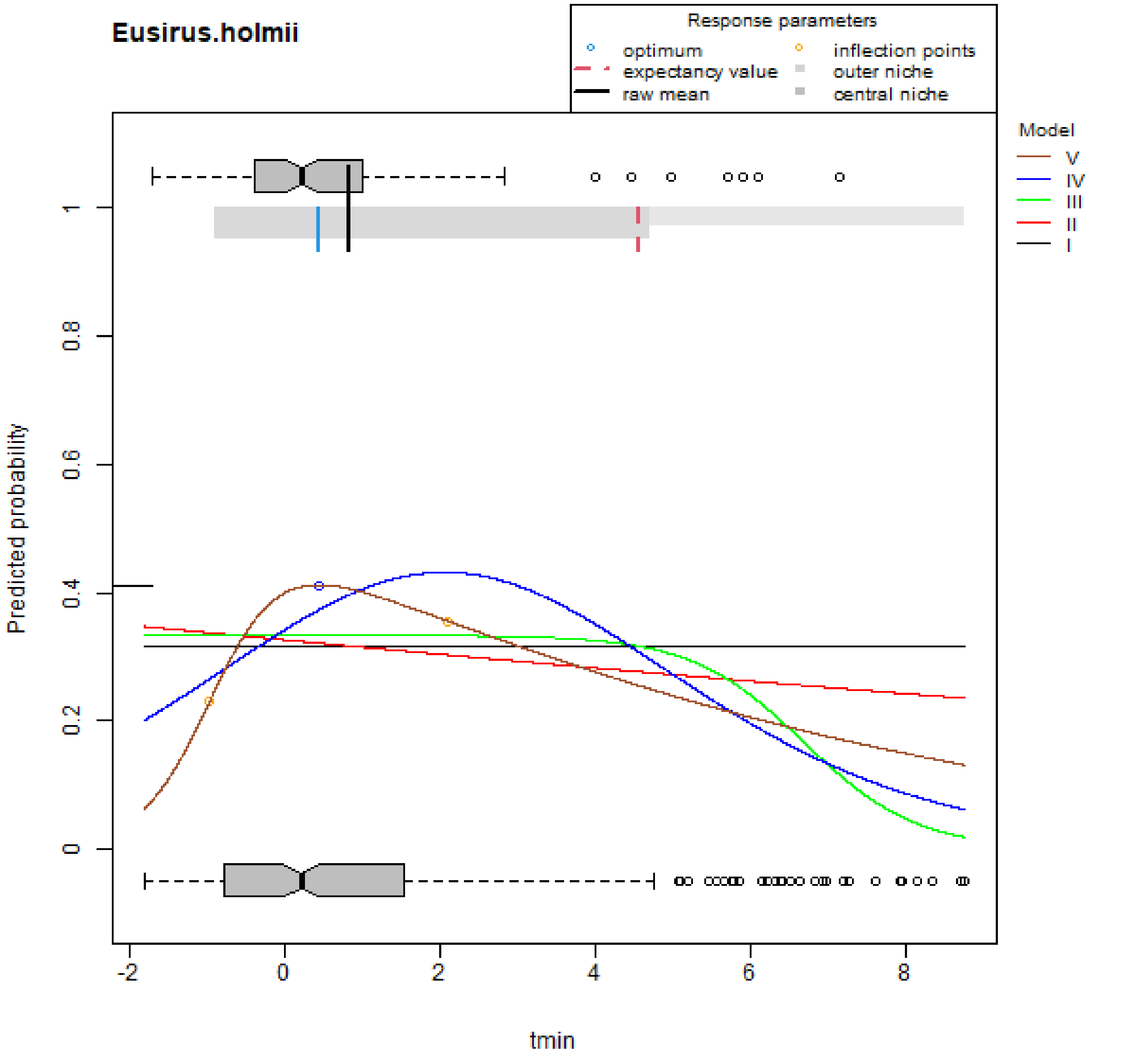
Eusirus.holmii
Response parameters
optimum
expectancy value
raw mean
inflection points
outer niche
central niche
Model
V
IV
III
II
I
Predicted probability
0
0.2
0.4
0.6
0.8
1
-2
0
2
4
6
8
tmin

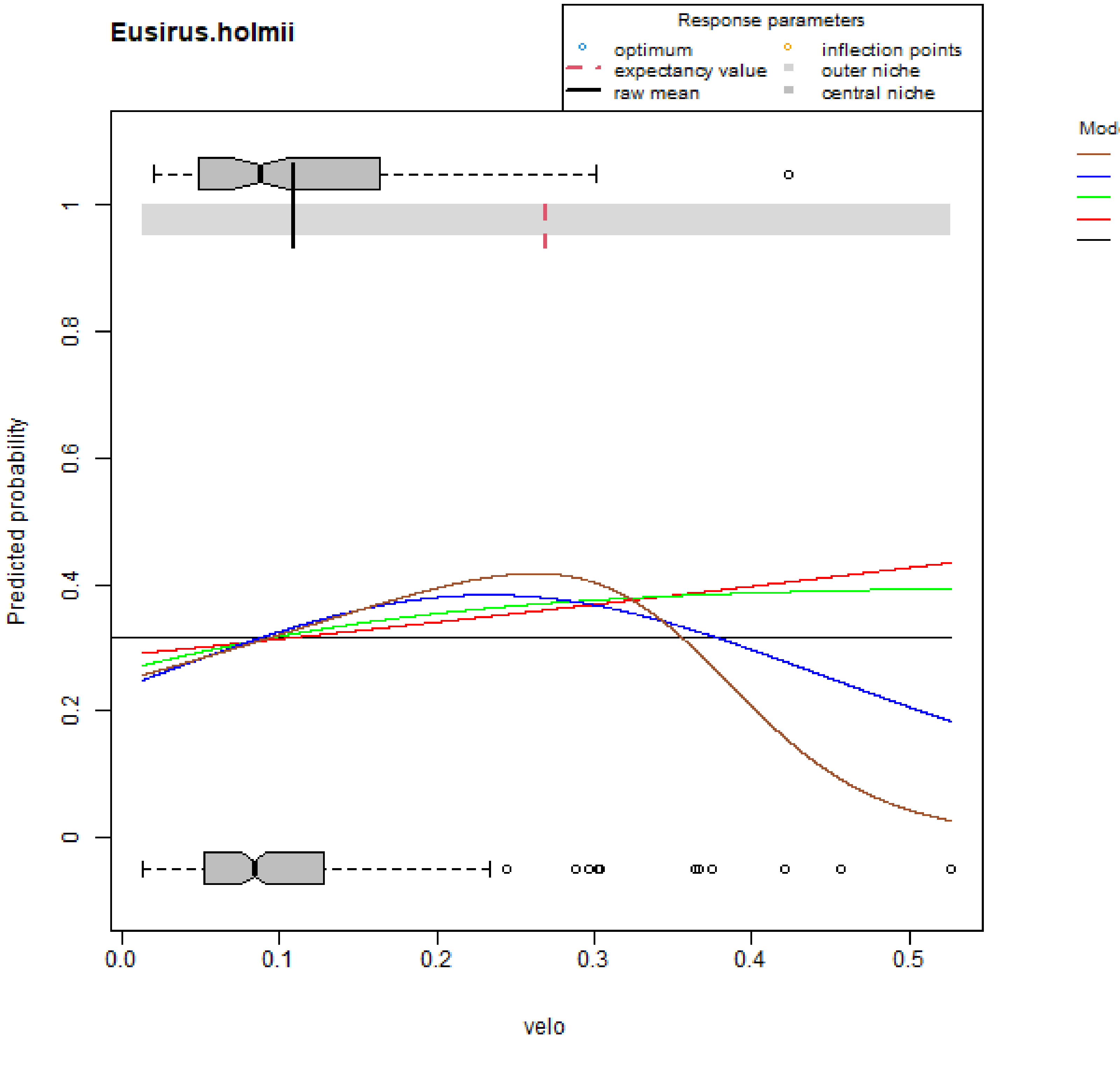
Eusirus.holmii
Response parameters
optimum
expectancy value
raw mean
inflection points
outer niche
central niche
Predicted probability
0
0.2
0.4
0.6
0.8
1
0.0
0.1
0.2
0.3
0.4
0.5
velo

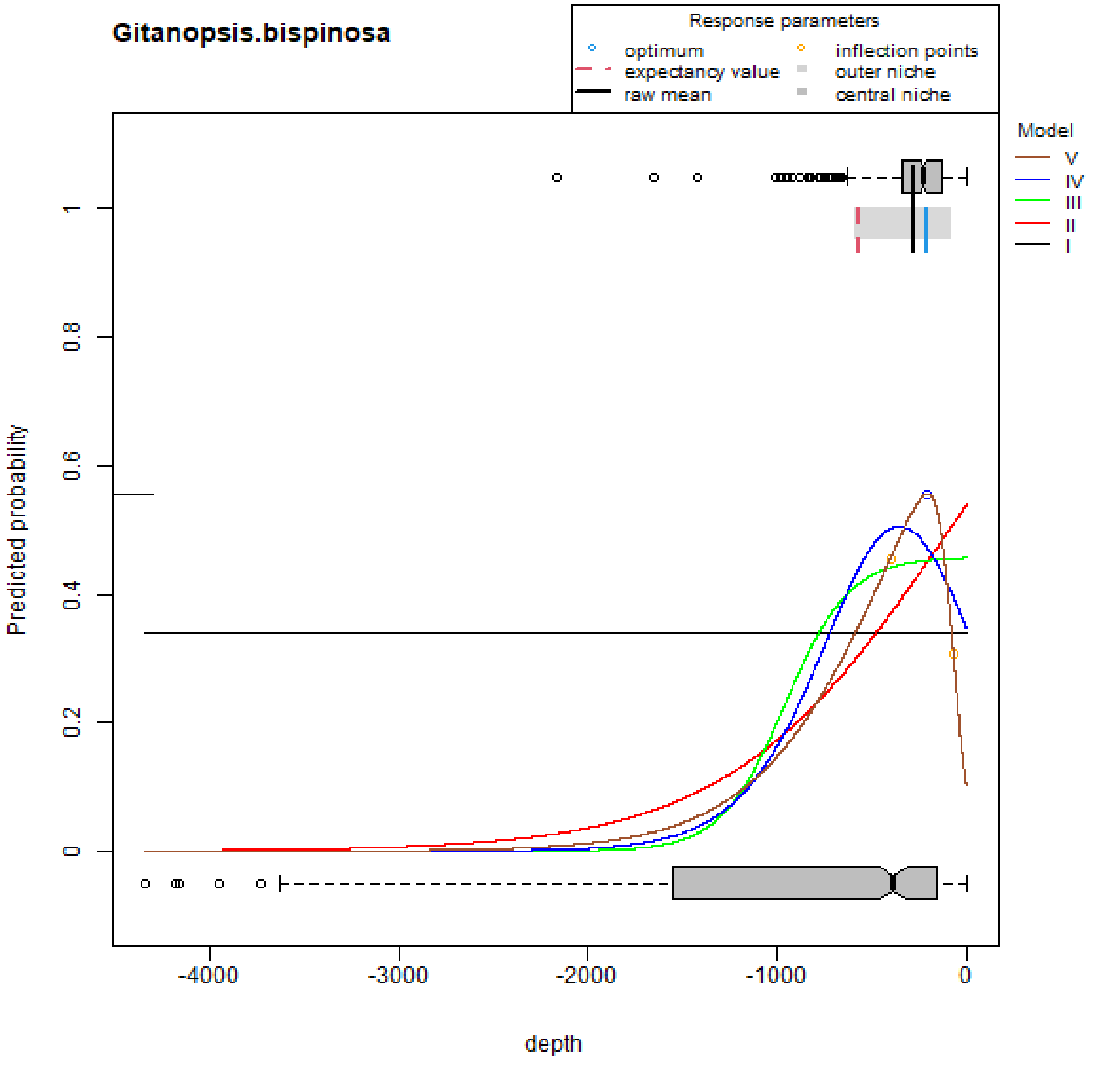
Gitanopsis.bispinosa
Response parameters
optimum
expectancy value
raw mean
inflection points
outer niche
central niche
Model
V
IV
III
II
I
Predicted probability
0
0.2
0.4
0.6
0.8
1
-4000
-3000
-2000
-1000
0
depth

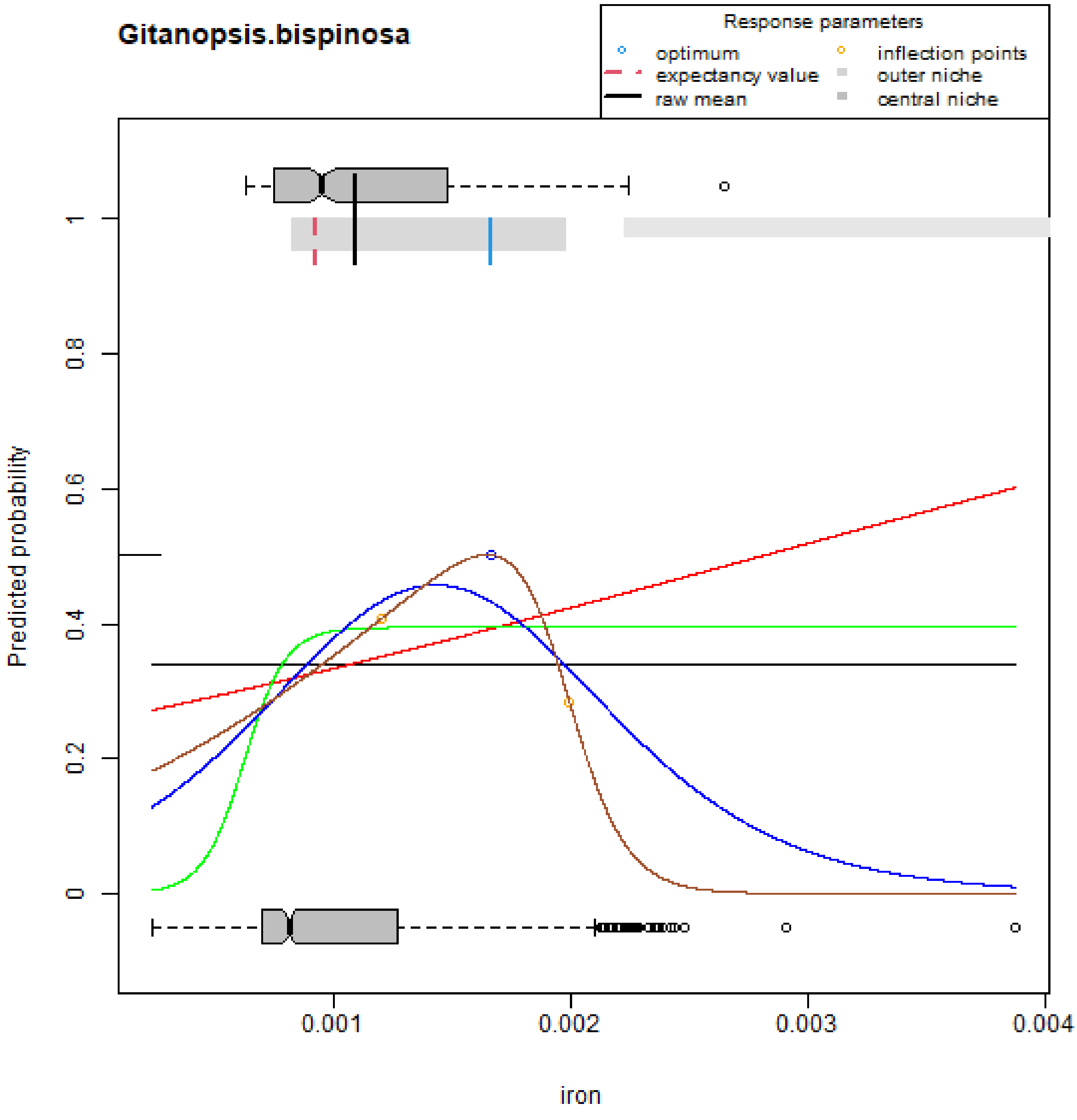

Gitanopsis.bispinosa
Response parameters
optimum
expectancy value
raw mean
inflection points
outer niche
central niche
Predicted probability
0
0.2
0.4
0.6
0.8
1
0.001
0.002
0.003
0.004
iron

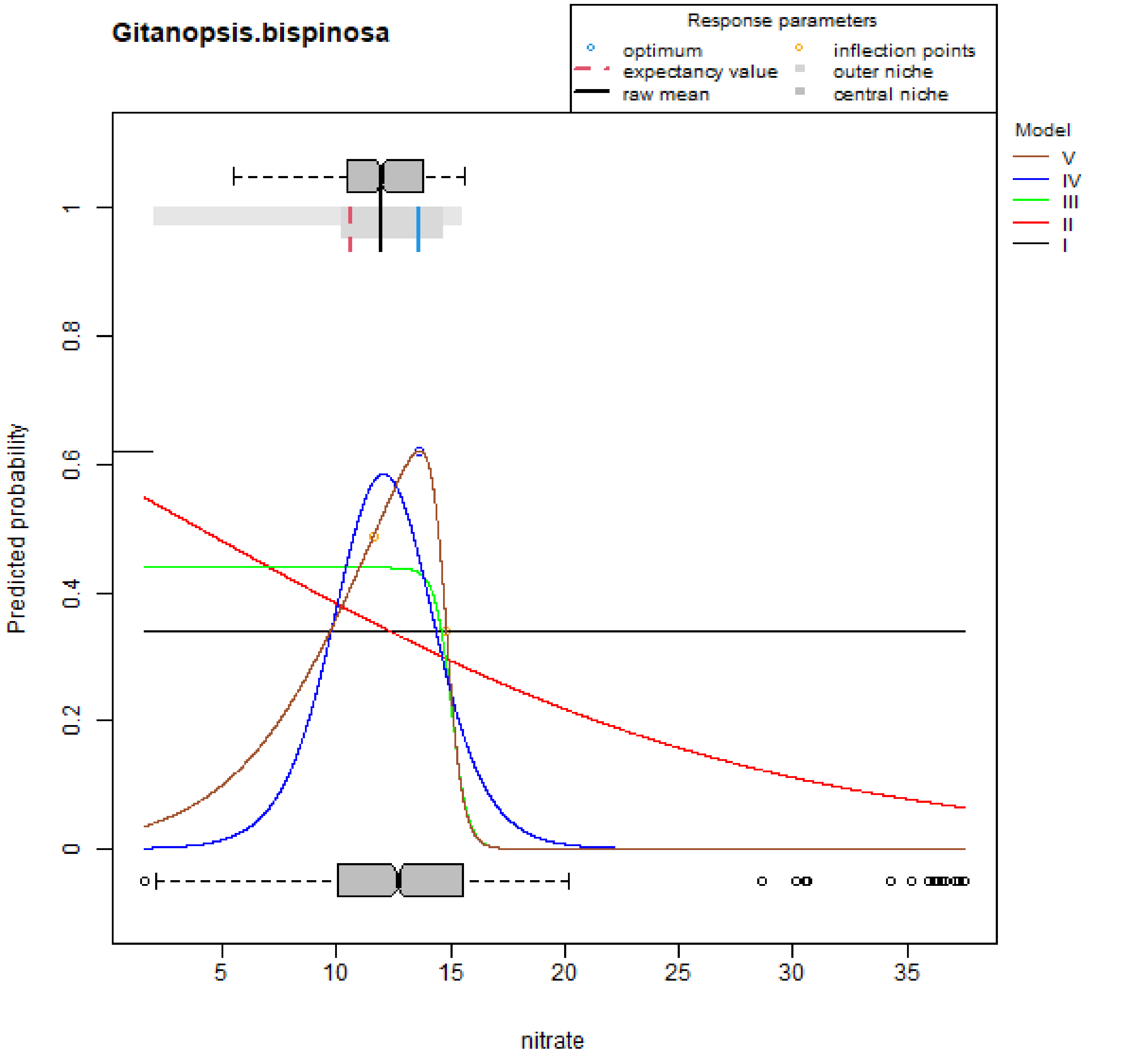

Gitanopsis.bispinosa
Response parameters
optimum
expectancy value
raw mean
inflection points
outer niche
central niche
Model
V
IV
III
II
I
Predicted probability
0
0.2
0.4
0.6
0.8
1
5
10
15
20
25
30
35
nitrate

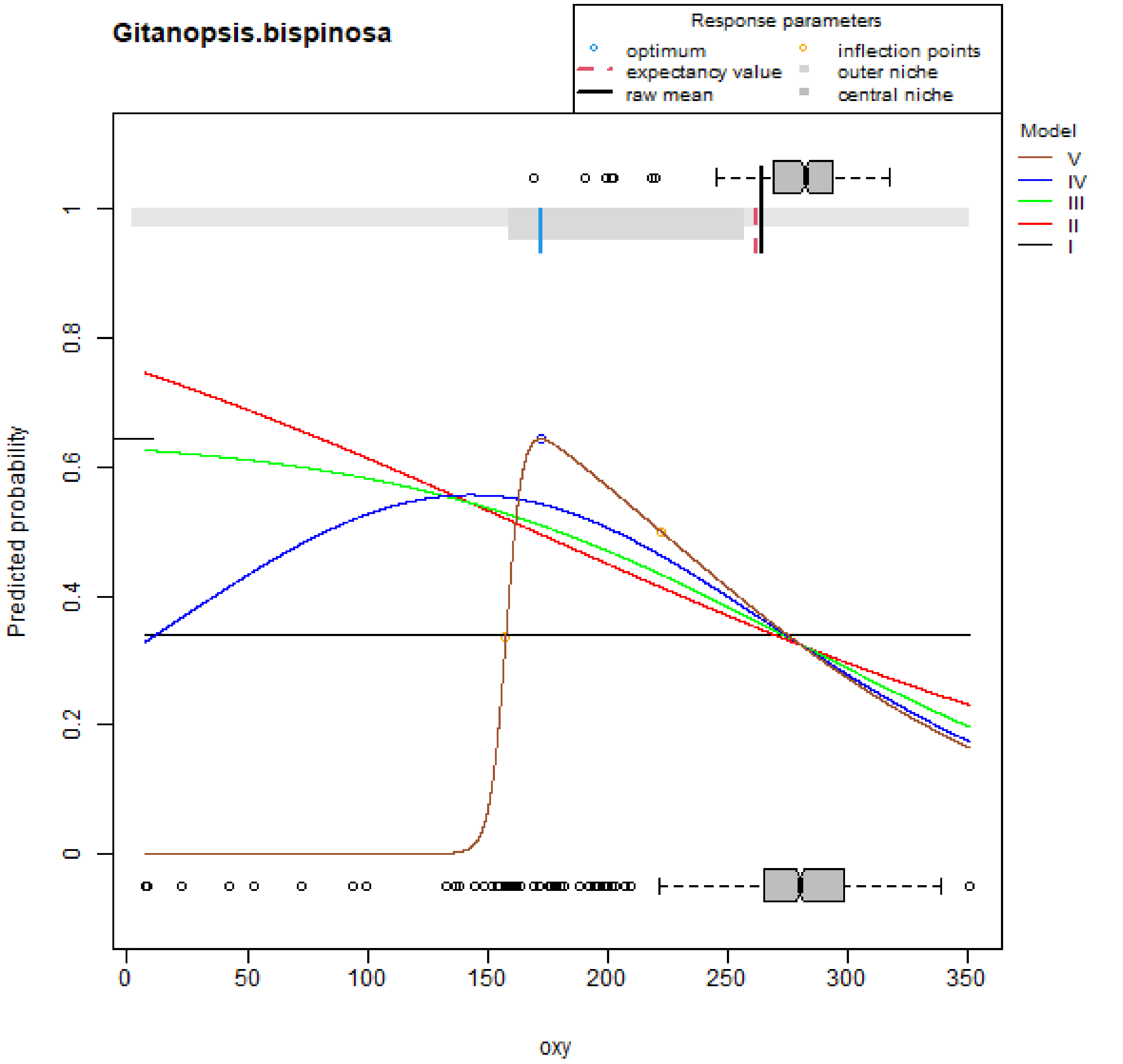

Gitanopsis.bispinosa
Response parameters
optimum
inflection points
expectancy value
outer niche
raw mean
central niche
Model
V
IV
III
II
I
Predicted probability
0
0.2
0.4
0.6
0.8
1
0
50
100
150
200
250
300
350
oxy

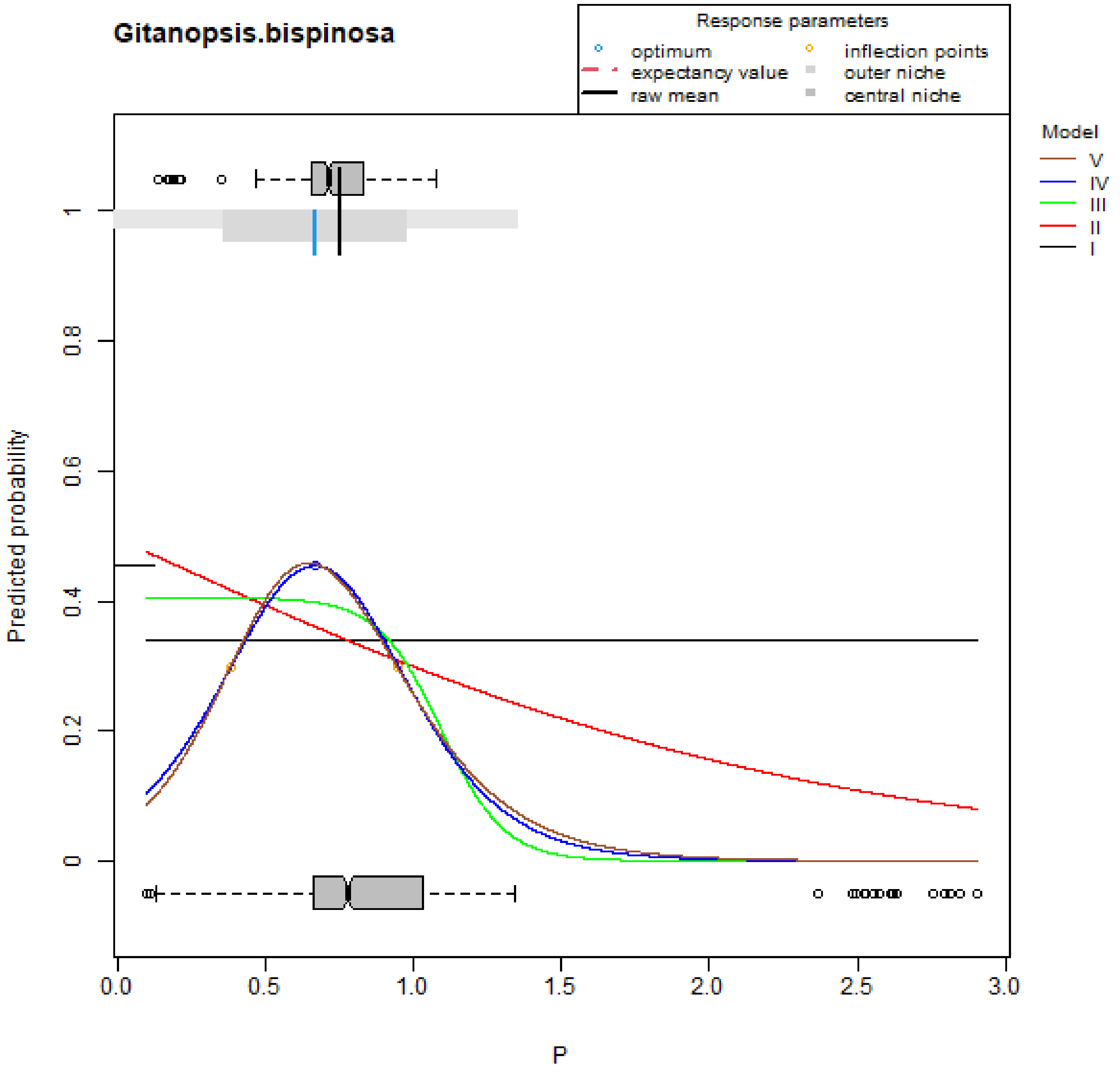

Gitanopsis.bispinosa
Response parameters
optimum
expectancy value
raw mean
inflection points
outer niche
central niche
Model
V
IV
III
II
I
Predicted probability
0
0.2
0.4
0.6
0.8
1
0.0
0.5
1.0
1.5
2.0
2.5
3.0
P

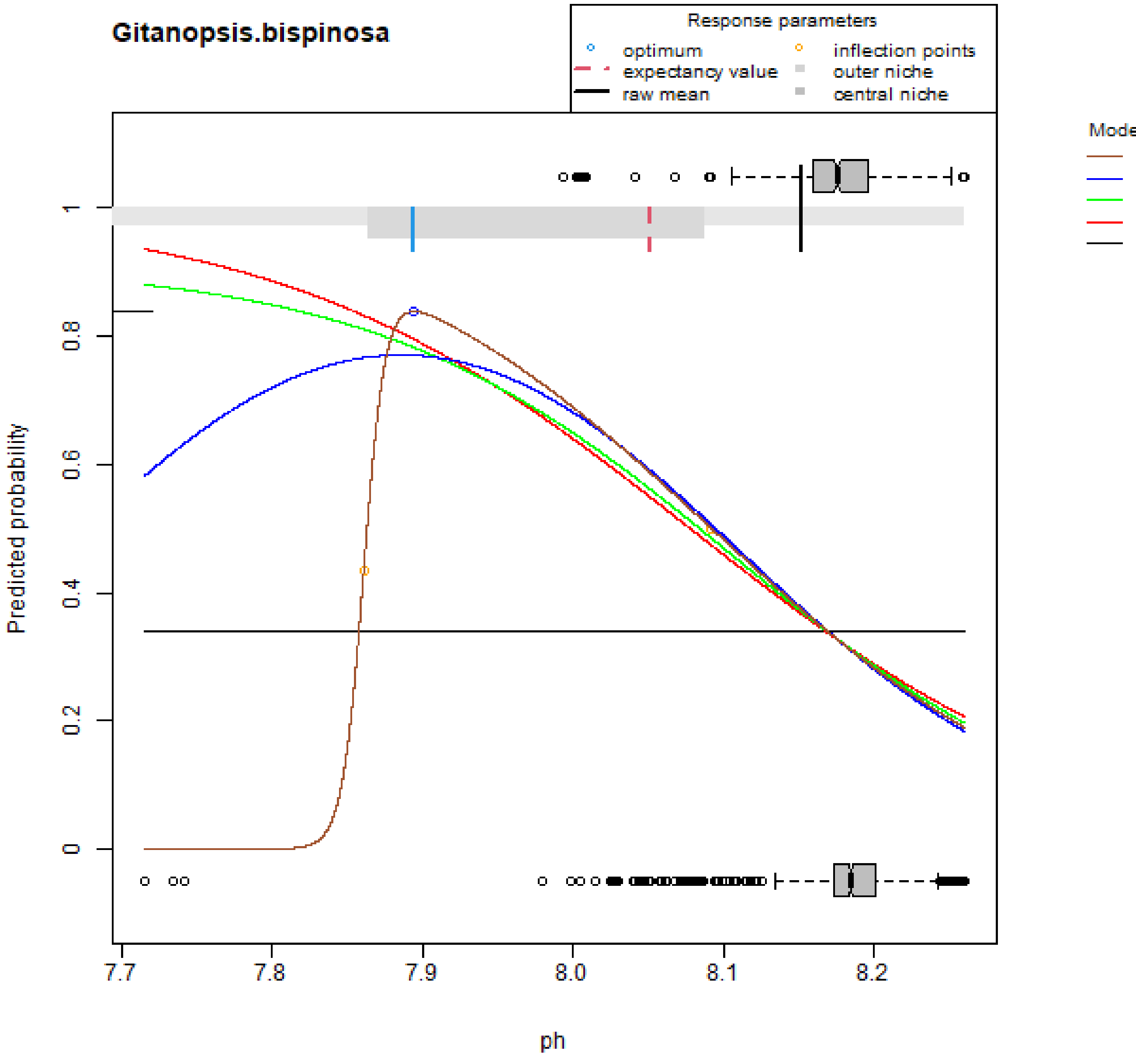

Gitanopsis.bispinosa
Response parameters
optimum
expectancy value
raw mean
inflection points
outer niche
central niche
Mode
Predicted probability
0
0.2
0.4
0.6
0.8
1
7.7
7.8
7.9
8.0
8.1
8.2
ph

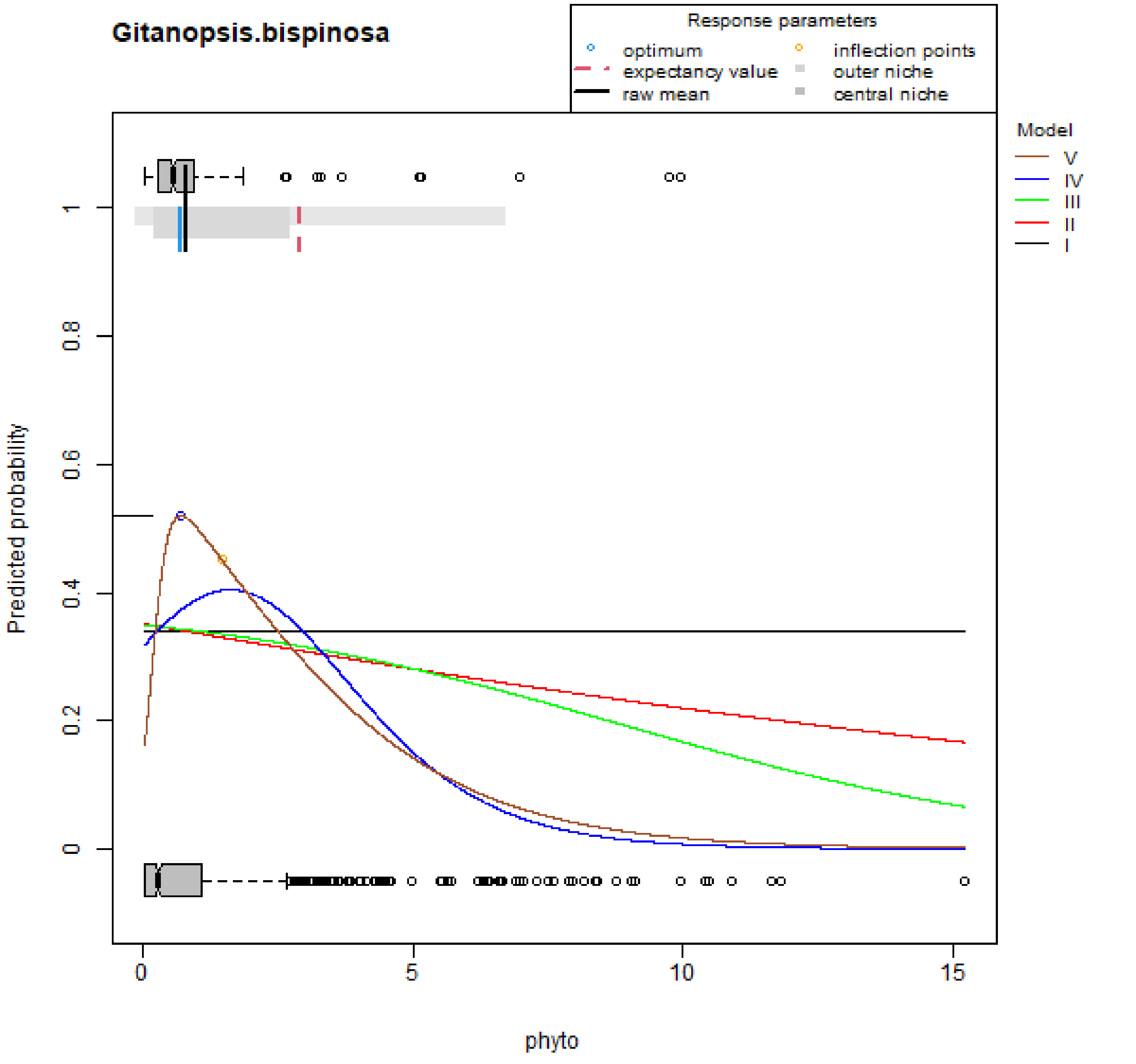
Gitanopsis.bispinosa
Response parameters
optimum
expectancy value
raw mean
inflection points
outer niche
central niche
Model
V
IV
III
II
I
Predicted probability
0
0.2
0.4
0.6
0.8
1
0
5
10
15
phyto

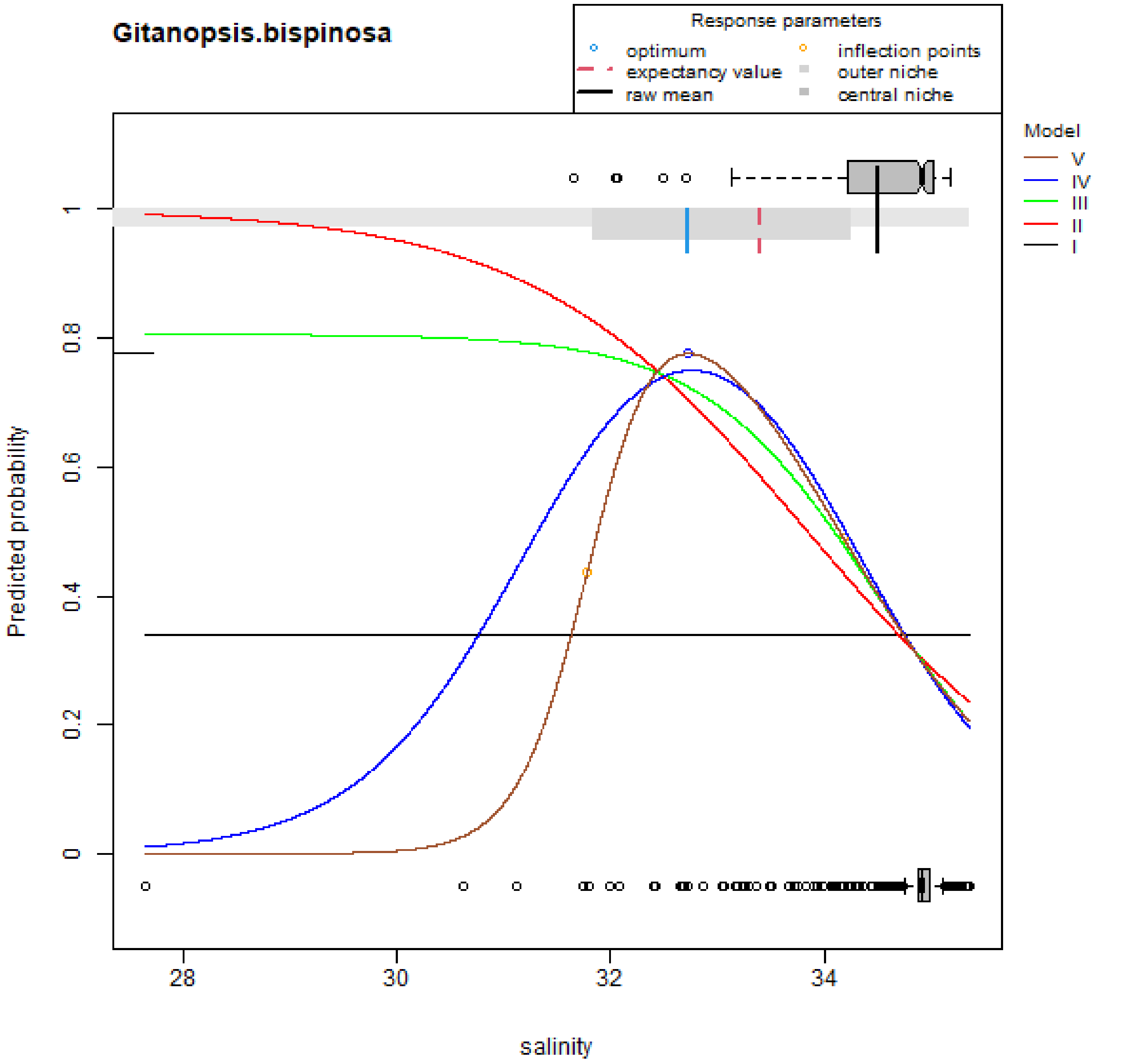
Gitanopsis.bispinosa
Response parameters
optimum
expectancy value
raw mean
inflection points
outer niche
central niche
Model
V
IV
III
II
I
Predicted probability
0
0.2
0.4
0.6
0.8
1
28
30
32
34
salinity

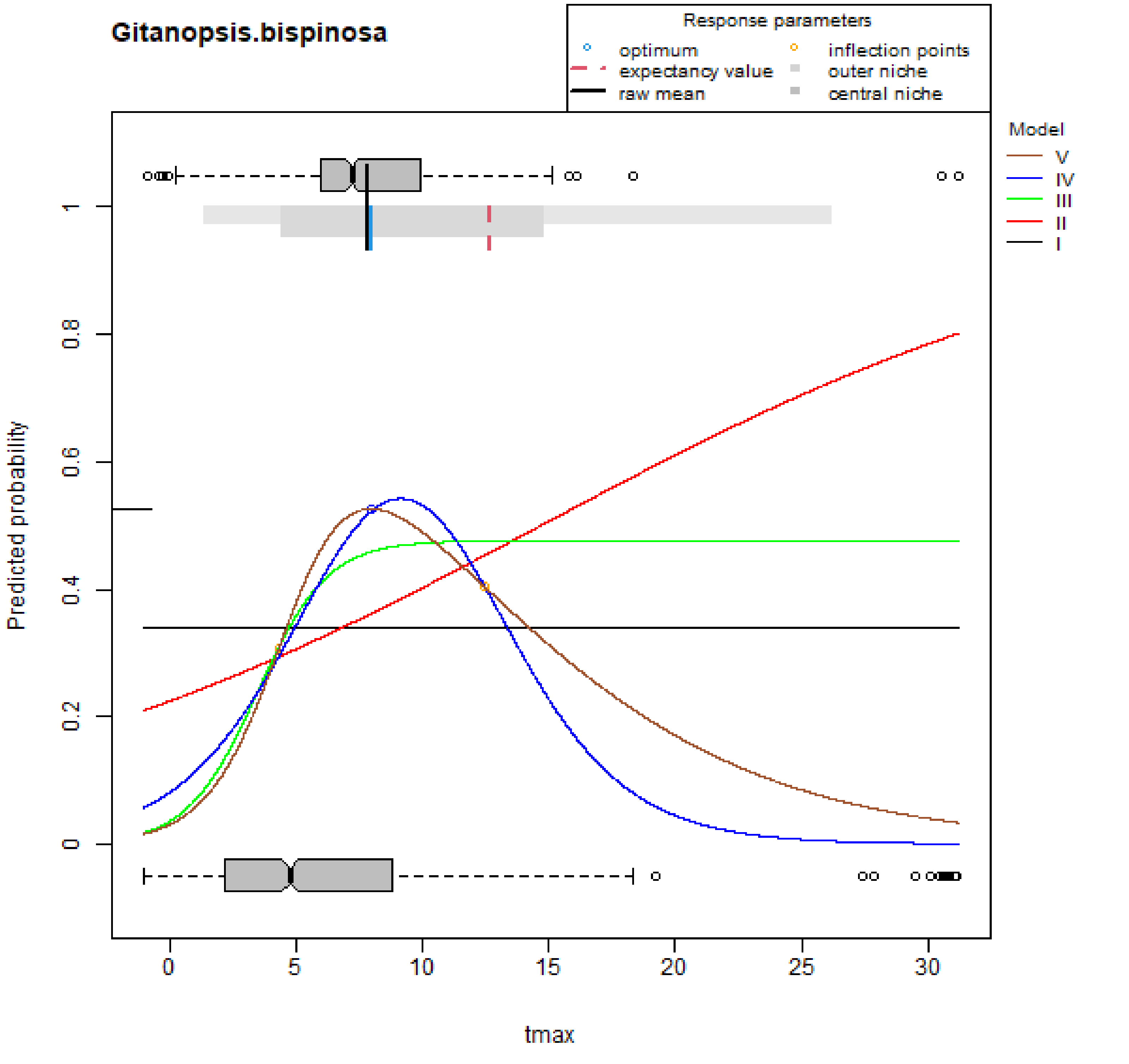

Gitanopsis.bispinosa
Response parameters
optimum
expectancy value
raw mean
inflection points
outer niche
central niche
Model
V
IV
III
II
I
Predicted probability
0
0.2
0.4
0.6
0.8
1
0
5
10
15
20
25
30
tmax

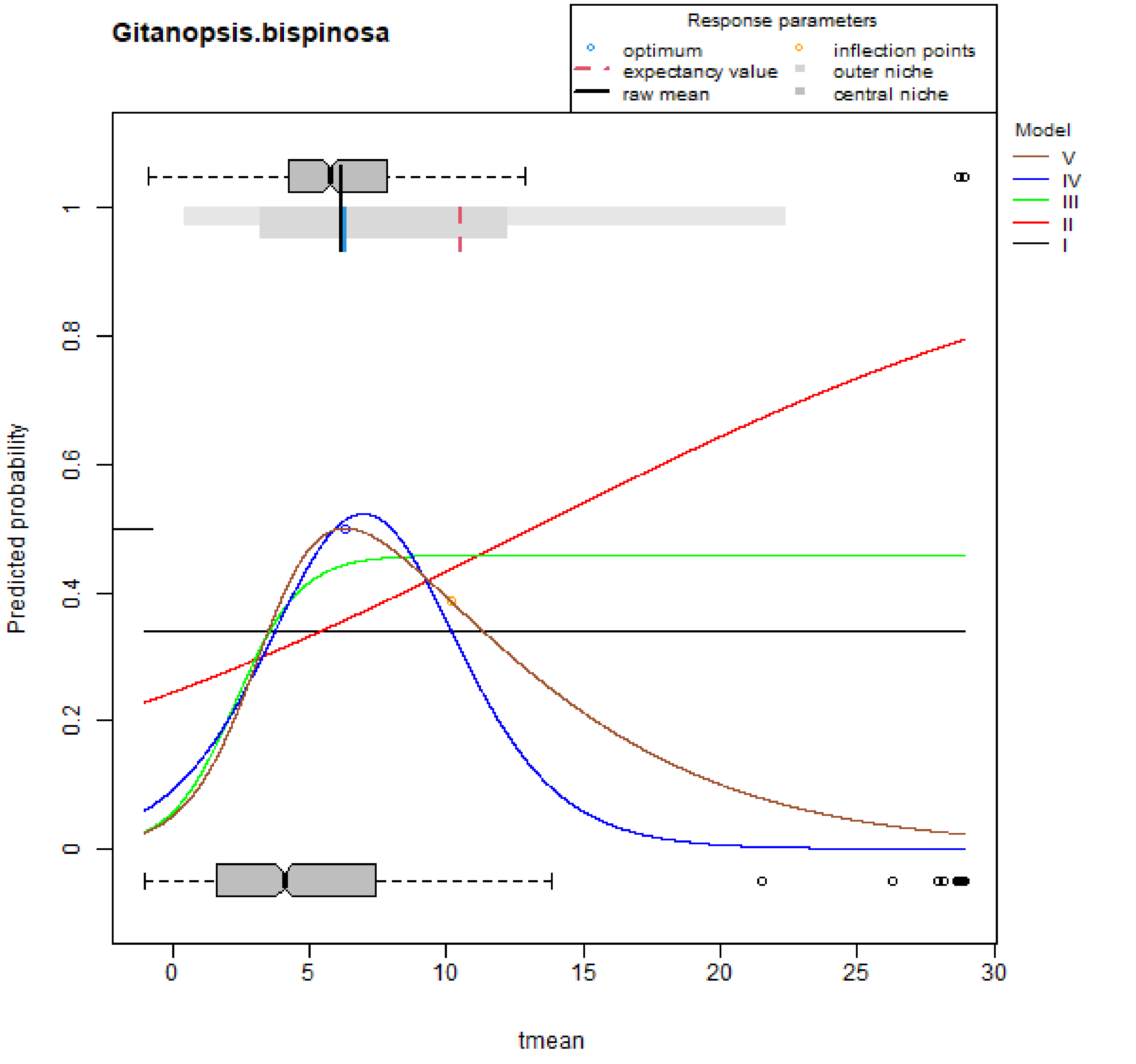

Gitanopsis.bispinosa
Response parameters
optimum
expectancy value
raw mean
inflection points
outer niche
central niche
Model
V
IV
III
II
I
Predicted probability
0
0.2
0.4
0.6
0.8
1
0
5
10
15
20
25
30
tmean

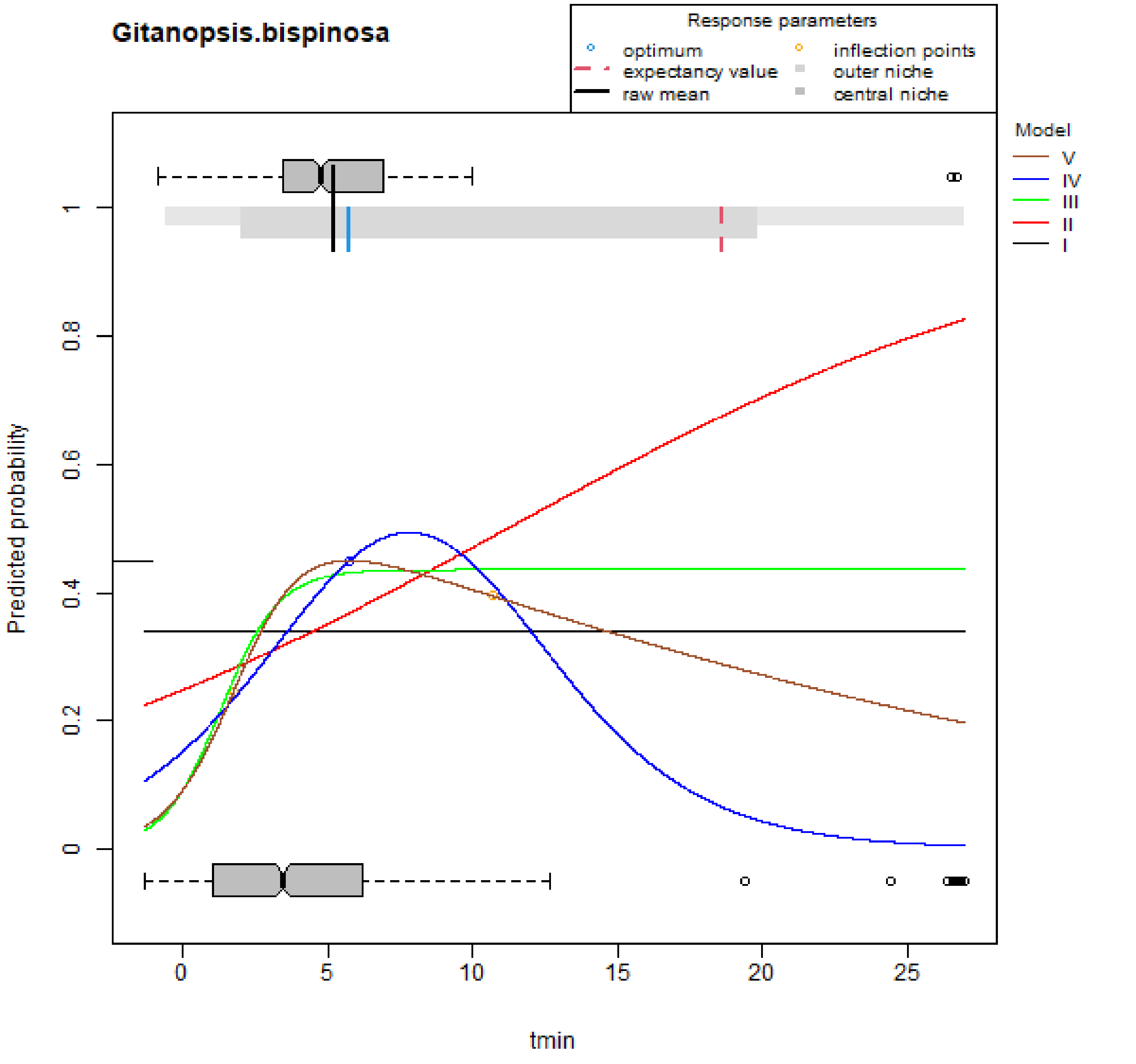
Gitanopsis.bispinosa
Response parameters
optimum
expectancy value
raw mean
inflection points
outer niche
central niche
Model
V
IV
III
II
I
Predicted probability
0
0.2
0.4
0.6
0.8
1
0
5
10
15
20
25
tmin

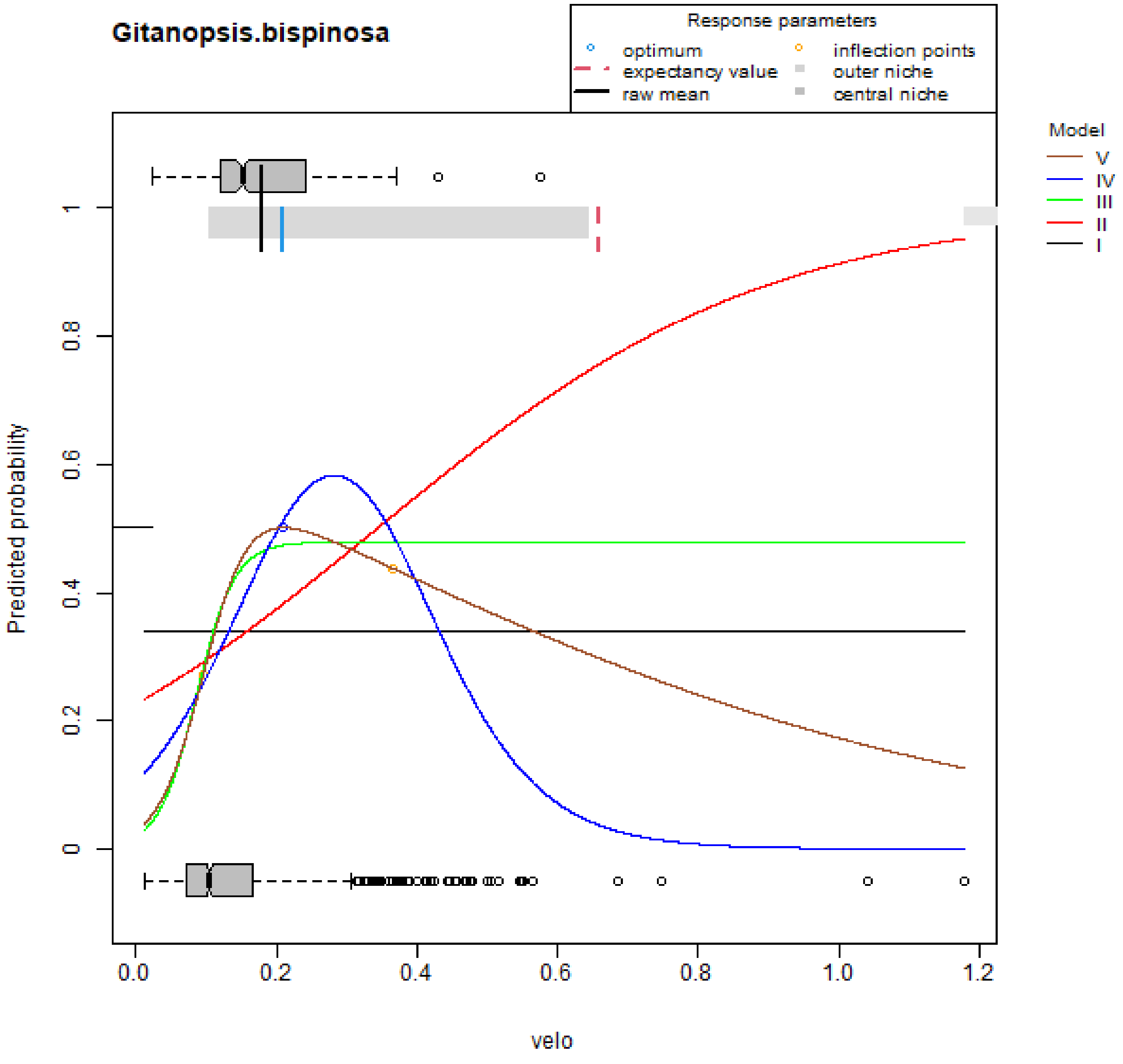

Gitanopsis.bispinosa
Response parameters
optimum
inflection points
expectancy value
outer niche
raw mean
central niche
Model
V
IV
III
II
I
Predicted probability
0
0.2
0.4
0.6
0.8
1
0.0
0.2
0.4
0.6
0.8
1.0
1.2
velo

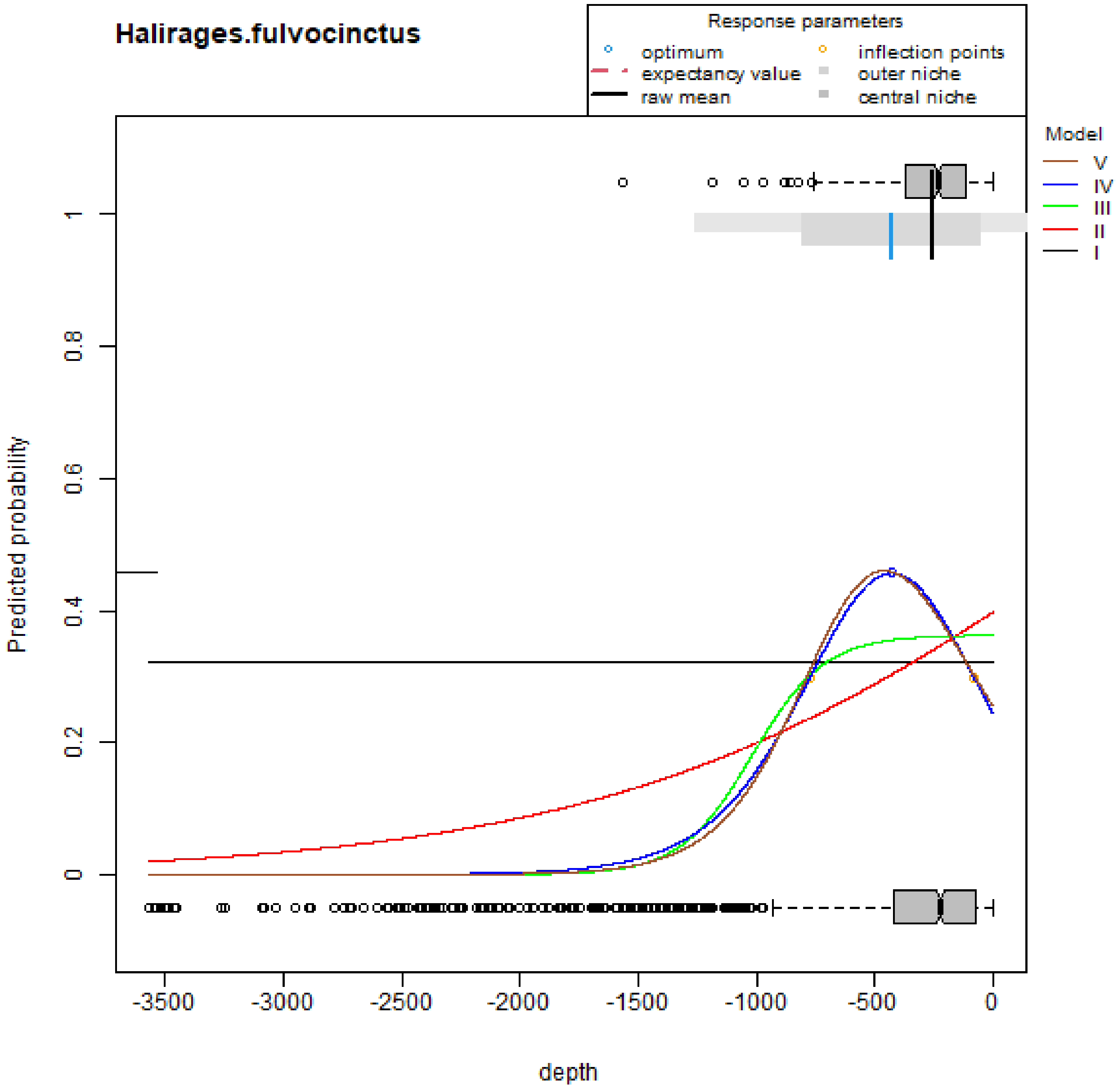

Halirages.fulvocinctus
Response parameters
optimum
expectancy value
raw mean
inflection points
outer niche
central niche
Model
V
IV
III
II
I
Predicted probability
0
0.2
0.4
0.6
0.8
1
-3500
-3000
-2500
-2000
-1500
-1000
-500
0
depth

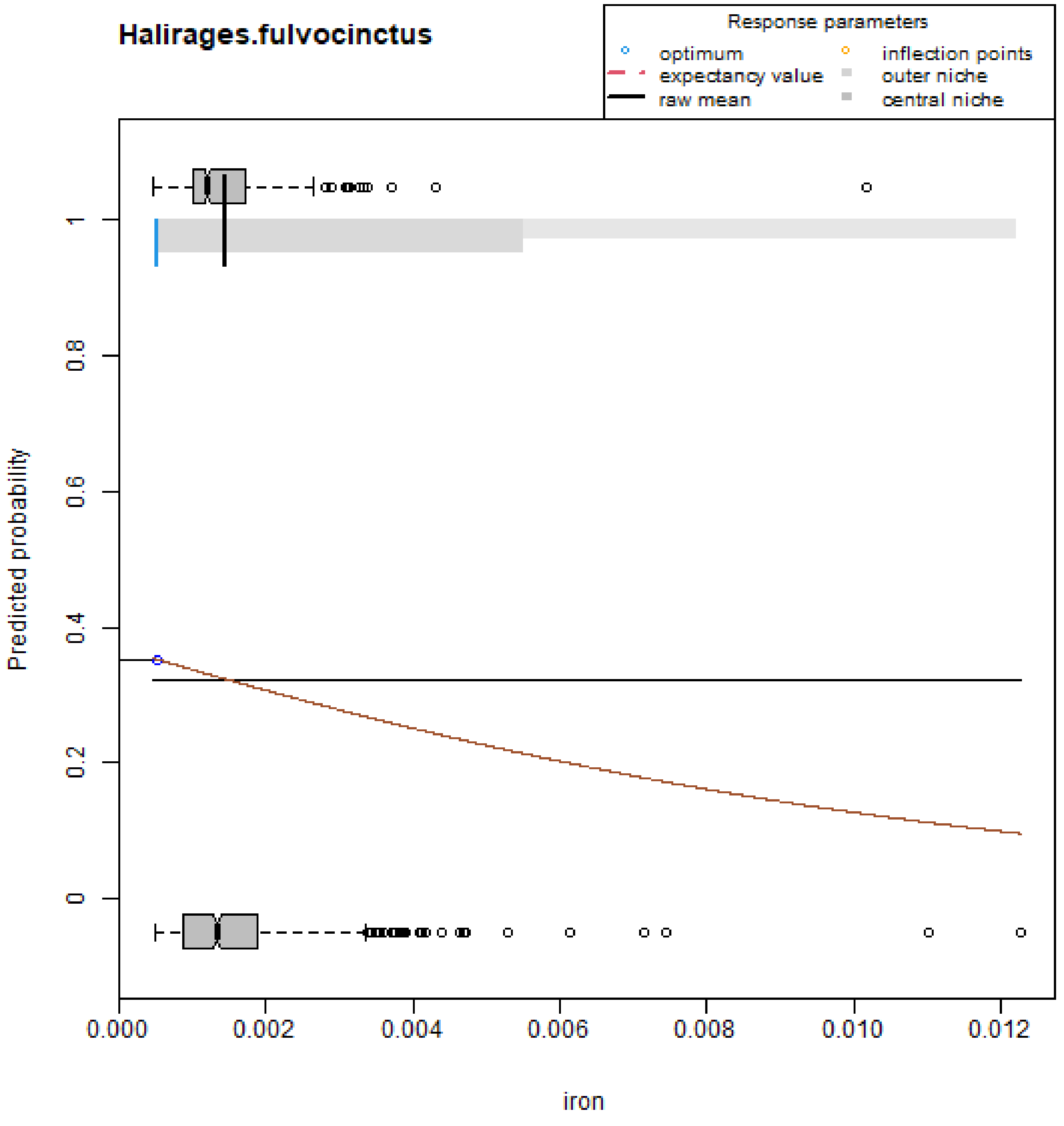

Halirages.fulvocinctus
Response parameters
optimum
expectancy value
raw mean
inflection points
outer niche
central niche
Predicted probability
0
0.2
0.4
0.6
0.8
1
0.000
0.002
0.004
0.006
0.008
0.010
0.012
iron

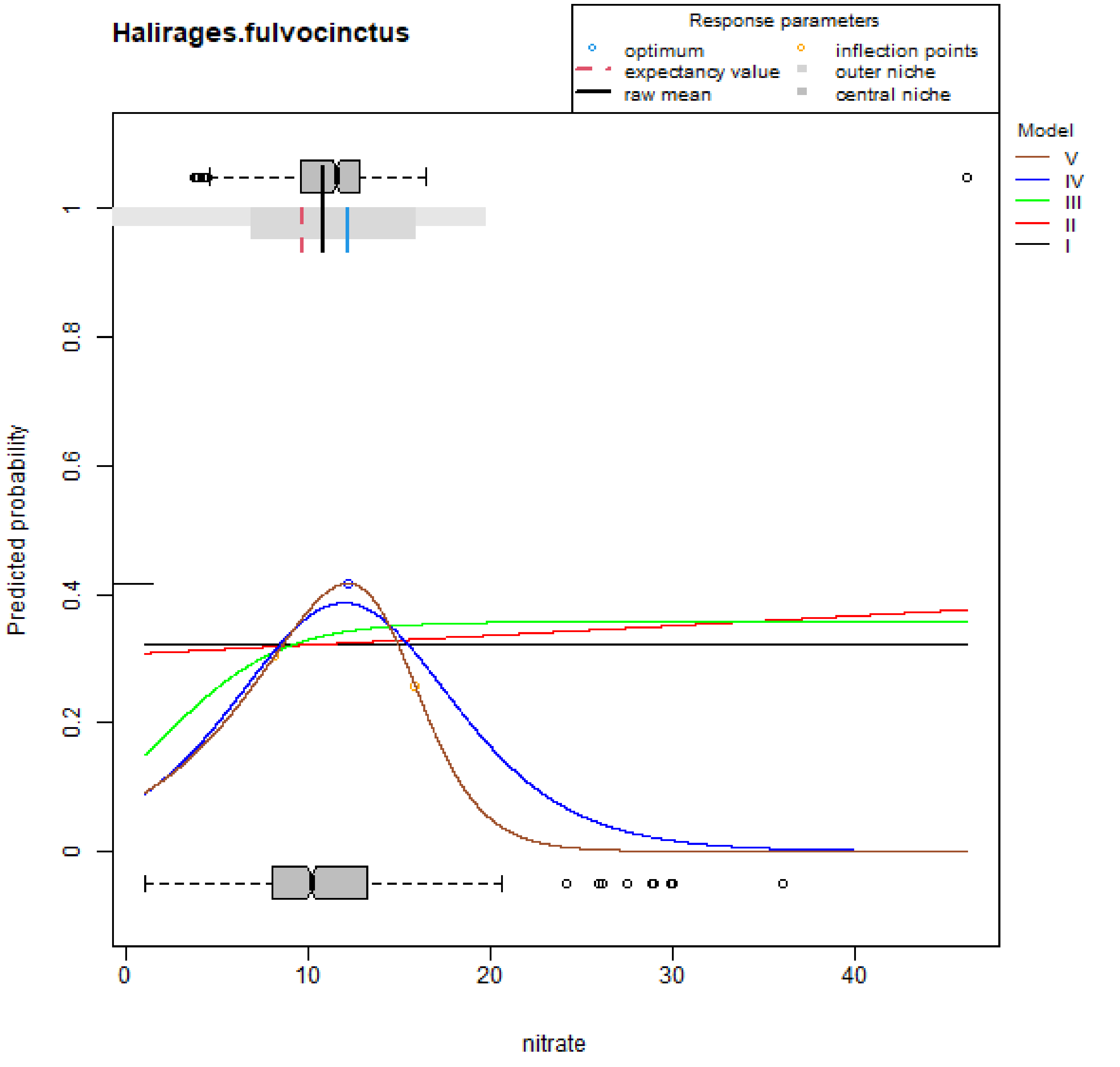

Halirages.fulvocinctus
Response parameters
optimum
expectancy value
raw mean
inflection points
outer niche
central niche
Model
V
IV
III
II
I
Predicted probability
0
0.2
0.4
0.6
0.8
1
0
10
20
30
40
nitrate

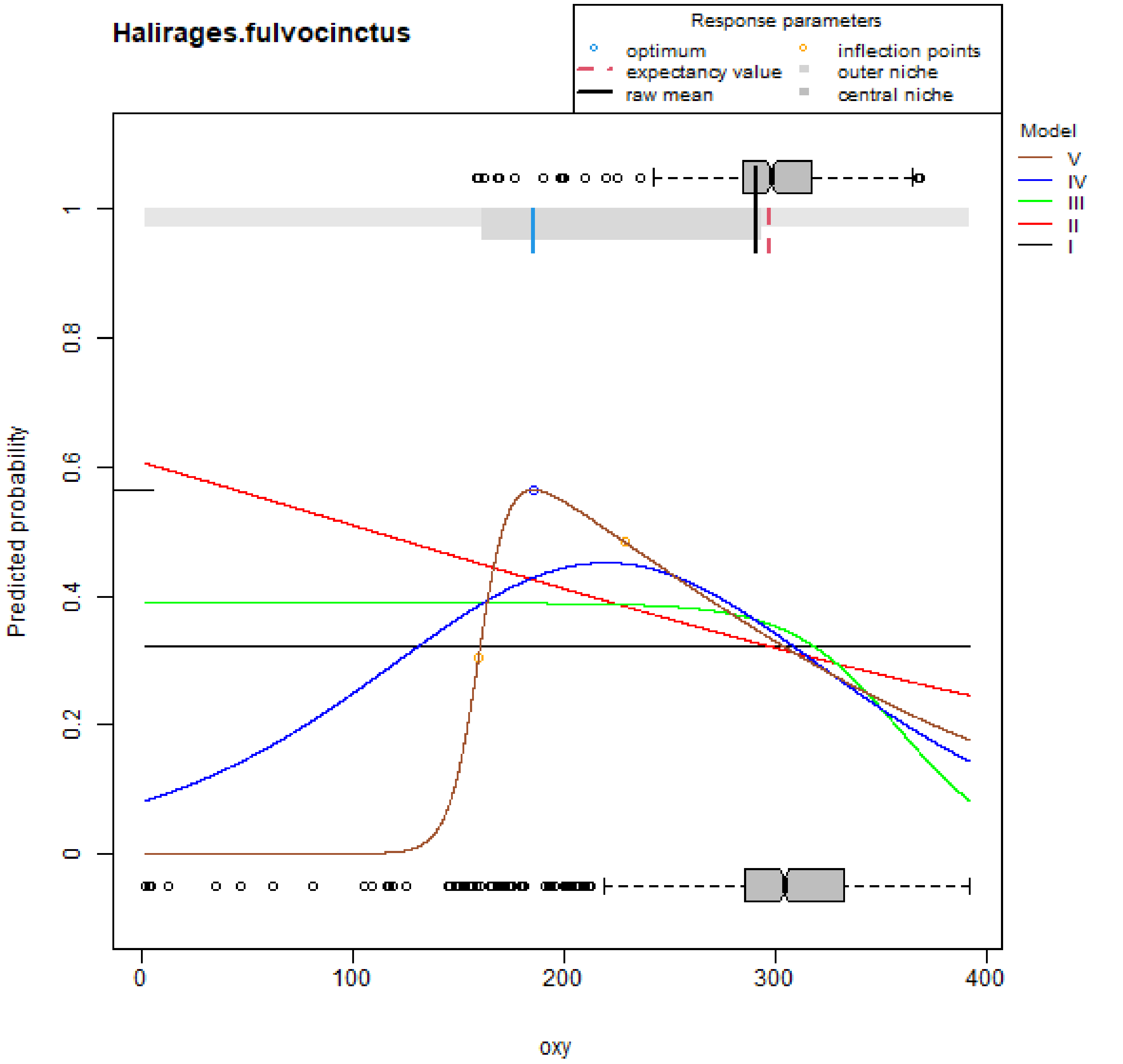

Halirages.fulvocinctus
Response parameters
optimum
expectancy value
raw mean
inflection points
outer niche
central niche
Model
V
IV
III
II
I
Predicted probability
0
0.2
0.4
0.6
0.8
1
0
100
200
300
400
oxy

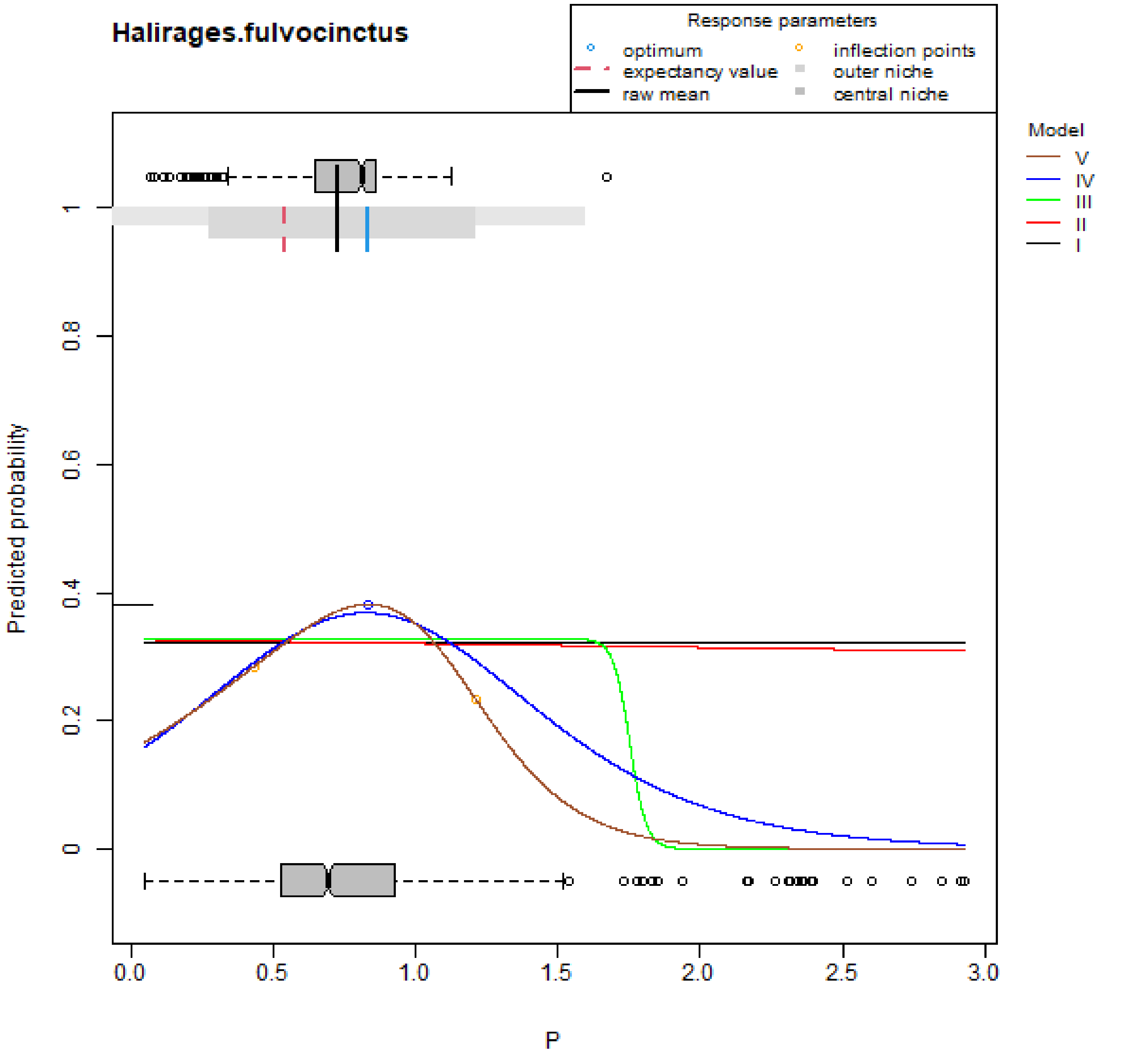

Halirages.fulvocinctus
Response parameters
optimum
expectancy value
raw mean
inflection points
outer niche
central niche
Model
V
IV
III
II
I
Predicted probability
0
0.2
0.4
0.6
0.8
1
0.0
0.5
1.0
1.5
2.0
2.5
3.0
P

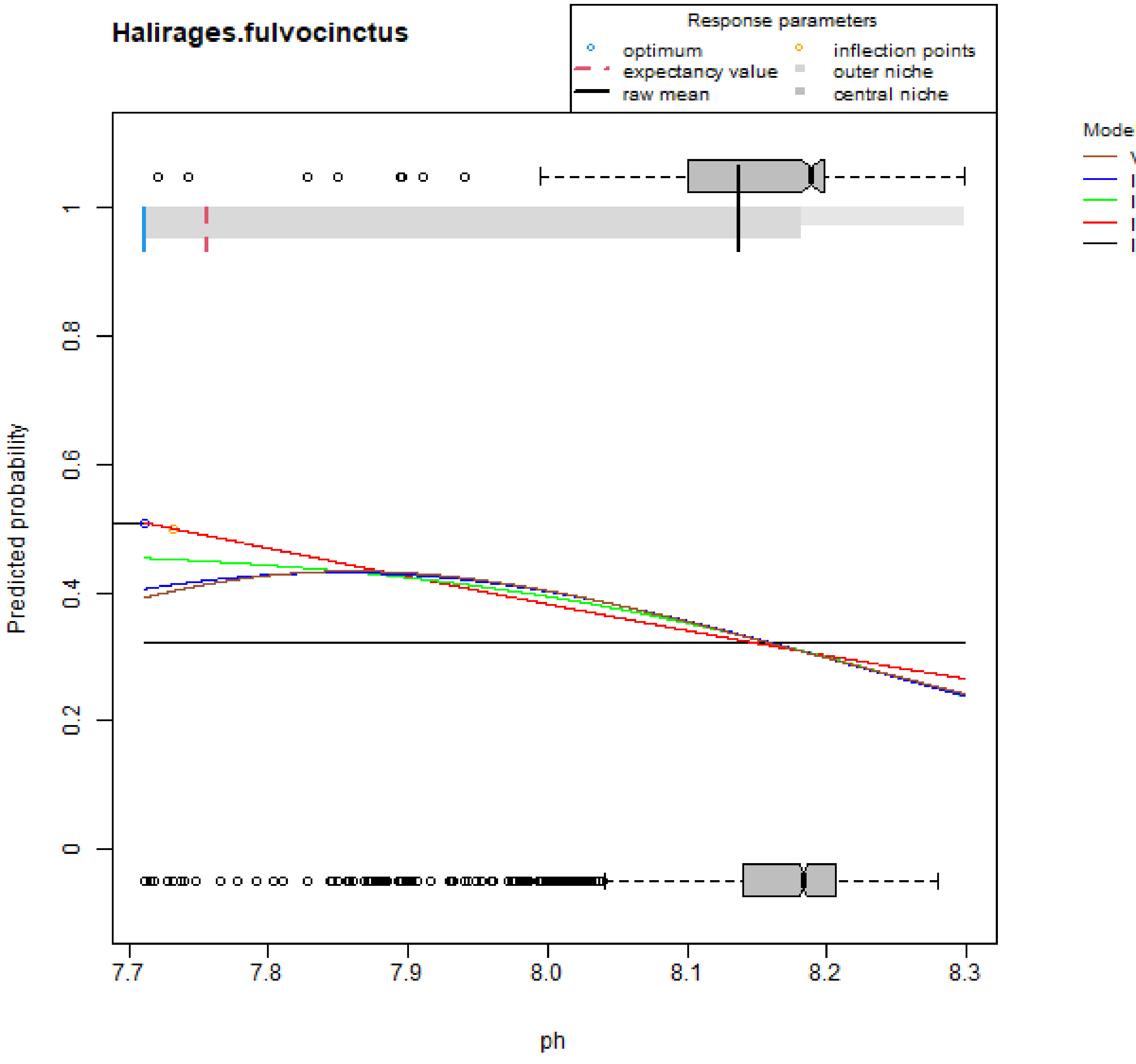

Halirages.fulvocinctus
Response parameters
optimum
expectancy value
raw mean
inflection points
outer niche
central niche
Predicted probability
0
0.2
0.4
0.6
0.8
1
7.7
7.8
7.9
8.0
8.1
8.2
8.3
ph

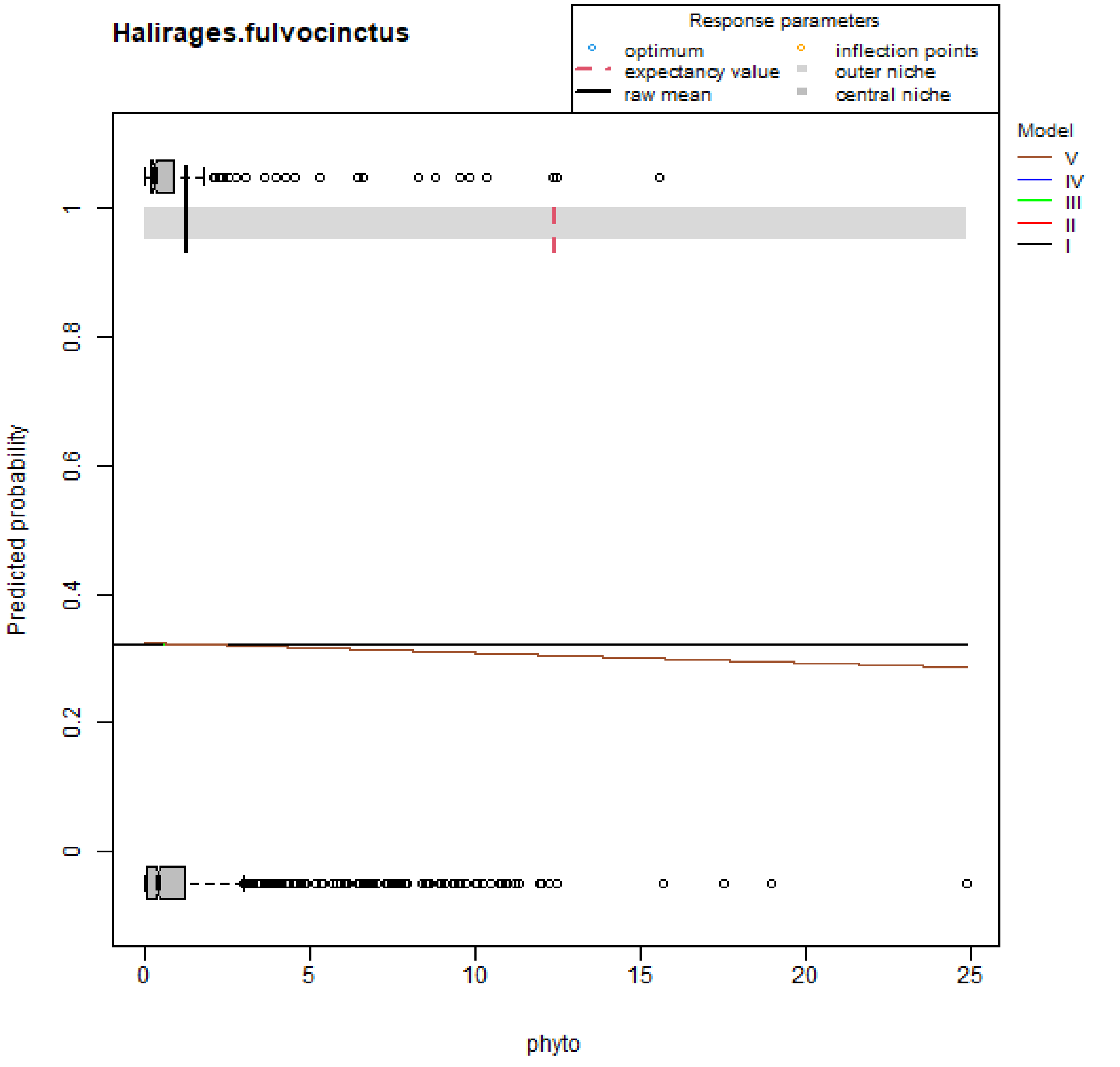

Halirages.fulvocinctus
Response parameters
optimum
expectancy value
raw mean
inflection points
outer niche
central niche
Model
V
IV
III
II
I
Predicted probability
0
0.2
0.4
0.6
0.8
1
0
5
10
15
20
25
phyto

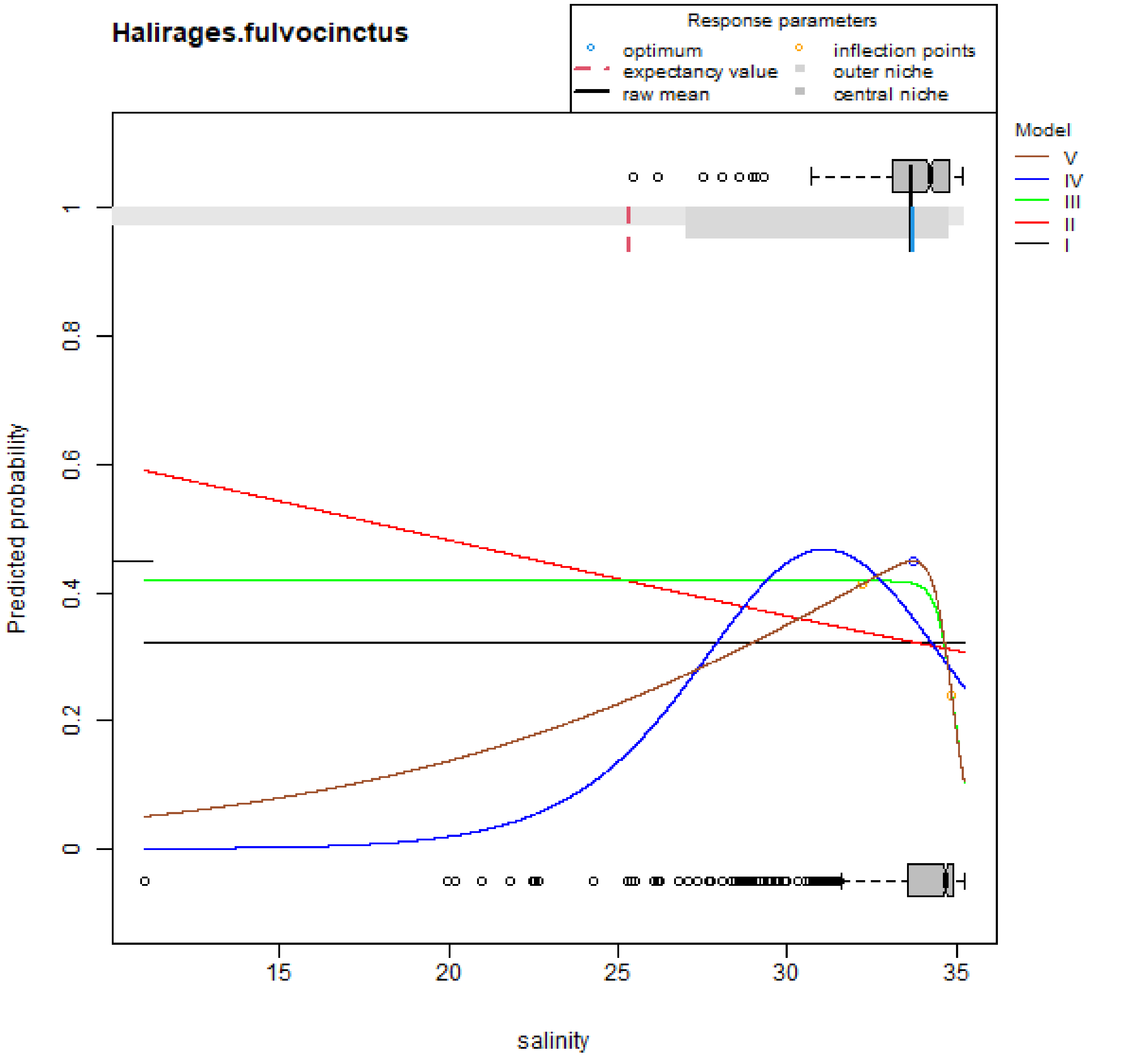

Halirages.fulvocinctus
Response parameters
optimum
expectancy value
raw mean
inflection points
outer niche
central niche
Model
V
IV
III
II
I
Predicted probability
0
0.2
0.4
0.6
0.8
1
15
20
25
30
35
salinity

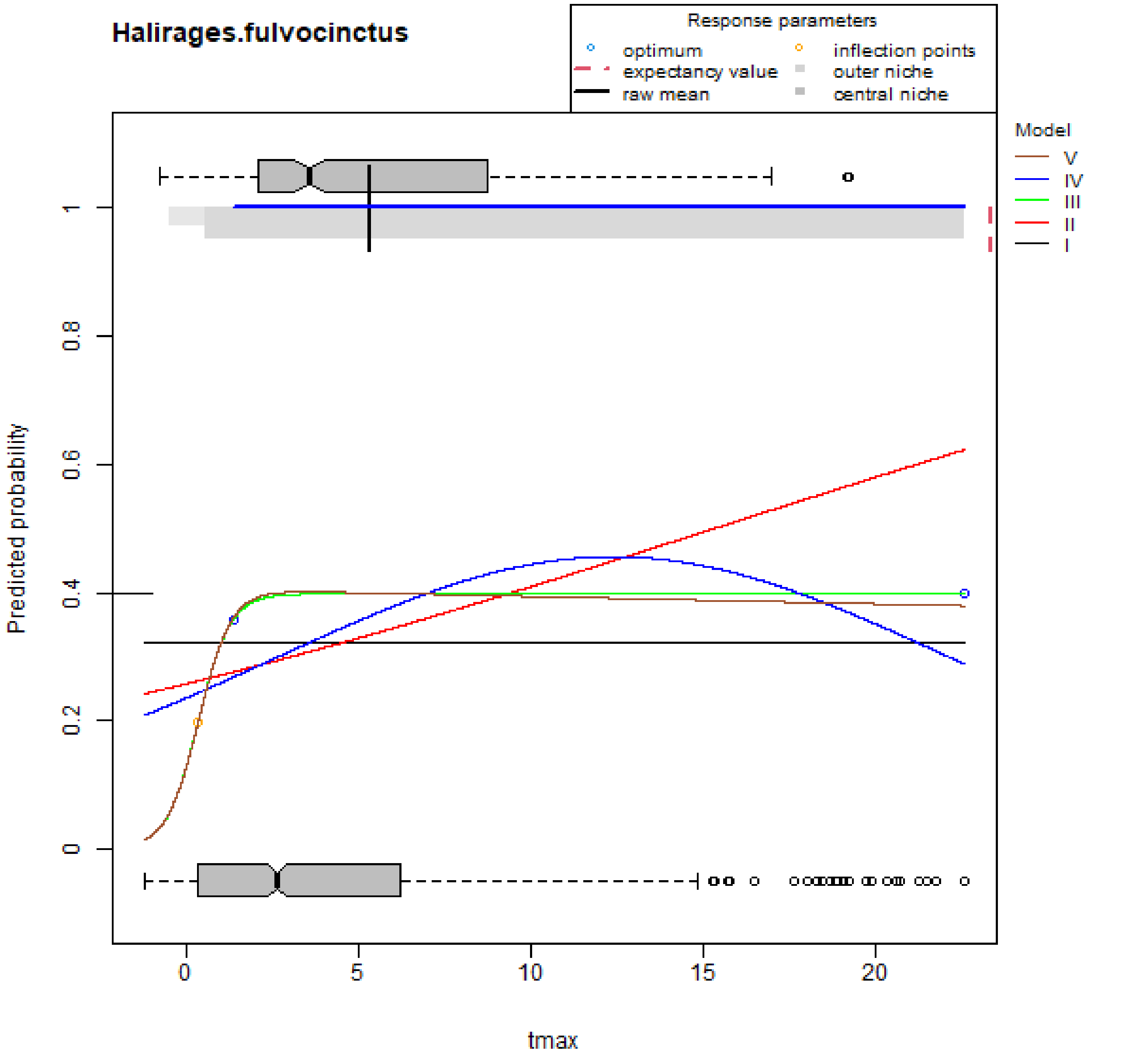
Halirages.fulvocinctus
Response parameters
optimum
inflection points
expectancy value
outer niche
raw mean
central niche
Model
V
IV
III
II
I
Predicted probability
0
0.2
0.4
0.6
0.8
1
0
5
10
15
20
tmax

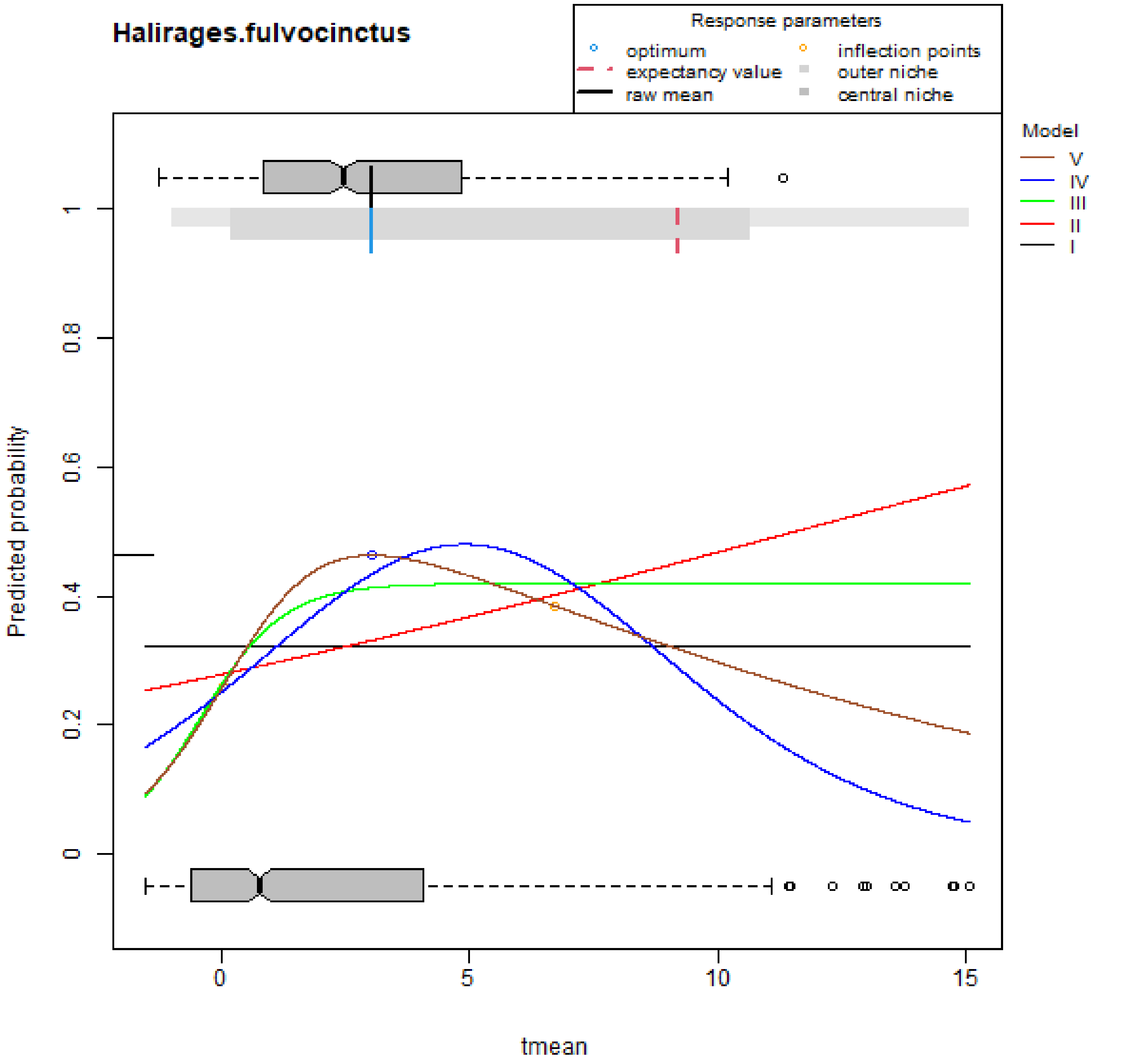

Halirages.fulvocinctus
Response parameters
optimum
inflection points
expectancy value
outer niche
raw mean
central niche
Model
V
IV
III
II
I
Predicted probability
0
0.2
0.4
0.6
0.8
1
tmean
0
5
10
15

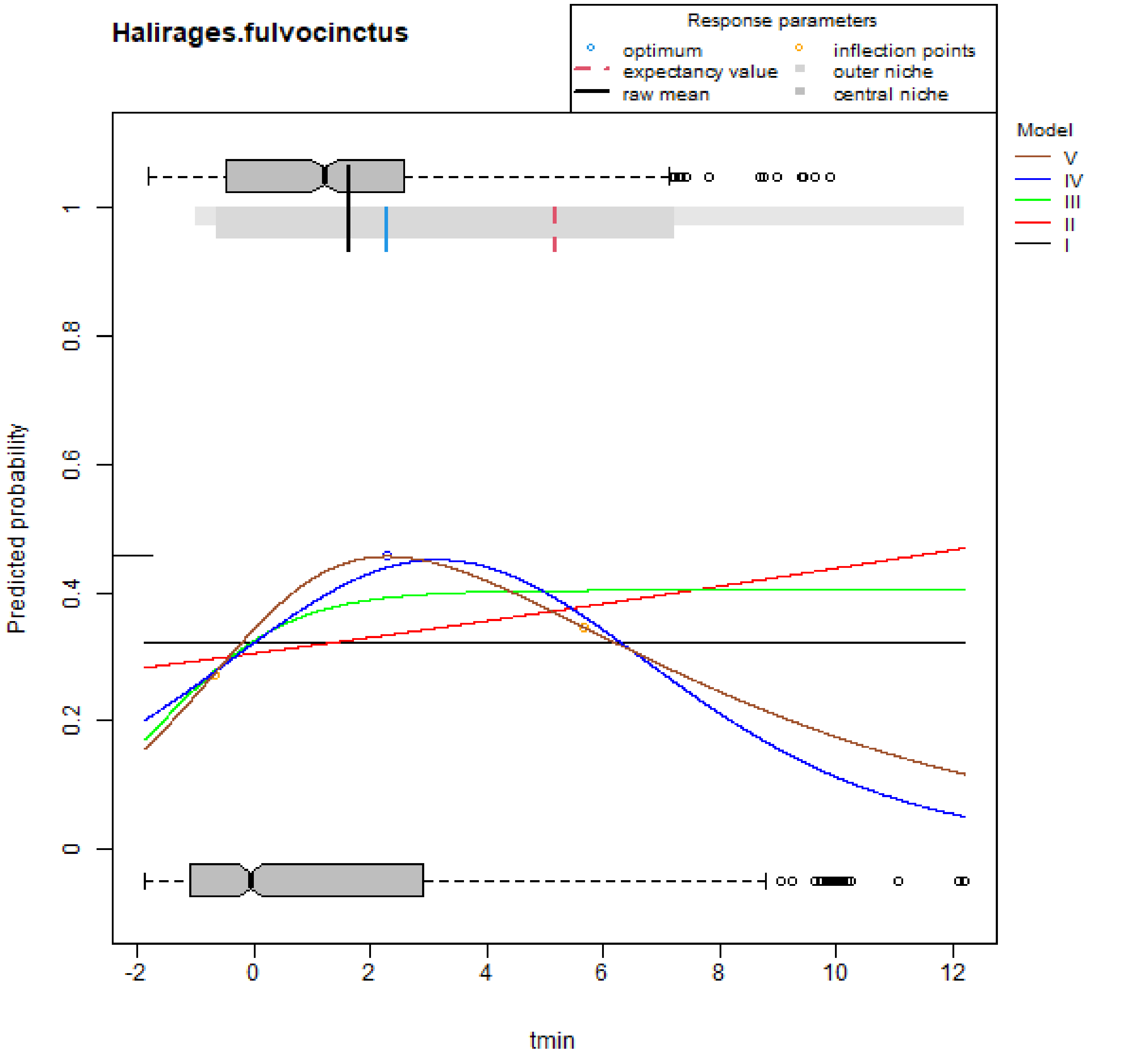

Halirages.fulvocinctus
Response parameters
optimum
expectancy value
raw mean
inflection points
outer niche
central niche
Model
V
IV
III
II
I
Predicted probability
0
0.2
0.4
0.6
0.8
1
-2
0
2
4
6
8
10
12
tmin

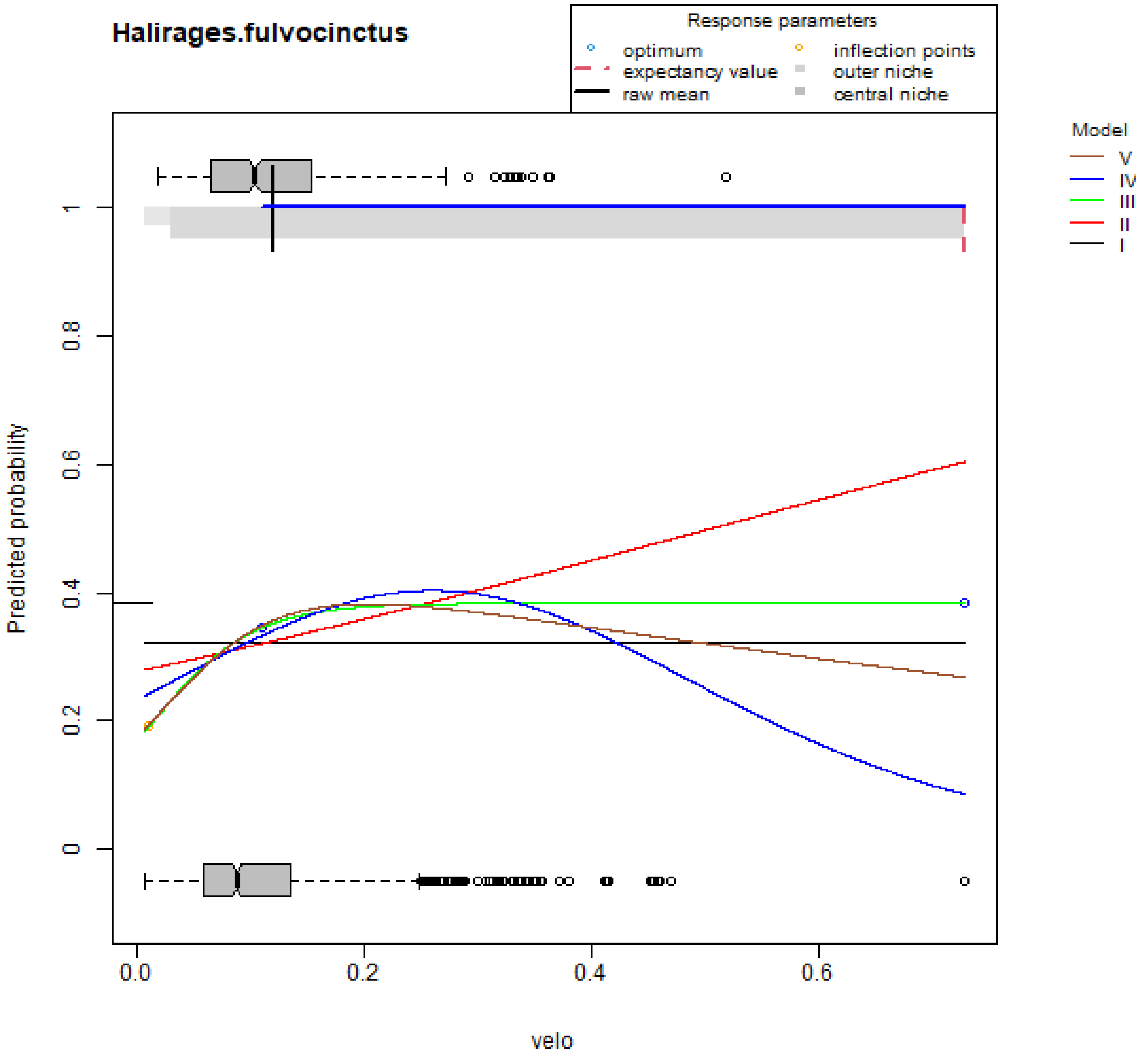
Halirages.fulvocinctus
Response parameters
optimum
inflection points
expectancy value
outer niche
raw mean
central niche
Model
V
IV
III
II
I
Predicted probability
0
0.2
0.4
0.6
0.8
1
0.0
0.2
0.4
0.6
velo

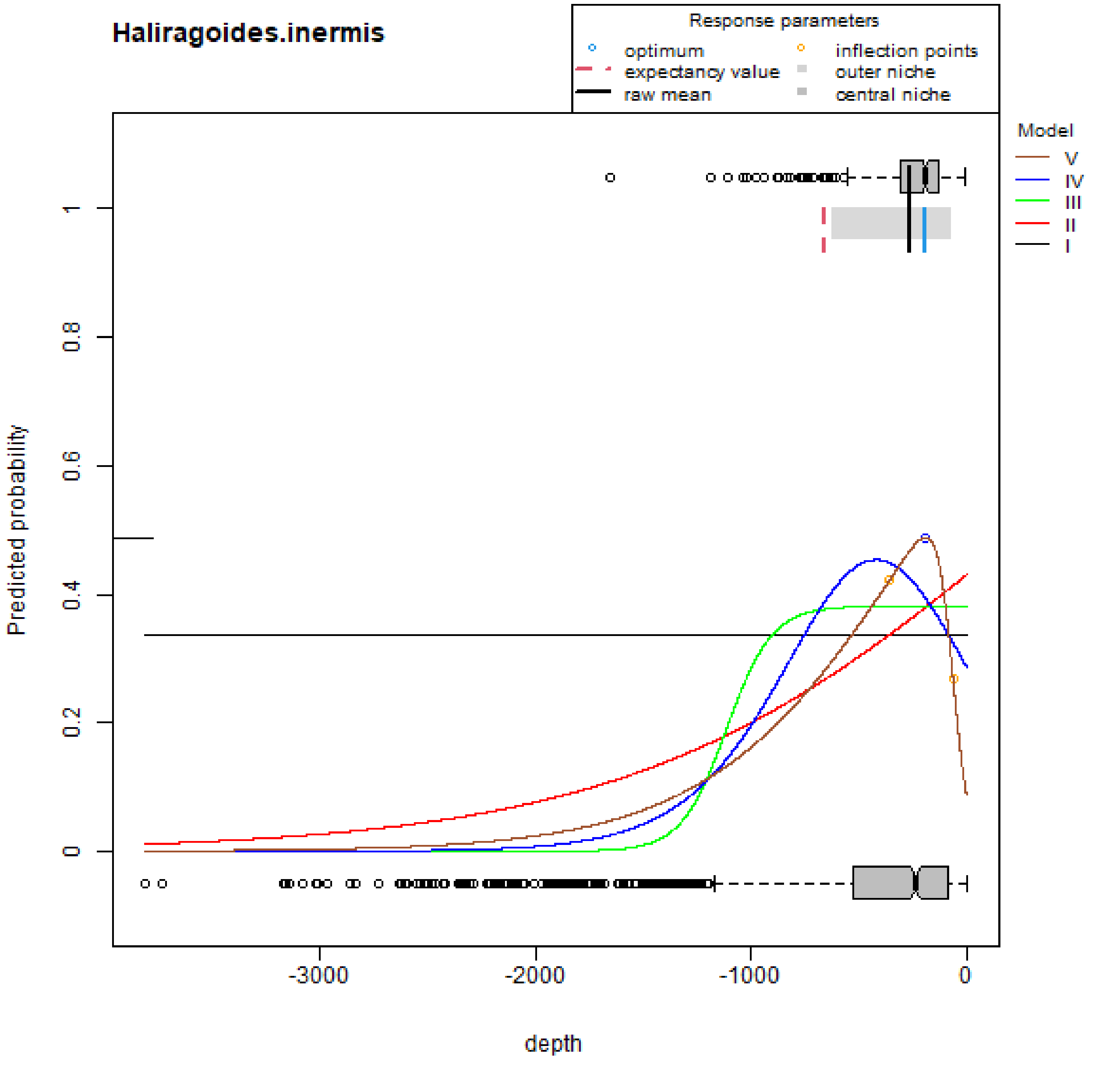

Haliragoides.inermis
Response parameters
optimum
expectancy value
raw mean
inflection points
outer niche
central niche
Model
V
IV
III
II
I
Predicted probability
0
0.2
0.4
0.6
0.8
1
-3000
-2000
-1000
0
depth

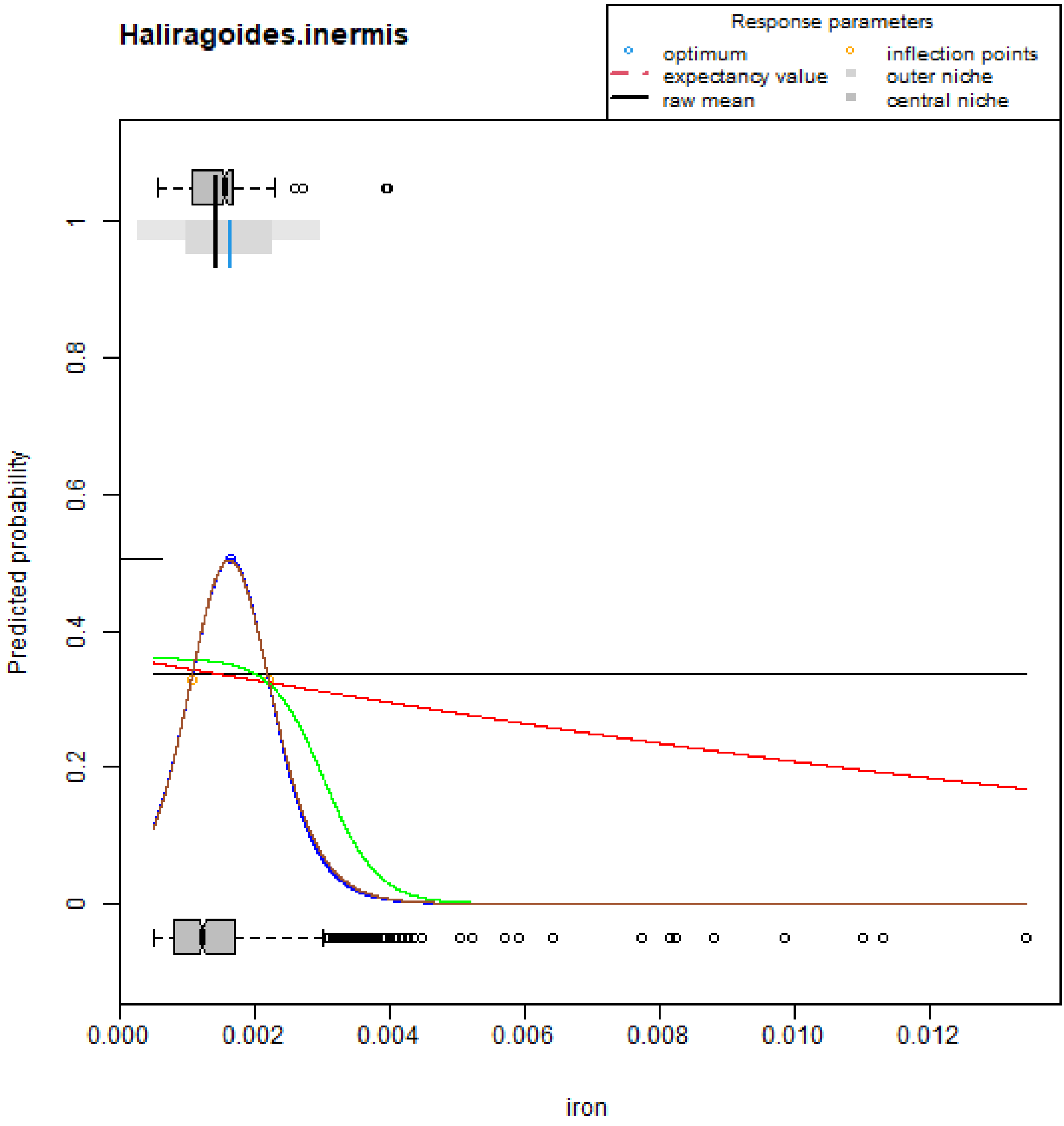

Haliragoides.inermis
Response parameters
optimum
expectancy value
raw mean
inflection points
outer niche
central niche
Predicted probability
0
0.2
0.4
0.6
0.8
1
0.000
0.002
0.004
0.006
0.008
0.010
0.012
iron

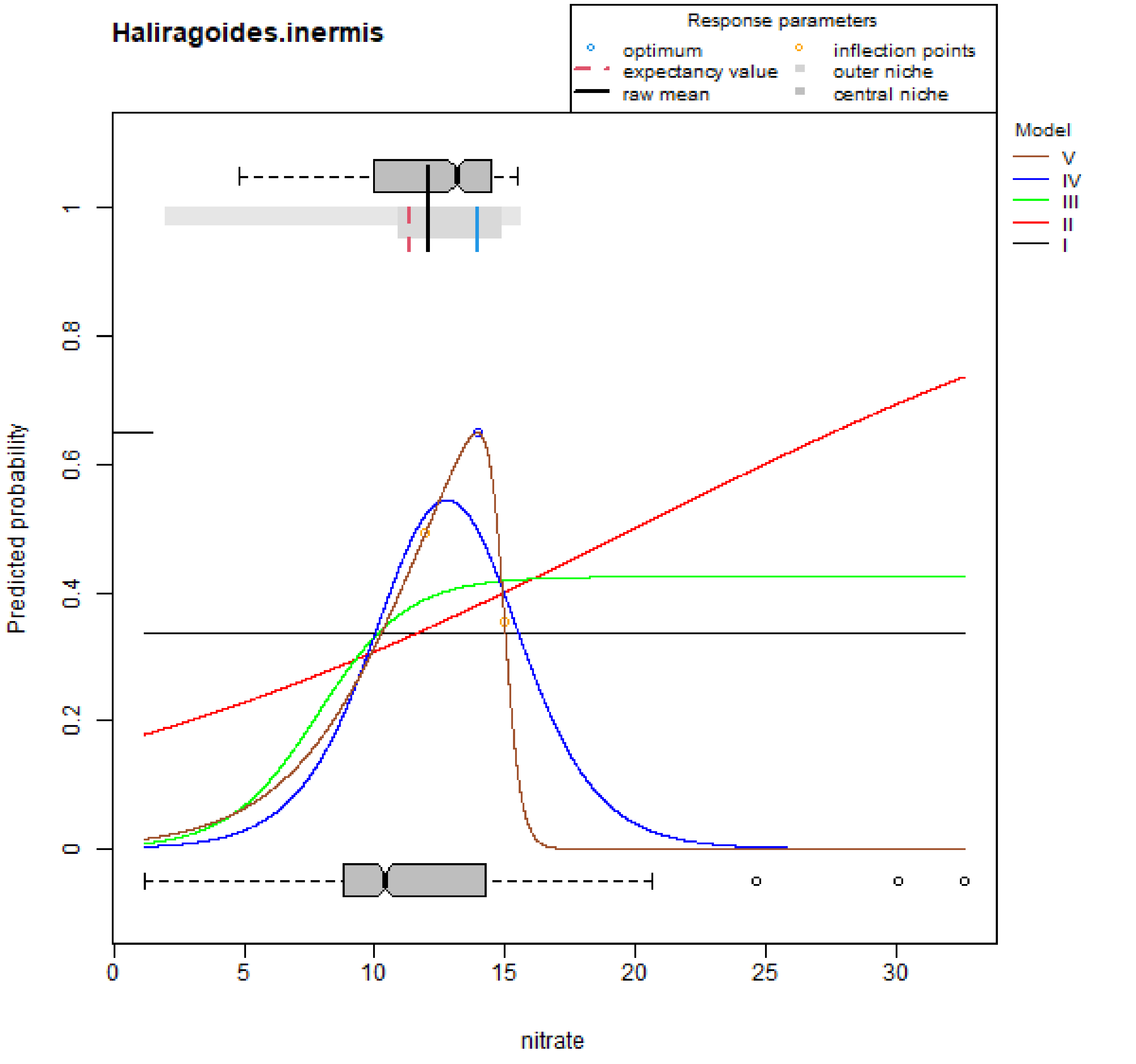

Haliragoides.inermis
Response parameters
optimum
expectancy value
raw mean
inflection points
outer niche
central niche
Model
V
IV
III
II
I
Predicted probability
0
0.2
0.4
0.6
0.8
1
0
5
10
15
20
25
30
nitrate

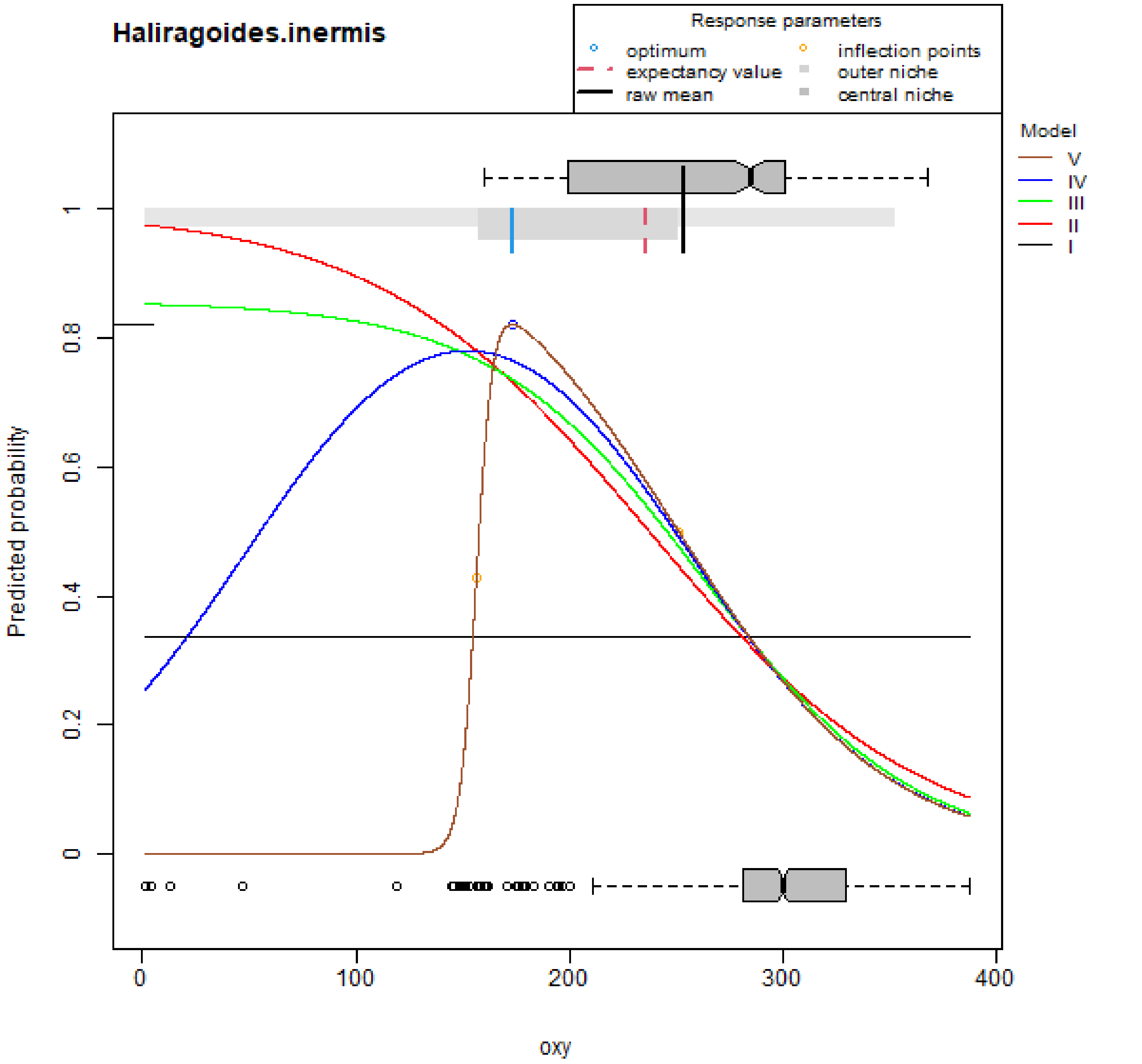

Haliragoides.inermis
Response parameters
optimum
expectancy value
raw mean
inflection points
outer niche
central niche
Model
V
IV
III
II
I
Predicted probability
0
0.2
0.4
0.6
0.8
1
0
100
200
300
400
oxy

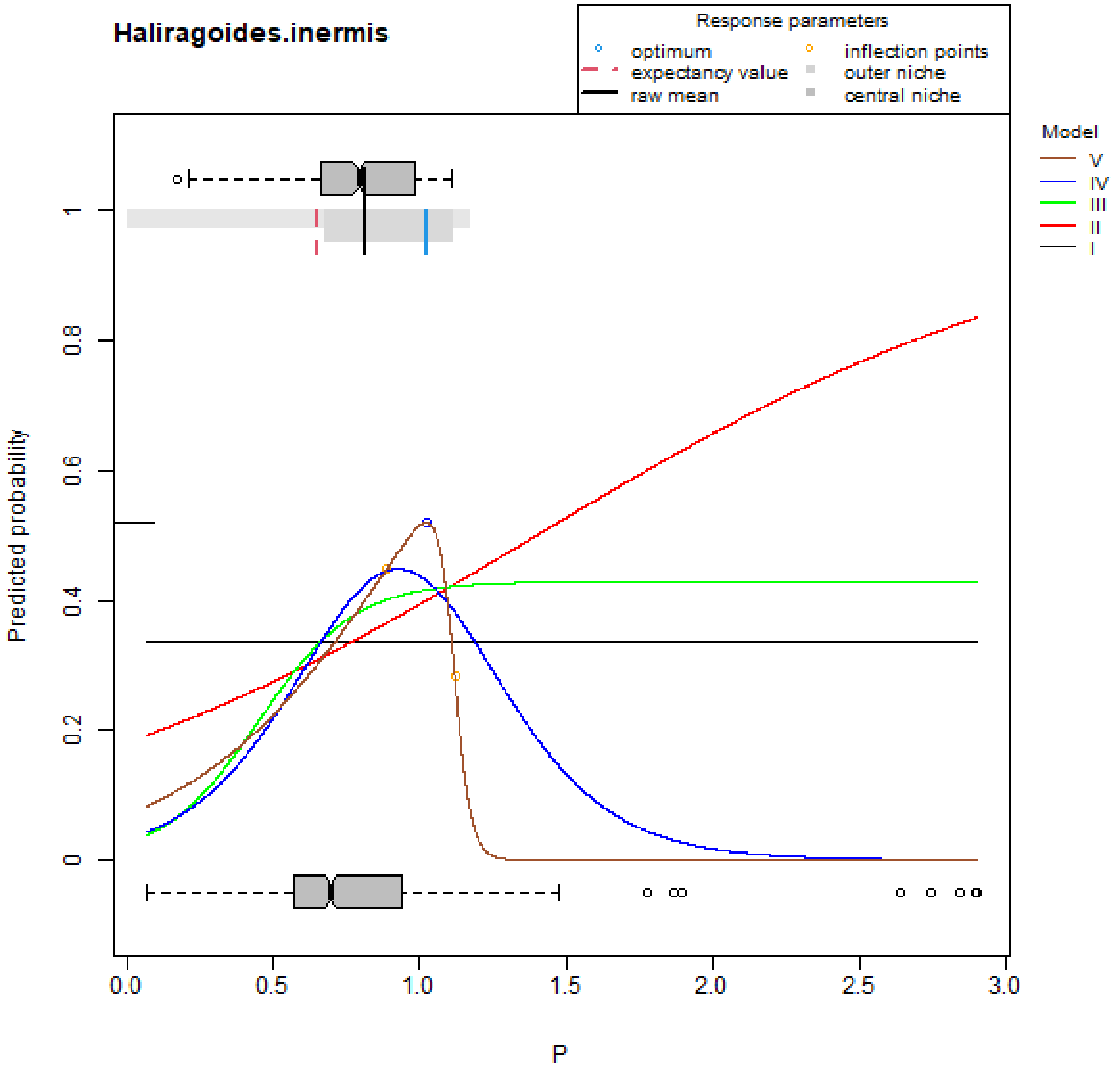
Haliragoides.inermis
Response parameters
optimum
expectancy value
raw mean
inflection points
outer niche
central niche
Model
V
IV
III
II
I
Predicted probability
0
0.2
0.4
0.6
0.8
1
0.0
0.5
1.0
1.5
2.0
2.5
3.0
P

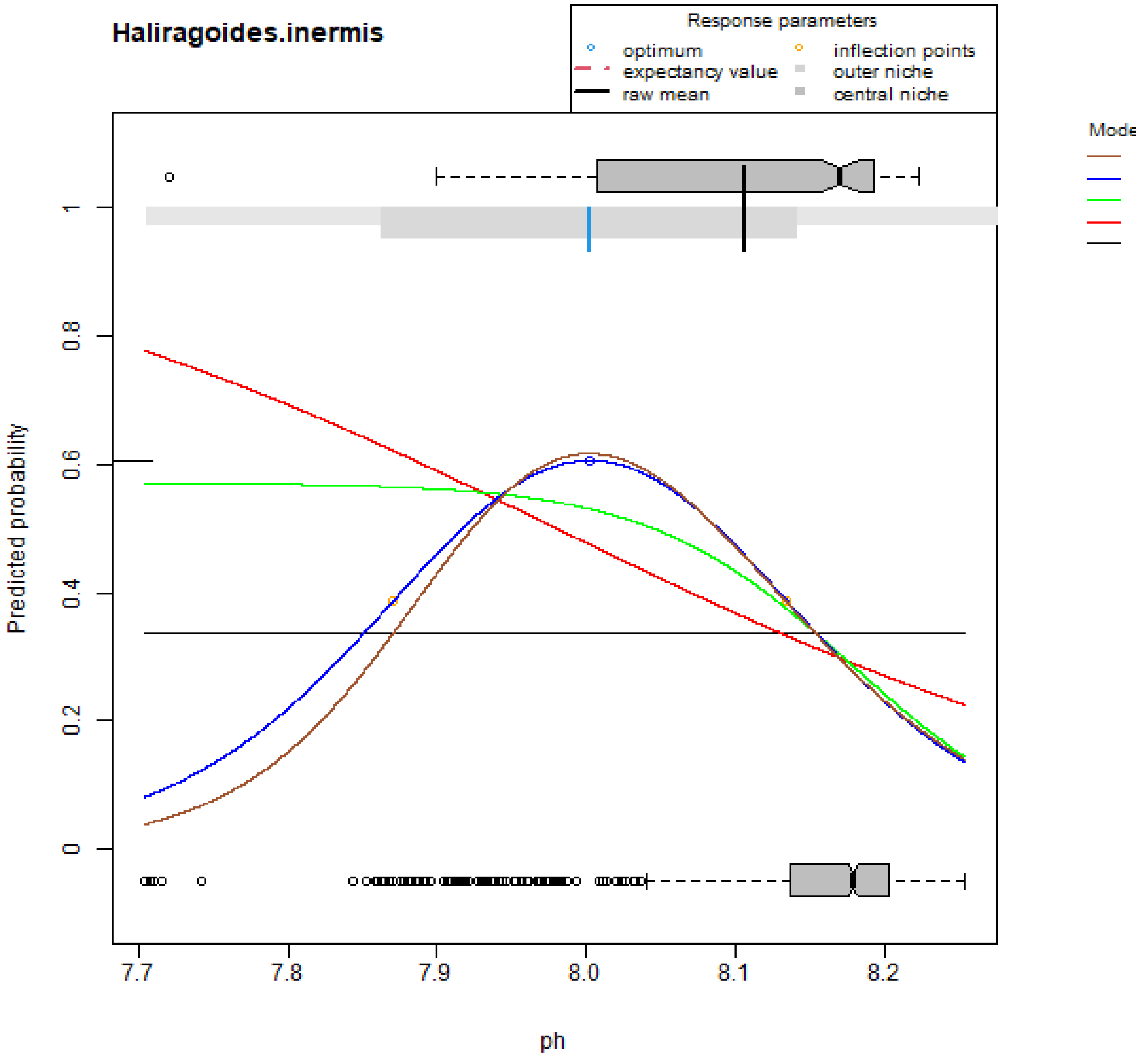

Haliragoides.inermis
Response parameters
optimum
expectancy value
raw mean
inflection points
outer niche
central niche
Mode
Predicted probability
0
0.2
0.4
0.6
0.8
1
7.7
7.8
7.9
8.0
8.1
8.2
ph

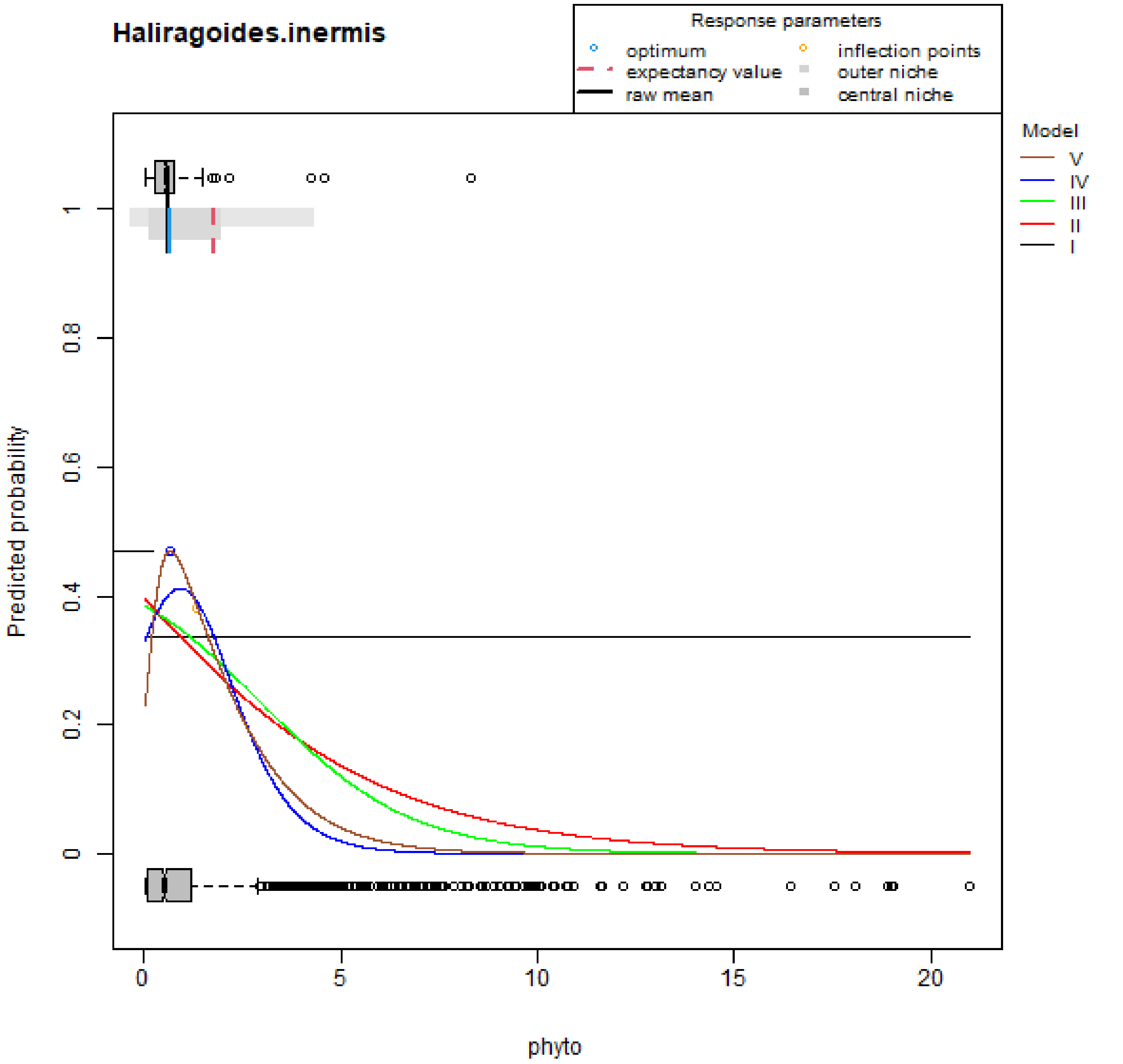

Haliragoides.inermis
Response parameters
optimum
expectancy value
raw mean
inflection points
outer niche
central niche
Model
V
IV
III
II
I
Predicted probability
0
0.2
0.4
0.6
0.8
1
0
5
10
15
20
phyto

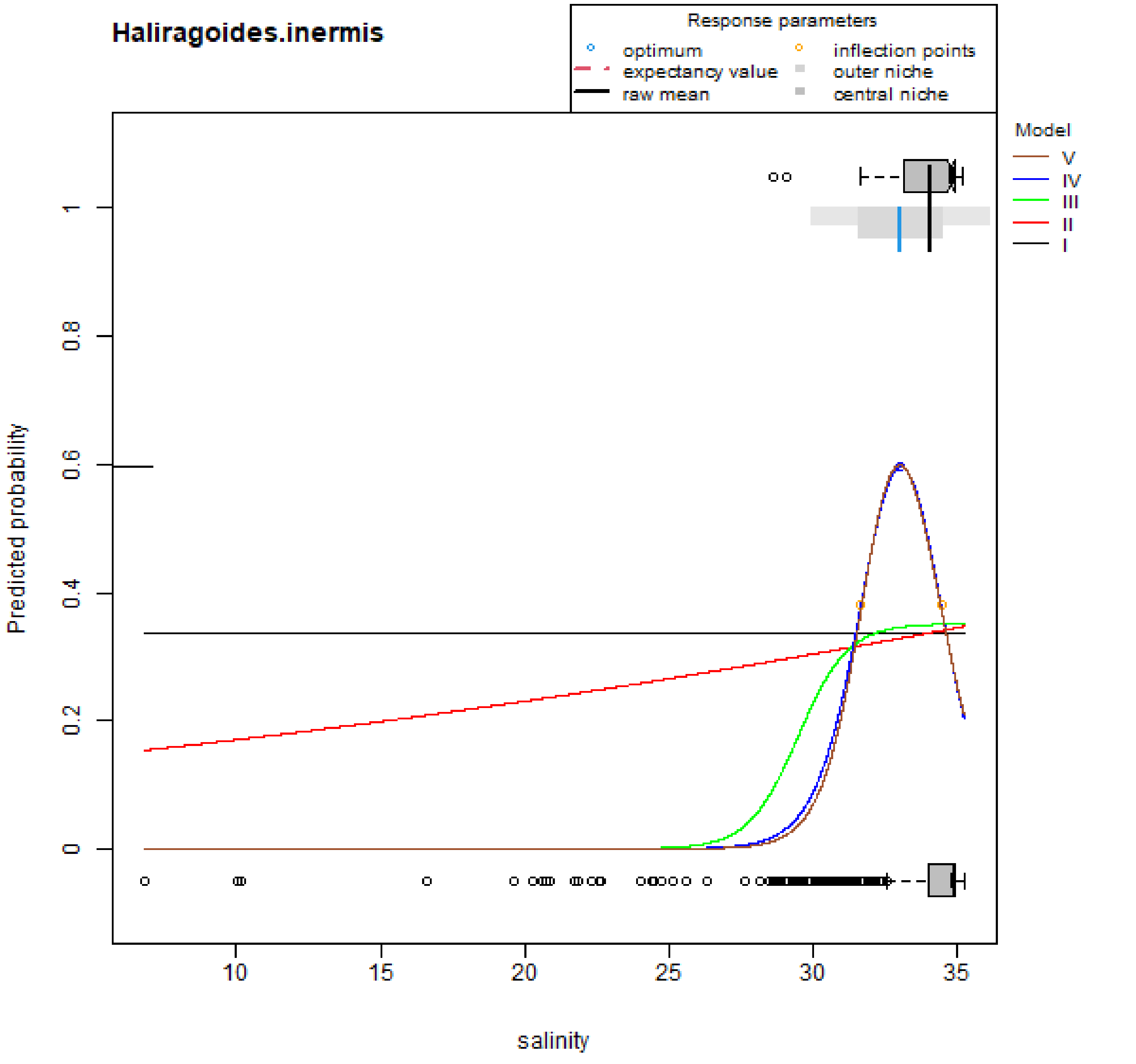

Haliragoides.inermis
Response parameters
optimum
expectancy value
raw mean
inflection points
outer niche
central niche
Model
V
IV
III
II
I
Predicted probability
0
0.2
0.4
0.6
0.8
1
10
15
20
25
30
35
salinity

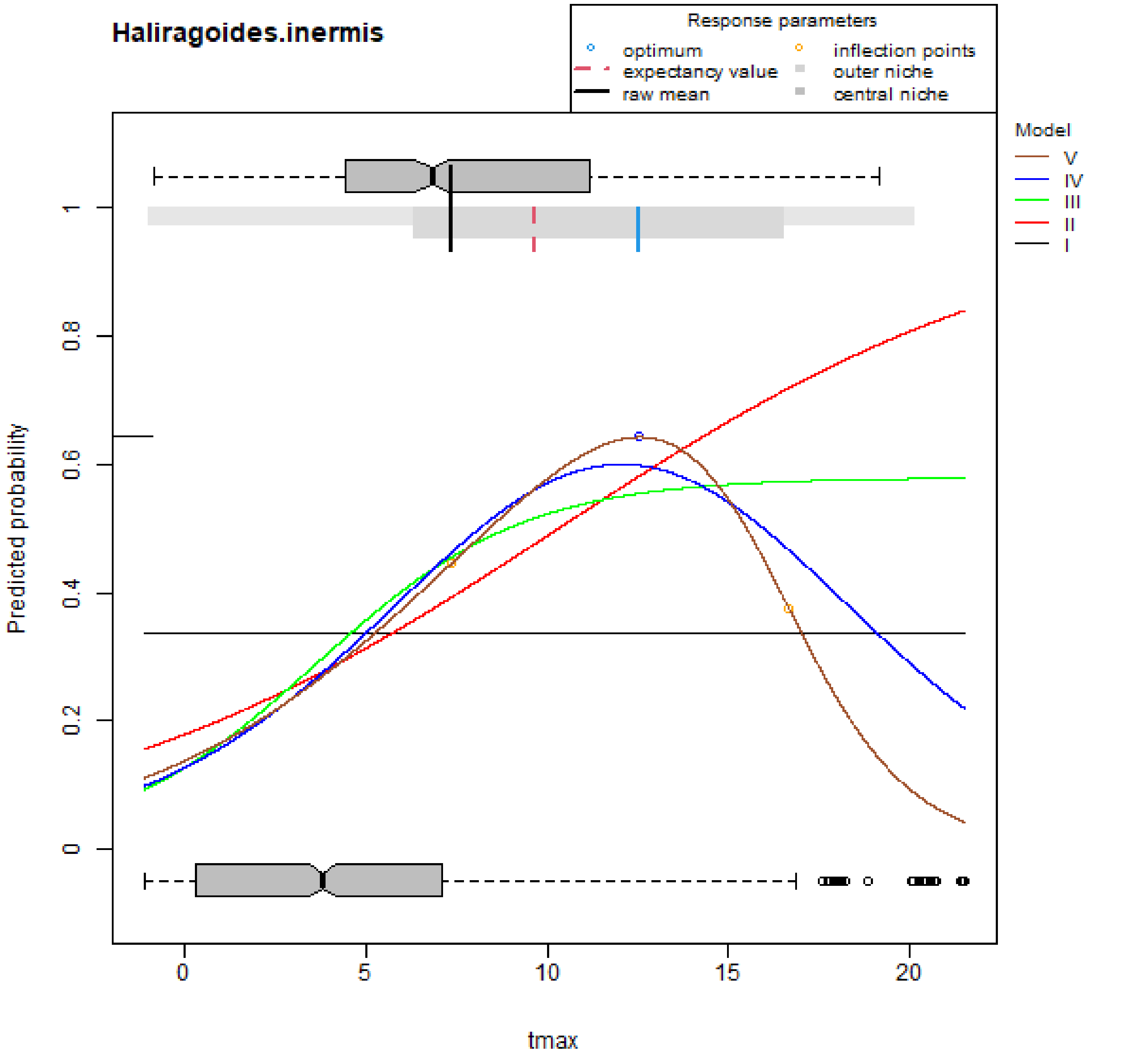
Haliragoides.inermis
Response parameters
optimum
expectancy value
raw mean
inflection points
outer niche
central niche
Model
V
IV
III
II
I
Predicted probability
0
0.2
0.4
0.6
0.8
1
0
5
10
15
20
tmax

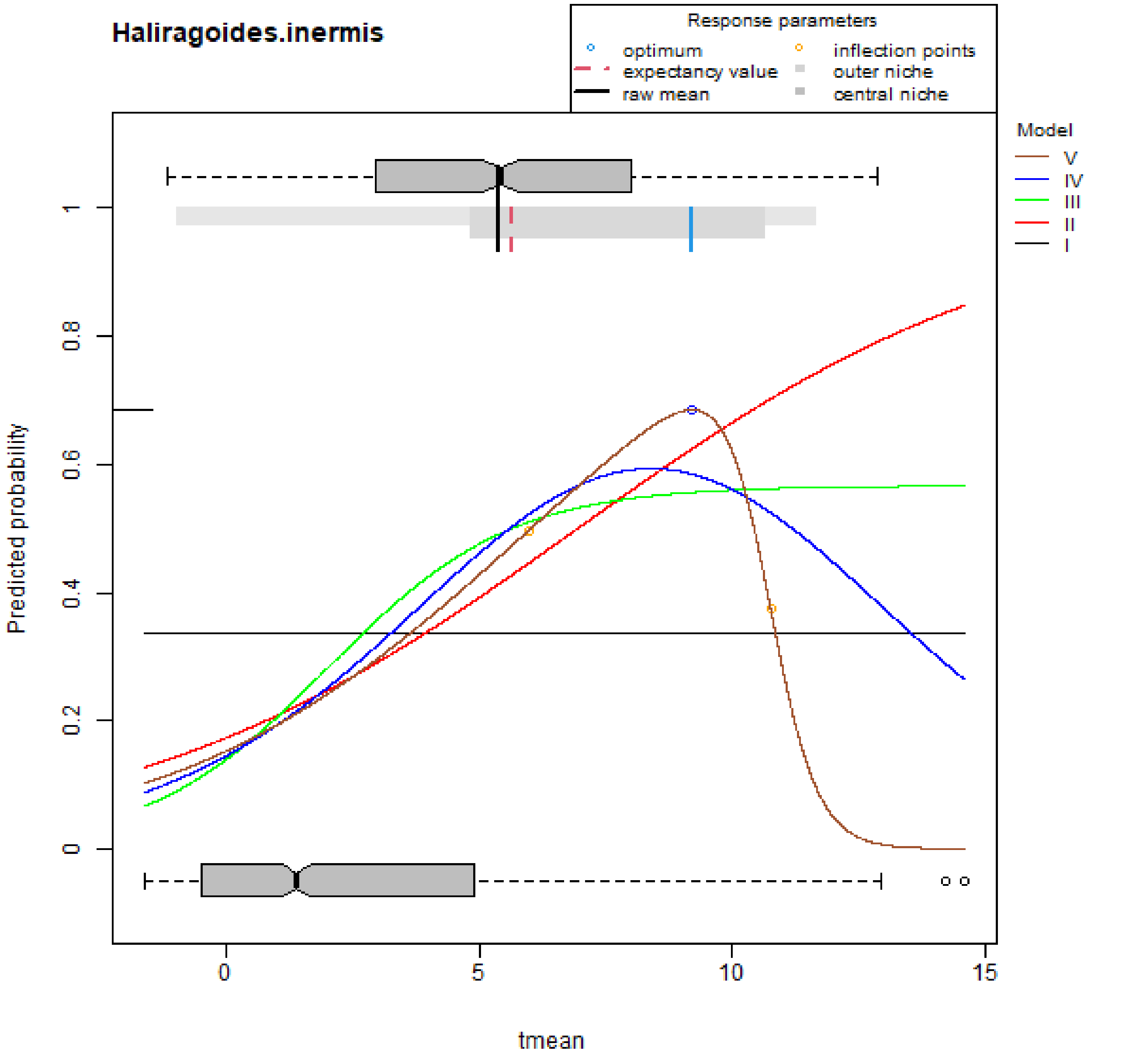

Haliragoides.inermis
Response parameters
optimum
expectancy value
raw mean
inflection points
outer niche
central niche
Model
V
IV
III
II
I
Predicted probability
0
0.2
0.4
0.6
0.8
1
0
5
10
15
tmean

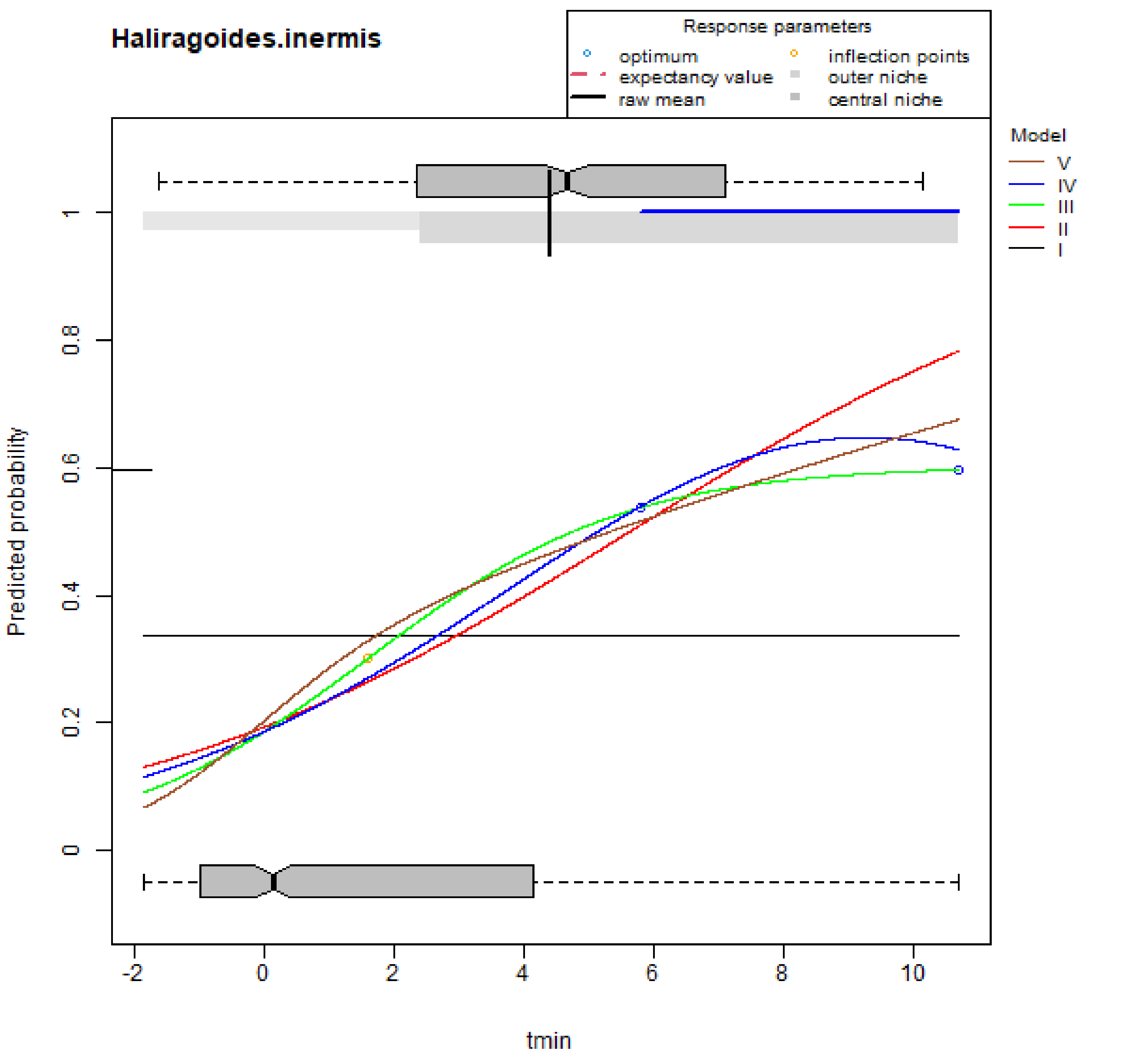
Haliragoides.inermis
Response parameters
optimum
expectancy value
raw mean
inflection points
outer niche
central niche
Model
V
IV
III
II
I
Predicted probability
0
0.2
0.4
0.6
0.8
1
-2
0
2
4
6
8
10
tmin

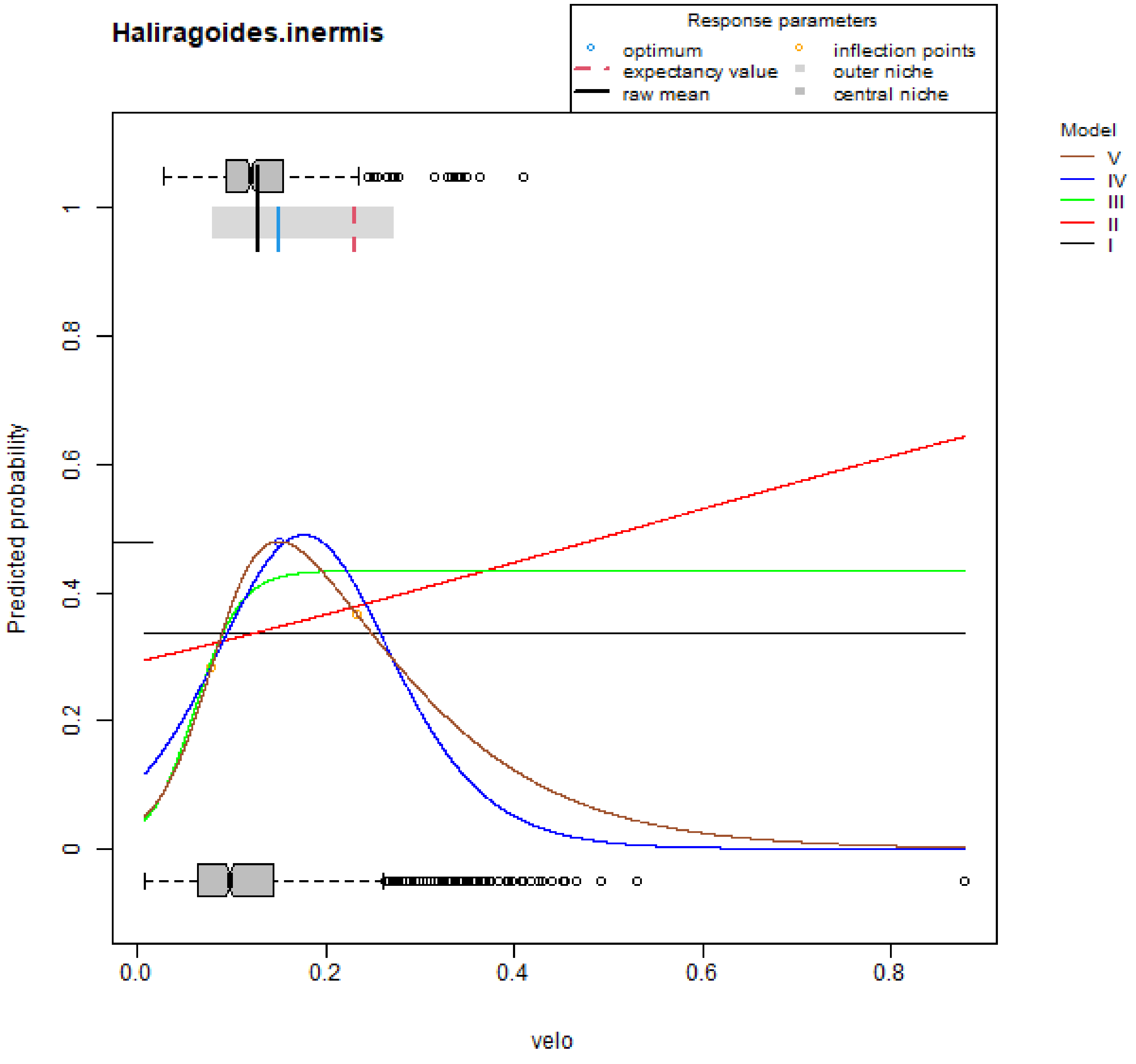

Haliragoides.inermis
Response parameters
optimum
expectancy value
raw mean
inflection points
outer niche
central niche
Model
V
IV
III
II
I
Predicted probability
0
0.2
0.4
0.6
0.8
1
0.0
0.2
0.4
0.6
0.8
velo

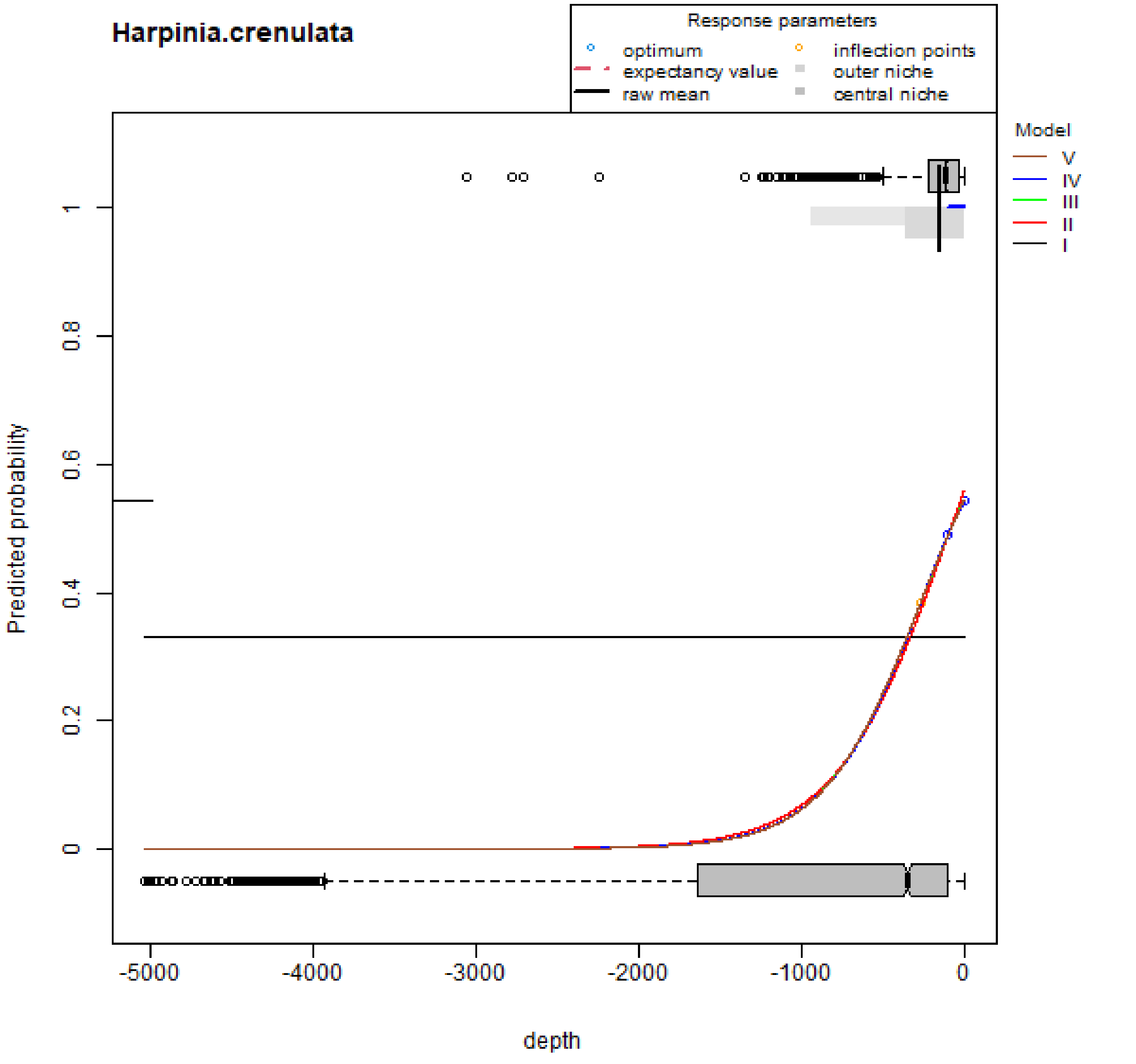

Harpinia.crenulata
Response parameters
optimum
expectancy value
raw mean
inflection points
outer niche
central niche
Model
V
IV
III
II
I
Predicted probability
0
0.2
0.4
0.6
0.8
1
-5000
-4000
-3000
-2000
-1000
0
depth

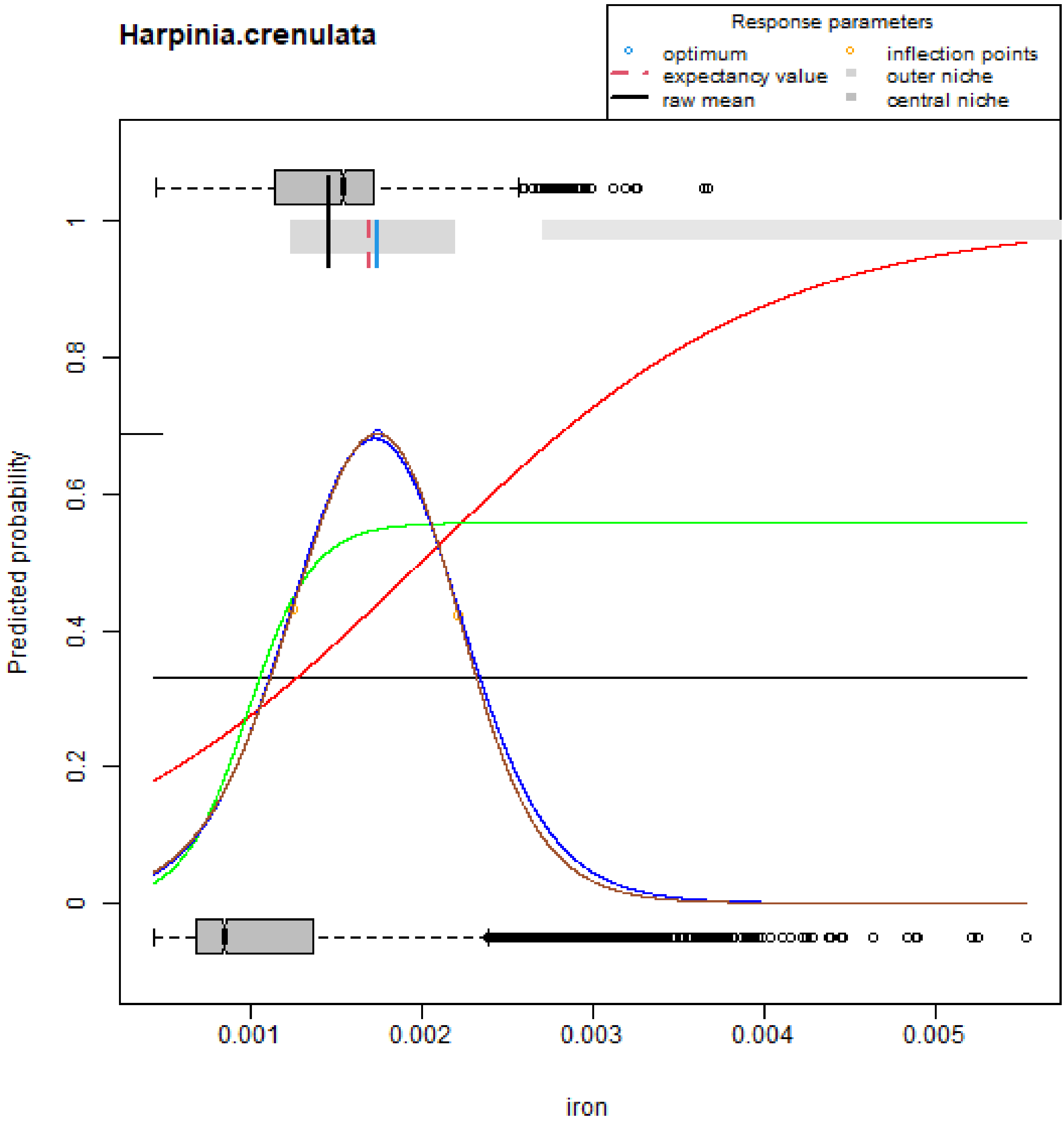

Harpinia.crenulata
Response parameters
optimum
expectancy value
raw mean
inflection points
outer niche
central niche
Predicted probability
0
0.2
0.4
0.6
0.8
1
0.001
0.002
0.003
0.004
0.005
iron

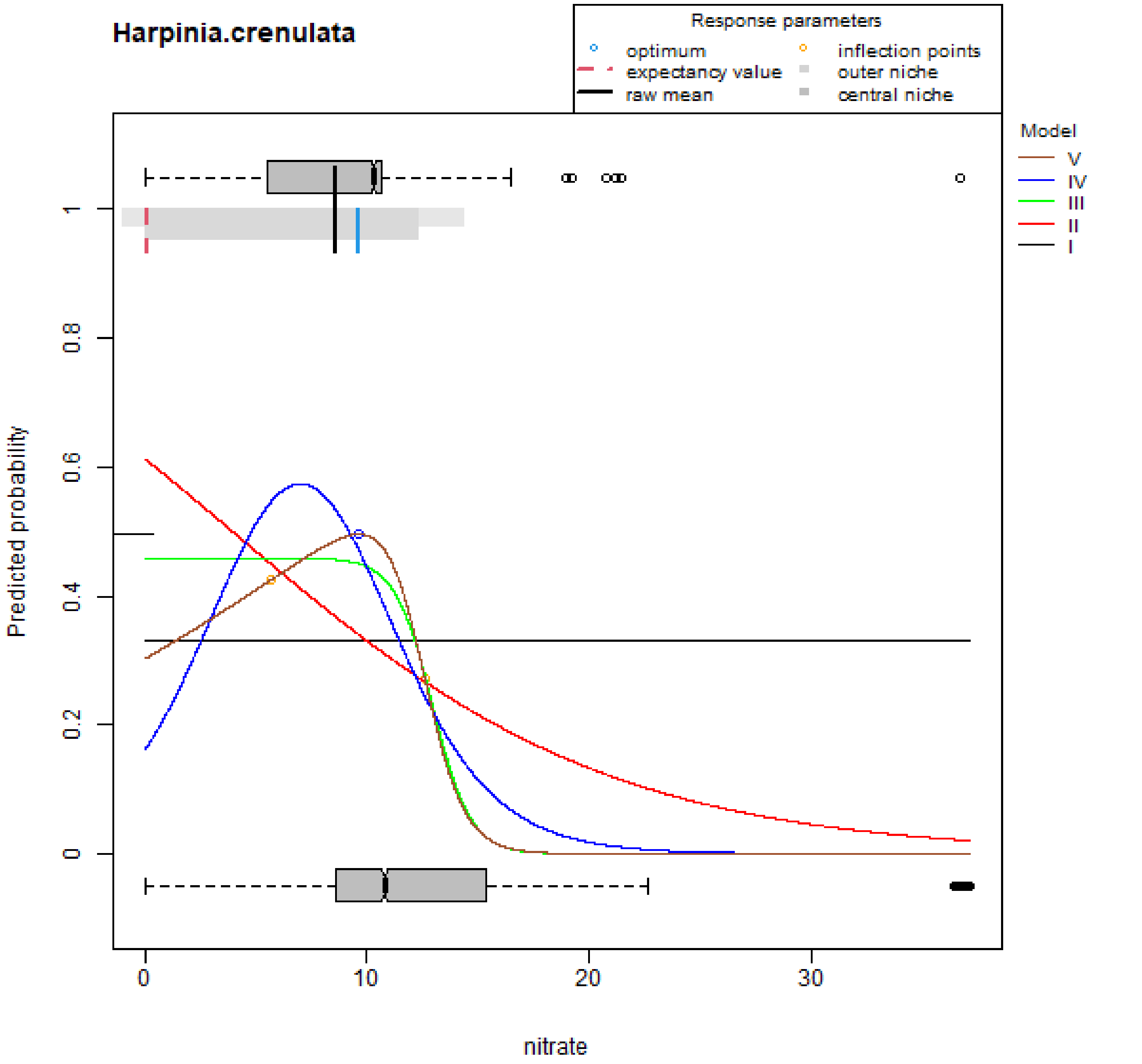
Harpinia.crenulata
Response parameters
optimum
expectancy value
raw mean
inflection points
outer niche
central niche
Model
V
IV
III
II
I
Predicted probability
0
0.2
0.4
0.6
0.8
1
0
10
20
30
nitrate

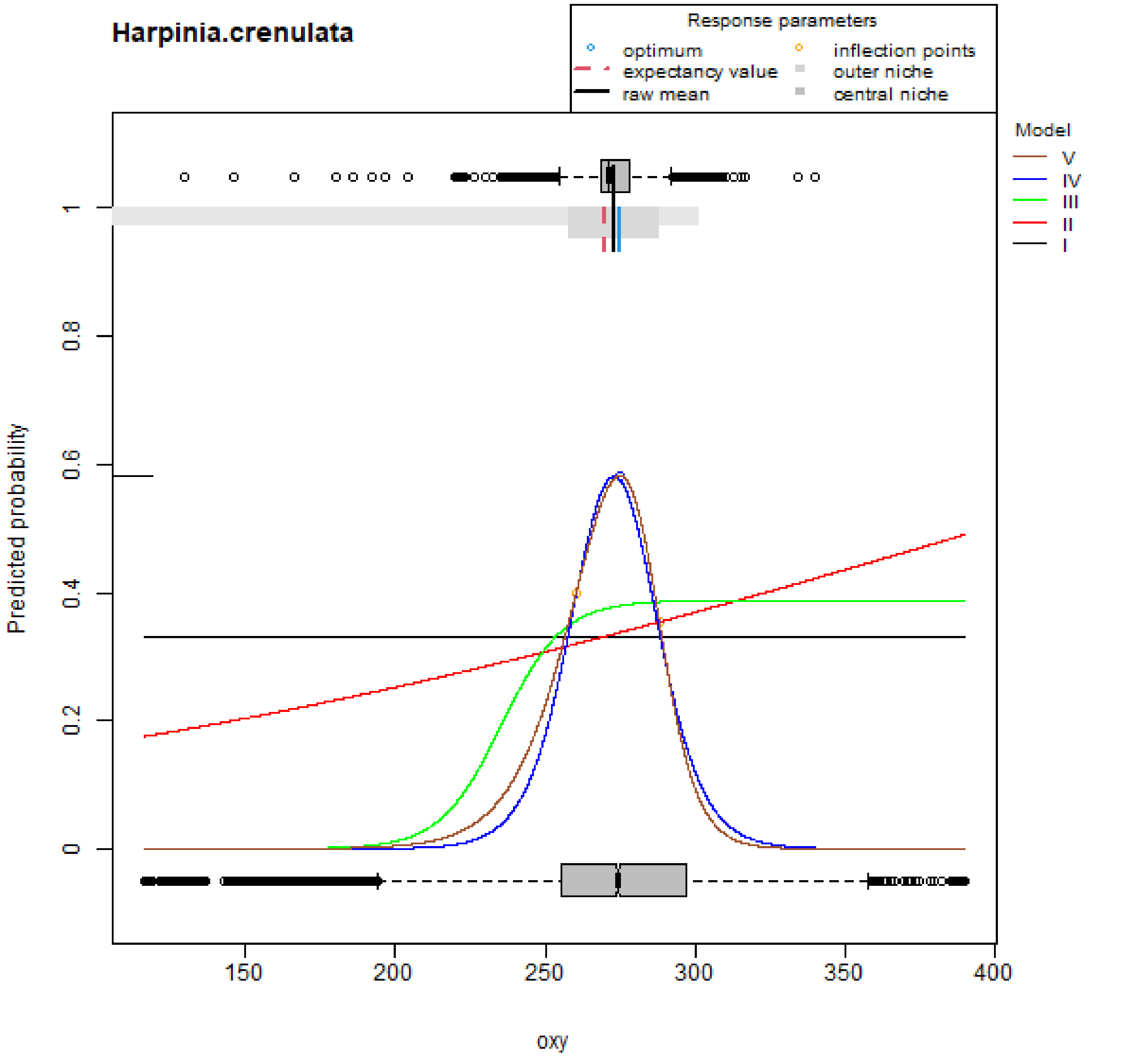

Harpinia.crenulata
Response parameters
optimum
expectancy value
raw mean
inflection points
outer niche
central niche
Model
V
IV
III
II
I
Predicted probability
0
0.2
0.4
0.6
0.8
1
150
200
250
300
350
400
oxy

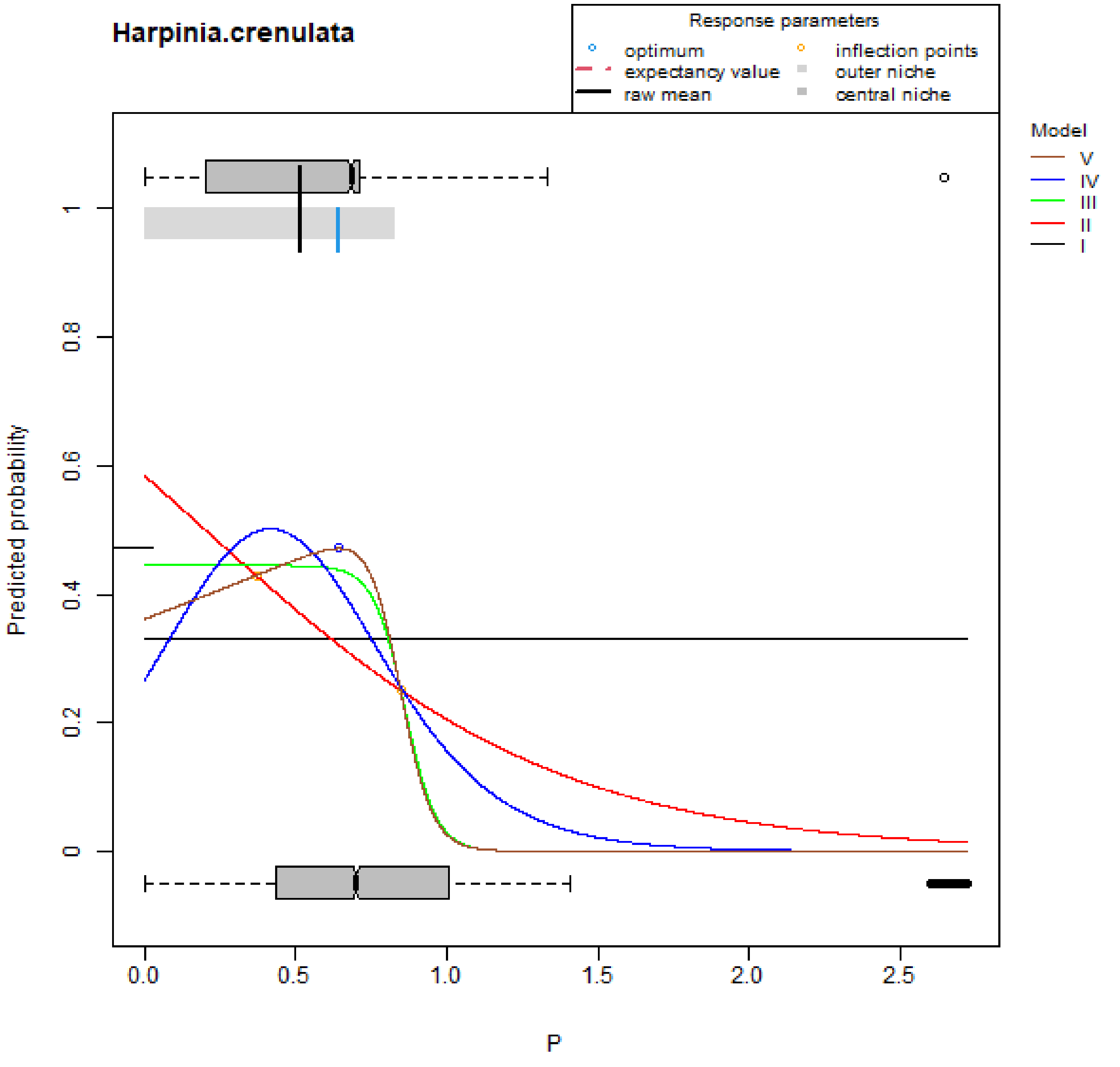

Harpinia.crenulata
Response parameters
optimum
inflection points
expectancy value
outer niche
raw mean
central niche
Model
V
IV
III
II
I
Predicted probability
0
0.2
0.4
0.6
0.8
1
0.0
0.5
1.0
1.5
2.0
2.5
P

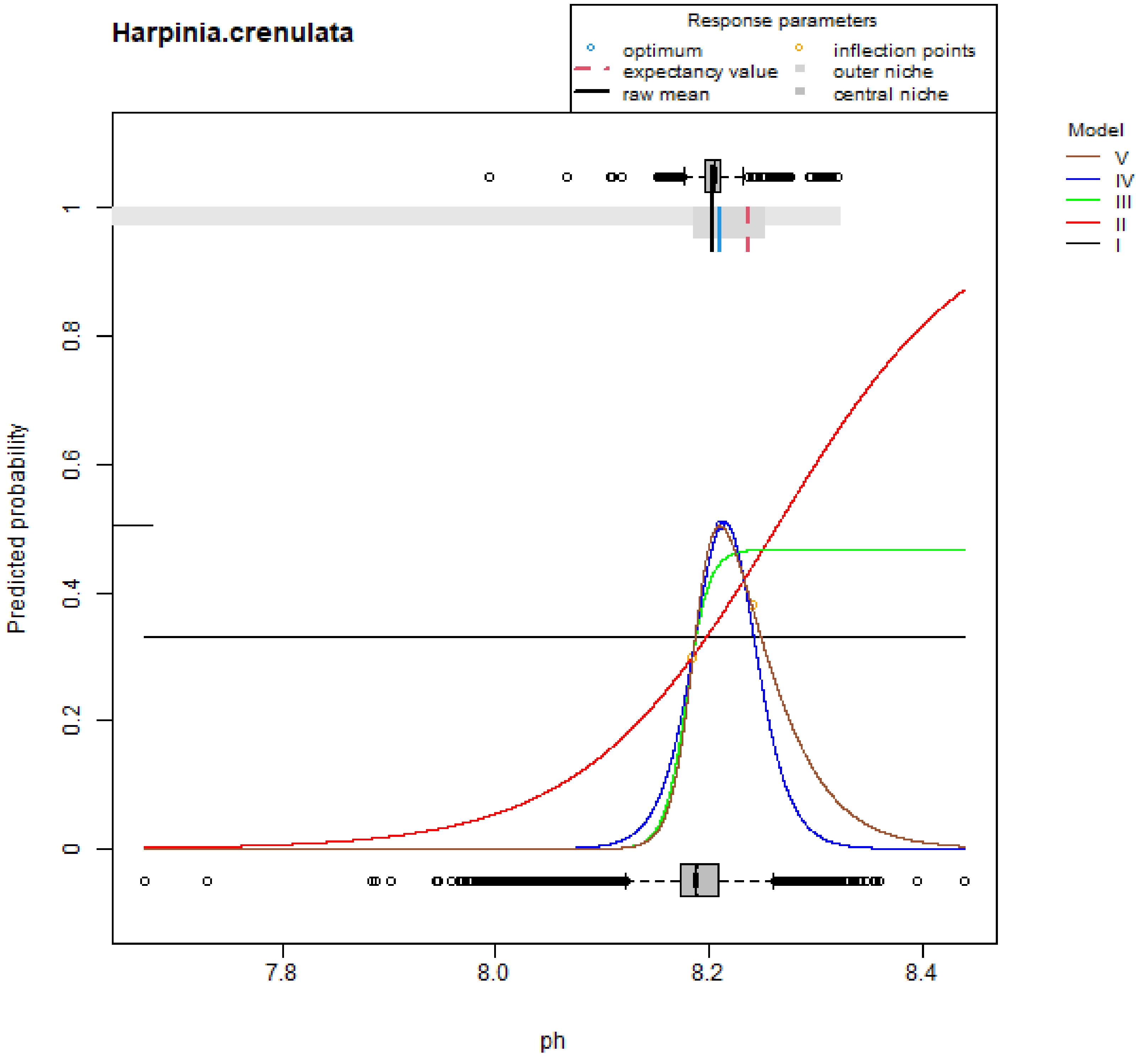

Harpinia.crenulata
Response parameters
optimum
expectancy value
raw mean
inflection points
outer niche
central niche
Model
V
IV
III
II
I
Predicted probability
0
0.2
0.4
0.6
0.8
1
7.8
8.0
8.2
8.4
ph

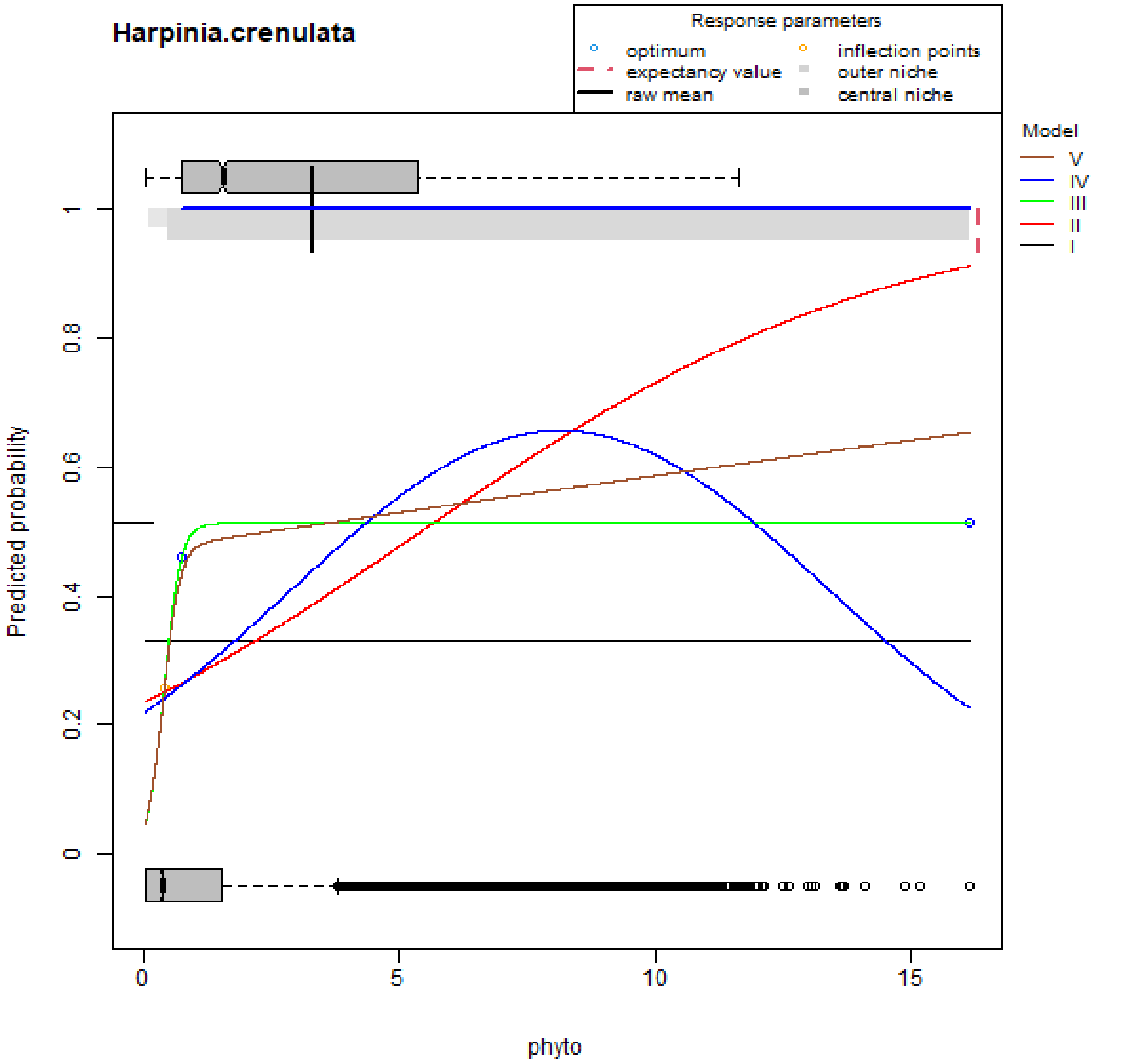

Harpinia.crenulata
Response parameters
optimum
expectancy value
raw mean
inflection points
outer niche
central niche
Model
V
IV
III
II
I
Predicted probability
0
0.2
0.4
0.6
0.8
1
0
5
10
15
phyto

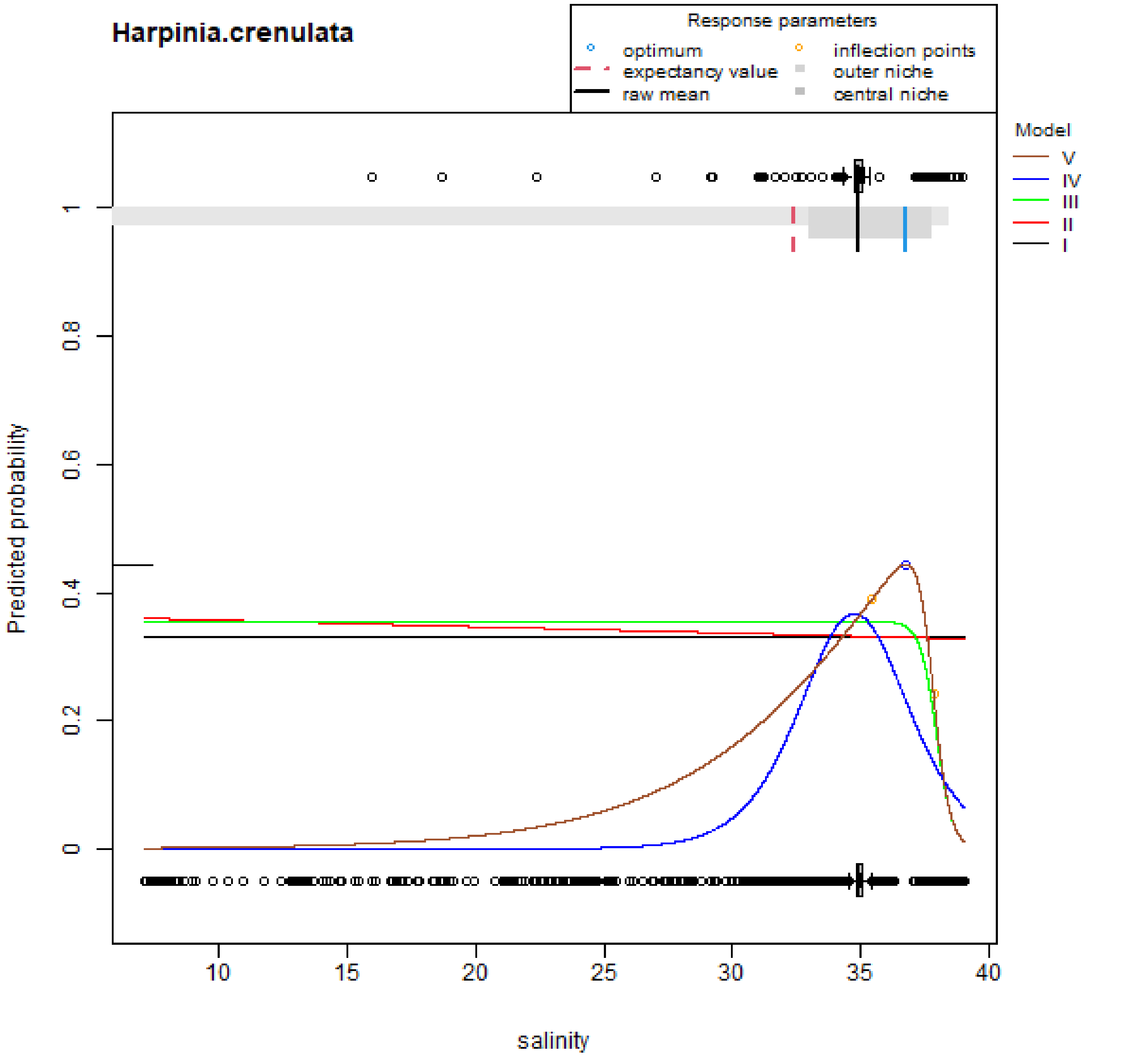
Harpinia.crenulata
Response parameters
optimum
expectancy value
raw mean
inflection points
outer niche
central niche
Model
V
IV
III
II
I
Predicted probability
0
0.2
0.4
0.6
0.8
1
10
15
20
25
30
35
40
salinity

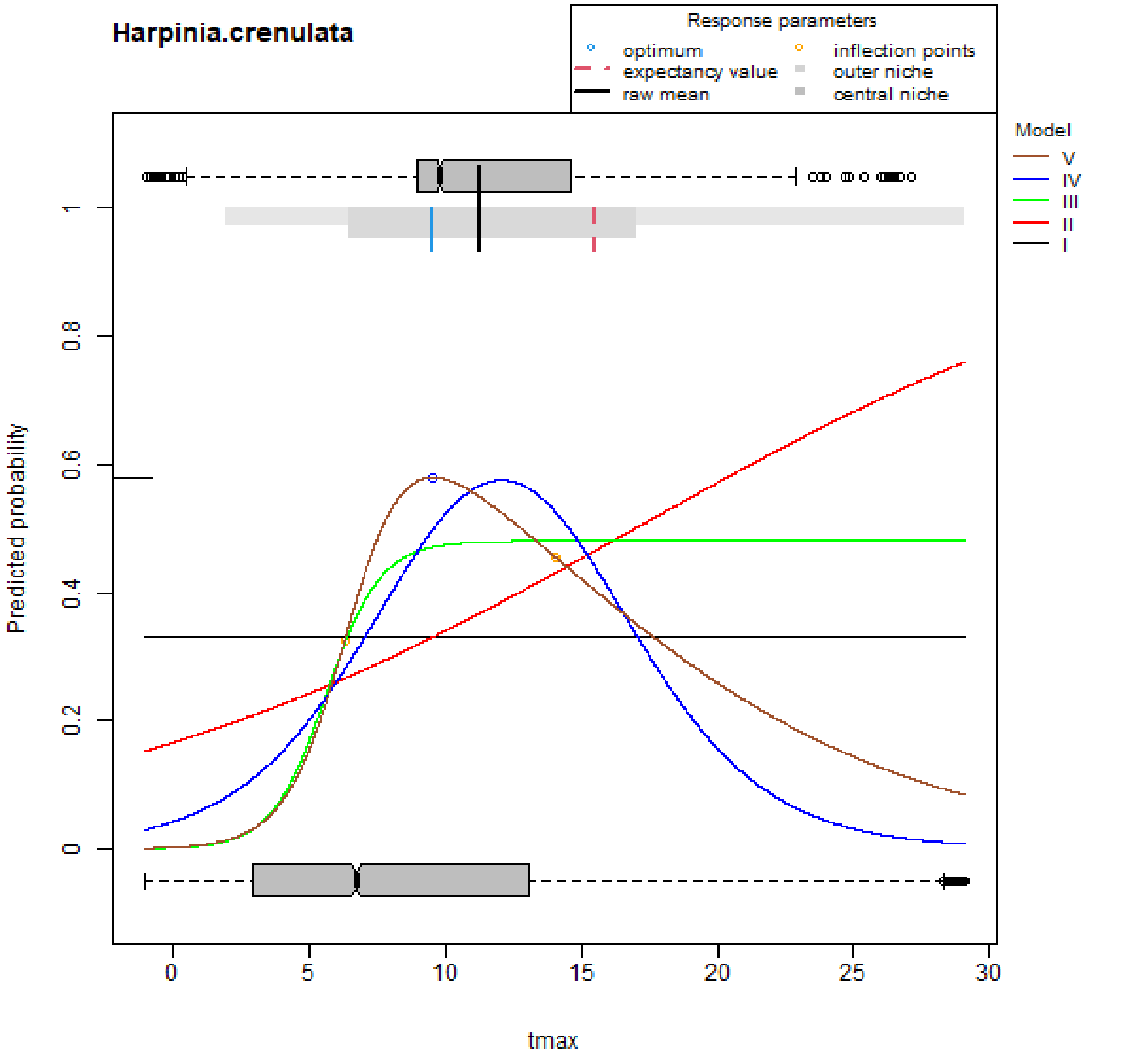

Harpinia.crenulata
Response parameters
optimum
expectancy value
raw mean
inflection points
outer niche
central niche
Model
V
IV
III
II
I
Predicted probability
0
0.2
0.4
0.6
0.8
1
0
5
10
15
20
25
30
tmax

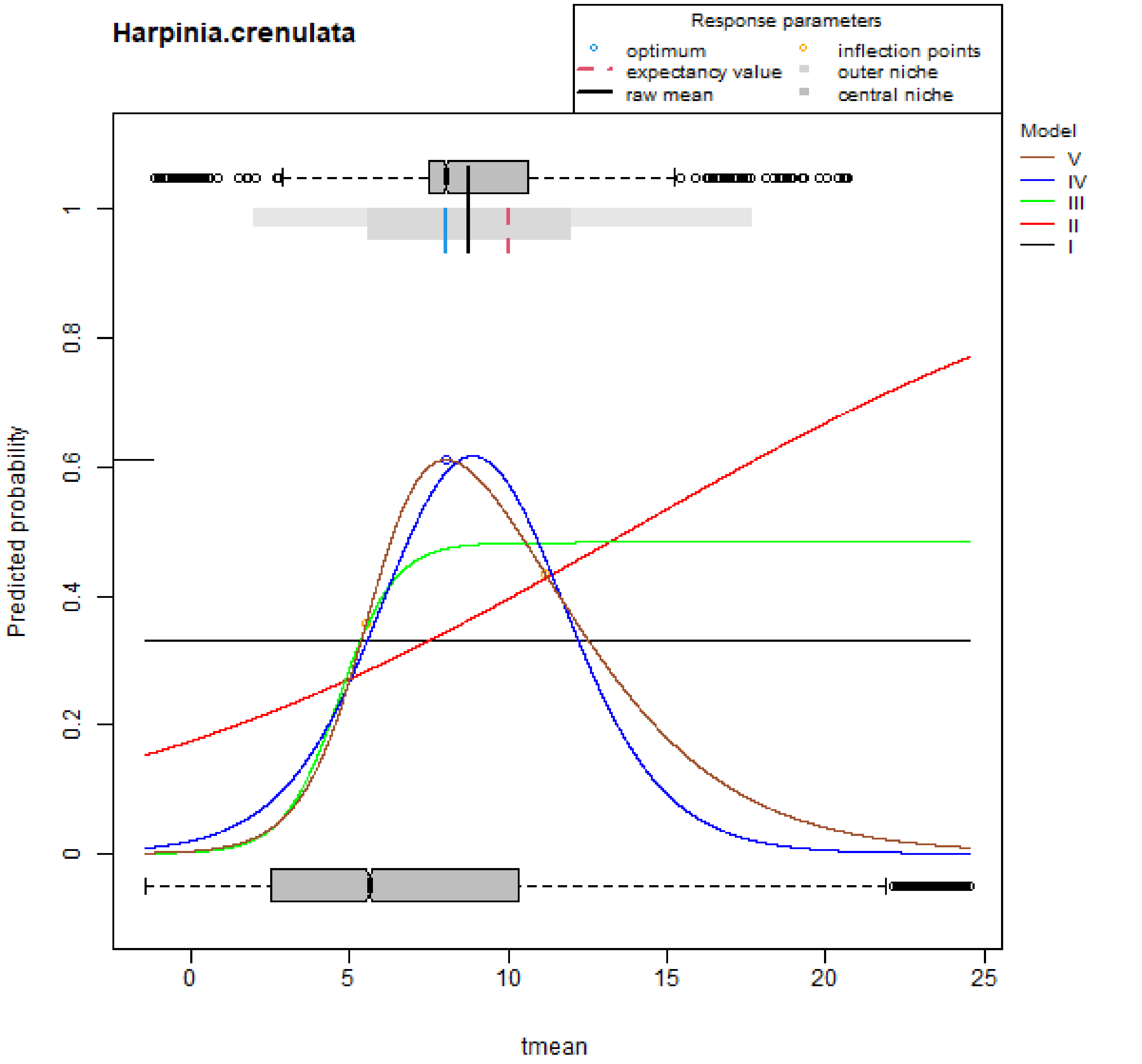

Harpinia.crenulata
Response parameters
optimum
expectancy value
raw mean
inflection points
outer niche
central niche
Model
V
IV
III
II
I
Predicted probability
0
0.2
0.4
0.6
0.8
1
0
5
10
15
20
25
tmean

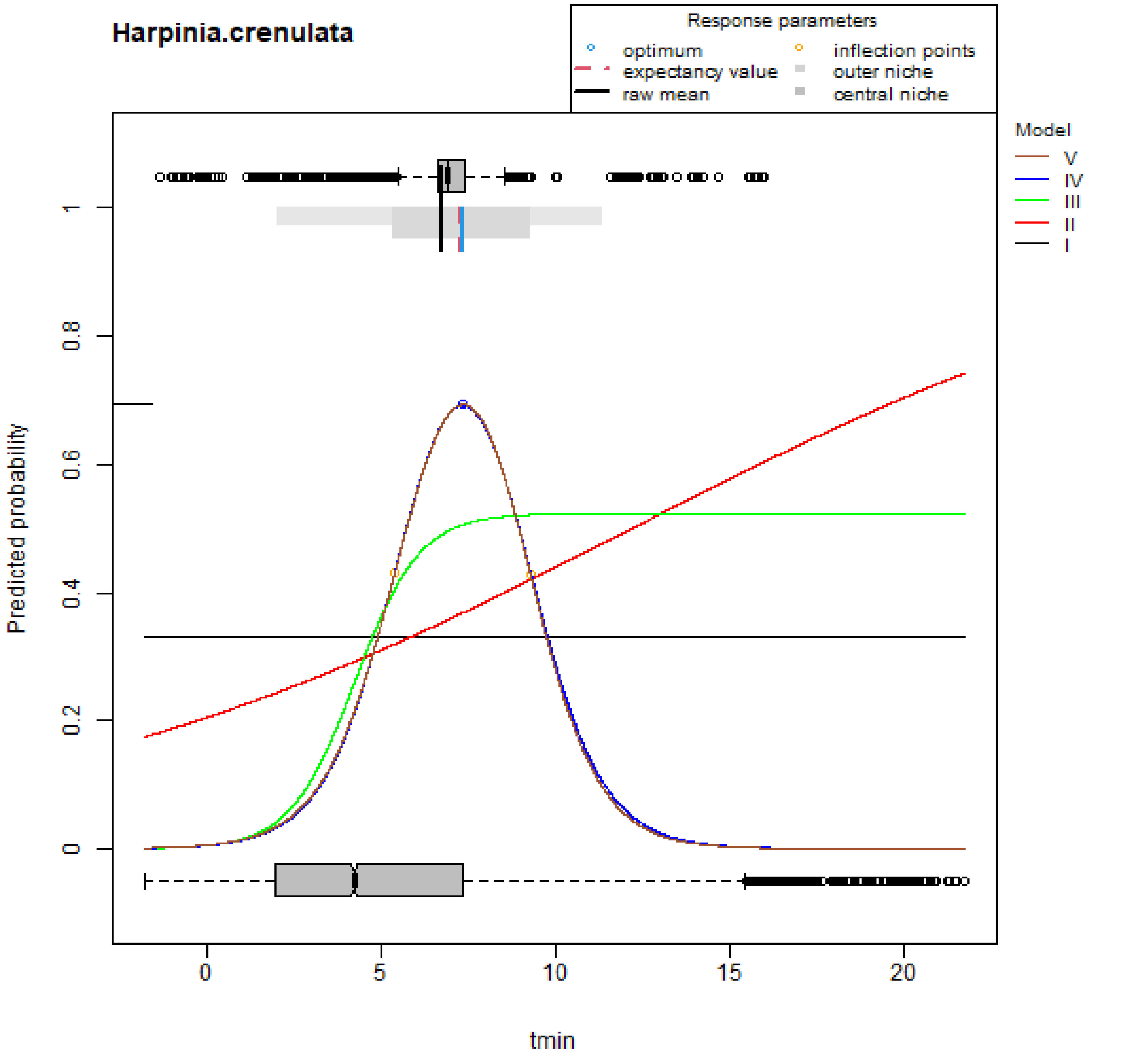

Harpinia.crenulata
Response parameters
optimum
expectancy value
raw mean
inflection points
outer niche
central niche
Model
V
IV
III
II
I
Predicted probability
0
0.2
0.4
0.6
0.8
1
0
5
10
15
20
tmin

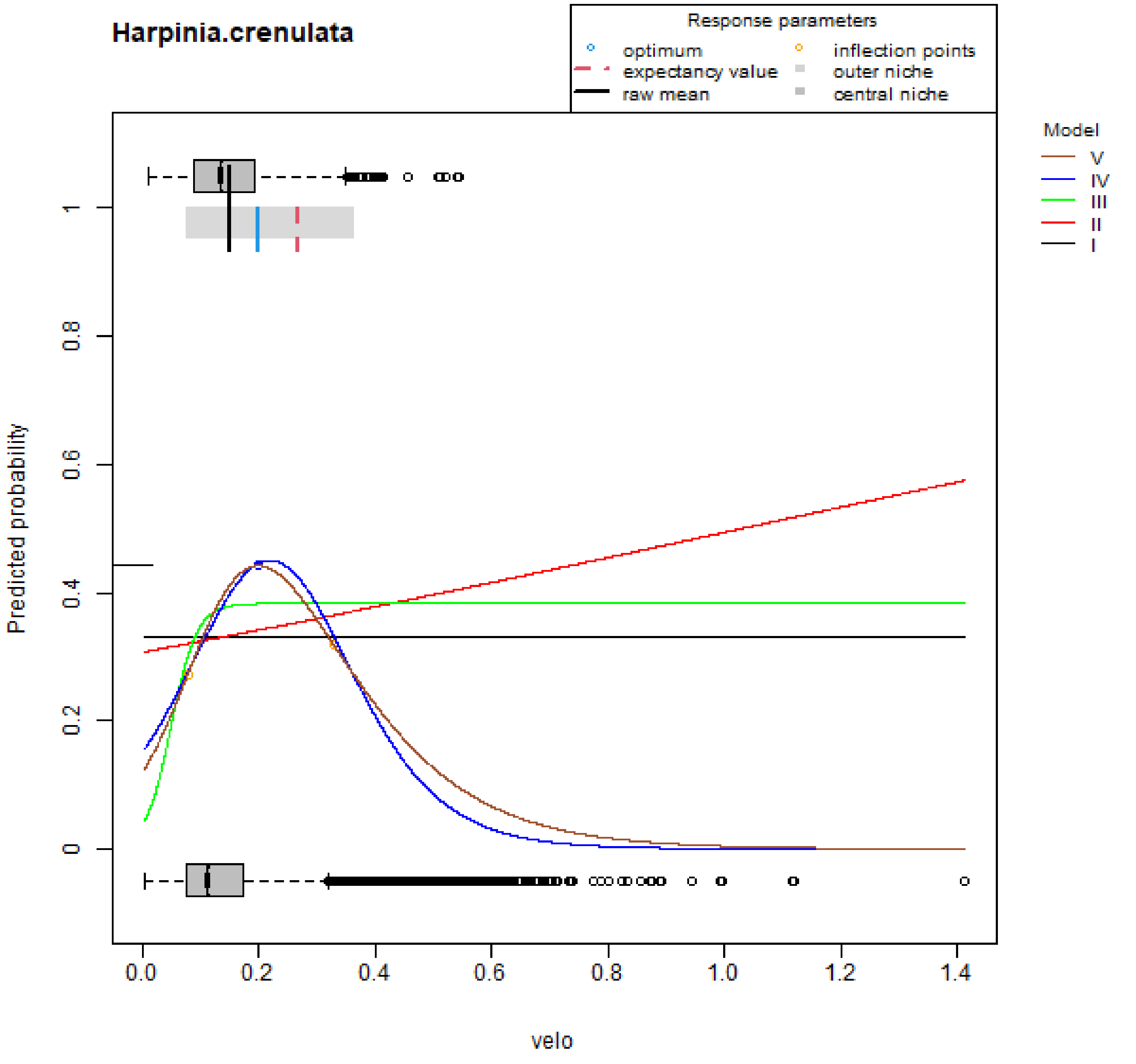

Harpinia.crenulata
Response parameters
optimum
expectancy value
raw mean
inflection points
outer niche
central niche
Model
V
IV
III
II
I
Predicted probability
0
0.2
0.4
0.6
0.8
1
0.0
0.2
0.4
0.6
0.8
1.0
1.2
1.4
velo

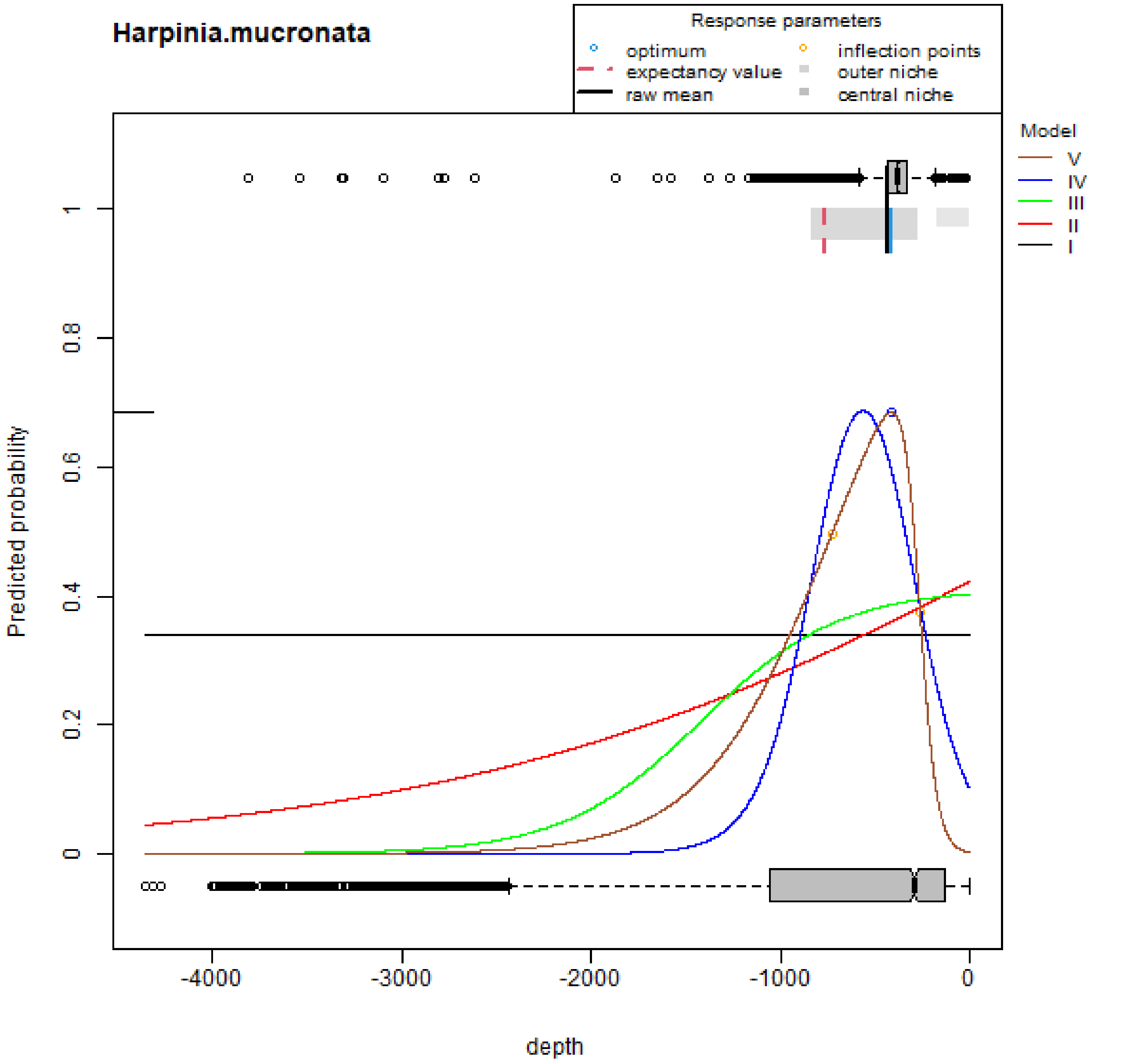

Harpinia.mucronata
Response parameters
optimum
expectancy value
raw mean
inflection points
outer niche
central niche
Model
V
IV
III
II
I
Predicted probability
0
0.2
0.4
0.6
0.8
1
-4000
-3000
-2000
-1000
0
depth

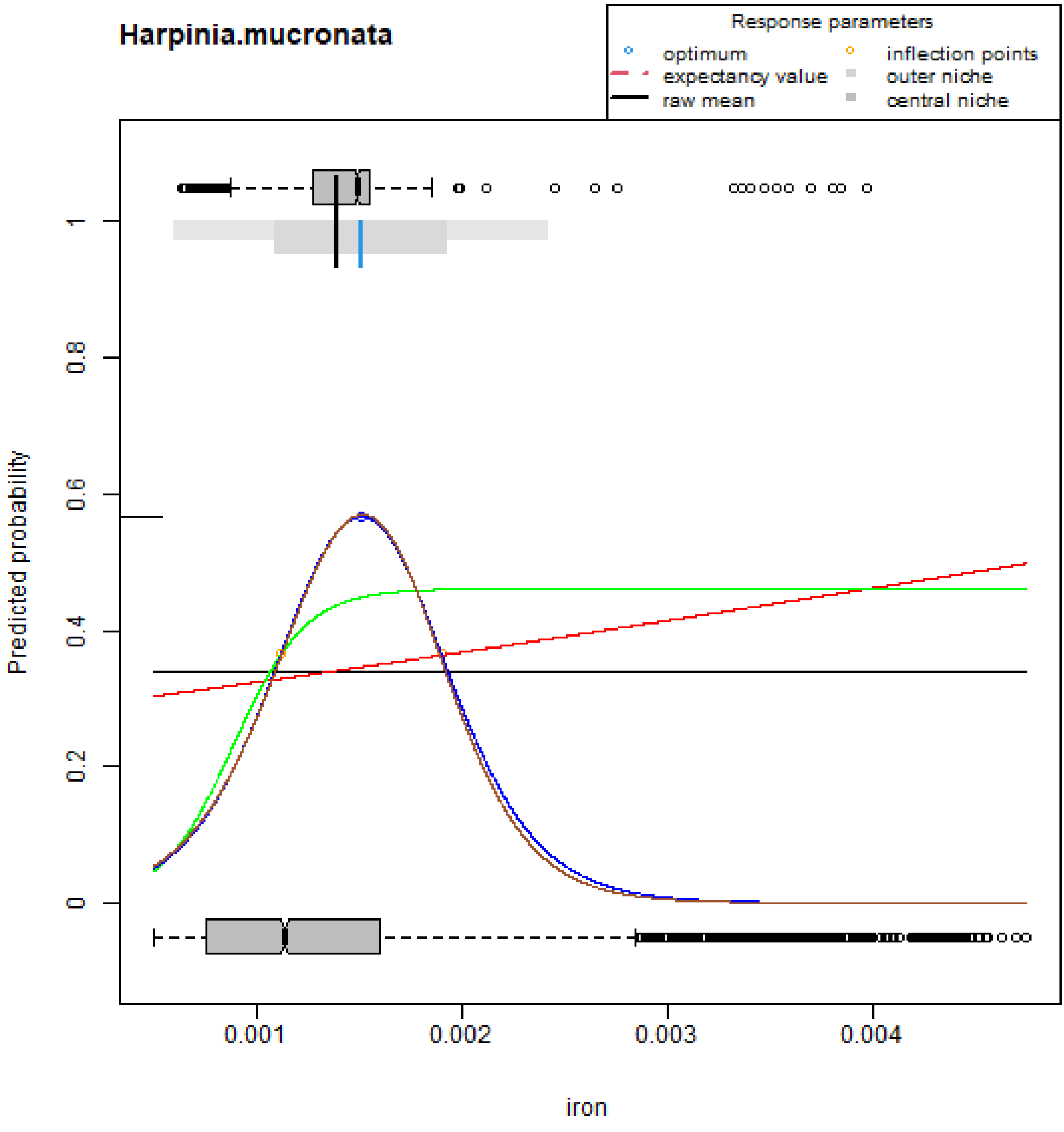

Harpinia.mucronata
Response parameters
optimum
expectancy value
raw mean
inflection points
outer niche
central niche
Predicted probability
0
0.2
0.4
0.6
0.8
1
0.001
0.002
0.003
0.004
iron

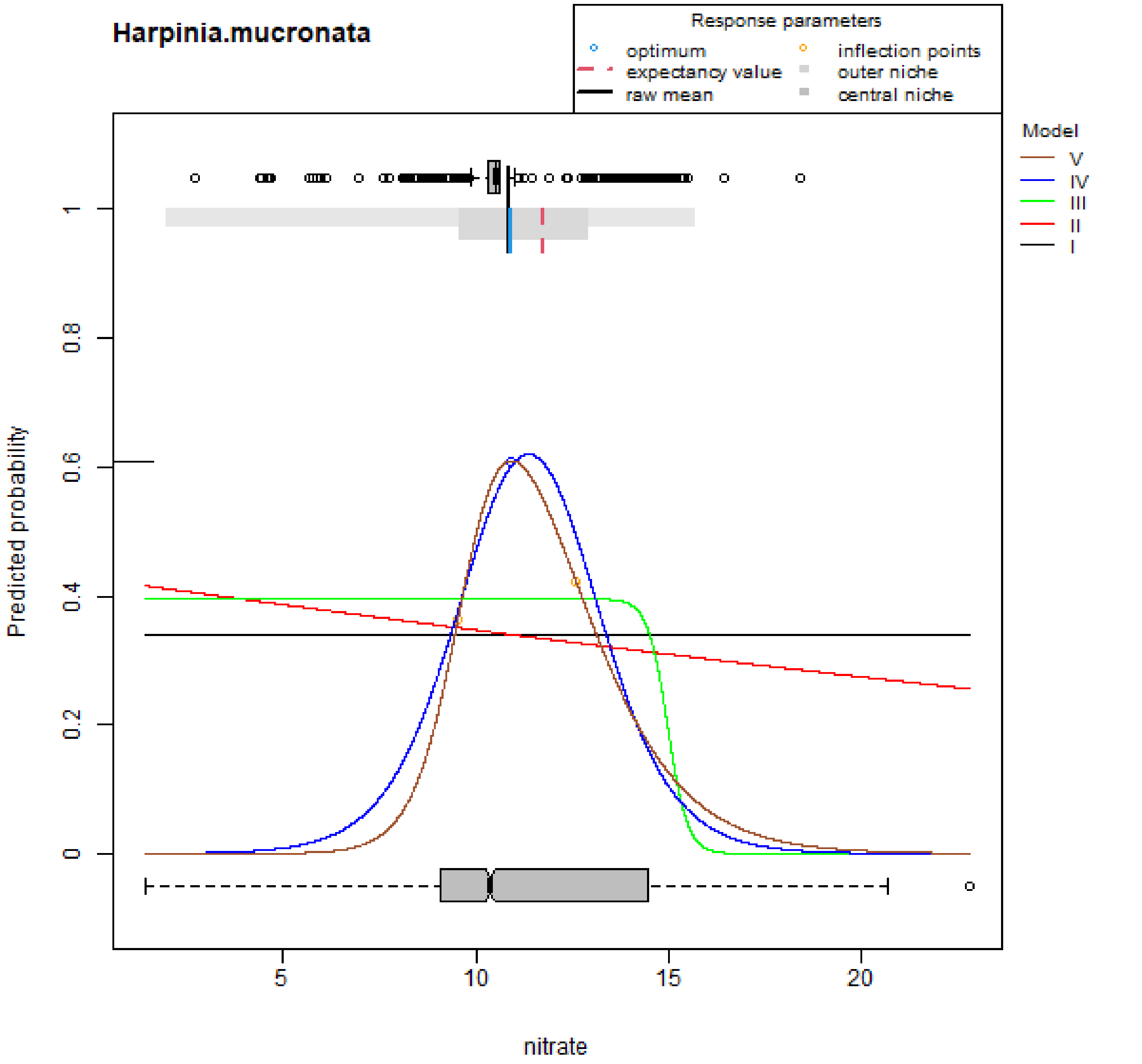

Harpinia.mucronata
Response parameters
optimum
expectancy value
raw mean
inflection points
outer niche
central niche
Model
V
IV
III
II
I
Predicted probability
0
0.2
0.4
0.6
0.8
1
5
10
15
20
nitrate

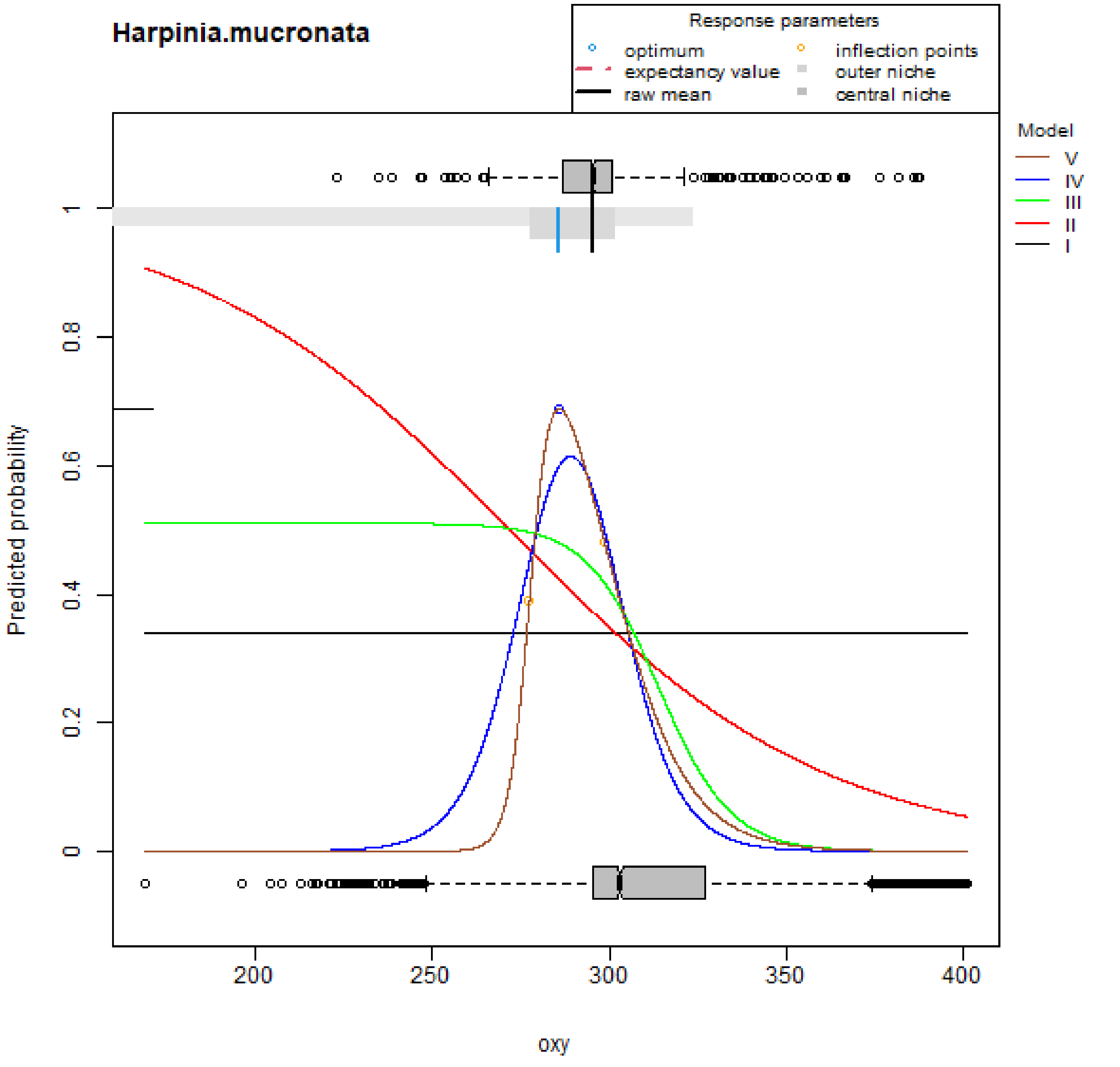

Harpinia.mucronata
Response parameters
optimum
expectancy value
raw mean
inflection points
outer niche
central niche
Model
V
IV
III
II
I
Predicted probability
0
0.2
0.4
0.6
0.8
1
200
250
300
350
400
oxy

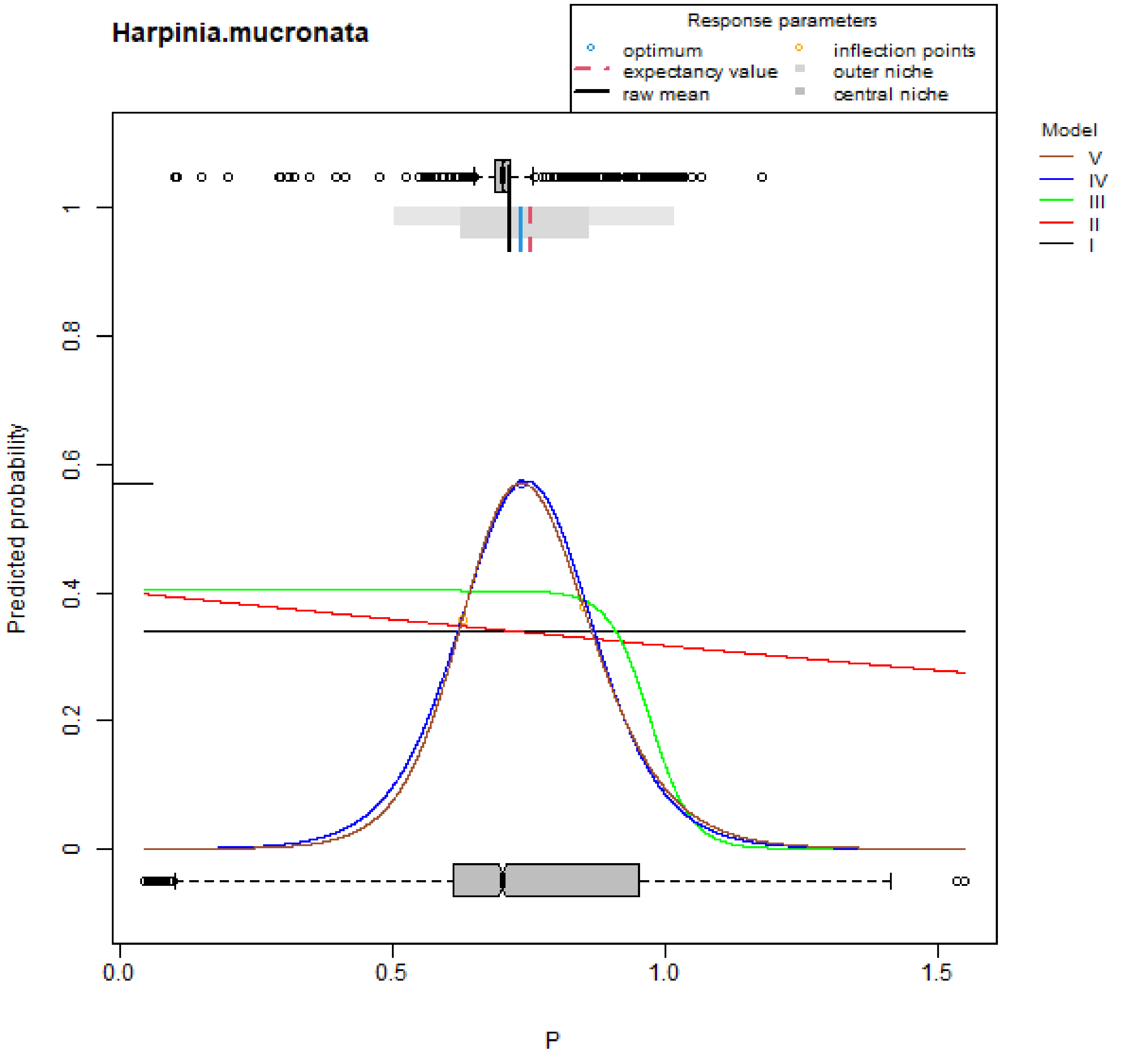

Harpinia.mucronata
Response parameters
optimum
expectancy value
raw mean
inflection points
outer niche
central niche
Model
V
IV
III
II
I
Predicted probability
0
0.2
0.4
0.6
0.8
1
0.0
0.5
1.0
1.5
P

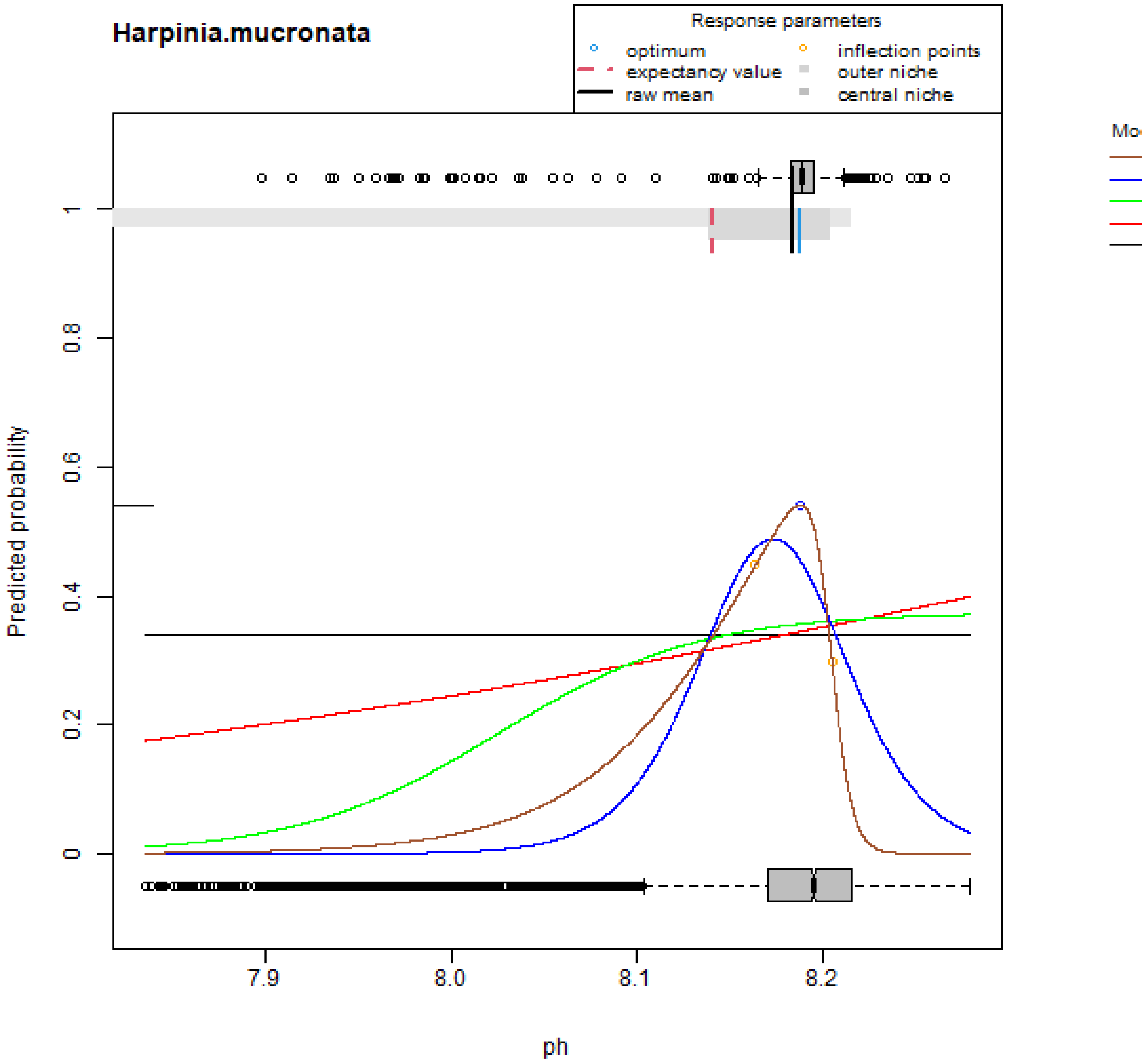

Harpinia.mucronata
Response parameters
optimum
expectancy value
raw mean
inflection points
outer niche
central niche
Mo
Predicted probability
0
0.2
0.4
0.6
0.8
1
7.9
8.0
8.1
8.2
ph

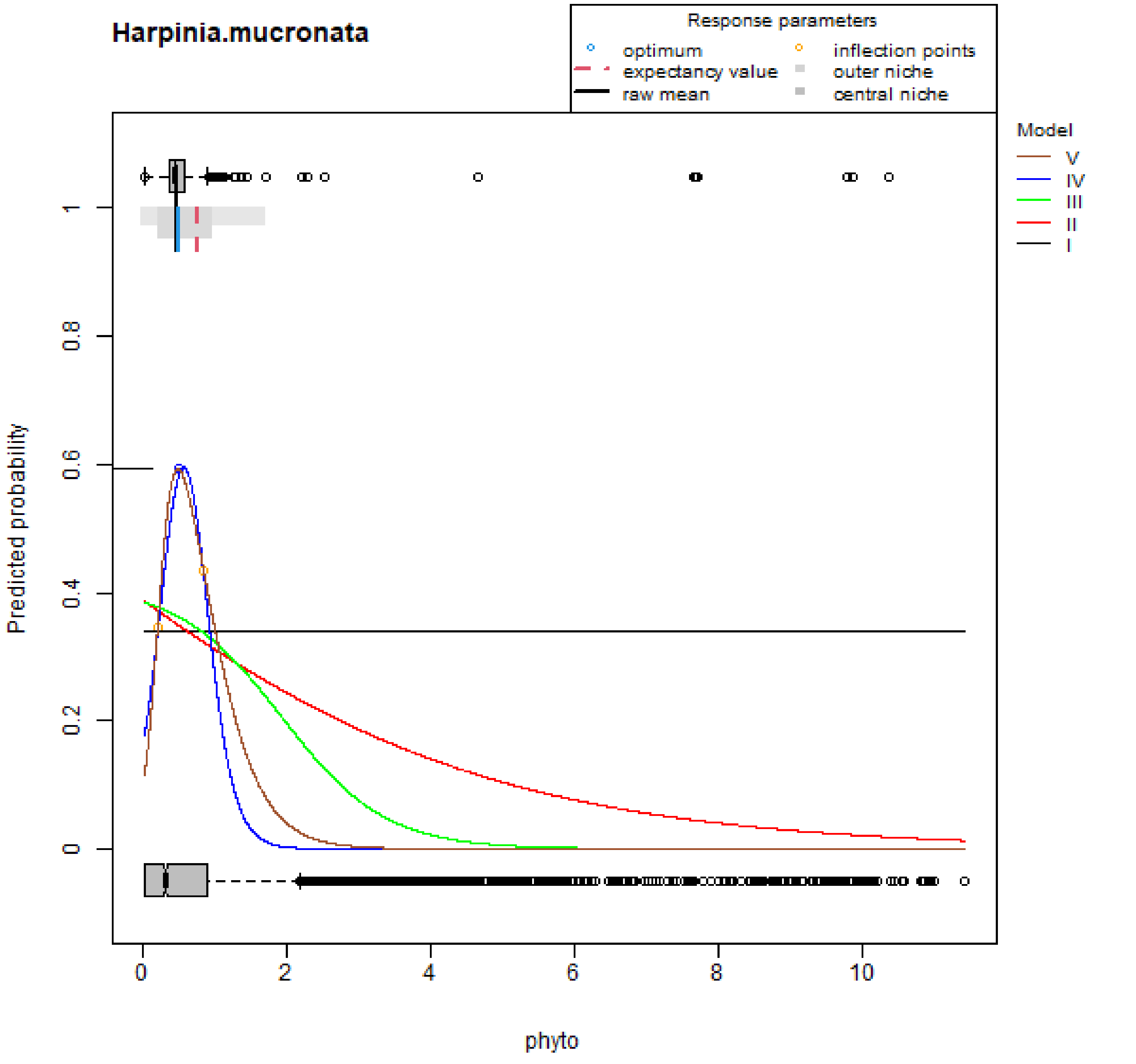

Harpinia.mucronata
Response parameters
optimum
expectancy value
raw mean
inflection points
outer niche
central niche
Model
V
IV
III
II
I
Predicted probability
0
0.2
0.4
0.6
0.8
1
0
2
4
6
8
10
phyto

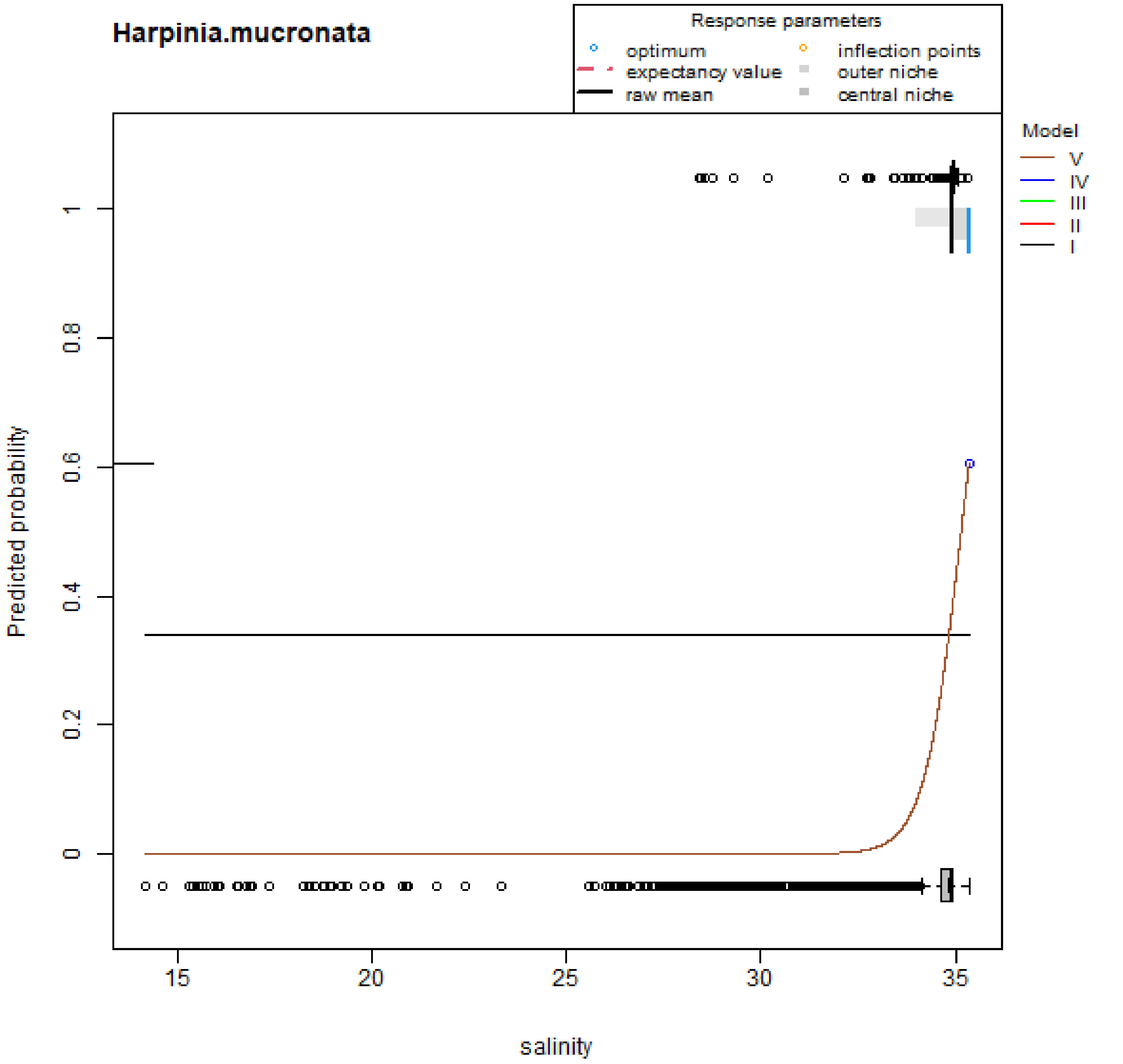

Harpinia.mucronata
Response parameters
optimum
inflection points
expectancy value
outer niche
raw mean
central niche
Model
V
IV
III
II
I
Predicted probability
0
0.2
0.4
0.6
0.8
1
15
20
25
30
35
salinity

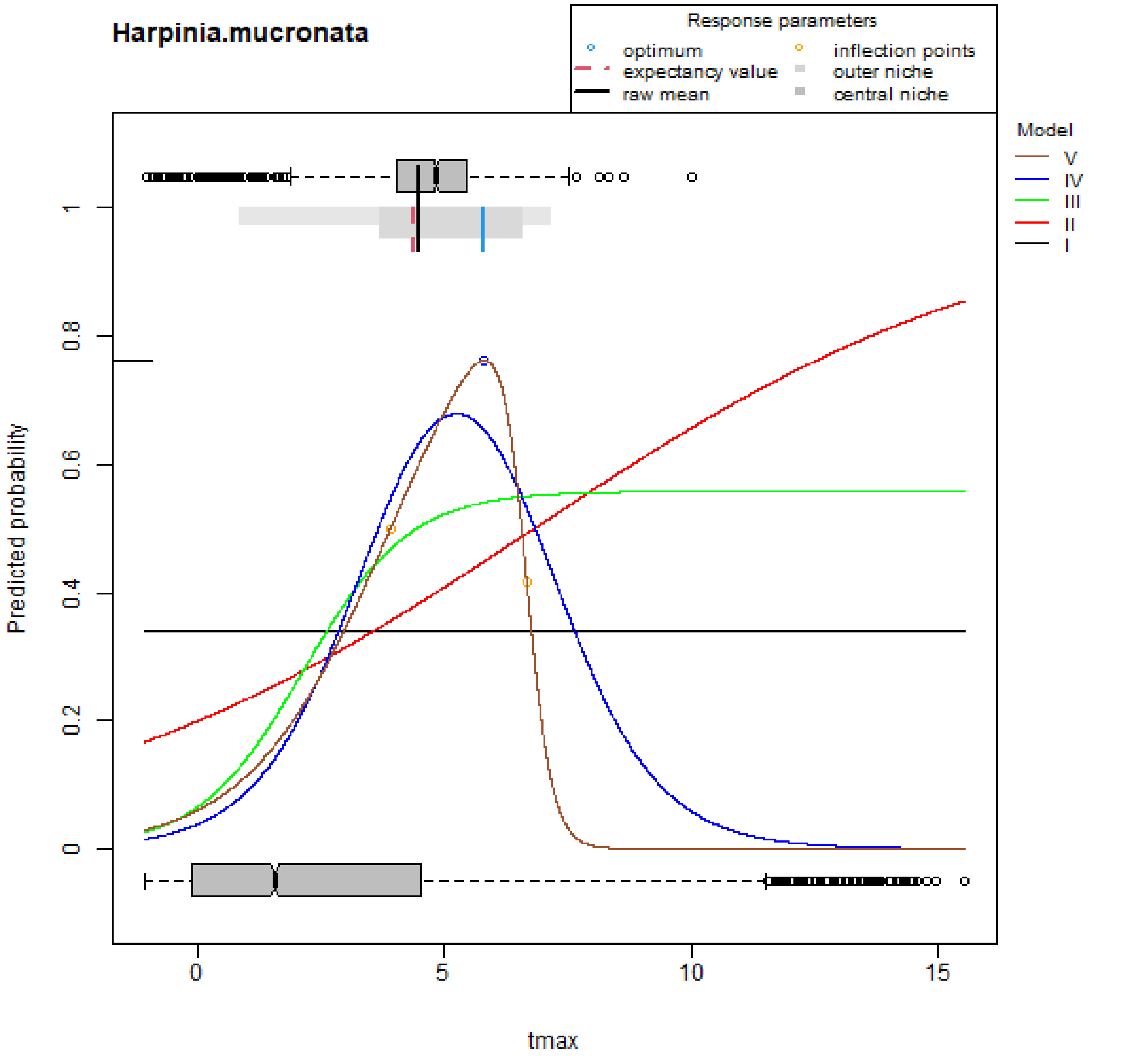
Harpinia.mucronata
Response parameters
optimum
expectancy value
raw mean
inflection points
outer niche
central niche
Model
V
IV
III
II
I
Predicted probability
0
0.2
0.4
0.6
0.8
1
0
5
10
15
tmax

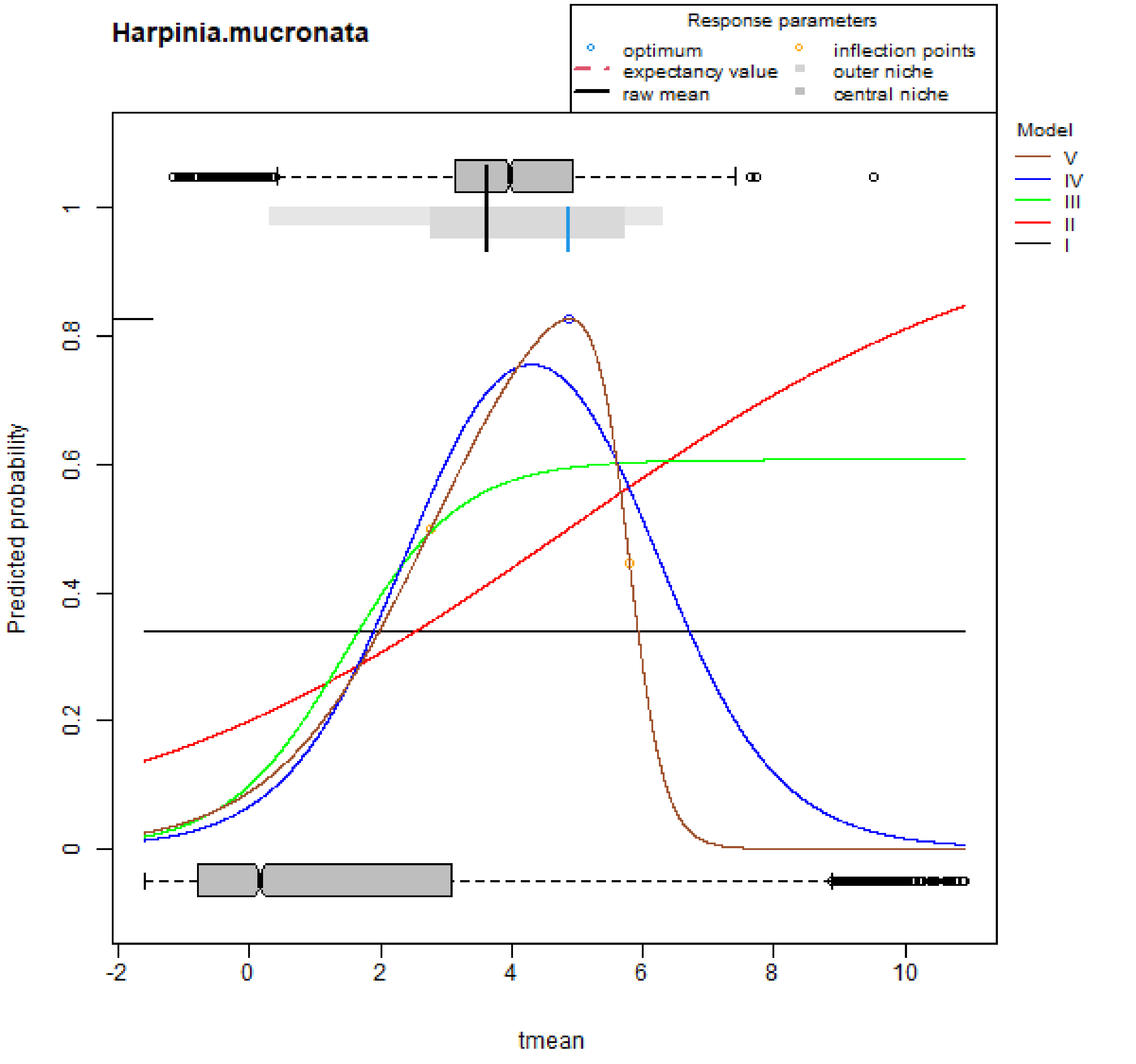

Harpinia.mucronata
Response parameters
optimum
expectancy value
raw mean
inflection points
outer niche
central niche
Model
V
IV
III
II
I
Predicted probability
0
0.2
0.4
0.6
0.8
1
-2
0
2
4
6
8
10
tmean

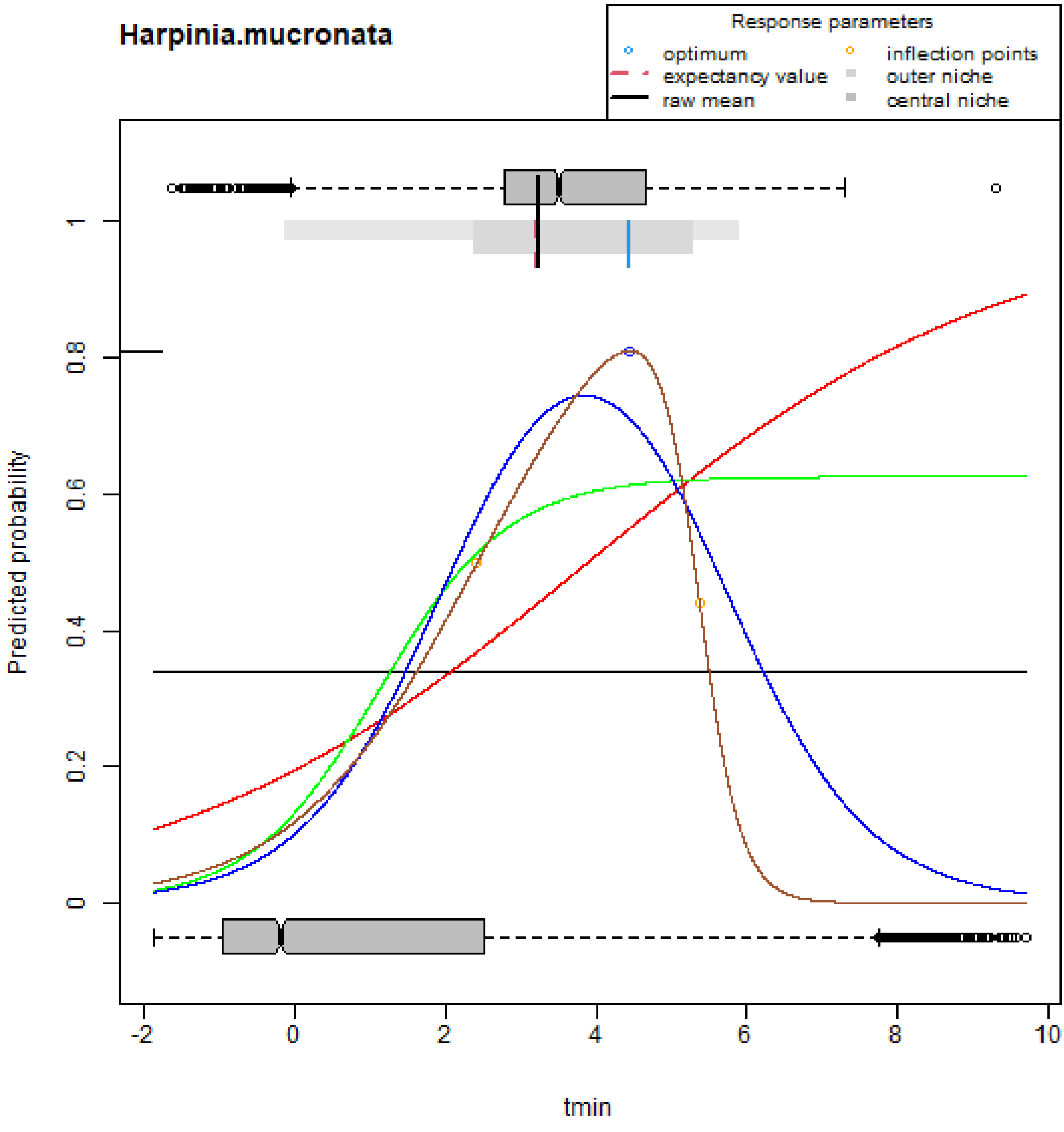

Harpinia.mucronata
Response parameters
optimum
expectancy value
raw mean
inflection points
outer niche
central niche
Model
V
IV
III
II
I
Predicted probability
0
0.2
0.4
0.6
0.8
1
-2
0
2
4
6
8
10
tmin

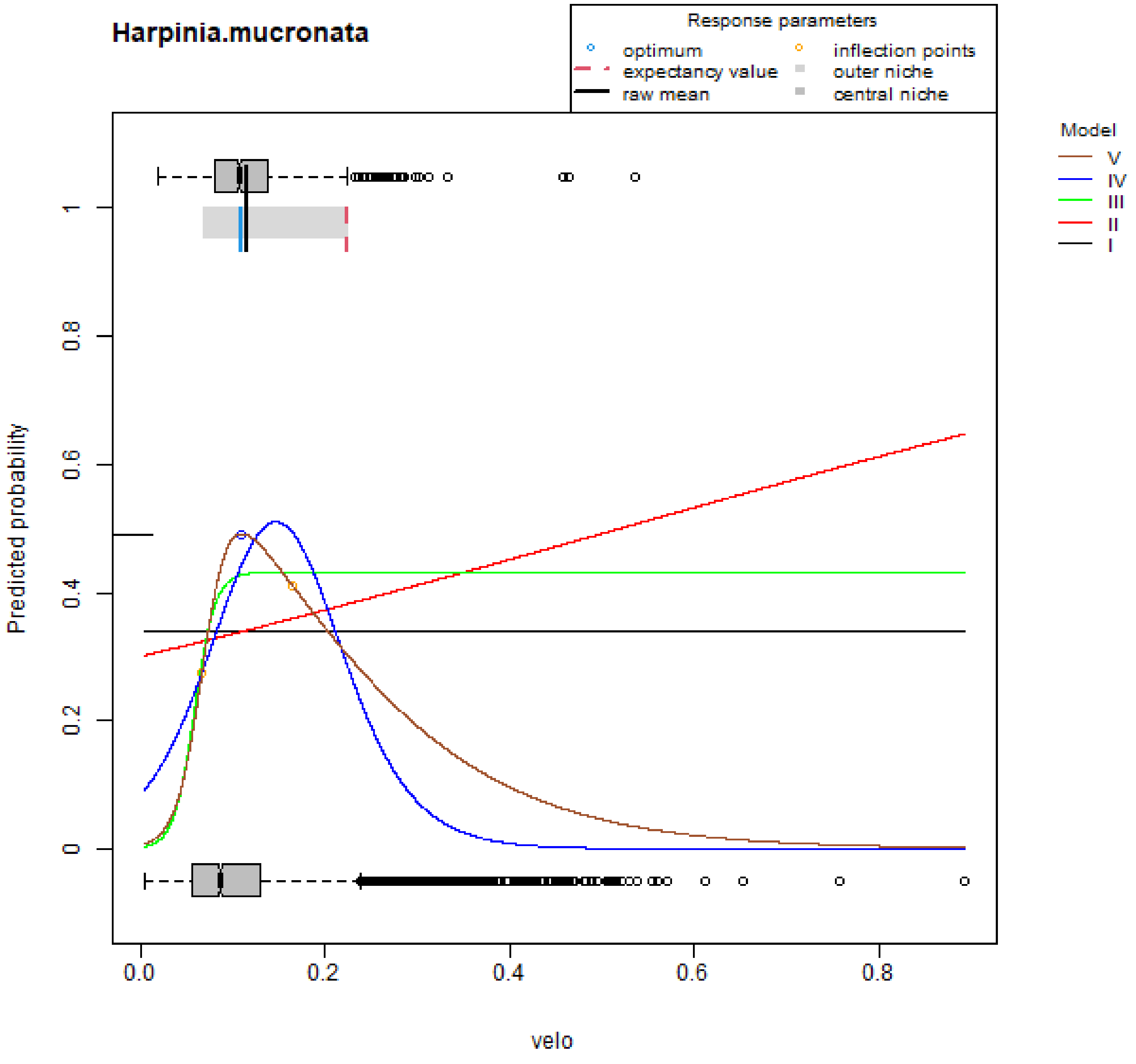
Harpinia.mucronata
Response parameters
optimum
expectancy value
raw mean
inflection points
outer niche
central niche
Model
V
IV
III
II
I
Predicted probability
0
0.2
0.4
0.6
0.8
1
0.0
0.2
0.4
0.6
0.8
velo

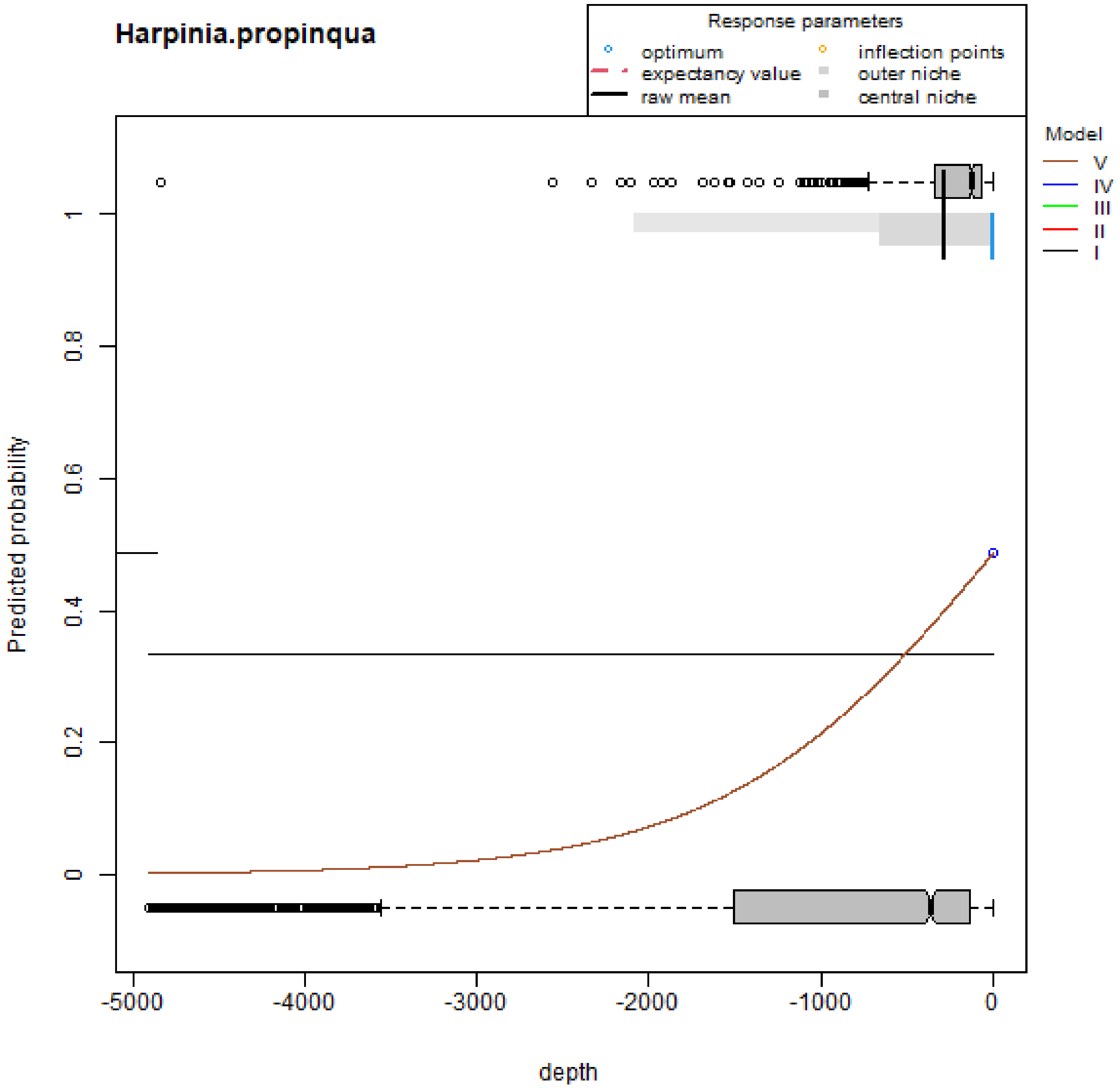

Harpinia.propinqua
Response parameters
optimum
expectancy value
raw mean
inflection points
outer niche
central niche
Model
V
IV
III
II
I
Predicted probability
0
0.2
0.4
0.6
0.8
1
-5000
-4000
-3000
-2000
-1000
0
depth

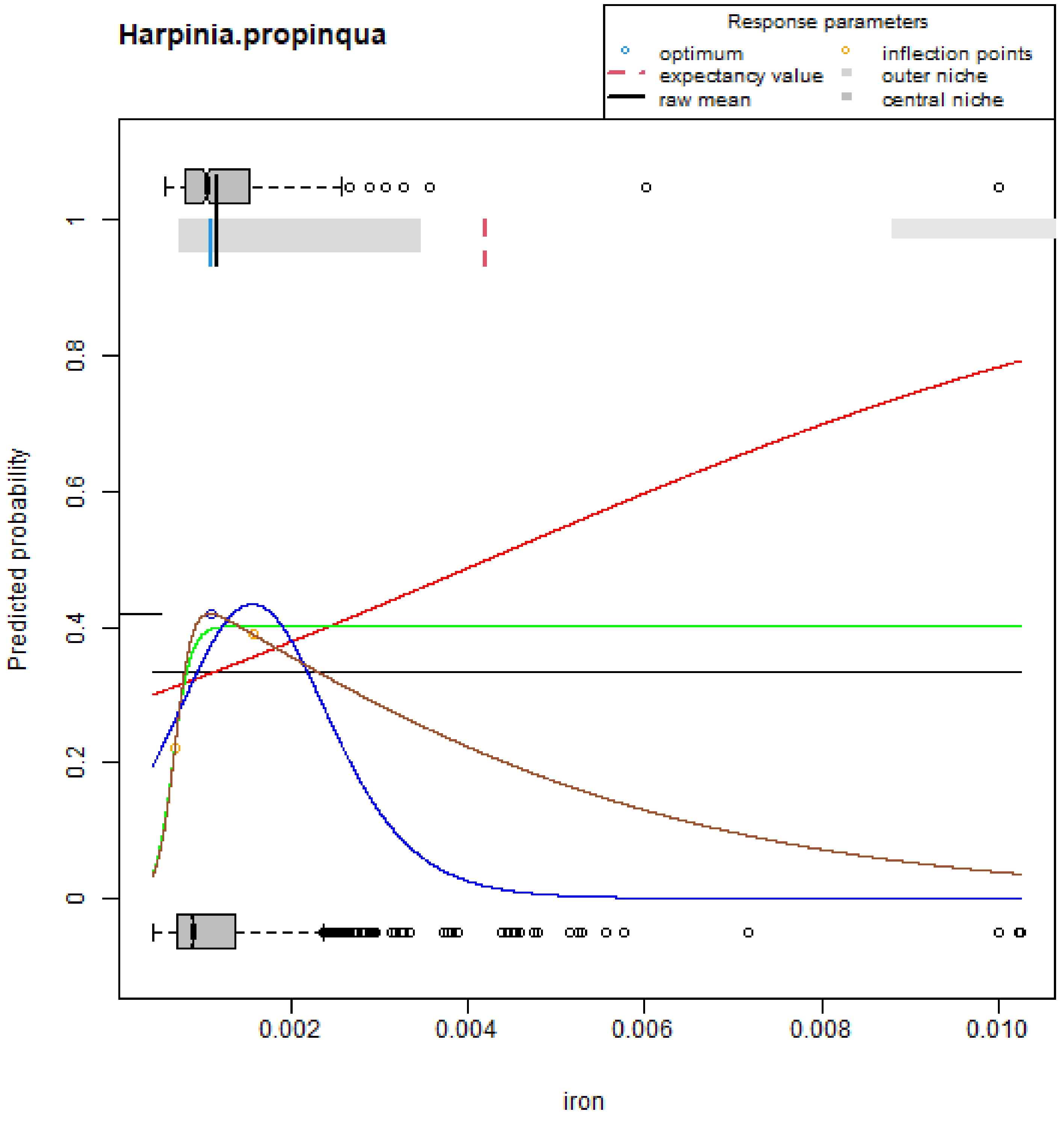

Harpinia.propinqua
Response parameters
optimum
expectancy value
raw mean
inflection points
outer niche
central niche
Predicted probability
0
0.2
0.4
0.6
0.8
1
0.002
0.004
0.006
0.008
0.010
iron

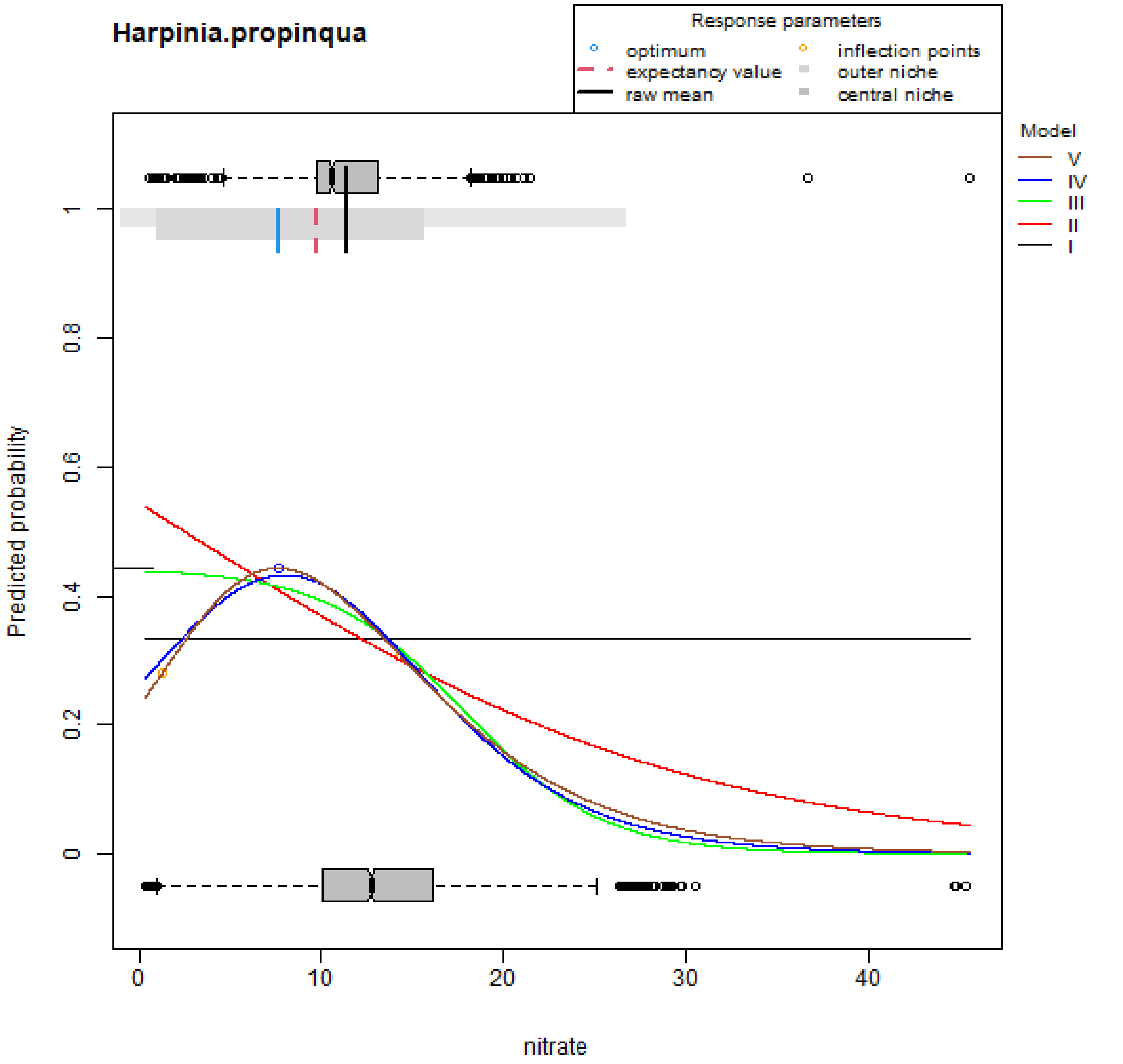

Harpinia.propinqua
Response parameters
optimum
expectancy value
raw mean
inflection points
outer niche
central niche
Model
V
IV
III
II
I
Predicted probability
0
0.2
0.4
0.6
0.8
1
0
10
20
30
40
nitrate

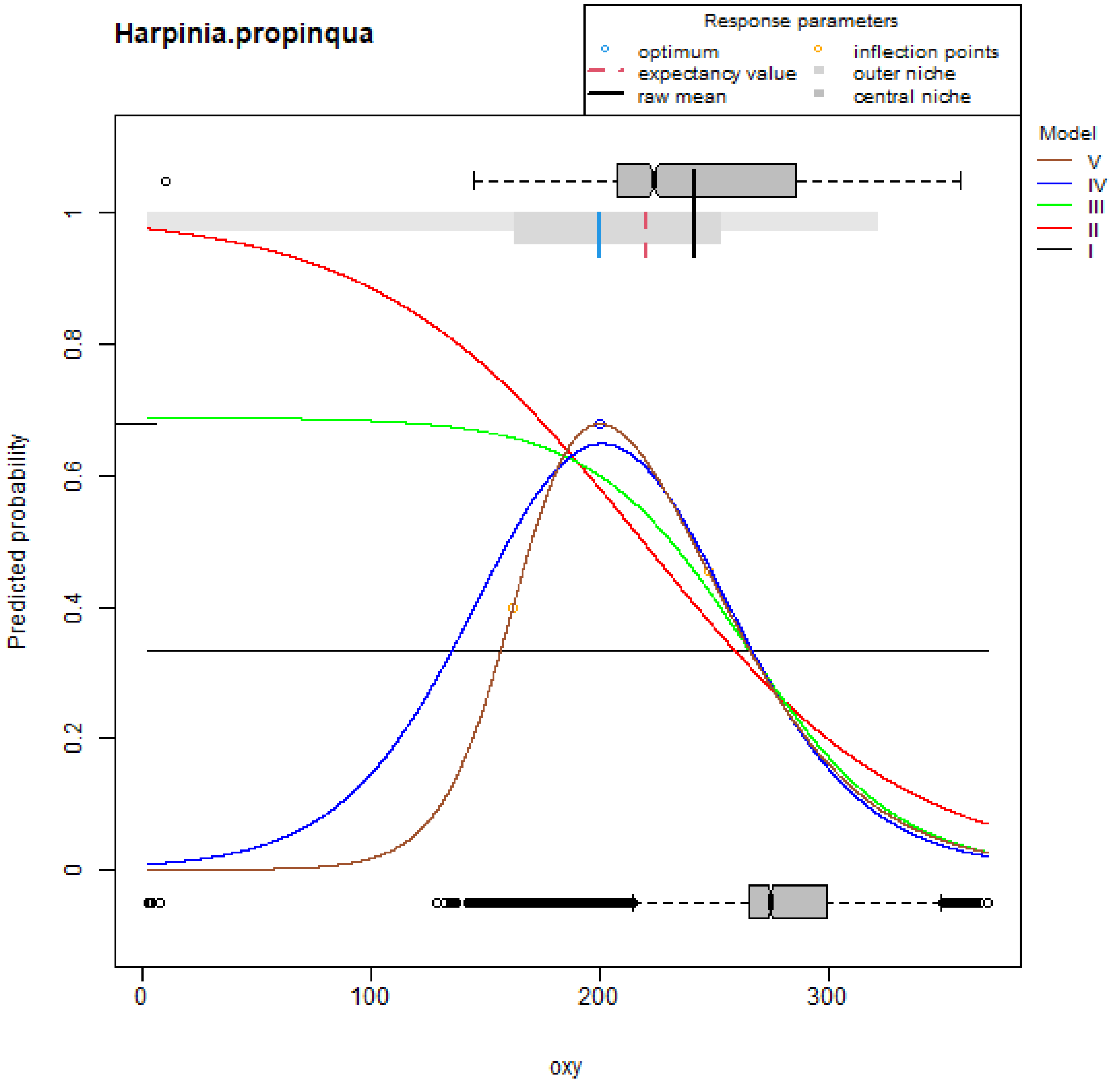
Harpinia.propinqua
Response parameters
optimum
expectancy value
raw mean
inflection points
outer niche
central niche
Model
V
IV
III
II
I
Predicted probability
0
0.2
0.4
0.6
0.8
1
0
100
200
300
oxy

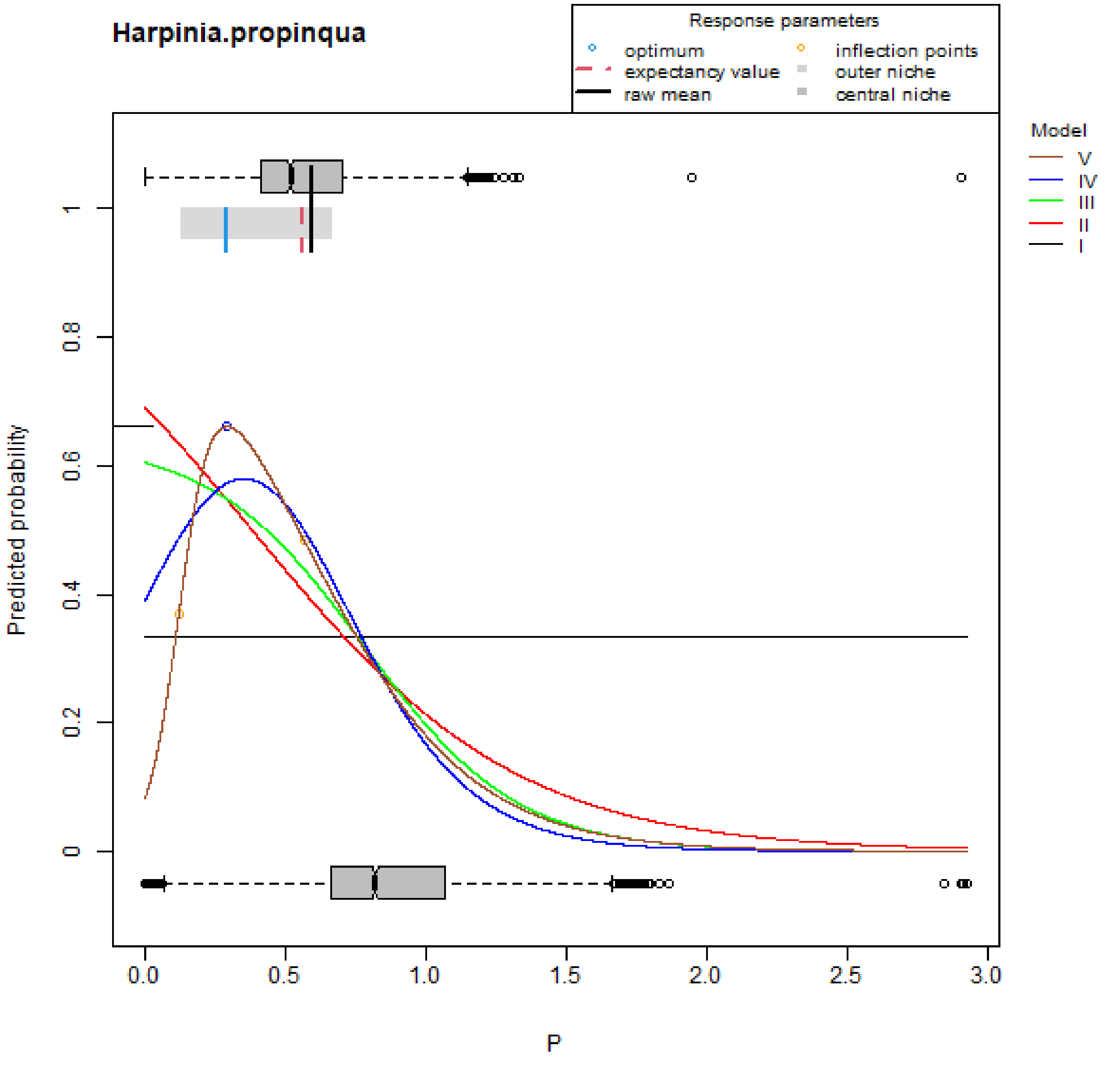
Harpinia.propinqua
Response parameters
optimum
expectancy value
raw mean
inflection points
outer niche
central niche
Model
V
IV
III
II
I
Predicted probability
0
0.2
0.4
0.6
0.8
1
0.0
0.5
1.0
1.5
2.0
2.5
3.0
P

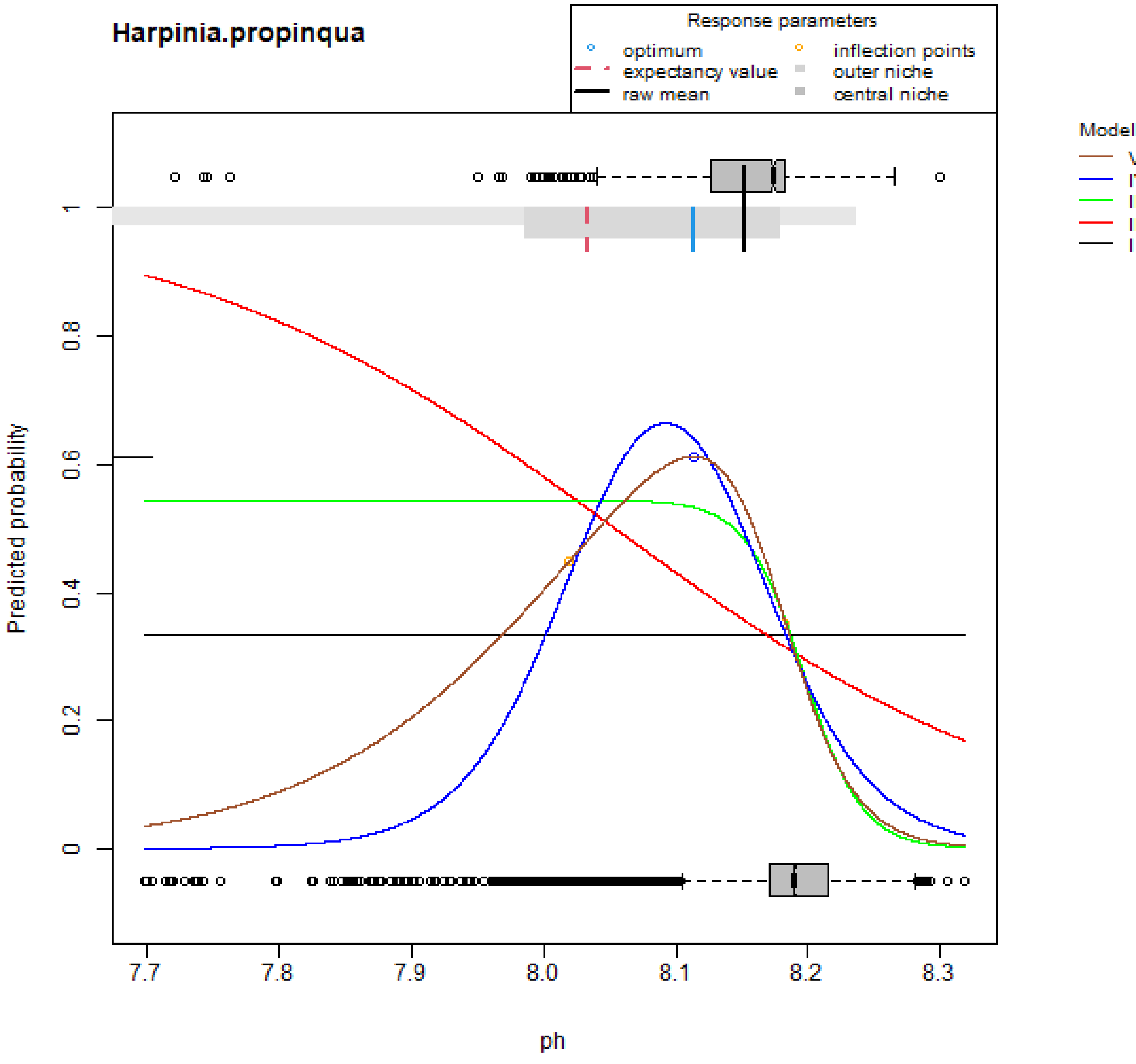

Harpinia.propinqua
Response parameters
optimum
expectancy value
raw mean
inflection points
outer niche
central niche
Model
Predicted probability
0
0.2
0.4
0.6
0.8
1
7.7
7.8
7.9
8.0
8.1
8.2
8.3
ph

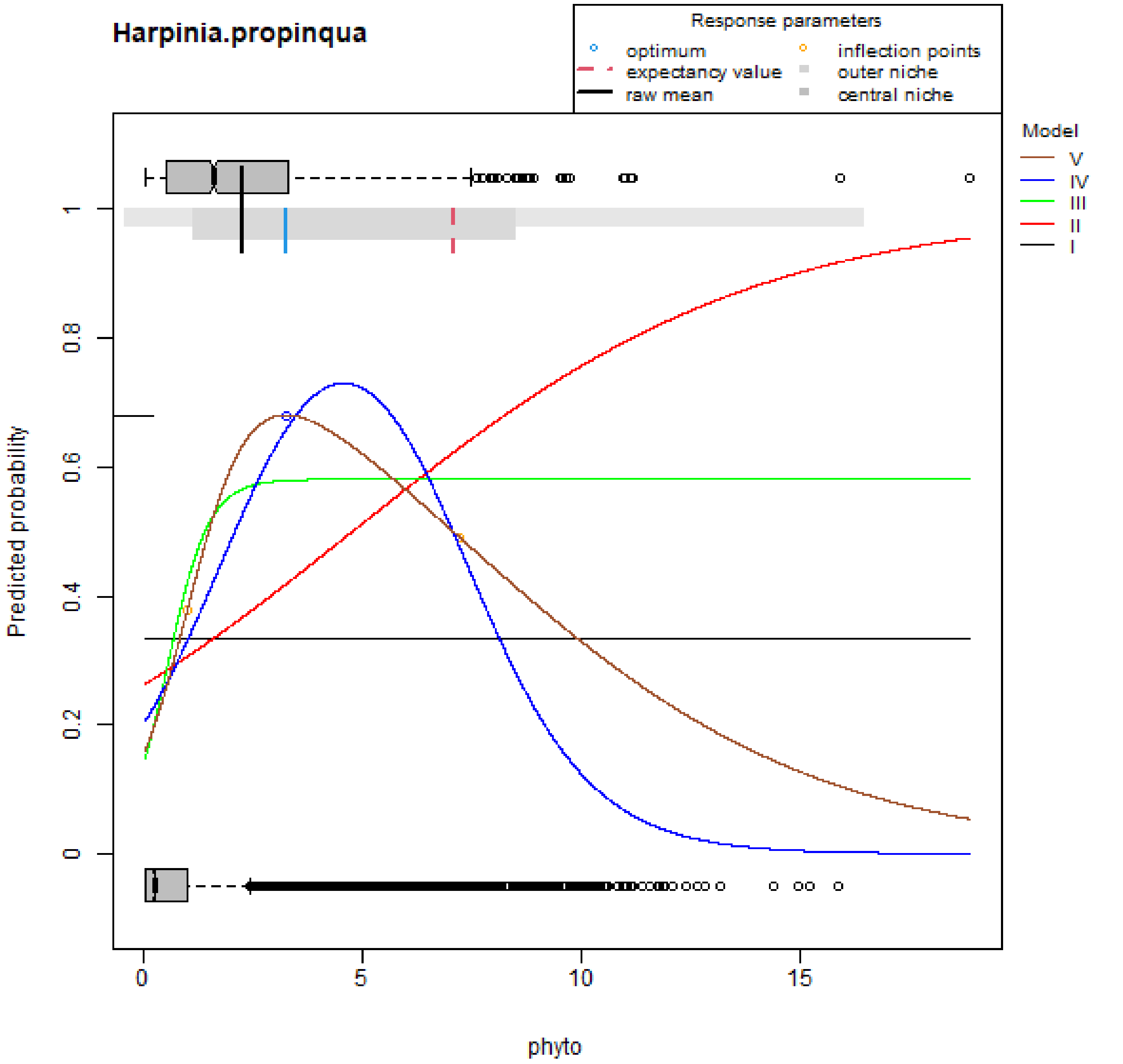

Harpinia.propinqua
Response parameters
optimum
expectancy value
raw mean
inflection points
outer niche
central niche
Model
V
IV
III
II
I
Predicted probability
0
0.2
0.4
0.6
0.8
1
0
5
10
15
phyto

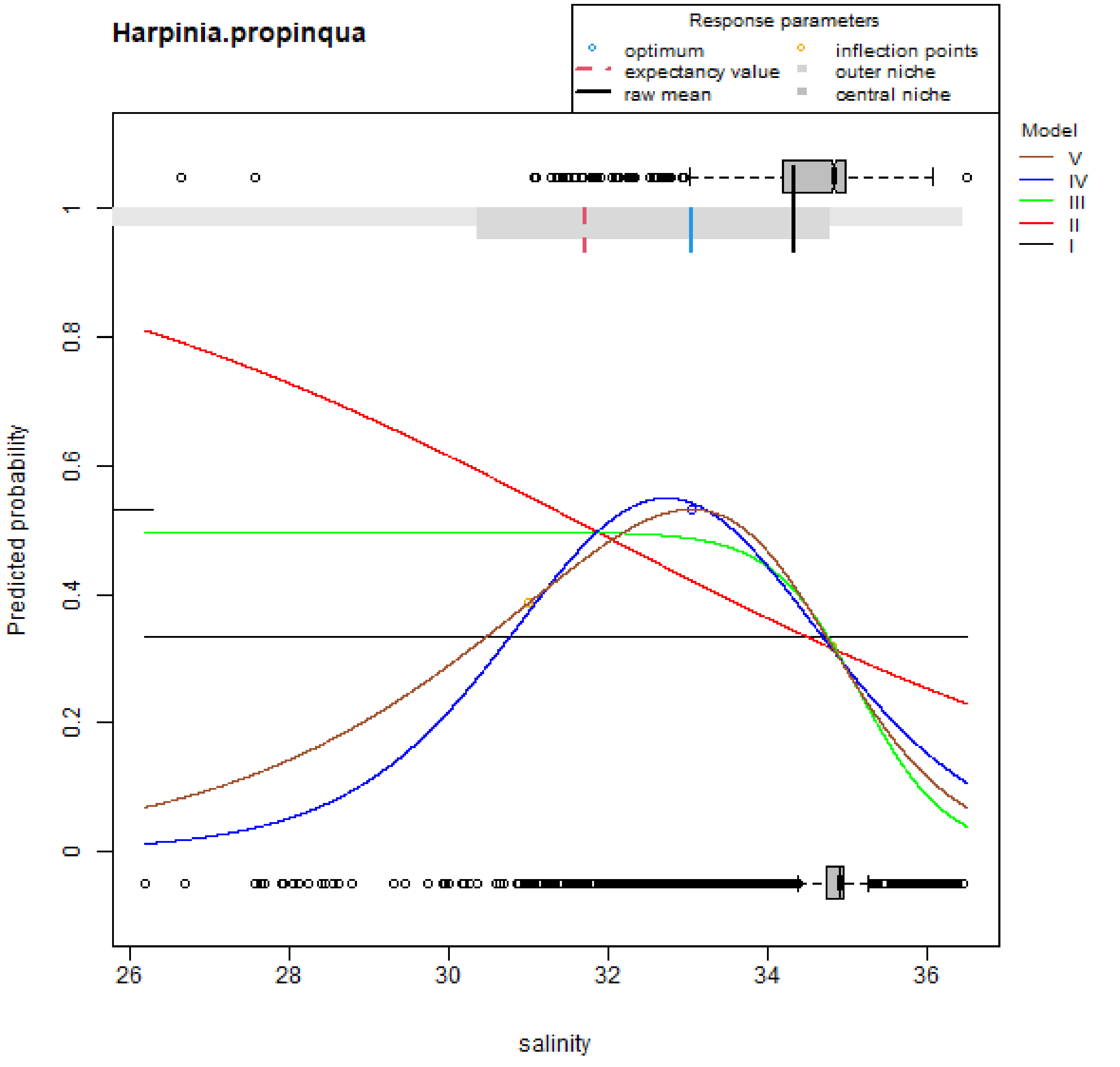

Harpinia.propinqua
Response parameters
optimum
expectancy value
raw mean
inflection points
outer niche
central niche
Model
V
IV
III
II
I
Predicted probability
0
0.2
0.4
0.6
0.8
1
26
28
30
32
34
36
salinity

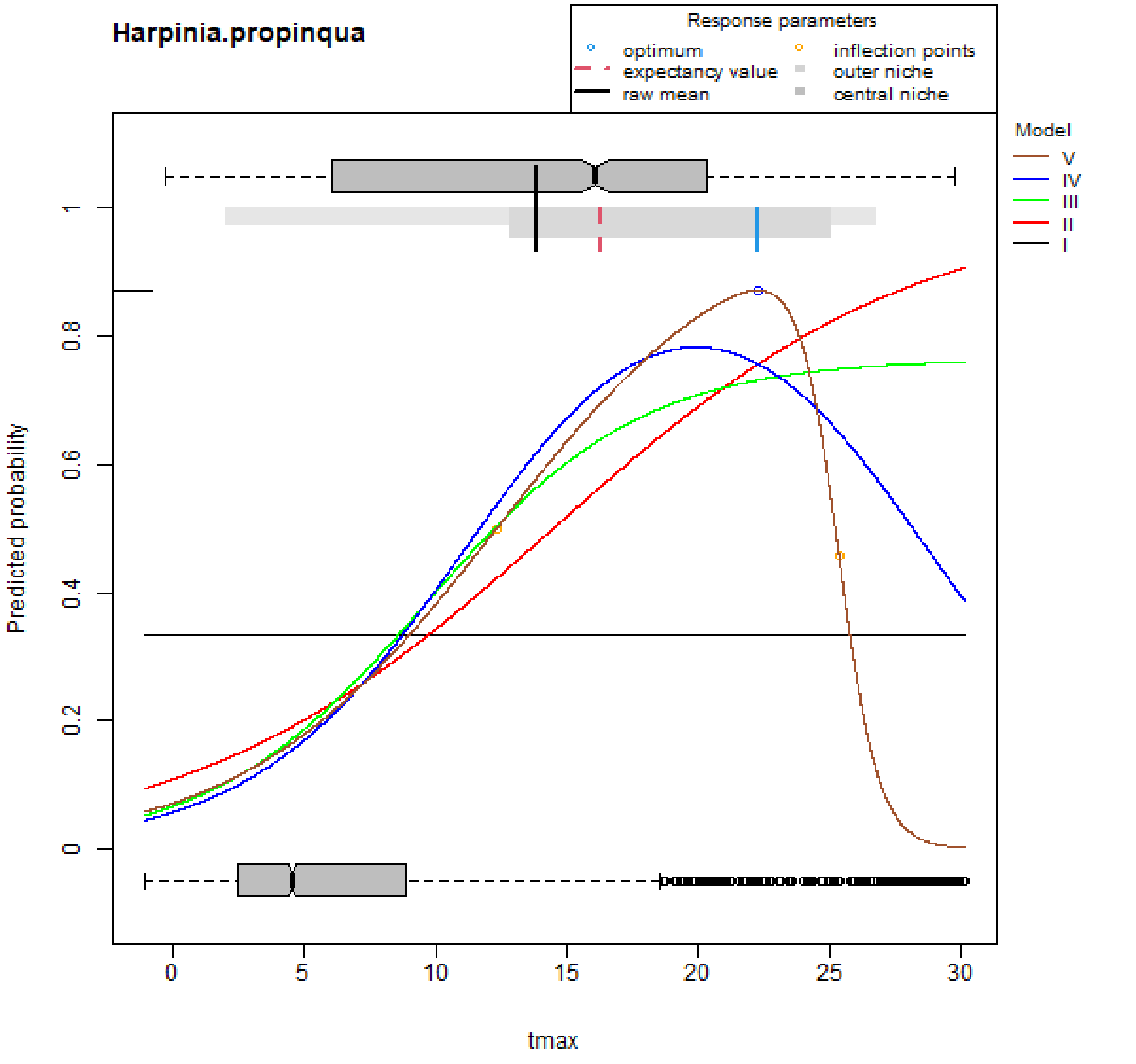
Harpinia.propinqua
Response parameters
optimum
expectancy value
raw mean
inflection points
outer niche
central niche
Model
V
IV
III
II
I
Predicted probability
0
0.2
0.4
0.6
0.8
1
0
5
10
15
20
25
30
tmax

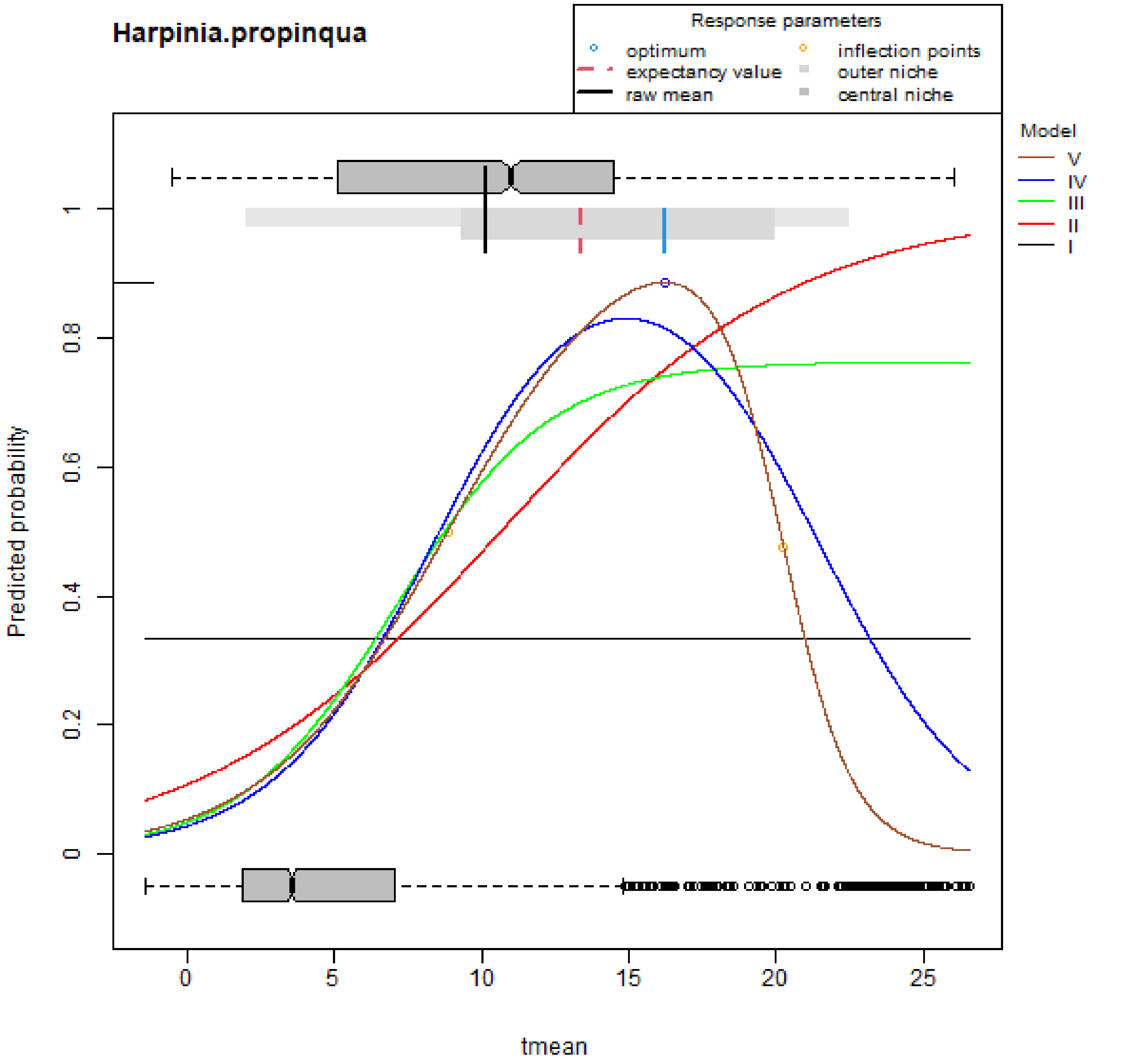

Harpinia.propinqua
Response parameters
optimum
inflection points
expectancy value
outer niche
raw mean
central niche
Model
V
IV
III
II
I
Predicted probability
0
0.2
0.4
0.6
0.8
1
0
5
10
15
20
25
tmean

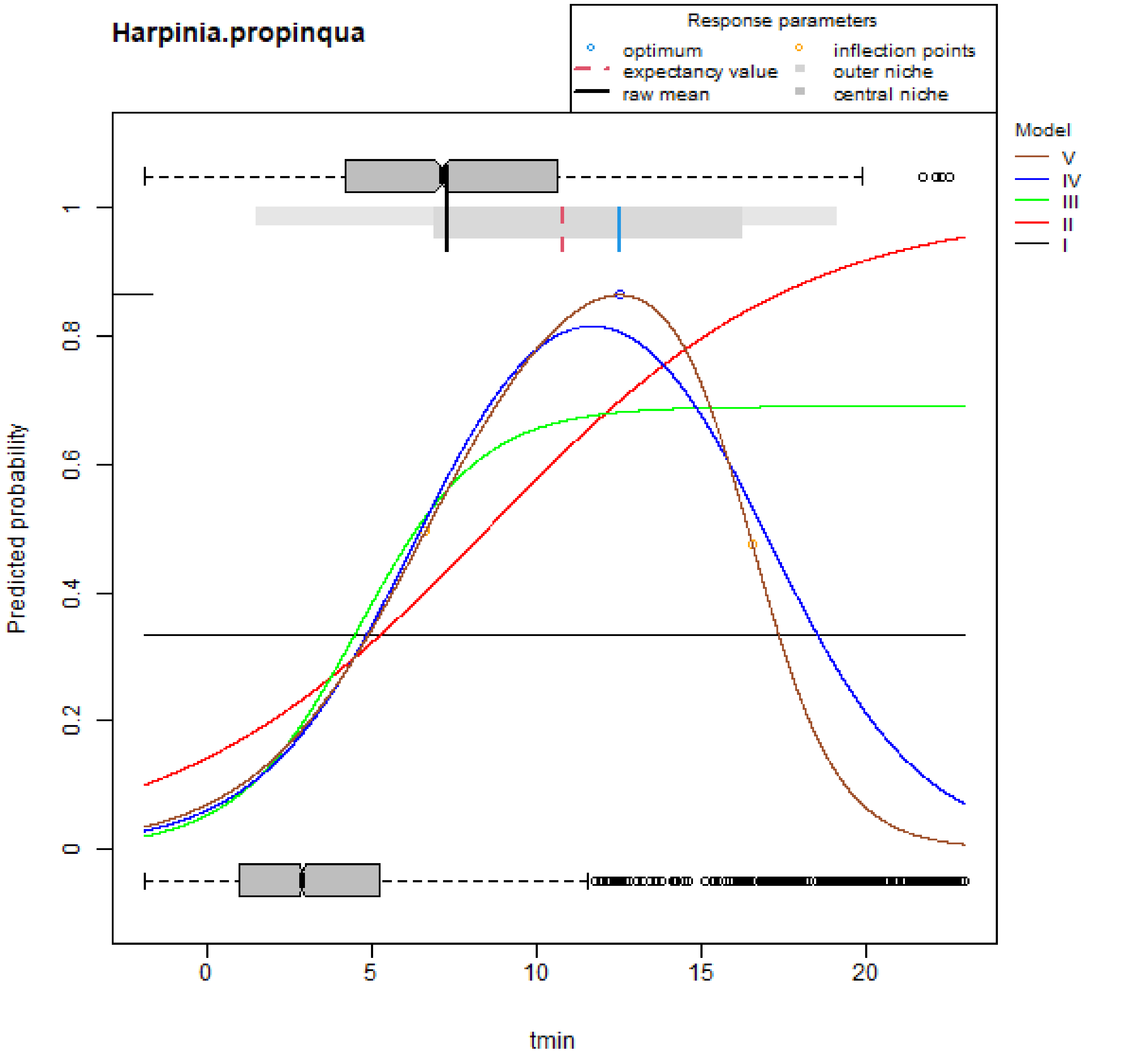

Harpinia.propinqua
Response parameters
optimum
expectancy value
raw mean
inflection points
outer niche
central niche
Model
V
IV
III
II
I
Predicted probability
0
0.2
0.4
0.6
0.8
1
0
5
10
15
20
tmin

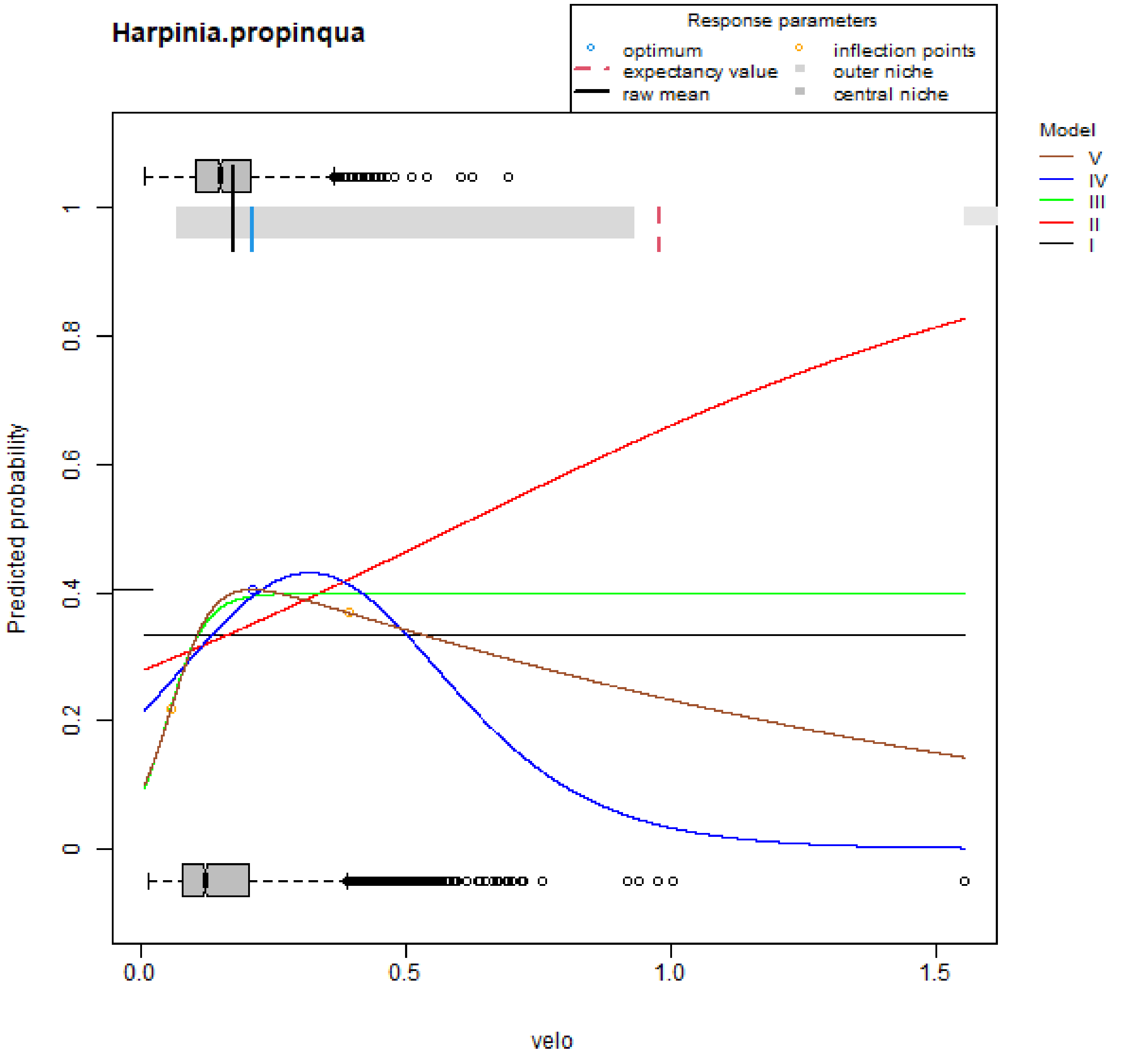

Harpinia.propinqua
Response parameters
optimum
expectancy value
raw mean
inflection points
outer niche
central niche
Model
V
IV
III
II
I
Predicted probability
0
0.2
0.4
0.6
0.8
1
0.0
0.5
1.0
1.5
velo

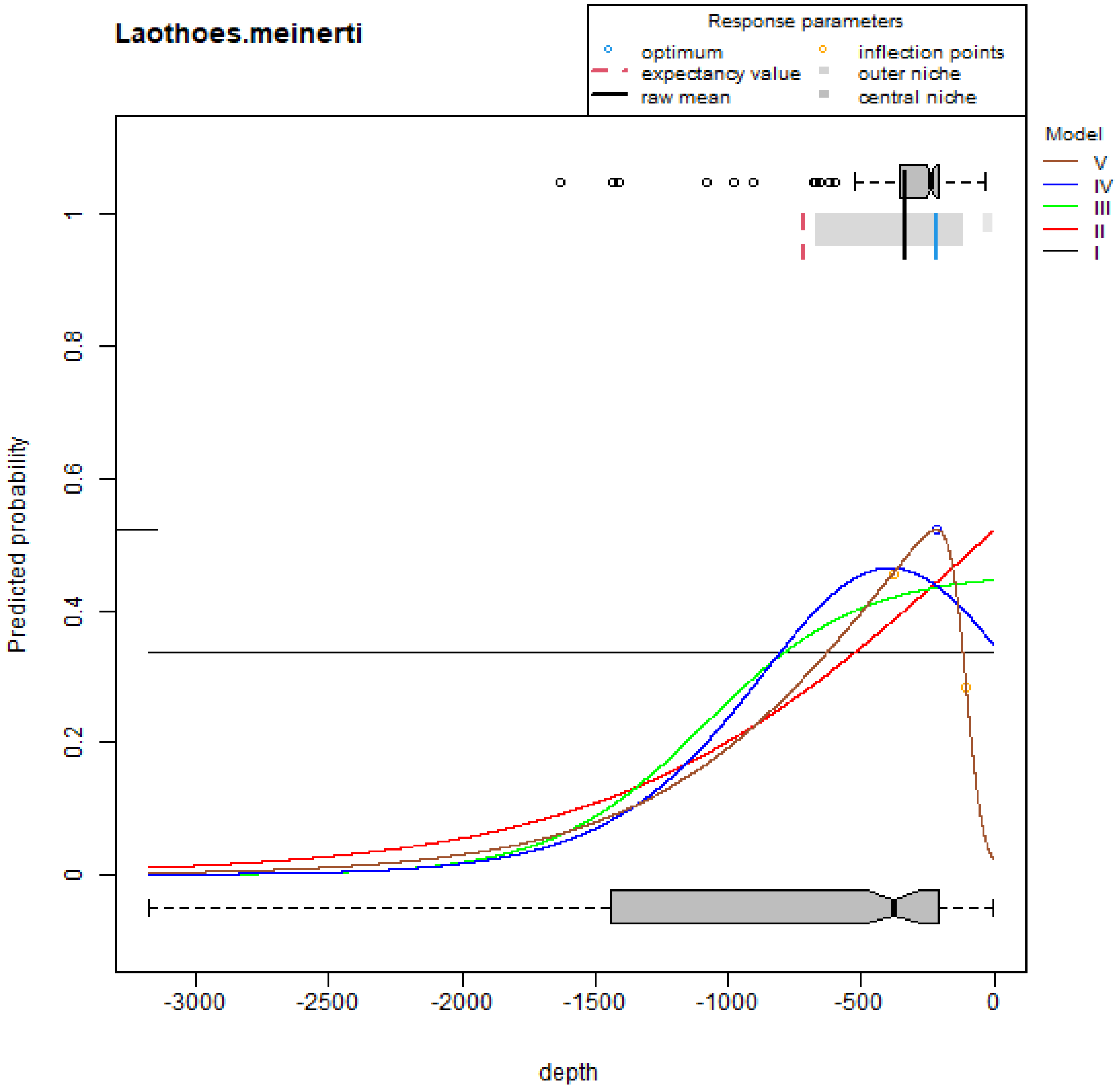

Laothoes.meinerti
Response parameters
optimum
expectancy value
raw mean
inflection points
outer niche
central niche
Model
V
IV
III
II
I
Predicted probability
0
0.2
0.4
0.6
0.8
1
-3000
-2500
-2000
-1500
-1000
-500
0
depth

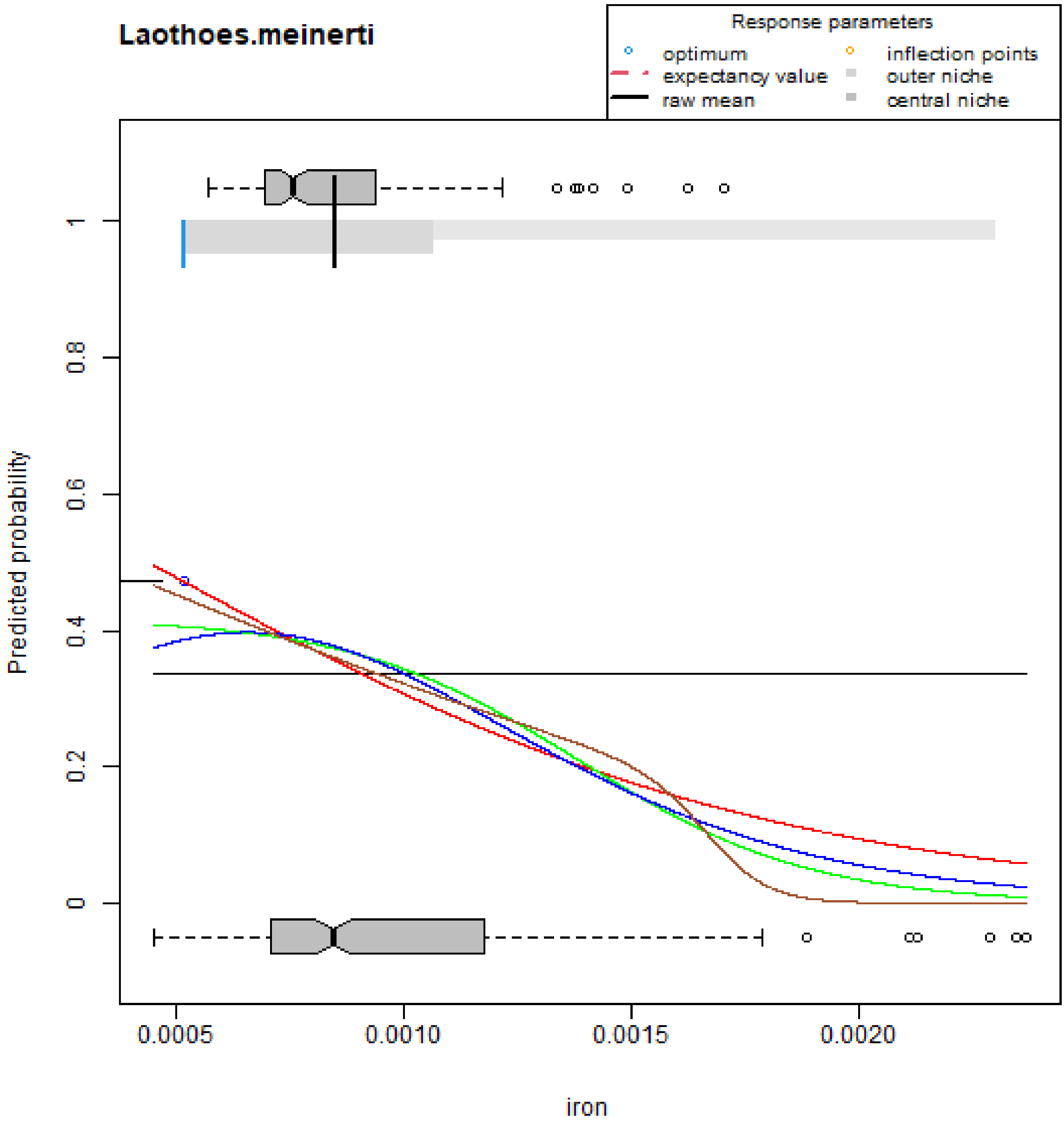
Laothoes.meinerti
Response parameters
optimum
expectancy value
raw mean
inflection points
outer niche
central niche
Predicted probability
0
0.2
0.4
0.6
0.8
1
0.0005
0.0010
0.0015
0.0020
iron

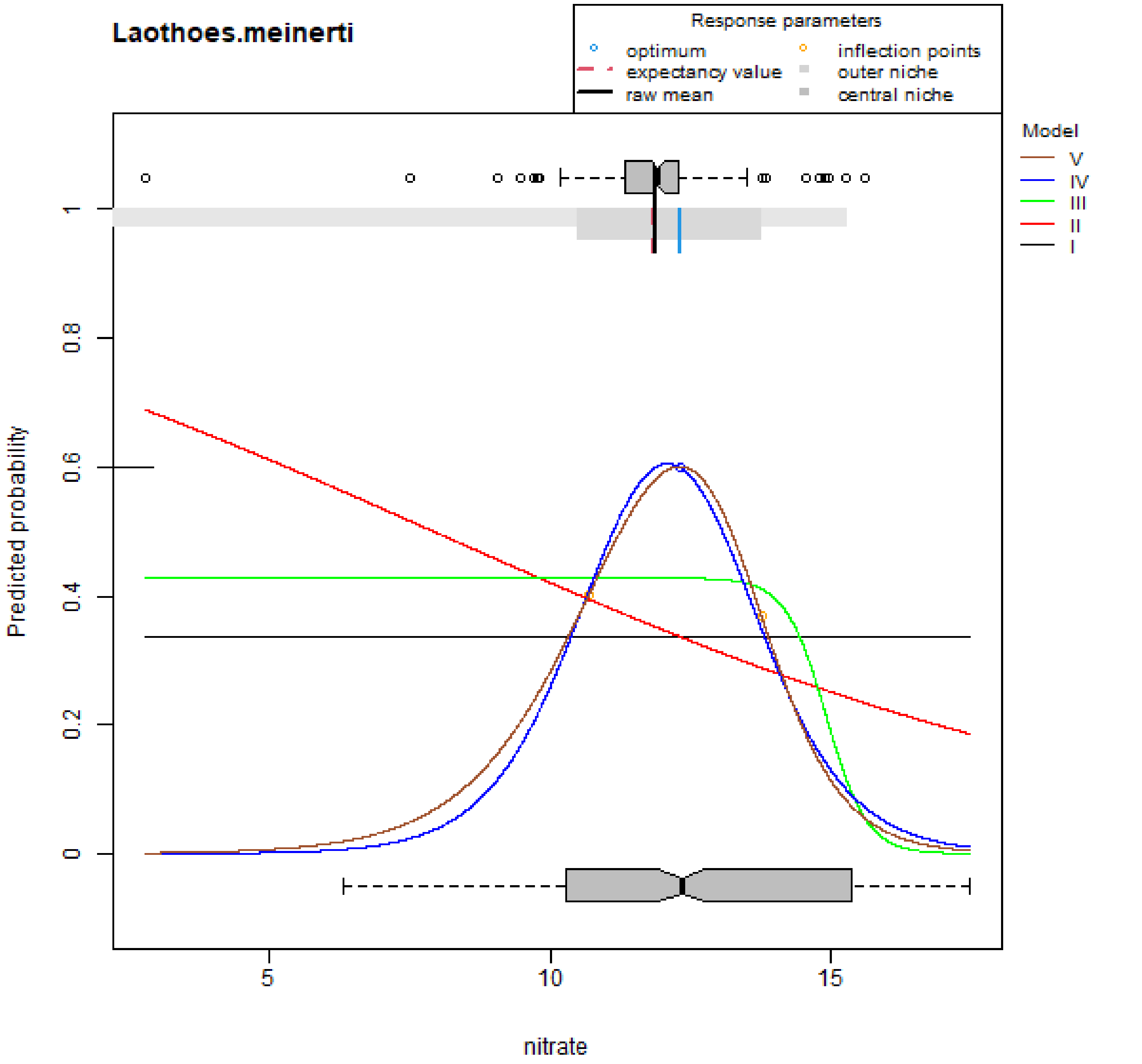
Laothoes.meinerti
Response parameters
optimum
expectancy value
raw mean
inflection points
outer niche
central niche
Model
V
IV
III
II
I
Predicted probability
0
0.2
0.4
0.6
0.8
1
5
10
15
nitrate

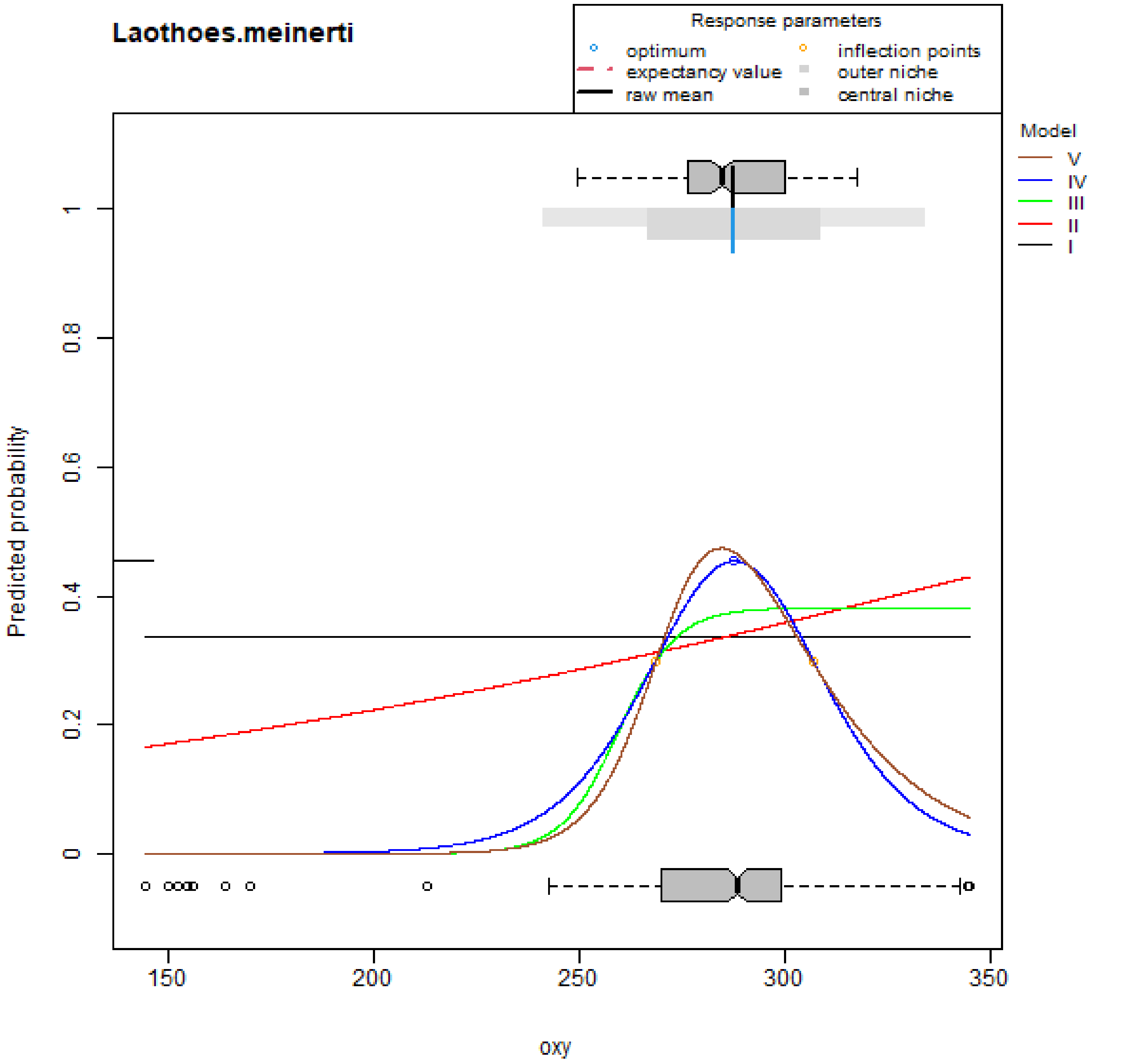

Laothoes.meinerti
Response parameters
optimum
expectancy value
raw mean
inflection points
outer niche
central niche
Model
V
IV
III
II
I
Predicted probability
0
0.2
0.4
0.6
0.8
1
150
200
250
300
350
oxy

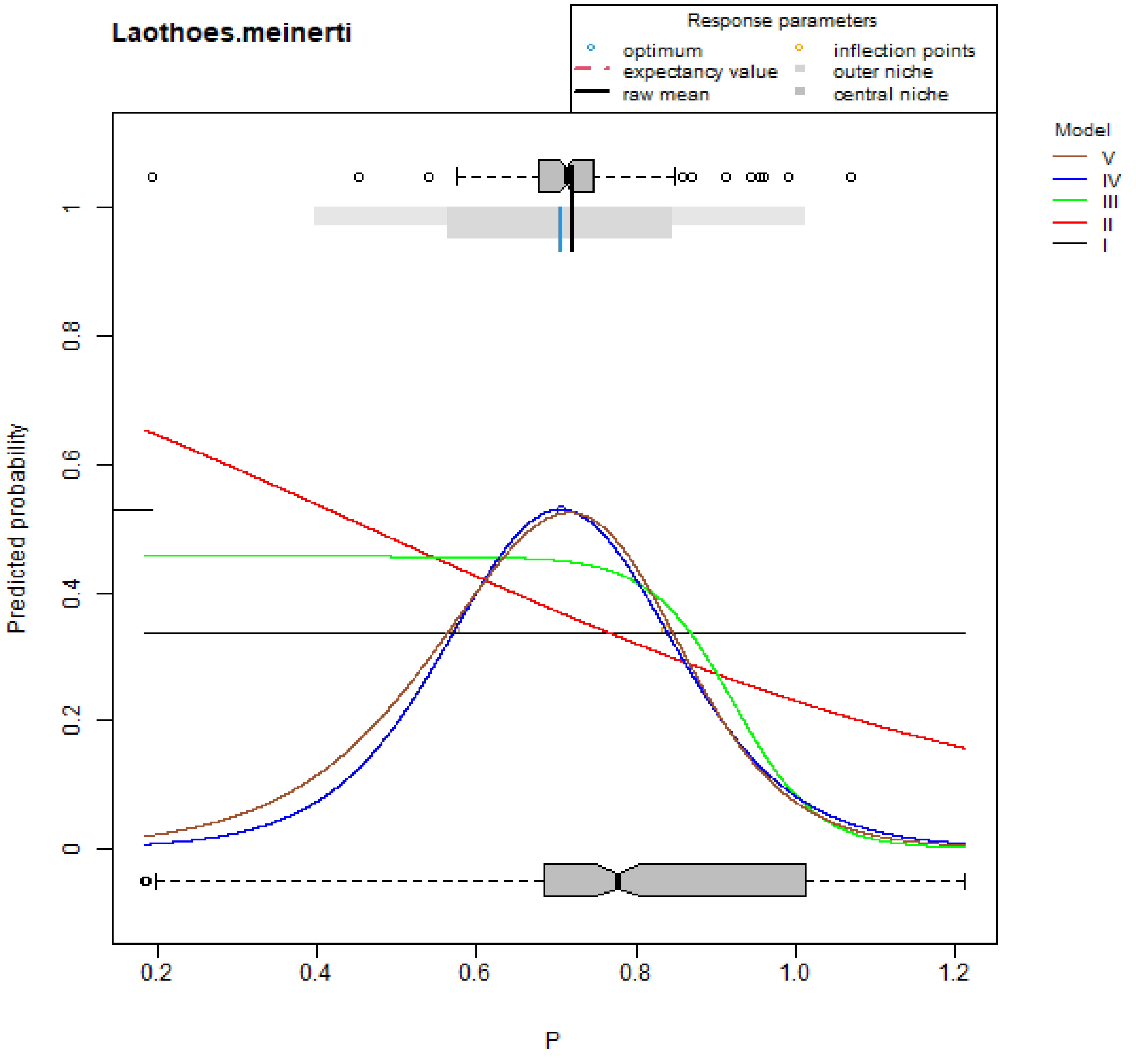

Laothoes.meinerti
Response parameters
optimum
expectancy value
raw mean
inflection points
outer niche
central niche
Model
V
IV
III
II
I
Predicted probability
0
0.2
0.4
0.6
0.8
1
0.2
0.4
0.6
0.8
1.0
1.2
P

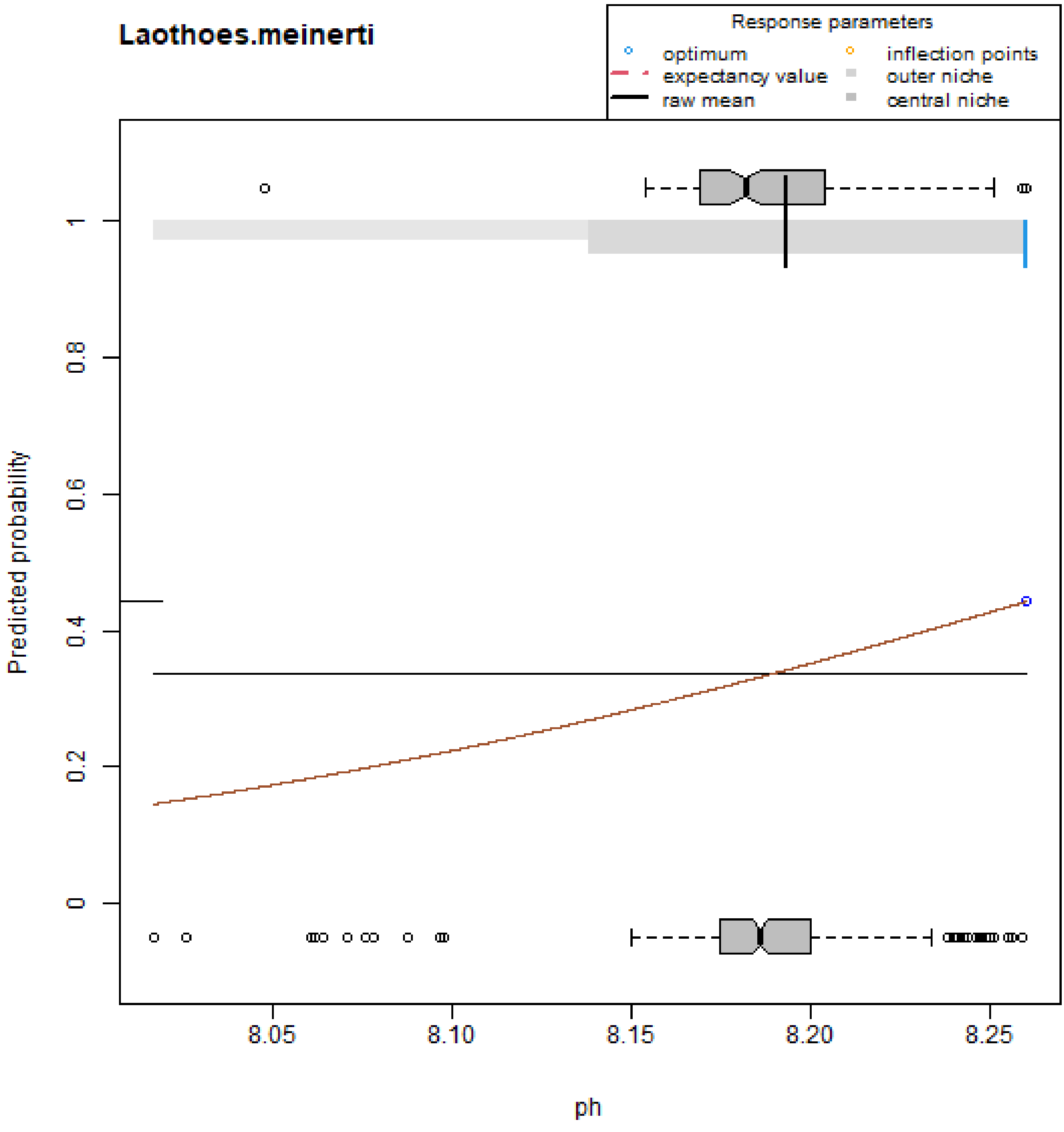
Laothoes.meinerti
Response parameters
optimum
expectancy value
raw mean
inflection points
outer niche
central niche
Predicted probability
0
0.2
0.4
0.6
0.8
1
8.05
8.10
8.15
8.20
8.25
ph

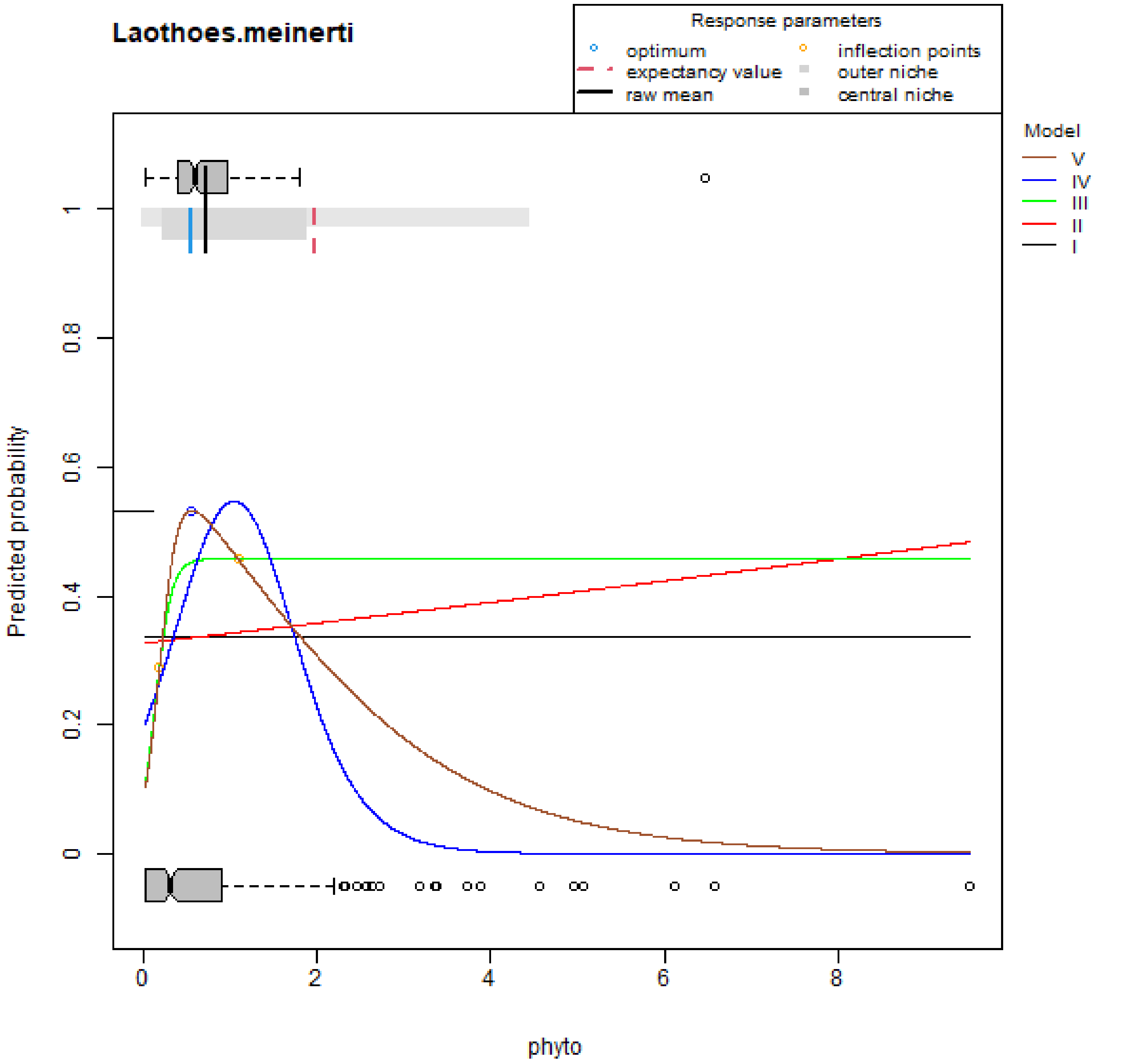
Laothoes.meinerti
Response parameters
optimum
inflection points
expectancy value
outer niche
raw mean
central niche
Model
V
IV
III
II
I
Predicted probability
0
0.2
0.4
0.6
0.8
1
0
2
4
6
8
phyto

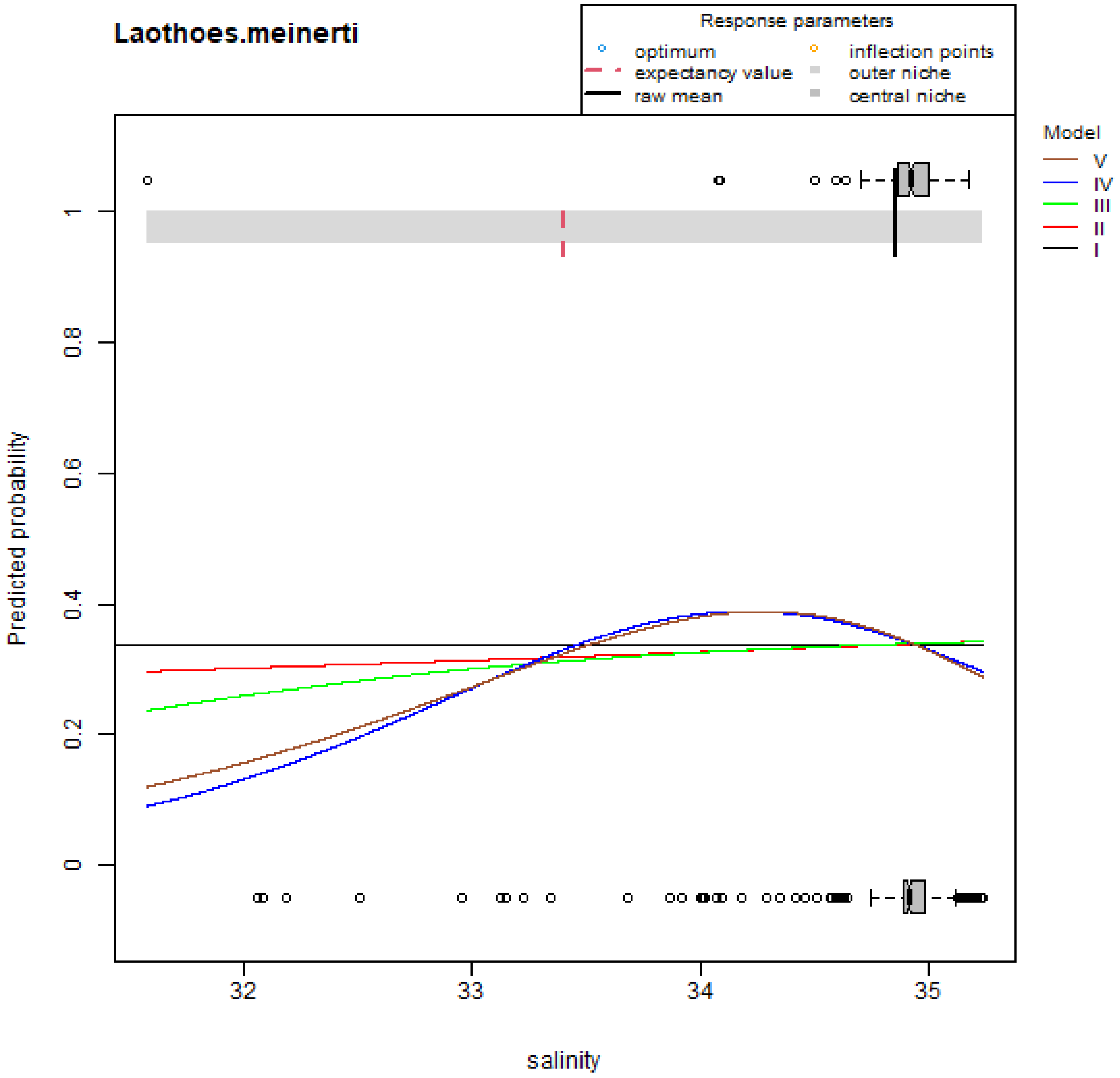

Laothoes.meinerti
Response parameters
optimum
expectancy value
raw mean
inflection points
outer niche
central niche
Model
V
IV
III
II
I
Predicted probability
0
0.2
0.4
0.6
0.8
1
32
33
34
35
salinity

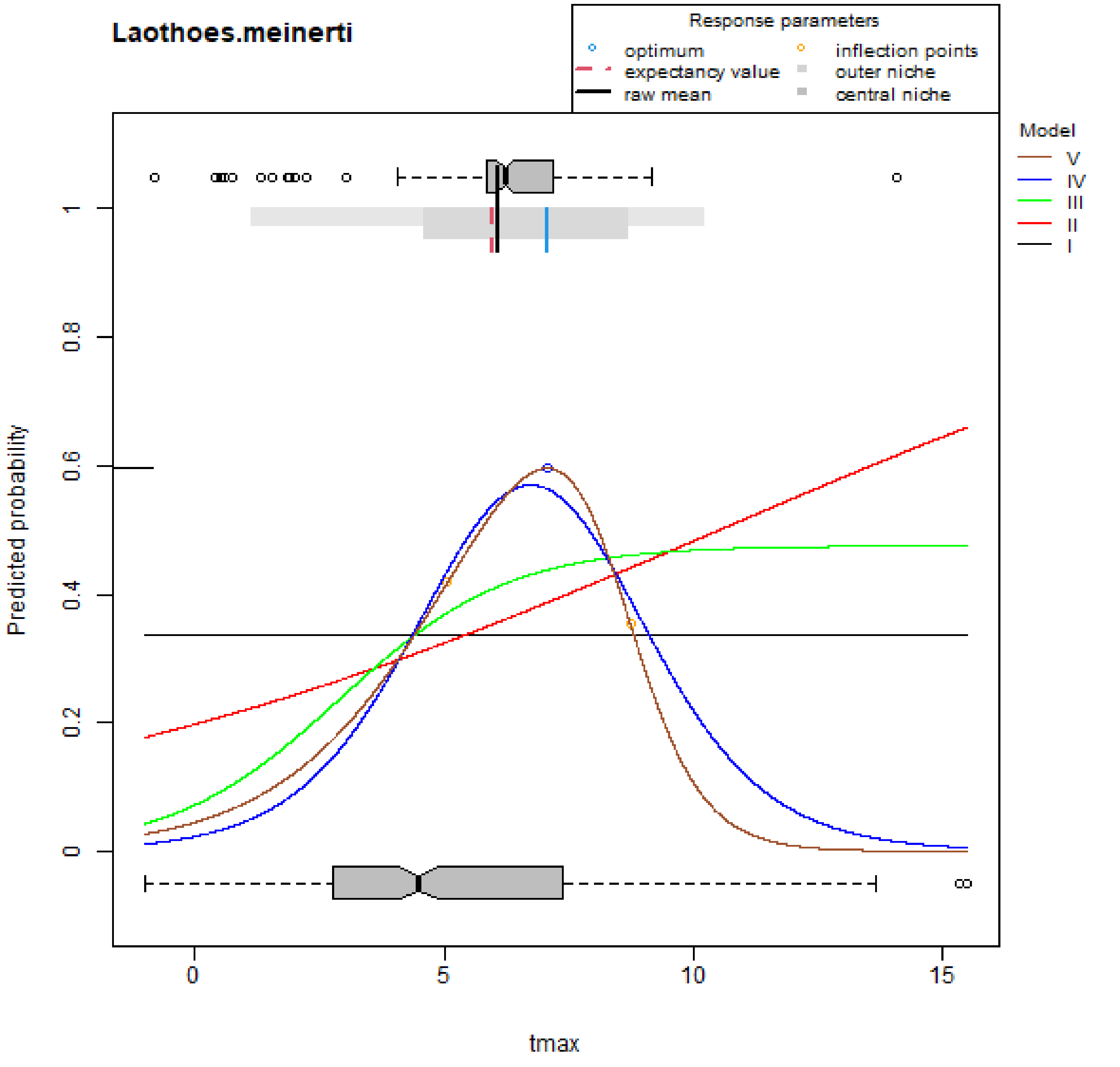

Laothoes.meinerti
Response parameters
optimum
inflection points
expectancy value
outer niche
raw mean
central niche
Model
V
IV
III
II
I
Predicted probability
0
0.2
0.4
0.6
0.8
1
0
5
10
15
tmax

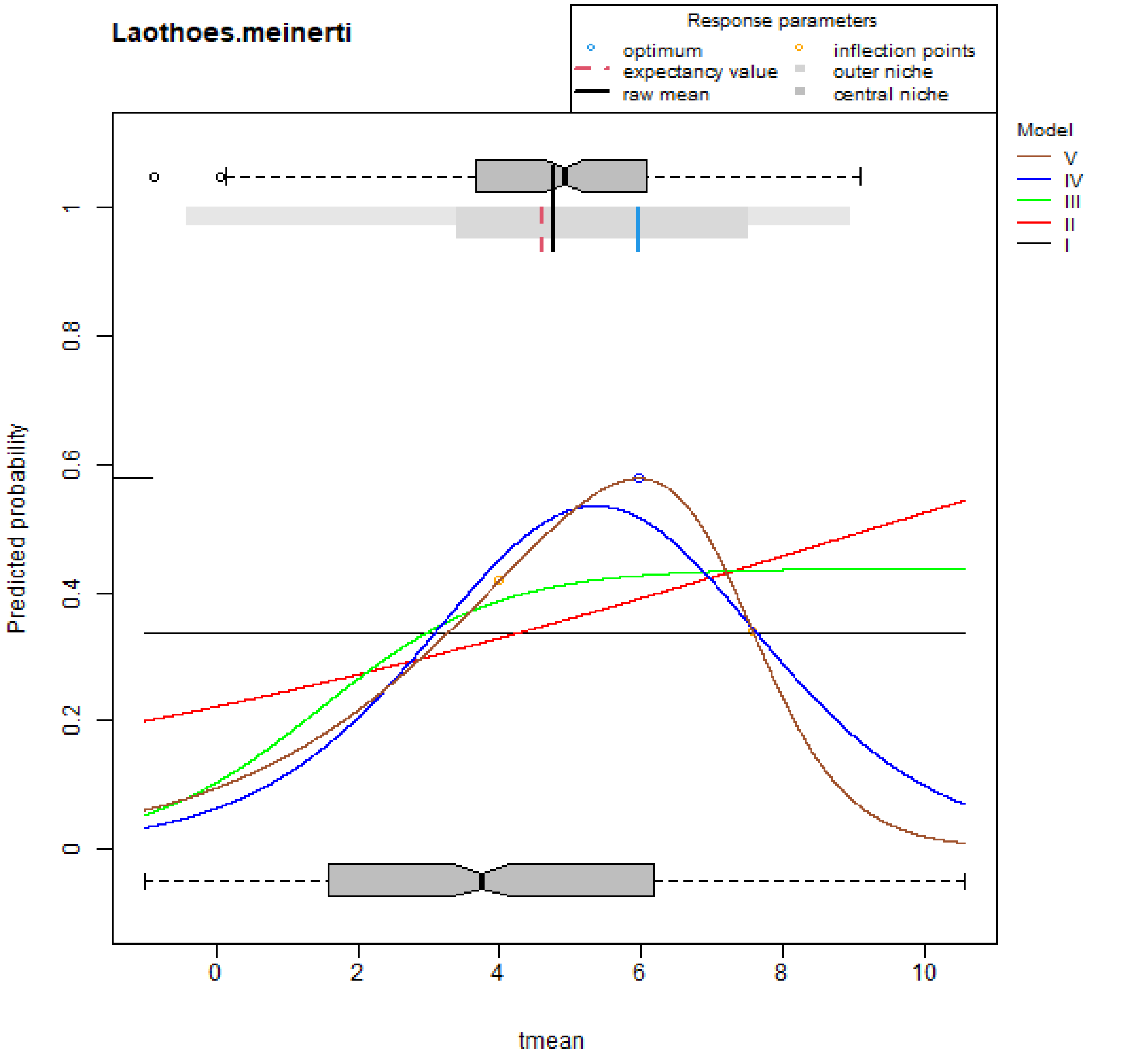

Laothoes.meinerti
Response parameters
optimum
expectancy value
raw mean
inflection points
outer niche
central niche
Model
V
IV
III
II
I
Predicted probability
0
0.2
0.4
0.6
0.8
1
0
2
4
6
8
10
tmean

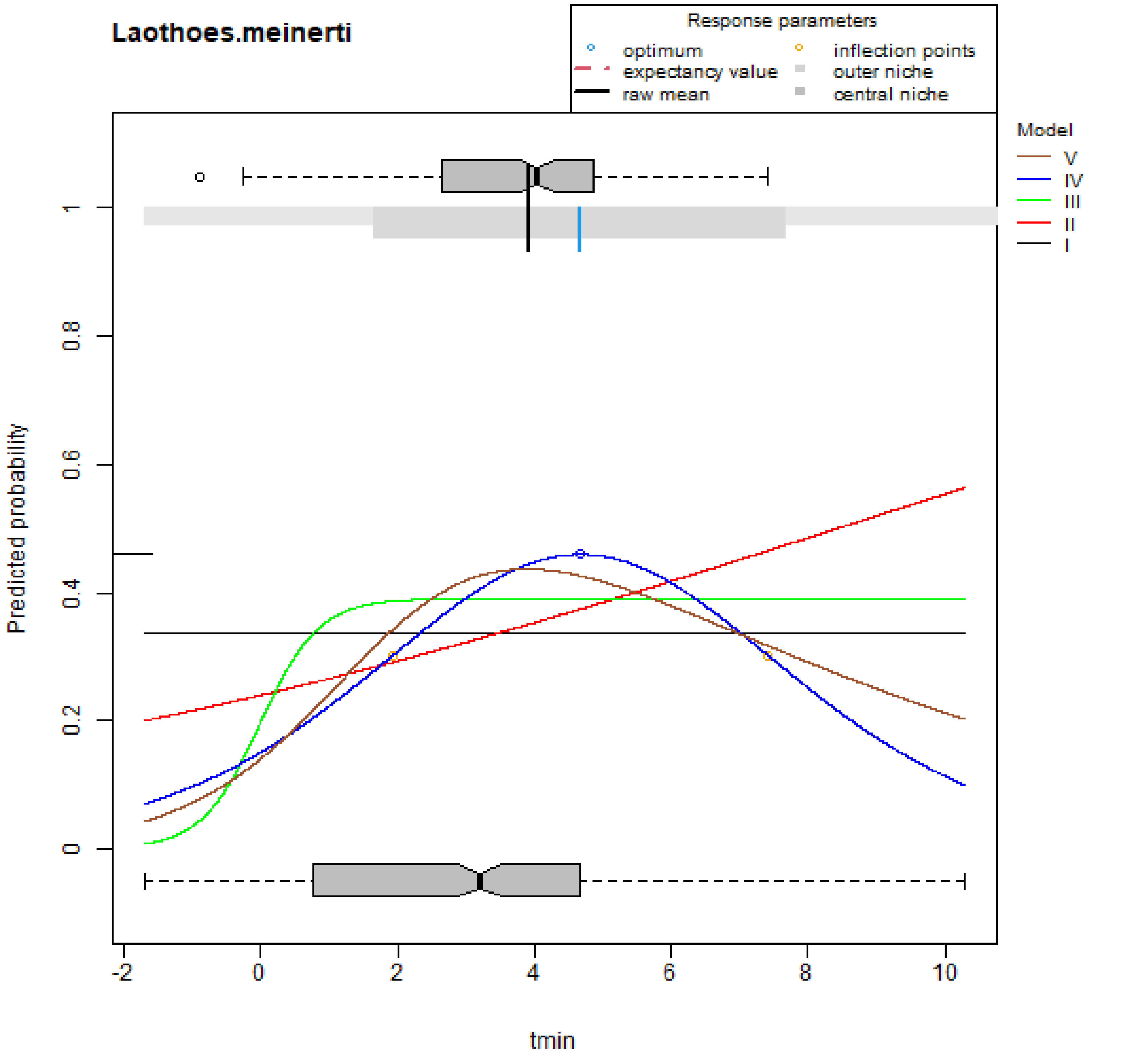

Laothoes.meinerti
Response parameters
optimum
expectancy value
raw mean
inflection points
outer niche
central niche
Model
V
IV
III
II
I
Predicted probability
0
0.2
0.4
0.6
0.8
1
-2
0
2
4
6
8
10
tmin

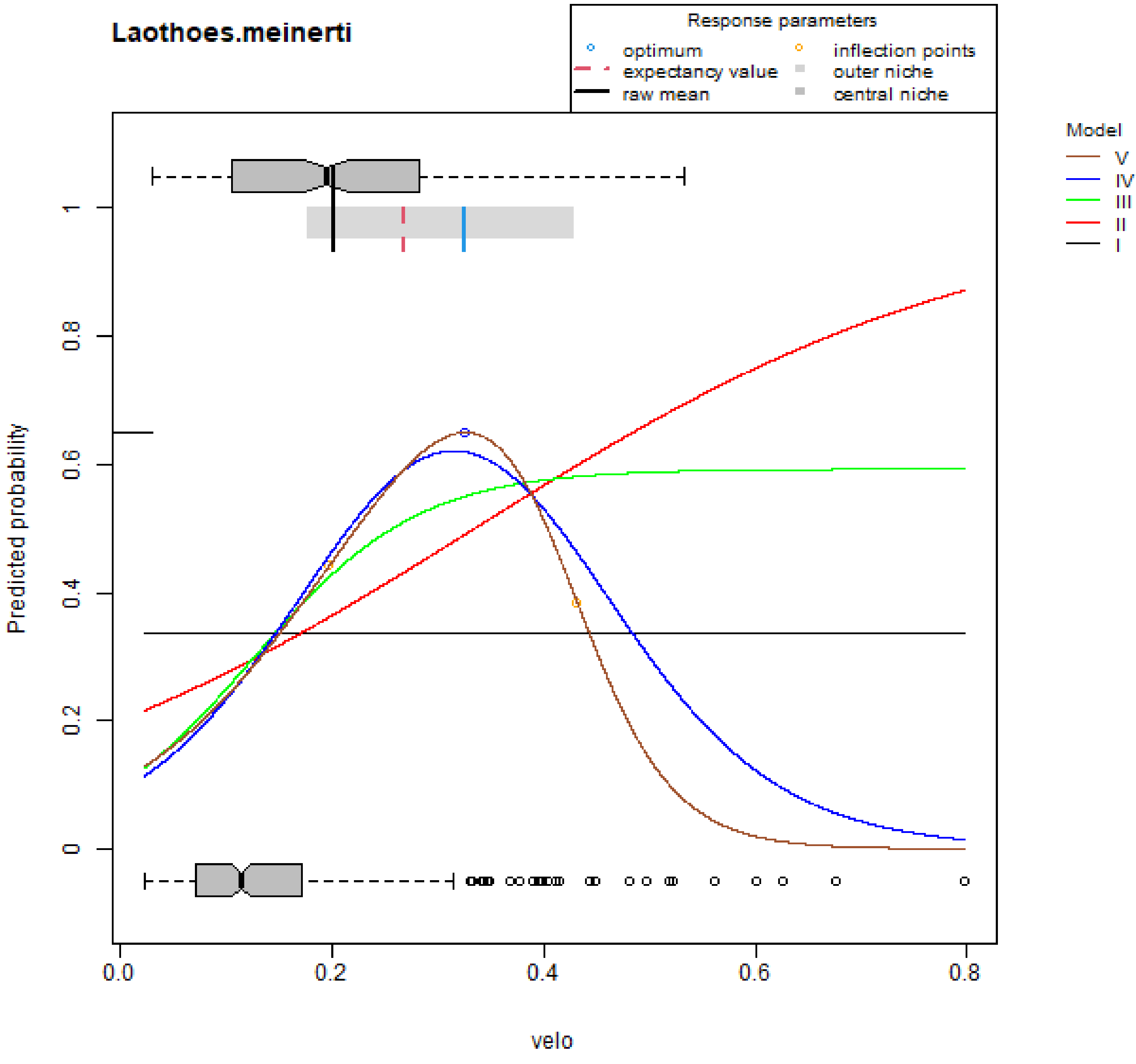

Laothoes.meinerti
Response parameters
optimum
expectancy value
raw mean
inflection points
outer niche
central niche
Model
V
IV
III
II
I
Predicted probability
0
0.2
0.4
0.6
0.8
1
0.0
0.2
0.4
0.6
0.8
velo

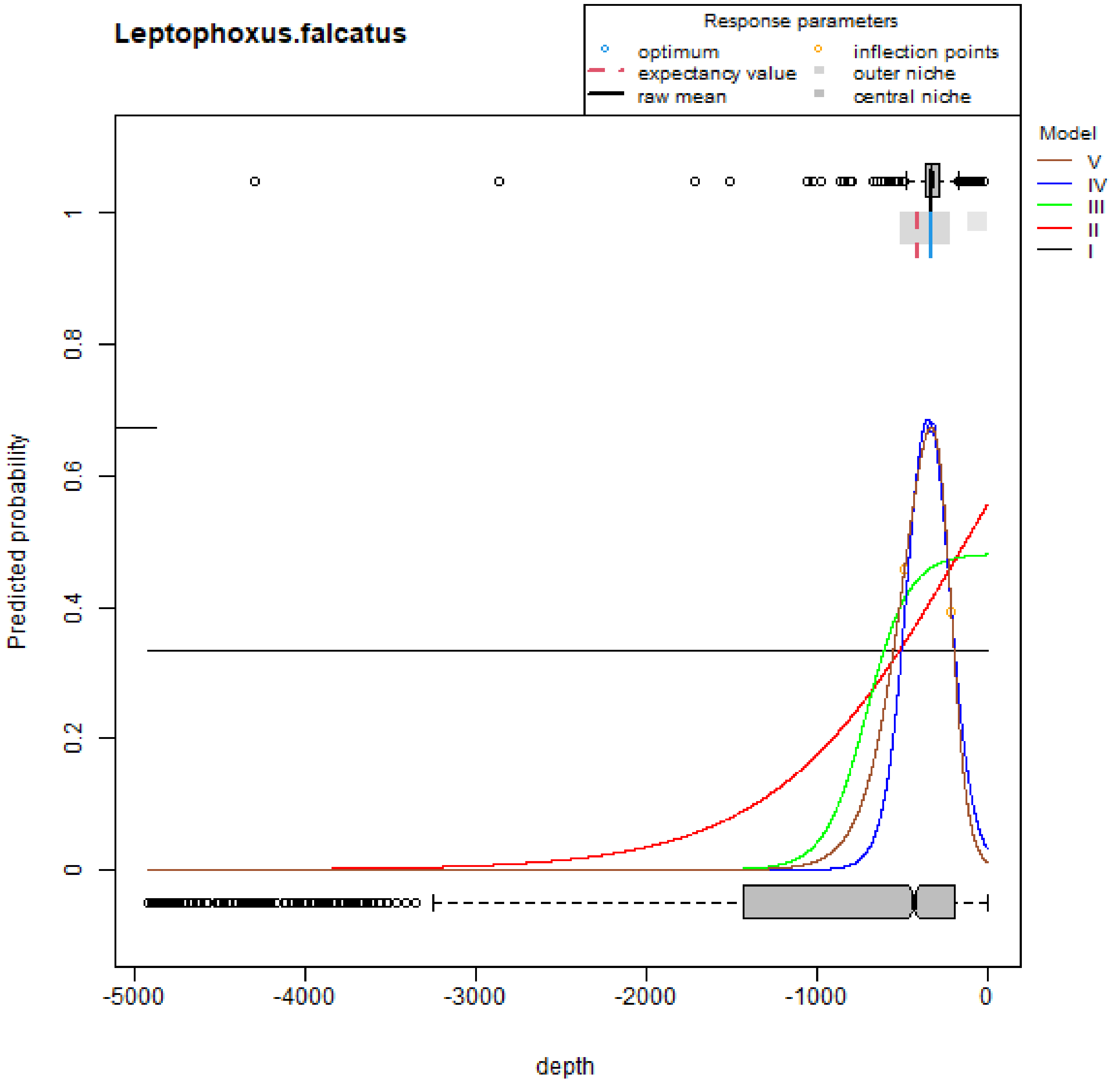
Leptophoxus.falcatus
Response parameters
optimum
expectancy value
raw mean
inflection points
outer niche
central niche
Model
V
IV
III
II
I
Predicted probability
0
0.2
0.4
0.6
0.8
1
-5000
-4000
-3000
-2000
-1000
0
depth

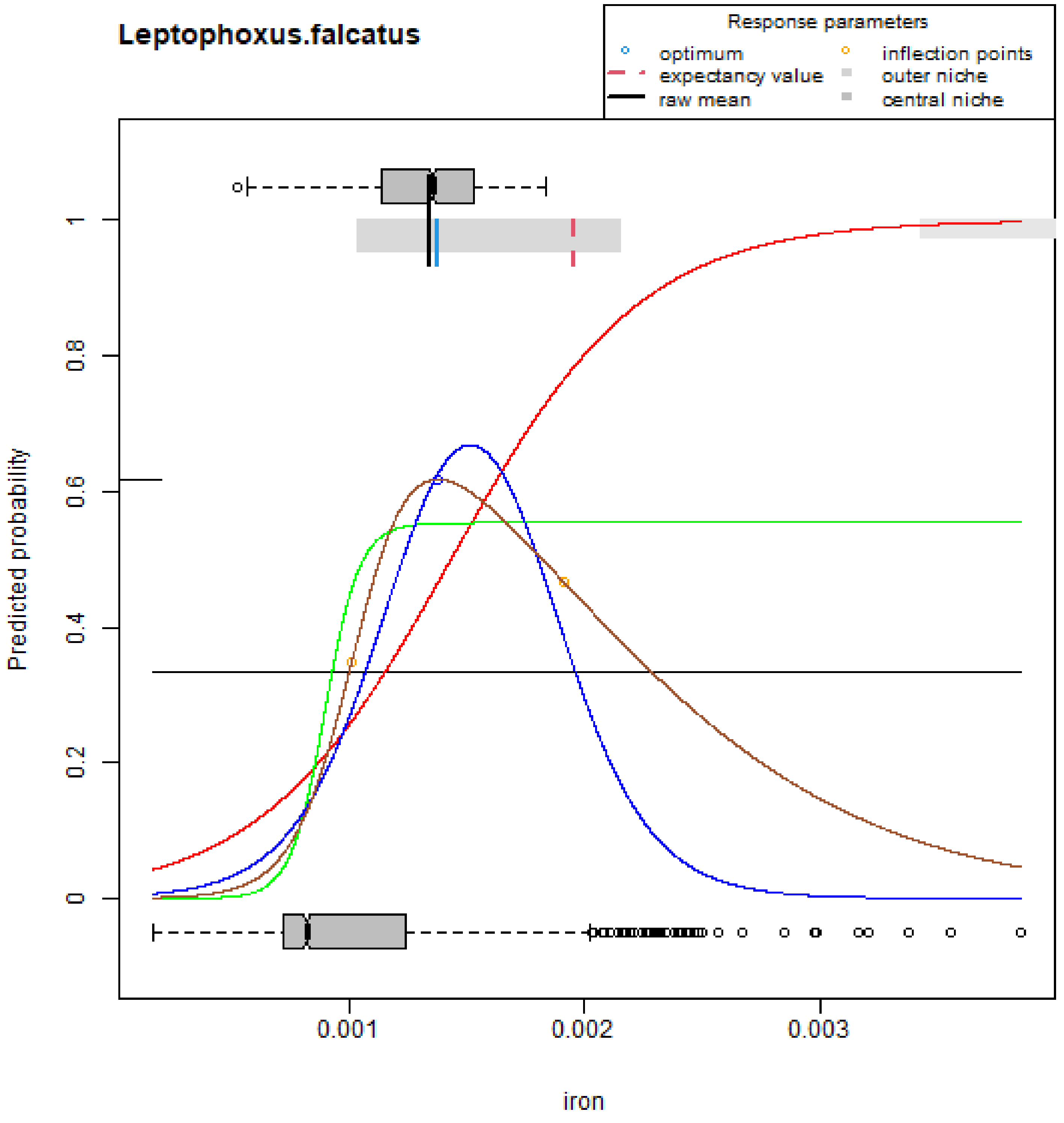

Leptophoxus.falcatus
Response parameters
optimum
expectancy value
raw mean
inflection points
outer niche
central niche
Predicted probability
0
0.2
0.4
0.6
0.8
1
0.001
0.002
0.003
iron

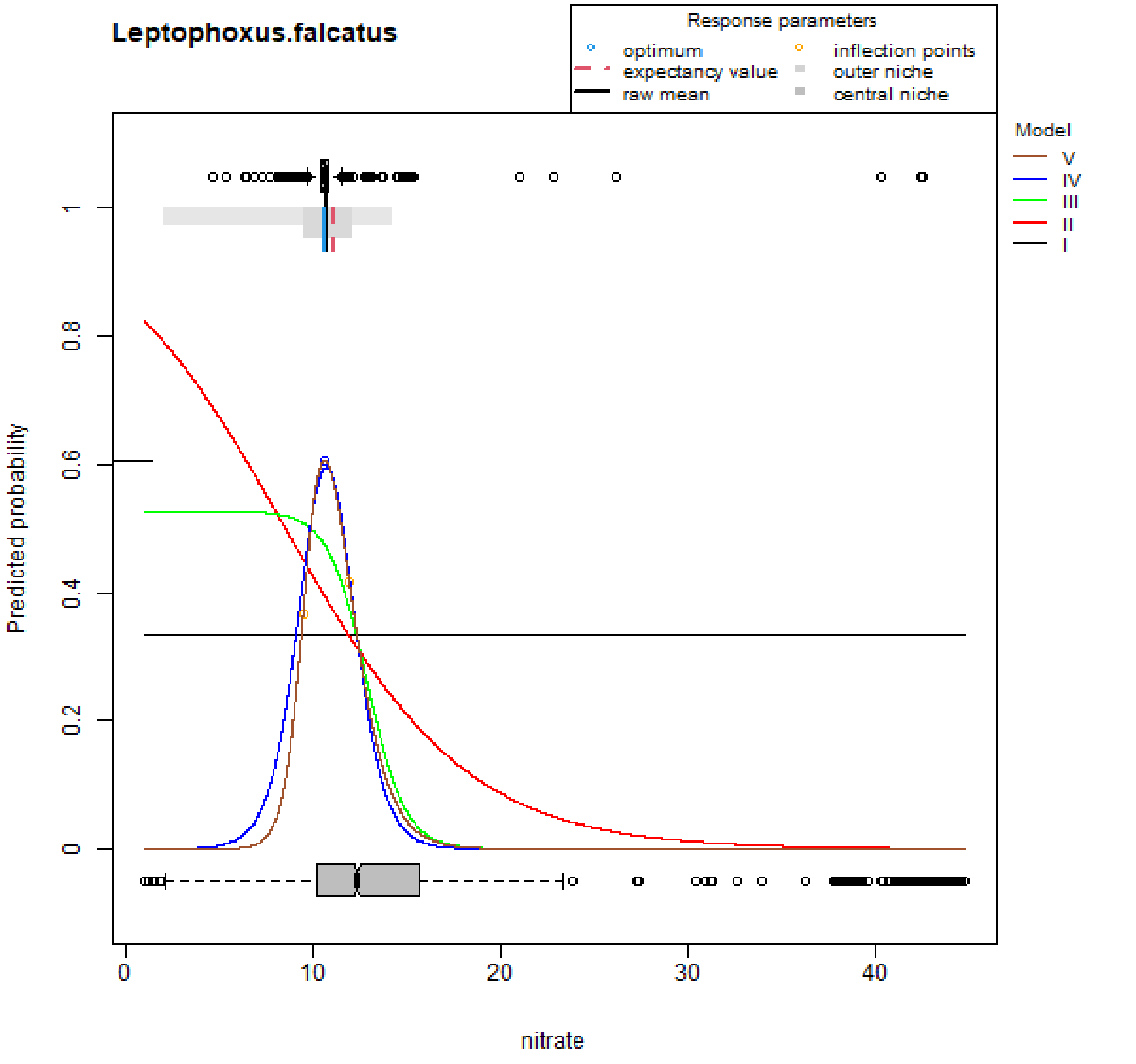

Leptophoxus.falcatus
Response parameters
optimum
expectancy value
raw mean
inflection points
outer niche
central niche
Model
V
IV
III
II
I
Predicted probability
0
0.2
0.4
0.6
0.8
1
0
10
20
30
40
nitrate

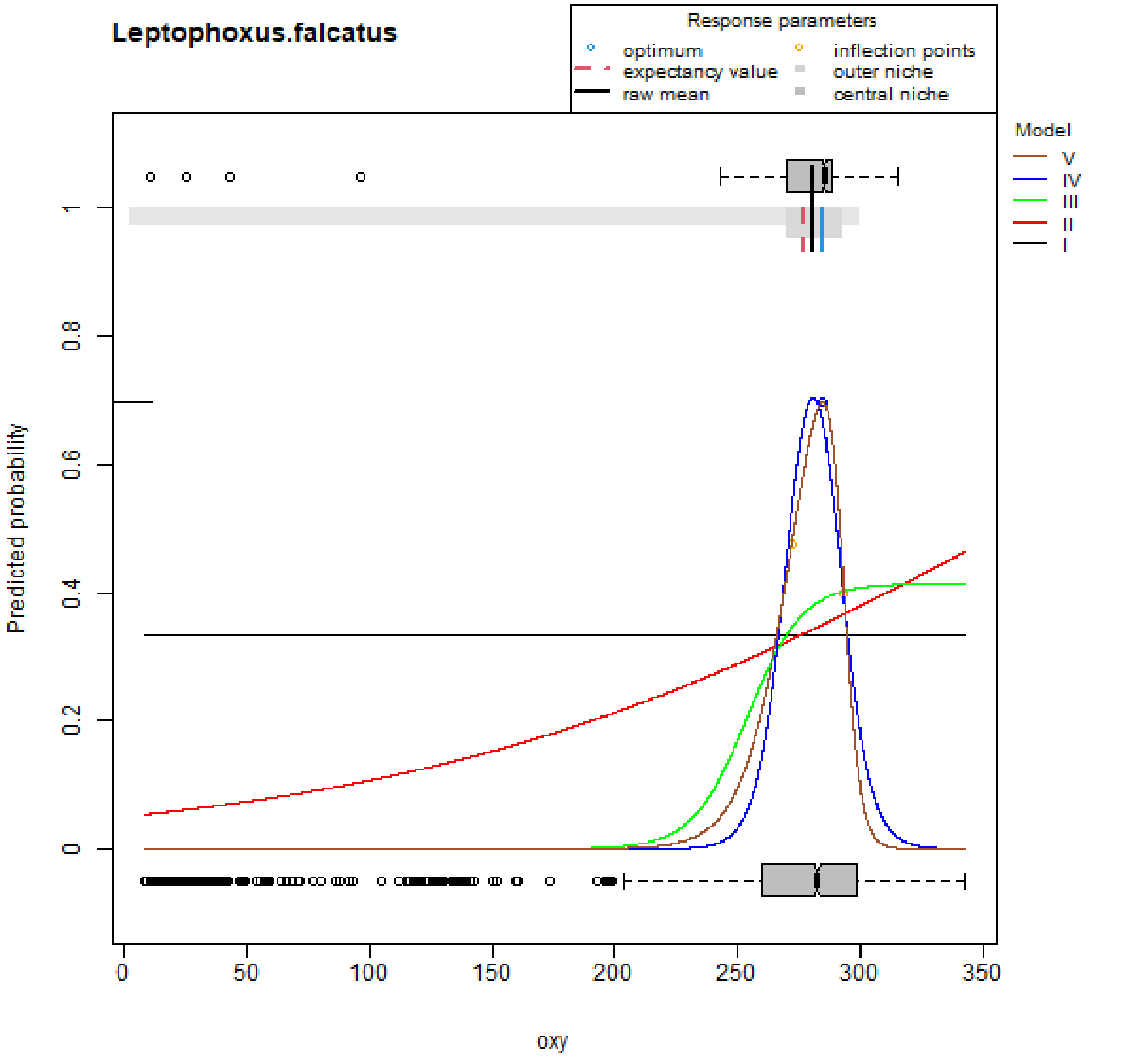

Leptophoxus.falcatus
Response parameters
optimum
expectancy value
raw mean
inflection points
outer niche
central niche
Model
V
IV
III
II
I
Predicted probability
0
0.2
0.4
0.6
0.8
1
0
50
100
150
200
250
300
350
oxy

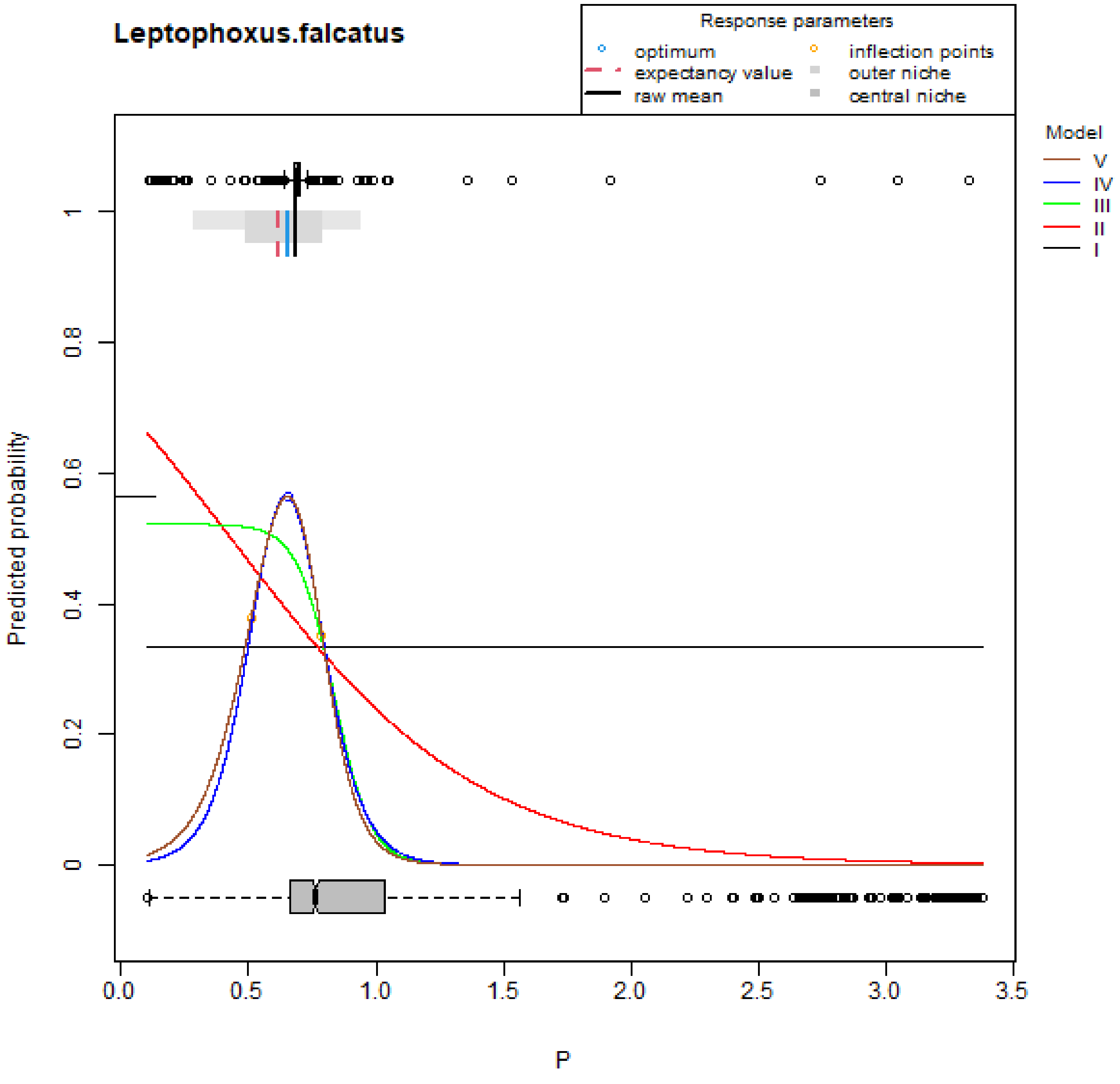
Leptophoxus.falcatus
Response parameters
optimum
expectancy value
raw mean
inflection points
outer niche
central niche
Model
V
IV
III
II
I
Predicted probability
0
0.2
0.4
0.6
0.8
1
0.0
0.5
1.0
1.5
2.0
2.5
3.0
3.5
P

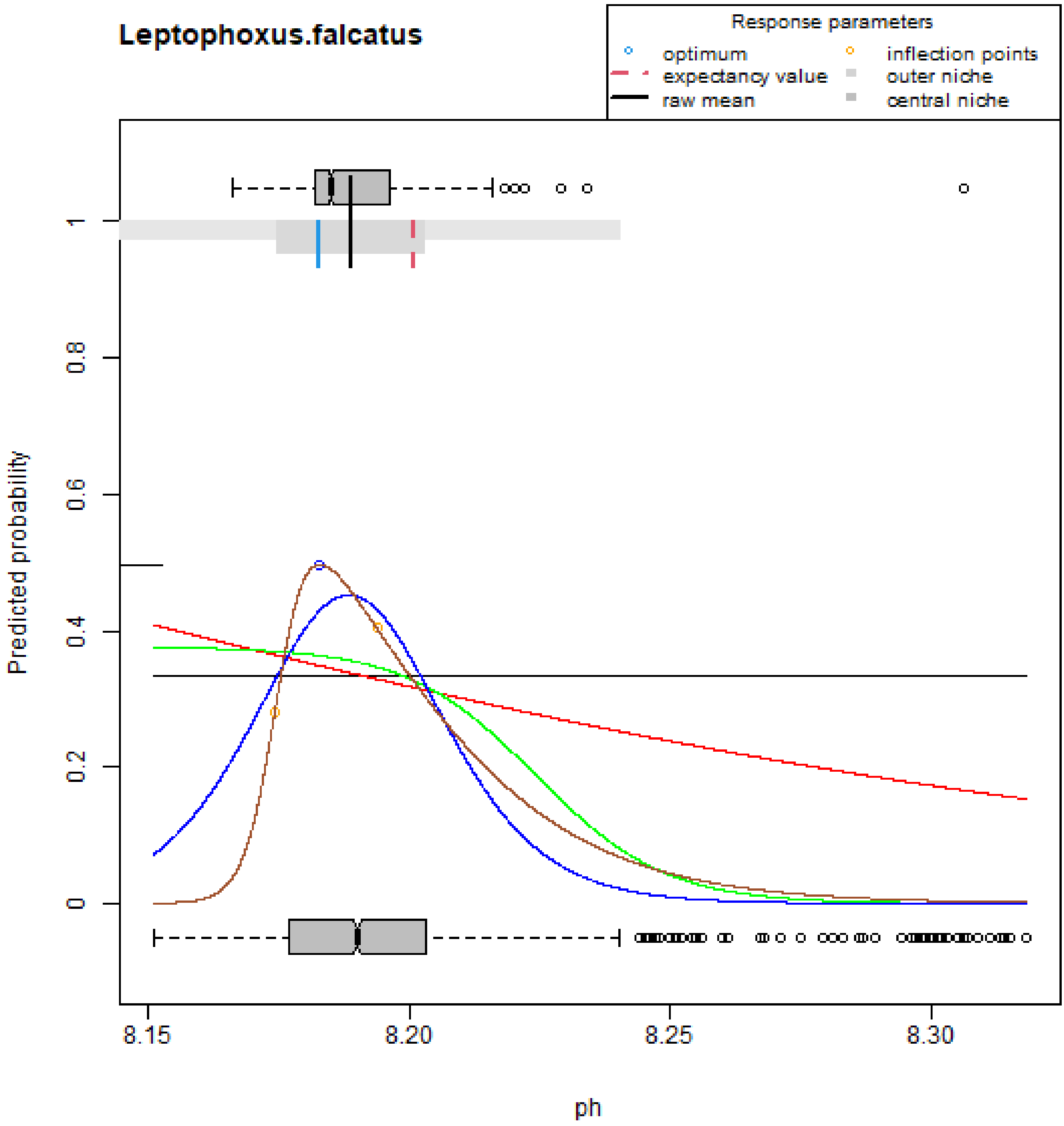
Leptophoxus.falcatus
Response parameters
optimum
expectancy value
raw mean
inflection points
outer niche
central niche
Predicted probability
0
0.2
0.4
0.6
0.8
1
8.15
8.20
8.25
8.30
ph

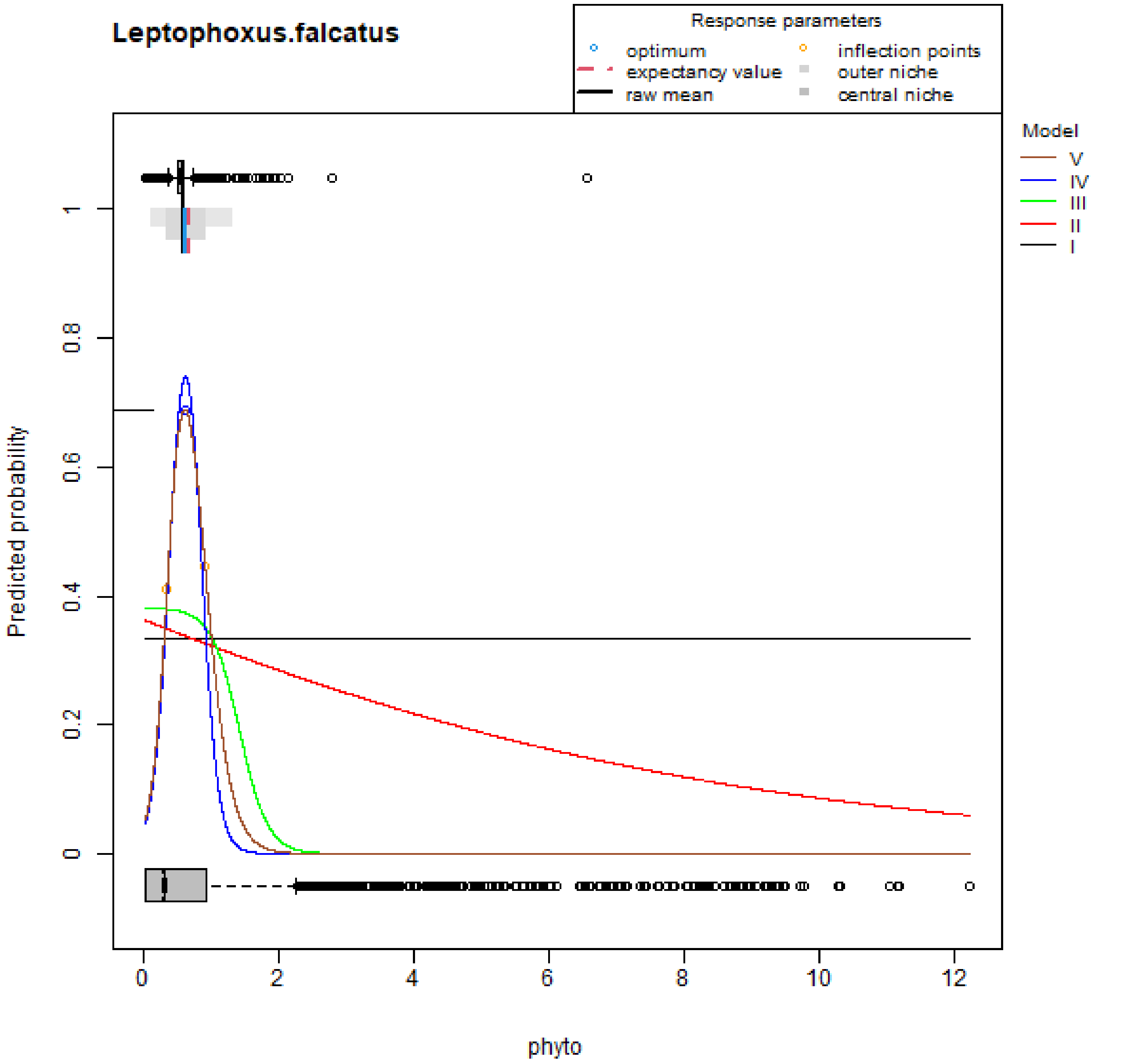
Leptophoxus.falcatus
Response parameters
optimum
expectancy value
raw mean
inflection points
outer niche
central niche
Model
V
IV
III
II
I
Predicted probability
0
0.2
0.4
0.6
0.8
1
0
2
4
6
8
10
12
phyto

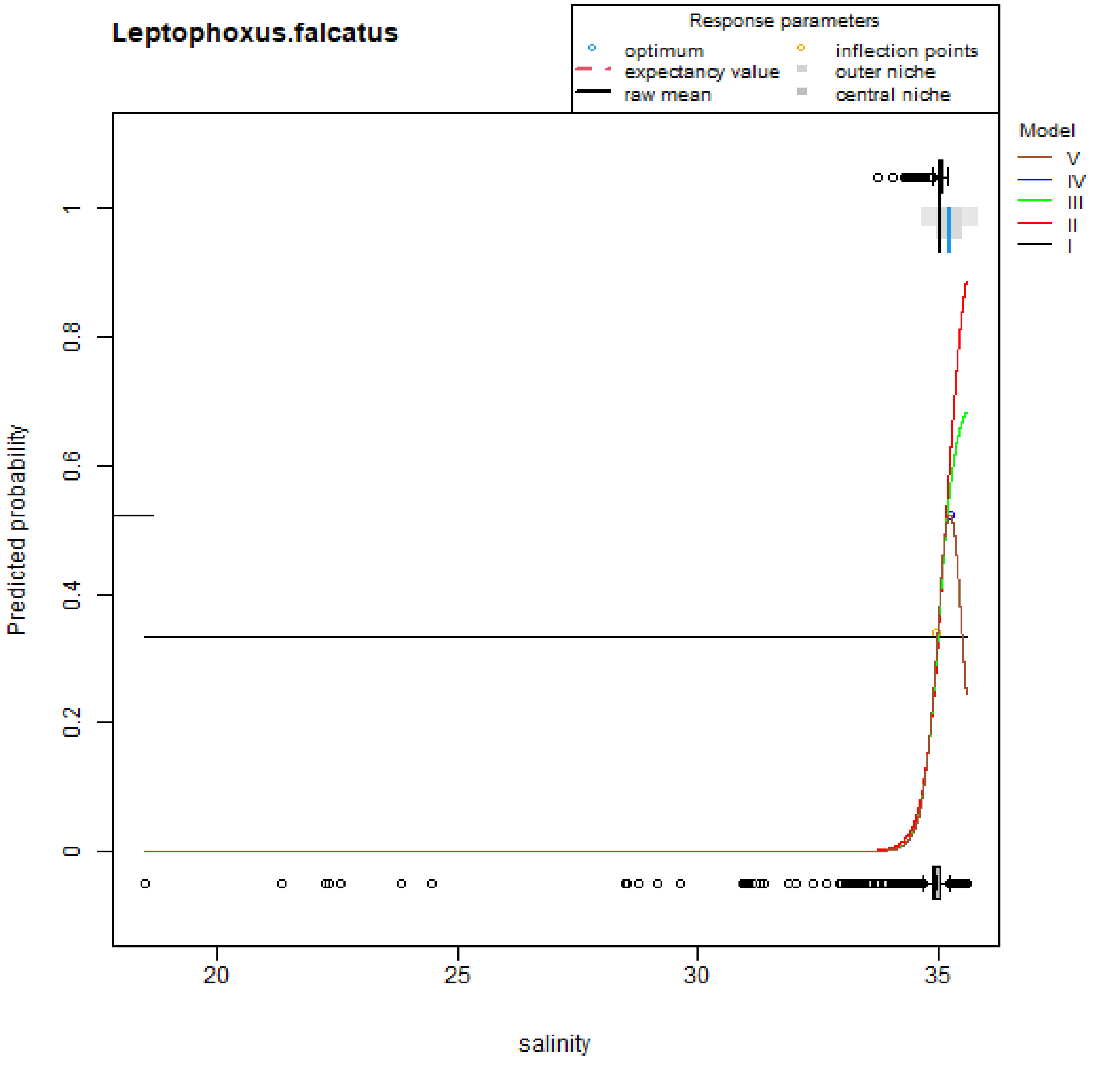

Leptophoxus.falcatus
Response parameters
optimum
expectancy value
raw mean
inflection points
outer niche
central niche
Model
V
IV
III
II
I
Predicted probability
0
0.2
0.4
0.6
0.8
1
20
25
30
35
salinity

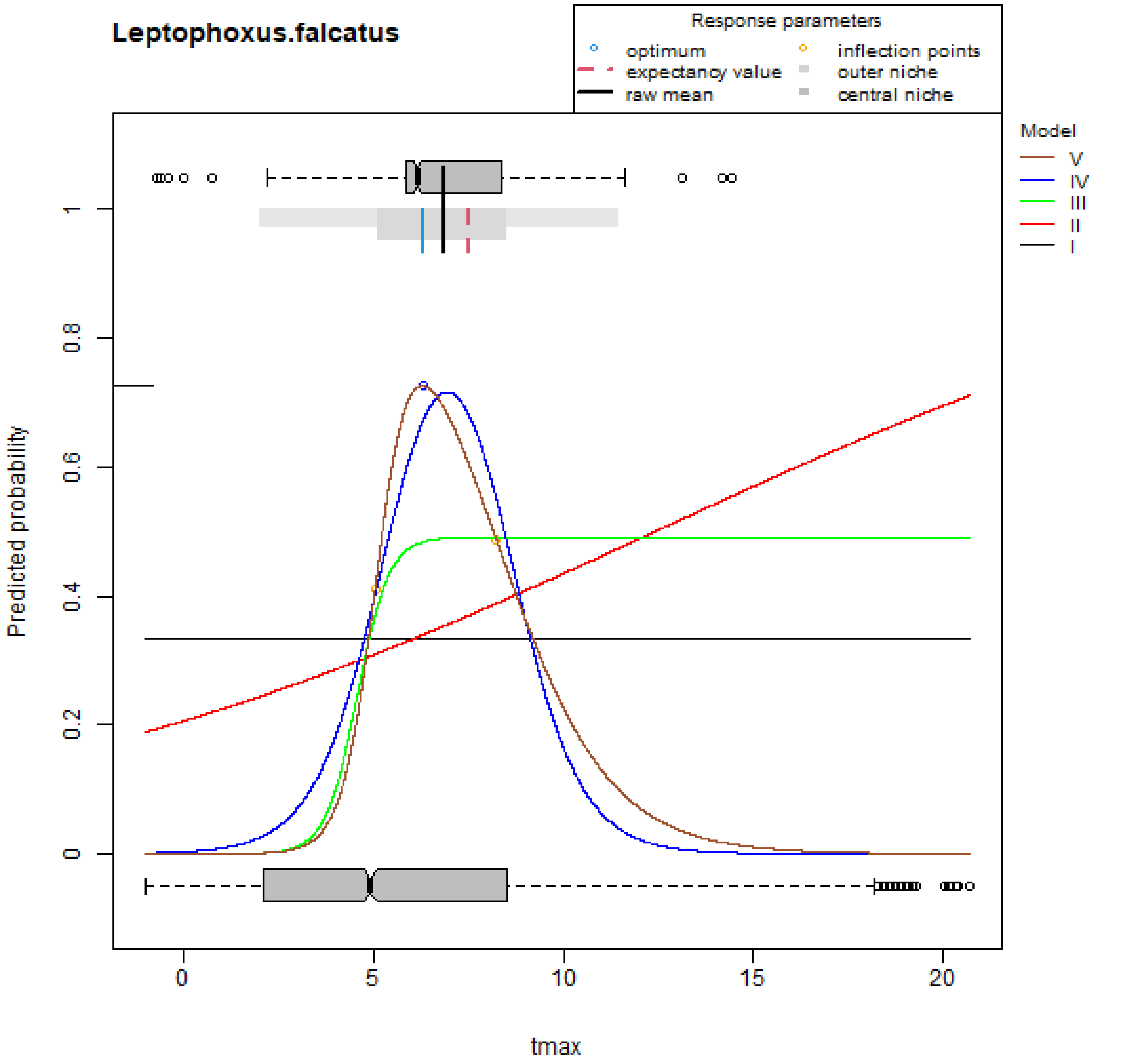
Leptophoxus.falcatus
Response parameters
optimum
expectancy value
raw mean
inflection points
outer niche
central niche
Model
V
IV
III
II
I
Predicted probability
0
0.2
0.4
0.6
0.8
1
0
5
10
15
20
tmax

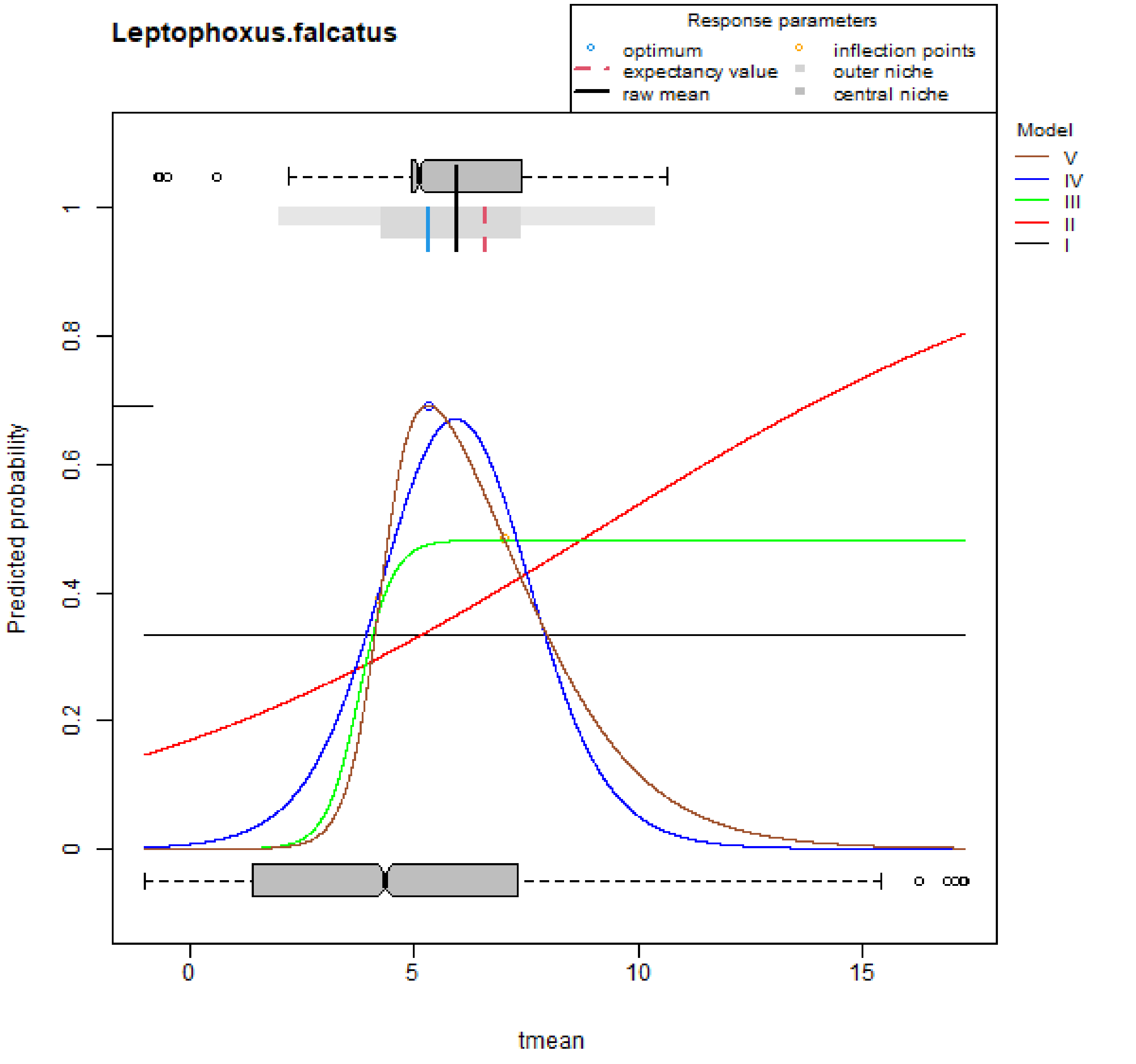
Leptophoxus.falcatus
Response parameters
optimum
expectancy value
raw mean
inflection points
outer niche
central niche
Model
V
IV
III
II
I
Predicted probability
0
0.2
0.4
0.6
0.8
1
0
5
10
15
tmean

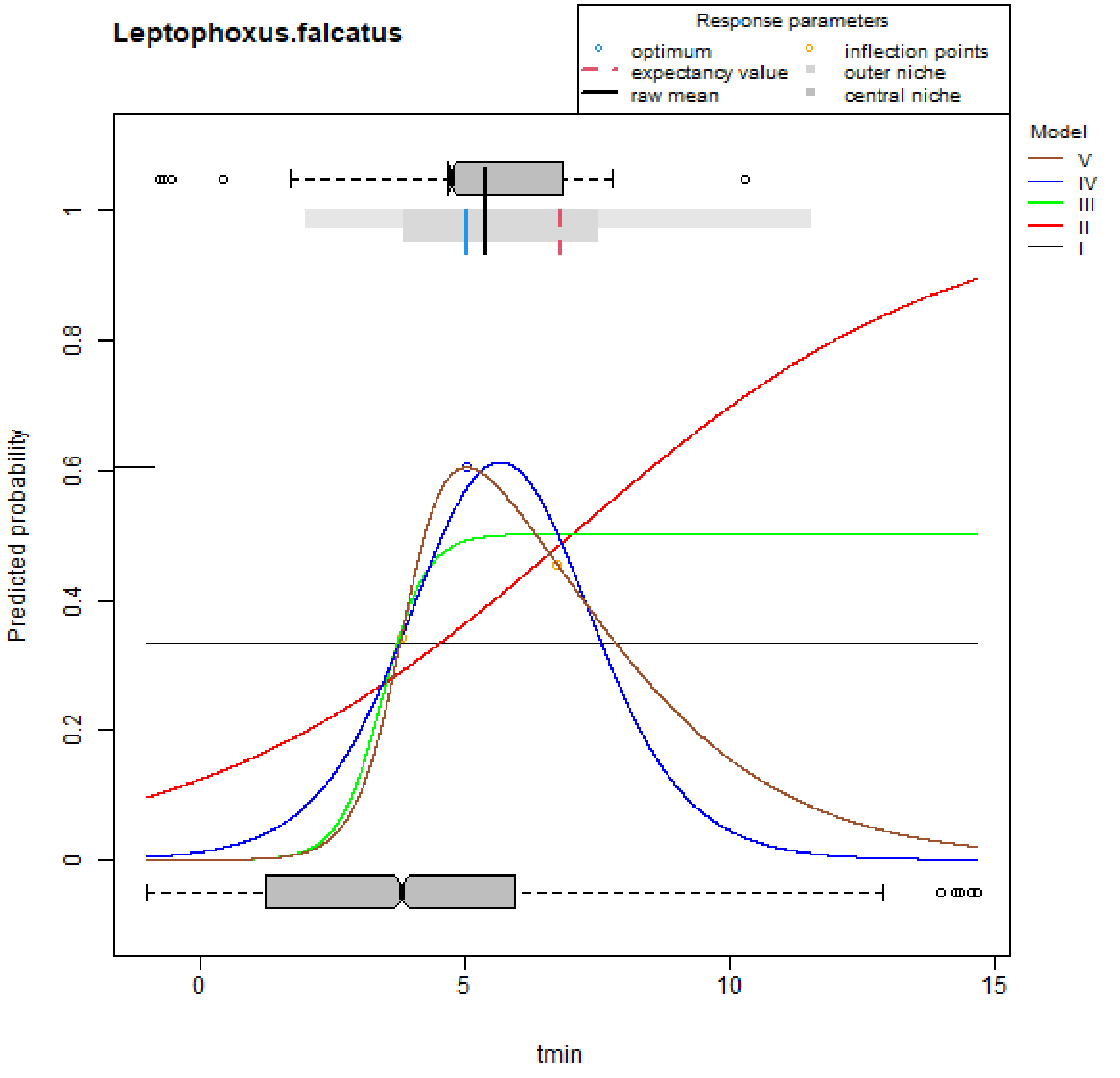

Leptophoxus.falcatus
Response parameters
optimum
expectancy value
raw mean
inflection points
outer niche
central niche
Model
V
IV
III
II
I
Predicted probability
0
0.2
0.4
0.6
0.8
1
0
5
10
15
tmin

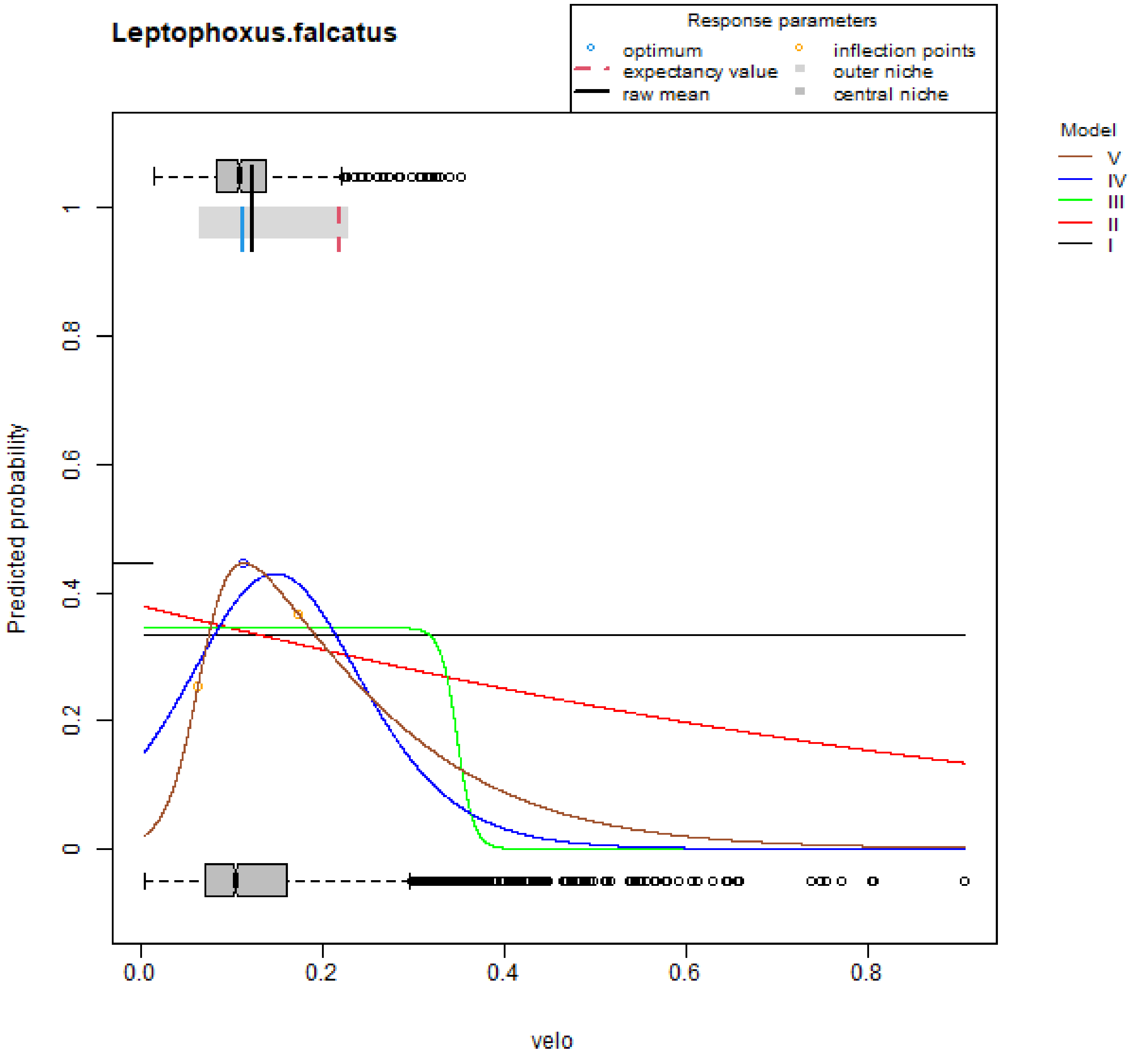

Leptophoxus.falcatus
Response parameters
optimum
expectancy value
raw mean
inflection points
outer niche
central niche
Model
V
IV
III
II
I
Predicted probability
0
0.2
0.4
0.6
0.8
1
0.0
0.2
0.4
0.6
0.8
velo

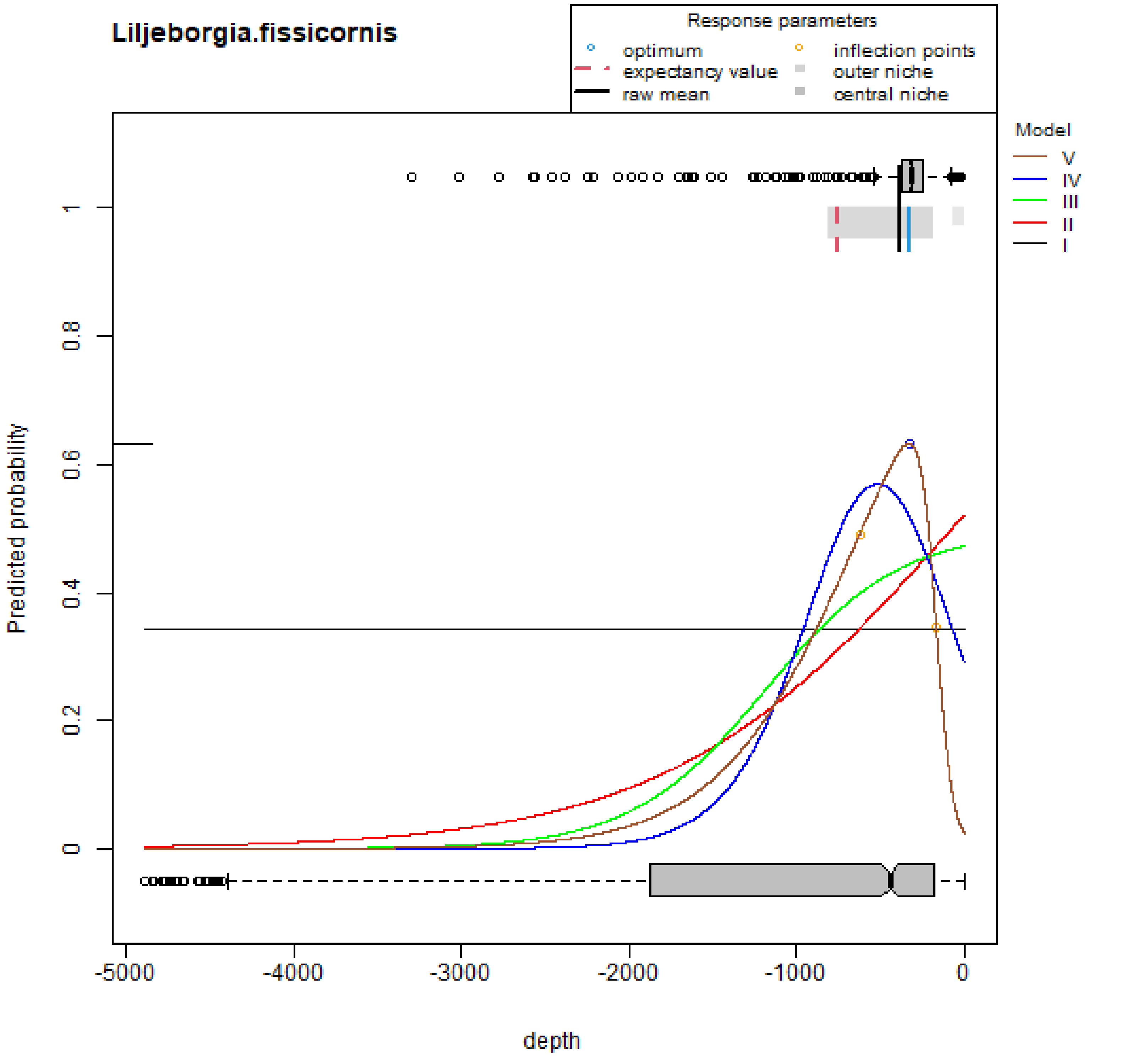
Liljeborgia.fissicornis
Response parameters
optimum
expectancy value
raw mean
inflection points
outer niche
central niche
Model
V
IV
III
II
I
Predicted probability
0
0.2
0.4
0.6
0.8
1
-5000
-4000
-3000
-2000
-1000
0
depth

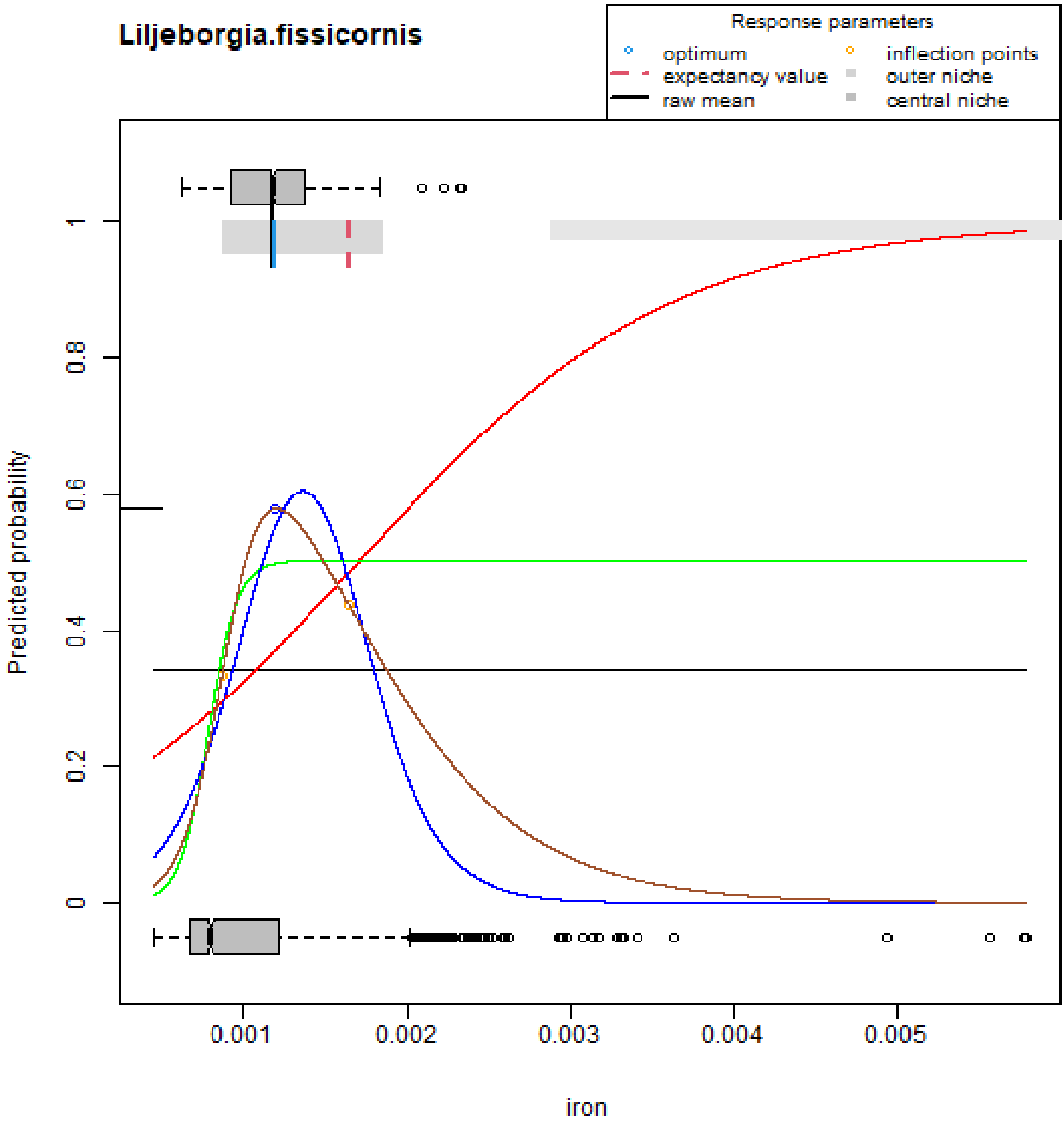
Liljeborgia.fissicornis
Response parameters
optimum
expectancy value
raw mean
inflection points
outer niche
central niche
Predicted probability
0
0.2
0.4
0.6
0.8
1
0.001
0.002
0.003
0.004
0.005
iron

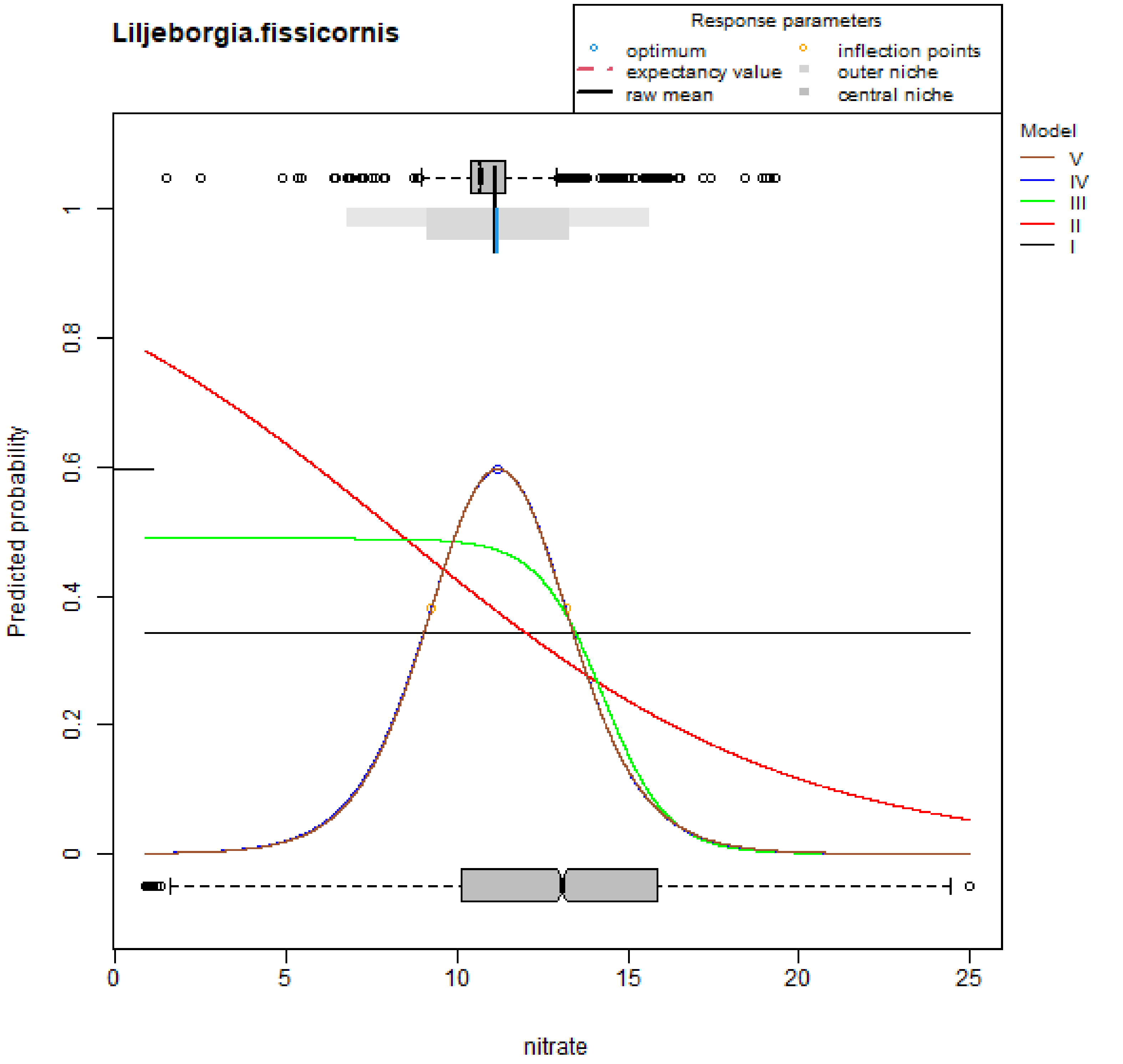

Liljeborgia.fissicornis
Response parameters
optimum
expectancy value
raw mean
inflection points
outer niche
central niche
Model
V
IV
III
II
I
Predicted probability
0
0.2
0.4
0.6
0.8
1
0
5
10
15
20
25
nitrate

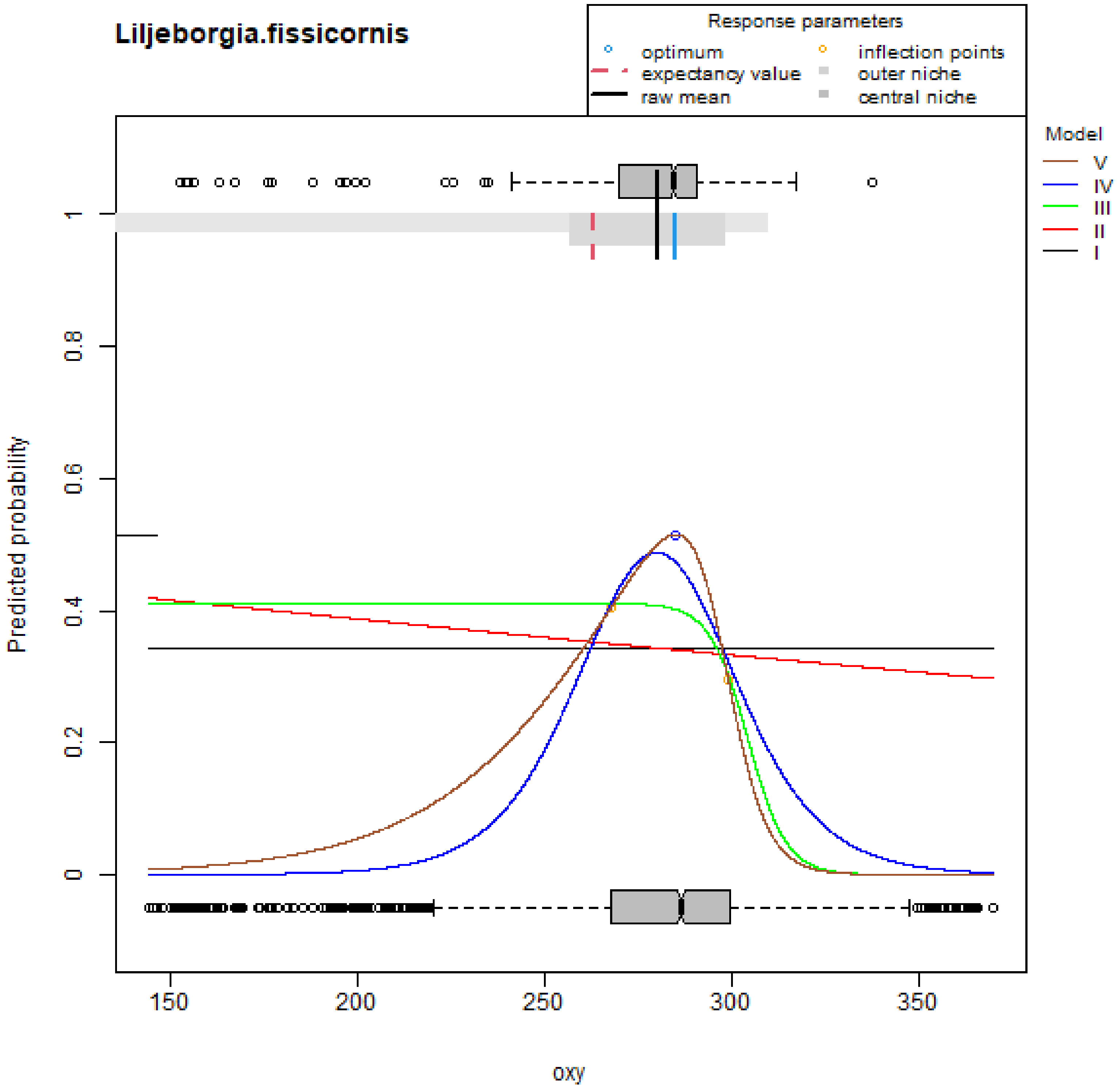
Liljeborgia.fissicornis
Response parameters
optimum
expectancy value
raw mean
inflection points
outer niche
central niche
Model
V
IV
III
II
I
Predicted probability
0
0.2
0.4
0.6
0.8
1
150
200
250
300
350
oxy

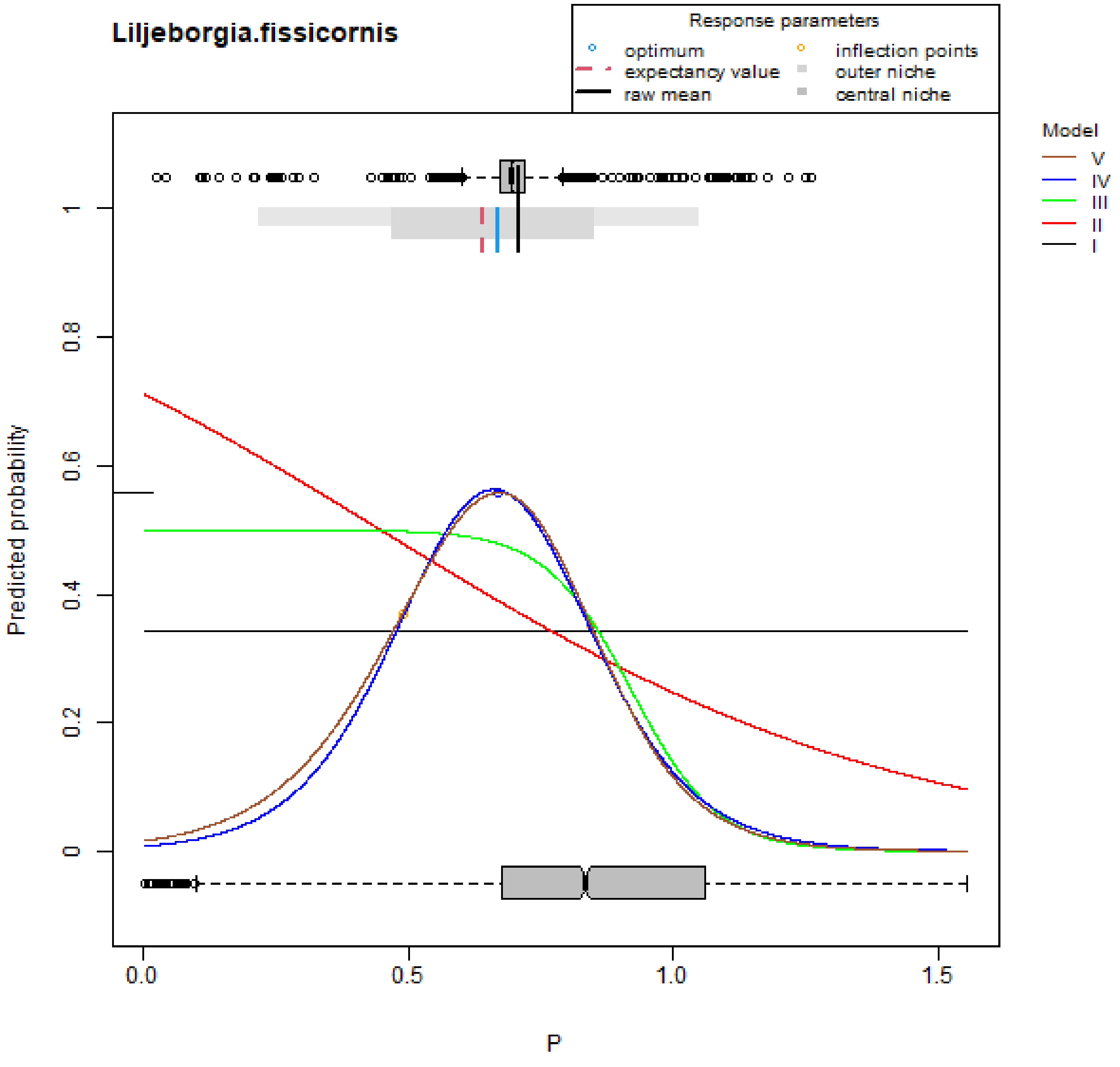

Liljeborgia.fissicornis
Response parameters
optimum
expectancy value
raw mean
inflection points
outer niche
central niche
Model
V
IV
III
II
I
Predicted probability
0
0.2
0.4
0.6
0.8
1
0.0
0.5
1.0
1.5
P

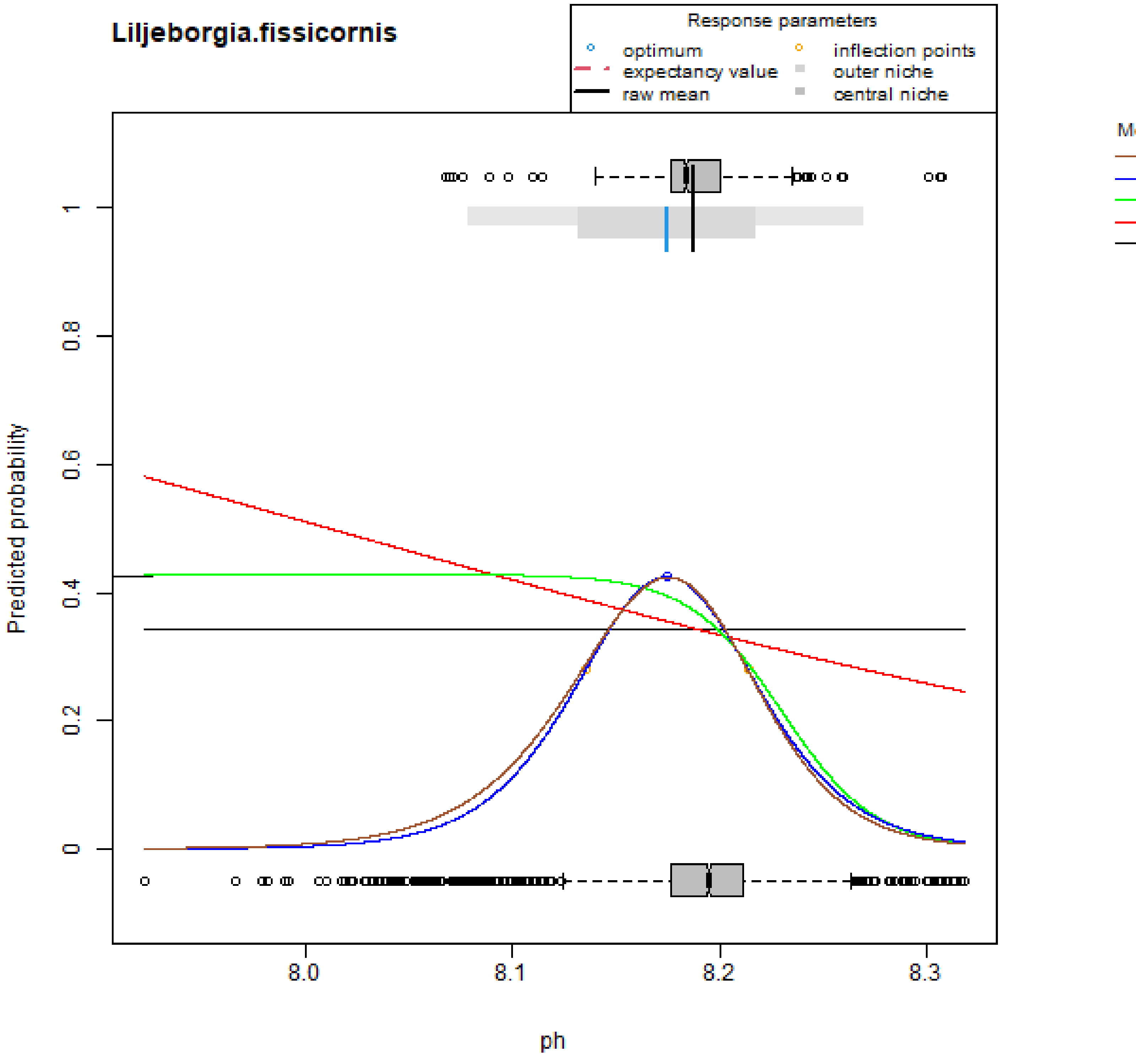

Liljeborgia.fissicornis
Response parameters
optimum
expectancy value
raw mean
inflection points
outer niche
central niche
Predicted probability
0
0.2
0.4
0.6
0.8
1
8.0
8.1
8.2
8.3
ph

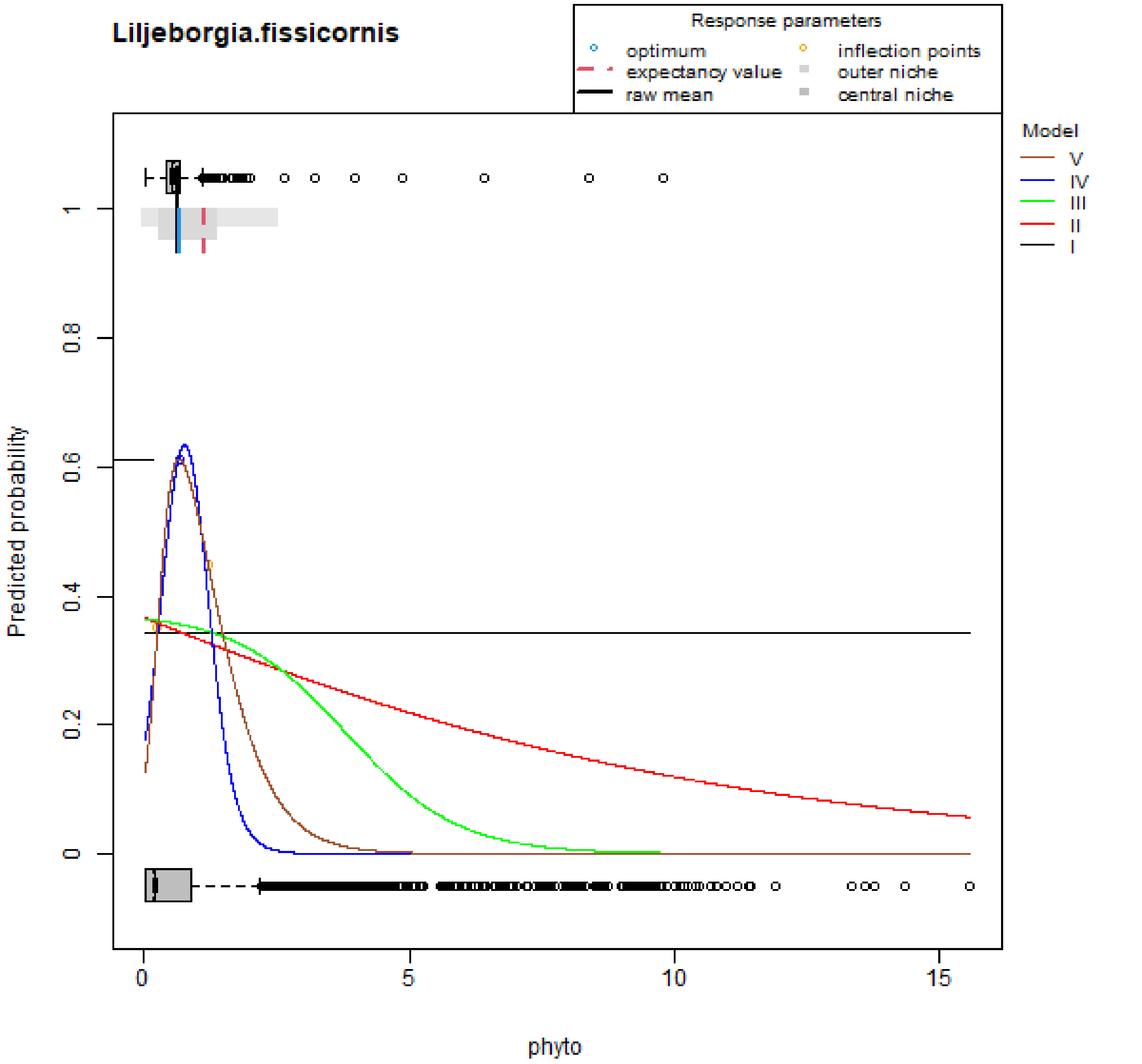

Liljeborgia.fissicornis
Response parameters
optimum
expectancy value
raw mean
inflection points
outer niche
central niche
Model
V
IV
III
II
I
Predicted probability
0
0.2
0.4
0.6
0.8
1
0
5
10
15
phyto

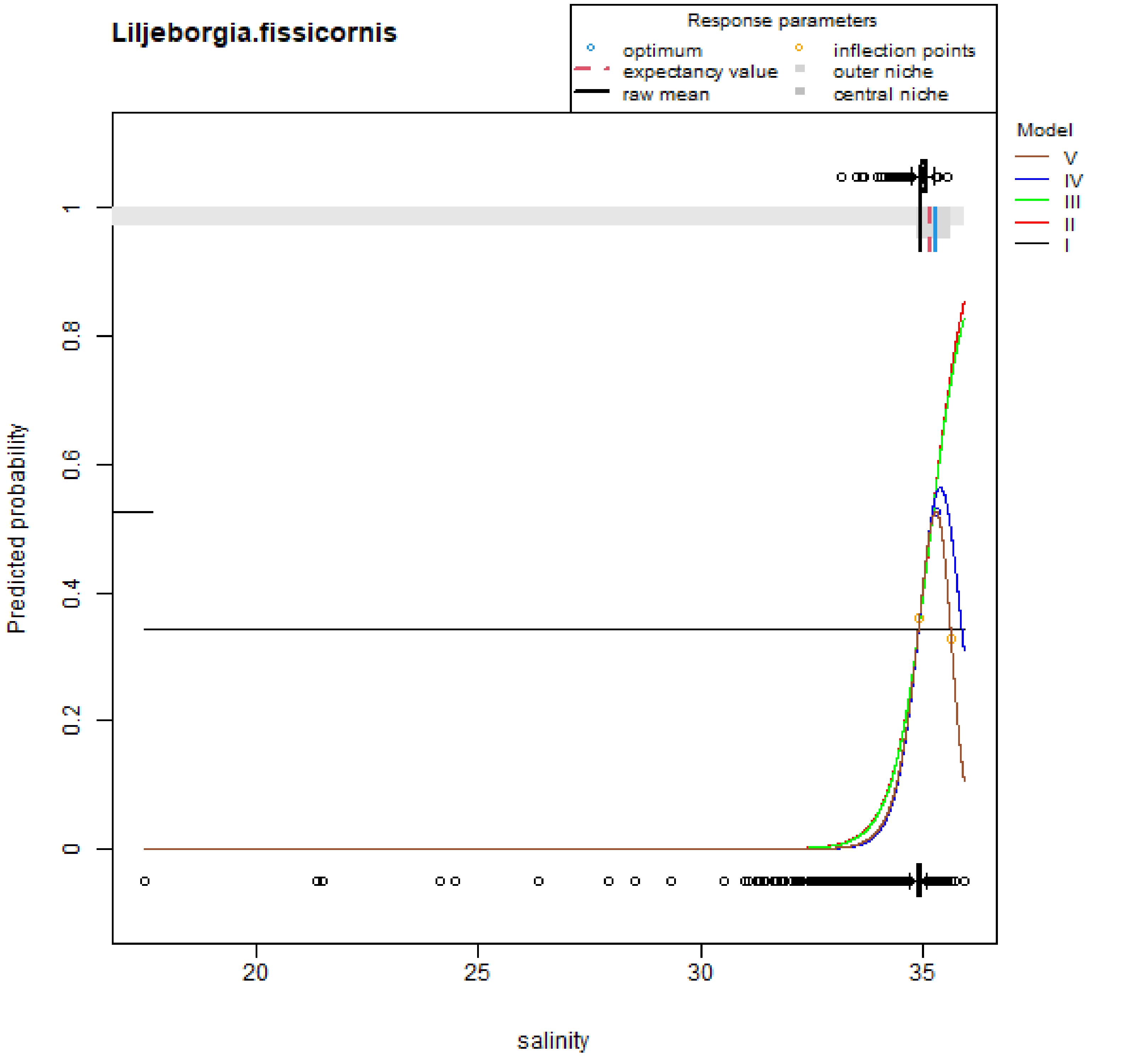
Liljeborgia.fissicornis
Response parameters
optimum
expectancy value
raw mean
inflection points
outer niche
central niche
Model
V
IV
III
II
I
Predicted probability
0
0.2
0.4
0.6
0.8
1
20
25
30
35
salinity

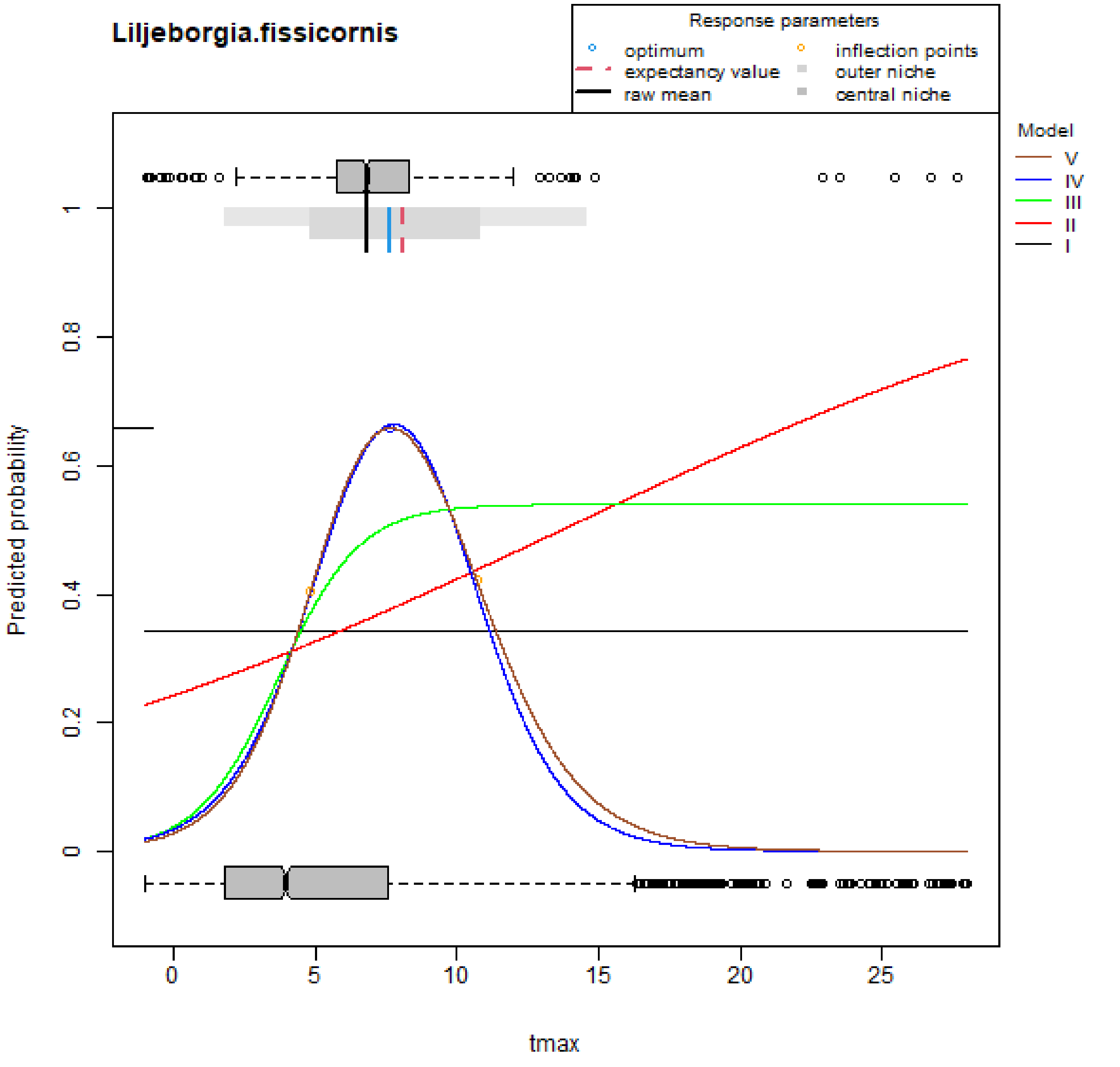

Liljeborgia.fissicornis
Response parameters
optimum
expectancy value
raw mean
inflection points
outer niche
central niche
Model
V
IV
III
II
I
Predicted probability
0
0.2
0.4
0.6
0.8
1
0
5
10
15
20
25
tmax

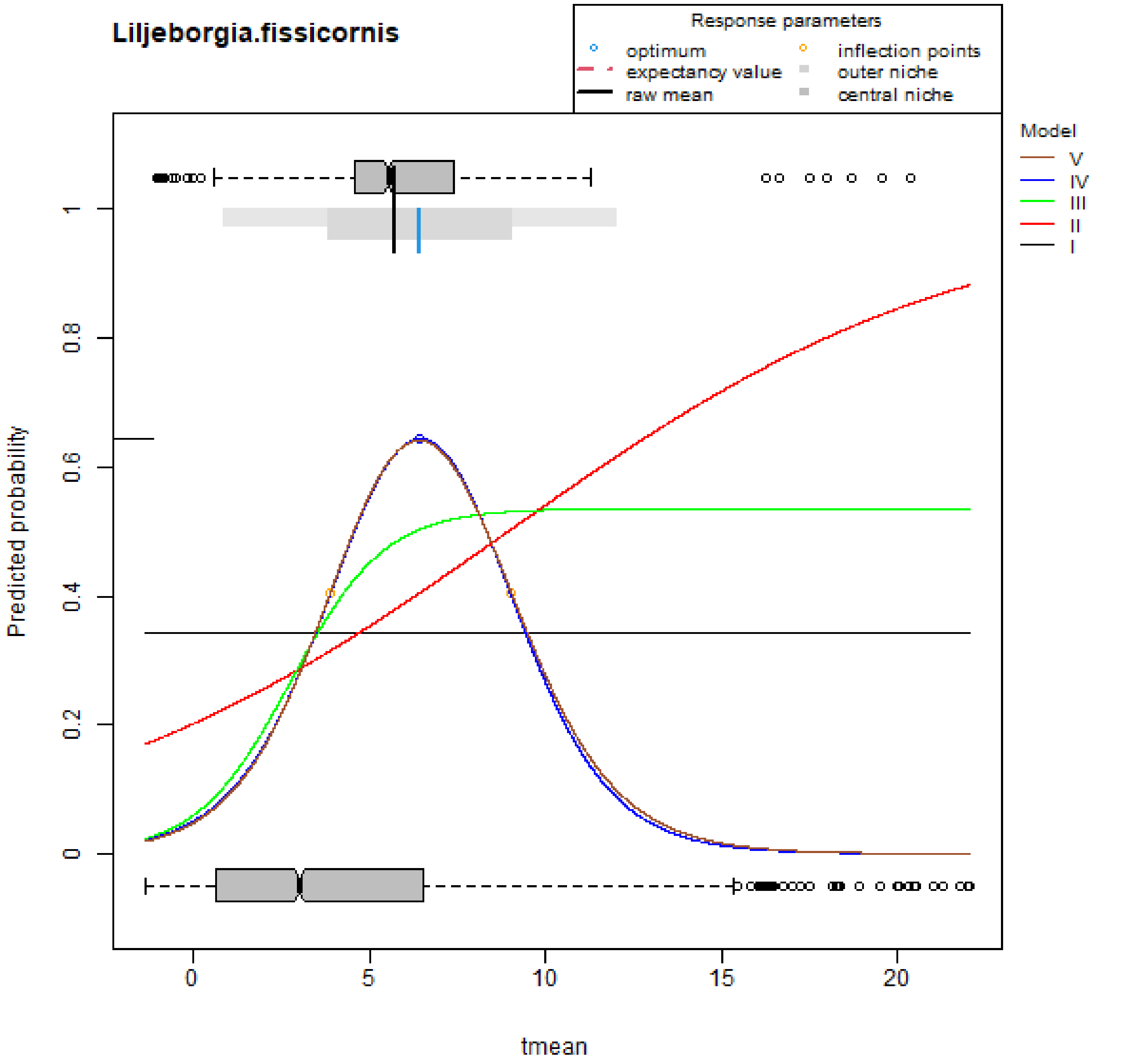

Liljeborgia.fissicornis
Response parameters
optimum
expectancy value
raw mean
inflection points
outer niche
central niche
Model
V
IV
III
II
I
Predicted probability
0
0.2
0.4
0.6
0.8
1
0
5
10
15
20
tmean

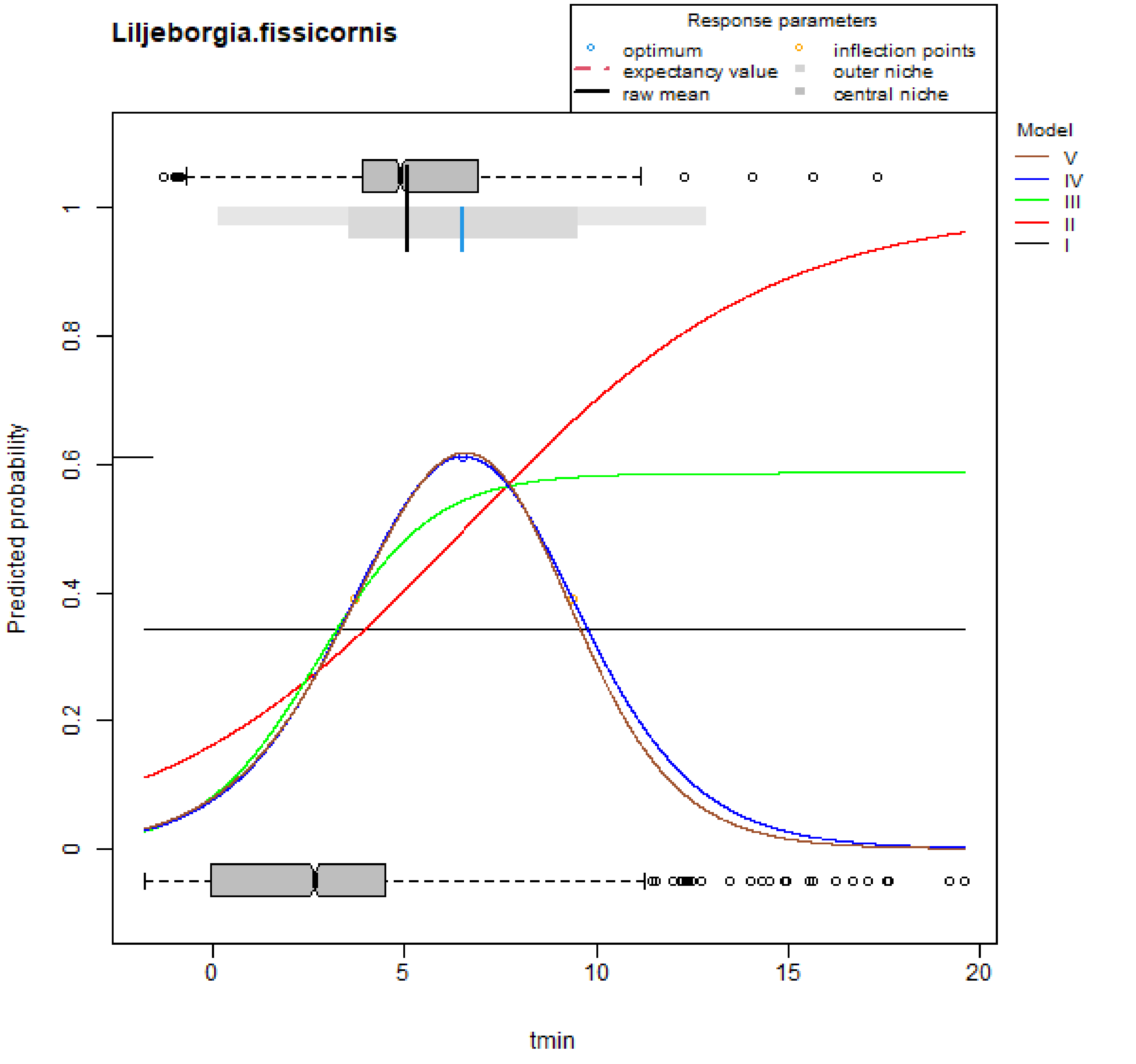

Liljeborgia.fissicornis
Response parameters
optimum
expectancy value
raw mean
inflection points
outer niche
central niche
Model
V
IV
III
II
I
Predicted probability
0
0.2
0.4
0.6
0.8
1
0
5
10
15
20
tmin

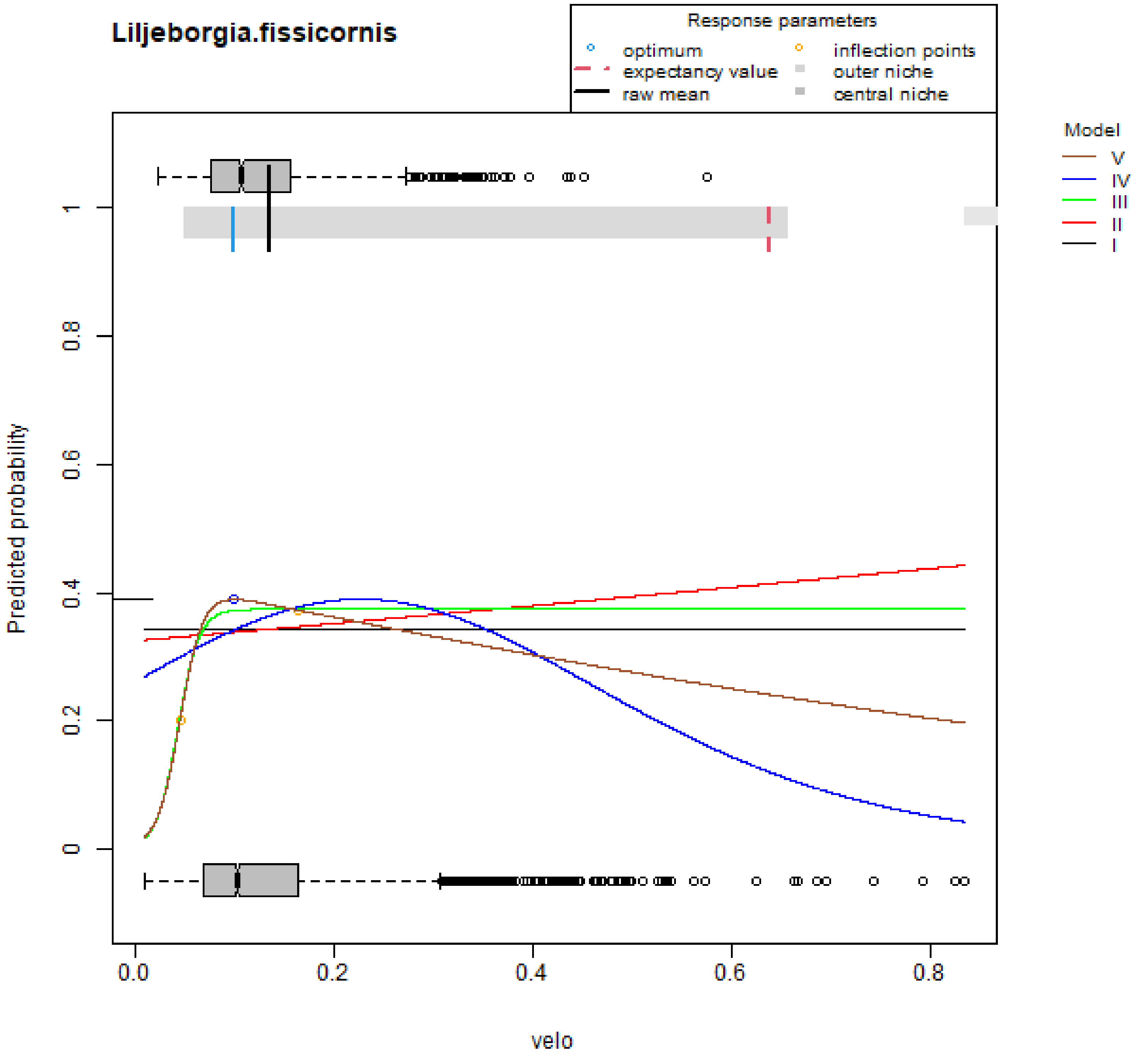

Liljeborgia.fissicornis
Response parameters
optimum
expectancy value
raw mean
inflection points
outer niche
central niche
Model
V
IV
III
II
I
Predicted probability
0
0.2
0.4
0.6
0.8
1
0.0
0.2
0.4
0.6
0.8
velo

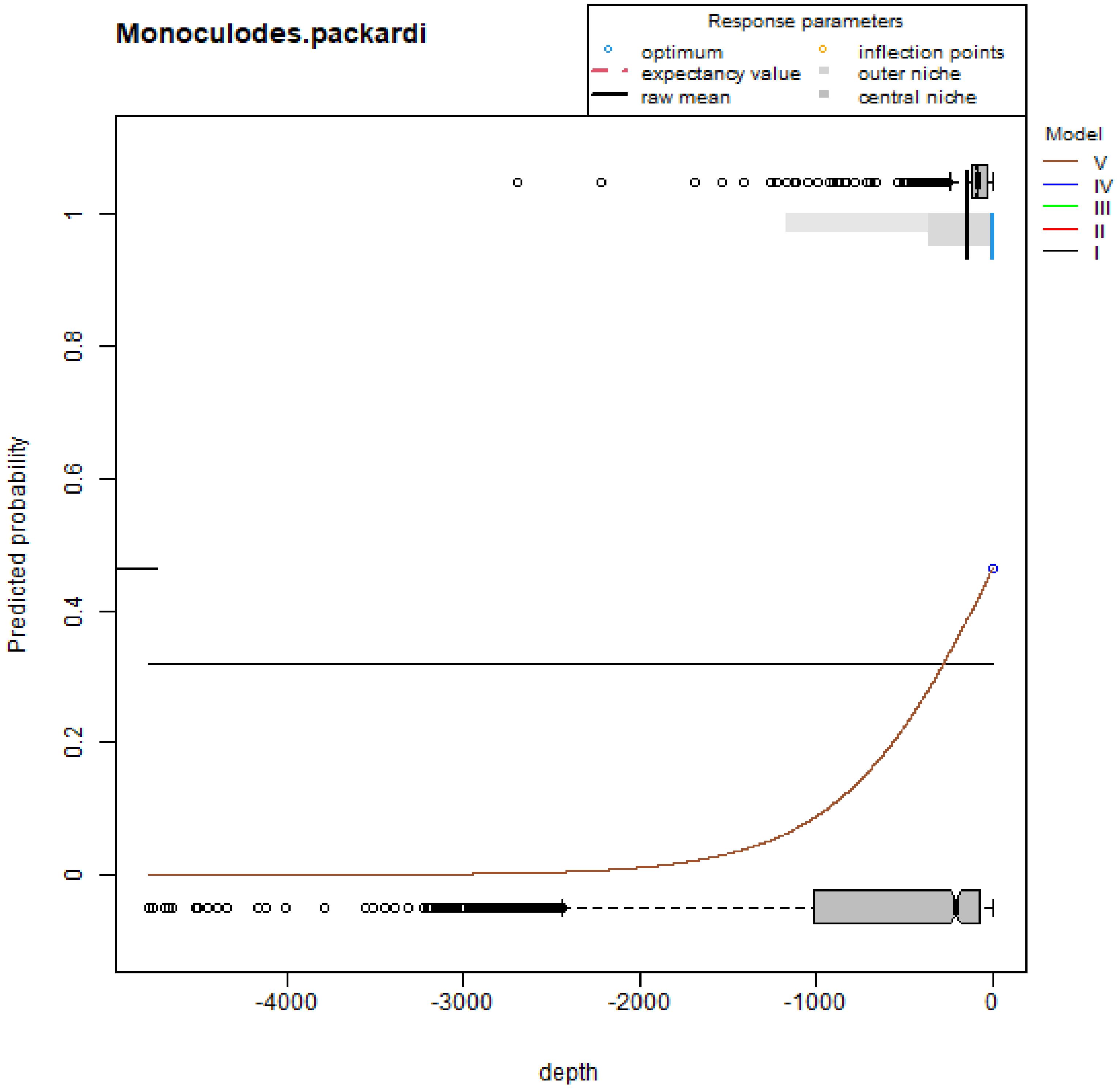
Monoculodes.packardi
Response parameters
optimum
expectancy value
raw mean
inflection points
outer niche
central niche
Model
V
IV
III
II
I
Predicted probability
0
0.2
0.4
0.6
0.8
1
-4000
-3000
-2000
-1000
0
depth

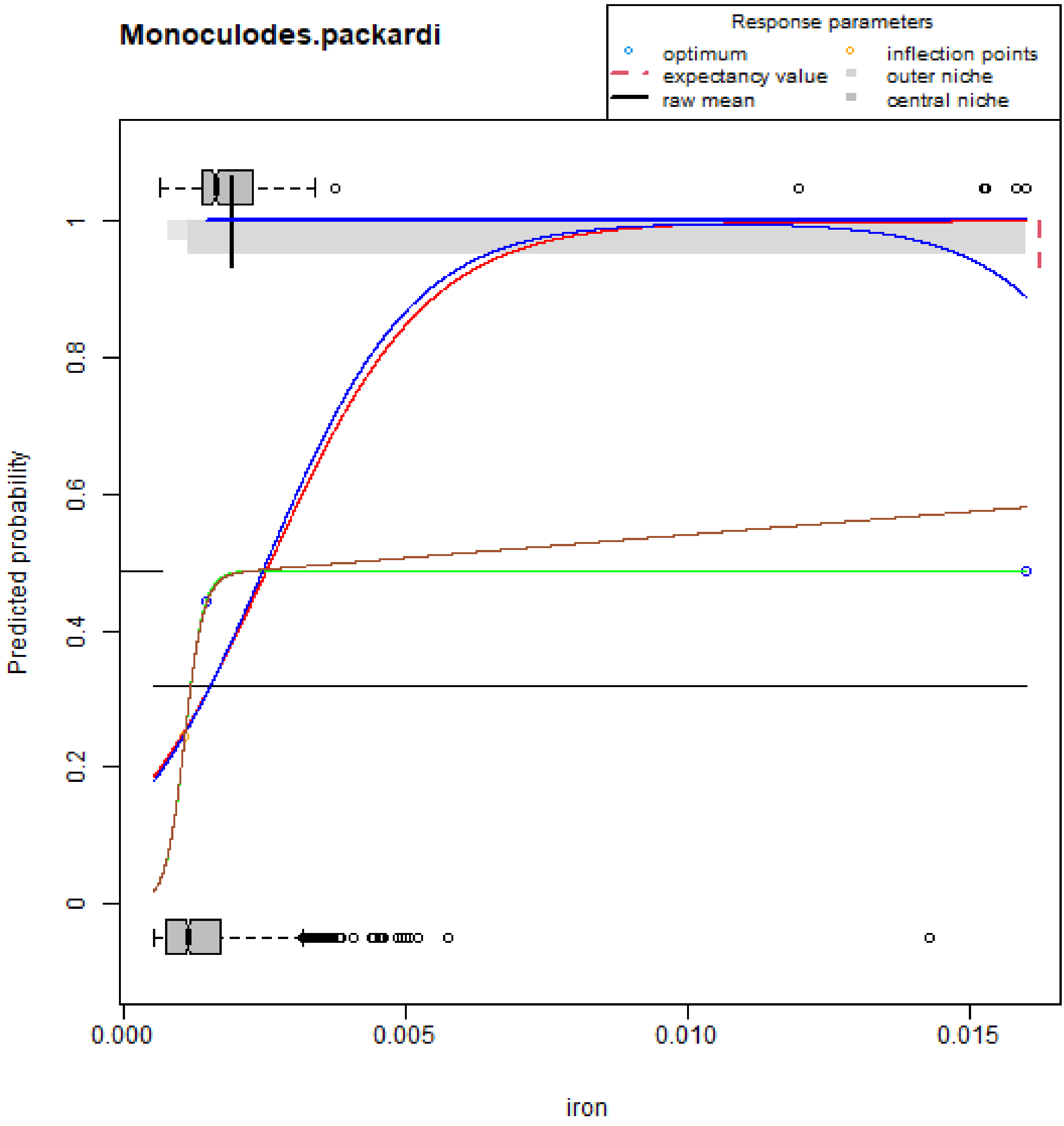

Monoculodes.packardi
Response parameters
optimum
expectancy value
raw mean
inflection points
outer niche
central niche
Predicted probability
0
0.2
0.4
0.6
0.8
1
0.000
0.005
0.010
0.015
iron

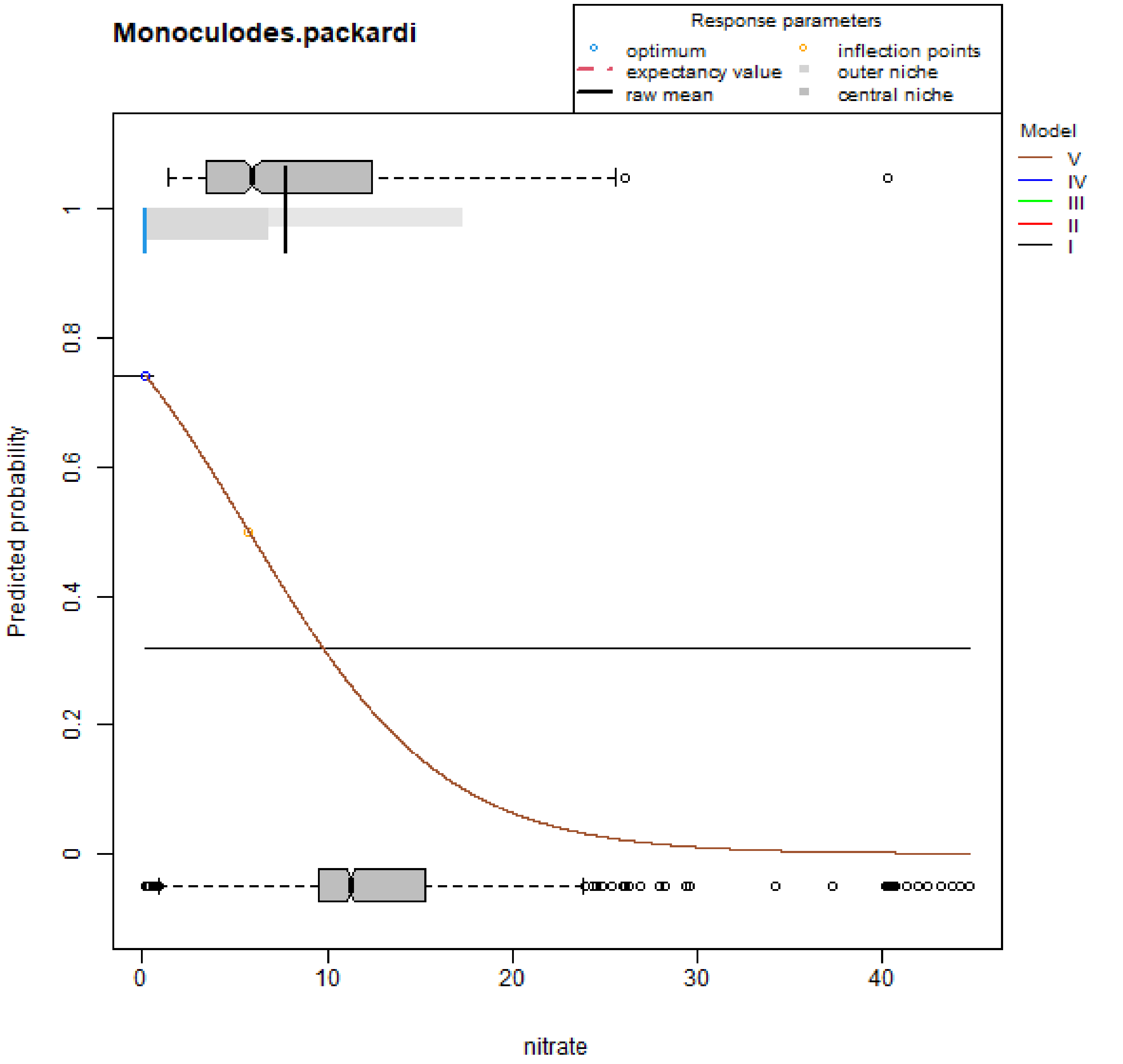
Monoculodes.packardi
Response parameters
optimum
expectancy value
raw mean
inflection points
outer niche
central niche
Model
V
IV
III
II
I
Predicted probability
0
0.2
0.4
0.6
0.8
1
0
10
20
30
40
nitrate

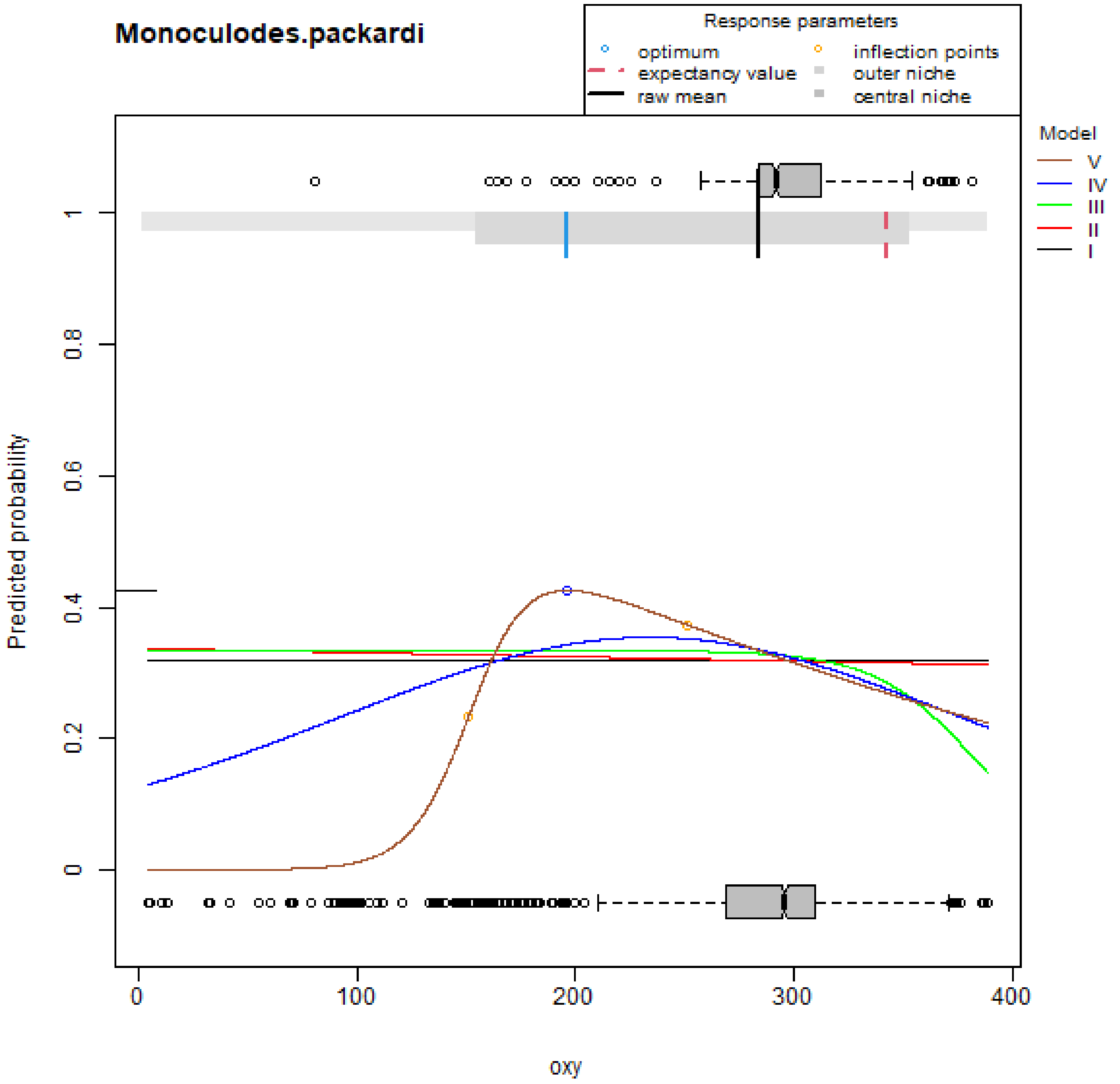

Monoculodes.packardi
Response parameters
optimum
expectancy value
raw mean
inflection points
outer niche
central niche
Model
V
IV
III
II
I
Predicted probability
0
0.2
0.4
0.6
0.8
1
0
100
200
300
400
oxy

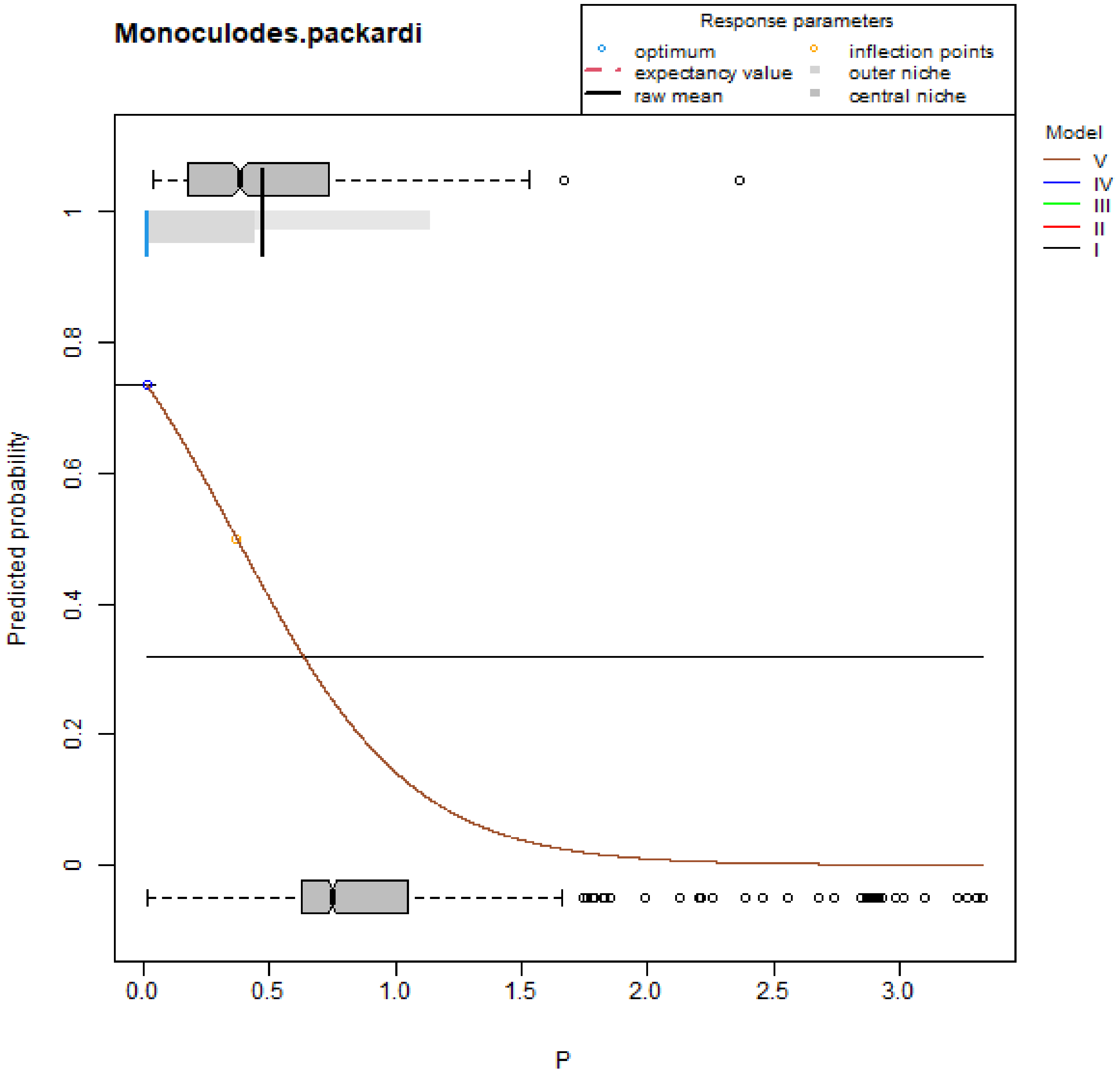

Monoculodes.packardi
Response parameters
optimum
expectancy value
raw mean
inflection points
outer niche
central niche
Model
V
IV
III
II
I
Predicted probability
0
0.2
0.4
0.6
0.8
1
0.0
0.5
1.0
1.5
2.0
2.5
3.0
P

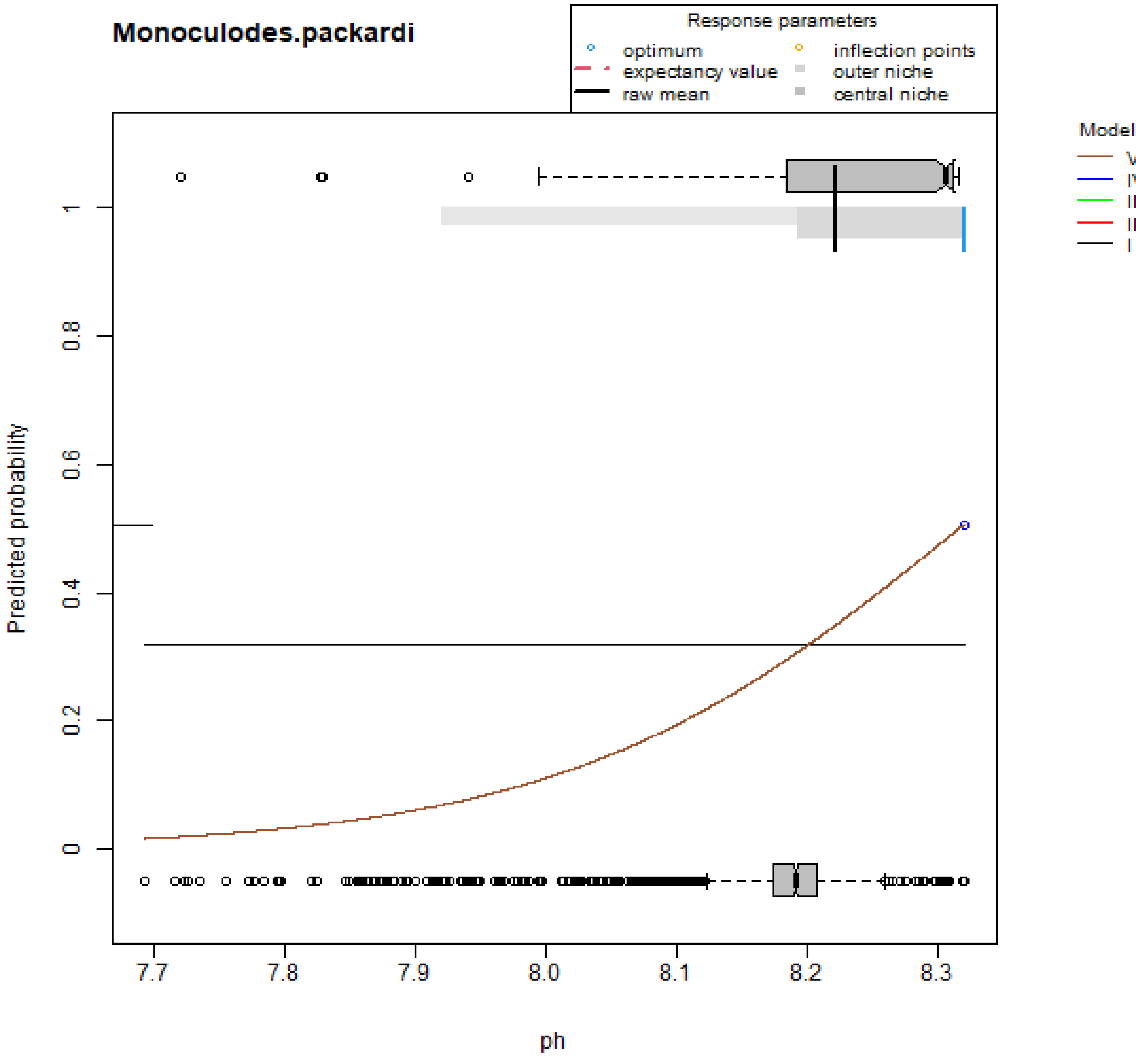

Monoculodes.packardi
Response parameters
optimum
inflection points
expectancy value
outer niche
raw mean
central niche
Model
Predicted probability
0
0.2
0.4
0.6
0.8
1
7.7
7.8
7.9
8.0
8.1
8.2
8.3
ph

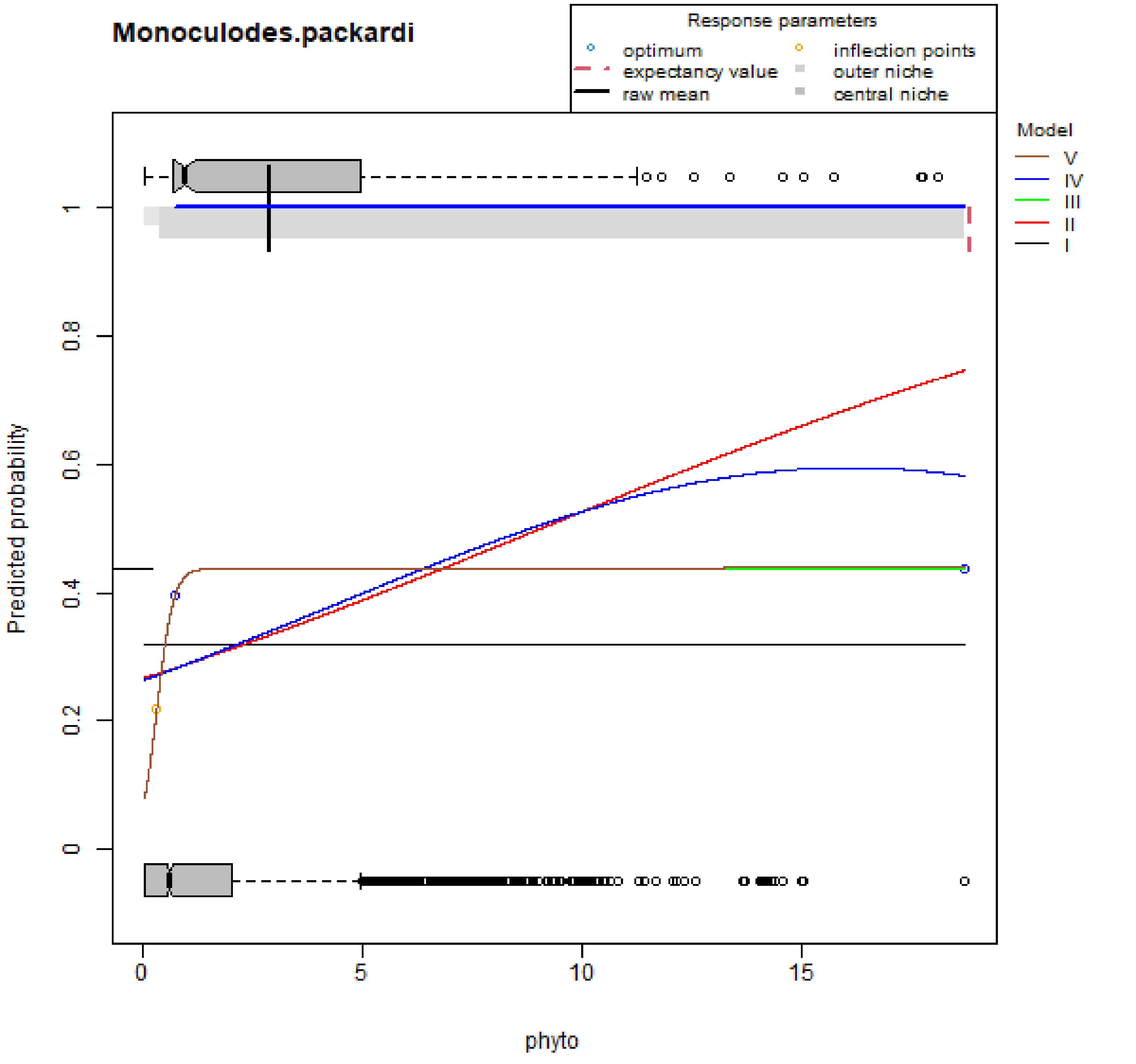

Monoculodes.packardi
Response parameters
optimum
inflection points
expectancy value
outer niche
raw mean
central niche
Model
V
IV
III
II
I
Predicted probability
0
0.2
0.4
0.6
0.8
1
0
5
10
15
phyto

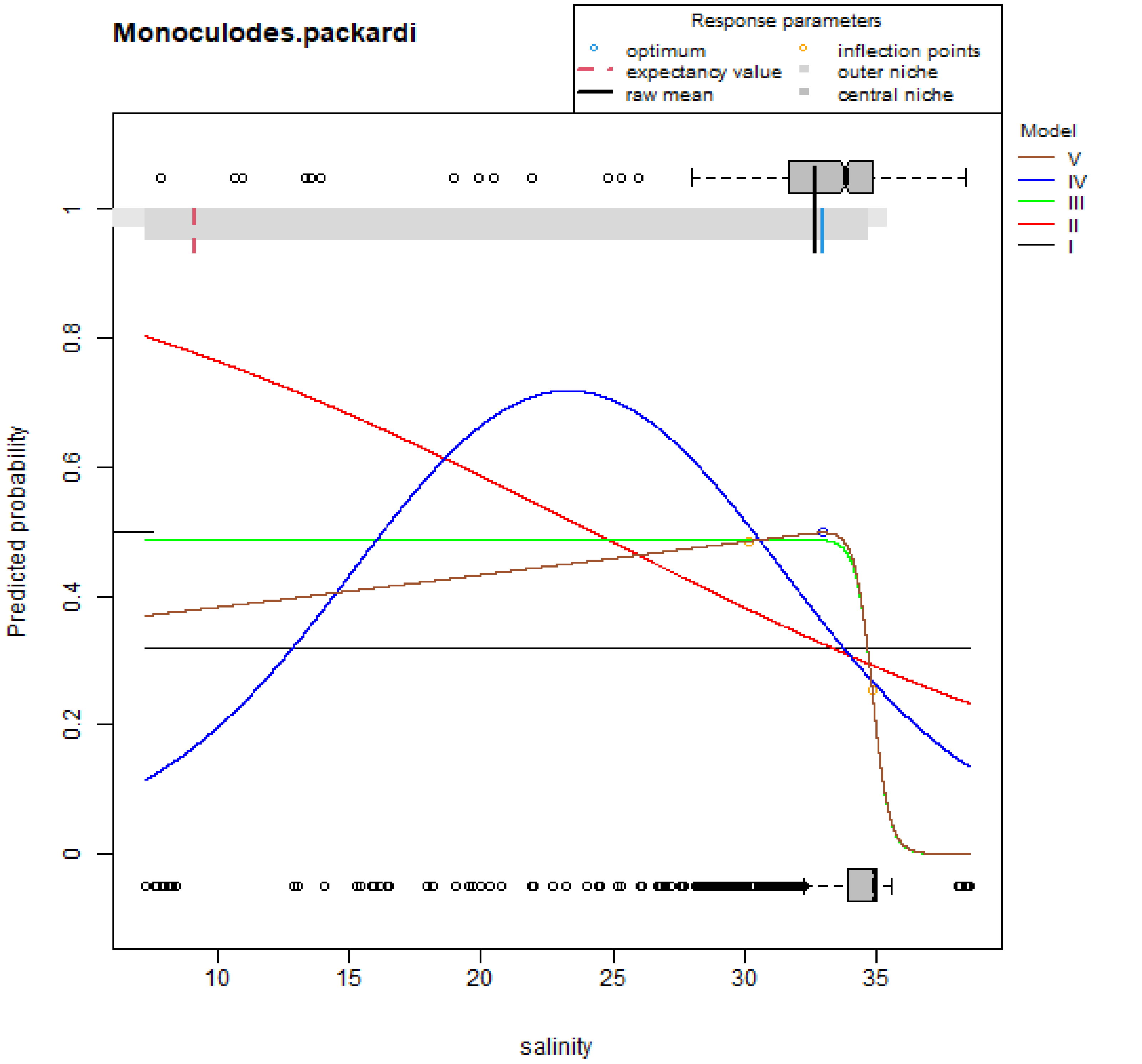

Monoculodes.packardi
Response parameters
optimum
expectancy value
raw mean
inflection points
outer niche
central niche
Model
V
IV
III
II
I
Predicted probability
0
0.2
0.4
0.6
0.8
1
10
15
20
25
30
35
salinity

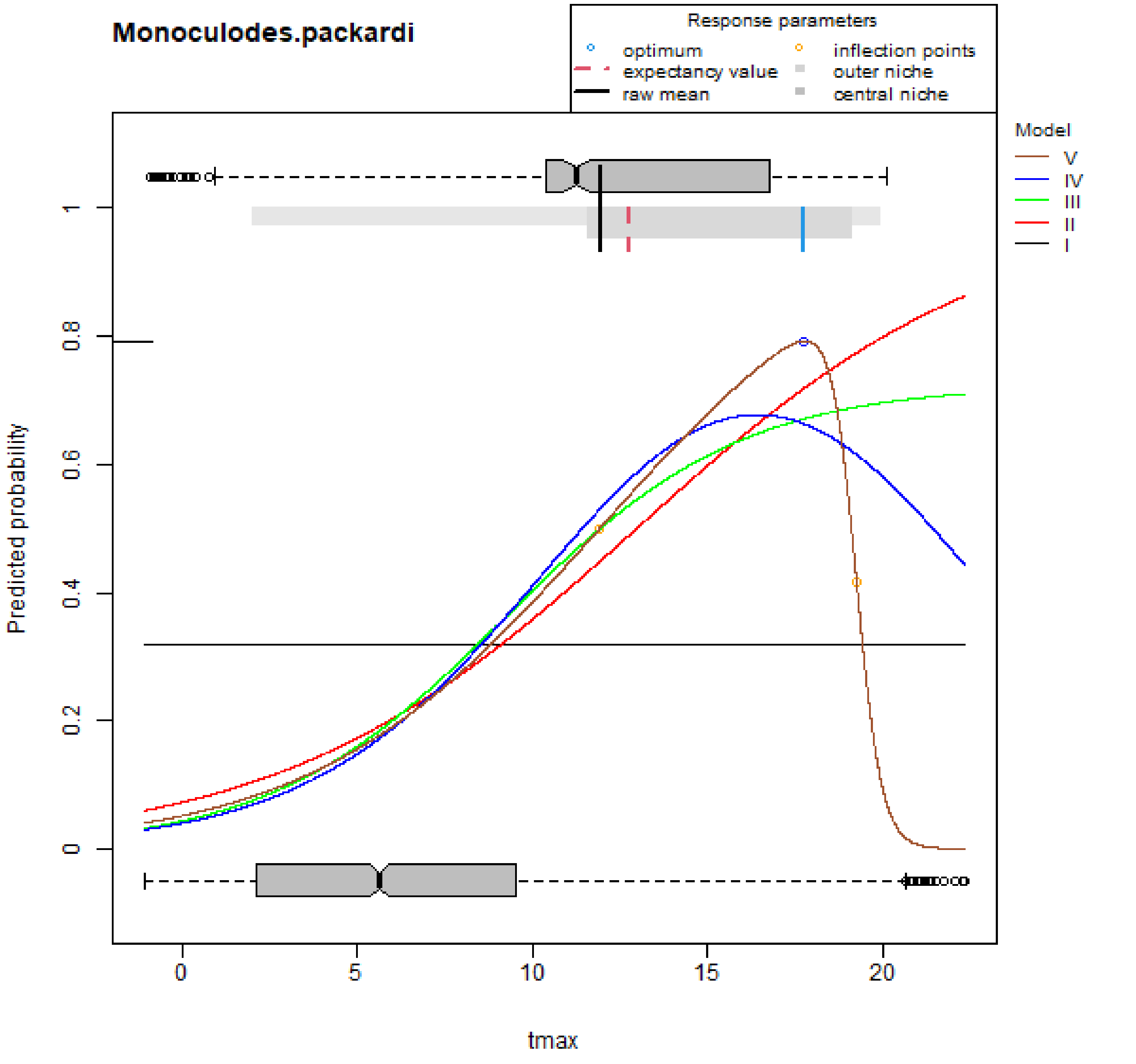

Monoculodes.packardi
Response parameters
optimum
expectancy value
raw mean
inflection points
outer niche
central niche
Model
V
IV
III
II
I
Predicted probability
0
0.2
0.4
0.6
0.8
1
0
5
10
15
20
tmax

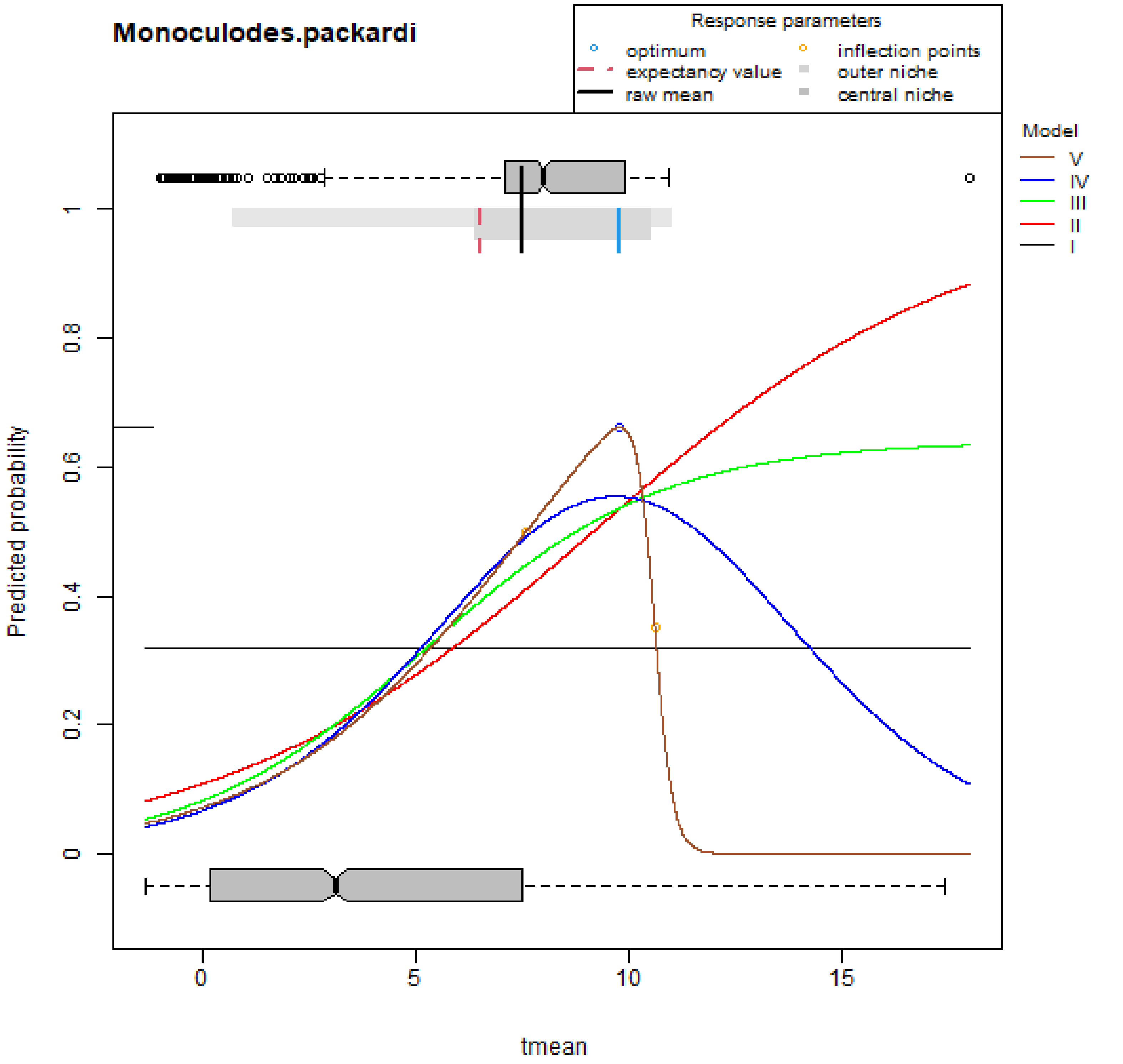
Monoculodes.packardi
Response parameters
optimum
expectancy value
raw mean
inflection points
outer niche
central niche
Model
V
IV
III
II
I
Predicted probability
0
0.2
0.4
0.6
0.8
1
0
5
10
15
tmean

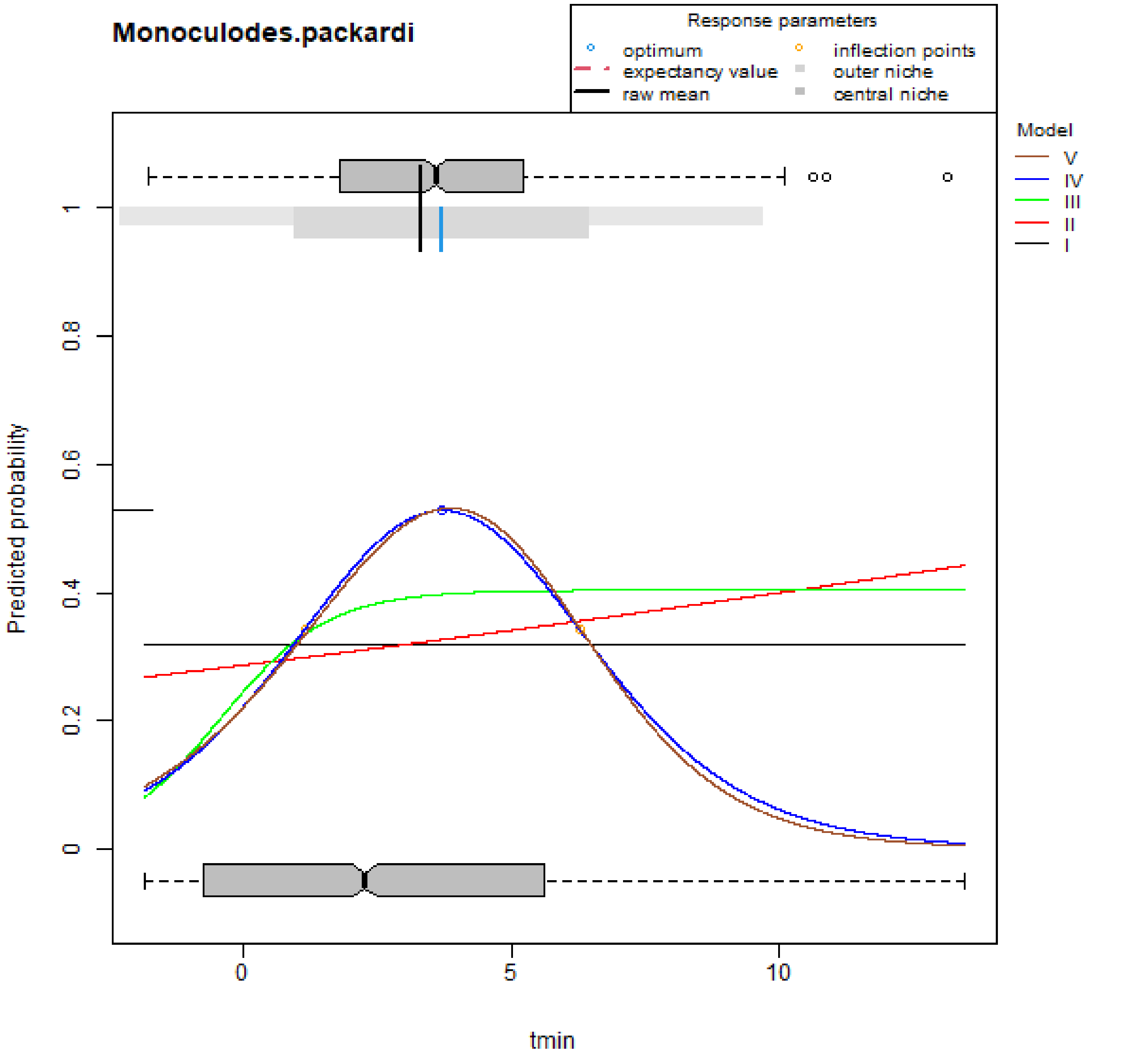

Monoculodes.packardi
Response parameters
optimum
expectancy value
raw mean
inflection points
outer niche
central niche
Model
V
IV
III
II
I
Predicted probability
0
0.2
0.4
0.6
0.8
1
0
5
10
tmin

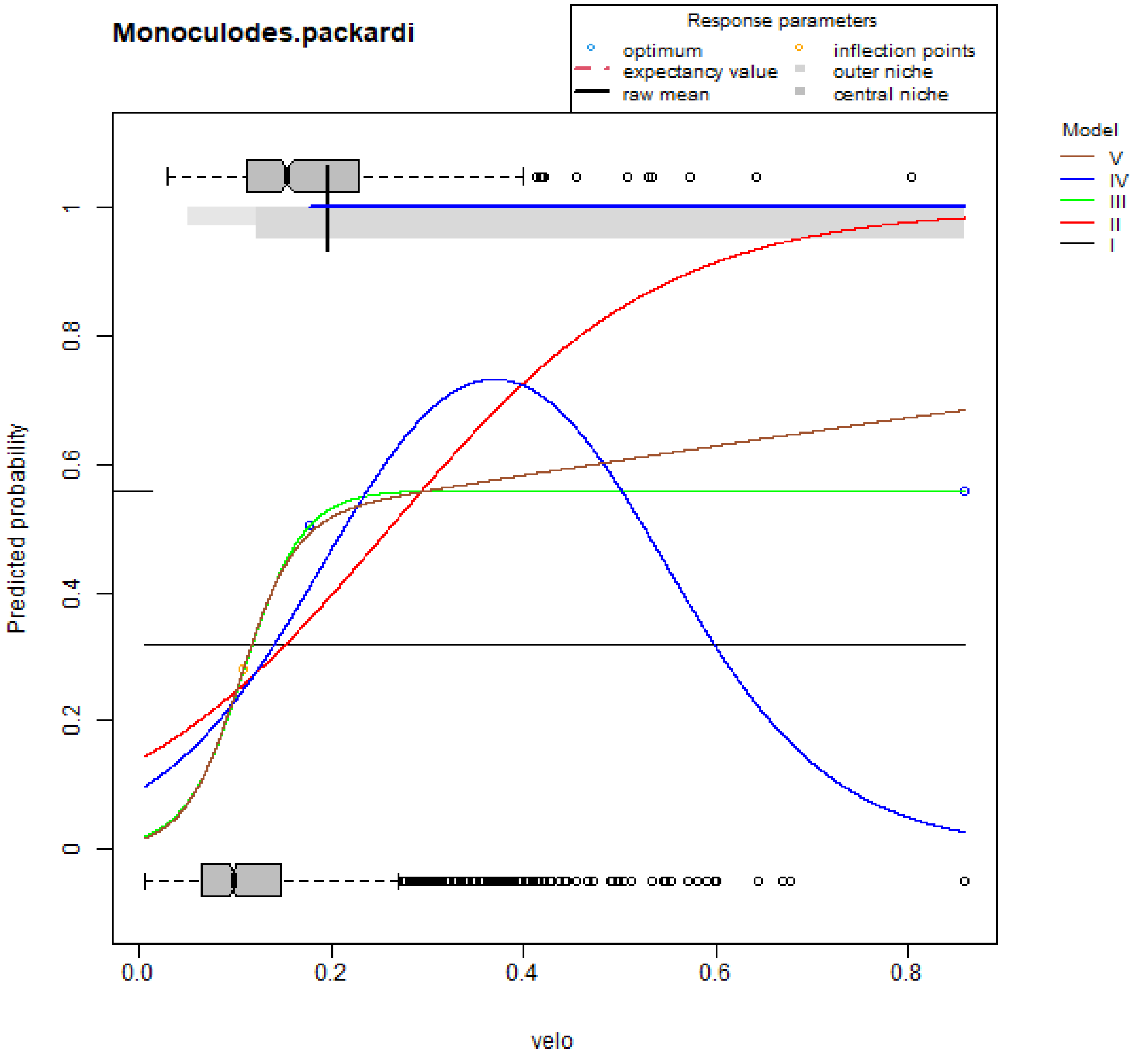

Monoculodes.packardi
Response parameters
optimum
expectancy value
raw mean
inflection points
outer niche
central niche
Model
V
IV
III
II
I
Predicted probability
0
0.2
0.4
0.6
0.8
1
0.0
0.2
0.4
0.6
0.8
velo

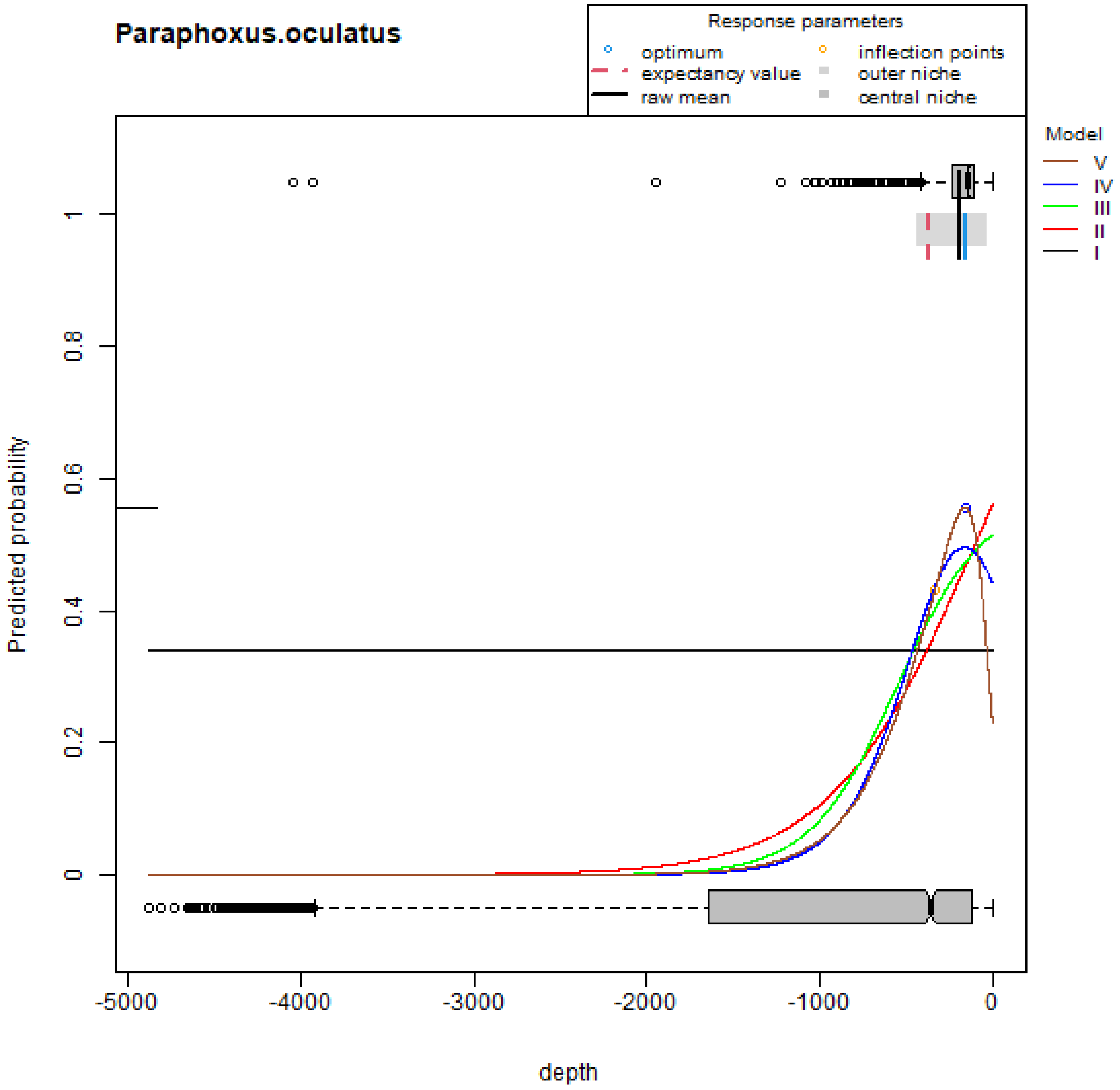

Paraphoxus.oculatus
Response parameters
optimum
expectancy value
raw mean
inflection points
outer niche
central niche
Model
V
IV
III
II
I
Predicted probability
0
0.2
0.4
0.6
0.8
1
-5000
-4000
-3000
-2000
-1000
0
depth

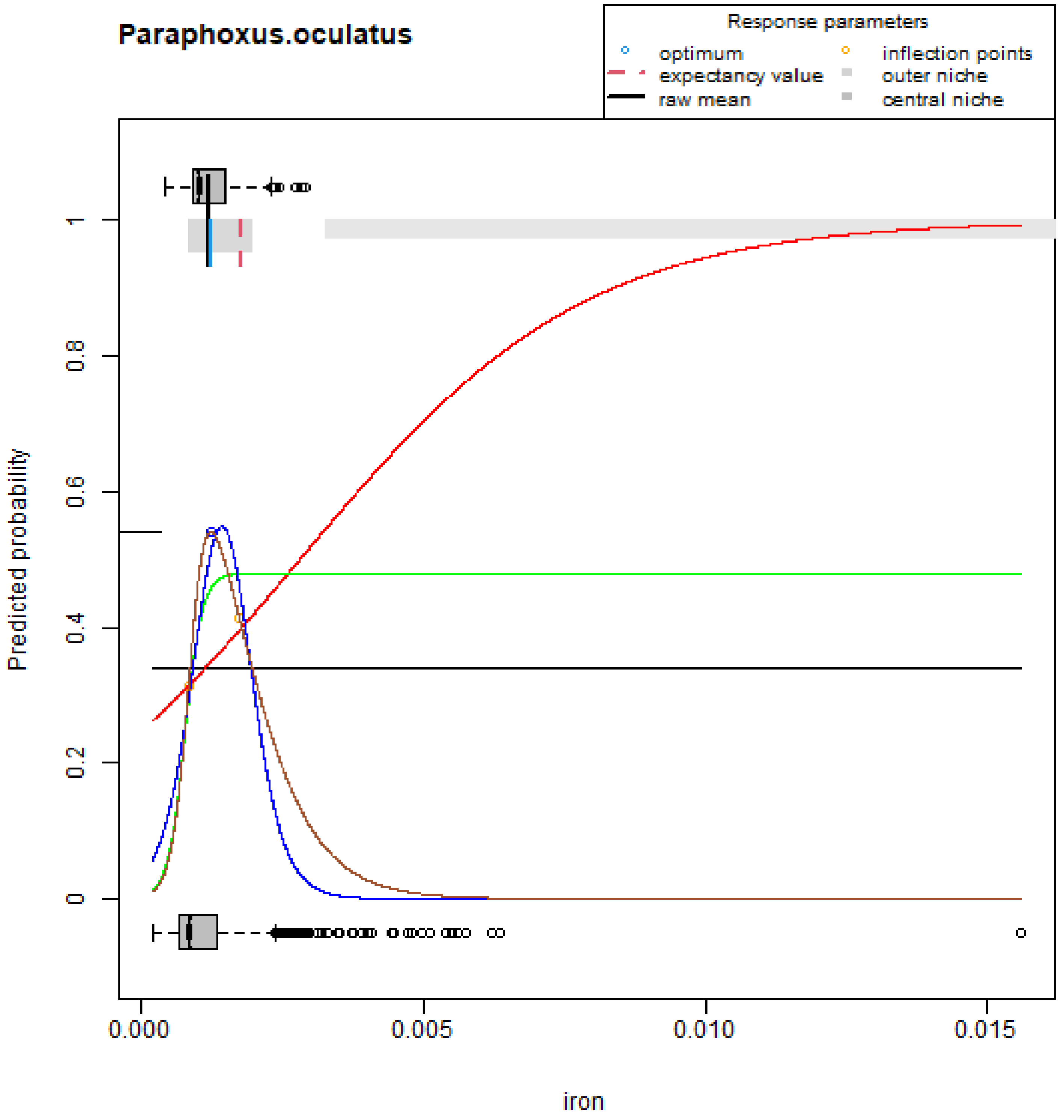

Paraphoxus.oculatus
Response parameters
optimum
expectancy value
raw mean
inflection points
outer niche
central niche
Predicted probability
0
0.2
0.4
0.6
0.8
1
0.000
0.005
0.010
0.015
iron

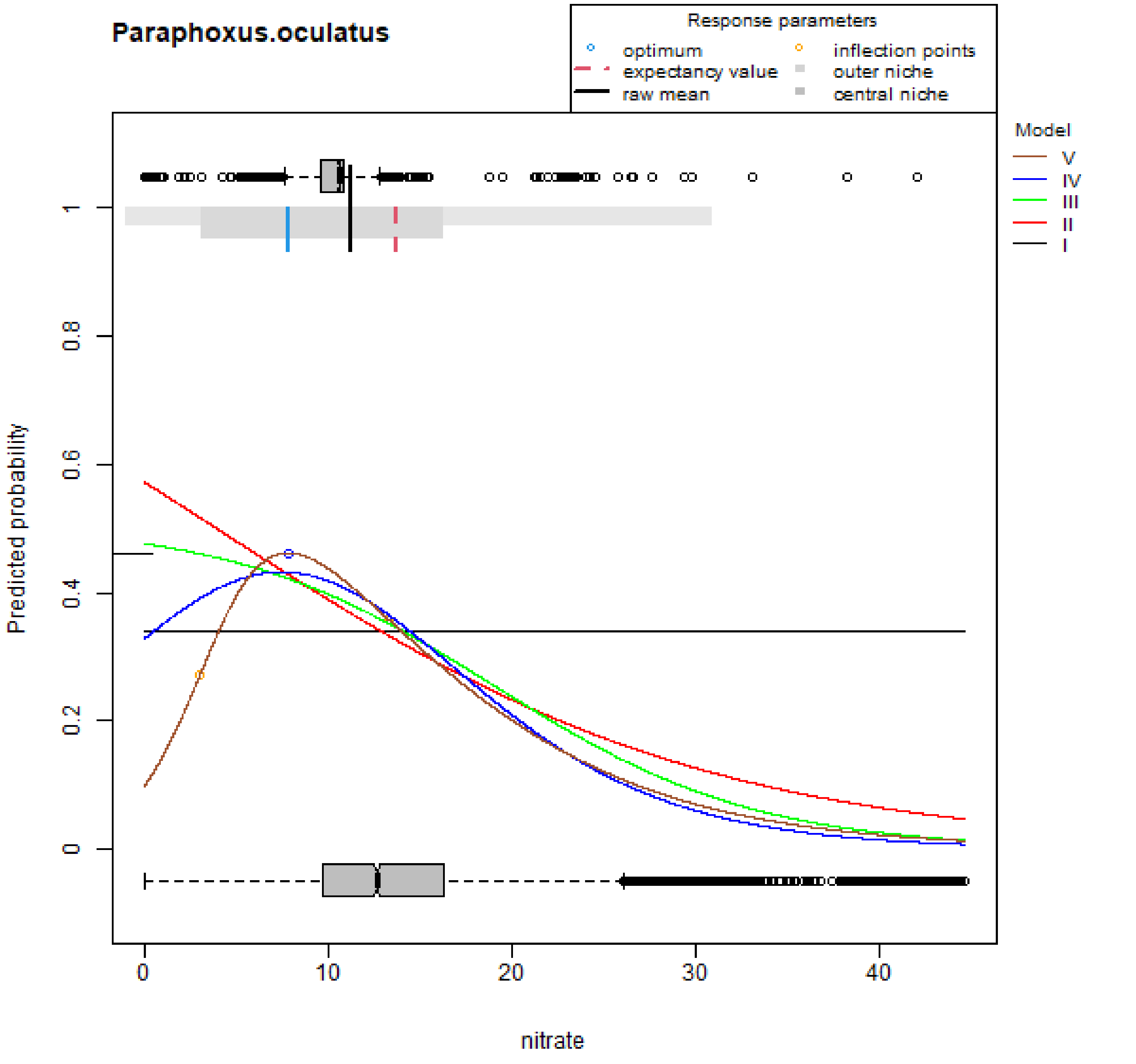

Paraphoxus.oculatus
Response parameters
optimum
expectancy value
raw mean
inflection points
outer niche
central niche
Model
V
IV
III
II
I
Predicted probability
0
0.2
0.4
0.6
0.8
1
0
10
20
30
40
nitrate

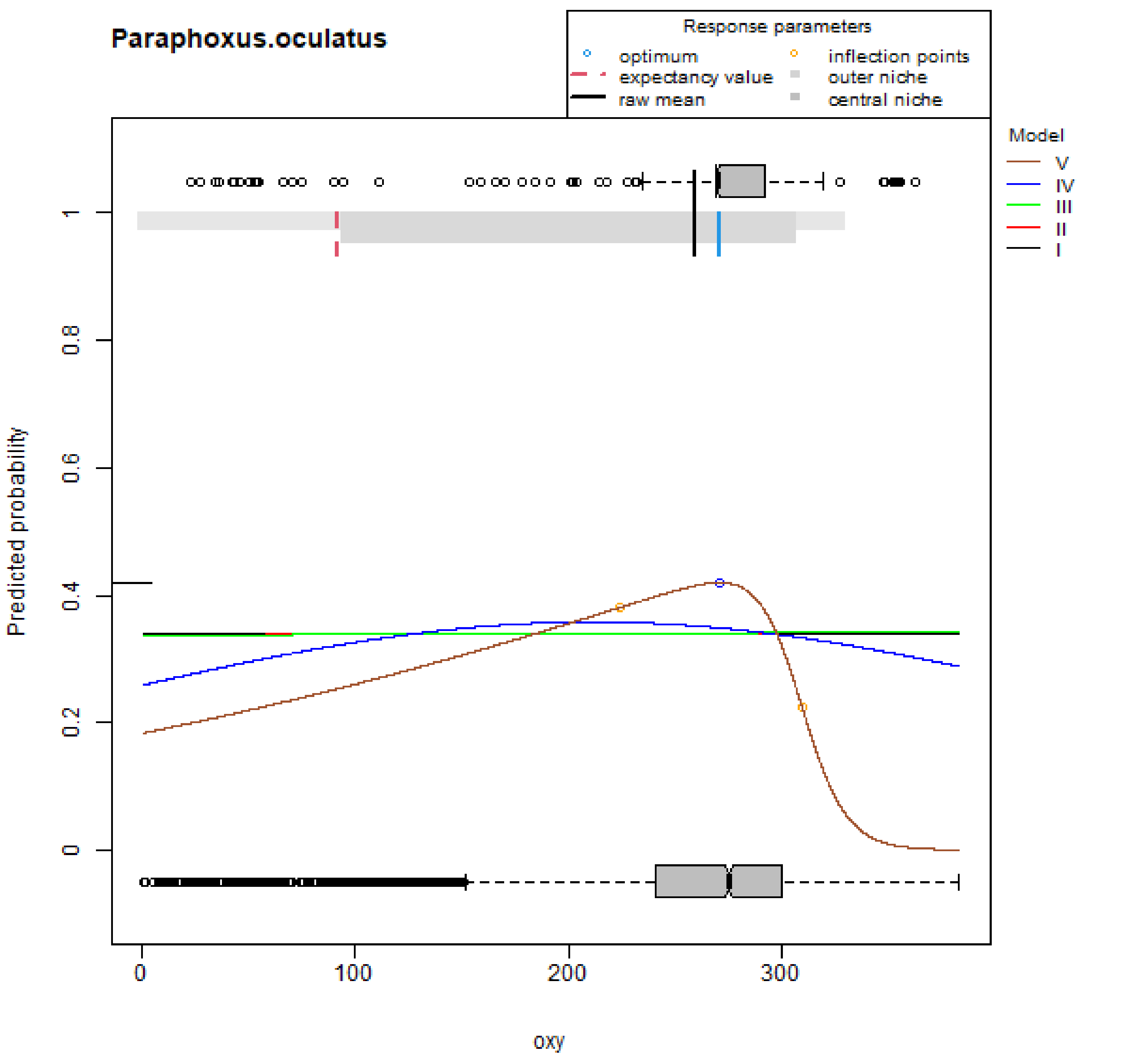

Paraphoxus.oculatus
Response parameters
optimum
expectancy value
raw mean
inflection points
outer niche
central niche
Model
V
IV
III
II
I
Predicted probability
0
0.2
0.4
0.6
0.8
1
0
100
200
300
oxy

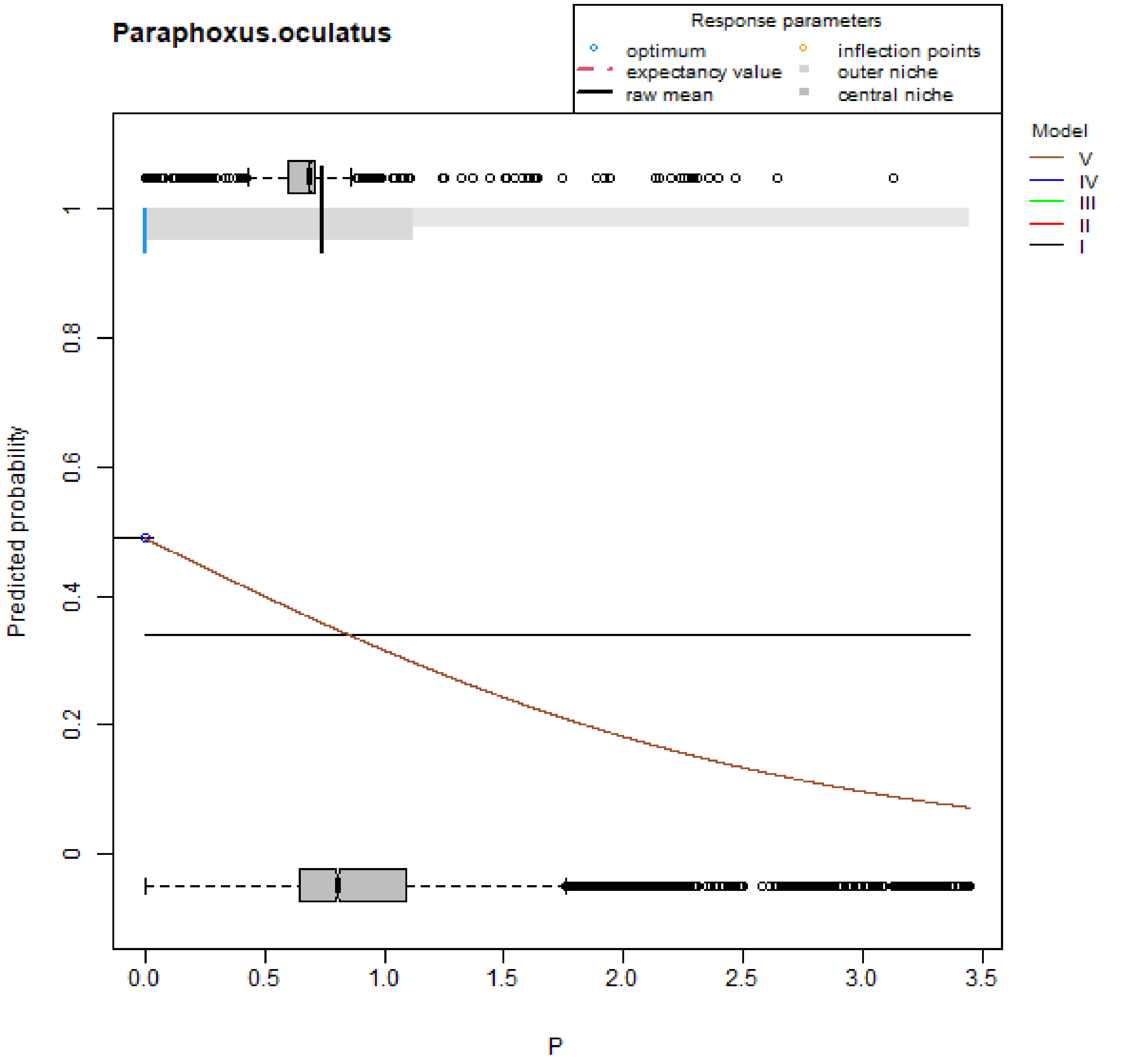
Paraphoxus.oculatus
Response parameters
optimum
expectancy value
raw mean
inflection points
outer niche
central niche
Model
V
IV
III
II
I
Predicted probability
0
0.2
0.4
0.6
0.8
1
0.0
0.5
1.0
1.5
2.0
2.5
3.0
3.5
P

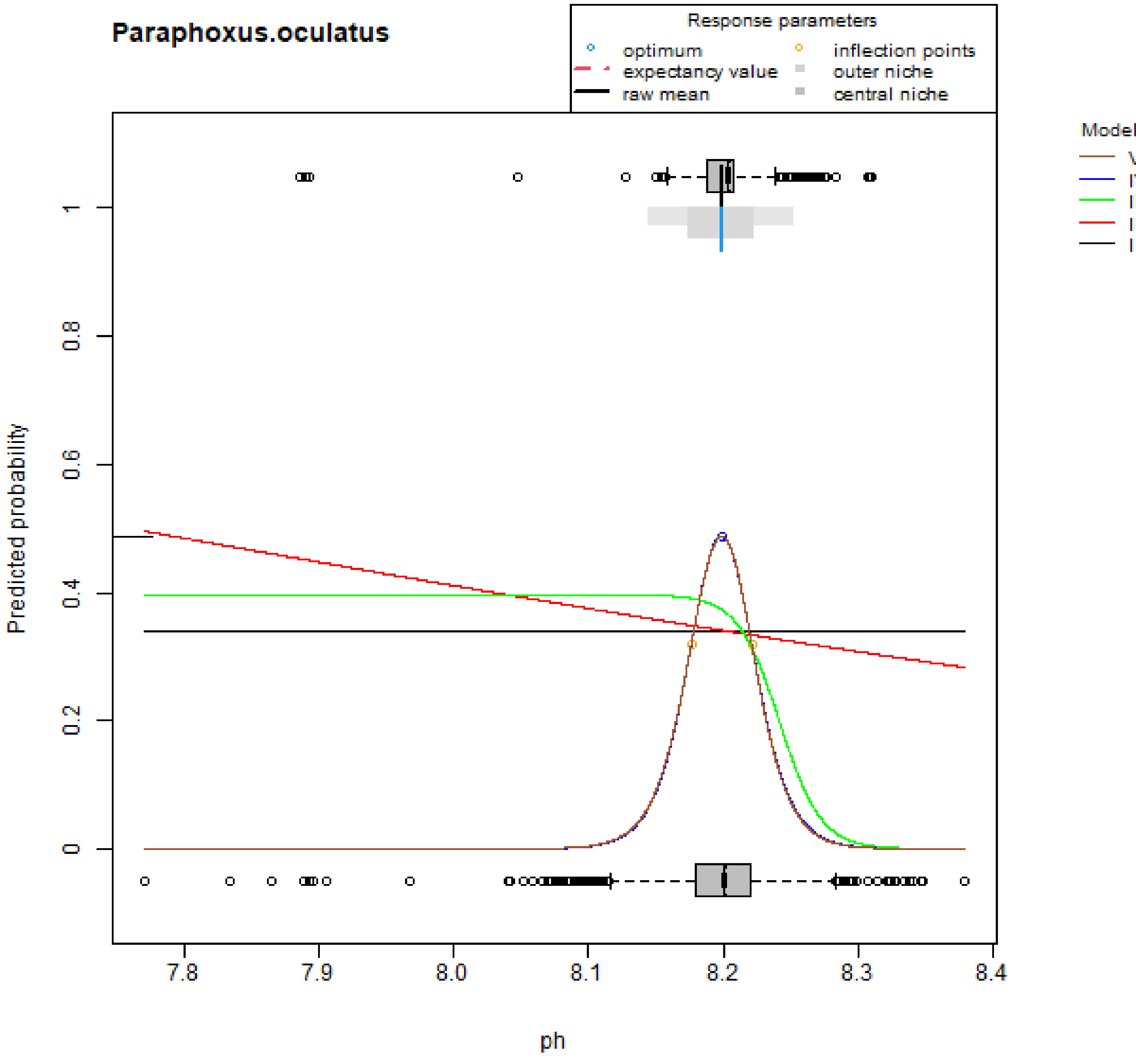

Paraphoxus.oculatus
Response parameters
optimum
expectancy value
raw mean
inflection points
outer niche
central niche
Model
Predicted probability
0
0.2
0.4
0.6
0.8
1
7.8
7.9
8.0
8.1
8.2
8.3
8.4
ph

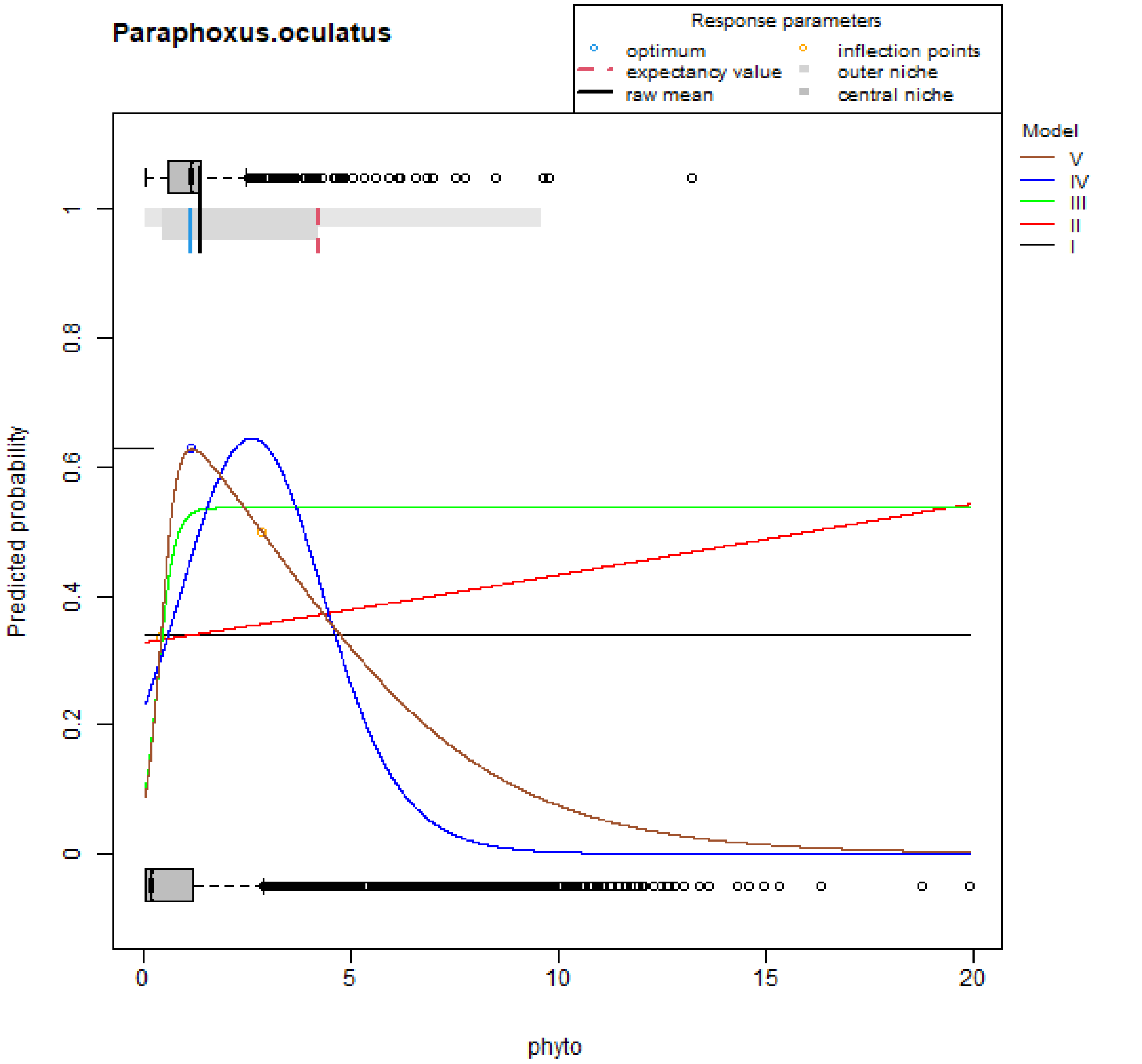
Paraphoxus.oculatus
Response parameters
optimum
expectancy value
raw mean
inflection points
outer niche
central niche
Model
V
IV
III
II
I
Predicted probability
0
0.2
0.4
0.6
0.8
1
0
5
10
15
20
phyto

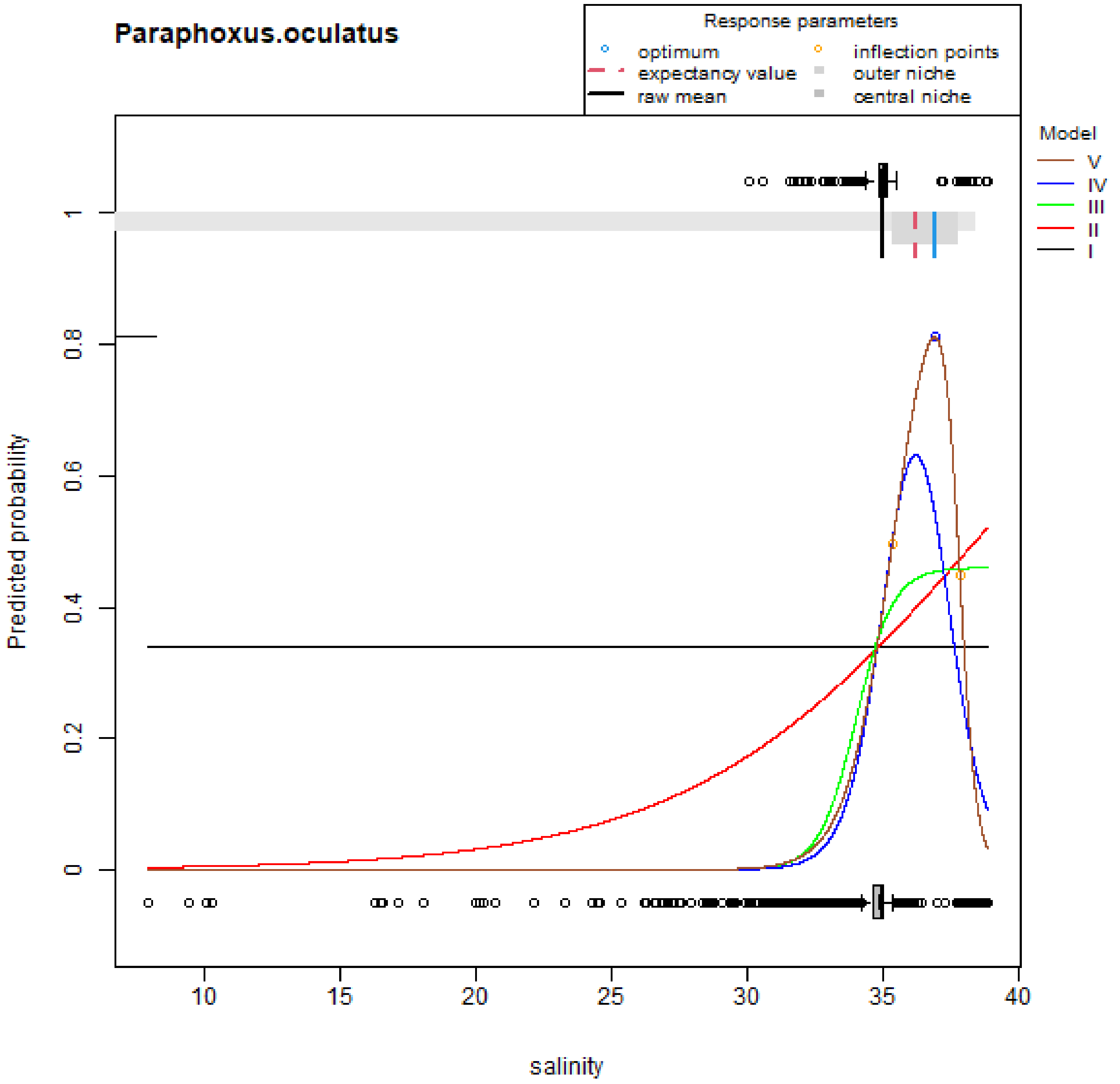

Paraphoxus.oculatus
Response parameters
optimum
expectancy value
raw mean
inflection points
outer niche
central niche
Model
V
IV
III
II
I
Predicted probability
0
0.2
0.4
0.6
0.8
1
10
15
20
25
30
35
40
salinity

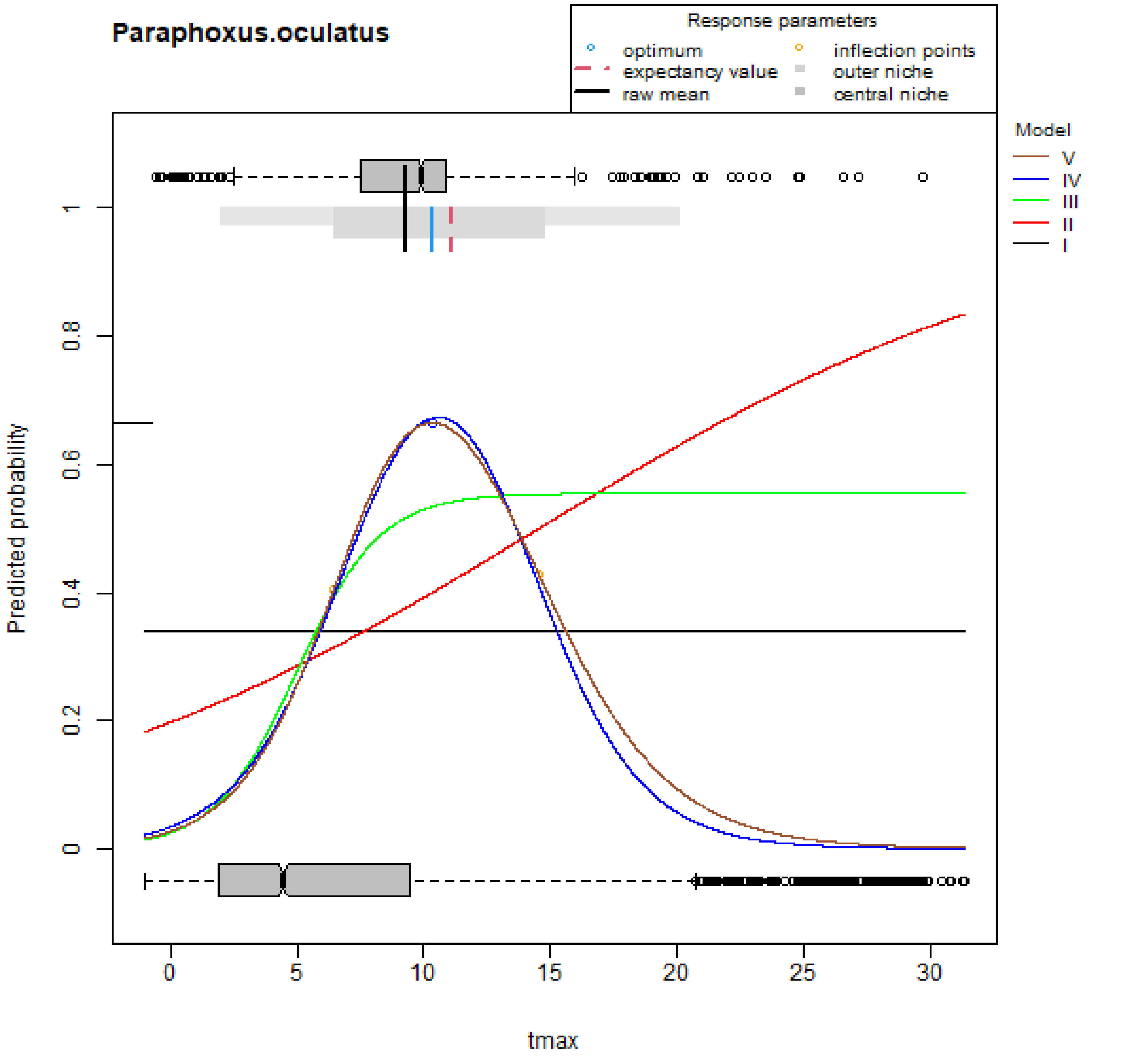

Paraphoxus.oculatus
Response parameters
optimum
expectancy value
raw mean
inflection points
outer niche
central niche
Model
V
IV
III
II
I
Predicted probability
0
0.2
0.4
0.6
0.8
1
0
5
10
15
20
25
30
tmax

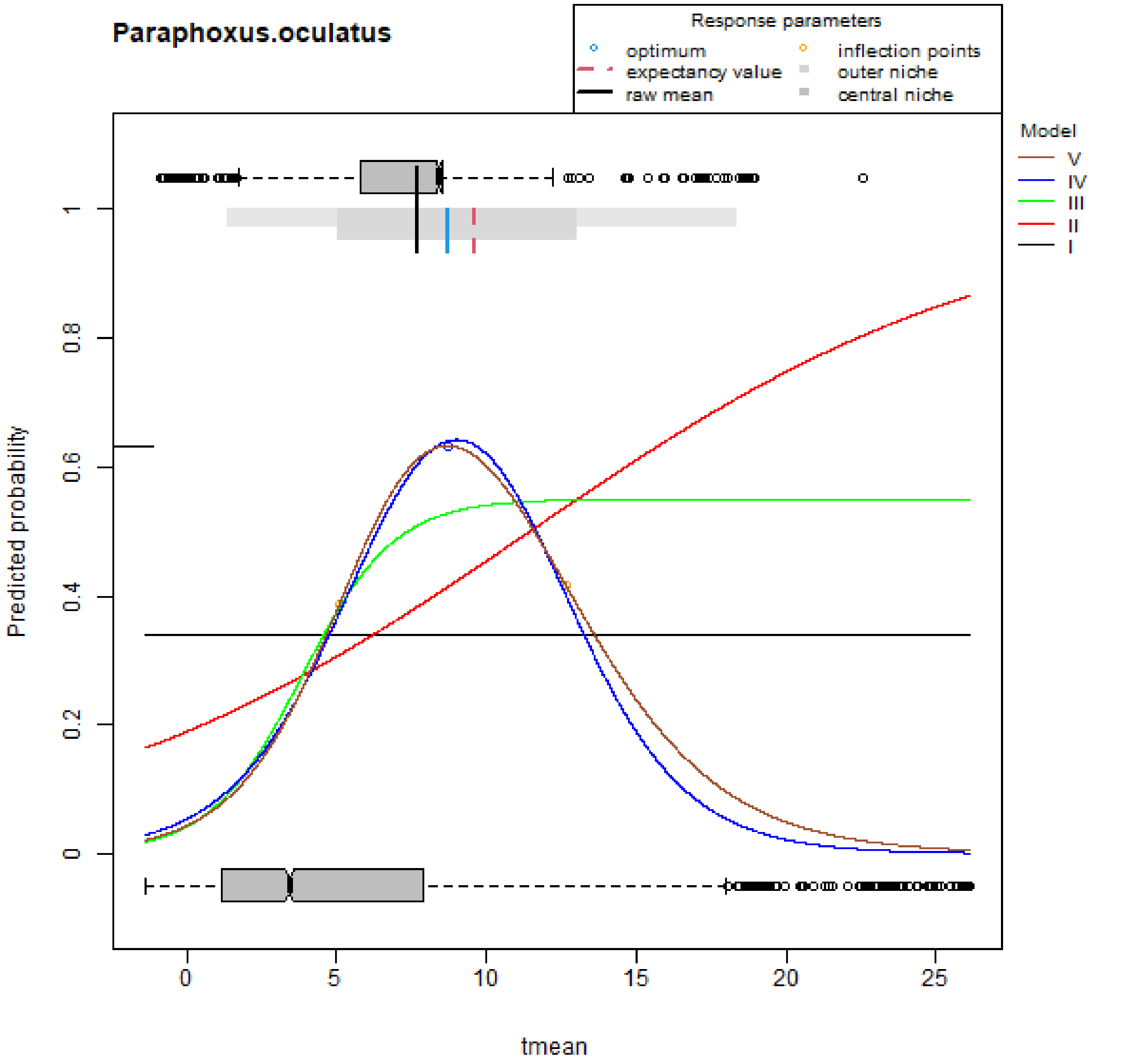

Paraphoxus.oculatus
Response parameters
optimum
expectancy value
raw mean
inflection points
outer niche
central niche
Model
V
IV
III
II
I
Predicted probability
0
0.2
0.4
0.6
0.8
1
0
5
10
15
20
25
tmean

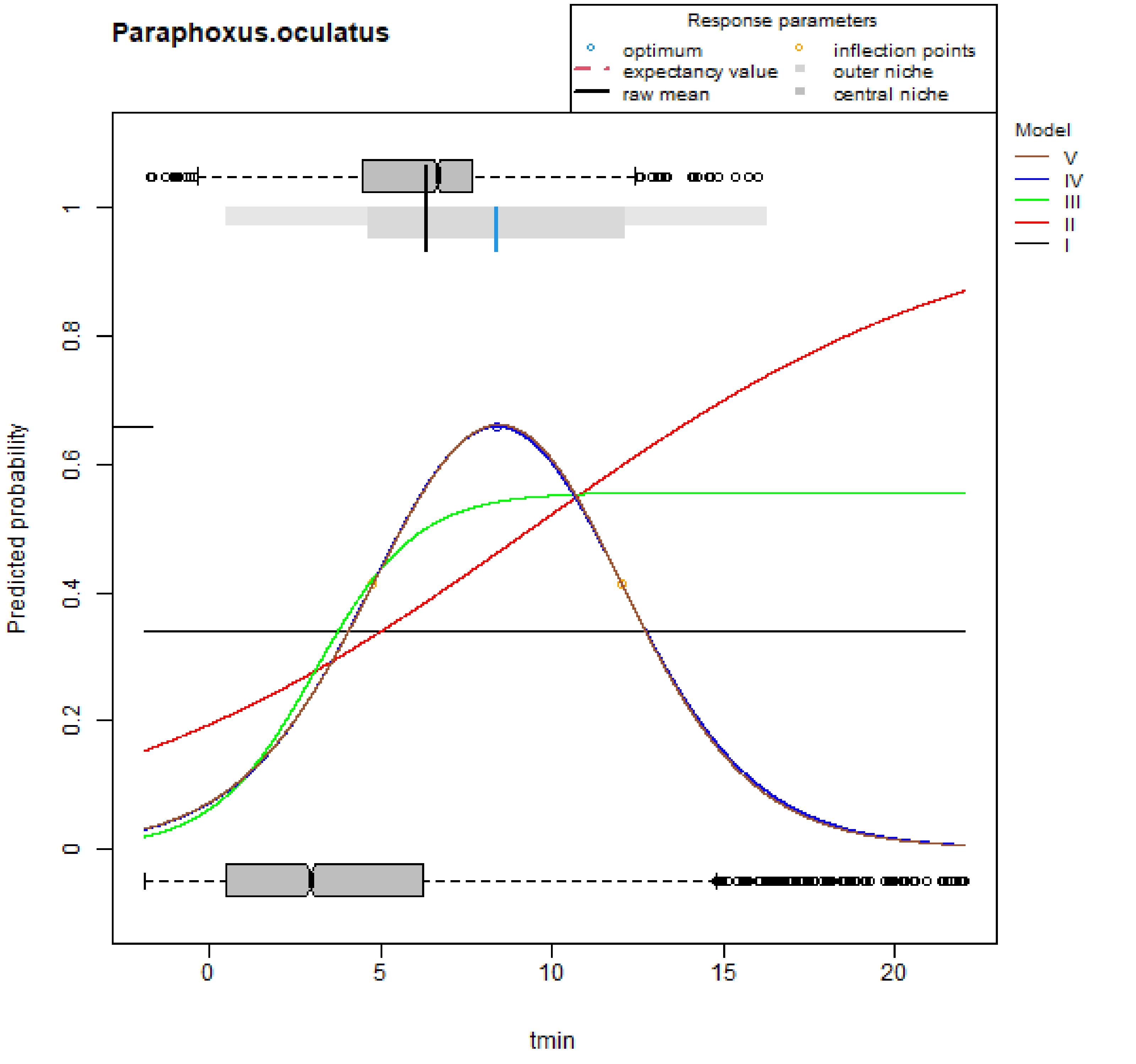

Paraphoxus.oculatus
Response parameters
optimum
expectancy value
raw mean
inflection points
outer niche
central niche
Model
V
IV
III
II
I
Predicted probability
0
0.2
0.4
0.6
0.8
1
0
5
10
15
20
tmin

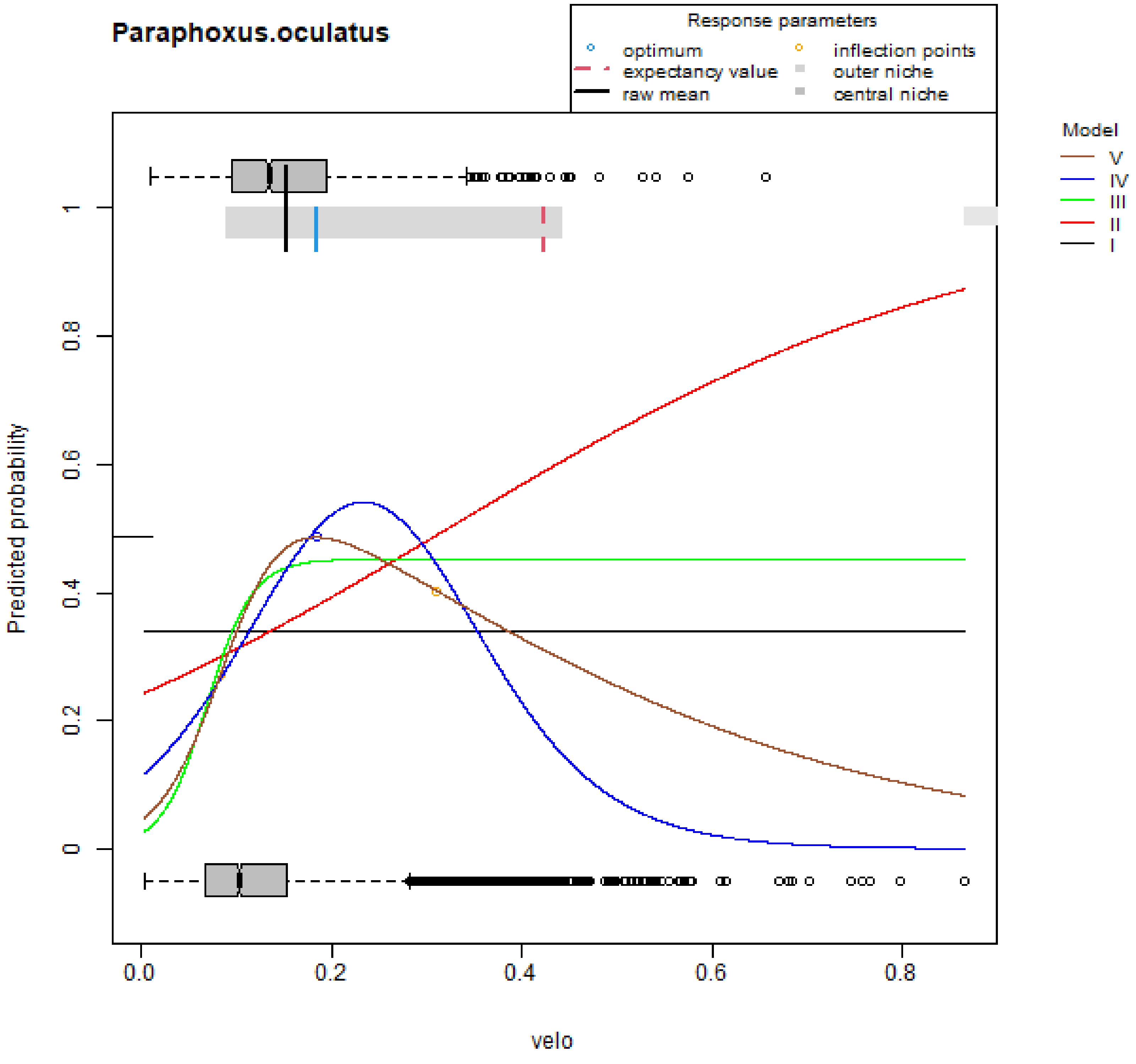

Paraphoxus.oculatus
Response parameters
optimum
inflection points
expectancy value
outer niche
raw mean
central niche
Model
V
IV
III
II
I
Predicted probability
0
0.2
0.4
0.6
0.8
1
0.0
0.2
0.4
0.6
0.8
velo

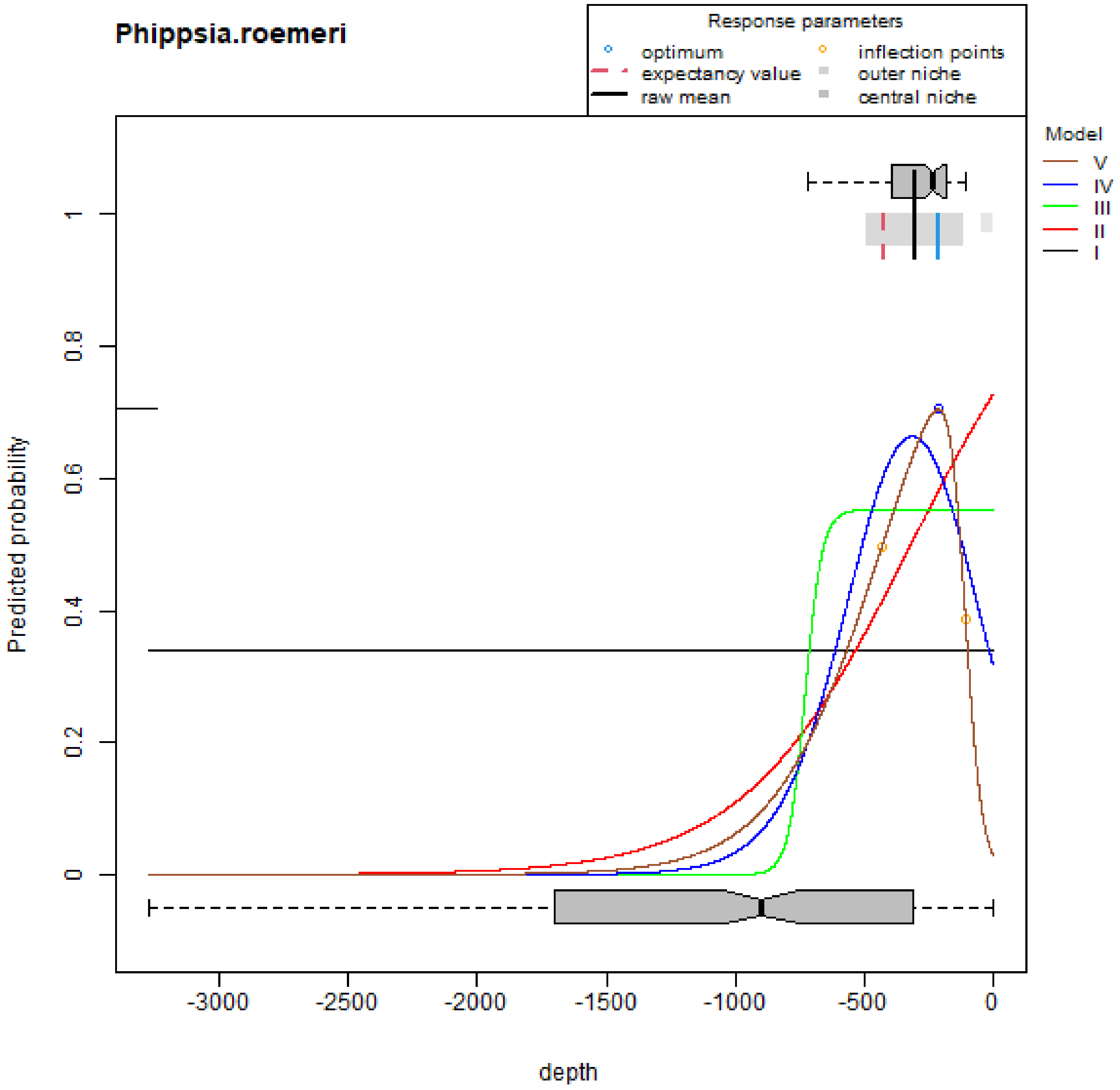

Phippsia.roemeri
Response parameters
optimum
expectancy value
raw mean
inflection points
outer niche
central niche
Model
V
IV
III
II
I
Predicted probability
0
0.2
0.4
0.6
0.8
1
-3000
-2500
-2000
-1500
-1000
-500
0
depth

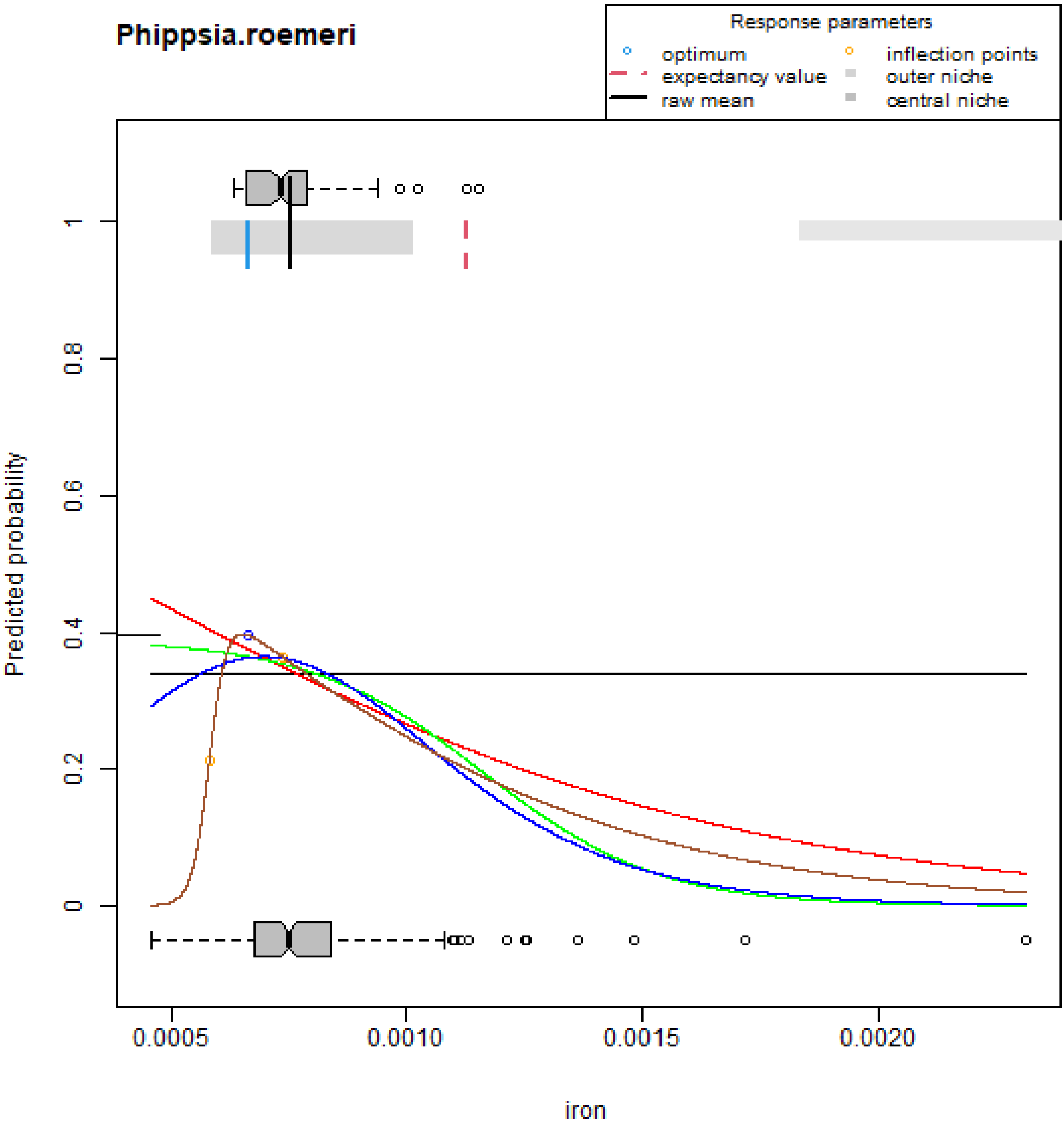
Phippsia.roemeri
Response parameters
optimum
expectancy value
raw mean
inflection points
outer niche
central niche
Predicted probability
0
0.2
0.4
0.6
0.8
1
0.0005
0.0010
0.0015
0.0020
iron

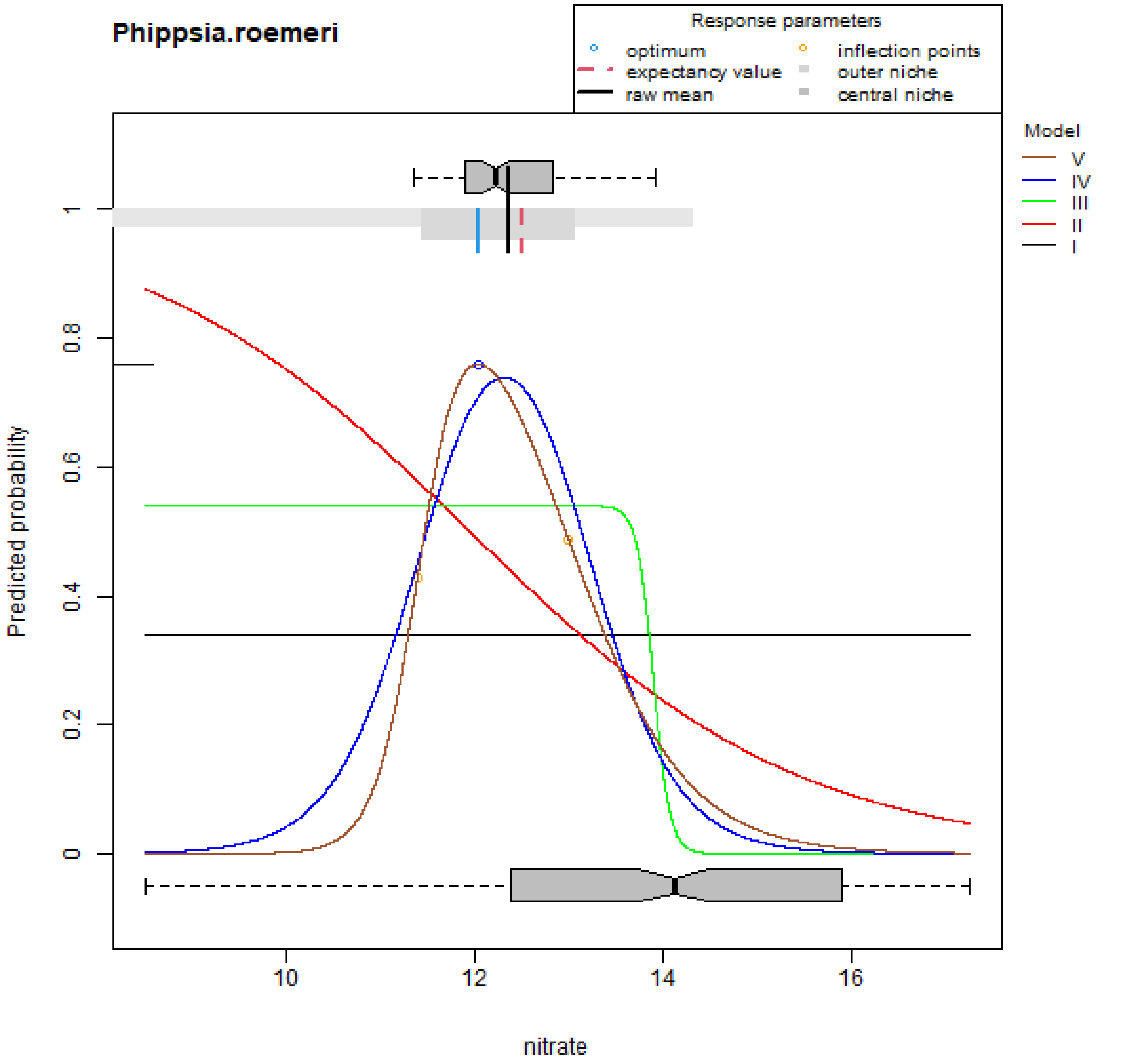
Phippsia.roemeri
Response parameters
optimum
expectancy value
raw mean
inflection points
outer niche
central niche
Model
V
IV
III
II
I
Predicted probability
0
0.2
0.4
0.6
0.8
1
10
12
14
16
nitrate

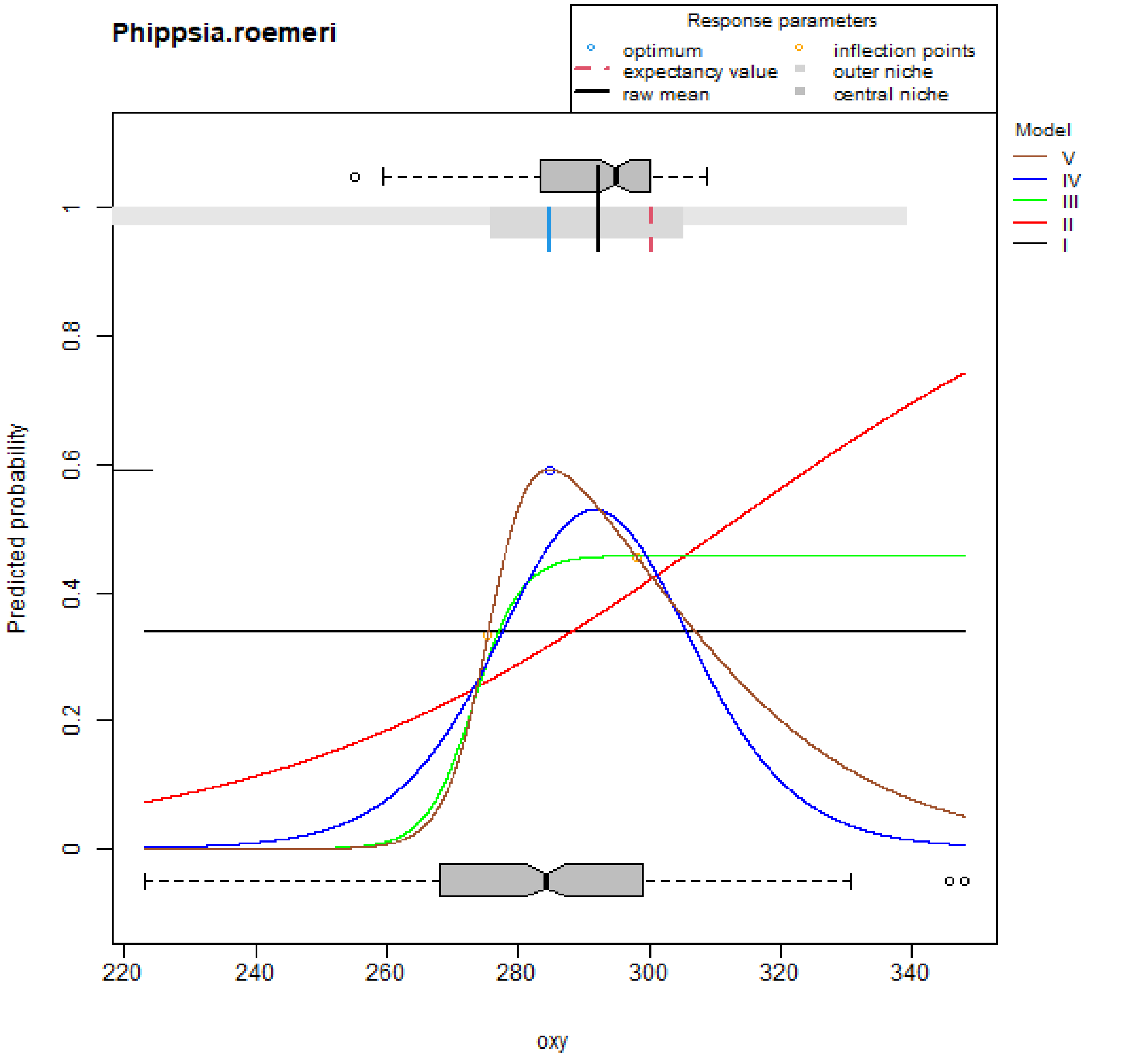

Phippsia.roemeri
Response parameters
optimum
expectancy value
raw mean
inflection points
outer niche
central niche
Model
V
IV
III
II
I
Predicted probability
0
0.2
0.4
0.6
0.8
1
220
240
260
280
300
320
340
oxy

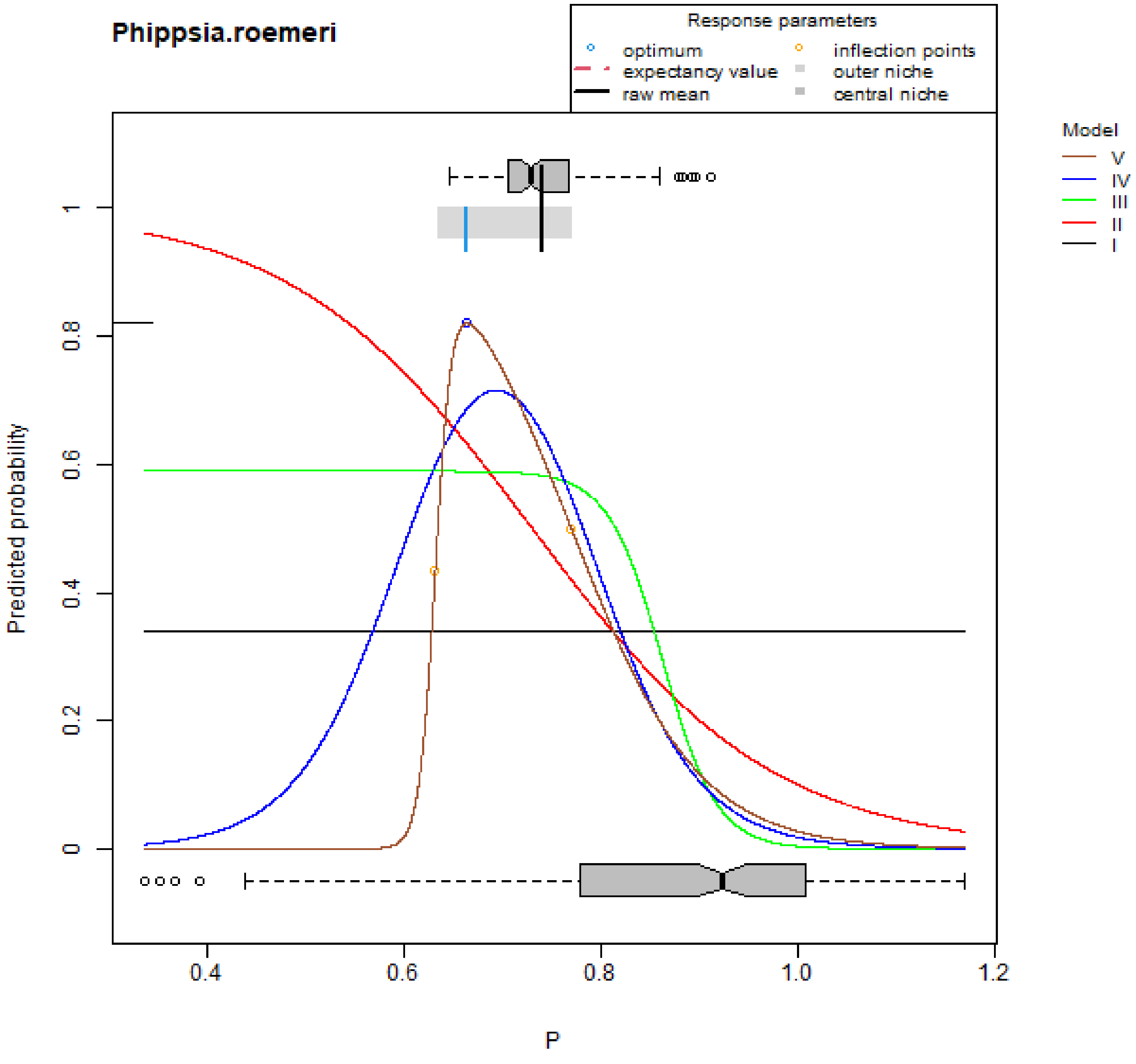
Phippsia.roemeri
Response parameters
optimum
expectancy value
raw mean
inflection points
outer niche
central niche
Model
V
IV
III
II
I
Predicted probability
0
0.2
0.4
0.6
0.8
1
0.4
0.6
0.8
1.0
1.2
P

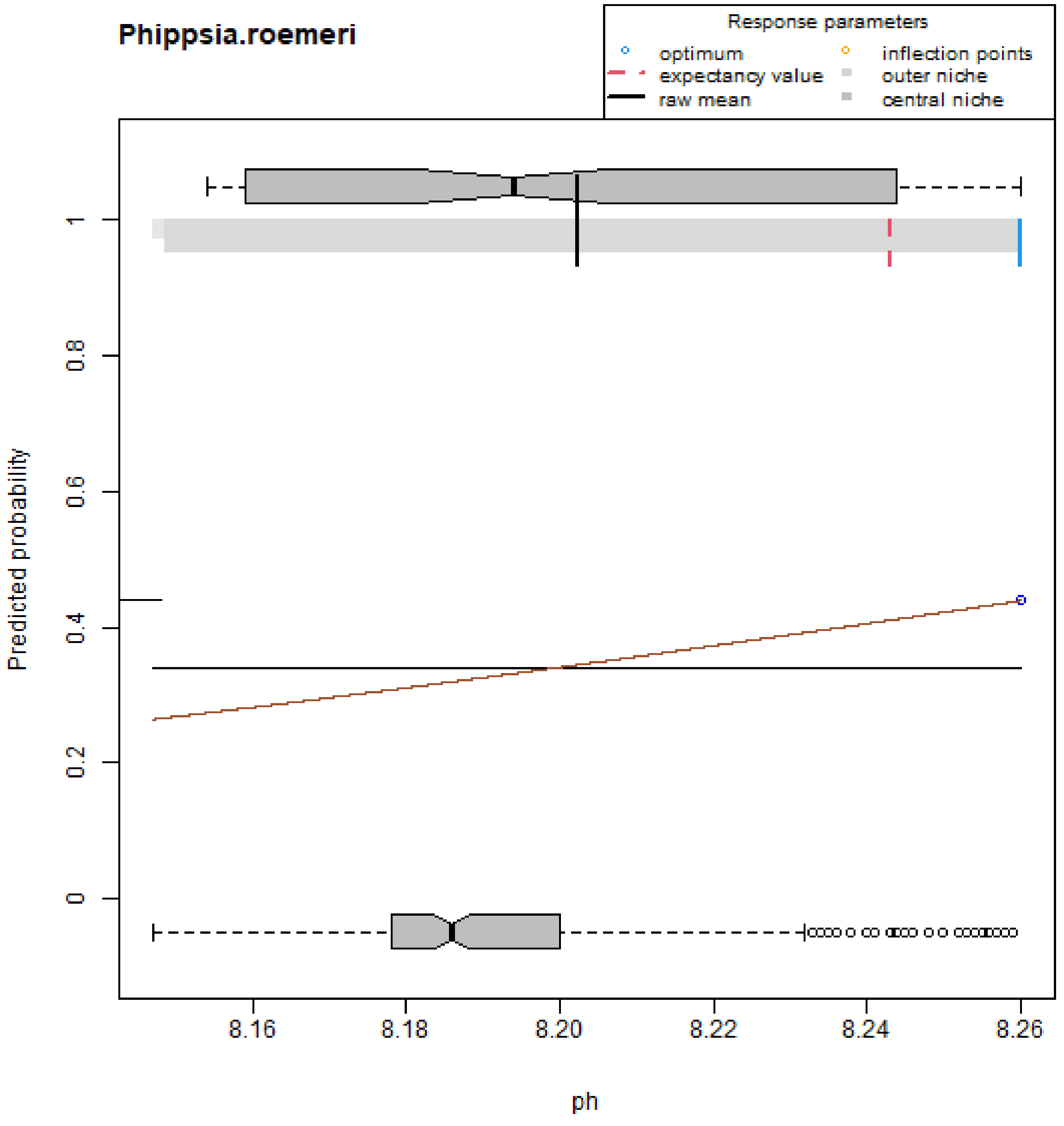

Phippsia.roemeri
Response parameters
optimum
expectancy value
raw mean
inflection points
outer niche
central niche
Predicted probability
0
0.2
0.4
0.6
0.8
1
8.16
8.18
8.20
8.22
8.24
8.26
ph

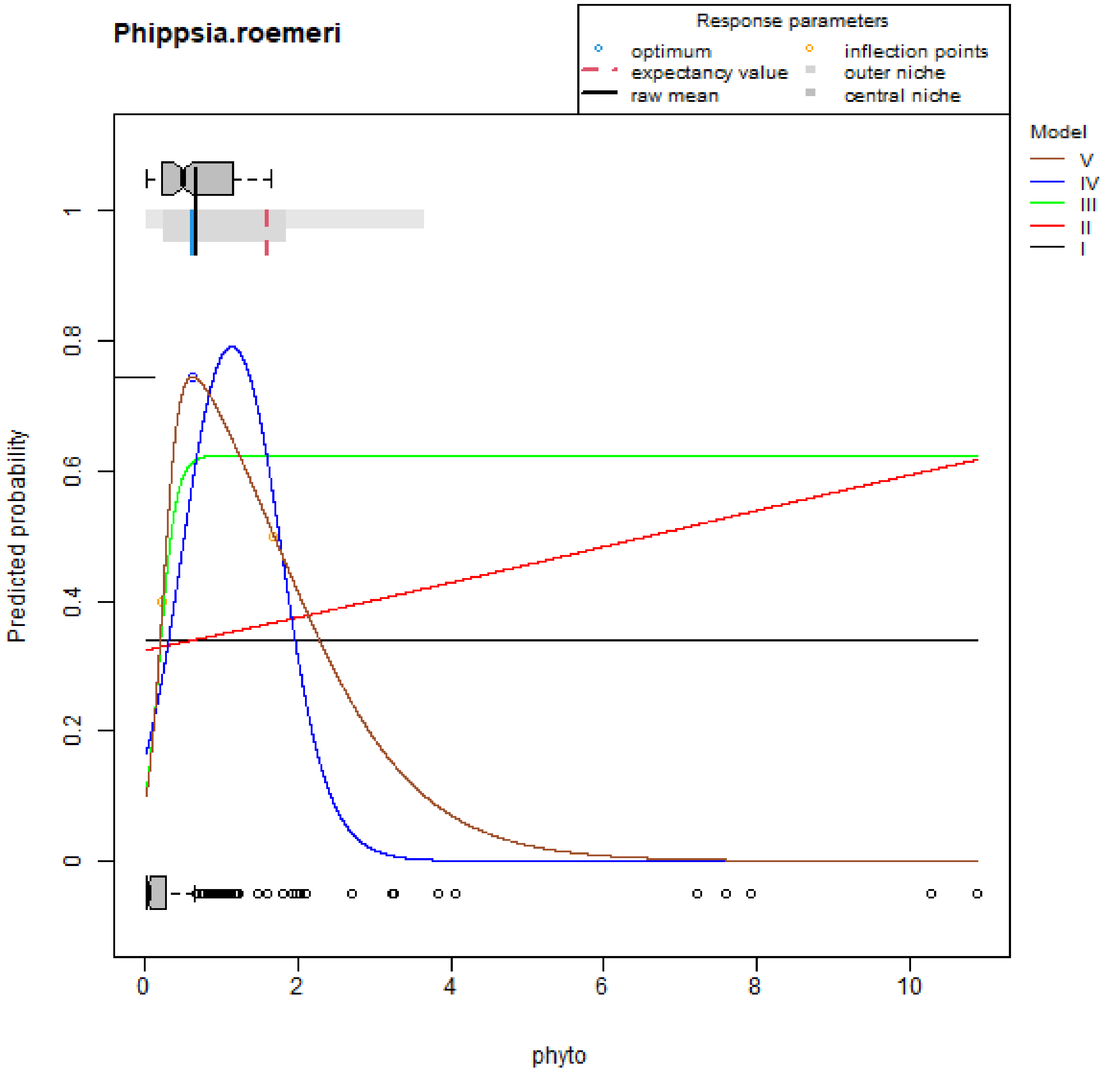

Phippsia.roemeri
Response parameters
optimum
expectancy value
raw mean
inflection points
outer niche
central niche
Model
V
IV
III
II
I
Predicted probability
0
0.2
0.4
0.6
0.8
1
0
2
4
6
8
10
phyto

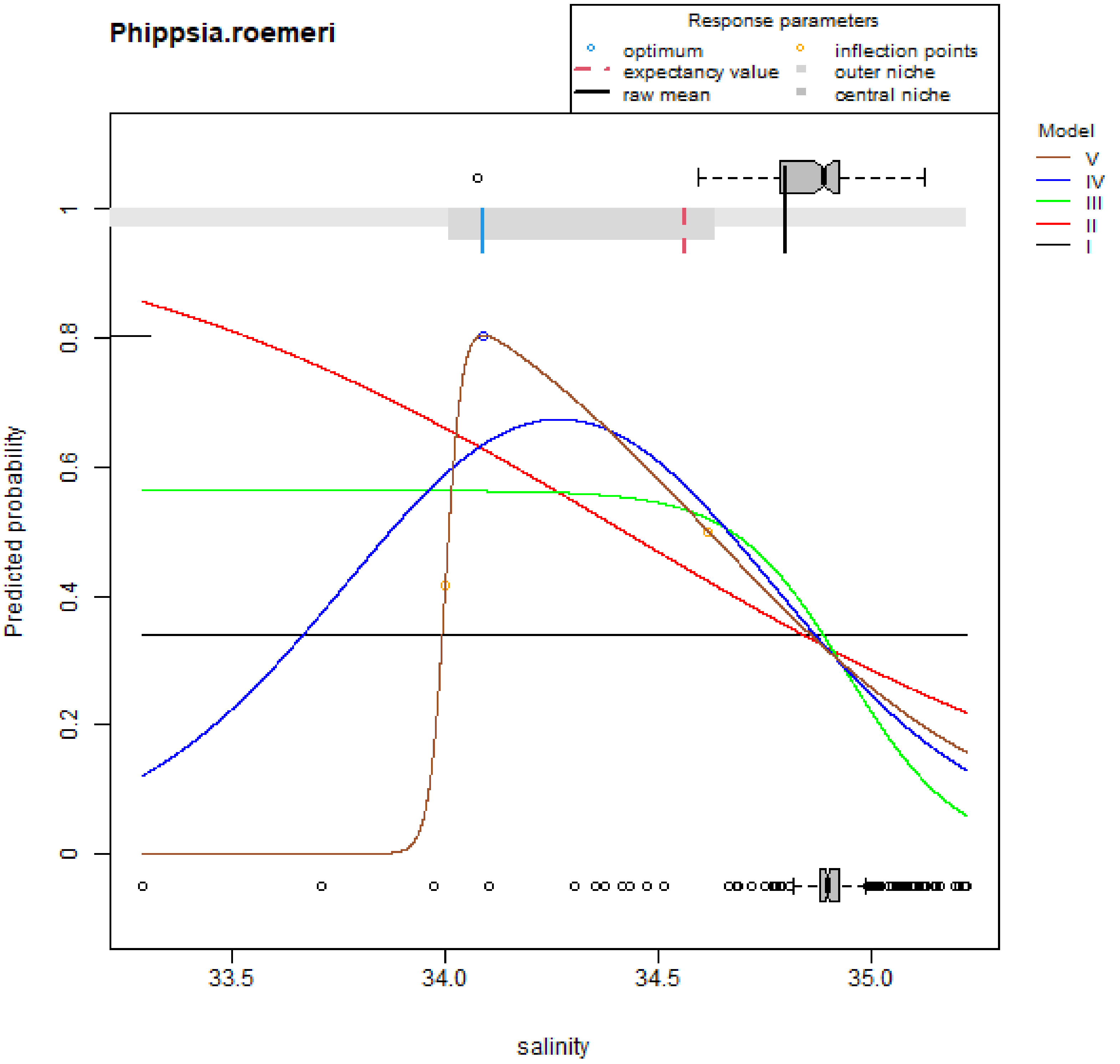

Phippsia.roemeri
Response parameters
optimum
inflection points
expectancy value
outer niche
raw mean
central niche
Model
V
IV
III
II
I
Predicted probability
0
0.2
0.4
0.6
0.8
1
33.5
34.0
34.5
35.0
salinity

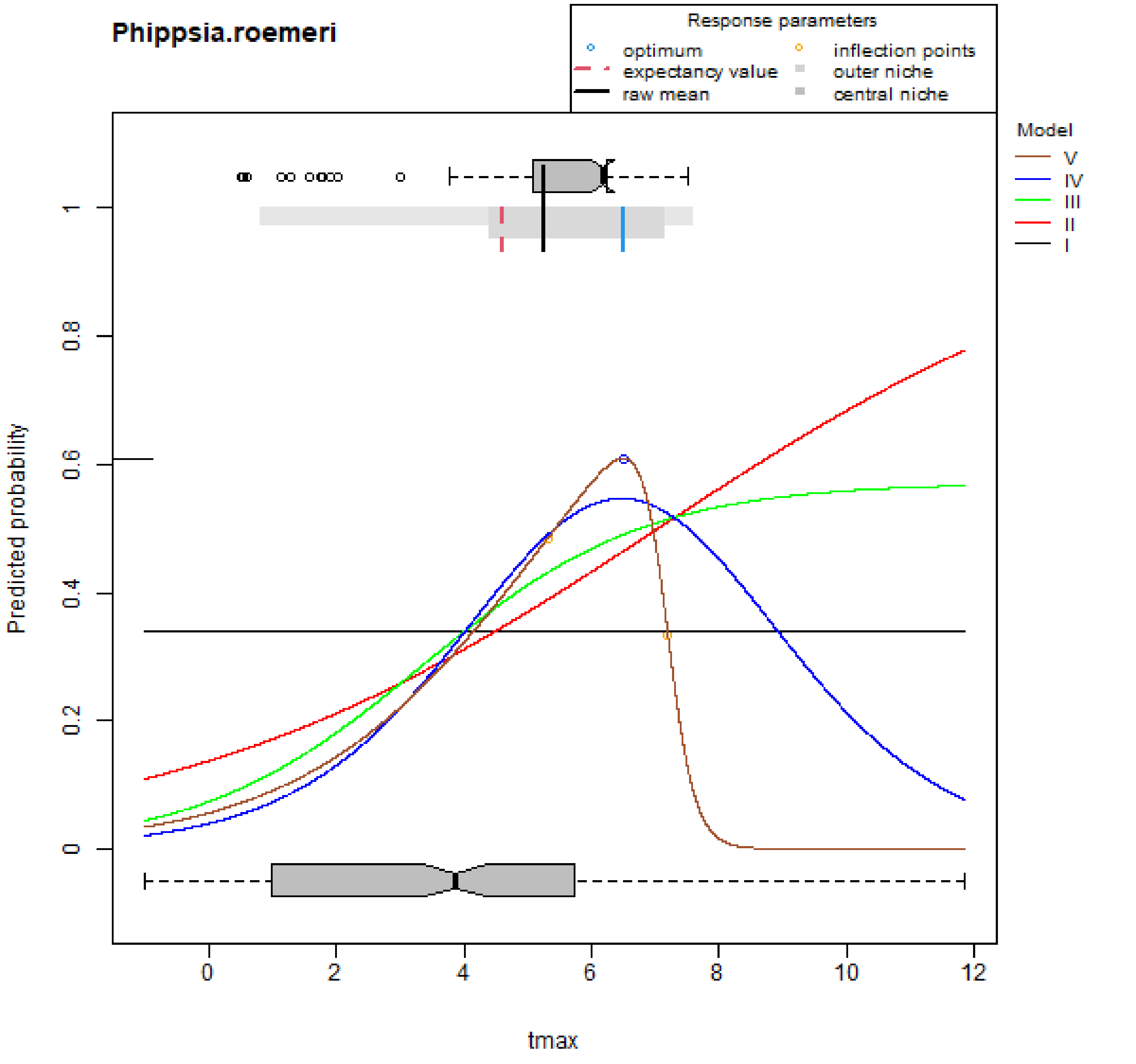

Phippsia.roemeri
Response parameters
optimum
inflection points
expectancy value
outer niche
raw mean
central niche
Model
V
IV
III
II
I
Predicted probability
0
0.2
0.4
0.6
0.8
1
0
2
4
6
8
10
12
tmax

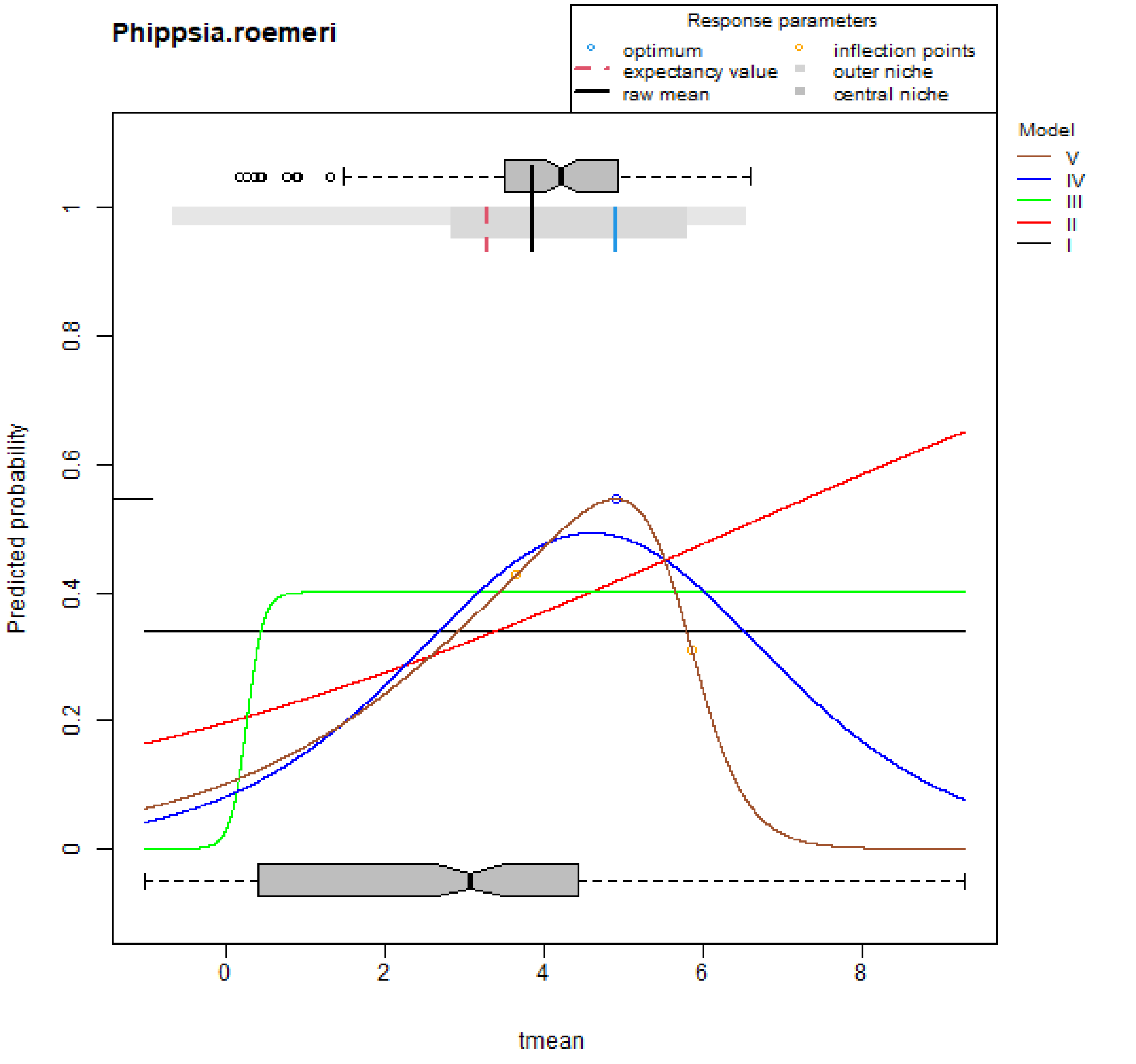

Phippsia.roemeri
Response parameters
optimum
expectancy value
raw mean
inflection points
outer niche
central niche
Model
V
IV
III
II
I
Predicted probability
0
0.2
0.4
0.6
0.8
1
0
2
4
6
8
tmean

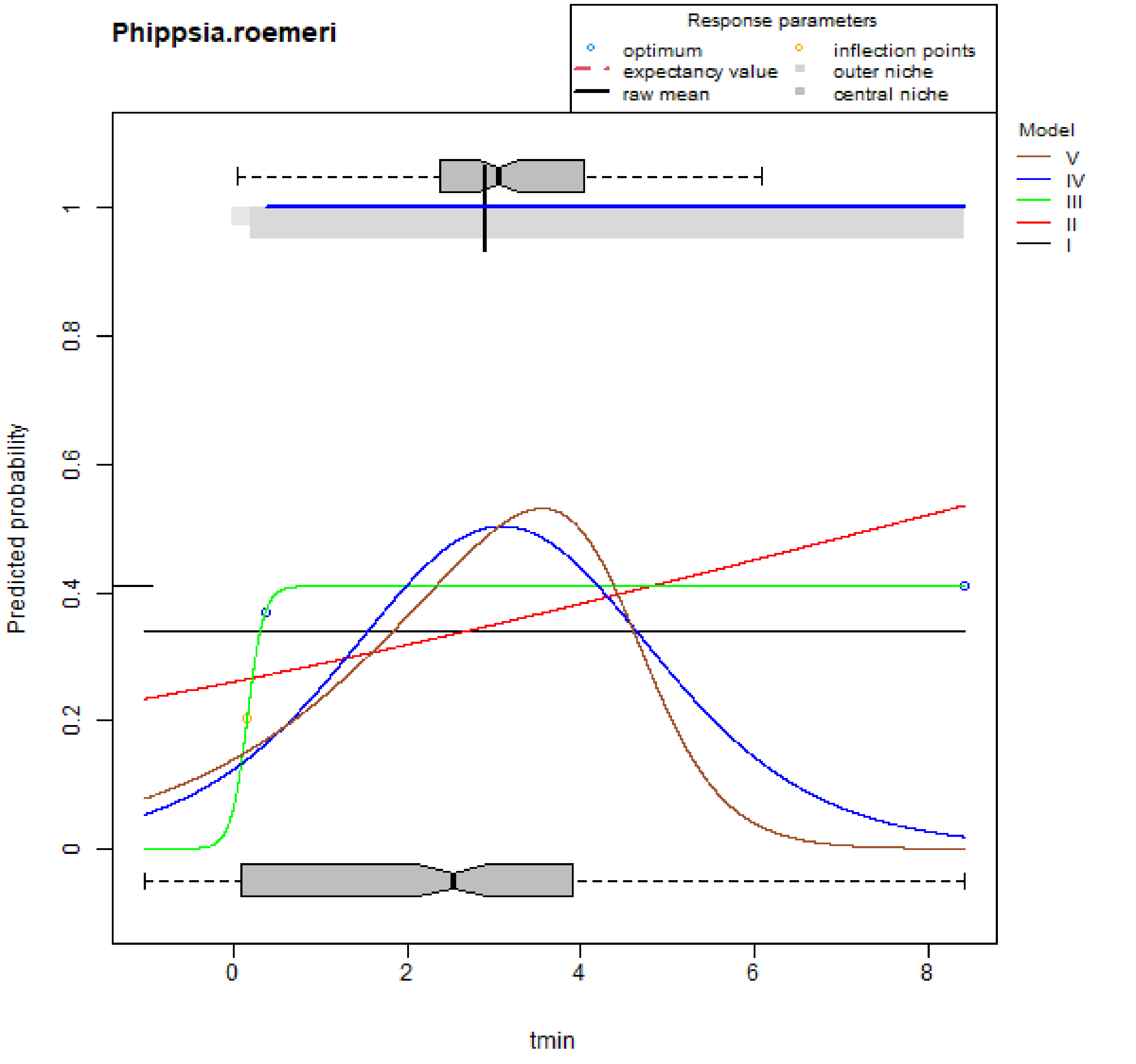
Phippsia.roemeri
Response parameters
optimum
inflection points
expectancy value
outer niche
raw mean
central niche
Model
V
IV
III
II
I
Predicted probability
0
0.2
0.4
0.6
0.8
1
0
2
4
6
8
tmin

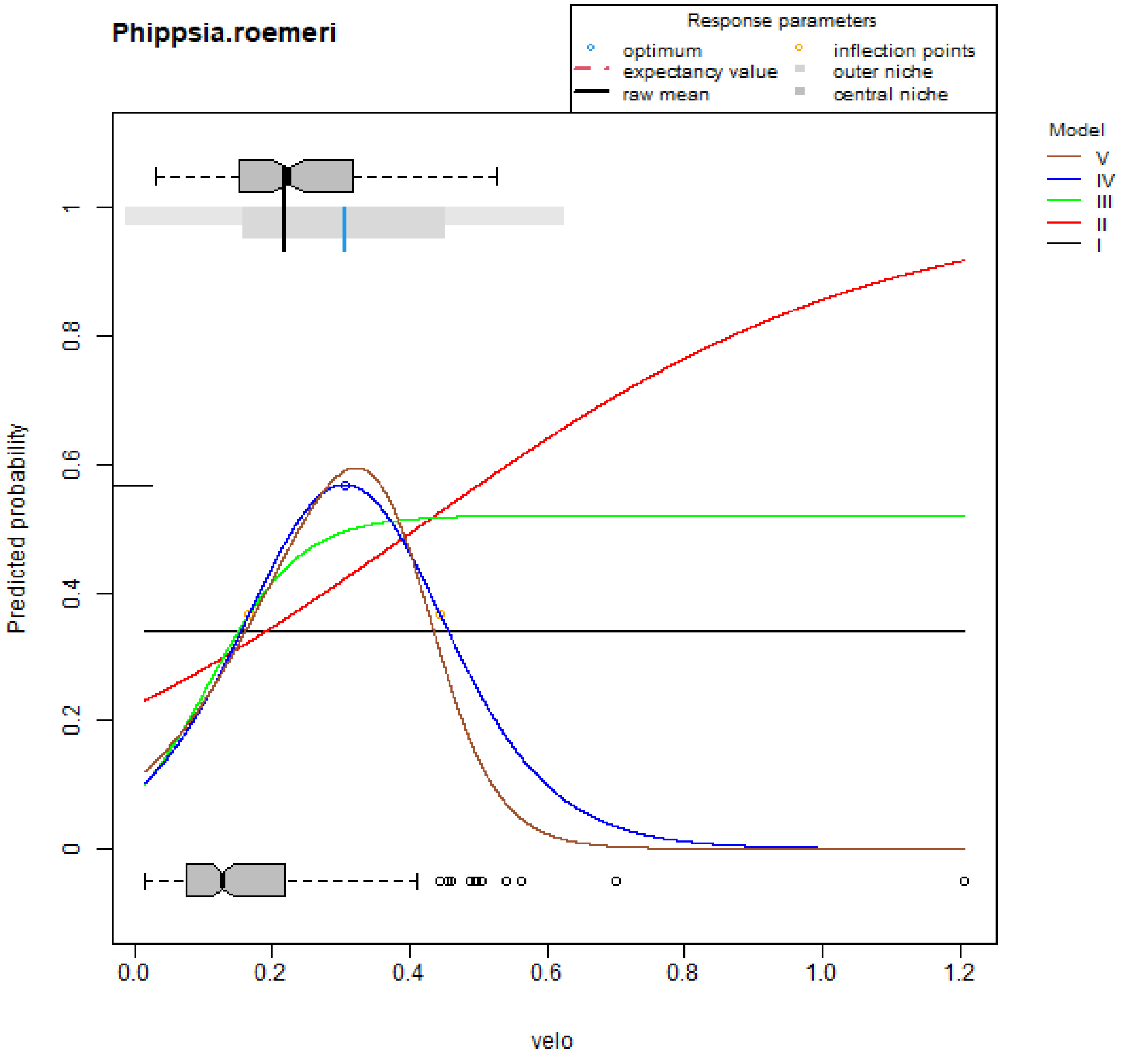
Phippsia.roemeri
Response parameters
optimum
expectancy value
raw mean
inflection points
outer niche
central niche
Model
V
IV
III
II
I
Predicted probability
0
0.2
0.4
0.6
0.8
1
0.0
0.2
0.4
0.6
0.8
1.0
1.2
velo

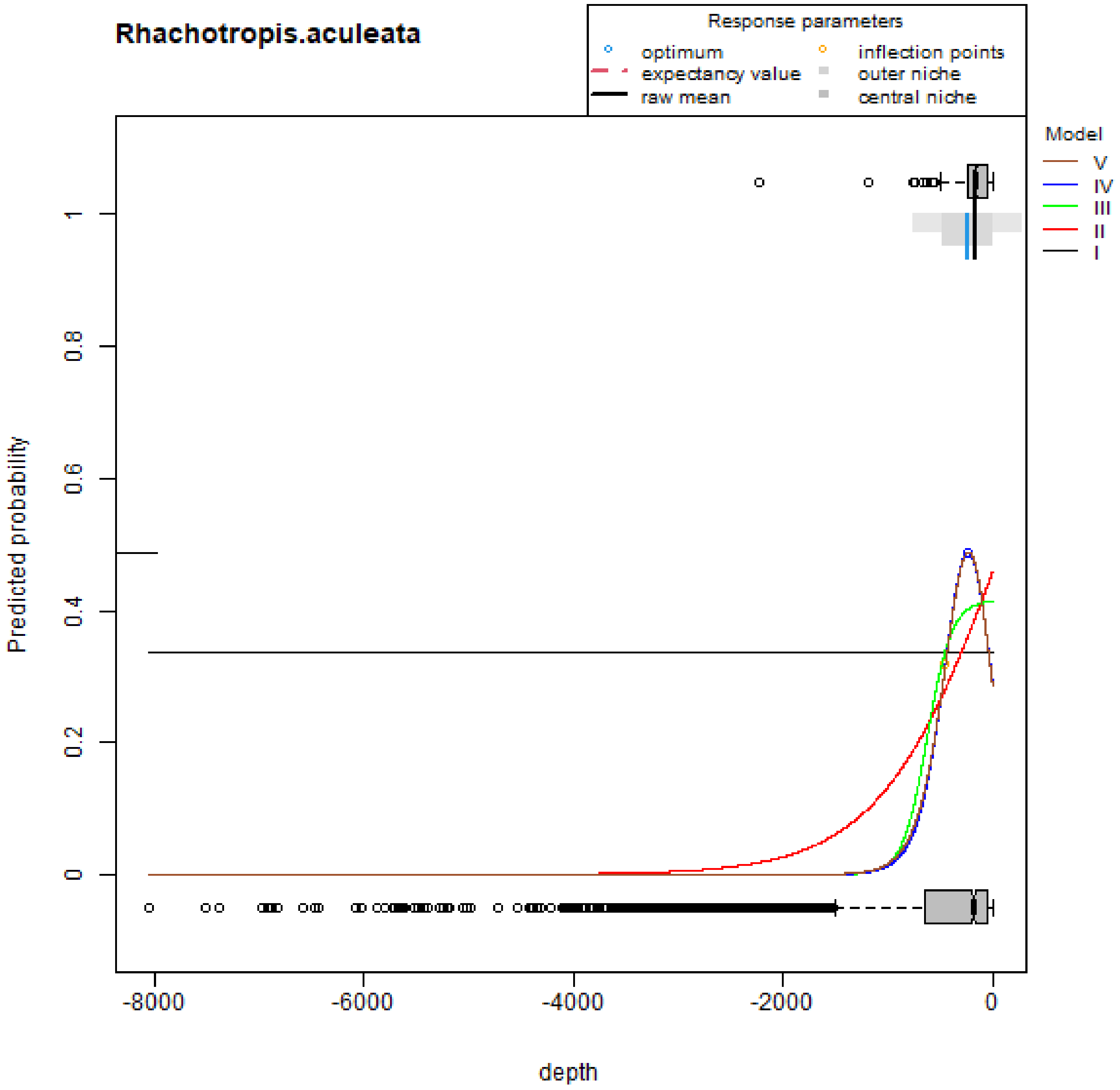

Rhachotropis.aculeata
Response parameters
optimum
expectancy value
raw mean
inflection points
outer niche
central niche
Model
V
IV
III
II
I
Predicted probability
0
0.2
0.4
0.6
0.8
1
-8000
-6000
-4000
-2000
0
depth

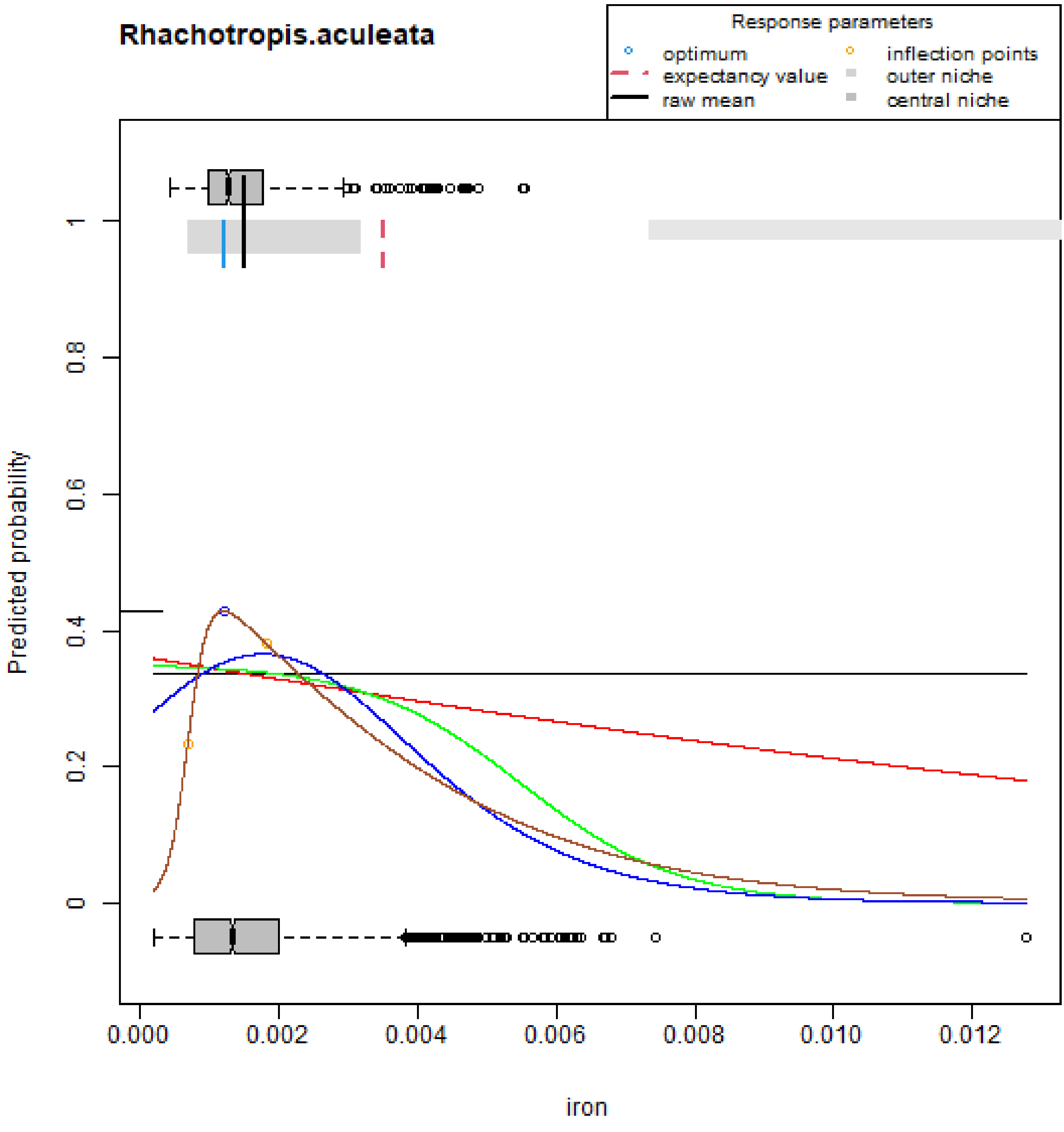
Rhachotropis.aculeata
Response parameters
optimum
expectancy value
raw mean
inflection points
outer niche
central niche
Predicted probability
0
0.2
0.4
0.6
0.8
1
0.000
0.002
0.004
0.006
0.008
0.010
0.012
iron

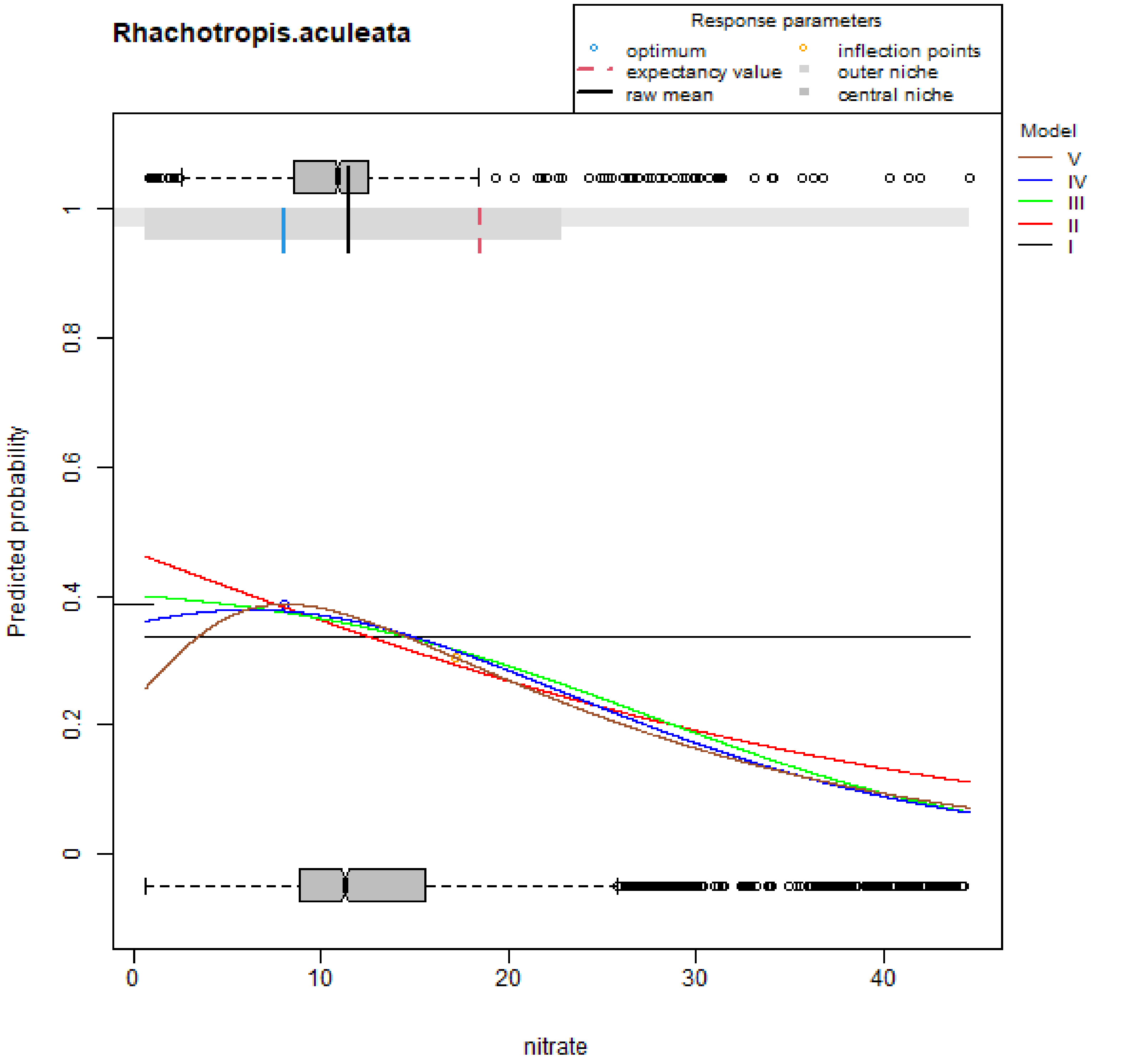

Rhachotropis.aculeata
Response parameters
optimum
expectancy value
raw mean
inflection points
outer niche
central niche
Model
V
IV
III
II
I
Predicted probability
0
0.2
0.4
0.6
0.8
1
0
10
20
30
40
nitrate

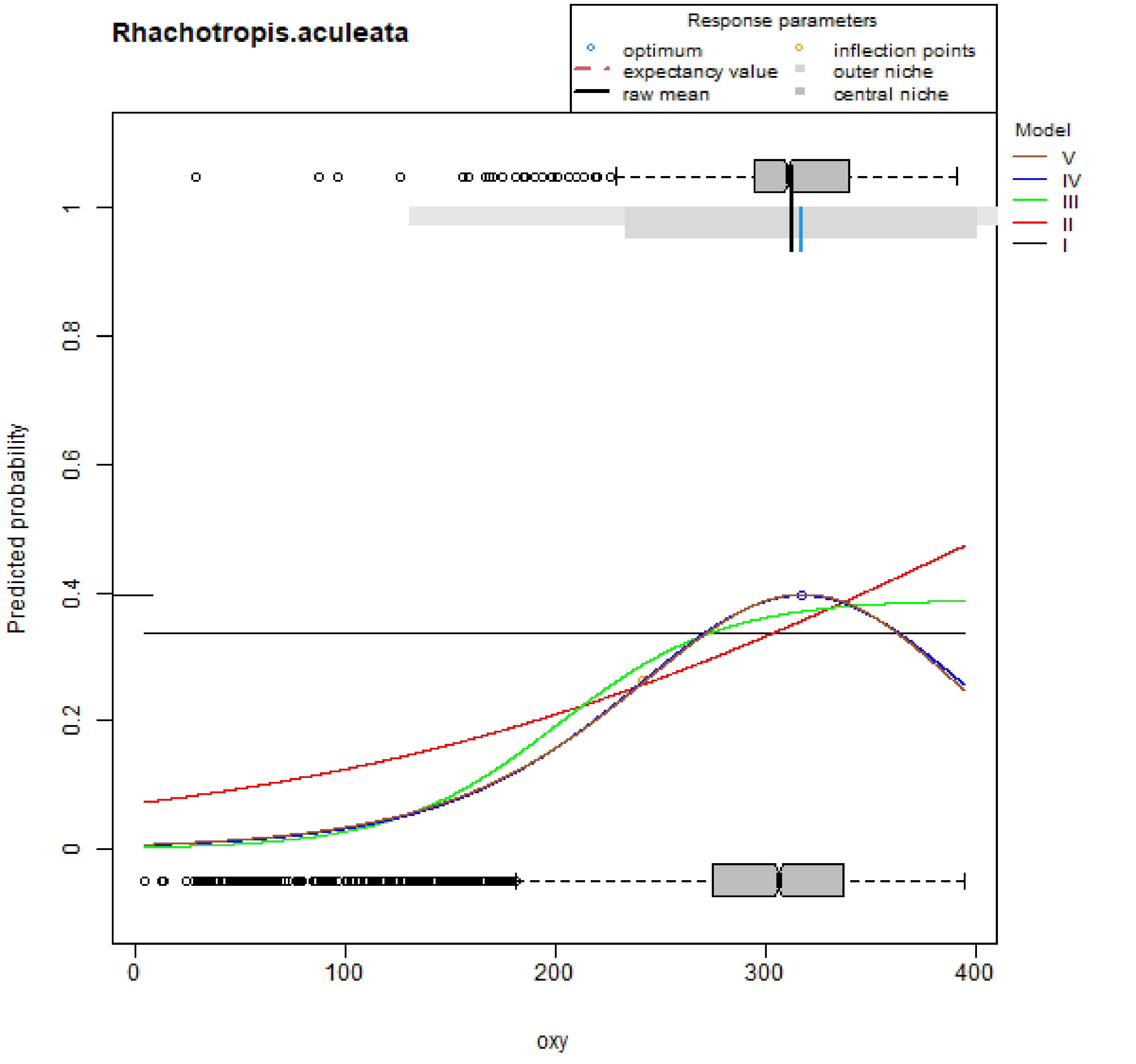
Rhachotropis.aculeata
Response parameters
optimum
expectancy value
raw mean
inflection points
outer niche
central niche
Model
V
IV
III
II
I
Predicted probability
0
0.2
0.4
0.6
0.8
1
0
100
200
300
400
oxy

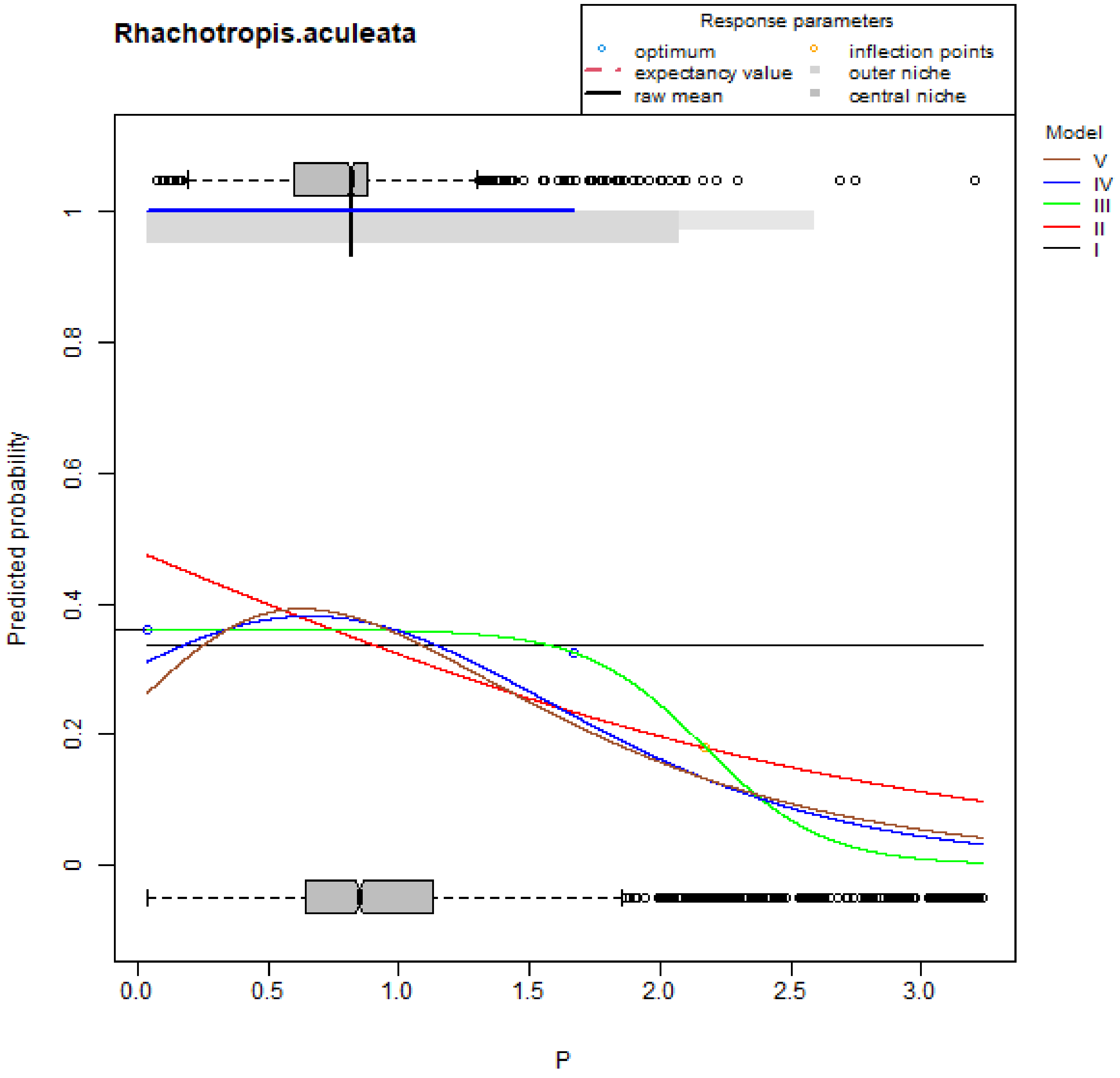

Rhachotropis.aculeata
Response parameters
optimum
inflection points
expectancy value
outer niche
raw mean
central niche
Model
V
IV
III
II
I
Predicted probability
0
0.2
0.4
0.6
0.8
1
0.0
0.5
1.0
1.5
2.0
2.5
3.0
P

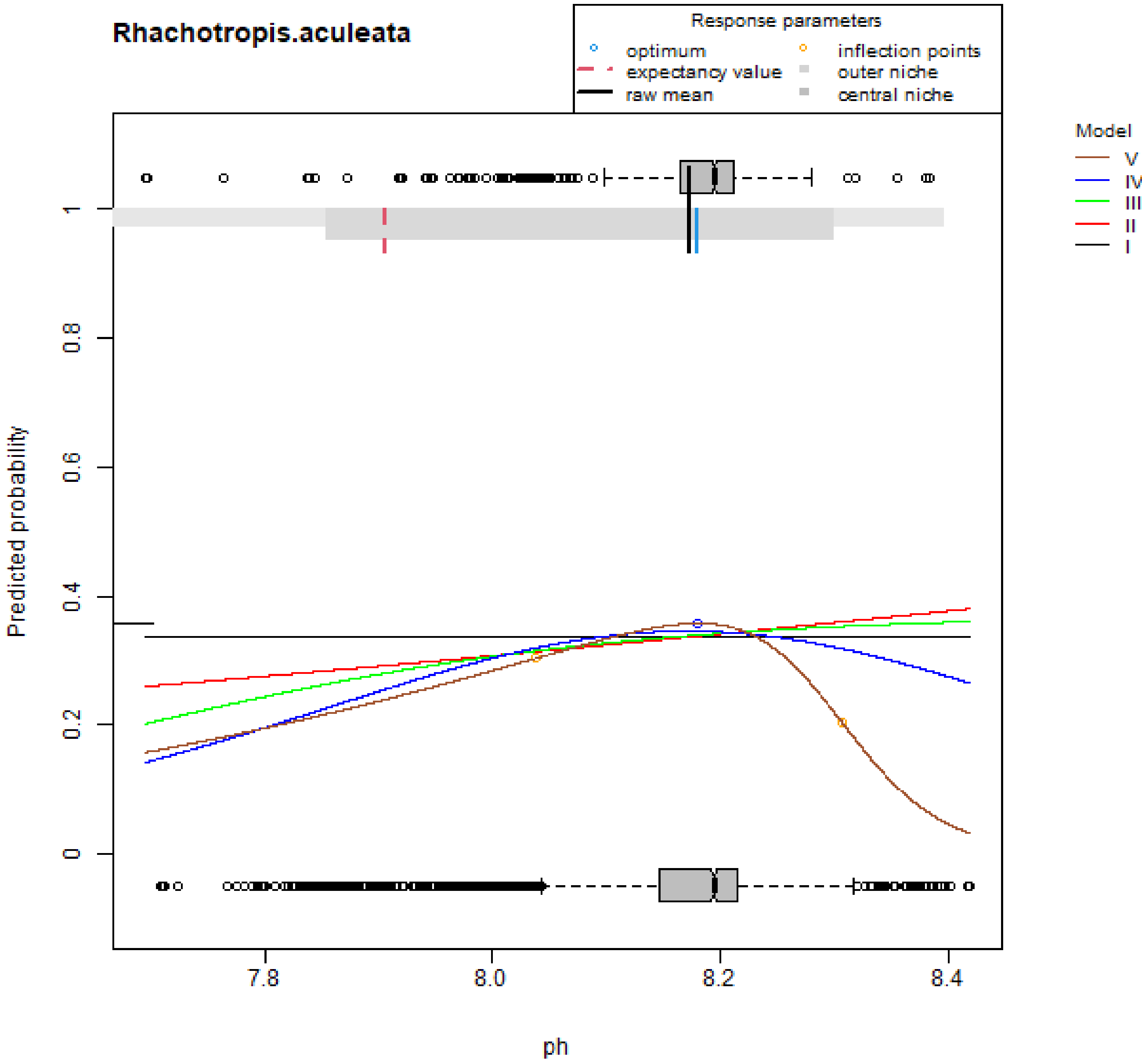

Rhachotropis.aculeata
Response parameters
optimum
expectancy value
raw mean
inflection points
outer niche
central niche
Model
V
IV
III
II
I
Predicted probability
0
0.2
0.4
0.6
0.8
1
7.8
8.0
8.2
8.4
ph

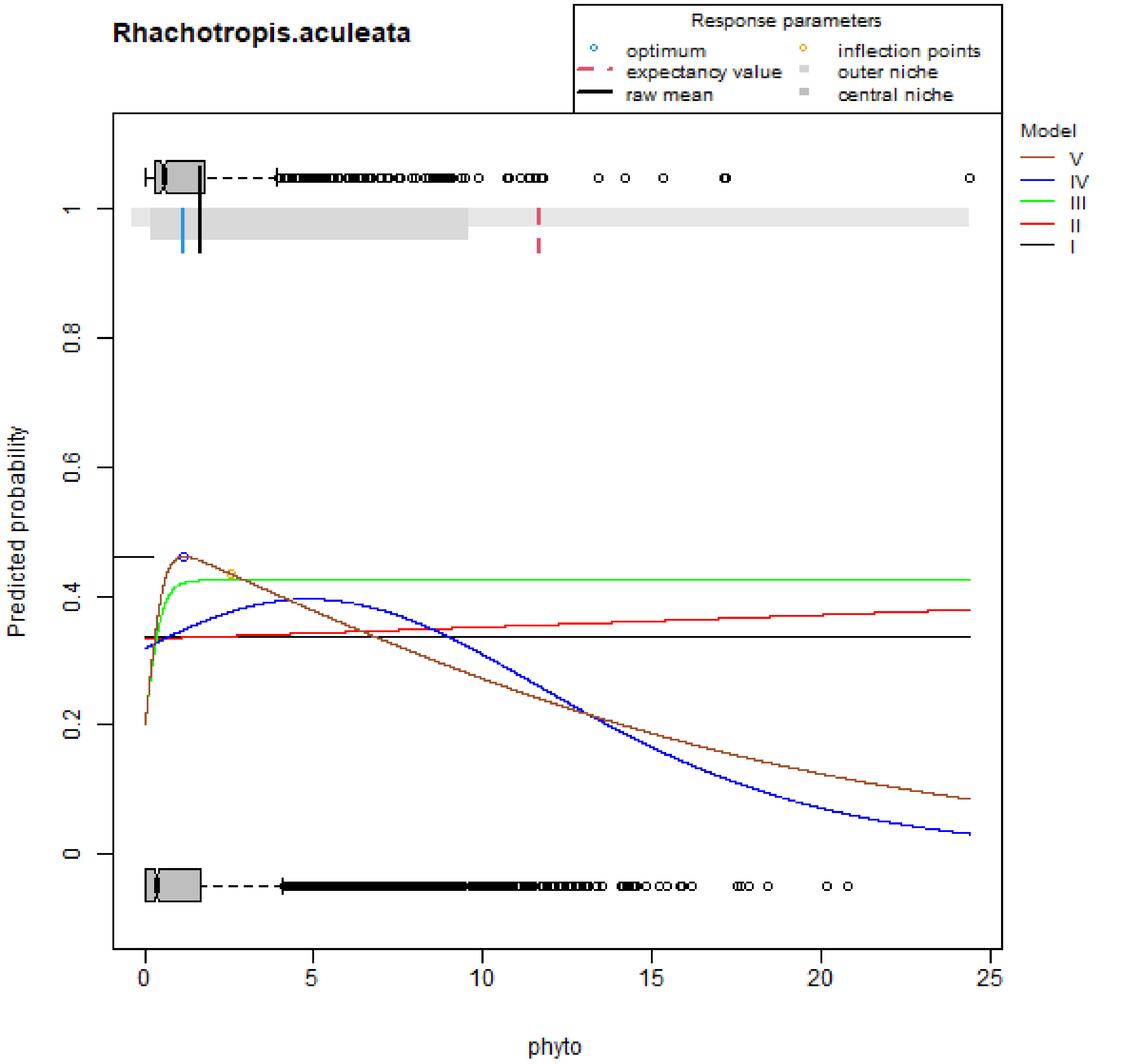

Rhachotropis.aculeata
Response parameters
optimum
expectancy value
raw mean
inflection points
outer niche
central niche
Model
V
IV
III
II
I
Predicted probability
0
0.2
0.4
0.6
0.8
1
0
5
10
15
20
25
phyto

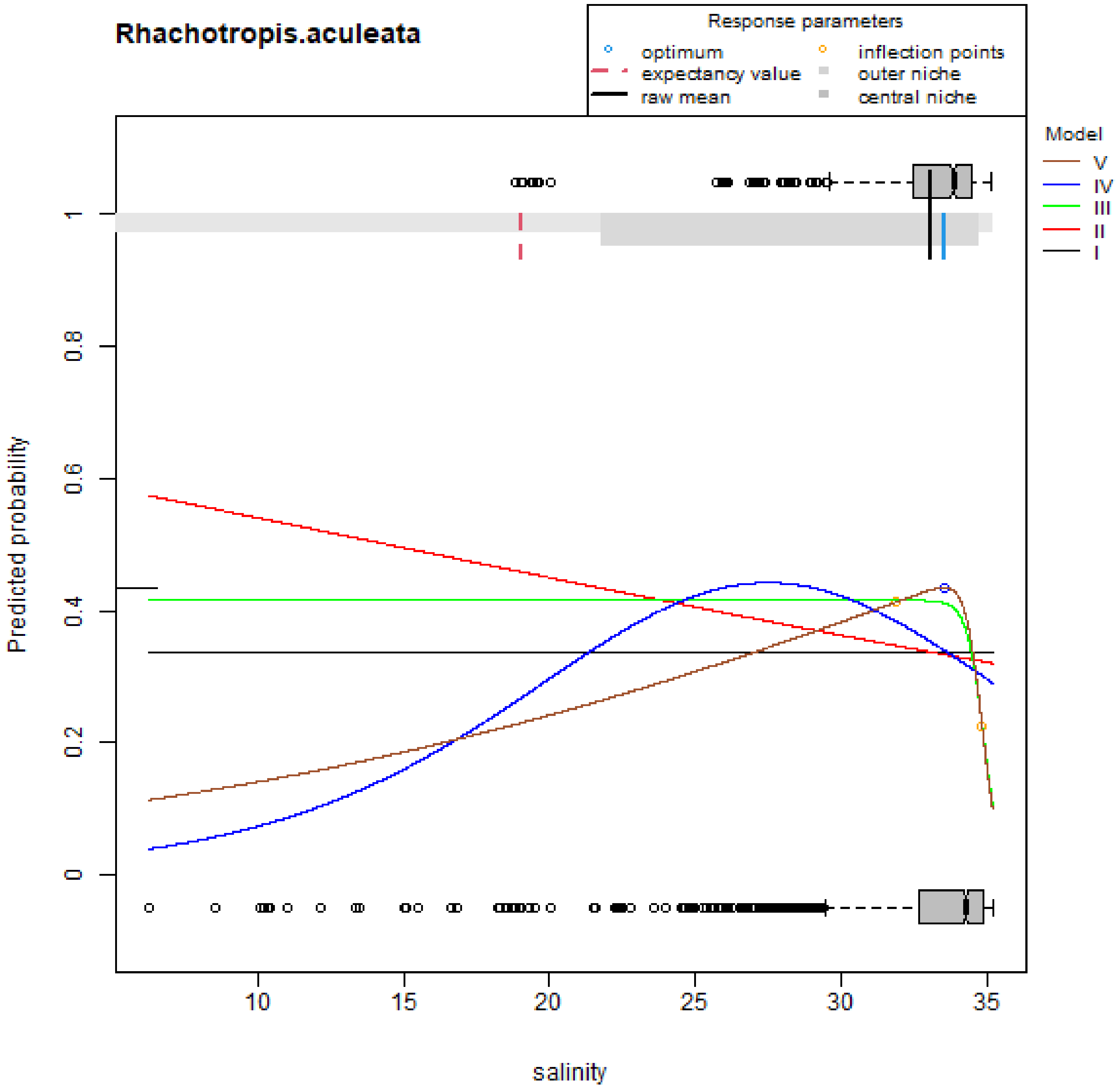

Rhachotropis.aculeata
Response parameters
optimum
expectancy value
raw mean
inflection points
outer niche
central niche
Model
V
IV
III
II
I
Predicted probability
0
0.2
0.4
0.6
0.8
1
10
15
20
25
30
35
salinity

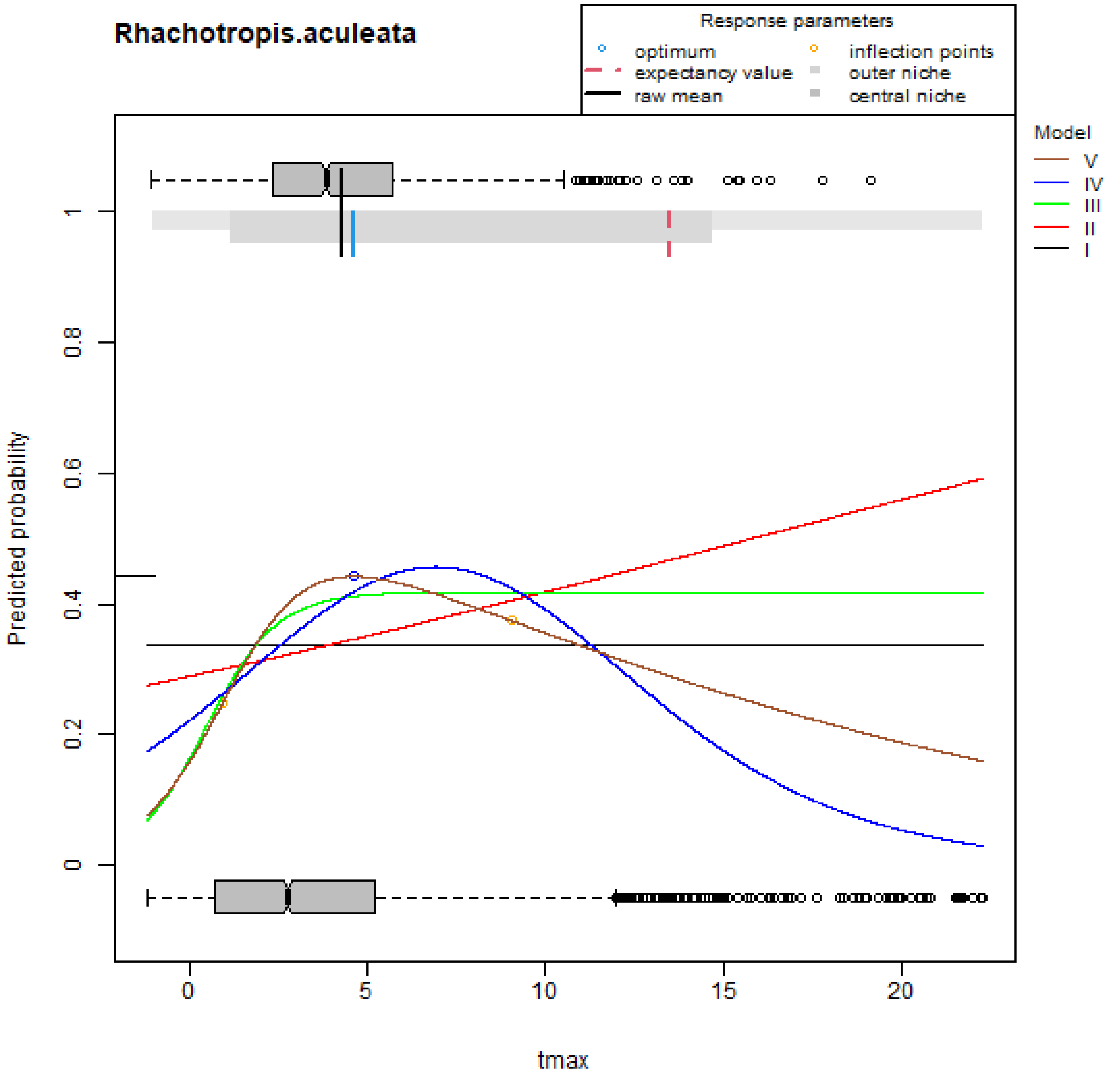

Rhachotropis.aculeata
Response parameters
optimum
inflection points
expectancy value
outer niche
raw mean
central niche
Model
V
IV
III
II
I
Predicted probability
0
0.2
0.4
0.6
0.8
1
0
5
10
15
20
tmax

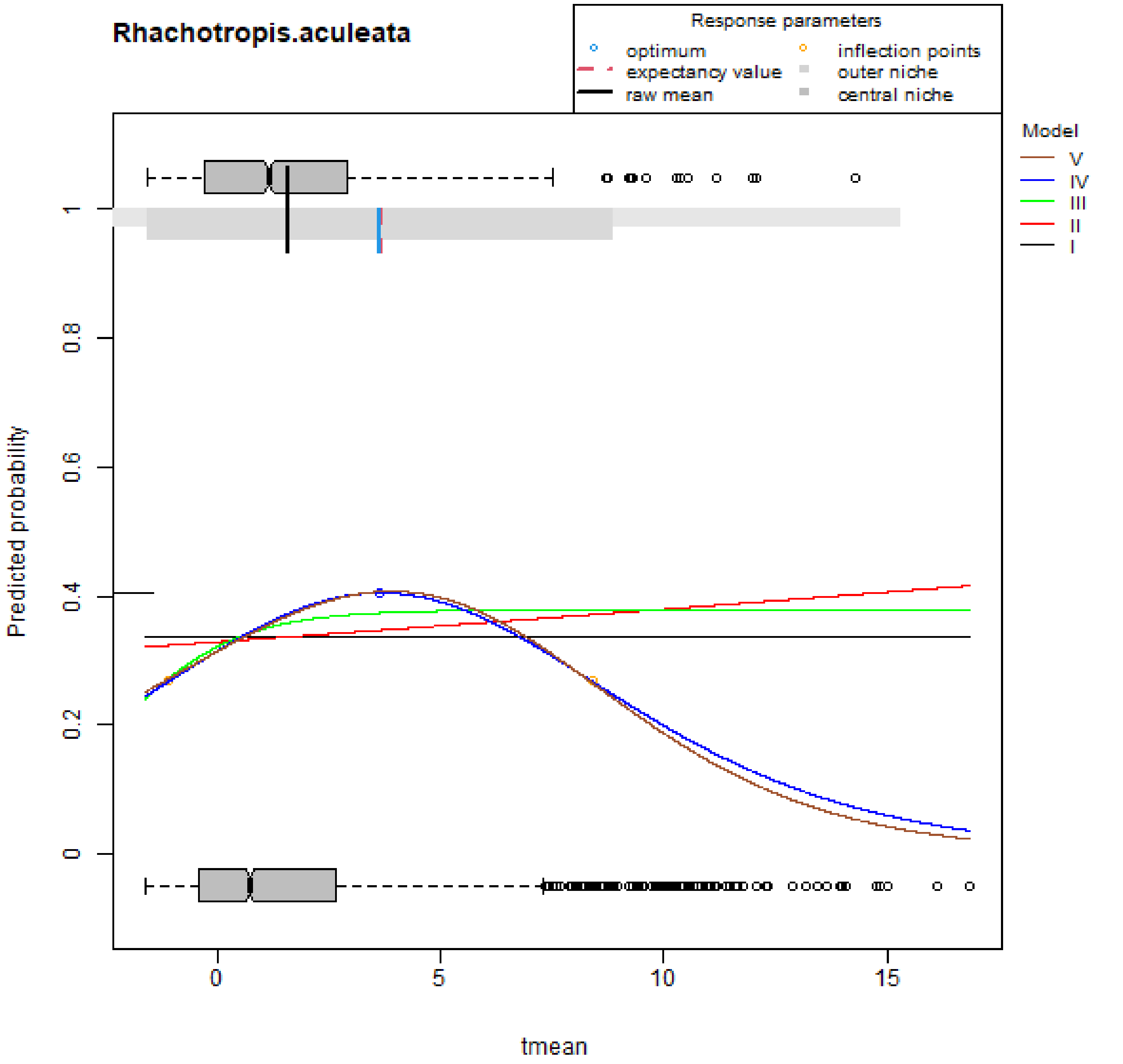
Rhachotropis.aculeata
Response parameters
optimum
expectancy value
raw mean
inflection points
outer niche
central niche
Model
V
IV
III
II
I
Predicted probability
0
0.2
0.4
0.6
0.8
1
0
5
10
15
tmean

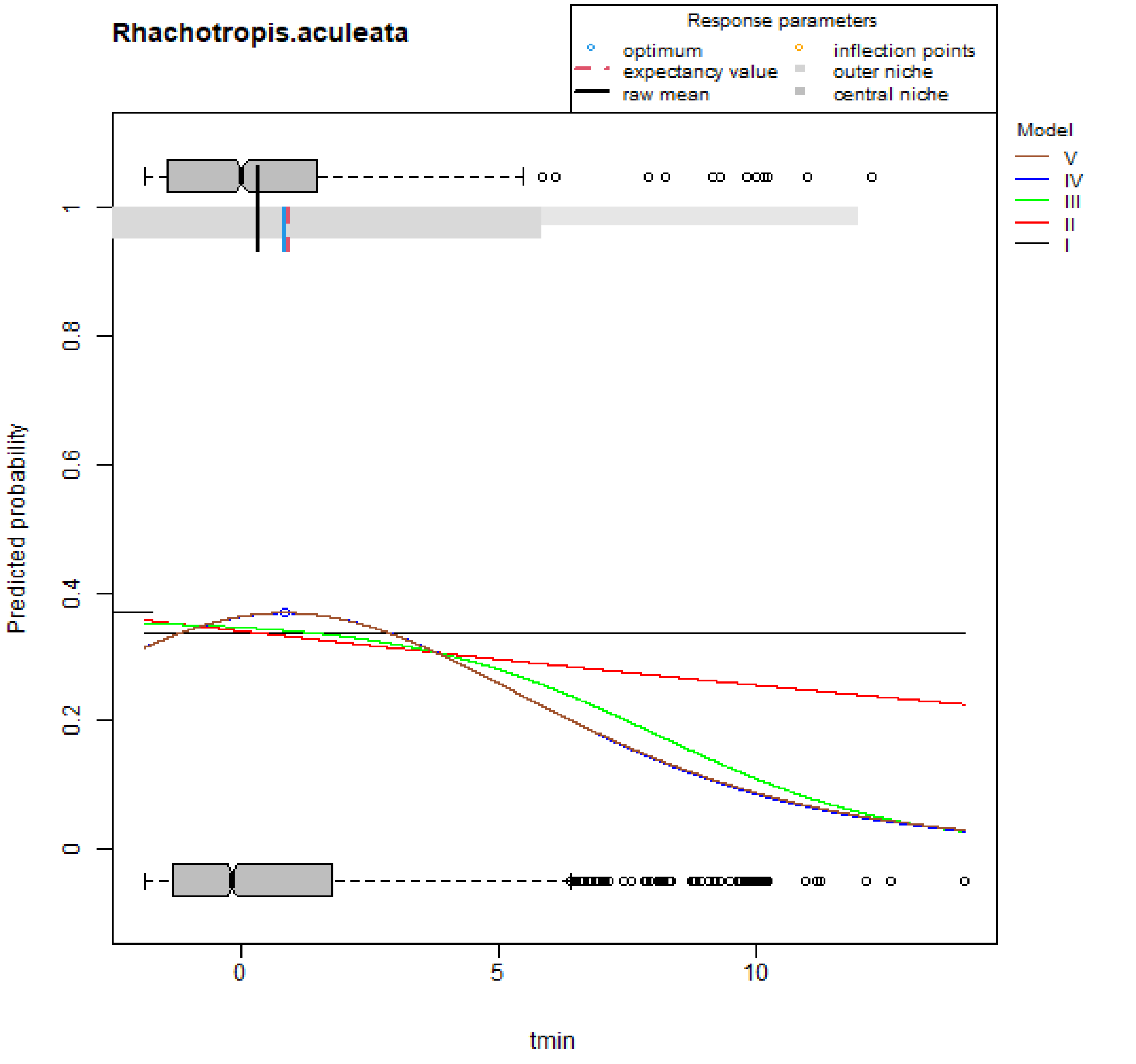

Rhachotropis.aculeata
Response parameters
optimum
expectancy value
raw mean
inflection points
outer niche
central niche
Model
V
IV
III
II
I
Predicted probability
0
0.2
0.4
0.6
0.8
1
0
5
10
tmin

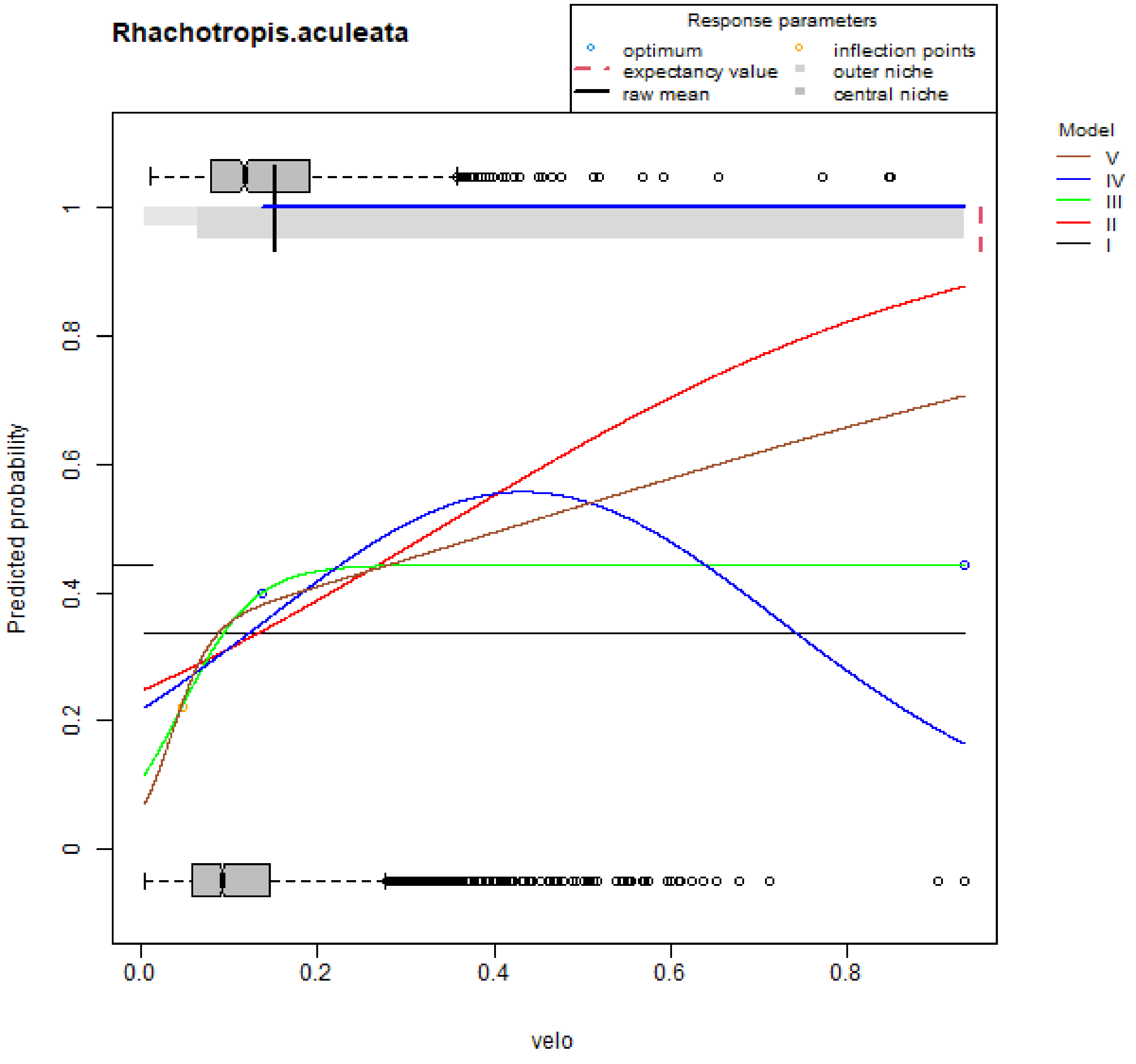

Rhachotropis.aculeata
Response parameters
optimum
inflection points
expectancy value
outer niche
raw mean
central niche
Model
V
IV
III
II
I
Predicted probability
0
0.2
0.4
0.6
0.8
1
0.0
0.2
0.4
0.6
0.8
velo

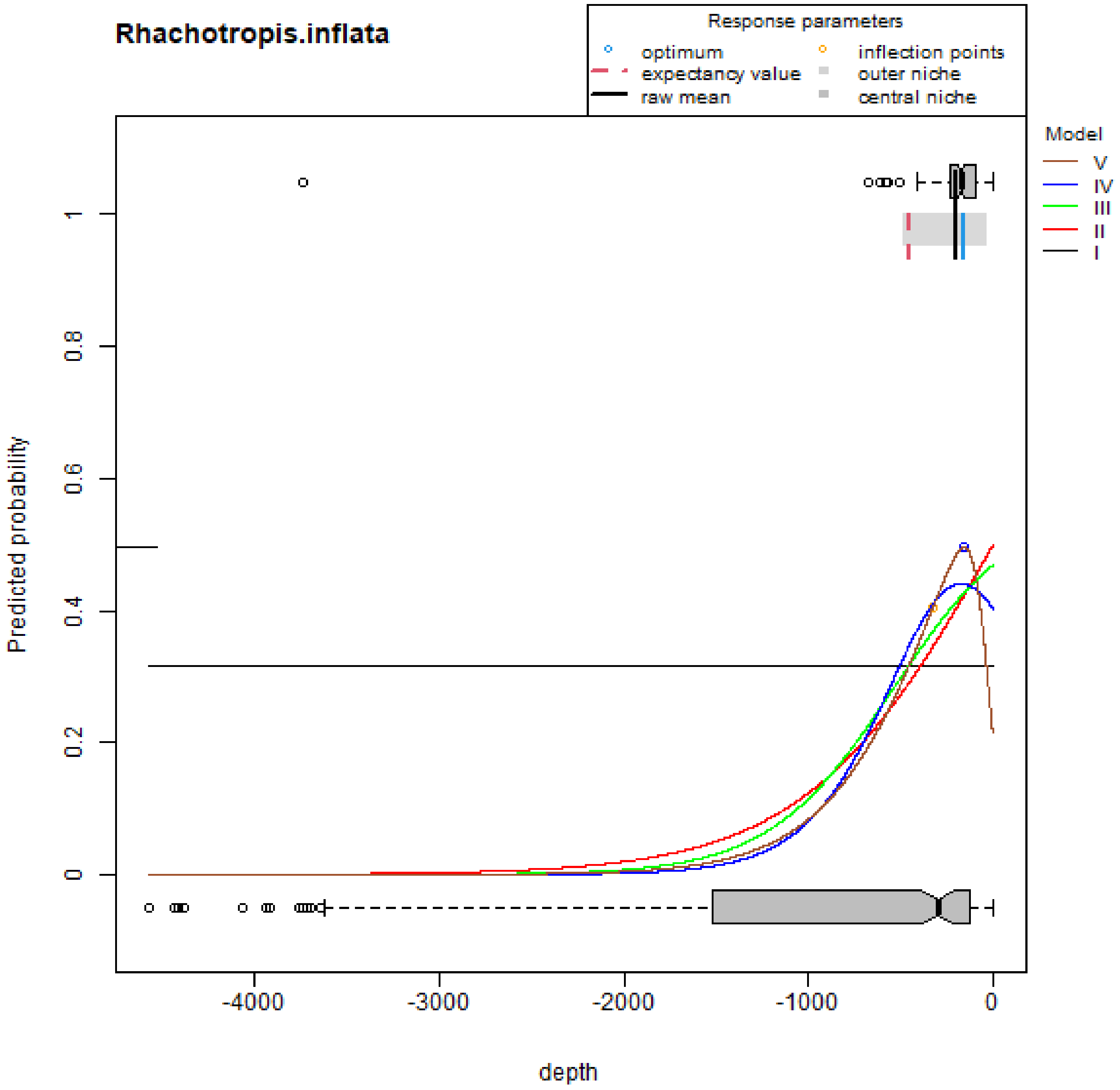

Rhachotropis.inflata
Response parameters
optimum
inflection points
expectancy value
outer niche
raw mean
central niche
Model
V
IV
III
II
I
Predicted probability
0
0.2
0.4
0.6
0.8
1
-4000
-3000
-2000
-1000
0
depth

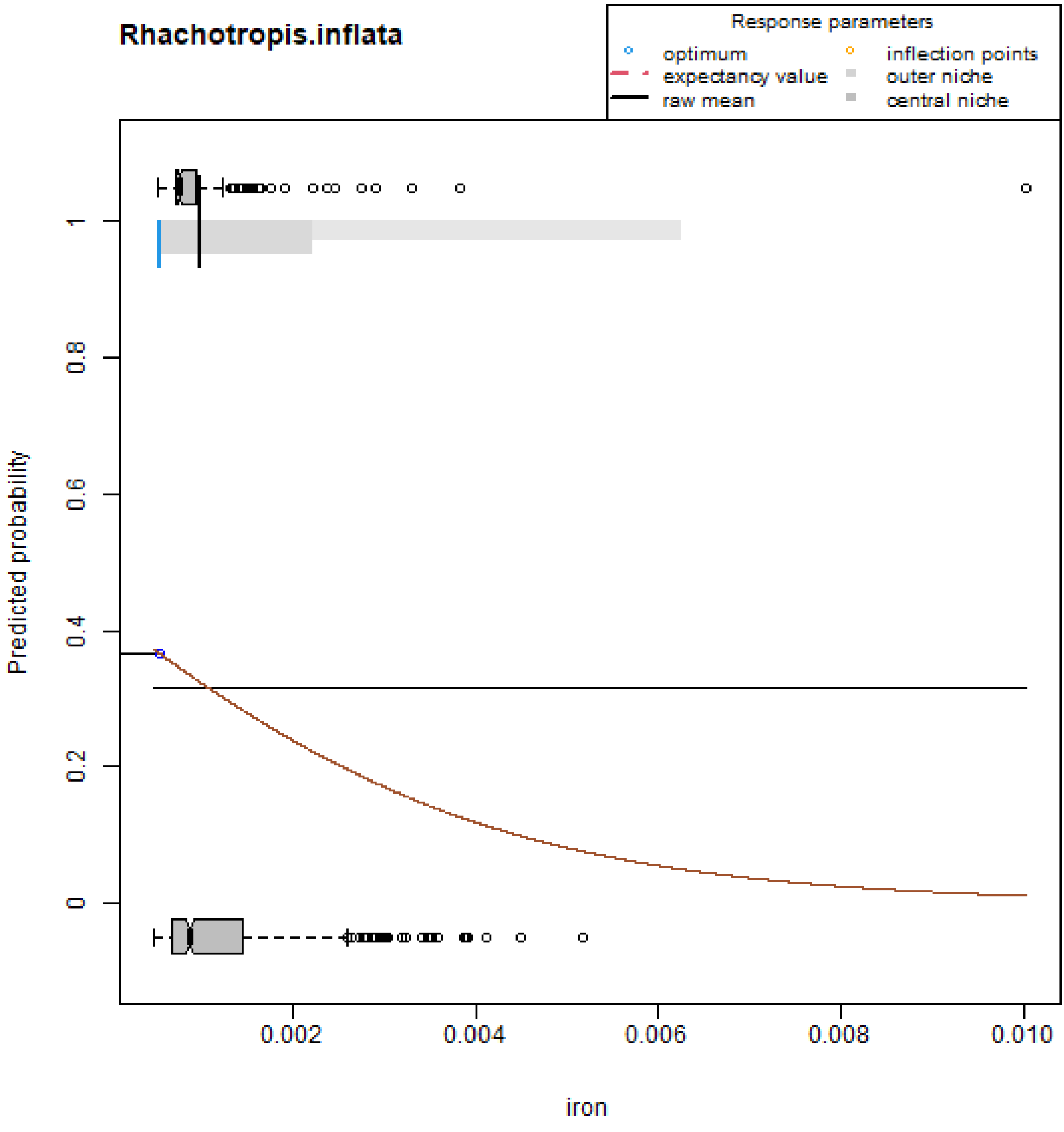

Rhachotropis.inflata
Response parameters
optimum
expectancy value
raw mean
inflection points
outer niche
central niche
Predicted probability
0
0.2
0.4
0.6
0.8
1
0.002
0.004
0.006
0.008
0.010
iron

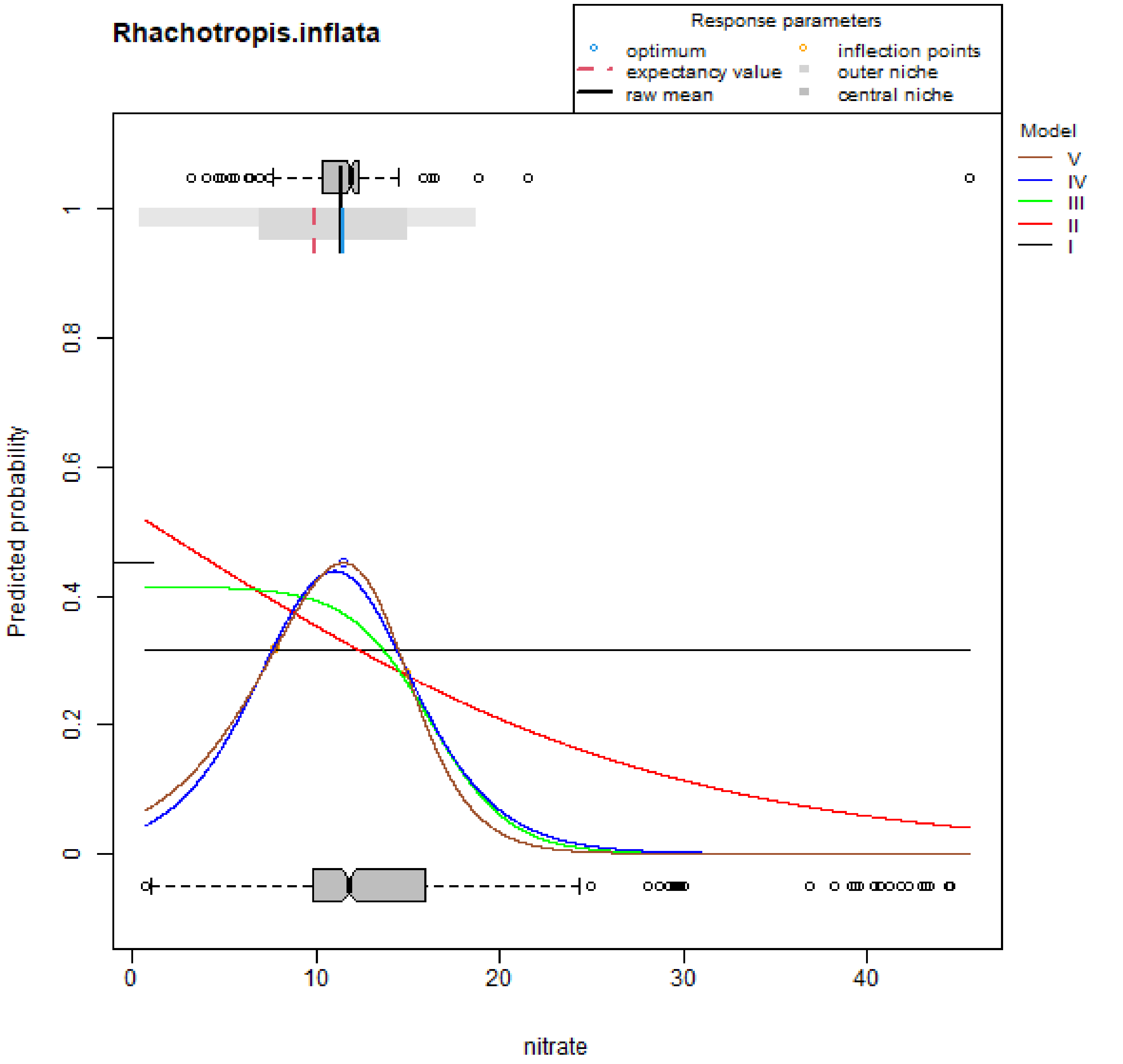
Rhachotropis.inflata
Response parameters
optimum
expectancy value
raw mean
inflection points
outer niche
central niche
Model
V
IV
III
II
I
Predicted probability
0
0.2
0.4
0.6
0.8
1
0
10
20
30
40
nitrate

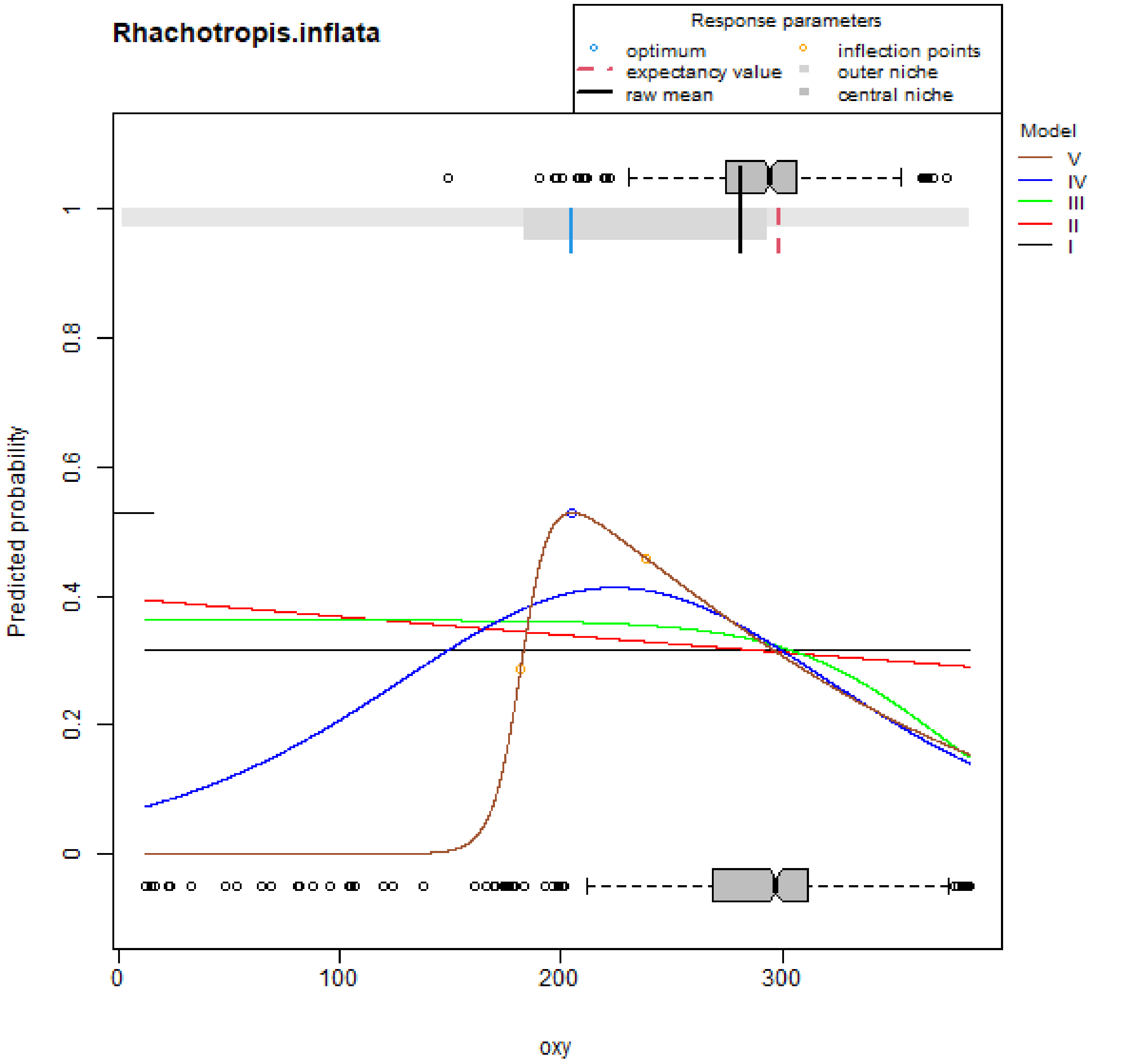
Rhachotropis.inflata
Response parameters
optimum
expectancy value
raw mean
inflection points
outer niche
central niche
Model
V
IV
III
II
I
Predicted probability
0
0.2
0.4
0.6
0.8
1
0
100
200
300
oxy

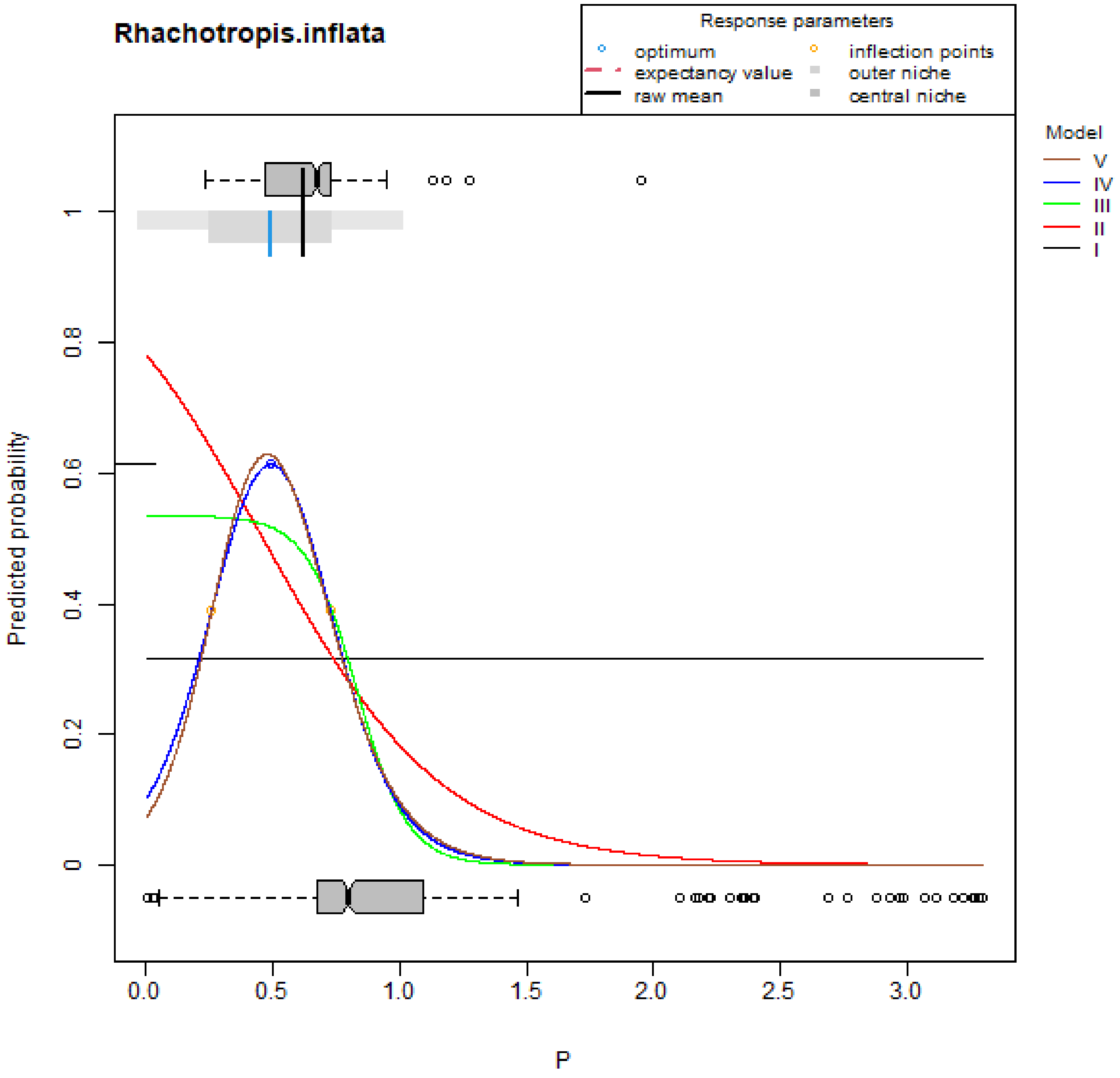
Rhachotropis.inflata
Response parameters
optimum
expectancy value
raw mean
inflection points
outer niche
central niche
Model
V
IV
III
II
I
Predicted probability
0
0.2
0.4
0.6
0.8
1
0.0
0.5
1.0
1.5
2.0
2.5
3.0
P

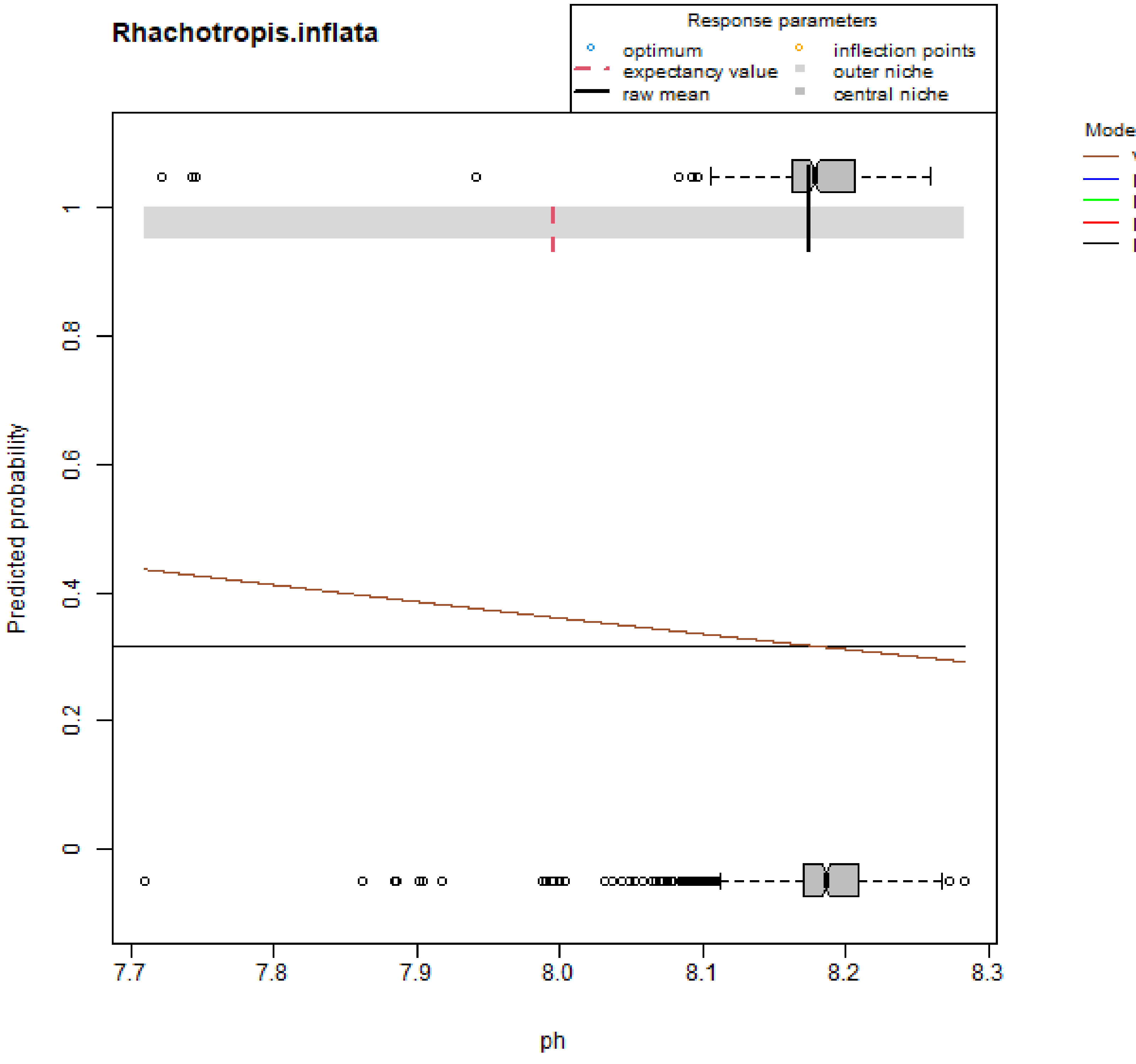
Rhachotropis.inflata
Response parameters
optimum
expectancy value
raw mean
inflection points
outer niche
central niche
Mode
Predicted probability
0
0.2
0.4
0.6
0.8
1
7.7
7.8
7.9
8.0
8.1
8.2
8.3
ph

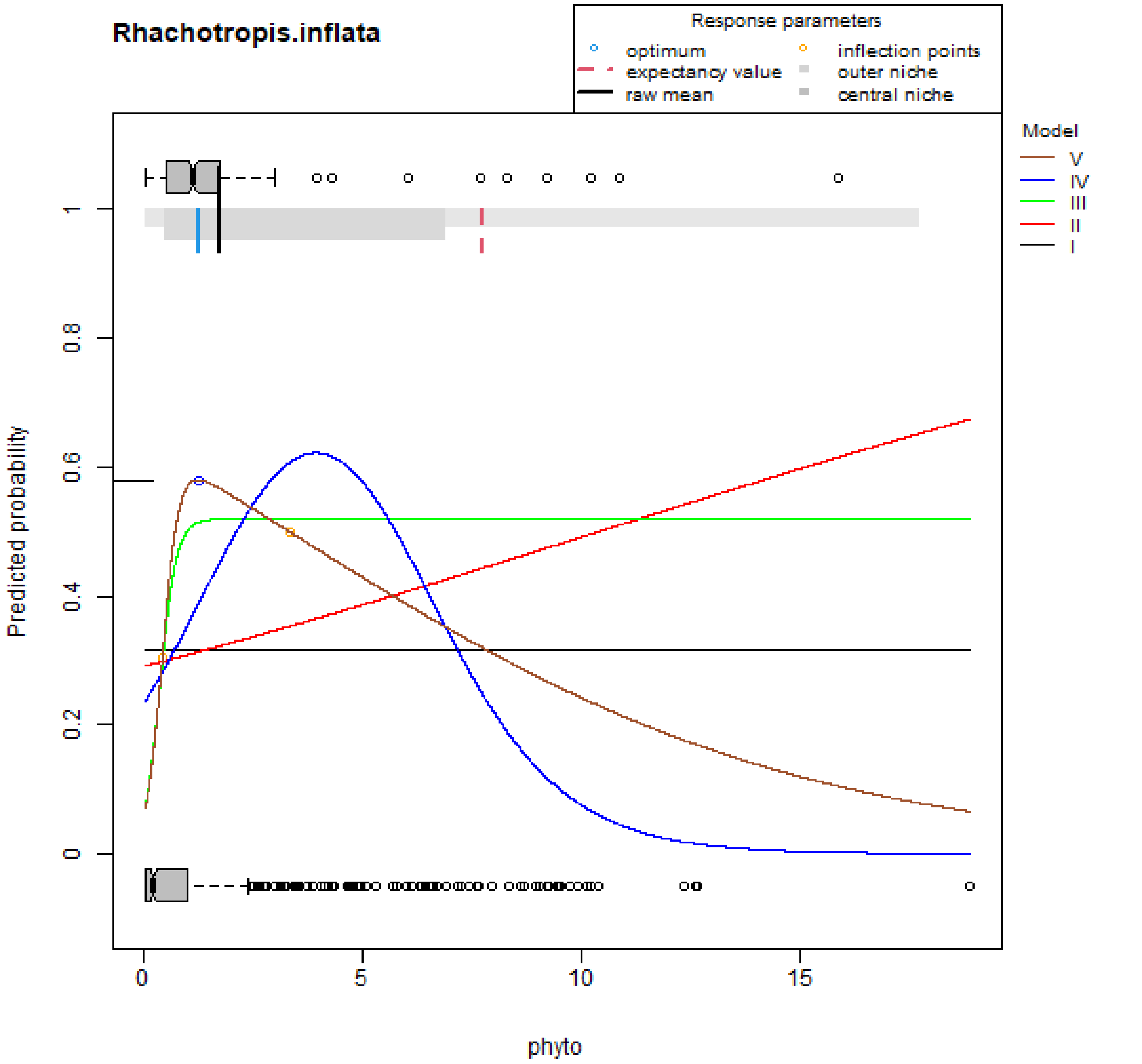

Rhachotropis.inflata
Response parameters
optimum
expectancy value
raw mean
inflection points
outer niche
central niche
Model
V
IV
III
II
I
Predicted probability
0
0.2
0.4
0.6
0.8
1
0
5
10
15
phyto

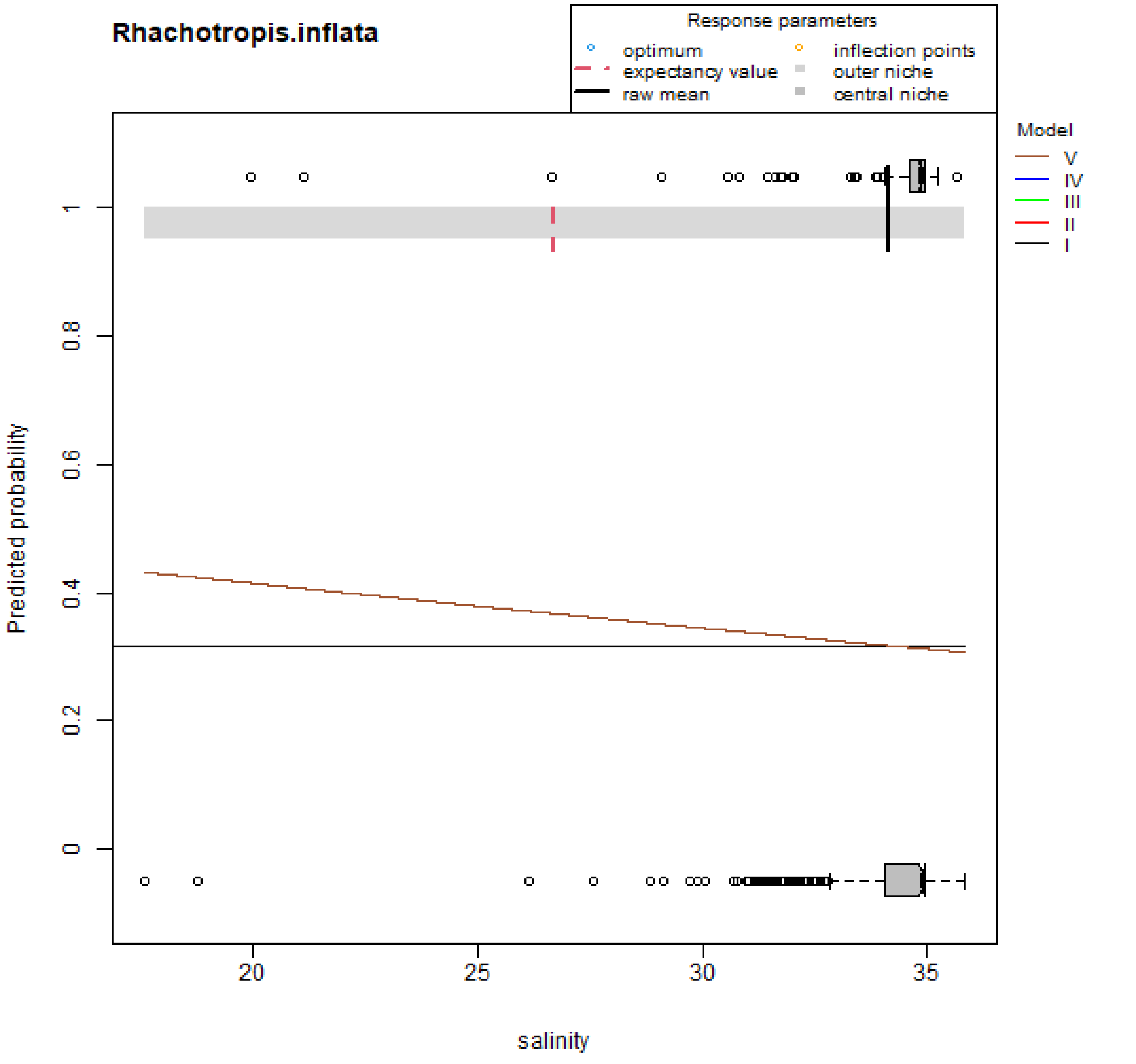
Rhachotropis.inflata
Response parameters
optimum
expectancy value
raw mean
inflection points
outer niche
central niche
Model
V
IV
III
II
I
Predicted probability
0
0.2
0.4
0.6
0.8
1
20
25
30
35
salinity

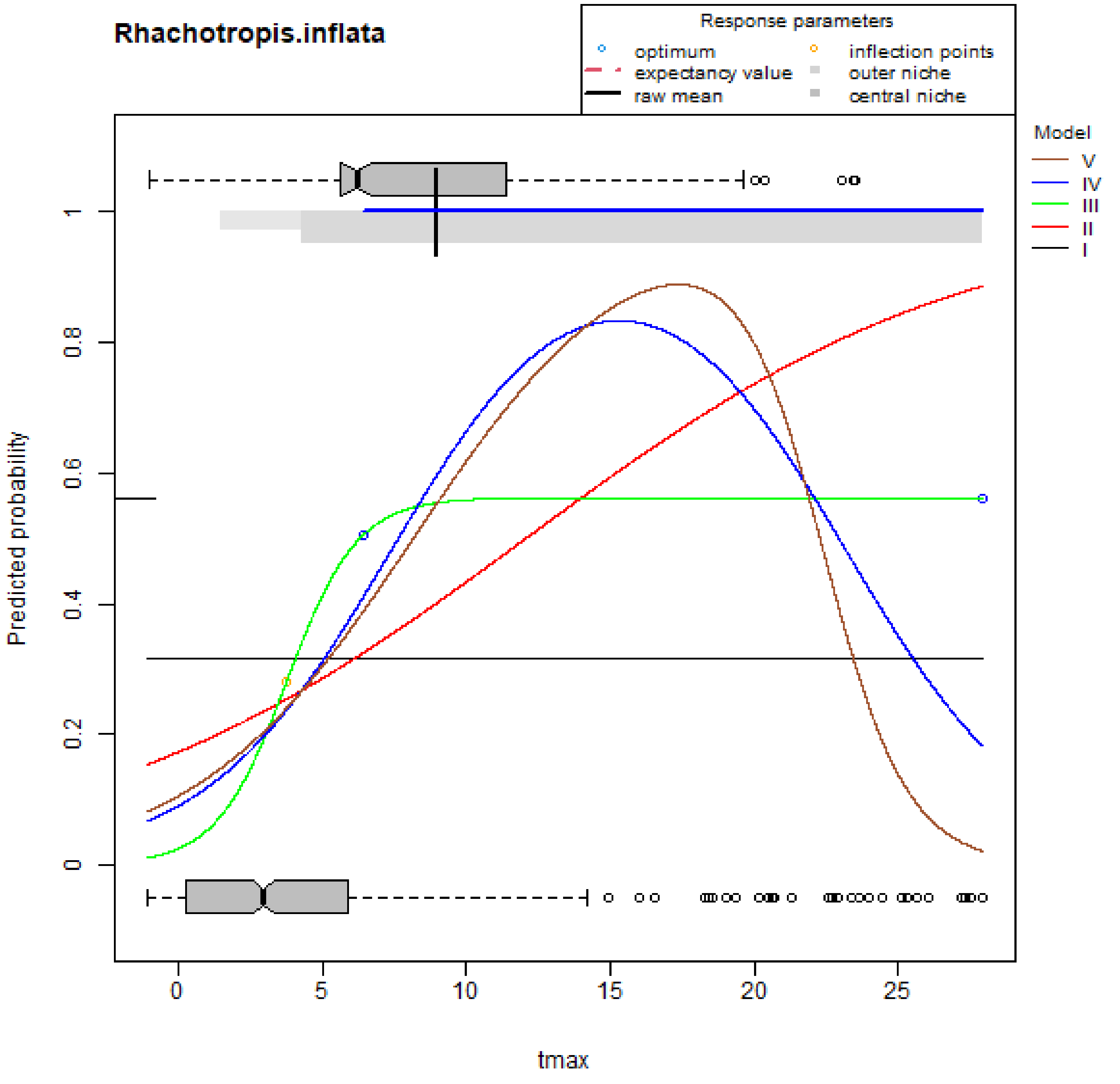
Rhachotropis.inflata
Response parameters
optimum
expectancy value
raw mean
inflection points
outer niche
central niche
Model
V
IV
III
II
I
Predicted probability
0
0.2
0.4
0.6
0.8
1
0
5
10
15
20
25
tmax

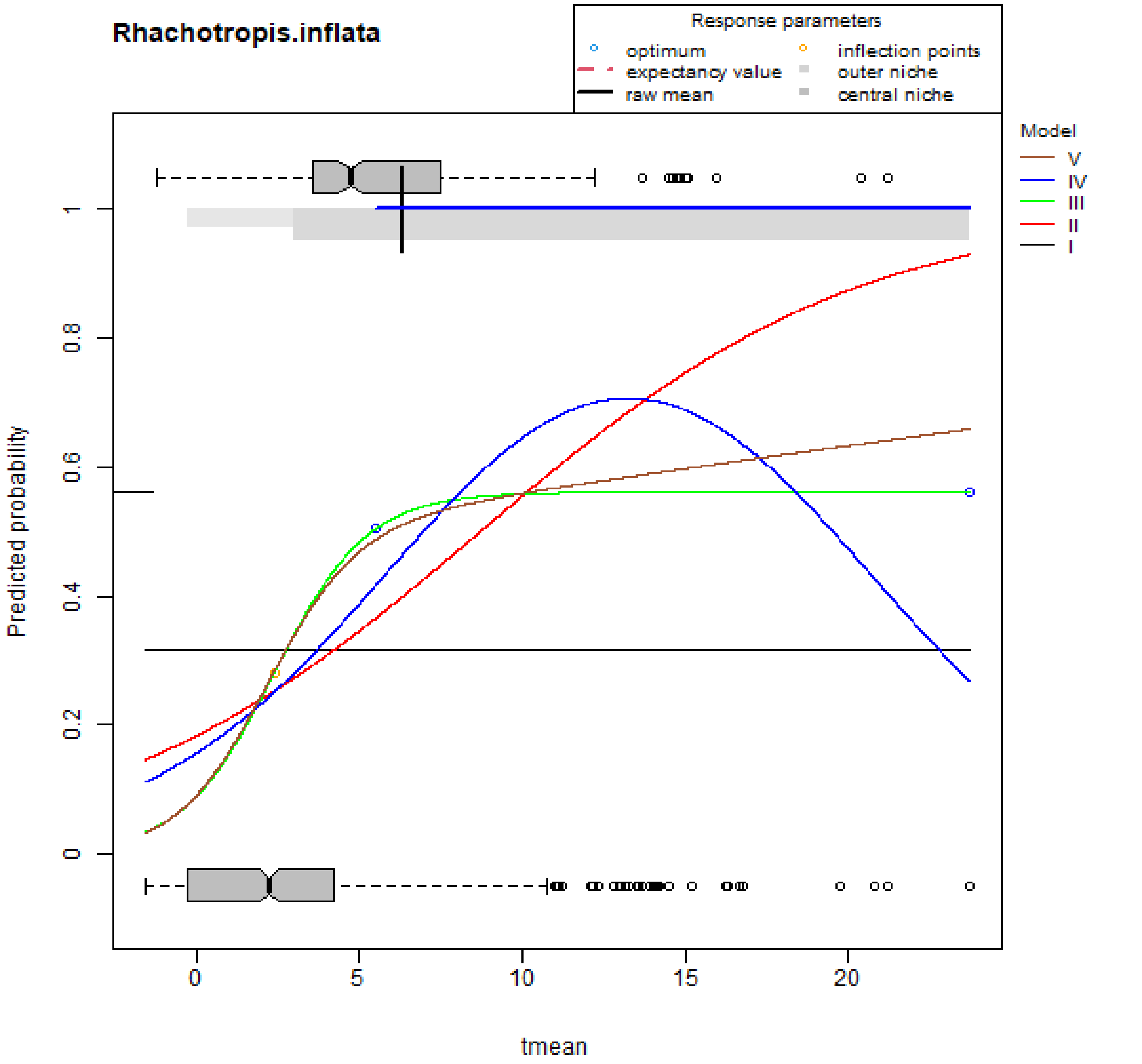

Rhachotropis.inflata
Response parameters
optimum
expectancy value
raw mean
inflection points
outer niche
central niche
Model
V
IV
III
II
I
Predicted probability
0
0.2
0.4
0.6
0.8
1
0
5
10
15
20
tmean

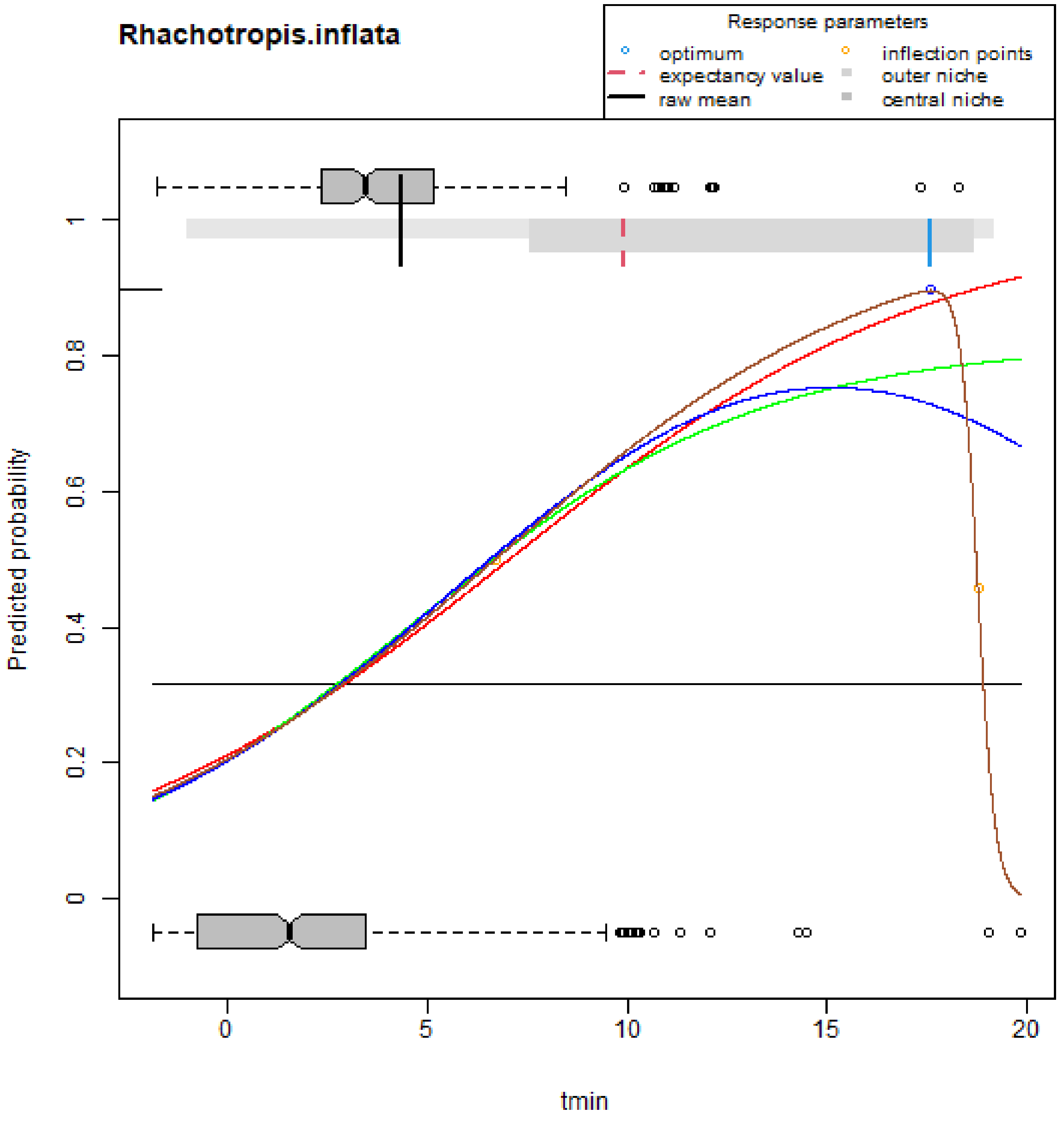

Rhachotropis.inflata
Response parameters
optimum
expectancy value
raw mean
inflection points
outer niche
central niche
Model
V
IV
III
II
I
Predicted probability
0
0.2
0.4
0.6
0.8
1
0
5
10
15
20
tmin

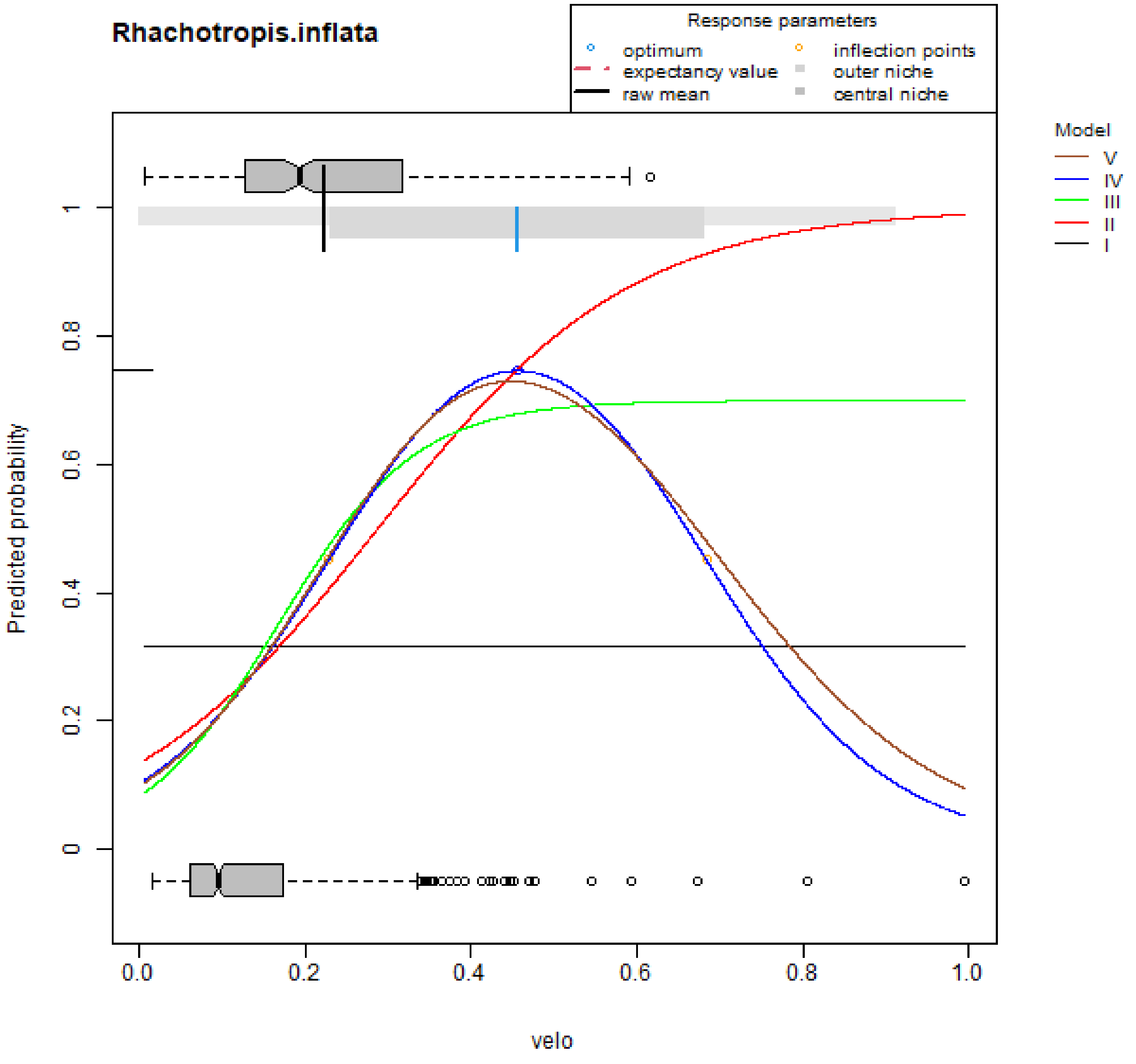
Rhachotropis.inflata
Response parameters
optimum
expectancy value
raw mean
inflection points
outer niche
central niche
Model
V
IV
III
II
I
Predicted probability
0
0.2
0.4
0.6
0.8
1
0.0
0.2
0.4
0.6
0.8
1.0
velo

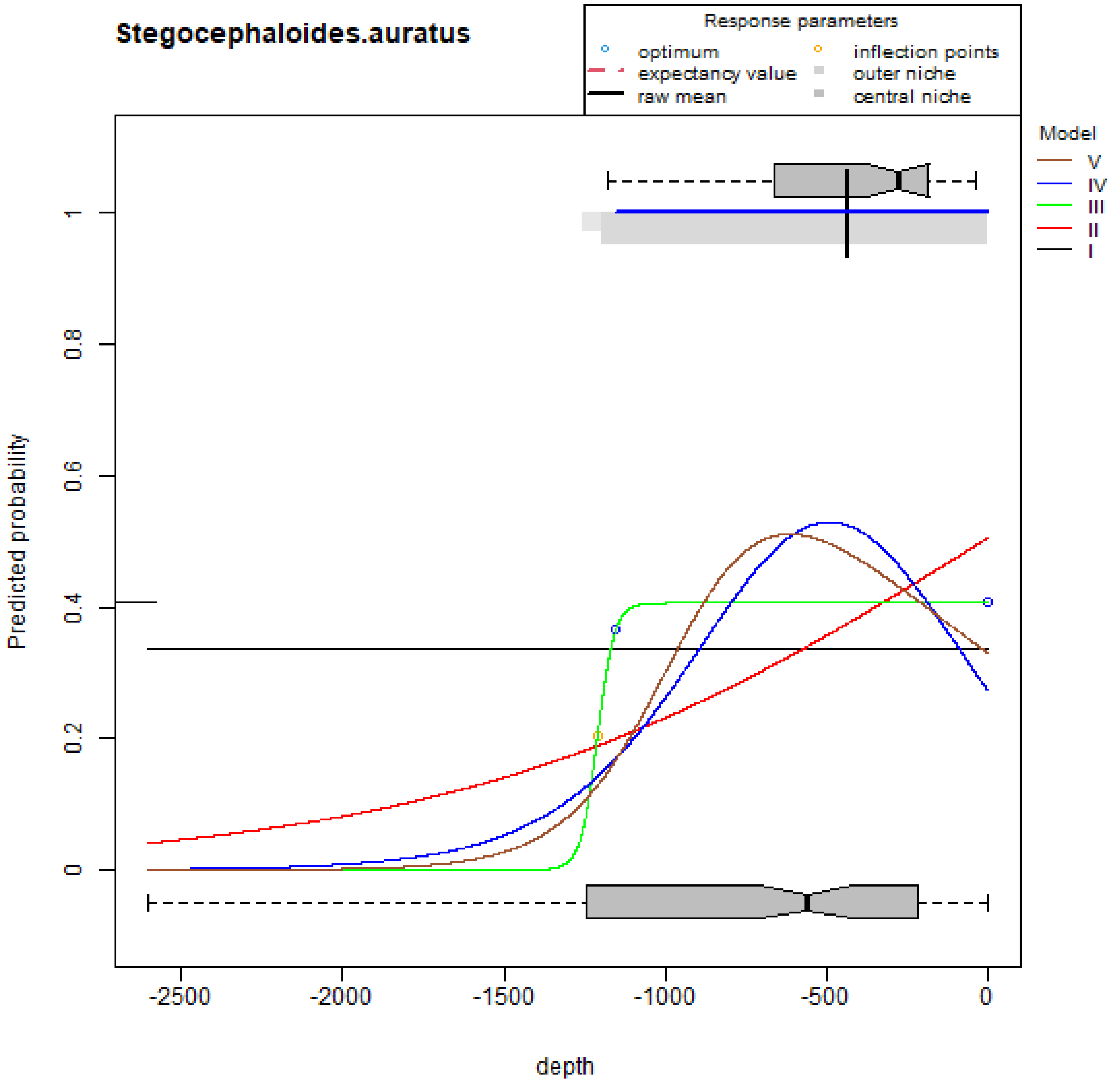
Stegocephaloides.auratus
Response parameters
optimum
expectancy value
raw mean
inflection points
outer niche
central niche
Model
V
IV
III
II
I
Predicted probability
0
0.2
0.4
0.6
0.8
1
-2500
-2000
-1500
-1000
-500
0
depth

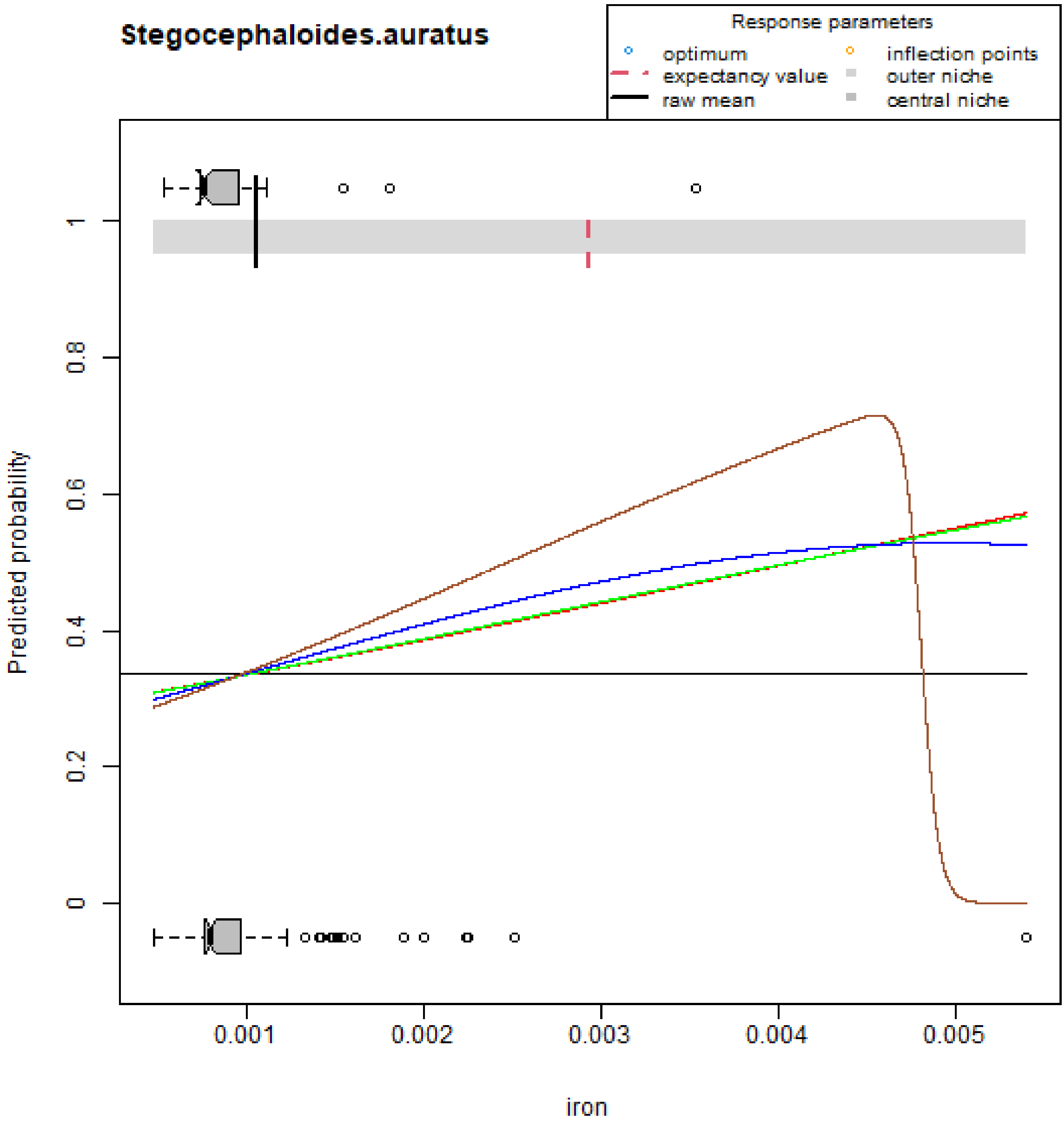

Stegocephaloides.auratus
Response parameters
optimum
expectancy value
raw mean
inflection points
outer niche
central niche
Predicted probability
0
0.2
0.4
0.6
0.8
1
0.001
0.002
0.003
0.004
0.005
iron

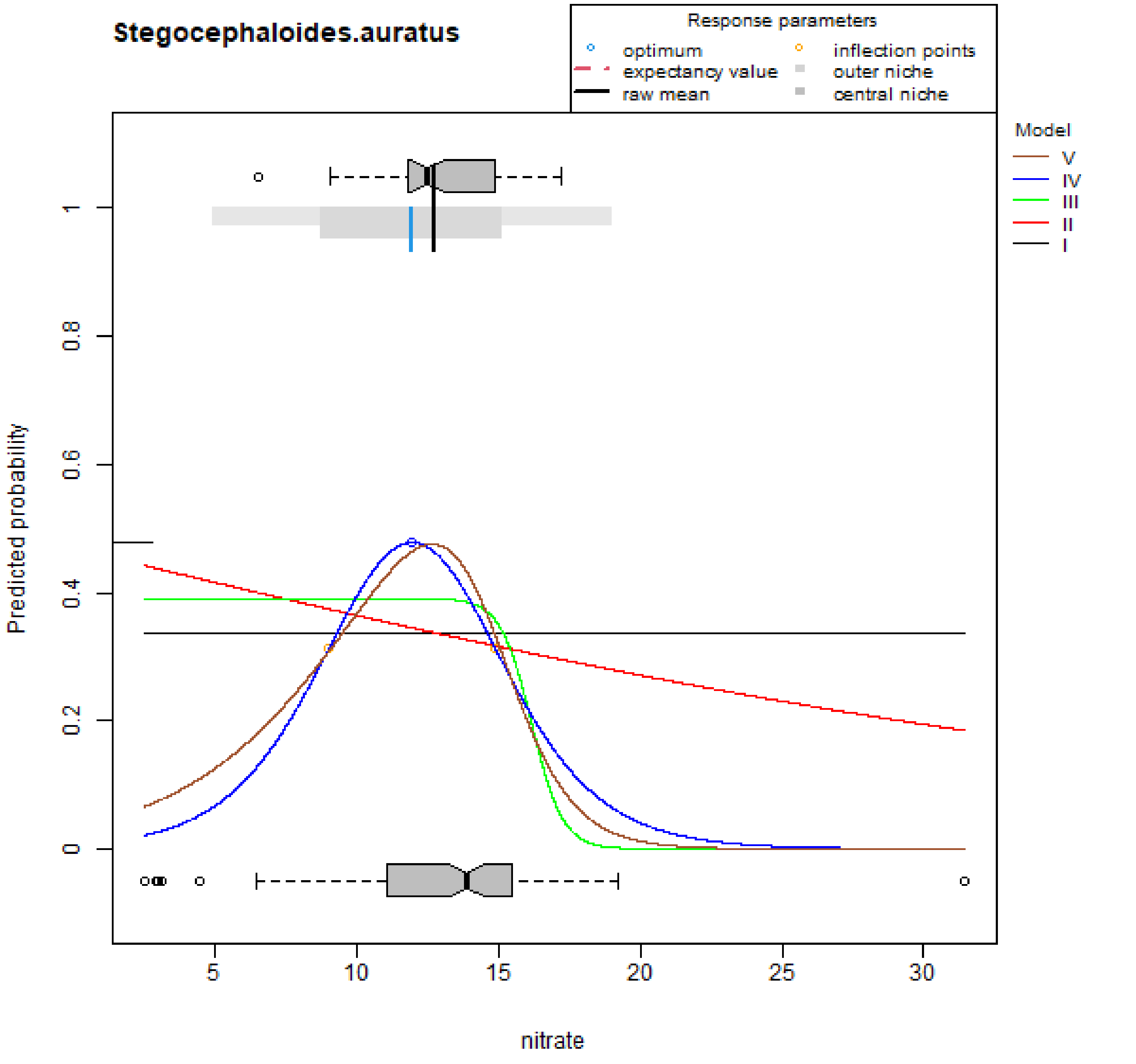

Stegocephaloides.auratus
Response parameters
optimum
expectancy value
raw mean
inflection points
outer niche
central niche
Model
V
IV
III
II
I
Predicted probability
0
0.2
0.4
0.6
0.8
1
5
10
15
20
25
30
nitrate

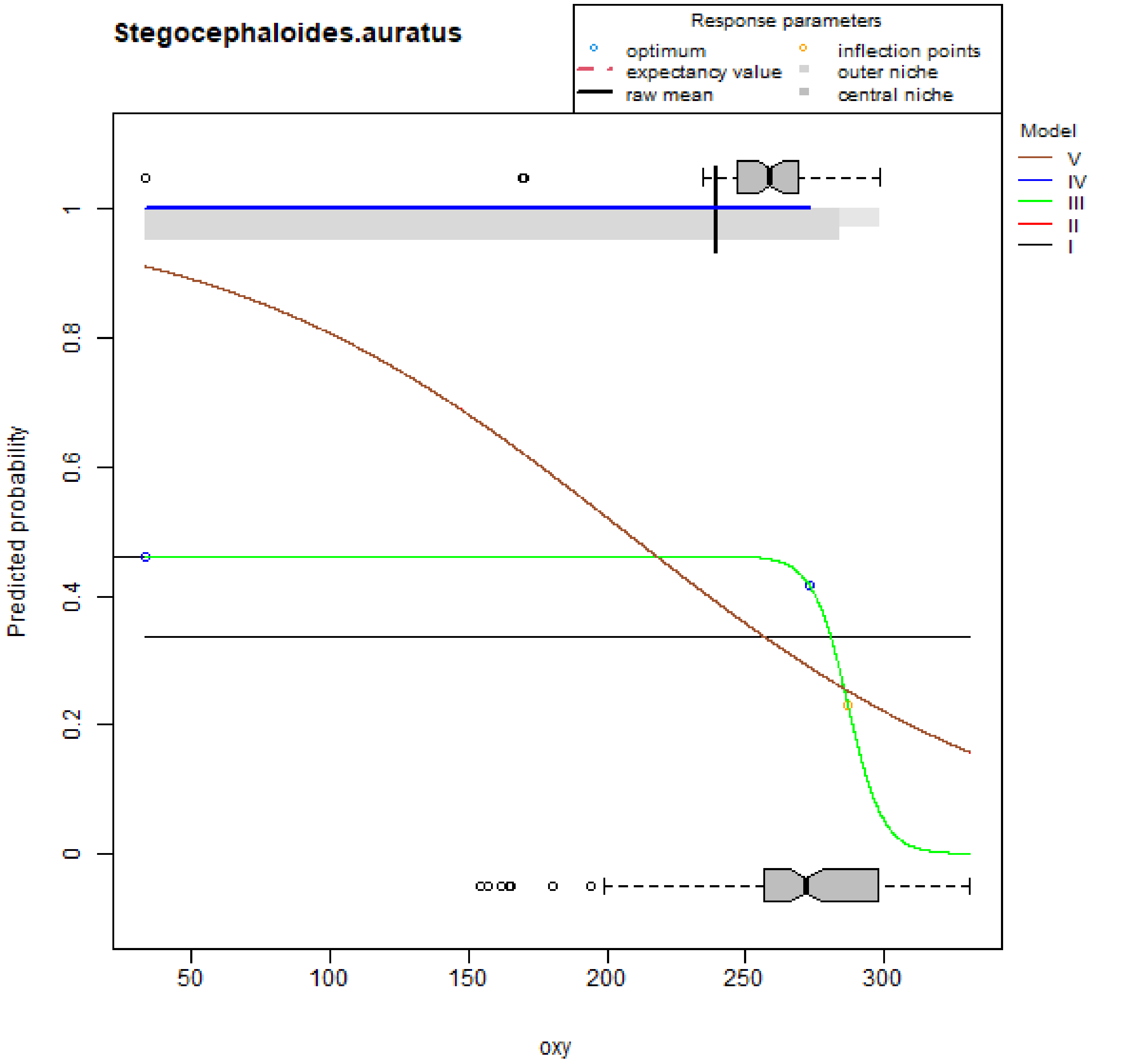

Stegocephaloides.auratus
Response parameters
optimum
expectancy value
raw mean
inflection points
outer niche
central niche
Model
V
IV
III
II
I
Predicted probability
0
0.2
0.4
0.6
0.8
1
50
100
150
200
250
300
oxy

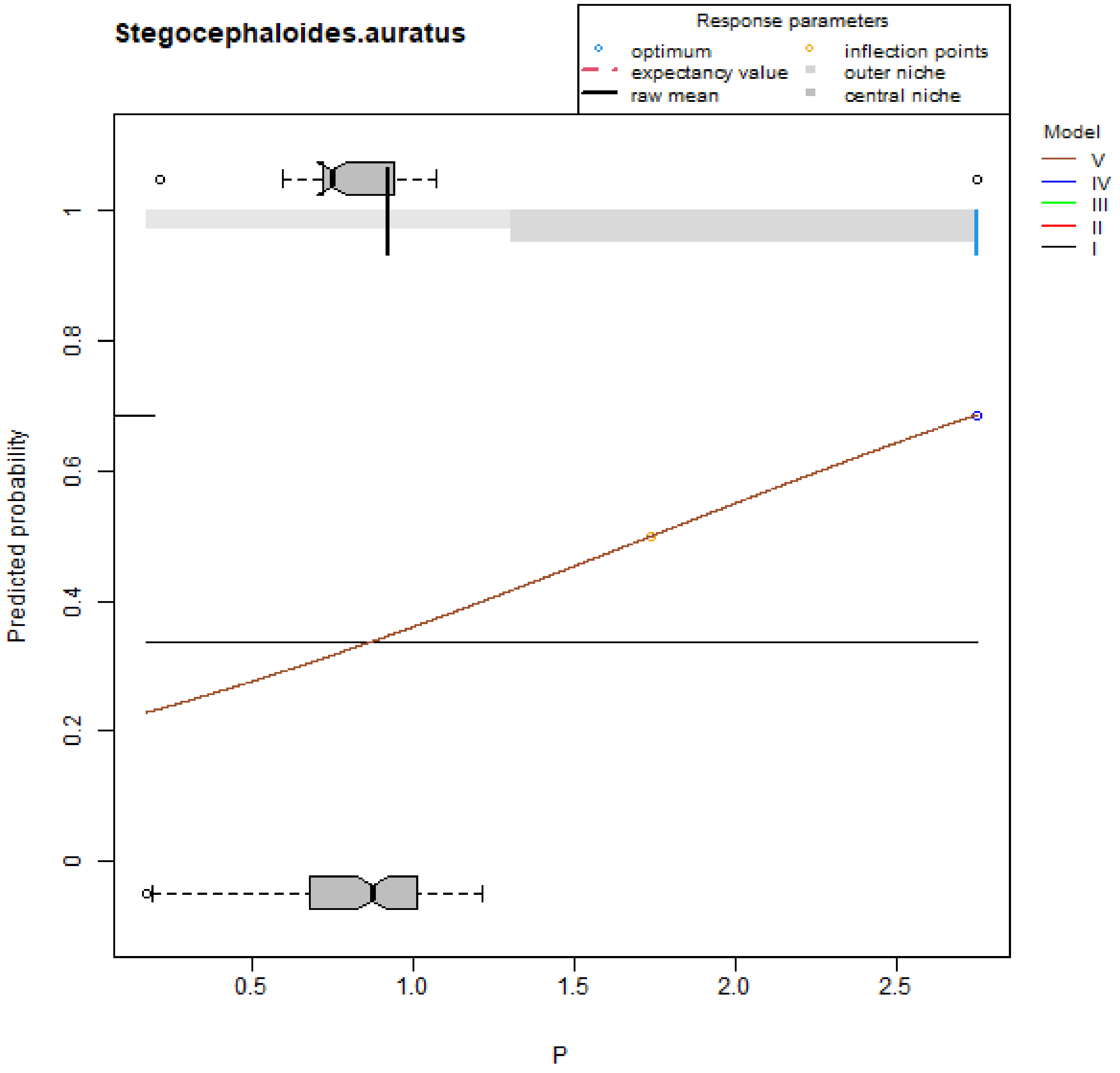

Stegocephaloides.auratus
Response parameters
optimum
expectancy value
raw mean
inflection points
outer niche
central niche
Model
V
IV
III
II
I
Predicted probability
0
0.2
0.4
0.6
0.8
1
0.5
1.0
1.5
2.0
2.5
P

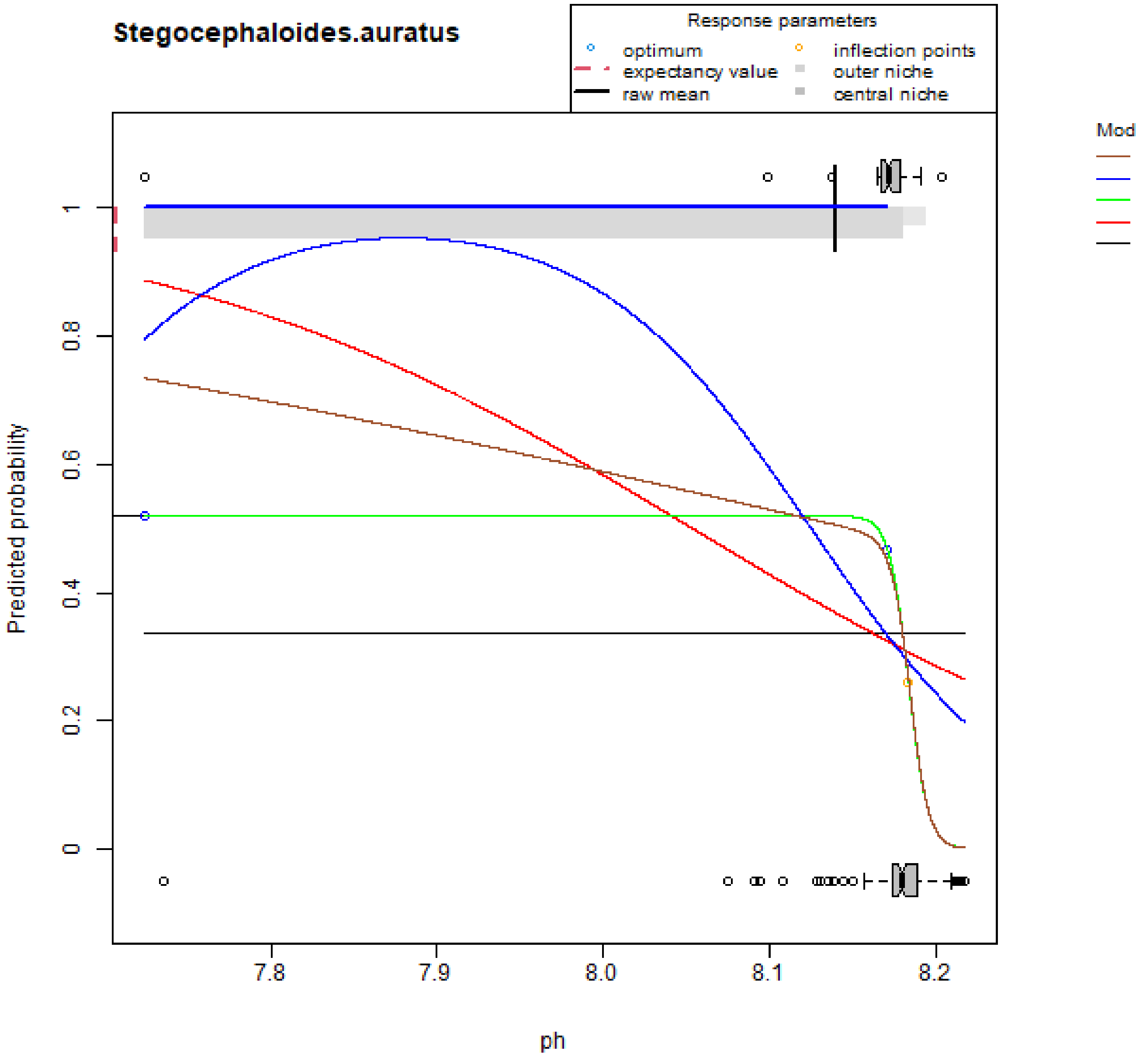

Stegocephaloides.auratus
Response parameters
optimum
expectancy value
raw mean
inflection points
outer niche
central niche
Mod
Predicted probability
0
0.2
0.4
0.6
0.8
1
7.8
7.9
8.0
8.1
8.2
ph

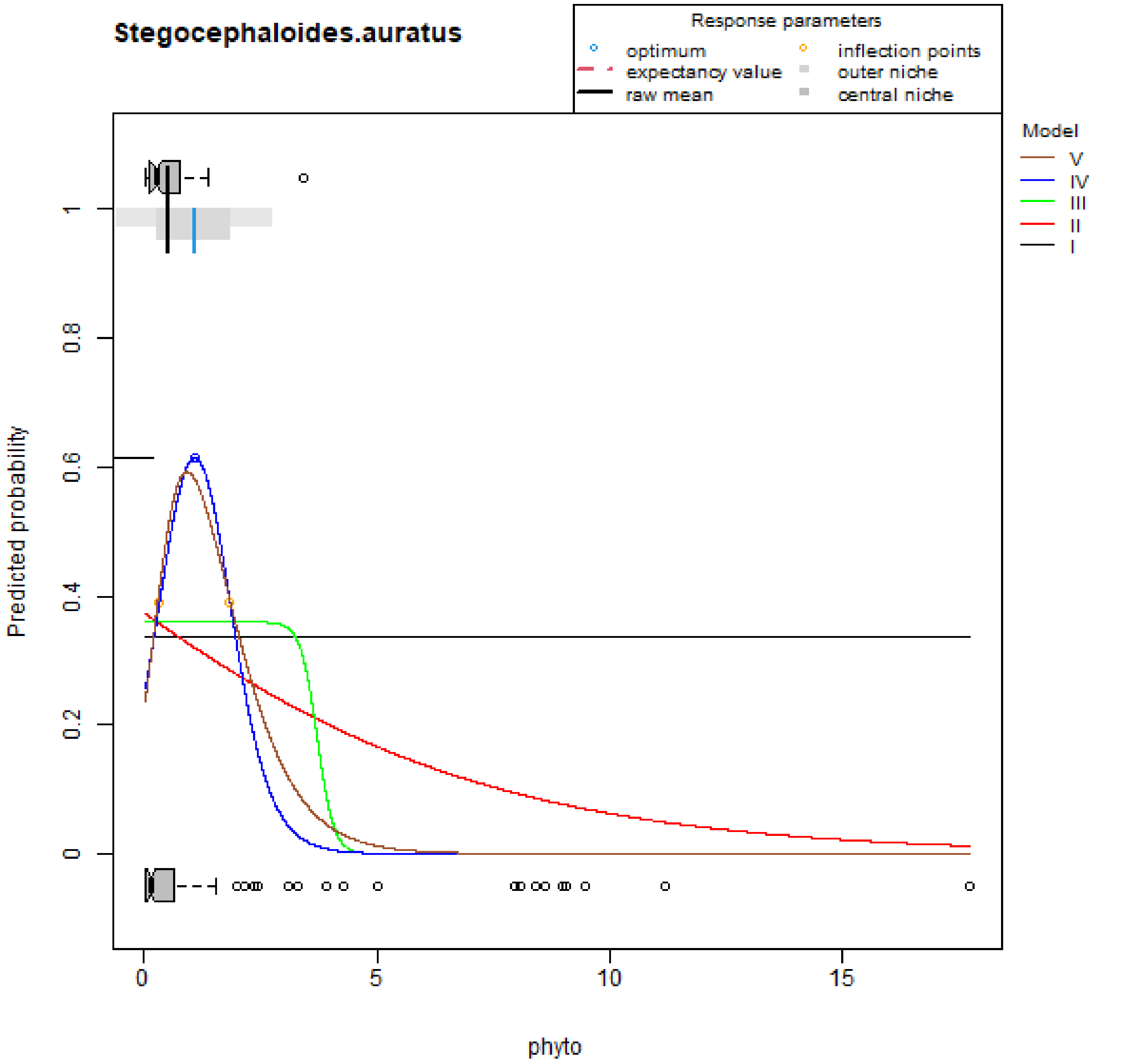

Stegocephaloides.auratus
Response parameters
optimum
inflection points
expectancy value
outer niche
raw mean
central niche
Model
V
IV
III
II
I
Predicted probability
0
0.2
0.4
0.6
0.8
1
0
5
10
15
phyto

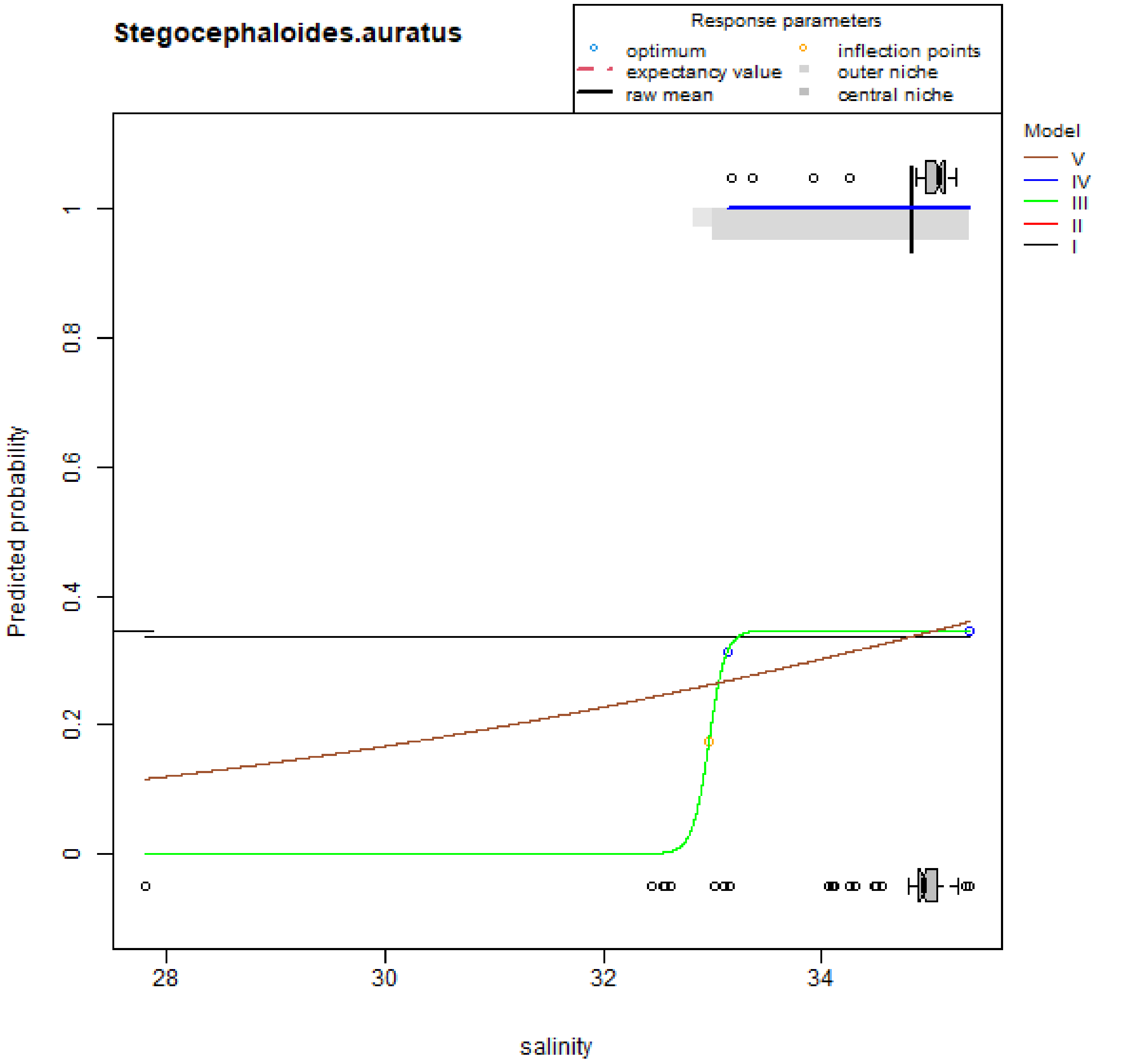
Stegocephaloides.auratus
Response parameters
optimum
expectancy value
raw mean
inflection points
outer niche
central niche
Model
V
IV
III
II
I
Predicted probability
0
0.2
0.4
0.6
0.8
1
28
30
32
34
salinity

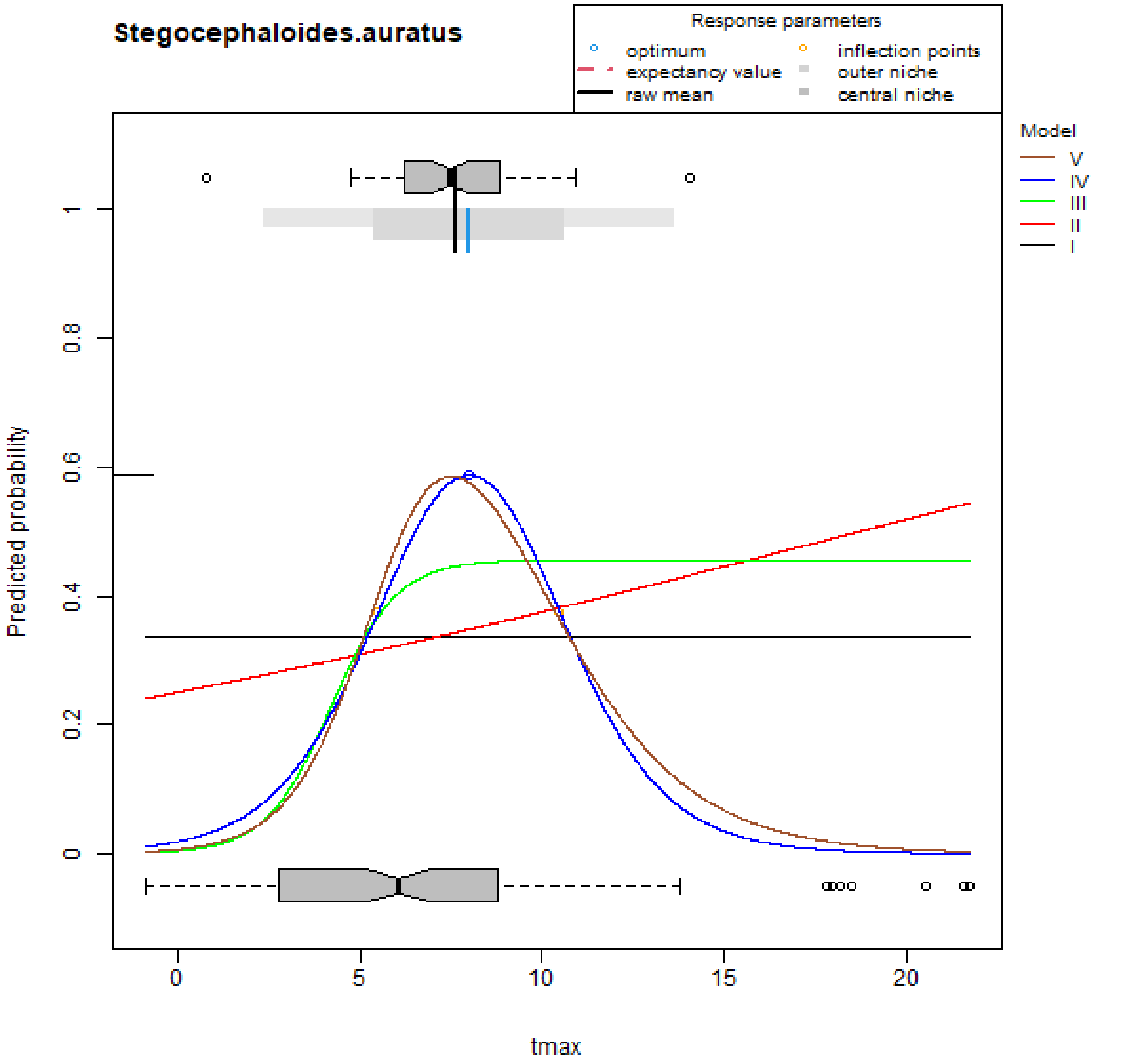

Stegocephaloides.auratus
Response parameters
optimum
expectancy value
raw mean
inflection points
outer niche
central niche
Model
V
IV
III
II
I
Predicted probability
0
0.2
0.4
0.6
0.8
1
0
5
10
15
20
tmax

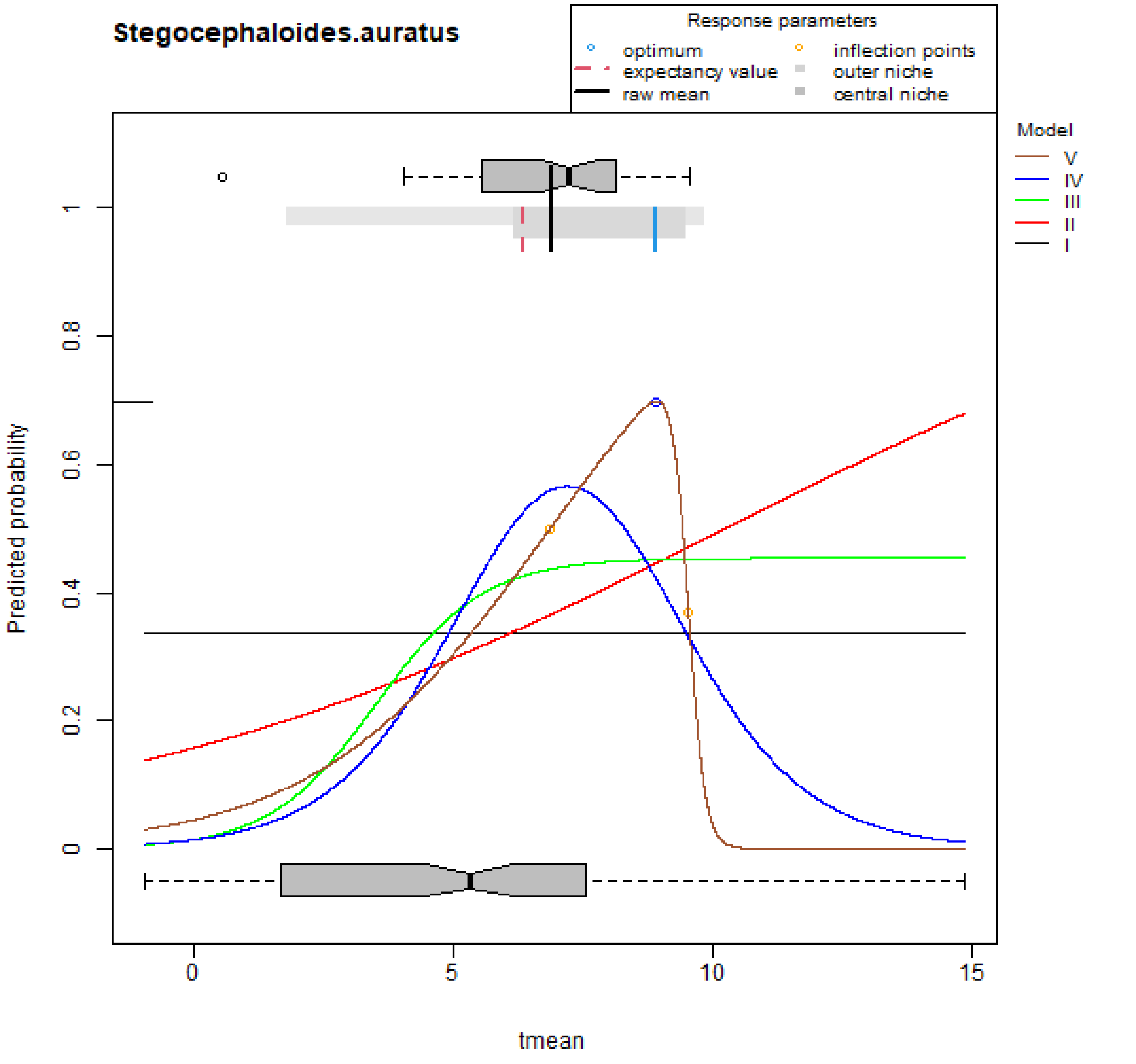

Stegocephaloides.auratus
Response parameters
optimum
expectancy value
raw mean
inflection points
outer niche
central niche
Model
V
IV
III
II
I
Predicted probability
0
0.2
0.4
0.6
0.8
1
0
5
10
15
tmean

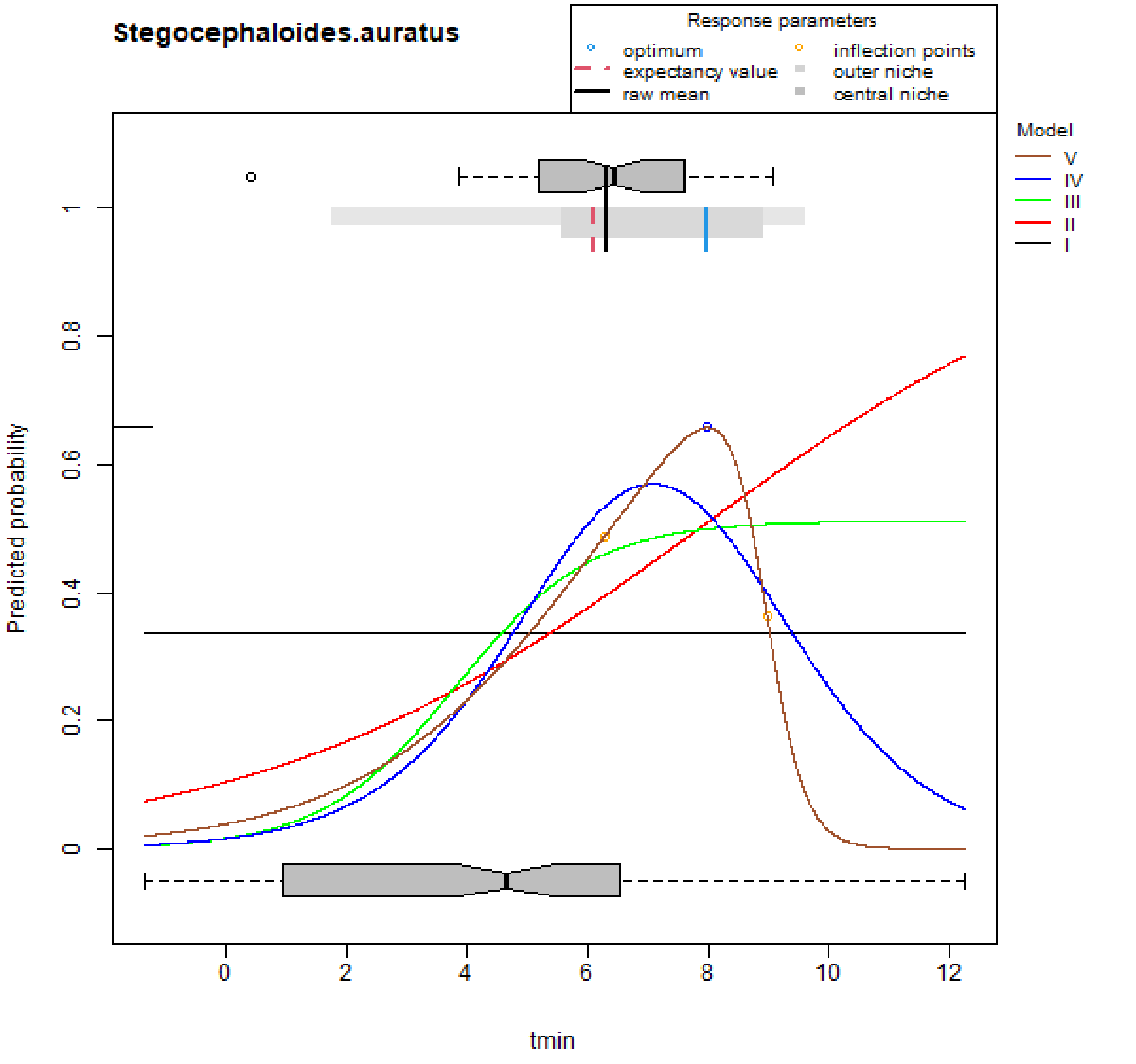

Stegocephaloides.auratus
Response parameters
optimum
expectancy value
raw mean
inflection points
outer niche
central niche
Model
V
IV
III
II
I
Predicted probability
0
0.2
0.4
0.6
0.8
1
0
2
4
6
8
10
12
tmin

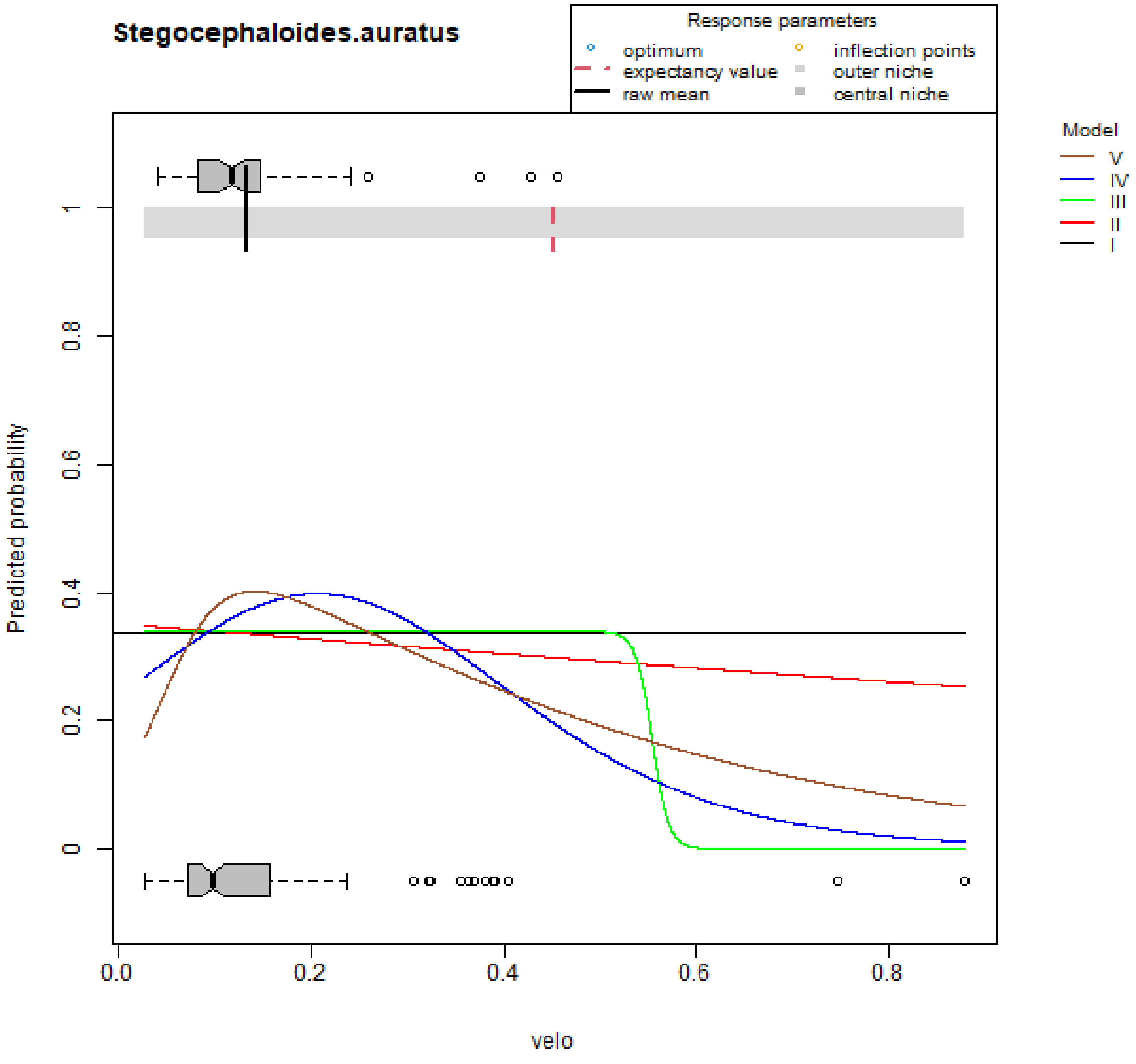
Stegocephaloides.auratus
Response parameters
optimum
expectancy value
raw mean
inflection points
outer niche
central niche
Model
V
IV
III
II
I
Predicted probability
0
0.2
0.4
0.6
0.8
1
0.0
0.2
0.4
0.6
0.8
velo

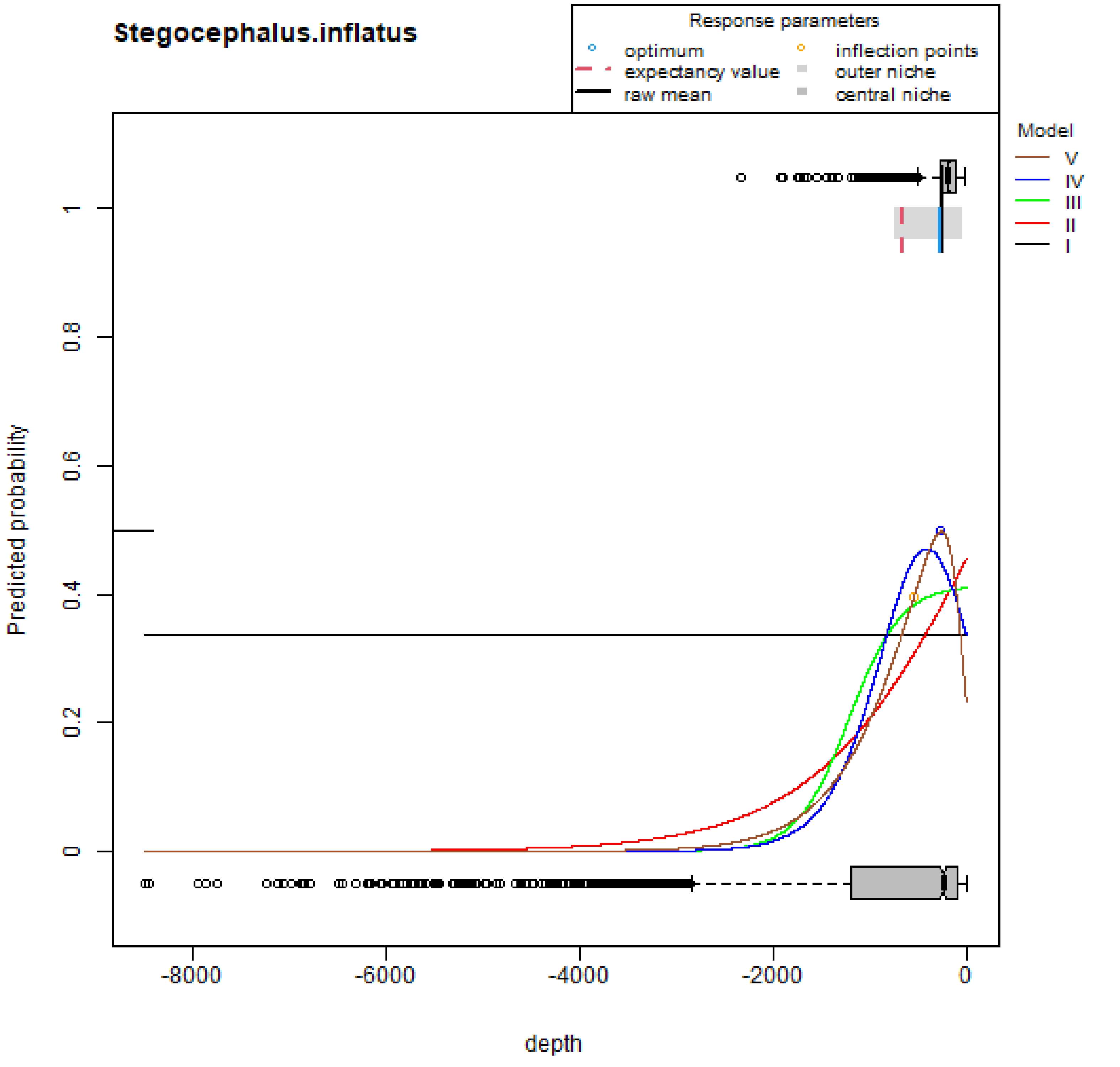

Stegocephalus.inflatus
Response parameters
optimum
expectancy value
raw mean
inflection points
outer niche
central niche
Model
V
IV
III
II
I
Predicted probability
0
0.2
0.4
0.6
0.8
1
-8000
-6000
-4000
-2000
0
depth

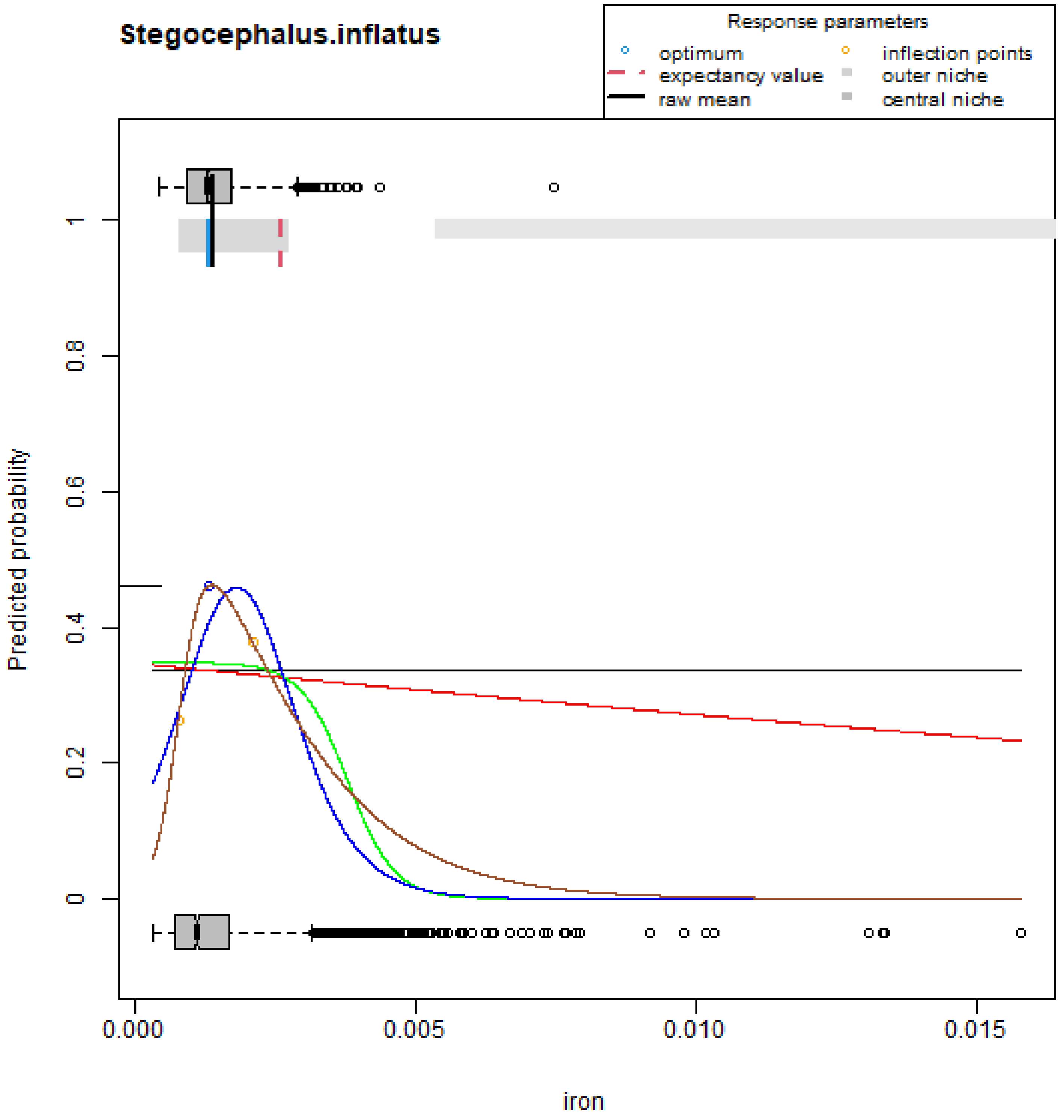
Stegocephalus.inflatus
Response parameters
optimum
expectancy value
raw mean
inflection points
outer niche
central niche
Predicted probability
0
0.2
0.4
0.6
0.8
1
0.000
0.005
0.010
0.015
iron

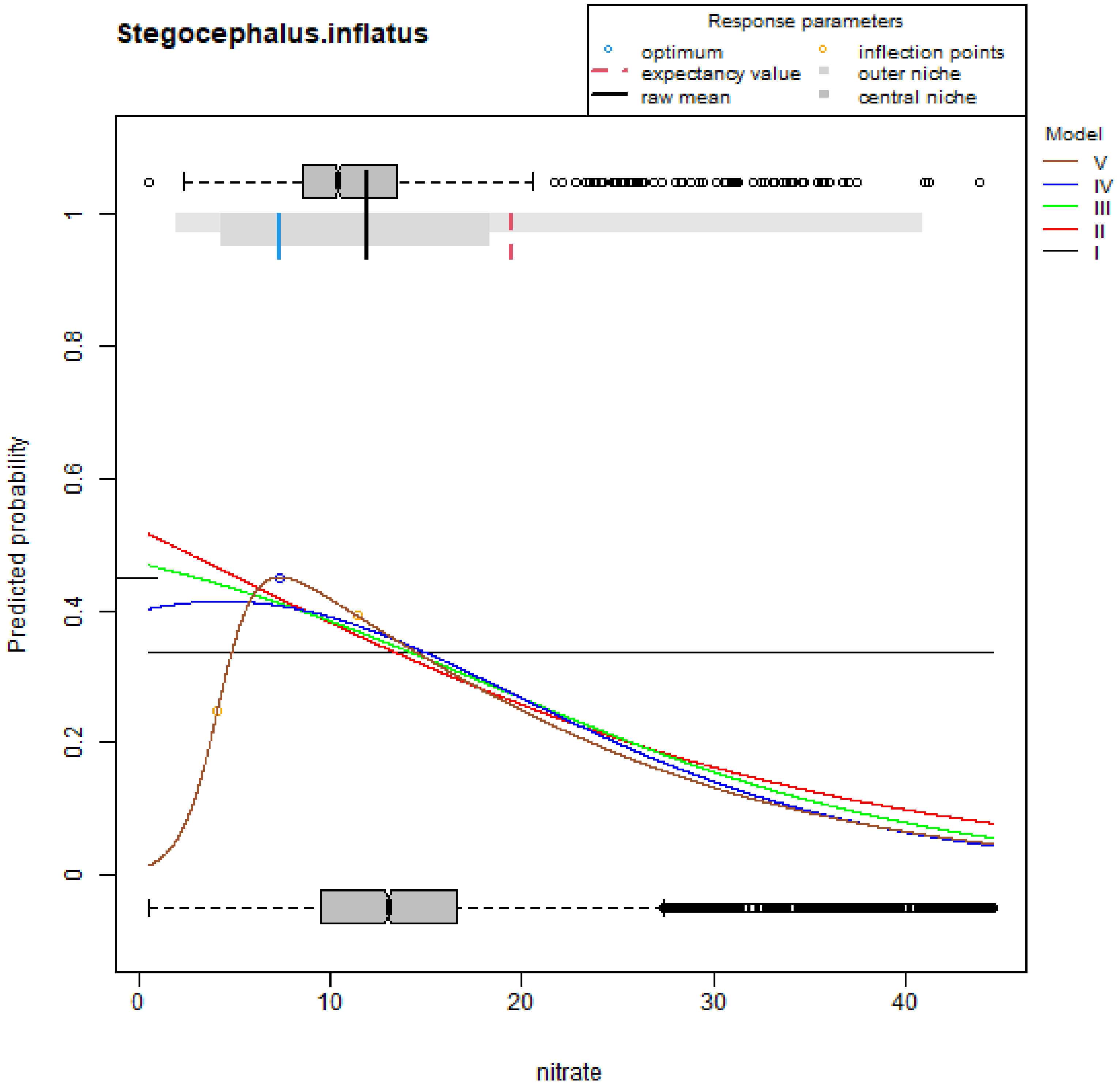

Stegocephalus.inflatus
Response parameters
optimum
expectancy value
raw mean
inflection points
outer niche
central niche
Model
V
IV
III
II
I
Predicted probability
0
0.2
0.4
0.6
0.8
1
0
10
20
30
40
nitrate

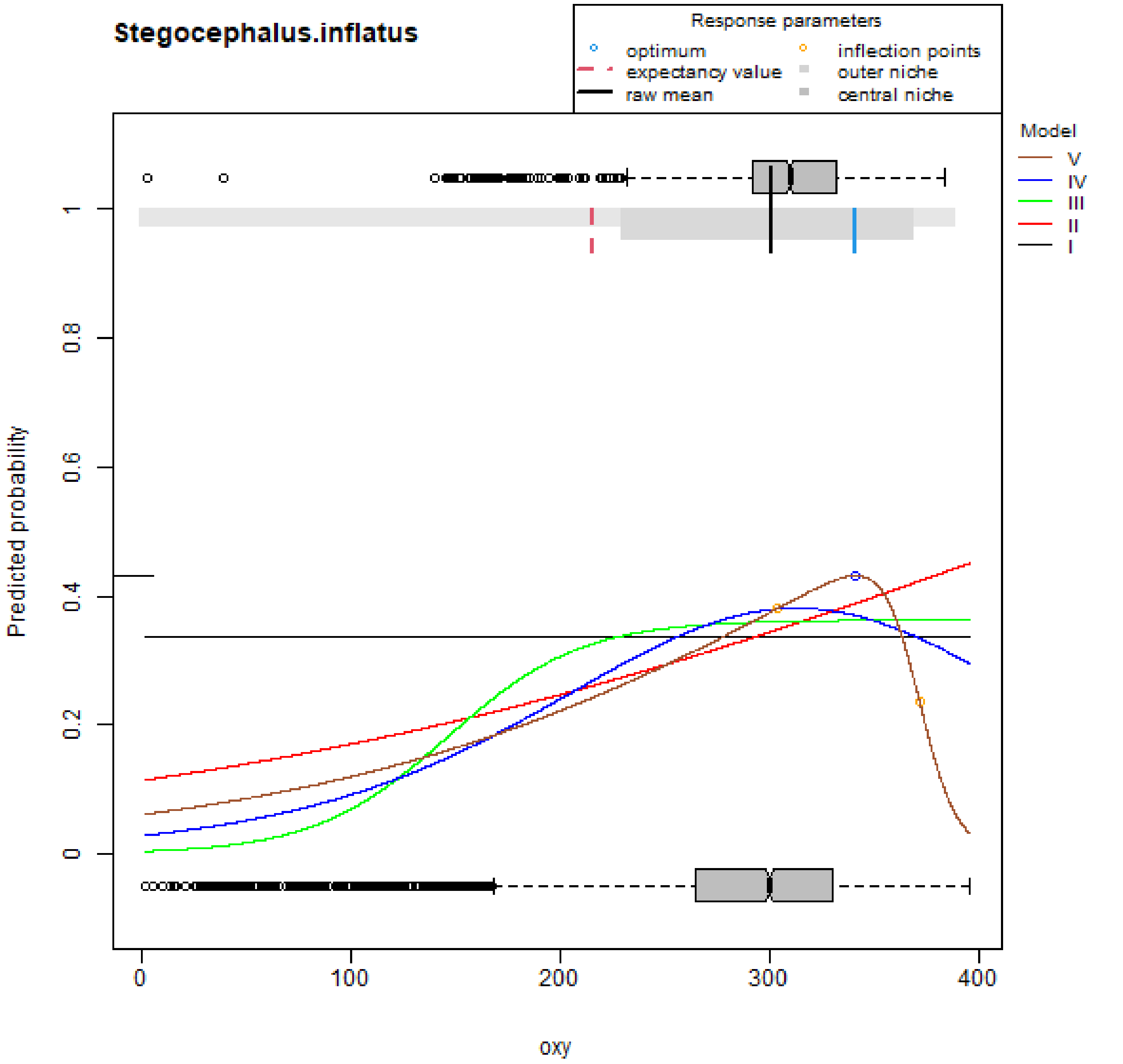

Stegocephalus.inflatus
Response parameters
optimum
expectancy value
raw mean
inflection points
outer niche
central niche
Model
V
IV
III
II
I
Predicted probability
0
0.2
0.4
0.6
0.8
1
0
100
200
300
400
oxy

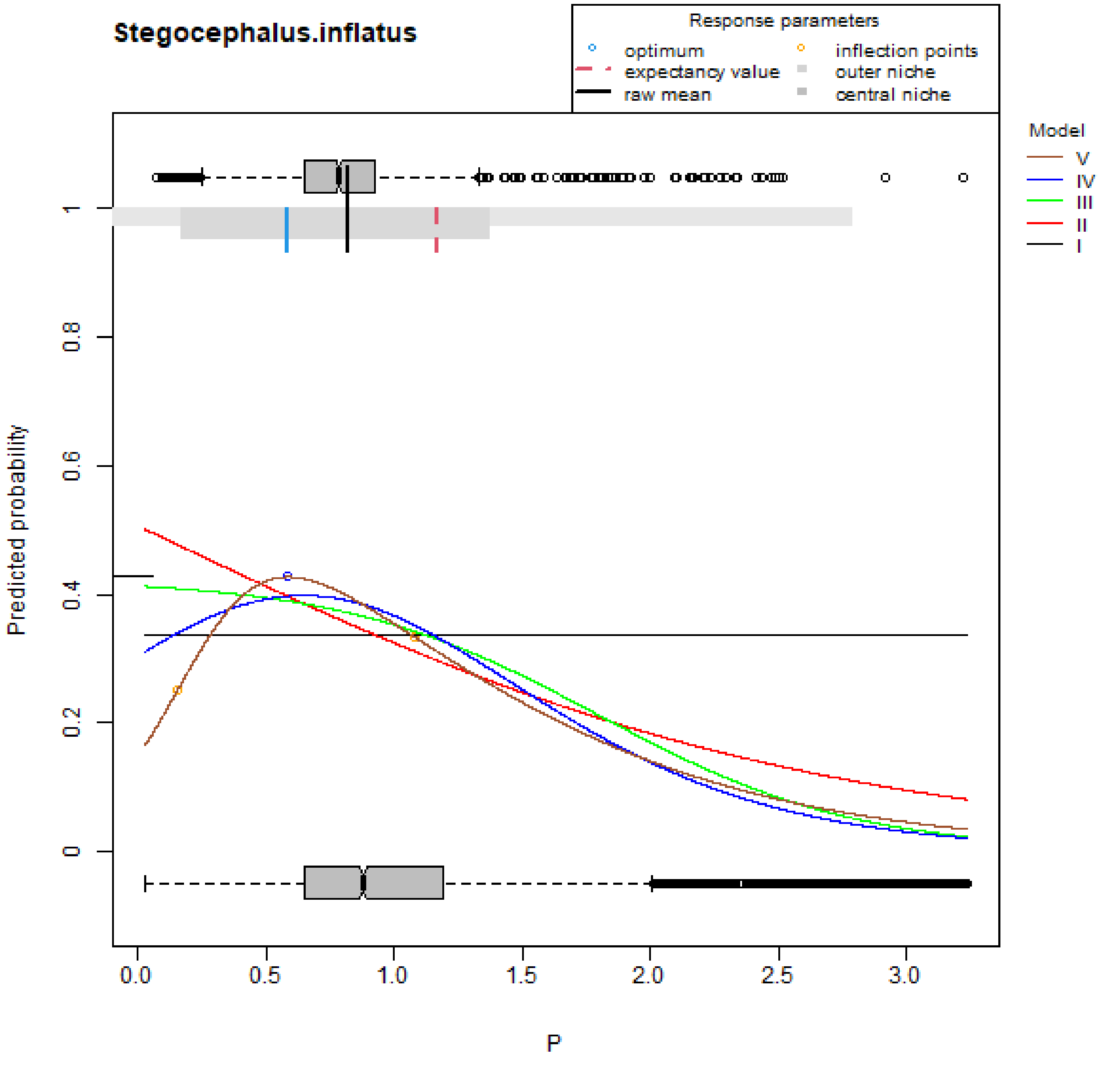

Stegocephalus.inflatus
Response parameters
optimum
expectancy value
raw mean
inflection points
outer niche
central niche
Model
V
IV
III
II
I
Predicted probability
0
0.2
0.4
0.6
0.8
1
0.0
0.5
1.0
1.5
2.0
2.5
3.0
P

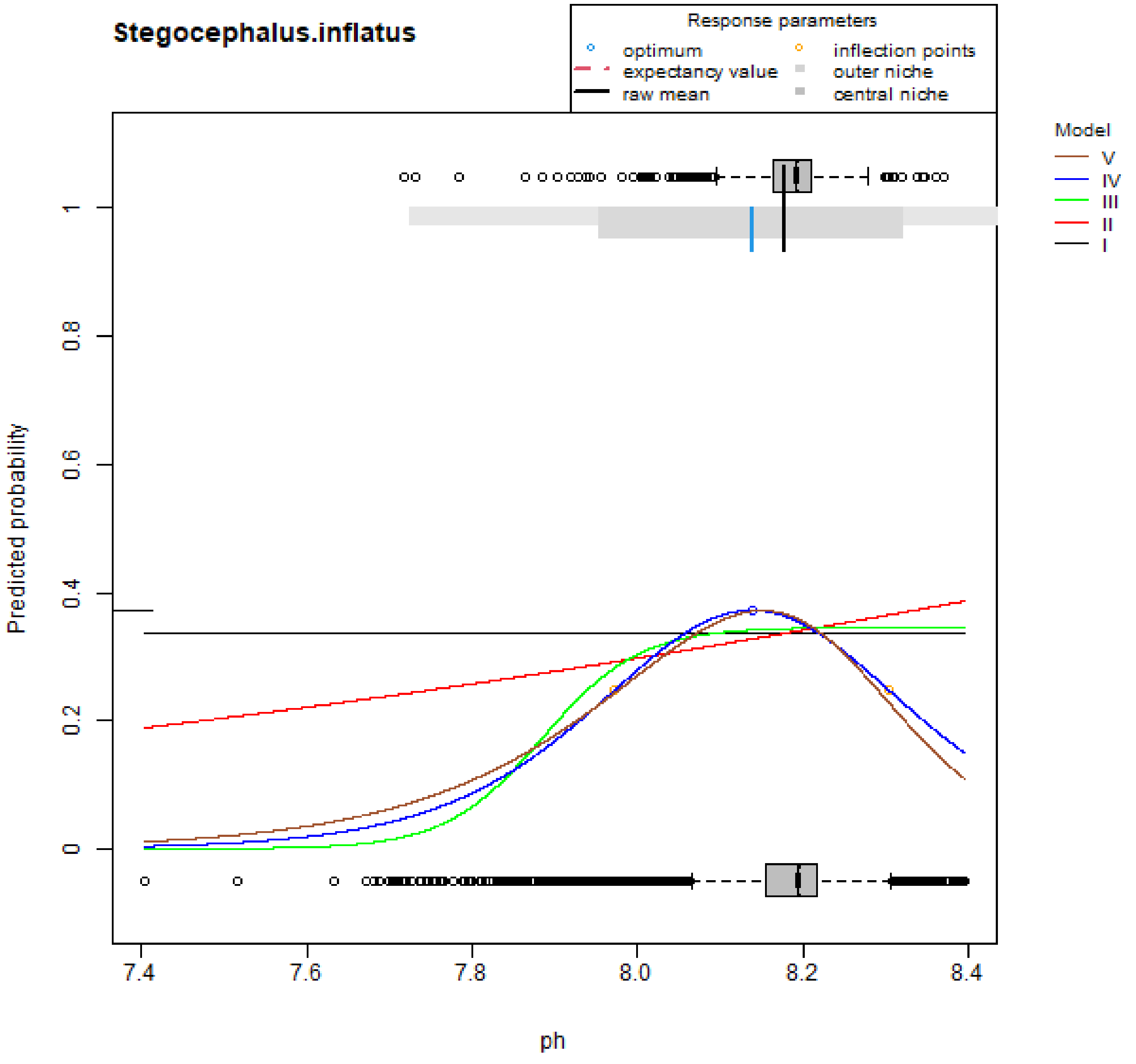
Stegocephalus.inflatus
Response parameters
optimum
expectancy value
raw mean
inflection points
outer niche
central niche
Model
V
IV
III
II
I
Predicted probability
0
0.2
0.4
0.6
0.8
1
7.4
7.6
7.8
8.0
8.2
8.4
ph

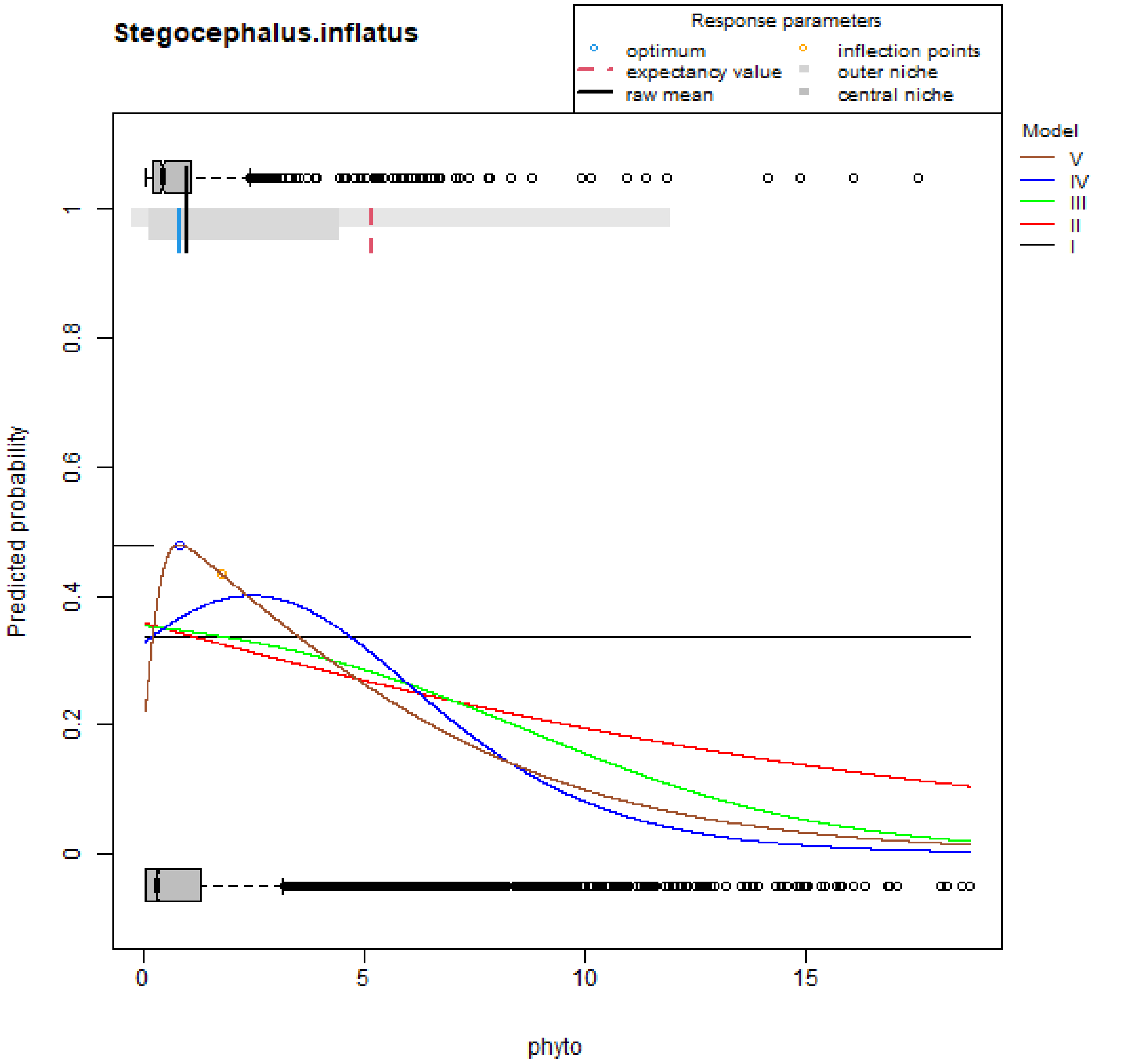

Stegocephalus.inflatus
Response parameters
optimum
expectancy value
raw mean
inflection points
outer niche
central niche
Model
V
IV
III
II
I
Predicted probability
0
0.2
0.4
0.6
0.8
1
0
5
10
15
phyto

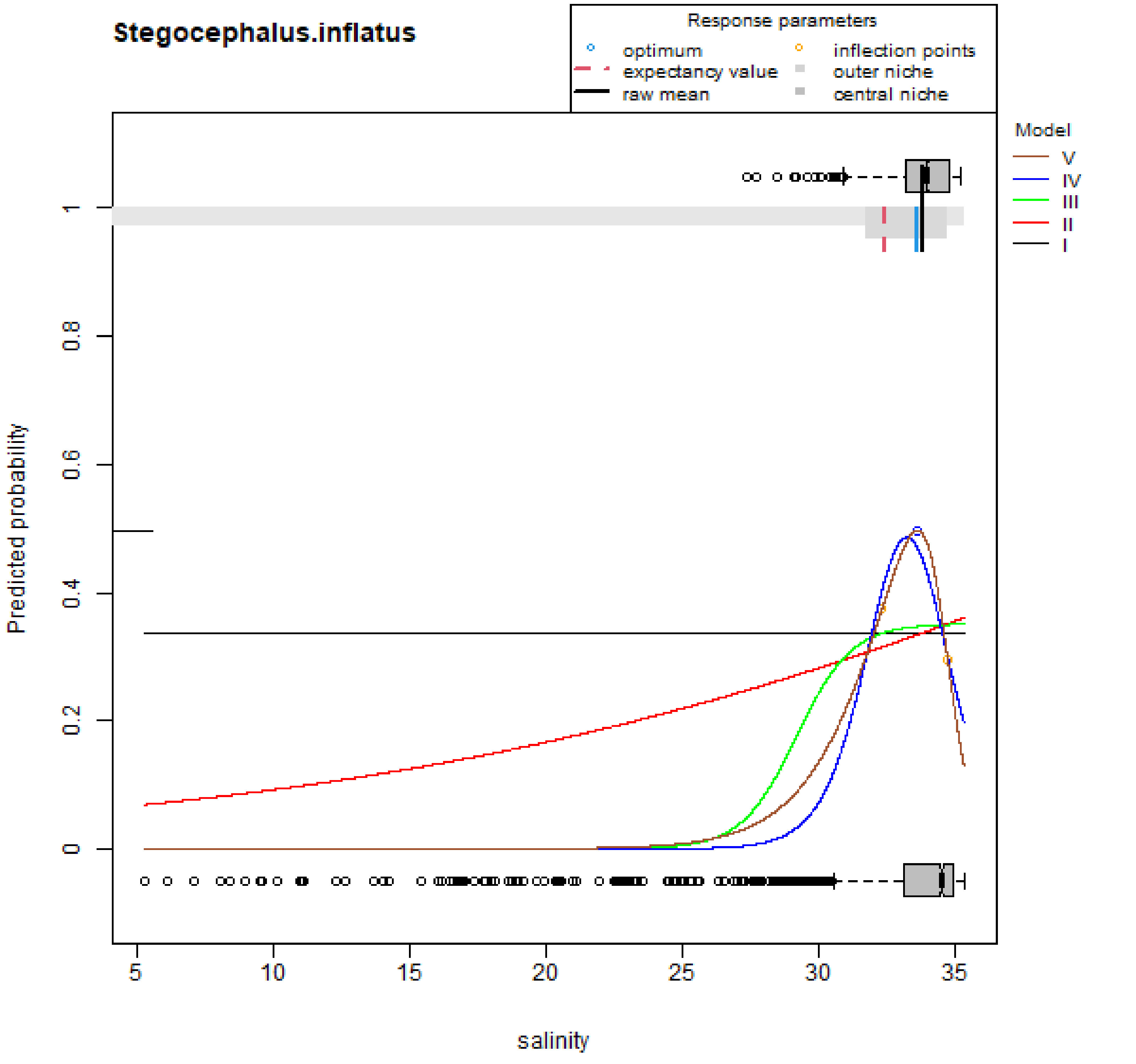

Stegocephalus.inflatus
Response parameters
optimum
expectancy value
raw mean
inflection points
outer niche
central niche
Model
V
IV
III
II
I
Predicted probability
0
0.2
0.4
0.6
0.8
1
5
10
15
20
25
30
35
salinity

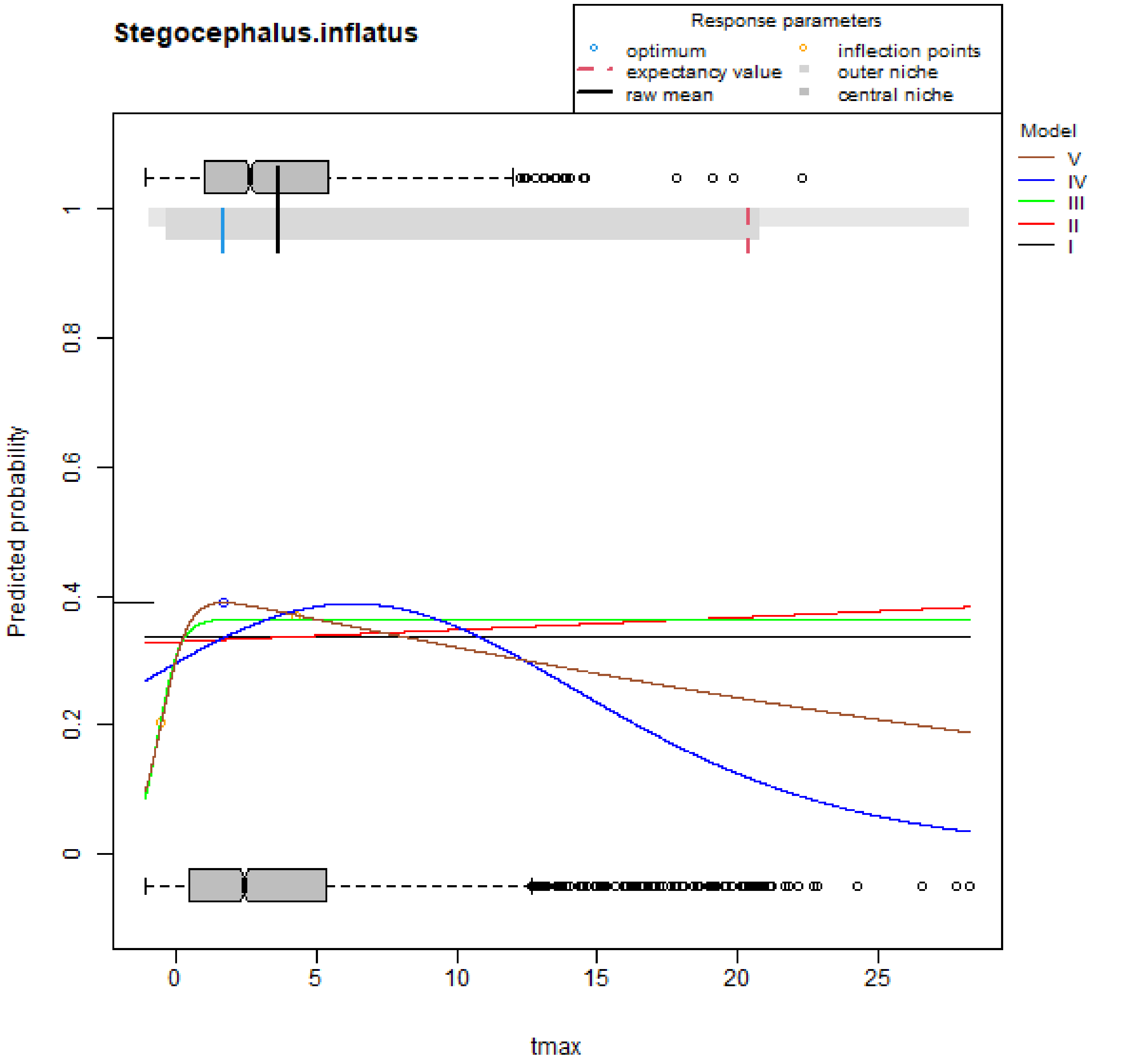

Stegocephalus.inflatus
Response parameters
optimum
expectancy value
raw mean
inflection points
outer niche
central niche
Model
V
IV
III
II
I
Predicted probability
0
0.2
0.4
0.6
0.8
1
0
5
10
15
20
25
tmax

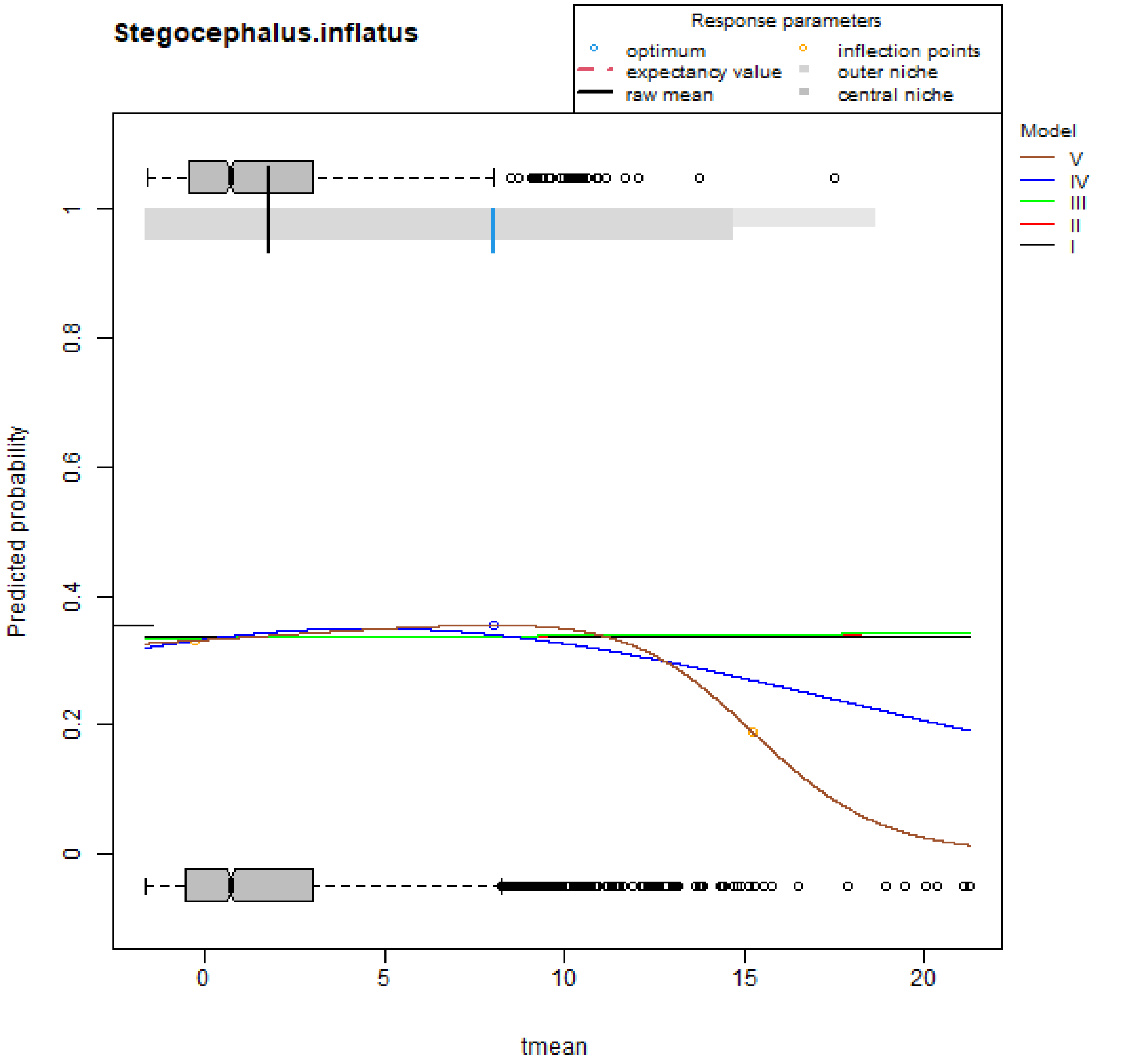
Stegocephalus.inflatus
Response parameters
optimum
expectancy value
raw mean
inflection points
outer niche
central niche
Model
V
IV
III
II
I
Predicted probability
0
0.2
0.4
0.6
0.8
1
0
5
10
15
20
tmean

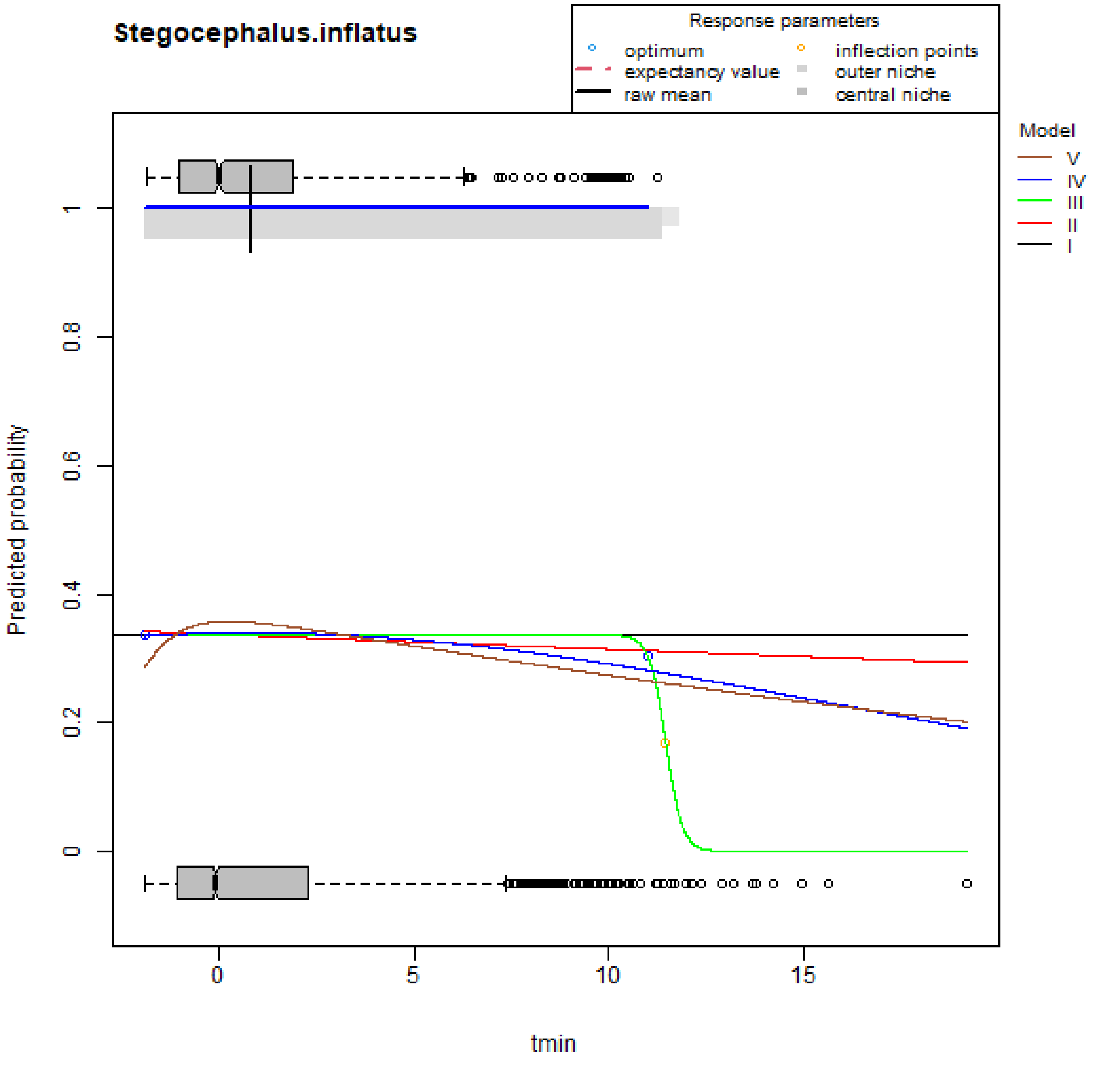

Stegocephalus.inflatus
Response parameters
optimum
expectancy value
raw mean
inflection points
outer niche
central niche
Model
V
IV
III
II
I
Predicted probability
0
0.2
0.4
0.6
0.8
1
0
5
10
15
tmin

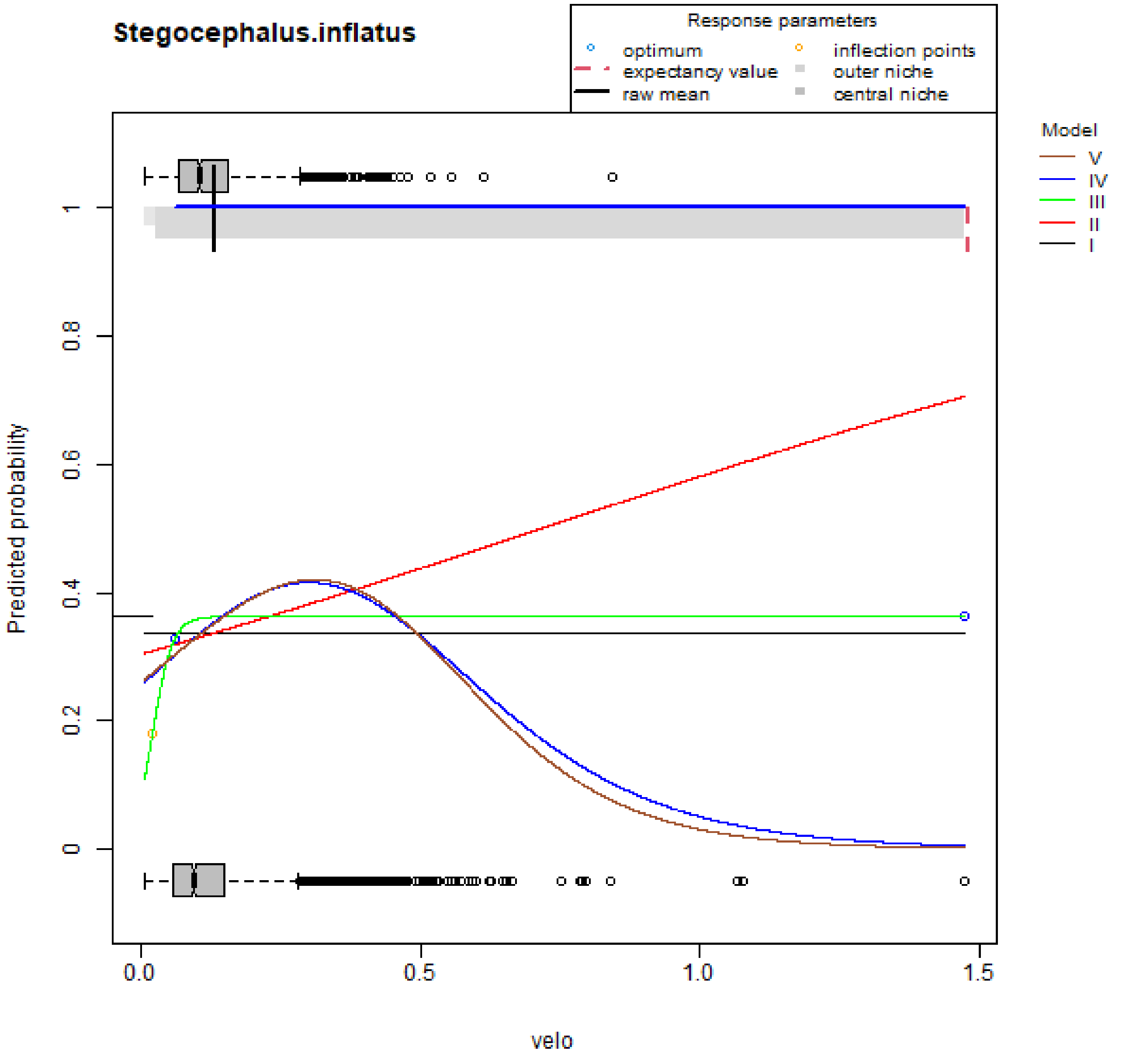

Stegocephalus.inflatus
Response parameters
optimum
inflection points
expectancy value
outer niche
raw mean
central niche
Model
V
IV
III
II
I
Predicted probability
0
0.2
0.4
0.6
0.8
1
0.0
0.5
1.0
1.5
velo
